# EIF1AX Nucleolar Condensates Enhance Susceptibilities for the Management of Endometrial Cancer

**DOI:** 10.1002/advs.202504238

**Published:** 2025-12-17

**Authors:** Chengyu Lv, Zihang Lin, Jiandong Sun, Yuhong Ye, Qibin Wu, Liangzhi Cai, Dabin Liu, Pengming Sun, Shie Wang

**Affiliations:** ^1^ Fujian Maternity and Child Health Hospital College of Clinical Medicine for Obstetrics & Gynecology and Pediatrics Fujian Medical University Fuzhou 350001 P. R. China; ^2^ Key Laboratory of Stem Cell Engineering and Regenerative Medicine of Fujian Province University Fujian Medical University Fuzhou 350122 P. R. China; ^3^ Department of Histology and Embryology School of Basic Medical Sciences Fujian Medical University Fuzhou 350122 P. R. China; ^4^ Department of Pathology The First Affiliated Hospital of Fujian Medical University Fujian Medical University Fuzhou 350005 P. R. China

**Keywords:** 2,5‐MeC, dacinostat, DDX21, endometrial cancer, EIF1AX, synthetic lethal

## Abstract

Endometrial cancer harboring TP53 aberrations presents a significant therapeutic challenge due to the lack of druggable targets. A promising strategy involves inducing senescence in cancer cells followed by targeted elimination using senolytic agents. The preliminary findings indicated that the aberrant subcellular localization of EIF1AX in endometrial cancer is significantly correlated with a poor prognosis. In this study, a compound library is employed to screen for therapeutic agents that induce the nuclear localization of EIF1AX in endometrial cancer cells, followed by a CRISPR library screen to identify senolytic compounds. The results demonstrated that the combination of 2,5‐MeC and dacinostat effectively inhibited tumor growth. Mechanistically, co‐immunoprecipitation mass spectrometry and cleavage under targets and tagmentation sequencing analyses demonstrated that 2,5‐MeC acts as a potent inducer of EIF1AX nucleolar translocation. This translocation promoted senescence by recruiting DDX21 to form nucleolar aggregates, which suppressed rDNA transcription. Additionally, RNA sequencing and antibody array analyses revealed that the synthetic lethality of 2,5‐MeC and dacinostat is mediated through the activation of the JNK/MAPK signaling pathway. Collectively, these findings highlight a novel therapeutic strategy for TP53‐aberrant endometrial cancer.

## Graphical abstract

This schematic illustrates the mechanism of a senolytic strategy in endometrial cancer. EIF1AX facilitates the incorporation of DDX21 into nucleolar condensates, an event that suppresses rDNA transcription and induces cellular senescence. The compound 2,5‐MeC exploits this pathway by promoting EIF1AX nucleolar translocation and condensate formation. Subsequently, the combination of dacinostat and 2,5‐MeC eliminates these senescent cells by inducing apoptosis.

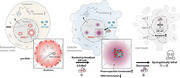

## 1 Introduction

Endometrial cancer (EC) is a common female reproductive system malignancy.^[^ ^1^ ^]^ Although clinical studies demonstrate that chemotherapy combined with bevacizumab (an anti‐VEGF antibody) is beneficial in TP53‐mutated EC,^[^ ^2^ ^]^ outcomes remain inadequate. Additionally, adavosertib, an inhibitor of the G2/M checkpoint kinase WEE1 known to promote apoptosis in TP53‐deficient cells, has shown limited benefit in initial trials.^[^ ^3^ ^]^ Given that TP53 is generally considered undruggable,^[^ ^4^ ^]^ the development of new therapeutic approaches is necessary and urgent.^[^ ^5^ ^]^

Eukaryotic translation initiation factor 1A, X‐linked (EIF1AX), is essential for initiating protein synthesis.^[^ ^6^ ^]^ EIF1AX mutations primarily promote the translation of mRNAs with long 5′UTRs, which often encode proteins involved in cell proliferation, differentiation, angiogenesis, invasion, and metastasis.^[^ ^7^ ^]^ EIF1AX is overexpressed in the nucleocytoplasmic compartment of breast cancer cells and promotes the G1/S phase transition by transcriptionally repressing p21 in a TP53‐independent manner.^[^ ^8^ ^]^ Our prior research has demonstrated differences in the subcellular localization of EIF1AX between normal and malignant endometrial tissue; this aberrant localization is significantly correlated with adverse patient outcomes.^[^ ^9^ ^]^ However, the precise role and mechanism of EIF1AX in endometrial biology and cancer have not been determined. Furthermore, the therapeutic potential of correcting its subcellular localization is unknown.

Sieben et al. have proposed a therapeutic strategy based on the principle of synthetic lethality (SL), in which an initial drug induces a specific vulnerability in tumor cells, and a second agent exploits this vulnerability to selectively eliminate these cells.^[^ ^10^ ^]^ The strategy can be applied to aging cancer cells, leveraging their unique physiological changes.^[^ ^11^ ^]^ However, the application of this approach is limited by the scarcity of effective and low‐toxicity senolytic agents. The development of high‐throughput screening technologies, such as small‐molecule inhibitor screens and CRISPR library screens, has facilitated the discovery of senolytic therapies applicable to cancer treatment. Wang et al. conducted CRISPR‐Cas9‐based senolytic screens and found that cFLIP inhibition increases the susceptibility of senescent cancer cells to apoptosis. Additionally, they demonstrated that DR5 activation primes senescent cells for apoptotic death, thereby enhancing senolytic efficacy.^[^ ^12^ ^]^ Li et al. also identified GSK3 inhibitors as selective agents capable of eliminating senescent liver cancer cells induced by MAT2A inhibition.^[^ ^13^ ^]^ These findings demonstrate that large‐scale screening is a powerful method for developing innovative senolytic treatments for cancer.

In the present study, we screened for compounds that specifically target EIF1AX, with the ultimate goal of developing novel treatments for EC. In particular, using customized compound and CRISPR library screening, we identified 2,5‐MeC as a compound that induces senescence in cancer cells through the nucleolar translocation of EIF1AX. We further evaluated the mechanisms by which EIF1AX mediates cellular senescence in EC cells, including the role of DDX21, as well as combination strategies involving dacinostat for the elimination of senescent EC cells. These results provide a basis for the development of therapeutic approaches for EC.

## 2 Results

### 2.1 EIF1AX Nucleolar Translocation Facilitates the Induction of Senescence in Endometrial Cancer

**Figure 1:**
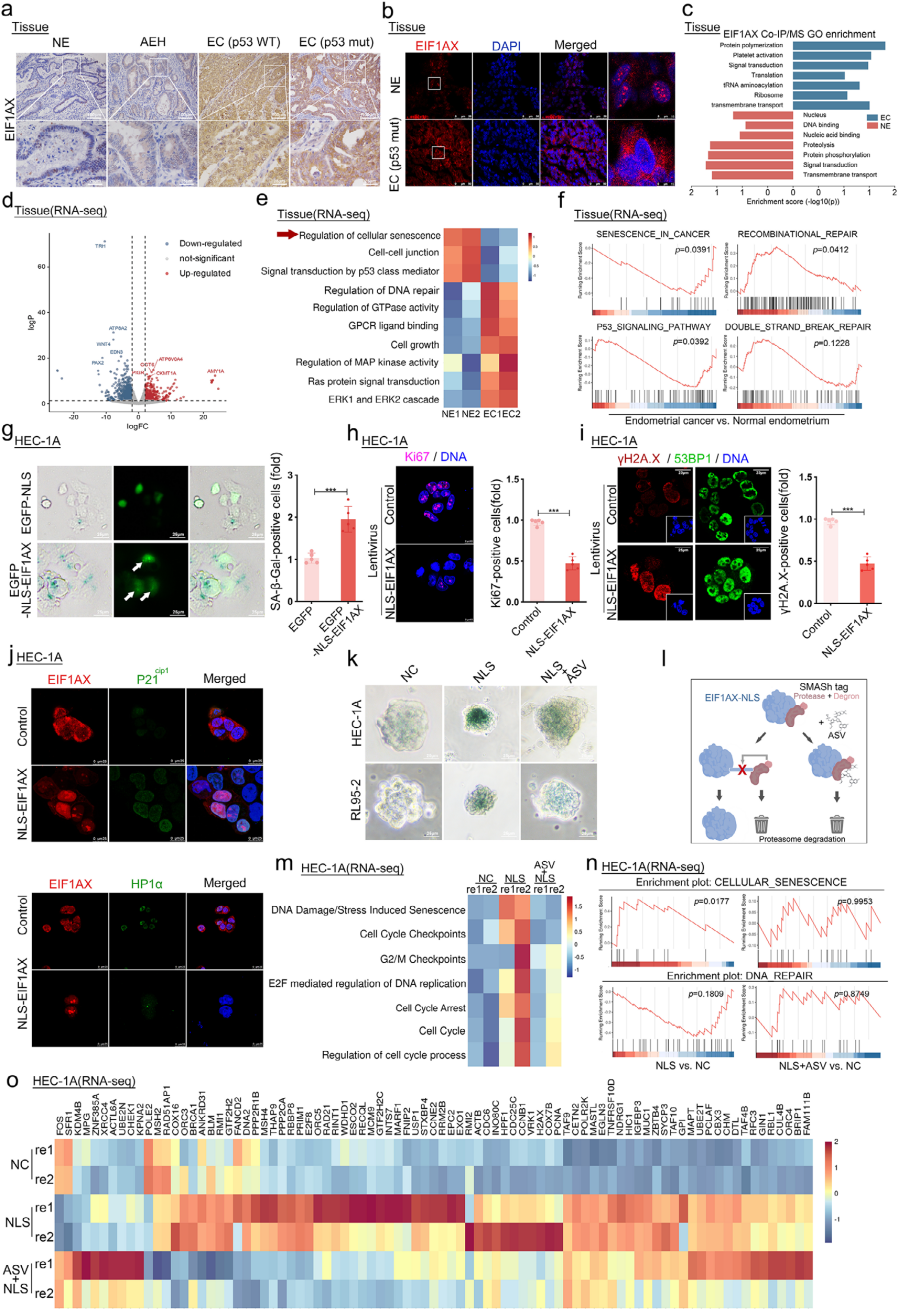
EIF1AX nucleolar translocation promotes cellular senescence in endometrial carcinoma cells. a) Immunohistochemical analysis of EIF1AX expression in endometrial carcinoma (TP53‐WT and TP53‐mut), endometrial atypical hyperplasia (AEH), and normal endometrium. Scale bars: 100 µm; 25 µm (inset). b) Immunofluorescence staining of EIF1AX in TP53‐mutant endometrial carcinoma and normal endometrium. Scale bars: 50 µm; 15 µm (inset). c) GO terms enriched in endometrial carcinoma (EC) and normal endometrium (NE) tissues based on proteomic analysis. d) Volcano plot showing differentially expressed genes in EC compared to NE from RNA‐seq data. e,f) Enriched GO and GSEA terms in EC versus normal control (NC) from RNA‐seq analysis. g–i) SA‐β‐gal staining and immunofluorescence of EIF1AX (g), Ki67 (h), γH2AX, and 53BP1 (i) in HEC‐1A cells. Scale bar: 25 µm. Data are presented as mean ± SD; ***p < 0.001 (Student's *t*‐test). j) Immunofluorescence of p21Cip1, HP1α, and EIF1AX in HEC‐1A cells. Scale bars: 25 µm. k) SA‐β‐gal staining in HEC‐1A and RL95‐2 endometrial cancer stem cell spheroids. Scale bar: 25 µm. l) Schematic of the EIF1AX‐SMASh degradation system. m–o) Heatmap and GSEA of enriched terms in EIF1AX‐WT (NC), EIF1AX‐NLS (NLS), and EIF1AX‐NLS+ASV (NLS+ASV) groups in HEC‐1A cells from RNA‐seq.

Our previous research has indicated that the nucleocytoplasmic translocation of EIF1AX occurs in EC, and cytoplasmic EIF1AX expression is significantly associated with aggressive clinicopathological characteristics and a poorer prognosis in patients with EC.^[^ ^9^ ^]^ However, the functions of EIF1AX in different subcellular compartments remain unclear. In the present study, EIF1AX was highly expressed in the cytoplasm of TP53‐mutant EC (**Figure** **1**a,b; Figure S1a, Supporting Information). Mass spectrometry (MS) results revealed that EIF1AX participated in nuclear and nucleic acid binding in the normal endometrium (NE). In EC, however, its function shifted toward translation and ribosome‐related processes (Figure 1c). A Gene Ontology (GO) analysis of differentially expressed genes (DEGs) between the NE and EC identified enrichment for terms related to “cellular senescence” and “DNA damage response” (Figure 1d,e; Figure S1b, Supporting Information). A Gene Set Enrichment Analysis (GSEA) showed the significant downregulation of the cellular senescence pathway and upregulation of the recombinational repair pathway in TP53‐mutated EC (Figure 1f).

To verify whether EIF1AX nuclear translocation affects cellular senescence, we overexpressed EIF1AX‐NLS in TP53‐mutant EC cell lines (HEC‐1A, RL95‐2, and KLE). Exogenous EIF1AX was primarily localized in the nucleolus (Figure S1c–e, Supporting Information). TP53‐mutant EC cells exhibiting EIF1AX nucleolar translocation displayed distinct senescence‐associated features (Figure 1g–j; Figures S1f–h and S2a–g, Supporting Information), including i) reduced cell proliferation, ii) increased SA‐β‐Gal‐positive cell counts, iii) elevated γH2AX foci, iv) upregulation of p21Cip1 and reduction of HP1α, v) mitochondrial dysfunction, and vi) accelerated senescence in primary EC cells. Additionally, EIF1AX nucleolar translocation promoted senescence in EC stem cell spheroids (Figure 1k; Figure S2h, Supporting Information). Notably, reducing EIF1AX expression in EC cells did not increase cellular senescence (Figure S2i,j, Supporting Information). This finding suggests that the effects of EIF1AX on senescence were independent of its cytoplasmic role in translation initiation.

To further explore whether senescence induced by EIF1AX nucleolar translocation was reversible, we designed an EIF1AX degradation system. Upon nucleolar translocation, EIF1AX tagged with a C‐terminal SMASh epitope can be degraded by treatment with asunaprevir (ASV, 1 µm) (Figure 1l). DEGs between the EIF1AX‐WT and EIF1AX‐NLS+ASV groups were not related to the cell cycle and senescence (Figure 1m–o). These findings suggest that senescence driven by EIF1AX nucleolar translocation was reversible, highlighting its potential as a target for senolytic therapy.

### 2.2 EIF1AX Nucleolar Translocation Exhibits Liquid‐Like Properties

The nucleolus represents a multilayered biomolecular condensate, whose formation through liquid–liquid phase separation (LLPS) facilitates the initial steps of ribosome biogenesis and other processes.^[^ ^14^ ^]^ Given the localization of EIF1AX in the nucleoli of both normal primary endometrial cells and EC cells overexpressing EIF1AX‐NLS (Figures S1c–e and S3a, Supporting Information), we hypothesized that EIF1AX may be associated with LLPS.

**Figure 2:**
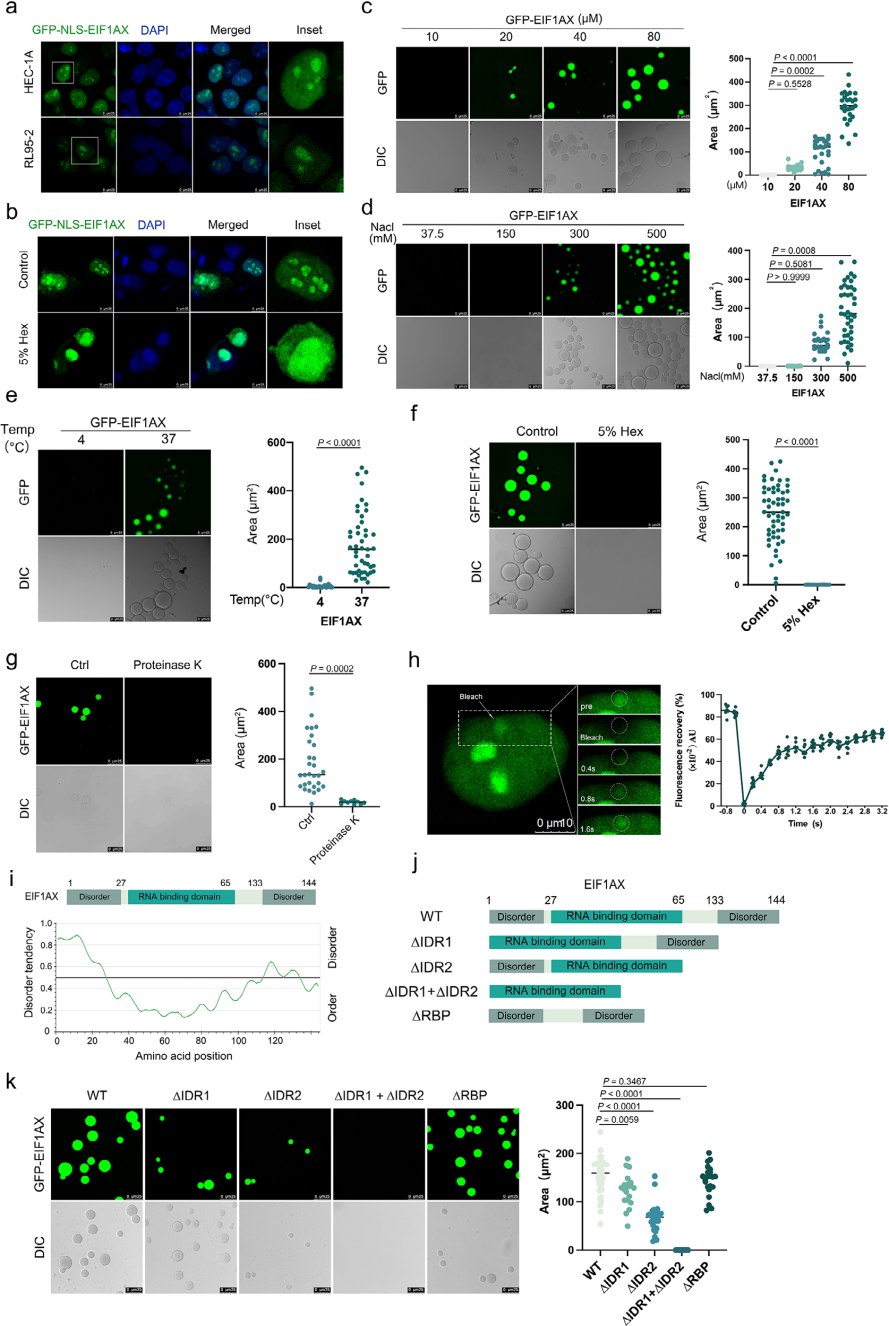
EIF1AX undergoes LLPS in vitro and in vivo. a,b) GFP‐EIF1AX‐NLS formed nucleolar puncta in HEC‐1A and RL95‐2 cells. Cells transfected with GFP‐EIF1AX‐NLS were treated with or without 5% 1,6‐hexanediol (Hex) for 1 min before imaging. Scale bars: 25 µm. c,d) Droplet formation assays with different concentrations of GFP‐EIF1AX‐NLS (c) or NaCl (d). Scale bars: 25 µm. e) Droplet formation of GFP‐EIF1AX‐NLS (40 µm) at 4 and 37 °C in the presence of 500 mm NaCl. Scale bars: 25 µm. f,g) Droplet formation assays of GFP‐EIF1AX‐NLS (40 µm) treated with 5% Hex (f) or proteinase K (g). Scale bars: 25 µm. h) Fluorescence recovery after photobleaching (FRAP) analysis in HEC‐1A cells expressing GFP‐EIF1AX‐NLS. Scale bars: 10 µm. i) Domain architecture and intrinsic disorder propensity of EIF1AX predicted by IUPred. Disorder scores range from 0 to 1, with scores > 0.5 indicating disordered regions. j) Schematic of the domain structure of EIF1AX. k) Droplet formation assays of GFP‐EIF1AX‐WT, ΔIDR1, ΔIDR2, ΔRBP, and ΔIDR1+ΔIDR2 proteins (80 µm) at room temperature with 500 mm NaCl. Scale bar: 25 µm. Data are presented as mean ± SD; *p*‐values were determined by one‐way ANOVA (panels c, d, k) and unpaired two‐tailed Student's *t*‐test (panels e, f, g).

To evaluate this hypothesis, we treated HEC‐1A cells with 5% 1,6‐hexanediol following EGFP‐EIF1AX‐NLS overexpression. The nuclear puncta induced by EIF1AX‐NLS overexpression were disrupted by 1,6‐hexanediol (**Figure** **2**a,b). In addition, droplets formed by GFP‐EIF1AX in vitro were more numerous and larger at higher protein and salt concentrations and high temperatures (Figure S3b, Supporting Information; Figure 2c–e), suggesting that hydrophobic interactions drive droplet formation. Treatment with either 1,6‐hexanediol or proteinase K abolished droplet formation (Figure 2f,g). These LLPS structures exhibited characteristic features, including a spherical morphology, fusion ability, and fluorescence recovery after photobleaching (FRAP).^[^ ^15^, ^16^ ^]^ We therefore performed a FRAP beam‐size analysis using ×63 and ×40 objectives to examine the biophysical properties of GFP‐EIF1AX condensates (Figure 2h).^[^ ^19^, ^20^ ^]^ The τ(×40)/τ(×63) ratio for GFP‐EIF1AX condensates with a diameter of 4 µm was 2.11, consistent with lateral diffusion (Figure S3c–e, Supporting Information). The calculated lateral diffusion coefficient (*D*) was 0.12 ± 0.01 µm^2^·s^−1^, which aligns with those reported for RNA‐binding proteins such as hnRNPA1 and an RNA helicase within nuclear LLPS droplets (Figure S3f, Supporting Information).^[^ ^17^, ^18^ ^]^ These data demonstrate that EIF1AX exhibits highly dynamic behavior, characterized by rapid molecular diffusion within and between condensates as well as within the surrounding nuclear environment. This dynamic movement was indicative of liquid‐like properties, supporting the hypothesis that EIF1AX nuclear condensates form through LLPS.

To identify the phase separation domains of EIF1AX, we constructed five truncated variants to evaluate droplet formation efficiency: ΔIDR1 (lacking the N‐terminal IDR, amino acids 1–27), ΔIDR2 (lacking the C‐terminal IDR, amino acids 133–144), ΔIDR1+ΔIDR2 (lacking both IDRs), and ΔRBP (lacking the RNA‐binding domain, amino acids 27–65) (Figure 2i,j). Immunofluorescence results showed that the deletion of both IDR1 and IDR2 abolished EIF1AX phase separation, whereas removal of the RBP domain had minimal effects (Figure 2k; Figure S3g,h, Supporting Information), indicating that IDR1 and IDR2 were essential for LLPS. To further analyze the disordered regions at the N‐ and C‐termini of human EIF1AX (PDB ID: 4KZY), we used ChimeraX. Among 16 residues, N11 and D142 exhibited high disorder scores. Mutations at N11 and D142 disrupted EIF1AX droplet formation in the nucleolus of EC cells (Figure S3i,j, Supporting Information), underscoring the critical role of these residues in mediating EIF1AX phase separation.

### 2.3 Identifying Proteins that Interact with EIF1AX to form Condensates

**Figure 3:**
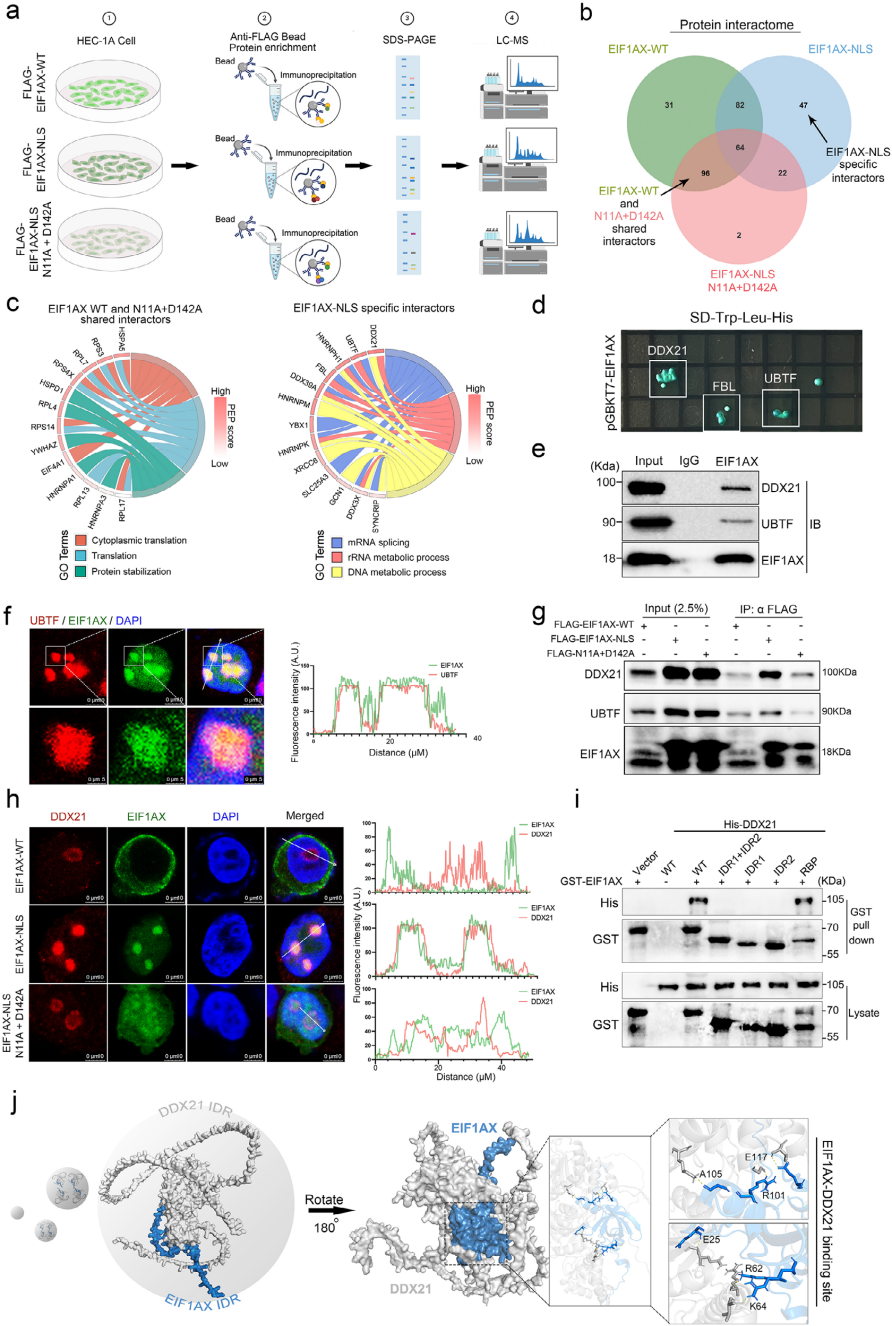
Identification of proteins interacting with EIF1AX within nucleolar condensates. a) Workflow of the mass spectrometry‐based strategy used to identify proteins interacting with EIF1AX‐WT (WT), EIF1AX‐NLS (NLS), and EIF1AX‐NLS N11A/D142A (N11A/D142A). b) Venn diagram showing common and unique interacting proteins among the WT, NLS, and N11A/D142A groups. c) Chord diagrams illustrating enriched pathways among common interactors (left) and group‐specific interactors (right). d,e) Candidate EIF1AX‐binding proteins identified through yeast two‐hybrid screening (d) and co‐immunoprecipitation (Co‐IP) assays (e). f) Immunofluorescence staining of UBTF and EIF1AX in HEC‐1A cells. Scale bars: 10 µm (overview); 5 µm (zoom). Fluorescence intensity profiles along the indicated arrow directions are shown. g) Co‐IP analysis of interactions among EIF1AX‐WT, EIF1AX‐NLS, and EIF1AX‐NLS N11A/D142A in HEC‐1A cells. h) Immunofluorescence staining of DDX21 in HEC‐1A cells expressing WT, NLS, or N11A/D142A EIF1AX. Scale bar: 10 µm. Fluorescence intensity tracings along the indicated arrows are quantified. i) Denaturing GST pull‐down assays showing interactions between His‐DDX21 and GST‐tagged EIF1AX‐WT, IDR1, IDR2, RBP, or IDR1+IDR2 fragments. j) Proposed model: EIF1AX forms phase‐separated condensates with DDX21 via its IDR region, while binding to DDX21 through its RBP domain.

To explore how EIF1AX forms nucleolar condensates and activates cellular senescence, we employed Co‐IP/MS to identify differentially interacting proteins among cells expressing EIF1AX‐WT, EIF1AX with nucleolar translocation (EIF1AX‐NLS), and an LLPS‐deficient nucleolar translocation mutant (EIF1AX‐NLS (N11A/D142A)) (**Figure** **3**a and Table S1, Supporting Information). A Venn diagram revealed 47 proteins uniquely interacting with EIF1AX‐NLS, including DDX21, UBTF, HNRNPH1, and FBL, which were involved in mRNA splicing and rRNA metabolism (Figure 3b–c; Figure S4a, Supporting Information). Yeast two‐hybrid and Co‐IP assays further confirmed that EIF1AX‐NLS binds to FBL, DDX21, and UBTF (Figure 3d,e; Figure S4b,c, Supporting Information), suggesting that rDNA transcription contributes to EIF1AX‐mediated cellular senescence.

To validate this hypothesis, we focused on DDX21, an initiator of rDNA transcription, and UBTF, a component of the RNA Pol I complex (Figure 3f).^[^ ^19^ ^]^ We further demonstrated that N11 and D142 were critical for EIF1AX to recruit DDX21 and UBTF (Figure 3g). Mutation of these residues caused DDX21 to redistribute from the nucleolus to the nucleoplasm (Figure 3h). Similarly, inhibiting EIF1AX phase separation with 1% HEX also impaired DDX21 recruitment (Figure S4d, Supporting Information).

Immunofluorescence staining and Co‐IP confirmed that DDX21 interacts with both EIF1AX and UBTF (Figure S4e–g, Supporting Information). In vitro, DDX21 undergoes phase separation with EIF1AX (Figure S5a–h, Supporting Information). DDX21 inhibition reduced EIF1AX nuclear translocation‐induced senescence in EC cells significantly (Figure S5i, Supporting Information). To assess interaction, we purified GST‐tagged truncated fragments of EIF1AX (IDR1, IDR2, RBP, and IDR1+IDR2) and incubated them with His‐DDX21. Specifically, only the RBP fragment co‐precipitated with DDX21 (Figure 3i; Figure S5j, Supporting Information). These results were further supported by yeast two‐hybrid and mutated fragment GST pull‐down assays (Figure S5k,l, Supporting Information). These findings indicate that the RBP domains of EIF1AX and their key amino acid residues were crucial for mediating interactions with DDX21, underscoring the importance of these regions in recruiting and partitioning DDX21 within EIF1AX condensates (Figure 3j).

### 2.4 EIF1AX‐DDX21 Facilitates the Hijacking of rDNAs to Reduce rDNA Transcription

**Figure 4:**
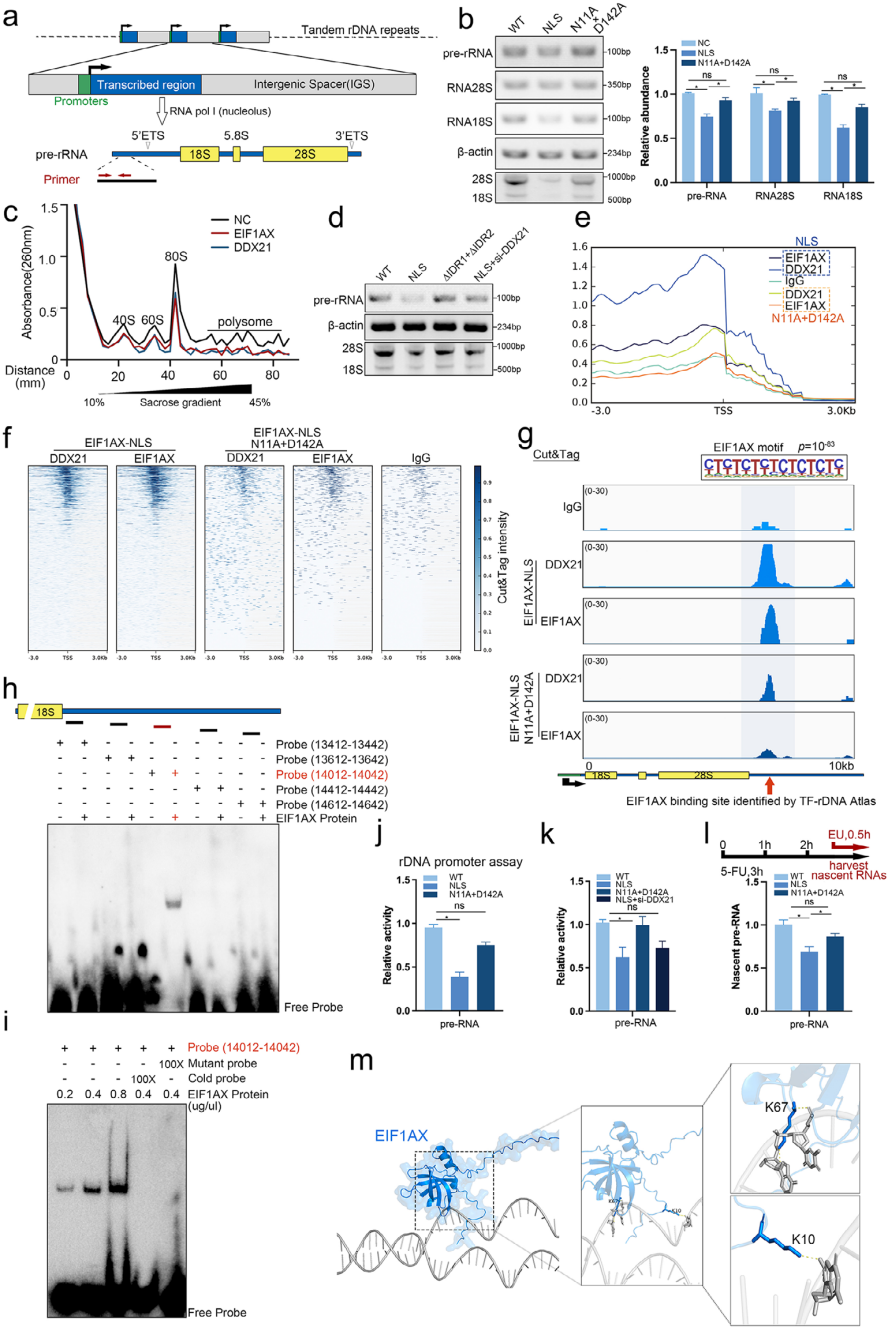
EIF1AX interacts with DDX21 to suppress rDNAs transcription. a) Schematic representation of rDNA organization and transcription. b) Levels of pre‐rRNA, 28S rRNA, and 18S rRNA in HEC‐1A cells expressing WT, NLS, or N11A/D142A EIF1AX. c) Sucrose gradient fractionation (10–45%) of cell lysates monitored by UV absorbance at 254 nm. Peaks correspond to 40S, 60S, and 80S ribosomal complexes. d) Pre‐rRNA levels in HEC‐1A cells expressing WT, NLS, N11A/D142A, or NLS with DDX21 knockdown (NLS+siDDX21). e,f) metaplots (e) and Heatmap (f) and showing enrichment of EIF1AX and DDX21 around transcription start sites (TSSs) of all annotated genes in HEC‐1A cells. g) CUT&Tag‐seq profiles of EIF1AX and DDX21 binding at the rDNA promoter in HEC‐1A cells. The EIF1AX‐binding motif identified at peak summits is shown. h,i) Electrophoretic mobility shift assay (EMSA). j) Luciferase reporter assay. k) CUT&Tag‐qPCR analysis. l) Nascent pre‐rRNA transcription in EIF1AX‐NLS‐expressing cells. m) Proposed model of EIF1AX binding to the 28S/18S promoter regions, as predicted by AlphaFold. Data are presented as mean ± SD; ns: not significant, *p < 0.05, as determined by one‐way ANOVA.

Based on the observed nucleolar translocation of EIF1AX, we evaluated whether its effect on cellular senescence was mediated through roles in nuclear translation,^[^ ^20^ ^]^ alternative splicing,^[^ ^21^ ^]^ or transcription^[^ ^22^ ^]^ within the nucleolus. As shown in Figures S6 and S7 (Supporting Information), EIF1AX nucleolar translocation did not significantly affect nuclear translation or alternative splicing. However, levels of both 28S and 18S rRNA, which were processed from pre‐rRNA (45S), were significantly reduced in EIF1AX‐NLS cells (**Figure** **4**a–c), suggesting that EIF1AX has a critical role in regulating rRNA biogenesis. Consistent with this, the knockdown of DDX21 rescued pre‐rRNA production in EIF1AX‐NLS cells (Figure 4d).

To further investigate the underlying mechanism, we performed CUT&Tag‐seq, which revealed enrichment for both EIF1AX and DDX21 around transcription start sites in EIF1AX‐NLS cells compared with EIF1AX‐NLS (N11A/D142A) cells (Figure 4e,f). Furthermore, by integrating publicly available ChIP‐seq datasets (GSE215313 and GSM1544525), we found that EIF1AX and DDX21 specifically bind to rDNA promoter regions but not to the intergenic spacer sequences of rDNA (Figure 4g; Figure S8a, Supporting Information). Electrophoretic mobility shift assays confirmed that EIF1AX binds directly to a probe corresponding to nucleotides 14 012–14 042 within the rDNA promoter region (Figure 4h,i; Figure S8b,c, Supporting Information). Consistent with these findings, luciferase reporter assays driven by the rDNA promoter showed significantly reduced activity in EIF1AX‐NLS cells (Figure 4j), whereas the knockdown of DDX21 in these cells restored pre‐rRNA production (Figure 4k).

To further determine whether EIF1AX regulates pre‐rRNA abundance at the transcriptional rather than the processing level, we used 5‐fluorouracil (5‐FU) to inhibit pre‐rRNA processing. EU staining showed a significant decrease in nascent pre‐rRNA in EIF1AX‐NLS cells, supporting transcriptional‐level regulation by EIF1AX (Figure 4l). RNA immunoprecipitation followed by qPCR (RIP‐qPCR) confirmed that 47S rRNA was not enriched in EIF1AX pull‐down assays, ruling out direct binding of EIF1AX to ribosomal RNA (Figure S8d, Supporting Information).

These findings highlight the importance of phase separation mediated by EIF1AX‐NLS and DDX21 in inhibiting rDNA promoter binding—a process crucial for the induction of cellular senescence (Figure 4m; Figure S8e, Supporting Information).

### 2.5 2,5‐MeC Promotes Cellular Senescence Through EIF1AX Nucleolar Condensates

**Figure 5:**
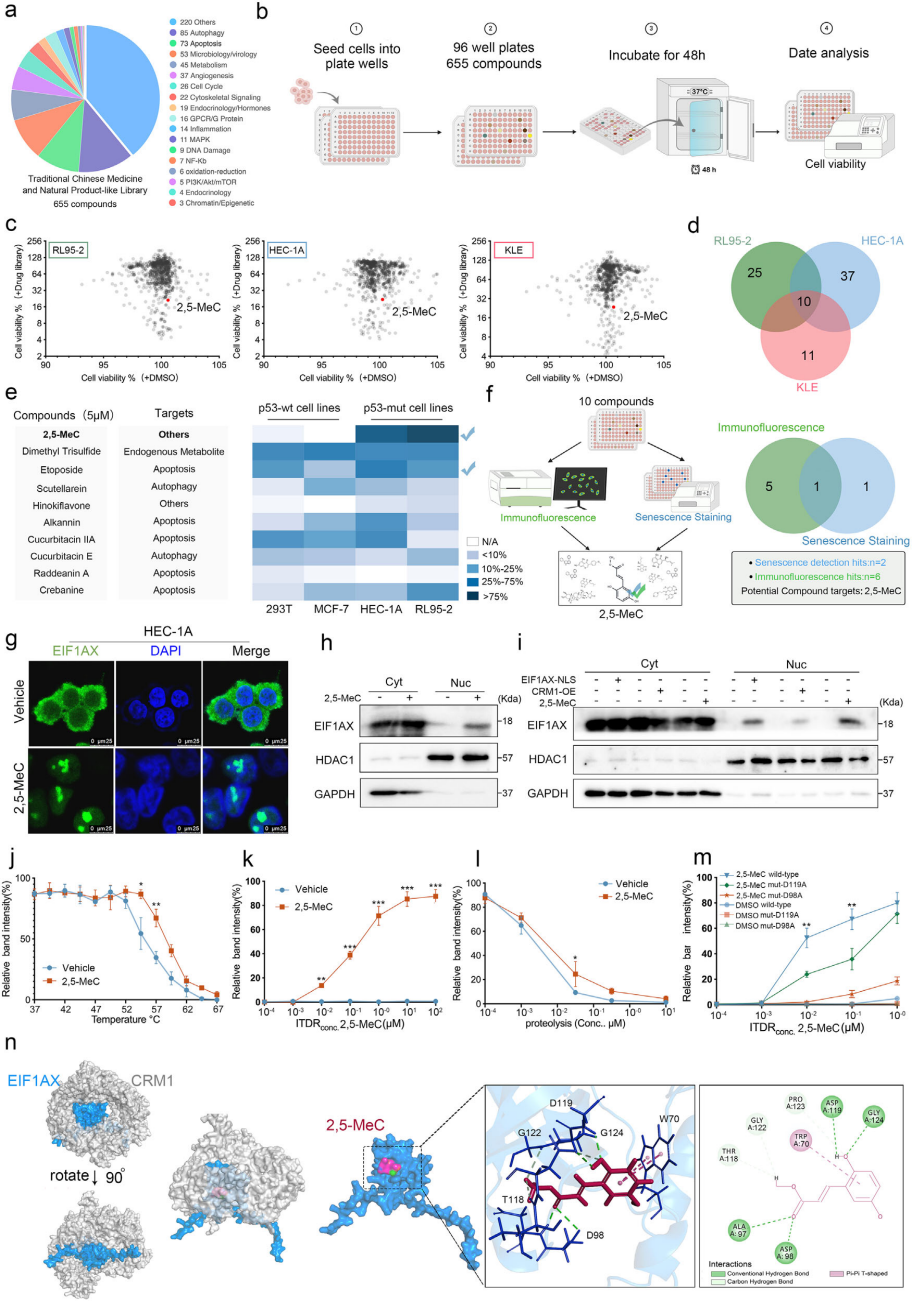
2,5‐MeC promotes EIF1AX nucleolar localization and condensate formation. a) Classification of the compound library according to molecular pathways associated with compound targets. b) Schematic of the compound screening workflow. c) Scatter plot showing cell viability in three endometrial cancer (EC) cell lines treated with compounds from the customized library screen. The red dot represents 2,5‐MeC (see also Figure S9a, Supporting Information). d) Venn diagram summarizing common hits from compound screening across three EC cell lines. e) Heatmap of SA‐β‐gal activity induced by selected compounds (ten hits from the customized screen: 1 µm, 2‐day treatment). f) Screening strategy for compounds inducing EIF1AX nucleolar localization. Venn diagram indicates overlap between immunofluorescence and senescence staining results. g,h) Immunofluorescence (g) and Western blot (h) showing EIF1AX subcellular localization in HEC‐1A cells treated with 2,5‐MeC. Scale bars: 25 µm. i) Western blot of EIF1AX abundance in cytoplasmic (Cyt) and nuclear soluble (Nuc) fractions from HEC‐1A cells with CRM1 overexpression (CRM1‐OE). HDAC1 and GAPDH served as loading controls. j) CETSA thermal stability curves of EIF1AX in HEC‐1A cells treated with 1 µm 2,5‐MeC under thermal denaturation (see also Figure S11a, Supporting Information). k) Isothermal dose‐response (ITDR) analysis in HEC‐1A cells treated with 2,5‐MeC (0.00001–100 µm) and heated at 57 °C (see also Figure S11b, Supporting Information). l) HEC‐1A cells treated with varying protease concentrations in the presence or absence of 2,5‐MeC (see also Figure S11c, Supporting Information). m) ITDR assay in HEC‐1A cells overexpressing a site‐mutated EIF1AX fragment, treated with 2,5‐MeC and heated at 57 °C (see also Figure S11d, Supporting Information). n) Proposed model: 2,5‐MeC inhibits the interaction between EIF1AX and CRM1. Data are presented as mean ± SD; *p < 0.05, **p < 0.01, ***p < 0.001 determined by unpaired two‐tailed Student's *t*‐test (panels j, k, l) and one‐way ANOVA (panel m).

The above results confirm that EIF1AX nucleolar localization can irreversibly induce cellular senescence. To explore the clinical potential of this finding, we screened a library of 655 small‐molecule compounds from the Traditional Chinese Medicine Monomer Library and natural product‐like compound library to identify agents that induce EIF1AX nucleolar localization and cellular senescence (**Figure** **5**a–f; Figure S9a, Supporting Information). Notably, 2,5‐MeC (Methyl 2,5‐dihydroxycinnamate), a compound isolated from the Chinese herb ChiShao (*Paeonia veitchii* Lynch), effectively promoted EIF1AX nucleolar translocation and increased SA‐β‐Gal‐positive cells in HEC‐1A and RL95‐2 cell lines (Figure 5e–h; Figure S9b–g, Supporting Information). Consistent with these findings, 2,5‐MeC inhibited the exportin 1(XPO1/CRM1)‐mediated cytoplasmic accumulation of EIF1AX (Figure 5i; Figure S9h, Supporting Information), in line with earlier reports indicating that CRM1 regulates EIF1AX localization.^[^ ^9^ ^]^ Furthermore, 2,5‐MeC treatment increased γH2AX foci and double‐strand breaks significantly in TP53‐mutant EC cells (Figure S10a–d, Supporting Information).

To determine whether 2,5‐MeC directly targets EIF1AX, we used a cellular thermal shift assay, which detects ligand‐induced stabilization of target proteins against thermal denaturation. We found that 2,5‐MeC stabilized EIF1AX in heat‐denatured HEC‐1A cell lysates (Figure 5j,k; Figure S11a, Supporting Information) but had no stabilizing effect on CRM1 under the same conditions. A drug affinity responsive target stability assay also showed that 2,5‐MeC enhances EIF1AX stability and protects it from protease digestion in HEC‐1A lysates (Figure 5l,m; Figure S11b–d, Supporting Information). Furthermore, pull‐down assays employing biotinylated 2,5‐MeC (bio‐2,5‐MeC) demonstrated a direct interaction with EIF1AX (Figure S11e,f, Supporting Information). Although 2,5‐MeC is an EGFR inhibitor,^[^ ^23^ ^]^ other inhibitors, such as alisertib and sapitinib, did not induce EIF1AX nucleolar translocation or suppress EC cell proliferation (Figure S11g,h, Supporting Information). These results support the conclusion that 2,5‐MeC targets EIF1AX directly to promote its nucleolar translocation and cellular functions, independent of EGFR inhibition (Figure 5n).

### 2.6 Dacinostat Promotes Apoptosis in 2,5‐MeC‐Induced Senescent EC Cells

**Figure 6:**
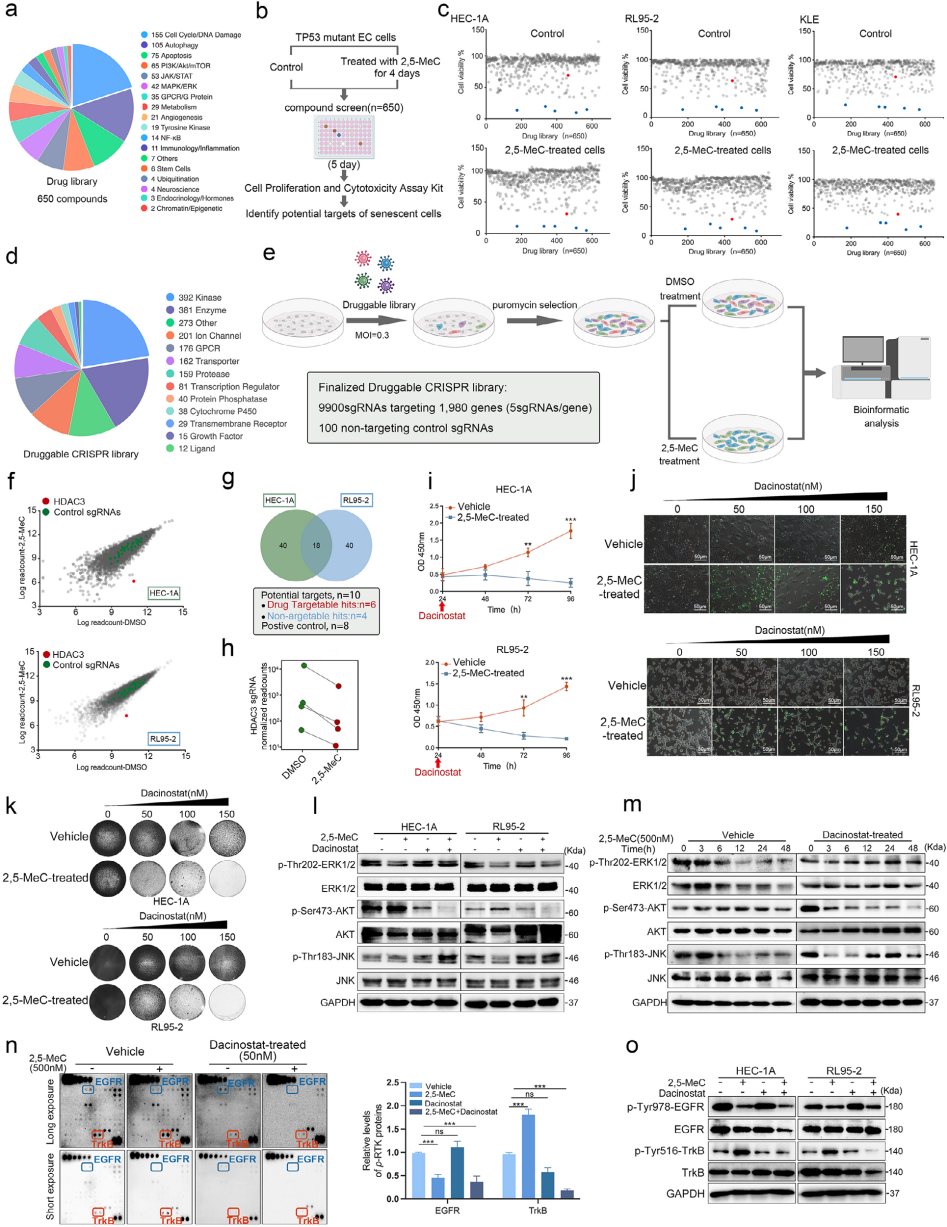
Screening of small‐molecule inhibitors compound library and CRISPR library reveals that 2,5‐MeC enhances cellular sensitivity to Dacinostat. a) Classification of the compound library based on molecular pathways associated with compound targets. b) Schematic representation of the small‐molecule inhibitors compound screening workflow. c) Scatter plot showing the effects of compounds on cell viability. red dot: Dacinostat induced cell death in 2,5‐MeC‐induced senescent cells (see also Figure S12a,c, Supporting Information). d) gRNA content and distribution of protein categories in the druggable genome. e) Schematic of the druggable CRISPR screening methodology. f) Relative abundances of sgRNA barcode sequences. g) Venn diagram showing CRISPR screening results in two endometrial cancer cell lines. h) Read counts of sgRNAs targeting HDAC3 in DMSO‐ and 2,5‐MeC‐treated groups. i) Growth curves of control and 2,5‐MeC‐treated cells sequentially exposed to 1 µm Dacinostat. Data are presented as mean ± SD; **p < 0.01, ***p < 0.001 by unpaired two‐tailed Student's *t*‐test. j) Apoptotic cells visualized by caspase‐3/7 assay after 48 h of Dacinostat treatment in 2,5‐MeC‐induced senescent cells. Scale bars: 50 µm. k) Colony formation assays of HEC‐1A and RL95‐2 cells treated with 2,5‐MeC for 2 days, followed by Dacinostat exposure for 2 days. l) Western blot analyses of 2,5‐MeC‐induced senescent cells treated with Dacinostat for 48 h. m) Western blot analyses of 2,5‐MeC‐induced senescent cells treated with Dacinostat at indicated time points. n) Human Phospho‐Receptor Tyrosine Kinase Array analysis of 2,5‐MeC‐treated HEC‐1A cells after Dacinostat treatment. RTK activation was validated by western blot (Figure S12s, Supporting Information). Data are presented as mean ± SD; ns: not significant, ***p < 0.001, as determined by one‐way ANOVA. o) Activation of receptor tyrosine kinases (RTKs) was validated by Western blot.

To identify drugs exhibiting synergistic effects with 2,5‐MeC in EC cells, we selected 650 small‐molecule inhibitors—covering key signaling pathways, such as cell cycle, autophagy, apoptosis, PI3K‐AKT, and MAPK pathways—from the CTRP (Cancer Therapeutics Response Portal), GDSC (Genomics of Drug Sensitivity in Cancer), and PRISM (Profiling Relative Inhibition Simultaneously in Mixtures) databases^[^ ^24^, ^25^, ^26^ ^]^ along with an sgRNA library targeting the druggable genome to screen for agents targeting EC cells with 2,5‐MeC‐induced senescence (**Figure** **6**a–e; Figure S12a, Supporting Information). Notably, the sgRNA library screen identified HDAC3 as the top regulator promoting apoptosis in 2,5‐MeC‐sensitized EC cells (Figure 6f–h). Consistently, small‐molecule inhibitor screening revealed that dacinostat, an HDAC3 inhibitor, exhibited synthetic lethality with 2,5‐MeC across three EC cell lines (Figure S12b,c, Supporting Information). Further validation showed that dacinostat increases apoptosis in 2,5‐MeC‐treated TP53‐mutant EC cells (Figure 6i–k; Figure S12d,e, Supporting Information). Other HDAC inhibitors, such as panobinostat and entinostat, did not affect 2,5‐MeC‐treated HEC‐1A cells (Figure S12f,g, Supporting Information), suggesting that a context‐specific mechanism contributes to the selective efficacy of dacinostat.

To explore the mechanism by which dacinostat promotes apoptosis in cells with 2,5‐MeC‐induced senescence, we performed RNA sequencing to detect differentially expressed genes following compound treatment. GSEA of the RNA‐seq data for cells treated with 2,5‐MeC and dacinostat revealed the upregulation of gene sets related to MAPK signaling (Figure S12h,i, Supporting Information). Western blot analyses further showed that levels of ERK phosphorylation were lower, whereas levels of JNK phosphorylation were higher in the 2,5‐MeC + Dacinostat group than in the vehicle group (Figure 6l), suggesting that MAPK signaling regulation contributes to the apoptotic effects.

Additionally, 2,5‐MeC reactivated AKT signaling, and this reactivation was suppressed by HDAC inhibitors (Figure 6l). The AKT inhibitors AZD5363 and MK2206 also induced apoptosis in 2,5‐MeC‐treated TP53‐mutant EC cells (Figure S12k–m, Supporting Information). The rapid AKT reactivation in 2,5‐MeC‐induced senescent cells was associated with TrkB activation and EGFR inactivation, both of which were disrupted by dacinostat in HEC‐1A cells (Figure 6m–o). These findings suggest that the pro‐apoptotic effects of dacinostat in senescent cells were mediated through the modulation of the MAPK and AKT signaling pathways.

### 2.7 2,5‐MeC and Dacinostat Synergistically Suppress ECC Proliferation In Vivo

**Figure 7:**
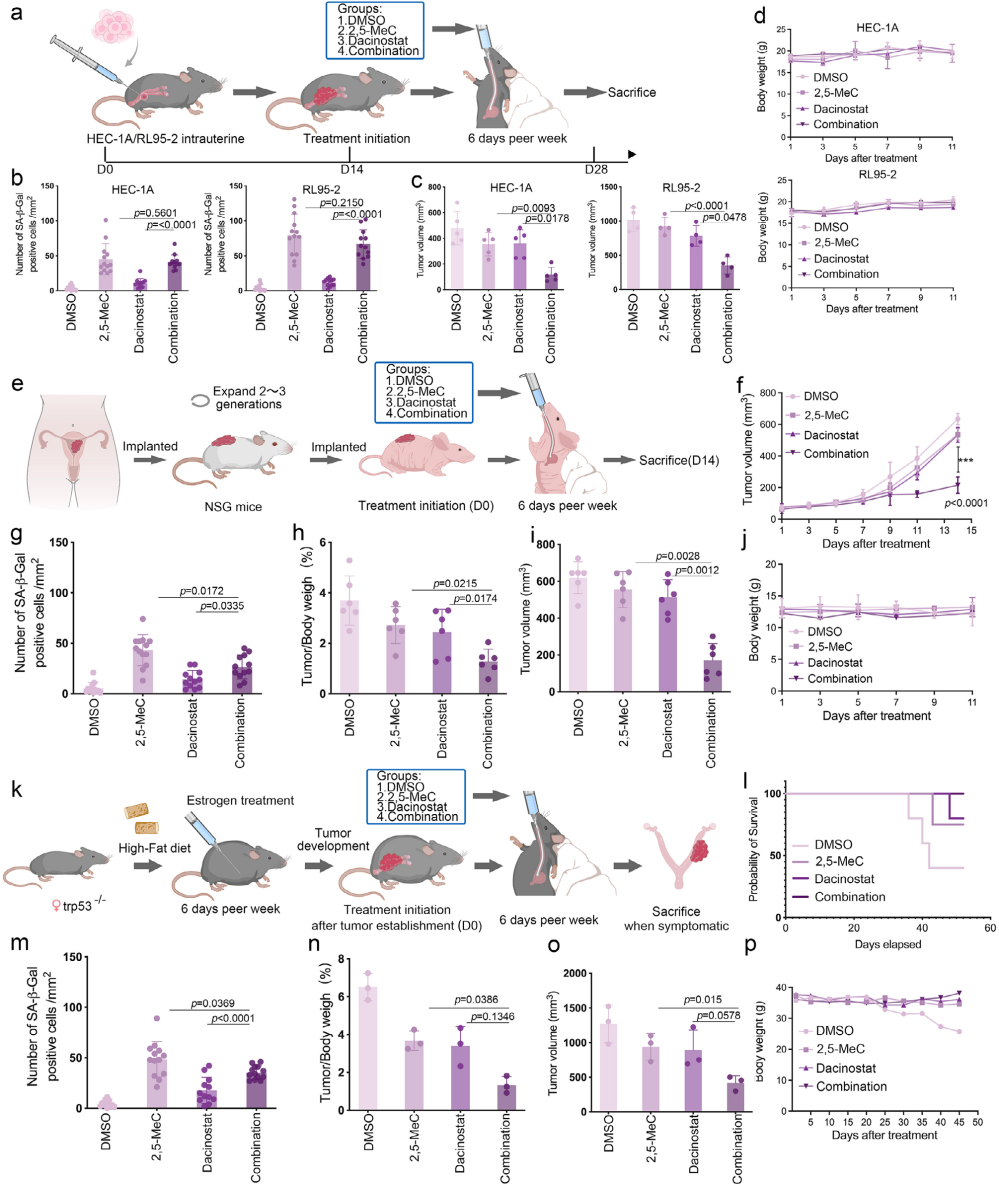
2,5‐MeC and Dacinostat synergistically inhibit ECC proliferation in vivo. a) Schematic of the experimental design for evaluating the anticancer effects of 2,5‐MeC and Dacinostat in a subcutaneous xenograft model. b–d) Tumor volume and weight analysis in HEC‐1A and RL95‐2 xenograft models following combination therapy. e) Schematic of the treatment protocol in a patient‐derived xenograft (PDX) model. f) Tumor growth curves for each treatment group in the PDX model. g–j) Evaluation of antitumor response in the PDX model. k) Schematic of the treatment strategy in a *Trp53*‐deficient endometrial cancer model. l) Survival curves of *Trp53*KO tumor‐bearing mice treated with vehicle (*n* = 6; median survival, 41 days), 2,5‐MeC (*n* = 6; Median survival: Not reached), Dacinostat (*n* = 6; Median survival: Not reached), or the combination (*n* = 6; Median survival: Not reached). m–p) Analysis of therapeutic efficacy in the *Trp53*‐deficient tumor model. Data are presented as mean ± SD; ***p < 0.001 by one‐way ANOVA.

To evaluate the anti‐tumor effects of 2,5‐MeC and dacinostat in vivo, we established xenograft models using HEC‐1A and RL95‐2 cells (**Figure** **7**a). The number of SA‐β‐gal‐positive cells was higher after 2,5‐MeC treatment than in the DMSO group, with no difference in tumor volume. The combination of 2,5‐MeC and dacinostat effectively promoted cellular senescence and reduced the tumor volume (Figure 7b,c; Figure S13a–c, Supporting Information). Moreover, the combined treatment did not cause weight loss or organ damage (Figure 7d; Figure S13d,e, Supporting Information), suggesting a favorable safety profile.

To further validate the synergistic effects of 2,5‐MeC and dacinostat in vivo, we utilized a patient‐derived xenograft (PDX) mouse model of EC (Figure 7e–j; Figure S13f, Supporting Information) as well as a Trp53KO somatic EC model (Figure 7k–p; Figure S14, Supporting Information). In these models, monotherapy with either 2,5‐MeC or dacinostat led to a slight reduction in tumor volume, whereas the combination treatment decreased tumor burden significantly (Figure S14a–f, Supporting Information). These findings indicate that the combination of 2,5‐MeC and dacinostat is a promising therapeutic strategy for EC.

## 3 Discussion

EC is a common malignancy worldwide. The Cancer Genome Atlas (TCGA) defines four prognostic molecular subgroups of EC,^[^ ^27^ ^]^ among which the subgroup with TP53 mutations has the poorest prognosis.^[^ ^28^ ^]^ Current treatment strategies include carboplatin plus paclitaxel, trastuzumab for ERBB2‐amplified tumors,^[^ ^29^ ^]^ and the combination of pembrolizumab with lenvatinib.^[^ ^30^ ^]^ The limited efficacy of existing chemotherapy regimens underscores the urgent need for novel therapies targeting TP53‐mutant cancers. In this study, we demonstrated that 2,5‐MeC induces the nucleolar translocation of EIF1AX, which recruits DDX21 to suppress rDNA transcription and promote cellular senescence. Furthermore, we showed that dacinostat activates JNK/MAPK signaling and effectively induces apoptosis in 2,5‐MeC‐induced senescent EC cells. This combination strategy represents a promising therapeutic approach for TP53‐mutant EC.

rDNA is responsible for producing rRNA and exhibits remarkable evolutionary conservation. The transcription of rDNA generates precursor rRNA, which undergoes multiple processing steps for association with ribosomal proteins, facilitating the assembly of precursor ribosomal subunits.^[^ ^31^ ^]^ The epigenetic landscape of rDNA is intricately linked to its function in aging, where disruptions in rRNA synthesis can activate a DNA damage response. Cellular aging is frequently accompanied by the epigenetic silencing of rDNA and a concomitant decline in rRNA expression.^[^ ^32^ ^]^ Yang et al. have shown that the NML complex promotes rDNA heterochromatin formation and represses rRNA transcription, thereby promoting the initiation and maintenance of senescence.^[^ ^33^ ^]^ DDX21, also known as RNA helicase II, has RNA unwinding activity^[^ ^34^ ^]^ and is involved in pre‐rRNA processing.^[^ ^35^ ^]^ DDX21 interacts with Pol I‐ and Pol II‐transcribed genes to coordinate transcription and rRNA processing. Chen et al. reported that DDX21 molecules assemble into ring‐shaped structures in the nucleolus that suppress pre‐rRNA transcription; their enlargement upon SLERT binding alleviates this repression and enhances pre‐rRNA synthesis.^[^ ^36^ ^]^ Our results revealed that the nuclear translocation of EIF1AX prompts a redistribution of DDX21 from the circumnucleolar region to the nucleolus, resulting in suppressed pre‐rRNA production and the induction of EC cell senescence.

Protein localization in the nucleus and cytoplasm varies between tumor and adjacent non‐tumor tissues, influencing tumor cell behavior through nucleocytoplasmic shuttling.^[^ ^37^ ^]^ Xiao et al. reported that INPP5F is predominantly localized in the nucleus in cells of adjacent non‐tumor tissues, whereas in hepatocellular carcinoma, cytoplasmic localization is more prevalent. The translocation of INPP5F into the cytoplasm, where it interacts with ASPH, facilitates tumor growth in hepatocellular carcinoma.^[^ ^37^ ^]^ Interestingly, EIF1AX has been reported to exert distinct functions in different tumor cells due to differences in its nuclear and cytoplasmic localization. In the cytoplasm of thyroid cancer cells, mutations often increase or aberrantly activate EIF1AX, leading to the preferential translation of specific oncogenic mRNAs and thereby driving tumorigenesis and uncontrolled proliferation.^[^ ^38^ ^]^ In the nucleus of breast cancer cells, EIF1AX inhibits transcription of the cell cycle‐dependent kinase inhibitor p21 (CDKN1A), a key downstream effector of the p53 pathway that blocks cell cycle progression, thereby alleviating cell cycle arrest and promoting abnormal cancer cell proliferation.^[^ ^8^ ^]^ Our previous studies have demonstrated that CRM1 mediates the cytoplasmic localization of EIF1AX in EC, a distinct subcellular distribution from that in the normal endometrium, by recognizing its nuclear localization sequence (LVFK).^[^ ^9^ ^]^ EIF1AX interacts with YBX‐1 to promote c‐Myc translation through the IRES pathway, leading to the suppression of apoptosis in EC cells.^[^ ^39^ ^]^ Our study shows that the nuclear relocalization of EIF1AX in EC cells promotes senescence through the phase separation‐mediated formation of condensates with DDX21. This process requires the intrinsically disordered termini of EIF1AX, where Asn residues (e.g., Asn11) drive phase separation via hydrogen bonding, while Asp residues (e.g., Asp142) confer stability through salt bridges. The N11A/D142A mutant exhibits altered biophysical properties and a loss of phase separation.

Guided by these findings, we screened a traditional Chinese medicine monomer library and discovered 2,5‐MeC. This compound induces EIF1AX nuclear import and senescence by targeting aspartic acid residues 98 and 119. We propose that this interaction is driven by electrostatic complementarity between the cationic compound and anionic residues on the protein.^[^ ^40^, ^41^ ^]^ Furthermore, using the synthetic lethality strategy, we found that dacinostat triggers JNK/MAPK signaling and effectively eliminates 2,5‐MeC‐induced senescent EC cells via apoptosis. The absence of commercially available TP53 wild‐type EC cell lines precluded a comparative treatment analysis with TP53‐mutant lines, a limitation acknowledged in this study.

In summary, these findings expand our understanding of nucleolar biology and its therapeutic potential in cancer. In particular, we identified a novel mechanism by which EIF1AX promotes cellular senescence and provide a therapeutic rationale for targeting aberrant protein localization in synthetic lethal strategies. Future work should focus on optimizing this combination therapy and evaluating its clinical efficacy, particularly for patients with EC with limited treatment options.

## 4 Experimental Section

### Cell Lines

The human EC cell lines HEC‐1A, KLE, and RL95‐2 were obtained from the Cell Bank/Stem Cell Bank of the Chinese Academy of Sciences. Cells were cultured in McCoy's 5A medium or DMEM/F12, as appropriate for each cell line.

### Patient Samples

Between October 21, 2021, and September 30, 2024, 20 patients (aged 46–76 years) who underwent curative surgery at Fujian Maternity and Child Health Hospital in Fuzhou, China (Ethics Approval Code: 2022KYLLRD01034) were enrolled in the study. All tumors carried a TP53 alteration, and none of the patients had received preoperative anticancer treatment. Ethical approval was granted by the Research Ethics Committee of the Medical University School of Obstetrics and Gynecology, and written informed consent was provided by each participant.

### Immunohistochemical Staining

Samples were dewaxed in xylene and rehydrated in graded ethanol. After antigen retrieval, sections were treated with 3% hydrogen peroxide for 1 h, blocked with 3% bovine serum albumin (BSA) for 1 h, and incubated with primary antibodies at 4 °C overnight. The next day, sections were incubated with a secondary antibody and subsequently treated with diluted DAB (3,3′‐diaminobenzidine) solution. The following primary antibodies and dilutions were used: DDX21 (Cell Signaling Technology, Danvers, MA, USA, #59278; 1:1000), PCNA (Cell Signaling Technology, #2586; 1:1000), Cleaved Caspase‐3 (Cell Signaling Technology, #9664; 1:1000), p16 (Sigma‐Aldrich, St. Louis, MO, USA, #SAB56000308; 1:1000), EIF1AX (Sigma‐Aldrich, #HPA002561; 1:1000), and γH2AX (Sigma‐Aldrich, #SAB5700688; 1:1000).

### CRISPR Screen

The library contained ≈10 000 sgRNAs targeting 1980 human genes (with 5 sgRNAs per gene and 100 non‐targeting control sgRNAs) and was obtained from AddGene (Pooled Library, Cat#200013; Watertown, MA, USA). All gene and sgRNA information is provided in Table S2 (Supporting Information). The library was introduced into HEC‐1A and RL95‐2 cells via lentiviral transduction. The transfected cells were cultured with DMSO or 1 µm 2,5‐MeC for 14 days, and sgRNA abundance was determined using Illumina deep sequencing.

### Compound Screens—Induction of Senescence

A customized library of 655 compounds sourced from the Traditional Chinese Medicine Monomer Library (MedChemExpress, HY‐L065) and Natural Product‐like Compound Library (MedChemExpress, HY‐L021L) was used for screening. Three EC cell lines (HEC‐1A, KLE, and RL95‐2) were seeded in 96‐well plates at a density of 1000–2000 cells per well. SA‐β‐gal staining was performed 48 h after treatment.

### Compound Screens—Senescent Cell Killing

A customized library consisting of 650 small‐molecule inhibitors was used for screening (MedChemExpress, Monmouth Junction, NJ, USA; Cat# HY‐L196, HY‐L006, HY‐L009, HY‐L001P, and HY‐L026P). HEC‐1A, KLE, and RL95‐2 cells were treated with 1 µM 2,5‐MeC for 4 days. Both control and 2,5‐MeC‐treated cells were then plated in 96‐well plates at a density of 1000–2000 cells per well. All compounds from the customized library were tested at three concentrations. Cell viability was assessed 48 h after treatment. The relative survival of control and 2,5‐MeC‐induced senescent cells in the presence of each drug was normalized against untreated control data after background signal subtraction. Complete information on all small‐molecule inhibitors is provided in Tables S3 and S4 (Supporting Information).

### SA‐β‐Gal Staining

The samples were fixed for 5 min in a buffer containing 2% (w/v) formaldehyde and 0.2% (w/v) glutaraldehyde. After being washed twice with phosphate‐buffered saline (PBS), the cells were incubated overnight at 37 °C in a staining buffer containing 1 mg mL^−1^ X‐gal (Beyotime Biotechnology, Haimen, China; C0602). SA‐β‐Gal‐stained cells were visualized using an optical microscope, and the percentage of SA‐β‐Gal‐positive cells was calculated.

### Immunofluorescence

Cells were fixed with 4% paraformaldehyde and permeabilized with 0.5% Triton X‐100, followed by blocking with 0.2% BSA. The samples were then incubated overnight with primary antibodies at 4 °C. After washing, the cells were incubated with fluorescently labeled secondary antibodies and counterstained with DAPI. Fluorescence imaging was conducted using a confocal microscope (Leica TCS SP8, Wetzlar, Germany). The primary antibodies were as follows: p53 (Santa Cruz Biotechnology, Dallas, TX, USA; Cat#sc‐126), p21 (Santa Cruz Biotechnology; Cat#sc‐6246) at 1:100 and DDX21, EIF1AX, and γH2AX at 1:200.

### Neutral Comet Assay

Cells were encapsulated in 1% low‐gelling‐temperature agarose, lysed, and subjected to electrophoresis. Comets were visualized using a Zeiss AxioObserver Z1 inverted microscope, and tail moments were analyzed using CASP software.

### Human Phospho‐RTK Array

According to the manufacturer's instructions, Phospho‐RTK arrays (RayBiotech, Peachtree Corners, GA, USA; Cat#AAH‐PRTK‐1) were used to analyze changes in kinase phosphorylation signaling in HEC‐1A cells following treatment with 2,5‐MeC and dacinostat. MAP of Phosphorylation Antibody Array is provided in Table S5 (Supporting Information).

### Animal Model

All animal experiments were approved by the Fujian Medical University Experimental Animal Care Commission (Code of Ethics: IACUC‐FMCHH‐2024‐075). Mice were provided water ad libitum and housed in conventional open‐top cages (five adult mice per cage).

### Animal Model—Xenografts

HEC‐1A and RL95‐2 cells (5 × 10⁶ cells per mouse) were inoculated into the uterine endometrium of 9‐week‐old female BALB/c nude mice (four to five mice per group). Mice were randomly assigned to experimental groups at the time of treatment initation.

### Animal Model—Patient‐Derived Xenograft (PDX) Mouse Model

Samples from patients with EC were minced and transplanted into severely immunodeficient NSG mice and then serially passaged for three generations in both NSG and nude mouse models. Finally, tumor fragments were subcutaneously engrafted into the right flank of 9‐week‐old female BALB/c nude mice (six mice per group). The tumor size was measured every 2 days using a caliper, and volume was calculated using the following formula: volume = (length × width^2^) × 0.5. The protocol was approved by the Institutional Research Ethics Committee of Fujian Maternity and Child Health Hospital (Code of Ethics: 2022KYLLRD01034). Participants volunteered and did not receive compensation. Detailed patient information is provided in Table S6 (Supporting Information).

### Animal Model—Transgenic Mice

*Trp53‐Flox* mice were crossed with progesterone receptor (*PR*)‐Cre mice to generate *PR* ^Cre/+^; *Trp53* ^flox/flox^ (Trp53 conditional knockout, CKO) C57BL/6 mice (Shanghai Model Organisms). Female C57BL/6 mice aged 16–26 weeks (5–10 mice per group) were fed a high‐fat diet for 5–7 months. Subsequently, the mice received intraperitoneal injections of an aqueous estrogen solution (MedChemExpress; Cat#HY‐B0234R) at a dose of 0.1 mg kg^−1^ for 5–7 months. The genotyping primers were as follows: *Trp53*‐Flox (Forward, 5′‐GAGCATGGAAGTAAGACCCCTTCT‐3′; Reverse, 5′‐GACAGGGTTTCTCTATGTAGCCCT‐3′) and *PR*‐Cre (Forward, 5′‐GCGCTAAGGATGACTCTGGTC‐3′; Reverse: 5′‐CCCTTCTCATGGAGATCTGTC‐3′).

Mice from Models A, B, and C were randomized into four groups: vehicle control, 2,5‐MeC (10 mg kg^−1^, oral gavage), dacinostat (5 mg kg^−1^, oral gavage), and the combination of 2,5‐MeC and Dacinostat. Treatments were administered orally 6 days per week for 14 days. Tumor volume, survival curves, and experimental endpoints were recorded for analyses. Tumor volumes were measured using calipers and calculated using the modified ellipsoidal formula: volume = ½ × length × width^2^. All animal experiments were approved by the Institutional Animal Care and Use Committee in accordance with NIH guidelines.

### RNA Sequencing

Cells were collected and subjected to RNA extraction using TRIzol. Samples were sent to Novogene Tech (Beijing, China) for sequencing and primary analyses. A gene set enrichment analysis was performed using DAVID Bioinformatics Resources 6.8.

### Co‐IP/Mass Spectrometry Analysis

Flag‐EIF1AX‐WT, Flag‐EIF1AX‐NLS, and Flag‐EIF1AX‐NLS N11A/D142A plasmids were transfected into HEC‐1A cells using Lipofectamine™ 3000 (Thermo Fisher, Waltham, MA, USA; L3000015). The cells were harvested and lysed in CHAPS lysis buffer (0.3% CHAPS, 40 mm HEPES pH 7.5, 1 mm EDTA, 300 mm NaCl, and 1× protease inhibitor cocktail (Roche, Basel, Switzerland) on ice for 2 h. After centrifugation at 13 000 × *g* for 30 min at 4 °C, the supernatants were collected and incubated with Anti‐Flag M2 Affinity Gel (Sigma–Aldrich; Cat#A2220) overnight at 4 °C with rotation. The beads were then centrifuged at 500 × *g* for 2 min, and bound proteins were eluted using Flag peptide for 2 h. Gel bands were excised, destained, reduced, alkylated, and digested with trypsin. LC‐MS/MS analysis was performed using an Acquity nanoUPLC system coupled to a TripleTOF^™^ 5600 mass spectrometer. Data processing was carried out using PEAKS Studio.

### GST Pull‐Down Assay

The purified GST‐tagged EIF1AX protein (30 µg) was immobilized onto glutathione agarose beads (50 µL bead slurry) for 2 h at 4 °C in a buffer containing 500 mm NaCl. The beads were then incubated with 25 µg of His‐DDX21 protein, which had been purified under either non‐denaturing (50 mm Tris, 500 mm NaCl, 50 mm imidazole, pH 7.5) or denaturing (50 mm Tris, 500 mm NaCl, 8 m urea or 6 m guanidine hydrochloride, 250 mm imidazole, pH 7.5) conditions, for 2 h at 4°C to facilitate binding. After incubation, the beads were washed three times with 1 mL of ice‐cold wash buffer (500 mm NaCl) to remove unbound material. Specifically bound proteins were eluted by boiling in 40 µL of 2× SDS‐PAGE sample buffer for 10 min and subsequently analyzed by western blotting.

### Molecular Docking

The protein structures of EIF1AX and DDX21 were obtained from the Protein Data Bank (PDB ID: 4KZY) and used for a docking analysis using leDock software (http://lephar.com). EIF1AX was docked into DDX21, and binding sites were defined as residues within a 2 Å radius of EIF1AX.

### Cellular Thermal Shift Assay

After treatment with 2,5‐MeC, HEC‐1A cells were collected and divided into 13 groups, followed by heating at temperatures ranging from 37 to 67 °C. After centrifugation, the cell pellets from each group were lysed on ice, and the total protein was extracted and subjected to western blot analyses.

### Drug Affinity–Responsive Target Stabilization Assay

HEC‐1A cells were collected and lysed on ice. The lysate was then divided into equal aliquots and treated with specified concentrations of 2,5‐MeC at 37 °C for 2 h. Subsequently, the aliquots were digested with protease for 30 min and mixed with SDS loading buffer for western blot detection.

### Long‐Term Cell Proliferation Assays (Colony Formation)

Cells were cultured and seeded into six‐well plates at a density of 0.5–4 × 10⁴ cells per well, depending on their growth rate, and maintained in medium containing the specified drugs for 10–14 days, with medium changes performed twice per week. Subsequently, the cells were fixed with 4% formaldehyde in PBS and stained with 0.1% crystal violet in aqueous solution.

### Statistical Analyses

All experiments were performed with at least three independent biological replicates. Data are expressed as the mean ± standard deviation (SD). Comparisons between two groups were performed using Student's *t*‐tests, while multiple group comparisons were performed using one‐way ANOVA followed by LSD or Dunnett's post‐hoc tests, as appropriate. Analyses were implemented in SPSS 17.0 software. A *p*‐value of less than 0.05 was considered statistically significant.

## Conflict of Interest

The authors declare no conflict of interest.

## Author Contributions

C.L., Z.L., J.S., and Y.Y. contributed equally to this work. S.W., P.S., and C.L. contributed to the study conception and design. Material preparation was performed by Z.L., J.S. Data collection was performed by C.L., J.S., and Z.L. Analysis was performed by Z.L., Q.W., L.C., Y.Y., and D.L. The first draft of the manuscript was written by C.L. and all authors commented on previous versions of the manuscript. All authors read and approved the final manuscript.

## Ethical Approval

This study was approved by the local ethics committee of Fujian Maternity and Child Health Hospital (Code of Ethics: 2022KYLLRD01034).

## Supporting information



Supporting Information

Supplemental Tables

## Data Availability

The data that support the findings of this study are available from the corresponding author upon reasonable request.
