# Supplementary material for: EIF1AX Nucleolar Condensates Enhance Susceptibilities for the Management of Endometrial Cancer
Source: Adv Sci (Weinh). 2025 Dec 17;13(12):e04238. doi: 10.1002/advs.202504238 (PMC12948210; doi:10.1002/advs.202504238)
Supplement: Supplementary file 1 — Supporting Information [file ADVS-13-e04238-s002.docx]

**Supplementary Figures**

**
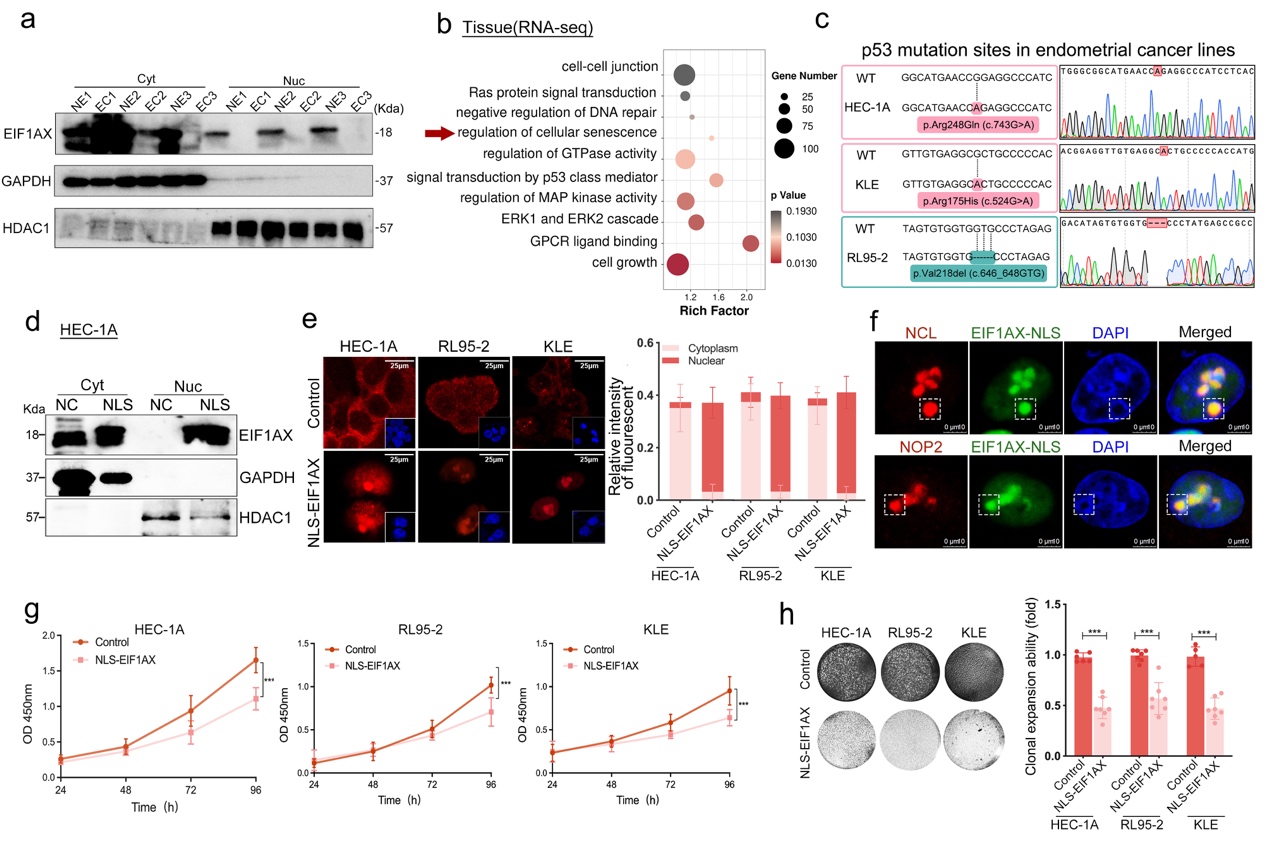
**

**Figure S1. EIF1AX nucleolar condensates related to cellular senescence in Endometrial cancer cells.** (a) Western blot analysis of EIF1AX expression in cytoplasmic (Cyt) and nuclear soluble (Nuc) fractions from TP53-mutant endometrial carcinoma (EC) and normal endometrium (NE). HDAC1 and GAPDH served as loading controls. (b) GO term enrichment analysis of differentially expressed genes between EC and NE based on RNA-seq. (c) Sanger sequencing confirming the TP53 mutation site in endometrial cancer lines. (d) Western blot analysis of EIF1AX expression in cytoplasmic and nuclear fractions of HEC-1A cells. HDAC1 and GAPDH were used as loading controls for nuclear and cytoplasmic fractions, respectively. (e) Immunofluorescence staining of EIF1AX in endometrial cancer cell lines. Scale bars: 25 μm. (f) Immunofluorescence staining of EIF1AX in HEC-1A cells. NOP2 and NCL was used as a nucleolar marker. Scale bars: 10 μm. (g, h) CCK-8 assay (g) and colony formation assay (h) in endometrial cancer cell lines. Data are presented as mean ± SD; ***P* < 0.01, ****P* < 0.001 by unpaired two-tailed Student’s *t*-test.

**
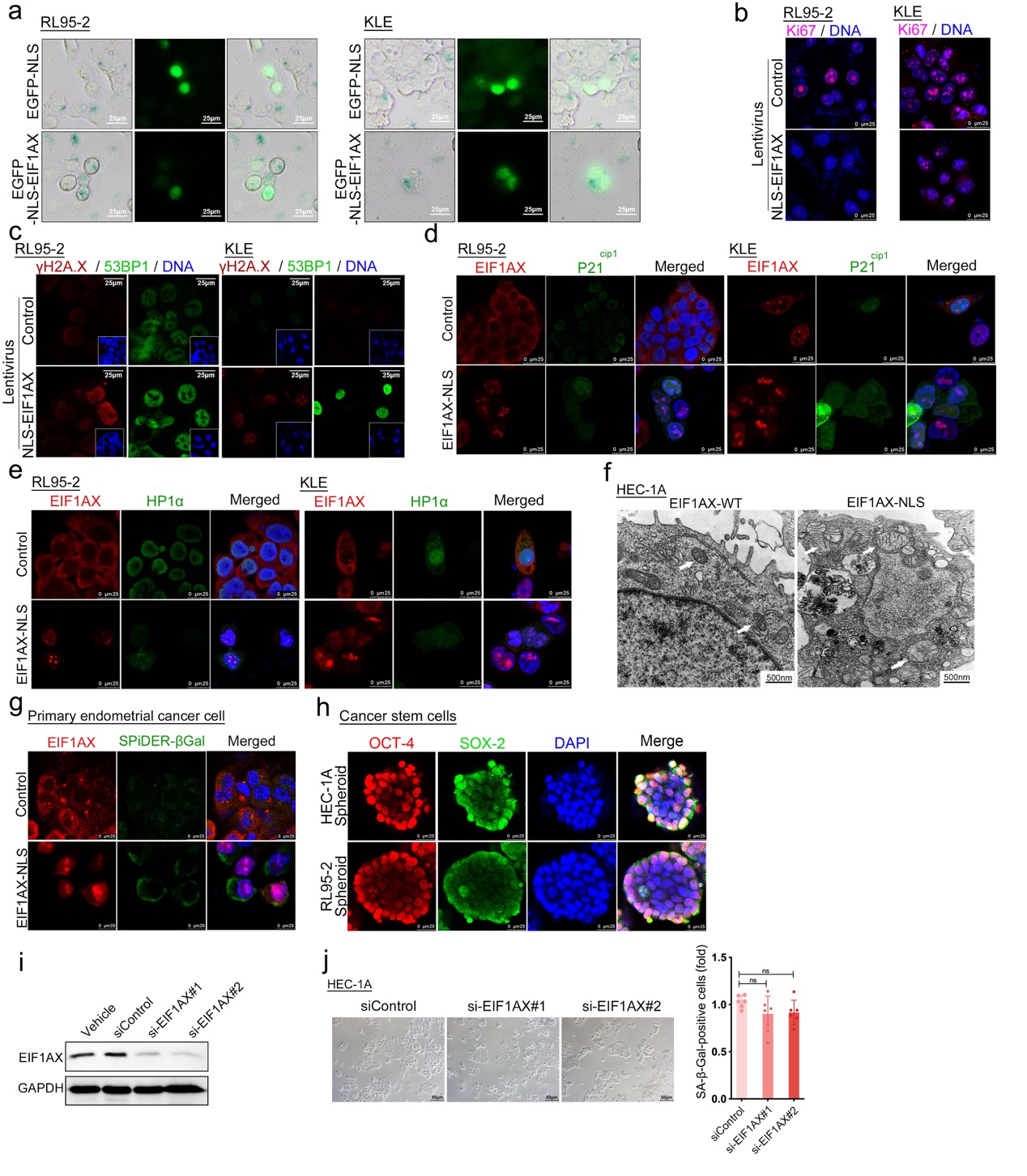
**

**Figure S2. EIF1AX nucleolar condensates promotes cellular senescence in Endometrial cancer cells.** (a) SA-β-gal and immunofluorescence staining of EIF1AX in RL95-2 and KLE cells transfected with plasmids expressing EGFP or EGFP-EIF1AX-NLS. Scale bars: 25 μm. (b-e) Immunofluorescence staining of Ki67, γH2AX, 53BP1, p21Cip1, HP1α, and EIF1AX following EIF1AX-NLS overexpression in RL95-2 and KLE cells. Scale bars: 25 μm­­. (f) Transmission electron microscopy images showing mitochondrial dysfunction (white arrowheads) in HEC-1A cells expressing EIF1AX-WT or EIF1AX-NLS. (g) Immunofluorescence staining of SPIDER-β-gal and EIF1AX in primary endometrial cancer cells. Scale bars: 25 μm. (h) Immunofluorescence staining of stemness-related transcription factors (OCT-4 and SOX2) in HEC-1A and RL95-2 endometrial cancer stem cell spheroids. Scale bars: 25 μm. (i) Western blot analysis of EIF1AX knockdown in HEC-1A cells. (j) SA-β-gal staining of HEC-1A cells treated with EIF1AX siRNA. Scale bars: 50 μm. Data are presented as mean ± SD; ns: not significant (*P* > 0.05) by one-way ANOVA.


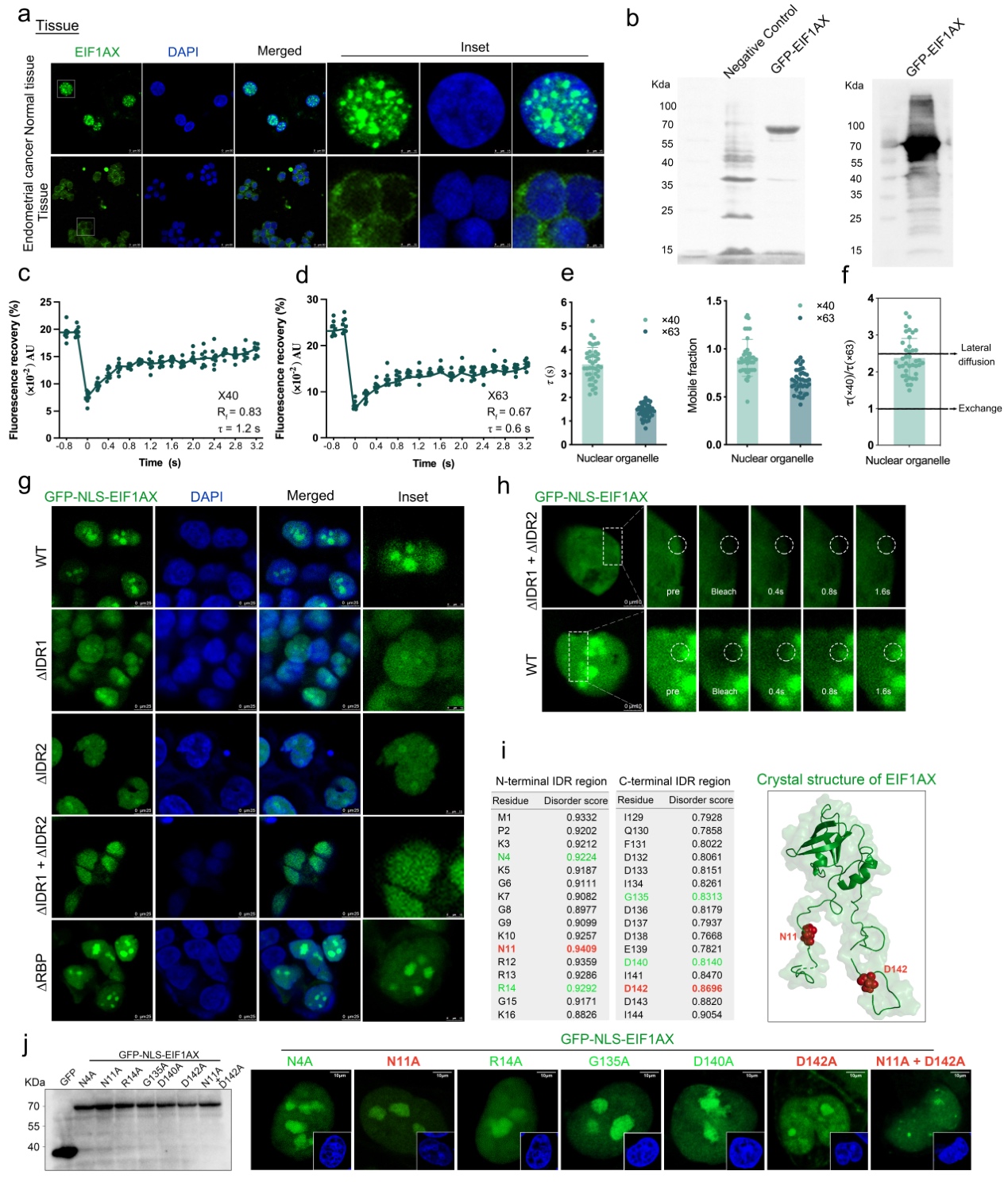


**Figure S3. EIF1AX nucleolar condensates exhibit liquid-like properties.** (a) EIF1AX formed nucleolar puncta in normal endometrial tissues. Scale bars: 50 μm; 15 μm (inset). (b) GFP-EIF1AX-NLS purified from E. coli was analyzed by SDS–PAGE and visualized with Coomassie blue staining. Protein expression was confirmed by western blot. (c, d) Representative fluorescence recovery after photobleaching (FRAP) curves acquired using ×40 (c) or ×63 (d) objectives in organelles larger than the laser beam. (e) Average FRAP values from (c) and (d). Data are presented as mean ± SD.

(f) FRAP beam-size bootstrap analysis. Beam size measurements (n = 33) yielded a ratio of ω²(×40)/ω²(×63) = 2.11 ± 0.05. A τ(×40)/τ(×63) ratio close to this value indicates recovery via lateral diffusion, whereas a ratio near 1 suggests exchange-driven recovery. SEM values of τ ratios were derived from τ measurements (nuclear organelle, n = 35 per objective) shown in (d). (g) Confocal microscopy images of HEC-1A cells expressing GFP-EIF1AX-NLS and various mutants. Scale bars: 25 μm; 15 μm (inset). (h) FRAP analysis in HEC-1A cells expressing GFP-EIF1AX-NLS and ΔIDR1+ΔIDR2. Scale bars: 10 μm (i) Intrinsic disorder profile and crystal structure of EIF1AX. (j) Left: Western blot of HEC-1A cells transduced with GFP-tagged EIF1AX truncation mutants. Right: Immunofluorescence images showing nucleolar puncta formation and localization of GFP-tagged EIF1AX truncation mutants in HEC-1A cells. Scale bar: 10 μm.


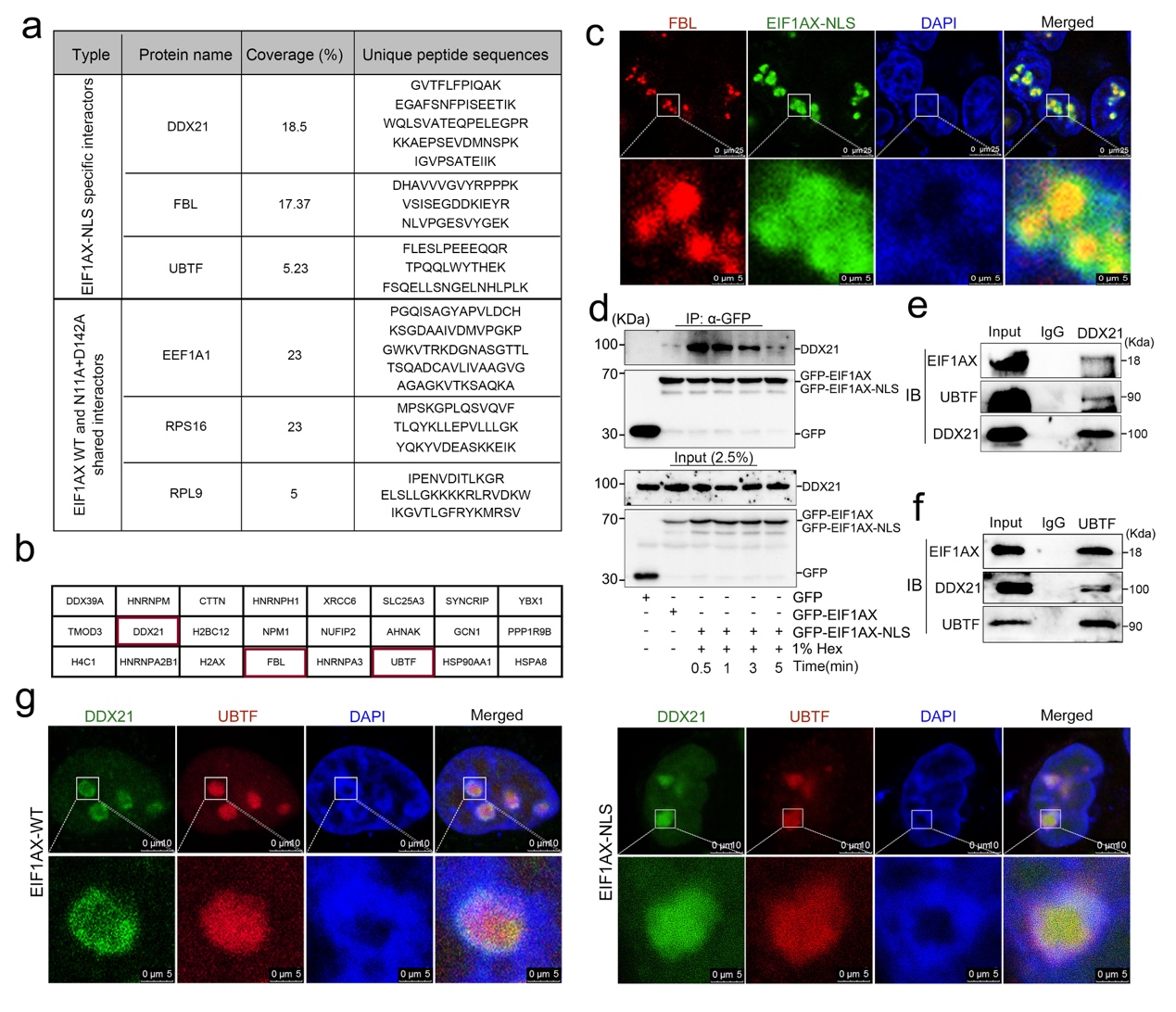


**Figure S4. Identification of proteins interacting with EIF1AX.** (a) Summary of mass spectrometry results identifying DDX21, FBL, and UBTF as EIF1AX-NLS interaction candidates. (b) Summary of EIF1AX-interacting candidates identified by yeast two-hybrid screening. (c) Immunofluorescence staining of FBL and EIF1AX in HEC-1A cells. Scale bars: 25 μm; 5 μm (inset). (d) The levels of DDX21 were assessed after a time course treatment with 1% HEX in HEC-1A cells. (e, f) Co-IP analysis of interactions among EIF1AX, DDX21, and UBTF in HEC-1A cells. (g) Immunofluorescence staining of UBTF and DDX21 in HEC-1A cells transduced with EIF1AX-WT (WT) or EIF1AX-NLS (NLS). Scale bars: 10 μm; 5 μm (inset).

**
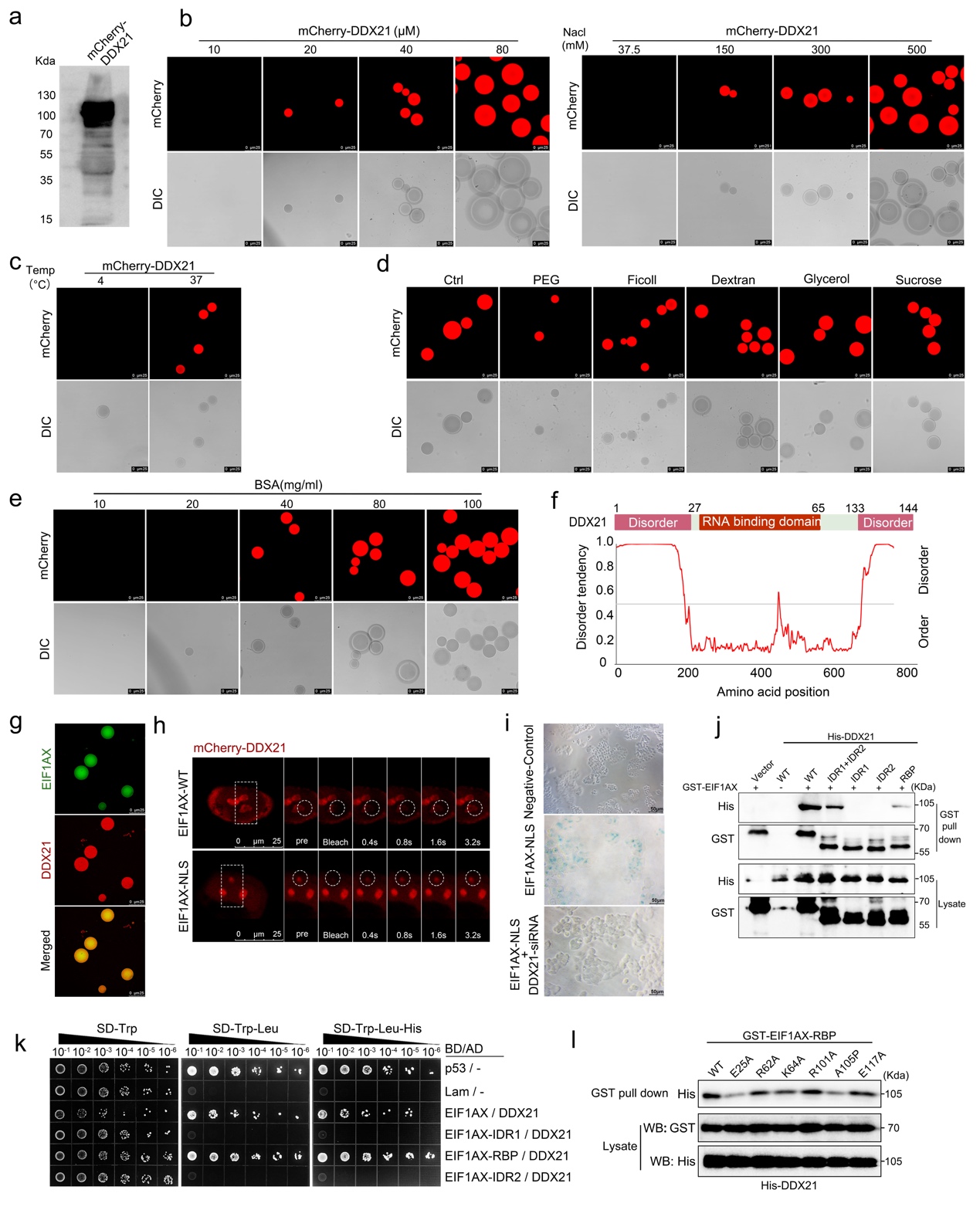
**

**Figure S5. DDX21 undergoes LLPS *in vitro* and *in vivo*.** (a) mCherry-DDX21 purified from E. coli was analyzed by SDS-PAGE and visualized with Coomassie blue staining. (b) Droplet formation of mCherry-DDX21 at room temperature in the presence of different concentrations of NaCl. Scale bar: 25 μm. (c) Droplet formation of mCherry-DDX21 (40 μM) at 4 °C or 37 °C in the presence of 500 mM NaCl. Scale bar: 25 μm. (d) Droplet formation of mCherry-DDX21 (40 μM) in the presence of 10% PEG-8000, Ficoll, dextran, 10% glycerol, or sucrose. Scale bar: 25 μm. (e) Droplet formation of mCherry-DDX21 (40 μM) with varying concentrations of BSA. Scale bar: 25 μm. (f) Domain architecture and intrinsic disorder propensity of DDX21 predicted by IUPred. Disorder scores range from 0 to 1; scores > 0.5 indicate disordered regions. (g) Droplet formation of mCherry-DDX21 (40 μM) and GFP-EIF1AX-NLS (40 μM) at room temperature with 500 mM NaCl. Scale bar: 25 μm. (h) FRAP analysis in HEC-1A cells expressing mCherry-DDX21. (i) SA-β-gal staining of HEC-1A cells treated with EIF1AX-NLS alone or in combination with DDX21 siRNA. Scale bar: 50 μm. (j) Non-denaturing GST pull-down assays showing interactions between His-DDX21 and GST-tagged EIF1AX-WT, IDR1, IDR2, RBP, or IDR1+IDR2 fragments. (k) Yeast two-hybrid assay showing interactions between DDX21 and truncated variants of EIF1AX. (l) Denaturing GST pull-down assay of interactions between His-DDX21 and GST-tagged EIF1AX-RBP variants (WT, E25A, R62A, K64A, R101A, A105P, E117A).


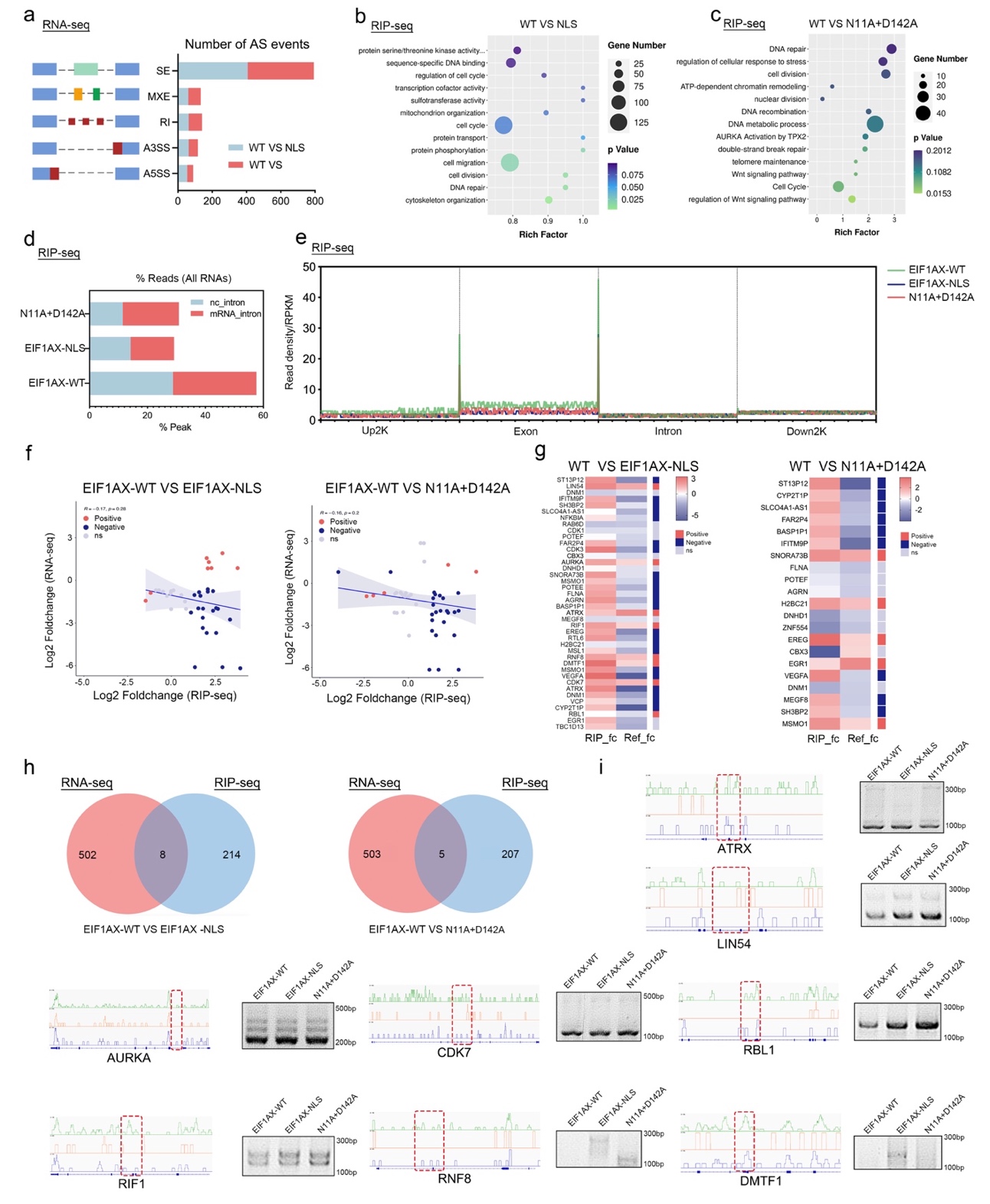


**Figure S6. EIF1AX was not associated with alternative mRNA splicing.** (a) Analysis of alternative splicing (AS) events affected by EIF1AX-NLS expression in HEC-1A cells by RNA-seq. (b, c) GO term enrichment analysis of RIP-seq results in EIF1AX-NLS (b) and EIF1AX-NLS N11A/D142A (c) groups. (d) Distribution of EIF1AX PIP-seq reads mapping to non-coding introns (nc_introns) and mRNA introns in HEC-1A cells. (e) RIP-seq signal profiles around exons showing differential expression between EIF1AX-NLS and EIF1AX-NLS N11A/D142A in HEC-1A cells (FDR < 0.1, fold change ≥ 10%). (f, g) Changes in exon usage between EIF1AX-WT and EIF1AX-NLS (f) or EIF1AX-NLS N11A/D142A (g). (h) Overlap of hits identified by both RNA-seq and RIP-seq in EIF1AX-WT vs. EIF1AX-NLS and EIF1AX-WT vs. EIF1AX-NLS N11A/D142A comparisons. (i) RT-PCR validation of common targets identified by both RNA-seq and RIP-seq (EIF1AX-WT vs. EIF1AX-NLS).


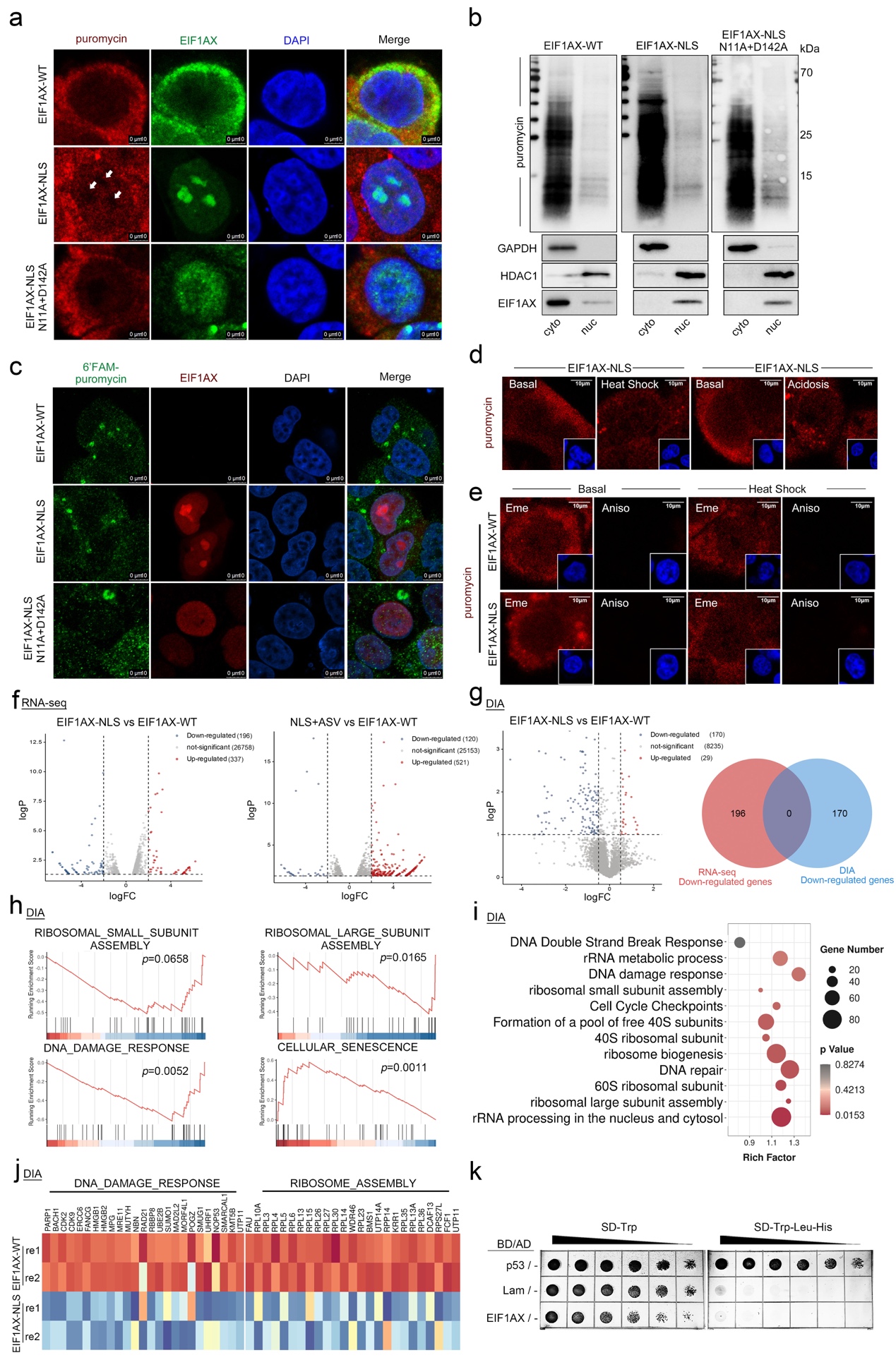


**Figure S7. EIF1AX was not associated with nuclear translation.** (a) Puromycin immunofluorescence was performed in HEC-1A cells transduced with plasmids expressing EIF1AX-WT, EIF1AX-NLS, or EIF1AX-NLS N11A/D142A. Scale bar: 10 μm (b) Western blot analysis of in vitro puromycination in cytosolic (1:5 dilution) and nuclear fractions. Bio-Rad Precision Plus Protein ladder was used. HDAC1 and GAPDH served as loading controls for nuclear and cytosolic fractions, respectively.

(c) FAM-puromycin signal in permeabilized thermally stressed cells under cytosol-free conditions. Scale bar: 10 μm. (d) Puromycin immunofluorescence in intact acidotic or thermally stressed cells. Scale bar: 10 μm. (e) Puromycin signal in thermally stressed cells is abolished by competition with 100:1 anisomycin but remains unaffected in cells untreated with elongation inhibitors such as emetine (200 μM). Scale bar: 10 μm. (f) Volcano plot of up- and down-regulated genes in HEC-1A cells treated with EIF1AX-WT, EIF1AX-NLS, or EIF1AX-NLS+ASV, as identified by RNA-seq. (g) Volcano plot showing differentially expressed genes between EIF1AX-WT and EIF1AX-NLS groups from DIA analysis; Venn diagram indicates overlap of down-regulated genes identified by both RNA-seq and DIA. (h) GSEA of RIBOSOME_ASSEMBLY and DNA_DAMAGE_RESPONSE terms in DIA data from EIF1AX-WT vs. EIF1AX-NLS treated HEC-1A cells. (i) GO term enrichment analysis of DIA data from EIF1AX-WT and EIF1AX-NLS treated HEC-1A cells. (j) Heatmap of log₂ fold changes in gene expression from DIA analysis comparing EIF1AX-WT and EIF1AX-NLS. (k) Validation of EIF1AX transcriptional activation using a yeast one-hybrid assay.


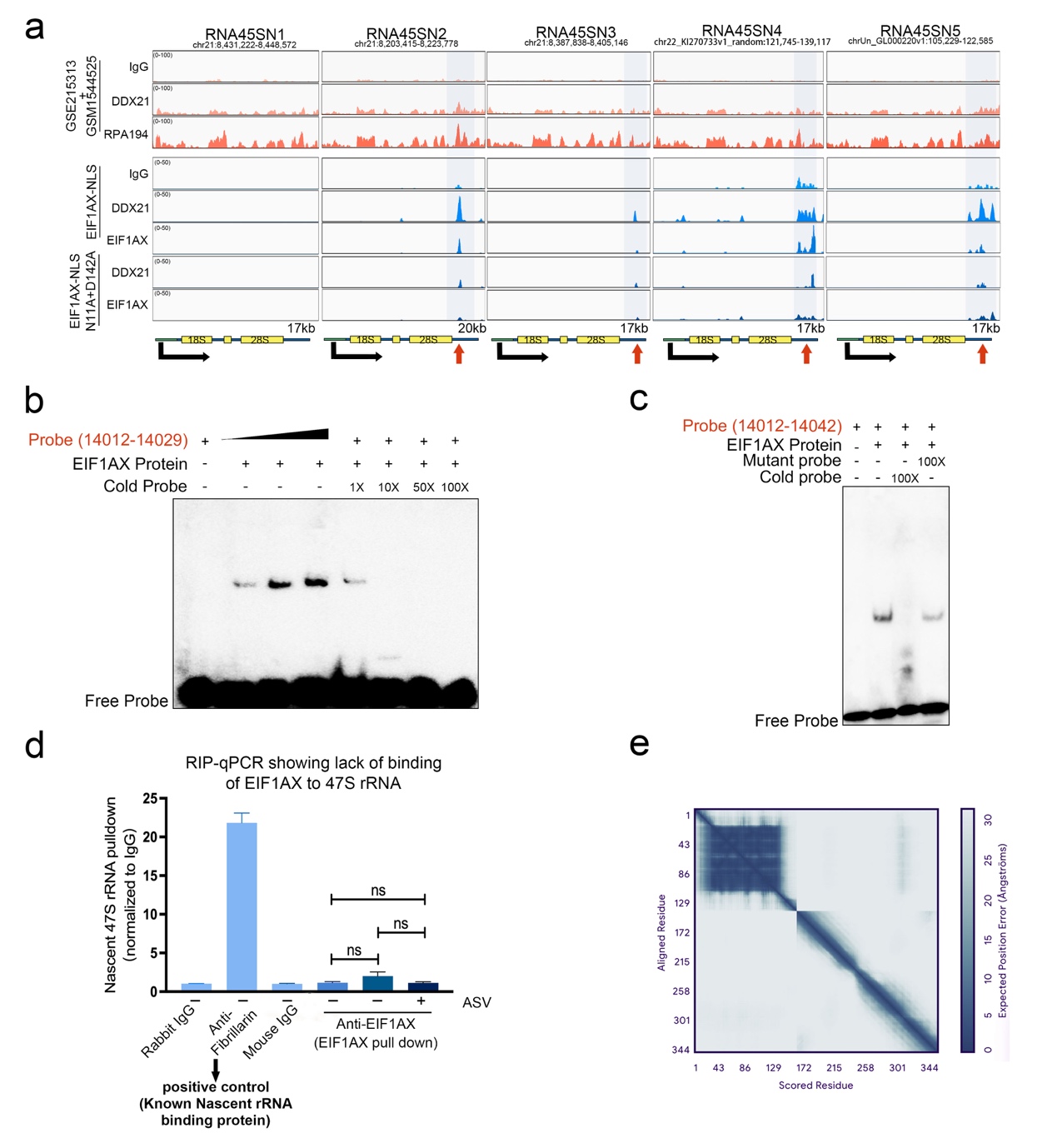


**Figure S8. EIF1AX binds to rDNA promoter**. (a) EIF1AX binds to promoter sequences in multiple rDNA regions. (b-c) Electrophoretic mobility shift assay (EMSA). (d) RIP-qPCR analysis of nascent 47S rRNA in HEC-1A cells. Fibrillarin, a known binder of nascent rRNA, served as a positive control. Data are presented as mean ± SD (n = 3 independent biological replicates). Statistical significance was determined by one-way ANOVA (panels b): ns, not significant (*P >* 0.05). (e) Predicted binding model of EIF1AX to the 28S/18S rDNA promoter regions, as predicted by AlphaFold.


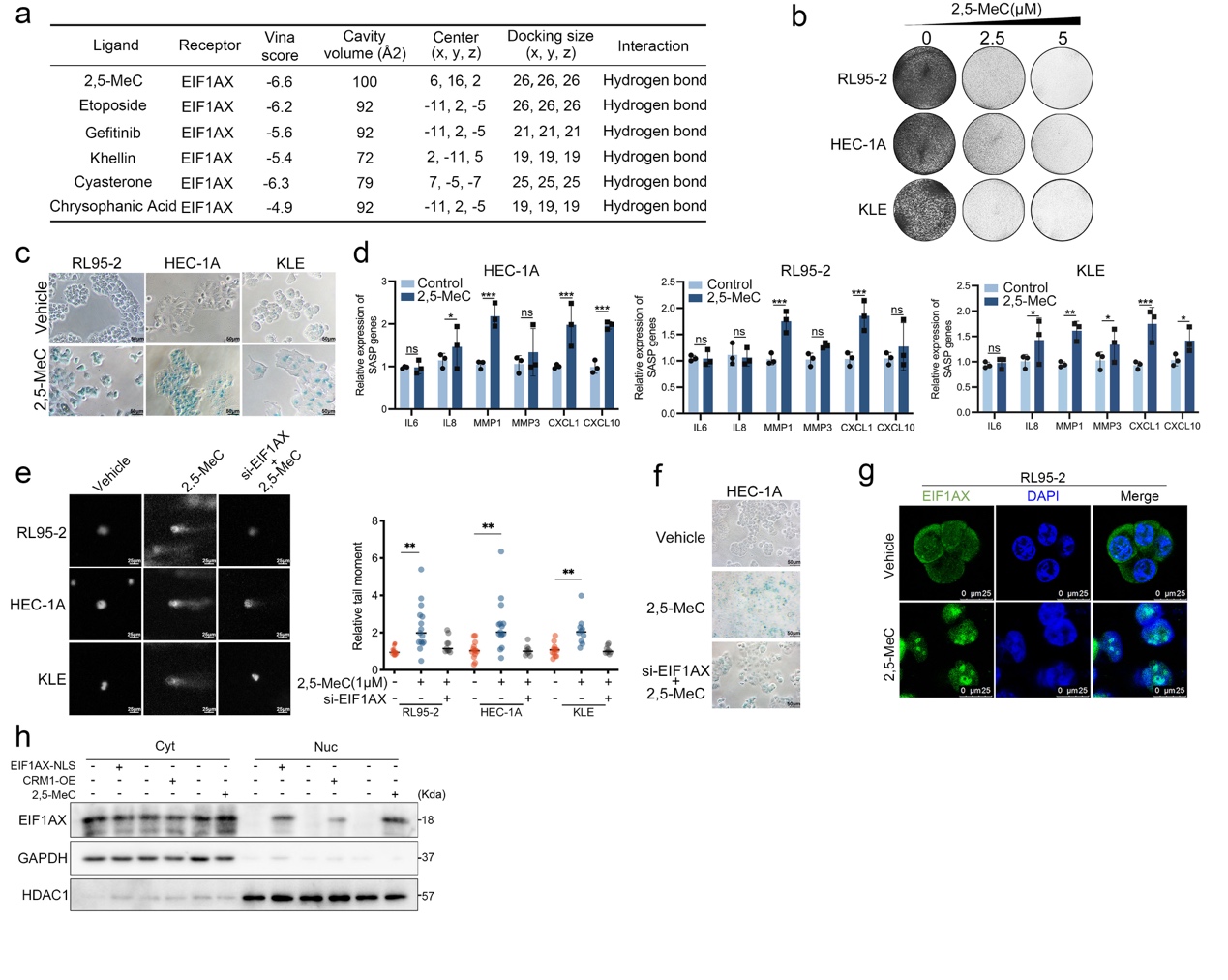


**Figure S9. 2,5-MeC induces senescence in TP53-mutant Endometrial cancer cells.** (a) Predicted binding of candidate drugs to EIF1AX structural pockets using AutoDock Vina. (b) Long-term colony formation assay of endometrial cancer (EC) cell lines treated with indicated concentrations of 2,5-MeC. (c) SA-β-gal staining showing senescence in EC cell lines after 2,5-MeC treatment. Scale bar: 50 μm. (d) qRT‒PCR analysis of senescence-associated secretory phenotype (SASP) gene expression following 2,5-MeC treatment. Data are presented as mean ± SD; *P*-values were determined by unpaired two-tailed Student’s *t*-test. (e) Neutral comet assay in EC cell lines treated with 2,5-MeC or si-EIF1AX for 2 days. Scale bar: 25 μm. Data are presented as mean ± SD; *P*-values were determined by one-way ANOVA. (f) SA-β-gal staining of HEC-1A cells after EIF1AX knockdown and subsequent treatment with 1 μM 2,5-MeC for 2 days. Scale bar: 50 μm. (g) Immunofluorescence staining of EIF1AX in RL95-2 cells treated with 2,5-MeC. Scale bar: 25 μm. (h) Western blot analysis of EIF1AX expression in cytoplasmic and nuclear soluble fractions from RL95-2 cells treated with 2,5-MeC or subjected to CRM1 overexpression (CRM1-OE). Data are presented as mean ± SD (n = 3 independent biological replicates). Statistical significance was determined by unpaired two-tailed Student’s *t*-tests (panels d, e): ns, not significant (*P >* 0.05); **P <* 0.05; ***P <* 0.01; ****P <* 0.001.


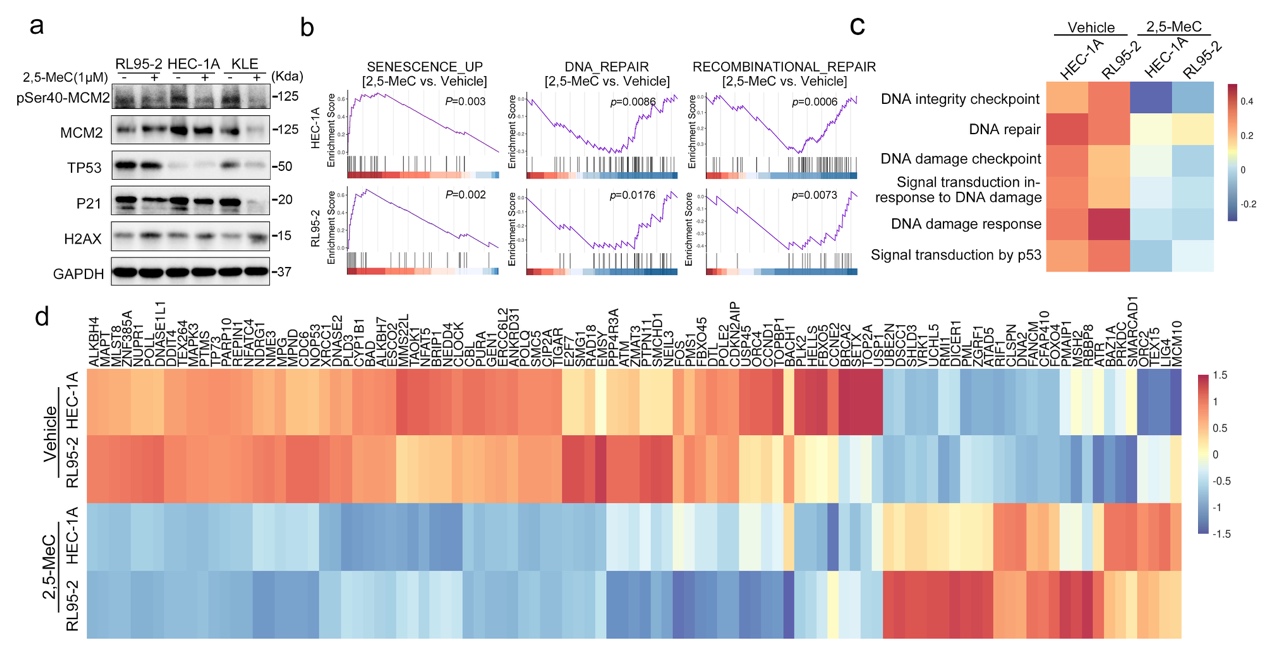


**Figure S10. 2,5-MeC promotes DNA damage accumulation in TP53-mutant endometrial cancer cells.** (a) Western blot analysis of endometrial cancer cell lines treated with 2,5-MeC (1 μM) for 2 days. (b) Gene Set Enrichment Analysis (GSEA) of senescence-associated signatures in RNA-seq data from HEC-1A and RL95-2 cells treated with 2,5-MeC for 2 days. (c) GSEA of RNA-seq data showing enrichment of DNA repair pathways (Recombinational Repair and DNA Repair) in HEC-1A and RL95-2 cells treated with 1 μM 2,5-MeC for 2 days. (d) Heatmap of log_2_ fold changes in gene expression in HEC-1A and RL95-2 cells treated with 2,5-MeC (1 μM, 2 days).


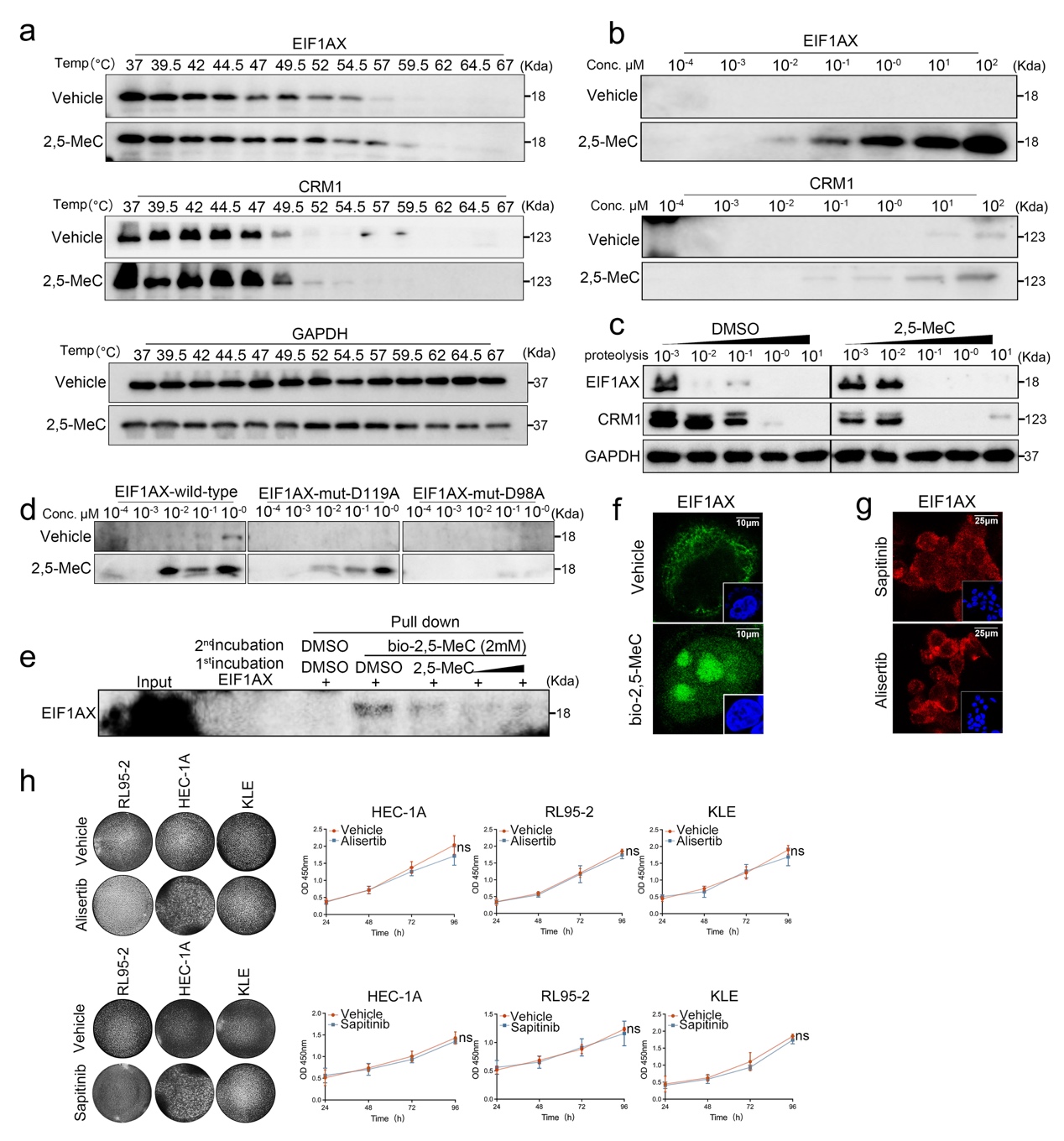


**Figure S11. EIF1AX was a target of 2,5-MeC.** (a) Western blot analysis of EIF1AX and CRM1 expression in HEC-1A cells treated with 1 μM 2,5-MeC under thermal denaturation. GAPDH served as a loading control (see also Figure 5j). (b) Western blot of EIF1AX and CRM1 expression in HEC-1A cells subjected to ITDR under 0.00001-100 μM 2,5-MeC with thermal challenge at 57 °C (see also Figure 5k). (c) Western blot of EIF1AX and CRM1 in HEC-1A cells digested with varying protease doses in the presence of 2,5-MeC (see also Figure 5l). (d) ITDR analysis in HEC-1A cells overexpressing a site-mutated EIF1AX fragment, treated with 2,5-MeC, and heated at 57 °C. (e) Purified EIF1AX was preincubated with free 2,5-MeC (1-10 mM), followed by incubation with 2 mM biotinylated-2,5-MeC. (f) Immunofluorescence of EIF1AX in HEC-1A cells treated with biotinylated-2,5-MeC (1 μM, 1 day). Scale bar: 10 μm.

(g) Immunofluorescence of EIF1AX in HEC-1A cells treated with EGFR inhibitors alisertib or sapitinib. Scale bar: 25 μm. (h) Long-term colony formation assay of TP53-mutant endometrial cancer cell lines cultured for 10-14 days at low confluence with or without EGFR inhibitors (alisertib or sapitinib).

**
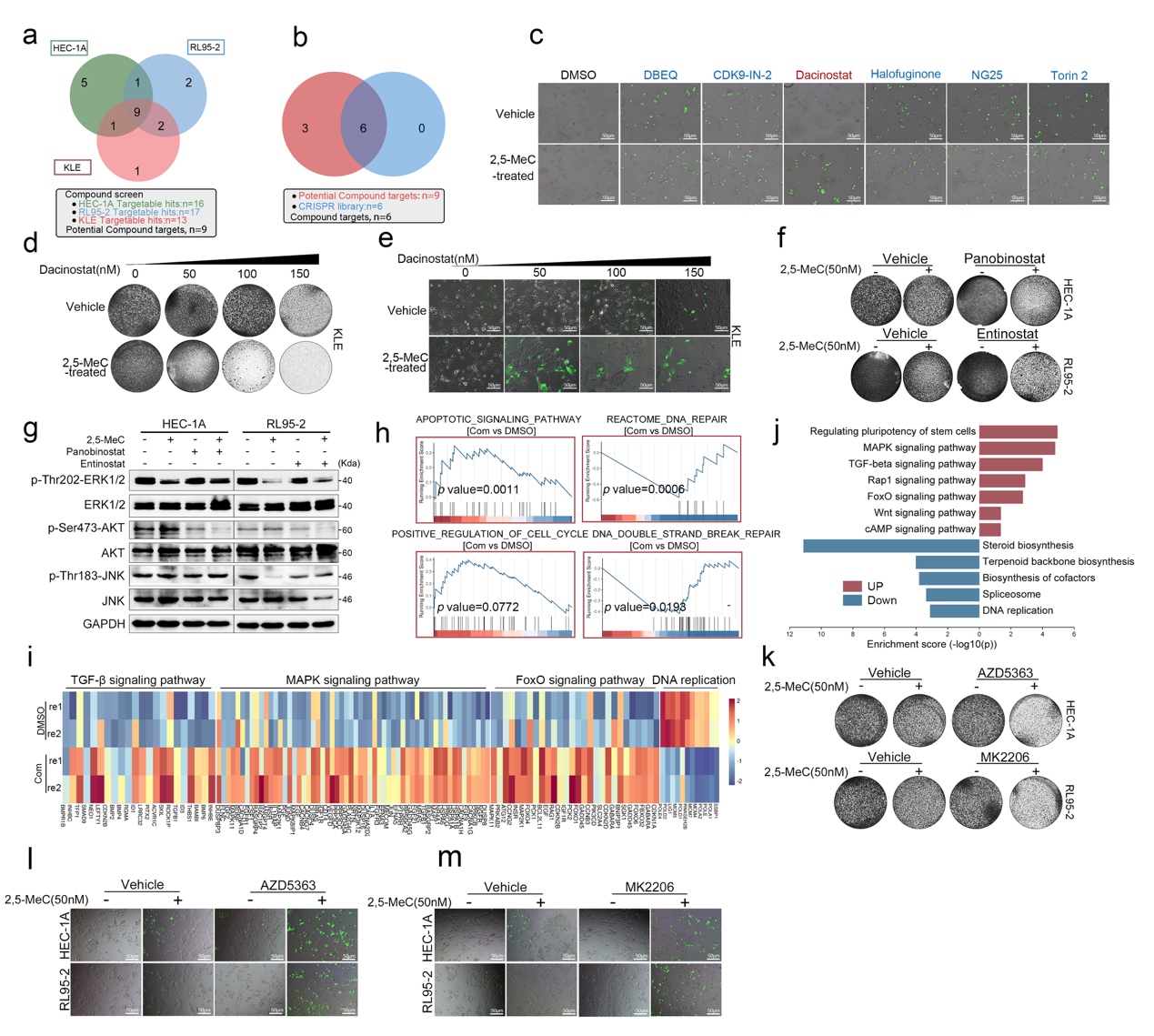
**

**Figure S12. Dacinostat induces apoptosis in 2,5-MeC-induced senescent cells via JNK/MAPK signaling activation.** (a, b) Venn diagrams summarizing compound library screening (a) and CRISPR screening (b) results across three endometrial cancer (EC) cell lines (see also Figure 6c). (c) Viability of 2,5-MeC-treated cells in response to different compounds. Scale bar: 50 μm. (d, e) Colony formation (d) and caspase-3/7 apoptosis assays (e) in KLE cells treated with 1 μM 2,5-MeC followed by 48 h Dacinostat exposure. Scale bar: 50 μm. (f, g) Colony formation (f) and Western blot analyses (g) in HEC-1A cells treated with Panobinostat or Entinostat for 48 h after 2,5-MeC exposure. Scale bar: 50 μm. (h) RNA-seq analysis of HEC-1A cells treated with 2,5-MeC combined with Dacinostat (com). (i) Heatmap of log₂ fold changes in gene expression in HEC-1A cells sequentially treated with 2,5-MeC and Dacinostat. (j) KEGG pathway analysis of gene sets from HEC-1A cells treated with 2,5-MeC and Dacinostat. (k–m) Colony formation (k) and caspase-3/7 apoptosis assays (l, m) in 2,5-MeC-induced senescent EC cells treated with AZD5363 or MK2206. Scale bar: 50 μm.


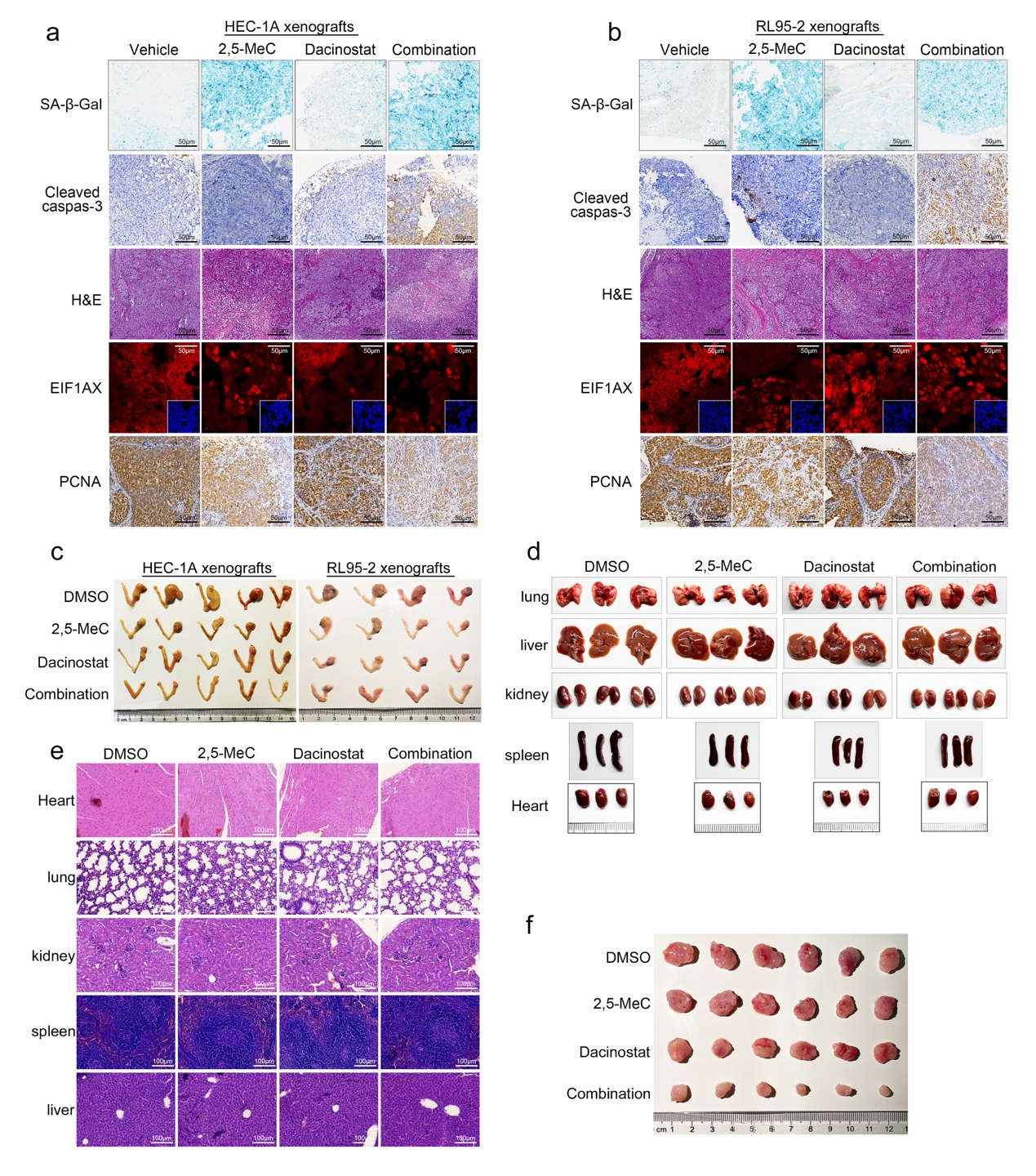


**Figure S13. Combination of pro-senescence treatment and Dacinostat suppresses tumor growth in endometrial cancer xenografts.** (a, b) H&E staining, SA-β-gal activity, and expression of PCNA, cleaved caspase-3, and EIF1AX in HEC-1A and RL95-2 xenografts treated with 2,5-MeC and Dacinostat. Scale bar: 50 μm. (c) Representative images of HEC-1A and RL95-2 xenografts after treatment with 2,5-MeC and Dacinostat. (d, e) Representative images of histopathological images of key organs (heart, liver, lungs, and kidneys) from the treated mice. Scale bar: 100 μm. (f) Representative image of a PDX model after treatment with 2,5-MeC and Dacinostat.


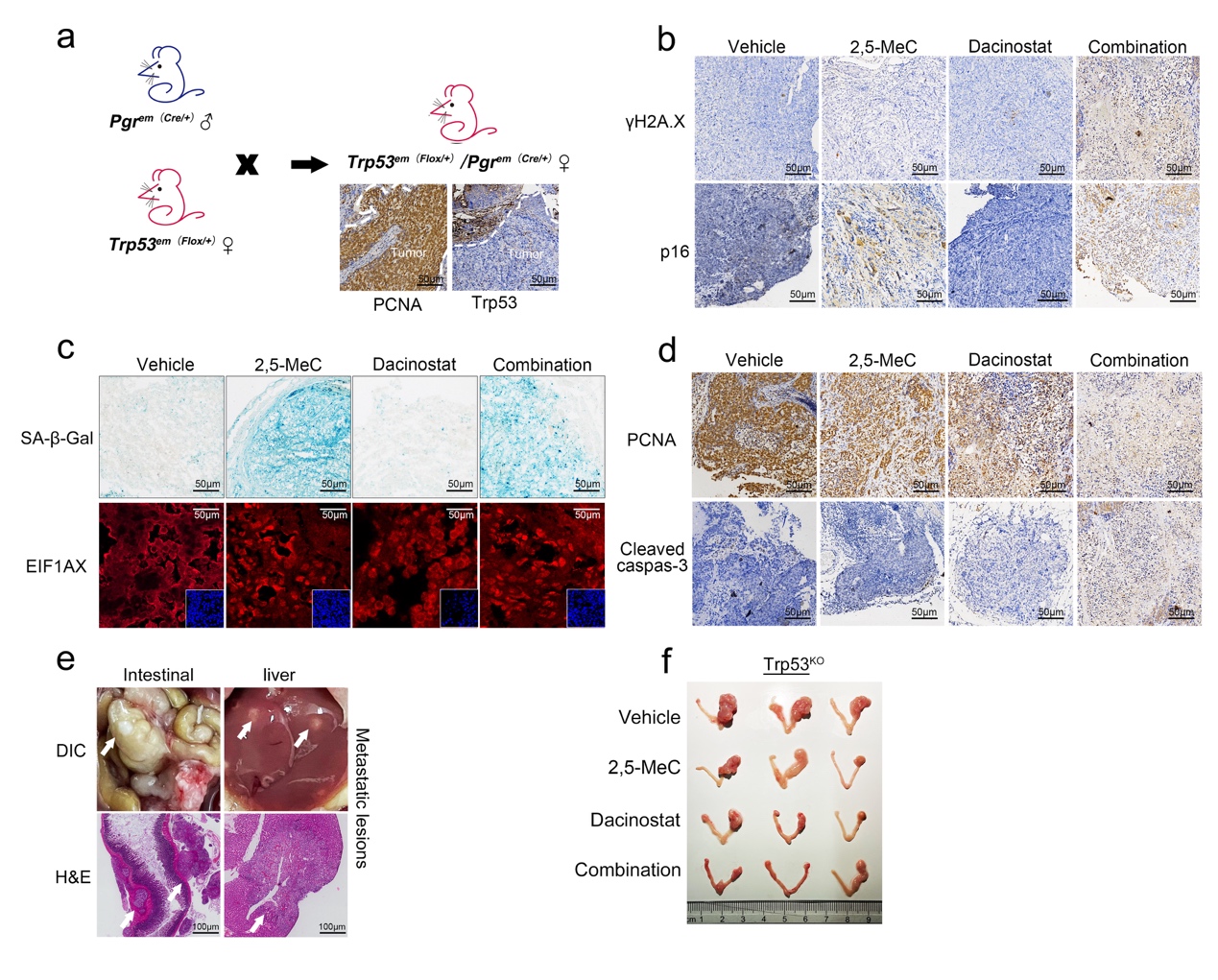


**Figure S14. Combination of pro-senescence treatment and Dacinostat suppresses tumor growth in *TP53*-deficient murine endometrial cancer models.** (a) Schematic of the *Trp53*‑deficient endometrial cancer model. (b–d) SA-β-gal staining and expression of EIF1AX, γH2AX, p16, PCNA, and cleaved caspase-3 following combination treatment with 2,5-MeC and Dacinostat. Scale bar: 50 μm. (e) Representative images of metastatic lesions in vehicle-treated *Trp53*‑deficient mice. Scale bar: 100 μm. (f) Representative images of *Trp53*‑deficient murine models after treatment with 2,5-MeC and Dacinostat.
