# Supplementary material for: EIF1AX Nucleolar Condensates Enhance Susceptibilities for the Management of Endometrial Cancer
Source: Adv Sci (Weinh). 2025 Dec 17;13(12):e04238. doi: 10.1002/advs.202504238 (PMC12948210; doi:10.1002/advs.202504238)
Supplement: Supplementary file 2 — Supplemental Tables [file ADVS-13-e04238-s001.pdf]

EIF1AX nucleolar condensates enhance susceptibilities for the management of endometrial cancer

Chengyu Lv, Zihang Lin, Jiandong Sun, Yuhong Ye, Qibin Wu, Liangzhi Cai, Dabin Liu, Pengming Sun, Shie Wang

Table of contents:

Supplementary Table 1 ······························· 1

Supplementary Table 2 ······························· 5

Supplementary Table 3 ······························· 238

Supplementary Table 4 ······························· 254

Supplementary Table 5 ······························· 267

Supplementary Table 6 ······························· 267

Supplementary Table 1. Interacting proteins of EIF1AX-WT, EIF1AX-NLS and N11A+D142A identified by Co-IP/MS.

1	**Supplementary Table S1. Interacting proteins of EIF1AX-WT, EIF1AX-NLS and N11A+D142A identified by Co-IP/MS.**													
2														
3	**All interacting proteins of EIF1AX-WT identified by Co-IP/MS**													
4	**Accession**	**Description**	**Exp. q-val**	**Sum PEP Score**	**Coverage**	**# Peptides**	**# PSMs**	**# Unique I**	**# Protein**	**# AAs**	**MW [kDa**	**calc. pI**	**Score Seq**	**# Peptides Sequest HT**
5	Q92614	Unconventional myosin-XVIIIa OS=Homo sapiens OX=9606 GN=MYO18A PE=1 SV=3	0	261.311	47	32	99	32	1	2054	232.971	6.3	172.8	32
6	O00159	Unconventional myosin-Ic OS=Homo sapiens OX=9606 GN=MYO1C PE=1 SV=4	0	182.064	66	24	140	24	1	1063	121.606	9.41	251.32	24
7	O94832	Unconventional myosin-Id OS=Homo sapiens OX=9606 GN=MYO1D PE=1 SV=2	0	169.346	64	34	91	9	1	1006	116.129	9.39	116.72	34
8	P11021	Endoplasmic reticulum chaperone BiP OS=Homo sapiens OX=9606 GN=HSPA5 PE=1 SV=2	0	159.819	30	29	46	28	1	654	72.288	5.16	73	29
9	P38646	Stress-70 protein, mitochondrial OS=Homo sapiens OX=9606 GN=HSPA9 PE=1 SV=2	0	158.944	50	34	81	1	1	679	73.635	6.16	113.75	34
10	P11142	Heat shock cognate 71 kDa protein OS=Homo sapiens OX=9606 GN=HSPA8 PE=1 SV=1	0	150.151	48	31	77	1	1	646	70.854	5.52	102.44	31
11	P47813	Eukaryotic translation initiation factor 1A, X-chromosomal OS=Homo sapiens OX=9606 GN=EIF1AX PE=1 !	0	147.152	22	41	50	41	1	144	16.432	5.24	53.7	41
12	Q9UM54	Unconventional myosin-VI OS=Homo sapiens OX=9606 GN=MYO6 PE=1 SV=4	0	142.588	37	34	50	32	1	1294	149.596	8.53	79.75	34
13	Q9ULV0	Unconventional myosin-Vb OS=Homo sapiens OX=9606 GN=MYO5B PE=1 SV=3	0	137.318	52	27	47	24	1	1848	213.538	7.2	65.12	27
14	P14618	Pyruvate kinase PKM OS=Homo sapiens OX=9606 GN=PKM PE=1 SV=4	0	128.22	20	41	47	41	1	531	57.9	7.84	56.09	41
15	P08238	Heat shock protein HSP 90-beta OS=Homo sapiens OX=9606 GN=HSP90AB1 PE=1 SV=4	0	114.665	30	28	45	27	1	724	83.212	5.03	61.21	28
16	P09327	Villin-1 OS=Homo sapiens OX=9606 GN=VIL1 PE=1 SV=4	0	112.214	43	25	37	22	1	827	92.637	6.39	62.16	25
17	P07900	Heat shock protein HSP 90-alpha OS=Homo sapiens OX=9606 GN=HSP90AA1 PE=1 SV=5	0	108.244	40	28	43	23	1	732	84.607	5.02	60.81	28
18	O43795	Unconventional myosin-Ib OS=Homo sapiens OX=9606 GN=MYO1B PE=1 SV=3	0	103.237	51	12	61	12	1	1136	131.902	9.38	157.38	12
19	P0DMV9	Heat shock 70 kDa protein 1B OS=Homo sapiens OX=9606 GN=HSPA1B PE=1 SV=1	0	101.418	27	27	43	27	1	641	70.009	5.66	48.99	27
20	Q9NQX4	Unconventional myosin-Vc OS=Homo sapiens OX=9606 GN=MYO5C PE=1 SV=2	0	100.044	24	25	34	25	1	1742	202.682	7.71	41.54	25
21	P29401	Transketolase OS=Homo sapiens OX=9606 GN=TKT PE=1 SV=3	0	97.428	36	20	33	17	1	623	67.835	7.66	54.78	20
22	P34932	Heat shock 70 kDa protein 4 OS=Homo sapiens OX=9606 GN=HSPA4 PE=1 SV=4	0	96.291	11	35	38	35	1	840	94.271	5.19	33.45	35
23	Q9Y230	RuvB-like 2 OS=Homo sapiens OX=9606 GN=RUVBL2 PE=1 SV=3	0	96.067	44	18	53	14	1	463	51.125	5.64	63.6	18
24	Q9Y2W1	Thyroid hormone receptor-associated protein 3 OS=Homo sapiens OX=9606 GN=THRAP3 PE=1 SV=2	0	89.767	20	14	22	14	1	955	108.601	10.15	36.97	14
25	Q9Y265	RuvB-like 1 OS=Homo sapiens OX=9606 GN=RUVBL1 PE=1 SV=1	0	80.661	20	21	22	21	1	456	50.196	6.42	28.85	21
26	P23396	40S ribosomal protein S3 OS=Homo sapiens OX=9606 GN=RPS3 PE=1 SV=2	0	79.107	41	17	23	14	1	243	26.671	9.66	37.39	17
27	Q9BYX7	Putative beta-actin-like protein 3 OS=Homo sapiens OX=9606 GN=POTEKP PE=5 SV=1	0	76.847	39	20	62	0	1	375	41.989	6.33	73.81	20
28	P22314	Ubiquitin-like modifier-activating enzyme 1 OS=Homo sapiens OX=9606 GN=UBA1 PE=1 SV=3	0	67.23	31	21	27	21	1	1058	117.774	5.76	43.19	21
29	O95425	Supervillin OS=Homo sapiens OX=9606 GN=SVIL PE=1 SV=2	0	64.965	30	16	27	12	1	2214	247.593	6.98	48.12	16
30	P10809	60 kDa heat shock protein, mitochondrial OS=Homo sapiens OX=9606 GN=HSPD1 PE=1 SV=2	0	64.55	26	17	22	10	1	573	61.016	5.87	33.14	17
31	Q9Y4I1	Unconventional myosin-Va OS=Homo sapiens OX=9606 GN=MYO5A PE=1 SV=2	0	63.128	15	24	27	24	1	1855	215.269	8.48	35.18	24
32	P18124	60S ribosomal protein L7 OS=Homo sapiens OX=9606 GN=RPL7 PE=1 SV=1	0	61.185	36	15	41	2	1	248	29.207	10.65	41.99	15
33	P62701	40S ribosomal protein S4, X isoform OS=Homo sapiens OX=9606 GN=RPS4X PE=1 SV=2	0	60.339	28	16	22	16	1	263	29.579	10.15	31.77	16
34	P36578	60S ribosomal protein L4 OS=Homo sapiens OX=9606 GN=RPL4 PE=1 SV=5	0	59.745	19	5	160	4	1	427	47.667	11.06	282.14	5
35	P61978	Heterogeneous nuclear ribonucleoprotein K OS=Homo sapiens OX=9606 GN=HNRNPK PE=1 SV=1	0	59.318	31	18	22	14	1	463	50.944	5.54	26.87	18
36	P62263	40S ribosomal protein S14 OS=Homo sapiens OX=9606 GN=RPS14 PE=1 SV=3	0	58.822	16	18	23	18	1	151	16.263	10.05	34.81	18
37	P63104	14-3-3 protein zeta/delta OS=Homo sapiens OX=9606 GN=YWHAZ PE=1 SV=1	0	58.562	11	23	27	23	1	245	27.728	4.79	18.95	23
38	P62906	60S ribosomal protein L10a OS=Homo sapiens OX=9606 GN=RPL10A PE=1 SV=2	0	57.962	24	17	23	17	1	217	24.816	9.94	24.33	17
39	P05388	60S acidic ribosomal protein P0 OS=Homo sapiens OX=9606 GN=RPLP0 PE=1 SV=1	0	57.786	14	17	19	17	1	317	34.252	5.97	21.18	17
40	P62424	60S ribosomal protein L7a OS=Homo sapiens OX=9606 GN=RPL7A PE=1 SV=2	0	54.973	32	15	24	15	1	266	29.977	10.61	17.36	15
41	P62241	40S ribosomal protein S8 OS=Homo sapiens OX=9606 GN=RPS8 PE=1 SV=2	0	53.768	31	15	18	12	1	208	24.19	10.32	27.88	15
42	Q02878	60S ribosomal protein L6 OS=Homo sapiens OX=9606 GN=RPL6 PE=1 SV=3	0	52.665	31	12	16	12	1	288	32.708	10.58	36.57	12
43	P62753	40S ribosomal protein S6 OS=Homo sapiens OX=9606 GN=RPS6 PE=1 SV=1	0	52.389	61	12	17	12	1	249	28.663	10.84	30.49	12
44	P60842	Eukaryotic initiation factor 4A-I OS=Homo sapiens OX=9606 GN=EIF4A1 PE=1 SV=1	0	50.804	22	15	20	11	1	406	46.125	5.48	14.29	15
45	O00571	ATP-dependent RNA helicase DDX3X OS=Homo sapiens OX=9606 GN=DDX3X PE=1 SV=3	0	50.802	34	13	37	3	1	662	73.198	7.18	32.15	13
46	P31946	14-3-3 protein beta/alpha OS=Homo sapiens OX=9606 GN=YWHAB PE=1 SV=3	0	50.331	6	21	23	21	1	246	28.065	4.83	10.11	21
47	P62249	40S ribosomal protein S16 OS=Homo sapiens OX=9606 GN=RPS16 PE=1 SV=2	0	50.247	34	11	17	11	1	146	16.435	10.21	27.15	11
48	P55786	Puromycin-sensitive aminopeptidase OS=Homo sapiens OX=9606 GN=NPEPPS PE=1 SV=2	0	49.379	19	15	20	15	1	919	103.211	5.72	21.08	15
49	P13010	X-ray repair cross-complementing protein 5 OS=Homo sapiens OX=9606 GN=XRCC5 PE=1 SV=3	0	48.657	36	7	12	7	1	732	82.652	5.81	15.62	7
50	P15880	40S ribosomal protein S2 OS=Homo sapiens OX=9606 GN=RPS2 PE=1 SV=2	0	47.838	21	17	19	16	1	293	31.305	10.24	18.22	17
51	P09651	Heterogeneous nuclear ribonucleoprotein A1 OS=Homo sapiens OX=9606 GN=HNRNPA1 PE=1 SV=5	0	47.787	28	12	33	1	1	372	38.723	9.13	34.85	12
52	P16615	Sarcoplasmic/endoplasmic reticulum calcium ATPase 2 OS=Homo sapiens OX=9606 GN=ATP2A2 PE=1 SV=	0	46.926	11	20	22	20	1	1042	114.683	5.34	17.12	20
53	P62136	Serine/threonine-protein phosphatase PP1-alpha catalytic subunit OS=Homo sapiens OX=9606 GN=PPP1CA I	0	46.92	25	14	23	12	1	330	37.488	6.33	27.36	14
54	P52597	Heterogeneous nuclear ribonucleoprotein F OS=Homo sapiens OX=9606 GN=HNRNPF PE=1 SV=3	0	46.222	14	15	16	14	1	415	45.643	5.58	11.15	15
55	O14818	Proteasome subunit alpha type-7 OS=Homo sapiens OX=9606 GN=PSMA7 PE=1 SV=1	0	45.72	33	13	22	13	1	248	27.87	8.46	32.73	13
56	P46781	40S ribosomal protein S9 OS=Homo sapiens OX=9606 GN=RPS9 PE=1 SV=3	0	45.434	28	13	15	12	1	194	22.578	10.65	15.93	13
57	P61247	40S ribosomal protein S3a OS=Homo sapiens OX=9606 GN=RPS3A PE=1 SV=2	0	44.308	27	9	12	9	1	264	29.926	9.73	26.21	9
58	P62258	14-3-3 protein epsilon OS=Homo sapiens OX=9606 GN=YWHAE PE=1 SV=1	0	44.3	15	15	18	15	1	255	29.155	4.74	18.48	15
59	P30050	60S ribosomal protein L12 OS=Homo sapiens OX=9606 GN=RPL12 PE=1 SV=1	0	43.149	18	15	20	15	1	165	17.808	9.42	23.28	15
60	P51991	Heterogeneous nuclear ribonucleoprotein A3 OS=Homo sapiens OX=9606 GN=HNRNPA3 PE=1 SV=2	0	43.051	55	12	19	11	1	378	39.571	9.01	28.88	12
61	P21589	5'-nucleotidase OS=Homo sapiens OX=9606 GN=NT5E PE=1 SV=1	0	42.7	22	13	18	6	1	574	63.327	7.03	20.52	13
62	P26373	60S ribosomal protein L13 OS=Homo sapiens OX=9606 GN=RPL13 PE=1 SV=4	0	42.125	27	9	13	8	1	211	24.247	11.65	18.56	9
63	P62847	40S ribosomal protein S24 OS=Homo sapiens OX=9606 GN=RPS24 PE=1 SV=1	0	41.626	15	12	15	12	1	133	15.413	10.78	17.47	12
64	P21796	Voltage-dependent anion-selective channel protein 1 OS=Homo sapiens OX=9606 GN=VDAC1 PE=1 SV=2	0	41.079	39	12	20	12	1	283	30.754	8.54	21.66	12
65	P18621	60S ribosomal protein L17 OS=Homo sapiens OX=9606 GN=RPL17 PE=1 SV=3	0	40.469	18	11	38	0	1	184	21.383	10.17	32.74	11
66	P62979	Ubiquitin-40S ribosomal protein S27a OS=Homo sapiens OX=9606 GN=RPS27A PE=1 SV=2	0	40.021	21	8	21	8	1	156	17.953	9.64	42.96	8
67	P05387	60S acidic ribosomal protein P2 OS=Homo sapiens OX=9606 GN=RPLP2 PE=1 SV=1	0	39.855	22	9	12	9	1	115	11.658	4.54	19.22	9
68	Q04917	14-3-3 protein eta OS=Homo sapiens OX=9606 GN=YWHAH PE=1 SV=4	0	39.414	16	10	17	10	1	246	28.201	4.84	32.04	10
69	P61981	14-3-3 protein gamma OS=Homo sapiens OX=9606 GN=YWHAG PE=1 SV=2	0	39.091	16	13	17	13	1	247	28.285	4.89	14.29	13
70	P62750	60S ribosomal protein L23a OS=Homo sapiens OX=9606 GN=RPL23A PE=1 SV=1	0	38.823	4	16	17	16	1	156	17.684	10.45	13.1	16
71	P46778	60S ribosomal protein L21 OS=Homo sapiens OX=9606 GN=RPL21 PE=1 SV=2	0	38.684	9	15	15	15	1	160	18.553	10.49	12.51	15
72	Q07020	60S ribosomal protein L18 OS=Homo sapiens OX=9606 GN=RPL18 PE=1 SV=2	0	38.641	37	14	23	3	1	188	21.621	11.72	17.9	14
73	O75083	WD repeat-containing protein 1 OS=Homo sapiens OX=9606 GN=WDR1 PE=1 SV=4	0	38.259	7	16	16	16	1	606	66.152	6.65	11.7	16
74	P60866	40S ribosomal protein S20 OS=Homo sapiens OX=9606 GN=RPS20 PE=1 SV=1	0	37.954	23	11	15	7	1	119	13.364	9.94	19.39	11
75	P62277	40S ribosomal protein S13 OS=Homo sapiens OX=9606 GN=RPS13 PE=1 SV=2	0	37	32	7	10	7	1	151	17.212	10.54	19.78	7
76	P32969	60S ribosomal protein L9 OS=Homo sapiens OX=9606 GN=RPL9 PE=1 SV=1	0	36.588	26	14	19	14	1	192	21.85	9.95	14.74	14
77	O60292	Signal-induced proliferation-associated 1-like protein 3 OS=Homo sapiens OX=9606 GN=SIPA1L3 PE=1 SV	0	36.386	34	11	13	11	1	1781	194.49	8.32	16.77	11
78	P18206	Vinculin OS=Homo sapiens OX=9606 GN=VCL PE=1 SV=4	0	35.966	18	13	15	11	1	1134	123.722	5.66	3.85	13
79	P62888	60S ribosomal protein L30 OS=Homo sapiens OX=9606 GN=RPL30 PE=1 SV=2	0	35.378	21	5	7	2	1	115	12.776	9.63	21.13	5
80	P61353	60S ribosomal protein L27 OS=Homo sapiens OX=9606 GN=RPL27 PE=1 SV=2	0	34.707	45	9	19	6	1	136	15.788	10.56	20.67	9
81	P40429	60S ribosomal protein L13a OS=Homo sapiens OX=9606 GN=RPL13A PE=1 SV=2	0	34.252	8	13	14	13	1	203	23.562	10.93	12.01	13
82	P16989	Y-box-binding protein 3 OS=Homo sapiens OX=9606 GN=YBX3 PE=1 SV=4	0	34.189	19	13	17	13	1	372	40.066	9.77	15.72	13
83	Q13200	26S proteasome non-ATPase regulatory subunit 2 OS=Homo sapiens OX=9606 GN=PSMD2 PE=1 SV=3	0	34.088	28	10	16	1	1	908	100.136	5.2	20.13	10
84	P55072	Transitional endoplasmic reticulum ATPase OS=Homo sapiens OX=9606 GN=VCP PE=1 SV=4	0	33.384	18	10	11	10	1	806	89.266	5.26	9.39	10
85	P62244	40S ribosomal protein S15a OS=Homo sapiens OX=9606 GN=RPS15A PE=1 SV=2	0	33.376	17	6	7	6	1	130	14.83	10.13	10.88	6
86	P36873	Serine/threonine-protein phosphatase PP1-gamma catalytic subunit OS=Homo sapiens OX=9606 GN=PPP1CC	0	32.716	37	12	20	1	1	323	36.96	6.54	14.06	12
87	P50454	Serpin H1 OS=Homo sapiens OX=9606 GN=SERPINH1 PE=1 SV=2	0	32.683	9	12	12	12	1	418	46.411	8.69	10.34	12
88	Q14254	Flotillin-2 OS=Homo sapiens OX=9606 GN=FLOT2 PE=1 SV=2	0	32.034	13	8	18	4	1	428	47.035	5.25	15.2	8
89	P62140	Serine/threonine-protein phosphatase PP1-beta catalytic subunit OS=Homo sapiens OX=9606 GN=PPP1CB PE	0	31.796	23	13	16	13	1	327	37.163	6.19	16.26	13
90	P08865	40S ribosomal protein SA OS=Homo sapiens OX=9606 GN=RPSA PE=1 SV=4	0	31.669	24	9	11	9	1	295	32.833	4.87	13.19	9
91	P39023	60S ribosomal protein L3 OS=Homo sapiens OX=9606 GN=RPL3 PE=1 SV=2	0	31.528	12	9	12	9	1	403	46.08	10.18	12.34	9
92	P27348	14-3-3 protein theta OS=Homo sapiens OX=9606 GN=YWHAQ PE=1 SV=1	0	31.463	6	11	12	11	1	245	27.747	4.78	11.87	11
93	P02786	Transferrin receptor protein 1 OS=Homo sapiens OX=9606 GN=TFRC PE=1 SV=2	0	31.103	30	10	12	10	1	760	84.818	6.61	11.75	10
94	Q9UHD8	Septin-9 OS=Homo sapiens OX=9606 GN=SEPTIN9 PE=1 SV=2	0	30.796	49	11	46	1	1	586	65.361	8.97	53.02	11
95	P27635	60S ribosomal protein L10 OS=Homo sapiens OX=9606 GN=RPL10 PE=1 SV=4	0	30.605	16	9	33	1	1	214	24.588	10.08	27.77	9
96	P42766	60S ribosomal protein L35 OS=Homo sapiens OX=9606 GN=RPL35 PE=1 SV=2	0	30.334	14	11	11	11	1	123	14.543	11.05	16.61	11
97	Q6UWP8	Suprabasin OS=Homo sapiens OX=9606 GN=SBSN PE=1 SV=2	0	29.923	43	5	15	2	1	590	60.505	7.01	22.53	5
98	P61254	60S ribosomal protein L26 OS=Homo sapiens OX=9606 GN=RPL26 PE=1 SV=1	0	29.76	19	11	16	10	1	145	17.248	10.55	15.37	11
99	Q16881	Thioredoxin reductase 1, cytoplasmic OS=Homo sapiens OX=9606 GN=TXNRD1 PE=1 SV=3	0	29.653	18	6	9	6	1	649	70.862	7.39	14.81	6
100	P62269	40S ribosomal protein S18 OS=Homo sapiens OX=9606 GN=RPS18 PE=1 SV=3	0	29.525	56	7	12	5	1	152	17.708	10.99	9.3	7
101	P42677	40S ribosomal protein S27 OS=Homo sapiens OX=9606 GN=RPS27 PE=1 SV=3	0	29.472	15	8	9	8	1	84	9.455	9.45	10.8	8
102	Q96P63	Serpin B12 OS=Homo sapiens OX=9606 GN=SERPINB12 PE=1 SV=1	0	29.156	55	9	13	9	1	405	46.247	5.53	13.53	9
103	P84103	Serine/arginine-rich splicing factor 3 OS=Homo sapiens OX=9606 GN=SRSF3 PE=1 SV=1	0	28.932	40	10	16	10	1	164	19.318	11.65	15.36	10
104	Q9Y383	Putative RNA-binding protein Luc7-like 2 OS=Homo sapiens OX=9606 GN=LUC7L2 PE=1 SV=2	0	28.931	13	9	10	9	1	392	46.486	10.01	13.36	9
105	P62266	40S ribosomal protein S23 OS=Homo sapiens OX=9606 GN=RPS23 PE=1 SV=3	0	28.575	36	10	13	10	1	143	15.798	10.49	10.95	10
106	P83731	60S ribosomal protein L24 OS=Homo sapiens OX=9606 GN=RPL24 PE=1 SV=1	0	28.436	10	11	15	11	1	157	17.768	11.25	11.36	11
107	P30153	Serine/threonine-protein phosphatase 2A 65 kDa regulatory subunit A alpha isoform OS=Homo sapiens OX=!	0	28.321	19	7	23	1	1	589	65.267	5.11	20.82	7
108	P62899	60S ribosomal protein L31 OS=Homo sapiens OX=9606 GN=RPL31 PE=1 SV=1	0	28.086	36	6	17	6	1	125	14.454	10.54	32.91	6
109	P62280	40S ribosomal protein S11 OS=Homo sapiens OX=9606 GN=RPS11 PE=1 SV=3	0	27.919	9	8	14	8	1	158	18.419	10.3	20.24	8
110	P63173	60S ribosomal protein L38 OS=Homo sapiens OX=9606 GN=RPL38 PE=1 SV=2	0	27.773	15	9	9	8	1	70	8.213	10.1	9.27	9
111	Q8IWV7	E3 ubiquitin-protein ligase UBR1 OS=Homo sapiens OX=9606 GN=UBR1 PE=1 SV=1	0	27.599	17	10	12	8	1	1749	200.08	6.01	12.64	10
112	P62195	26S proteasome regulatory subunit 8 OS=Homo sapiens OX=9606 GN=PSMC5 PE=1 SV=1	0	27.352	23	8	11	8	1	406	45.597	7.55	15.23	8
113	Q9UQ35	Serine/arginine repetitive matrix protein 2 OS=Homo sapiens OX=9606 GN=SRRM2 PE=1 SV=2	0	26.551	17	8	10	8	1	2752	299.438	12.06	10.18	8
114	P46776	60S ribosomal protein L27a OS=Homo sapiens OX=9606 GN=RPL27A PE=1 SV=2	0	26.349	10	8	9	8	1	148	16.551	11	12.31	8
115	P62081	40S ribosomal protein S7 OS=Homo sapiens OX=9606 GN=RPS7 PE=1 SV=1	0	26.295	15	8	10	8	1	194	22.113	10.1	11.92	8
116	Q15717	ELAV-like protein 1 OS=Homo sapiens OX=9606 GN=ELAVL1 PE=1 SV=2	0	26.228	22	8	12	7	1	326	36.069	9.17	17.3	8
117	P31948	Stress-induced-phosphoprotein 1 OS=Homo sapiens OX=9606 GN=STIP1 PE=1 SV=1	0	26.038	11	9	10	8	1	543	62.599	6.8	10.89	9
118	O15231	Zinc finger protein 185 OS=Homo sapiens OX=9606 GN=ZNF185 PE=1 SV=3	0	25.902	13	6	7	6	1	689	73.48	7.01	7.55	6
119	Q16629	Serine/arginine-rich splicing factor 7 OS=Homo sapiens OX=9606 GN=SRSF7 PE=1 SV=1	0	25.742	24	4	4	4	1	238	27.35	11.82	11.47	4
120	P42765	3-ketoacyl-CoA thiolase, mitochondrial OS=Homo sapiens OX=9606 GN=ACAA2 PE=1 SV=2	0	25.625	26	8	10	8	1	397	41.898	8.09	8.16	8
121	P53007	Tricarboxylate transport protein, mitochondrial OS=Homo sapiens OX=9606 GN=SLC25A1 PE=1 SV=2	0	25.497	34	6	9	6	1	311	33.991	9.89	13.85	6
122	P62829	60S ribosomal protein L23 OS=Homo sapiens OX=9606 GN=RPL23 PE=1 SV=1	0	25.461	35	8	11	8	1	140	14.856	10.51	9.68	8
123	P49755	Transmembrane emp24 domain-containing protein 10 OS=Homo sapiens OX=9606 GN=TMED10 PE=1 SV=	0	25.336	47	10	11	10	1	219	24.96	7.44	4.58	10
124	Q86V81	THO complex subunit 4 OS=Homo sapiens OX=9606 GN=ALYREF PE=1 SV=3	0	25.308	53	5	11	5	1	257	26.872	11.15	12.88	5
125	O43242	26S proteasome non-ATPase regulatory subunit 3 OS=Homo sapiens OX=9606 GN=PSMD3 PE=1 SV=2	0	24.917	39	7	9	7	1	534	60.939	8.44	12.05	7
126	Q6P5R6	60S ribosomal protein L22-like 1 OS=Homo sapiens OX=9606 GN=RPL22L1 PE=1 SV=2	0	24.617	8	6	8	6	1	122	14.598	9.38	4.25	6
127	Q12931	Heat shock protein 75 kDa, mitochondrial OS=Homo sapiens OX=9606 GN=TRAP1 PE=1 SV=3	0	24.407	56	7	12	7	1	704	80.06	8.21	16.01	7
128	P11177	Pyruvate dehydrogenase E1 component subunit beta, mitochondrial OS=Homo sapiens OX=9606 GN=PDHB	0	24.295	14	7	10	7	1	359	39.208	6.65	9.92	7
129	P55036	26S proteasome non-ATPase regulatory subunit 4 OS=Homo sapiens OX=9606 GN=PSMD4 PE=1 SV=1	0	23.708	11	6	10	3	1	377	40.711	4.79	14.78	6
130	P46783	40S ribosomal protein S10 OS=Homo sapiens OX=9606 GN=RPS10 PE=1 SV=1	0	23.679	15	6	11	2	1	165	18.886	10.15	15.4	6
131	Q8IWV8	E3 ubiquitin-protein ligase UBR2 OS=Homo sapiens OX=9606 GN=UBR2 PE=1 SV=1	0	23.655	10	9	9	9	1	1755	200.408	6.24	6.55	9
132	P62841	40S ribosomal protein S15 OS=Homo sapiens OX=9606 GN=RPS15 PE=1 SV=2	0	23.347	15	11	13	8	1	145	17.029	10.39	6.13	11
133	P84098	60S ribosomal protein L19 OS=Homo sapiens OX=9606 GN=RPL19 PE=1 SV=1	0	23.331	28	5	9	5	1	196	23.451	11.47	15.47	5

134	P50914	60S ribosomal protein L14 OS=Homo sapiens OX=9606 GN=RPL14 PE=1 SV=4	0	23.244	49	8	12	8	1	215	23.417	10.93	11.24	8
135	P61513	60S ribosomal protein L37a OS=Homo sapiens OX=9606 GN=RPL37A PE=1 SV=2	0	23.012	14	8	9	8	1	92	10.268	10.43	8.63	8
136	P62851	40S ribosomal protein S25 OS=Homo sapiens OX=9606 GN=RPS25 PE=1 SV=1	0	22.652	22	7	10	7	1	125	13.734	10.11	9.17	7
137	Q9Y5M8	Signal recognition particle receptor subunit beta OS=Homo sapiens OX=9606 GN=SRPRB PE=1 SV=3	0	22.434	39	5	5	5	1	271	29.684	9.04	6.44	5
138	Q9H9B4	Sideroflexin-1 OS=Homo sapiens OX=9606 GN=SFXN1 PE=1 SV=4	0	22.329	9	6	7	6	1	322	35.596	9.07	10.23	6
139	Q13151	Heterogeneous nuclear ribonucleoprotein A0 OS=Homo sapiens OX=9606 GN=HNRNPA0 PE=1 SV=1	0	22.071	19	6	7	6	1	305	30.822	9.29	10.05	6
140	Q6IAA8	Ragulator complex protein LAMTOR1 OS=Homo sapiens OX=9606 GN=LAMTOR1 PE=1 SV=2	0	21.63	13	5	5	5	1	161	17.734	5.15	6.71	5
141	Q07955	Serine/arginine-rich splicing factor 1 OS=Homo sapiens OX=9606 GN=SRSF1 PE=1 SV=2	0	21.494	31	8	10	8	1	248	27.728	10.36	8.63	8
142	Q99729	Heterogeneous nuclear ribonucleoprotein A/B OS=Homo sapiens OX=9606 GN=HNRNPAB PE=1 SV=2	0	21.301	17	5	6	5	1	332	36.202	8.21	4.67	5
143	P62854	40S ribosomal protein S26 OS=Homo sapiens OX=9606 GN=RPS26 PE=1 SV=3	0	21.278	21	4	8	4	1	115	13.007	11	15.15	4
144	P14866	Heterogeneous nuclear ribonucleoprotein L OS=Homo sapiens OX=9606 GN=HNRNPL PE=1 SV=2	0	21.07	59	4	7	4	1	589	64.092	8.22	6.2	4
145	P61313	60S ribosomal protein L15 OS=Homo sapiens OX=9606 GN=RPL15 PE=1 SV=2	0	20.949	15	11	11	11	1	204	24.131	11.62	3.58	11
146	P46777	60S ribosomal protein L5 OS=Homo sapiens OX=9606 GN=RPL5 PE=1 SV=3	0	20.931	7	8	8	8	1	297	34.341	9.72	7.45	8
147	Q9Y277	Voltage-dependent anion-selective channel protein 3 OS=Homo sapiens OX=9606 GN=VDAC3 PE=1 SV=1	0	20.726	15	5	5	5	1	283	30.639	8.66	11.29	5
148	P17980	26S proteasome regulatory subunit 6A OS=Homo sapiens OX=9606 GN=PSMC3 PE=1 SV=3	0	20.652	15	9	10	7	1	439	49.172	5.24	7.54	9
149	P05386	60S acidic ribosomal protein P1 OS=Homo sapiens OX=9606 GN=RPLP1 PE=1 SV=1	0	20.526	4	7	10	7	1	114	11.507	4.32	6	7
150	P46779	60S ribosomal protein L28 OS=Homo sapiens OX=9606 GN=RPL28 PE=1 SV=3	0	20.523	36	8	9	8	1	137	15.738	12.02	5.91	8
151	Q13501	Sequestosome-1 OS=Homo sapiens OX=9606 GN=SQSTM1 PE=1 SV=1	0	20.379	8	8	8	8	1	440	47.657	5.22	7.09	8
152	Q8TDD1	ATP-dependent RNA helicase DDX54 OS=Homo sapiens OX=9606 GN=DDX54 PE=1 SV=2	0	20.313	9	5	5	5	1	881	98.534	10.02	7.19	5
153	P30048	Thioredoxin-dependent peroxide reductase, mitochondrial OS=Homo sapiens OX=9606 GN=PRDX3 PE=1 S	0	19.666	39	7	7	7	1	256	27.675	7.78	10.49	7
154	Q99460	26S proteasome non-ATPase regulatory subunit 1 OS=Homo sapiens OX=9606 GN=PSMD1 PE=1 SV=2	0	19.597	32	3	6	3	1	953	105.769	5.39	11.12	3
155	P22102	Trifunctional purine biosynthetic protein adenosine-3 OS=Homo sapiens OX=9606 GN=GART PE=1 SV=1	0	19.548	10	6	7	3	1	1010	107.699	6.7	5.48	6
156	P62333	26S proteasome regulatory subunit 10B OS=Homo sapiens OX=9606 GN=PSMC6 PE=1 SV=1	0	19.509	12	5	5	5	1	389	44.145	7.49	4.88	5
157	P31942	Heterogeneous nuclear ribonucleoprotein H3 OS=Homo sapiens OX=9606 GN=HNRNPH3 PE=1 SV=2	0	19.104	20	7	9	7	1	346	36.903	6.87	5.62	7
158	P51571	Translocon-associated protein subunit delta OS=Homo sapiens OX=9606 GN=SSR4 PE=1 SV=1	0	18.955	36	5	5	5	1	173	18.987	6.15	8.55	5
159	P62917	60S ribosomal protein L8 OS=Homo sapiens OX=9606 GN=RPL8 PE=1 SV=2	0	18.836	22	5	9	5	1	257	28.007	11.03	17.91	5
160	Q04837	Single-stranded DNA-binding protein, mitochondrial OS=Homo sapiens OX=9606 GN=SSBP1 PE=1 SV=1	0	18.549	16	6	7	6	1	148	17.249	9.6	10.78	6
161	P25786	Proteasome subunit alpha type-1 OS=Homo sapiens OX=9606 GN=PSMA1 PE=1 SV=1	0	18.196	20	5	6	5	1	263	29.537	6.61	4.27	5
162	Q5SSJ5	Heterochromatin protein 1-binding protein 3 OS=Homo sapiens OX=9606 GN=HP1BP3 PE=1 SV=1	0	18.178	23	4	6	4	1	553	61.169	9.67	11.95	4
163	Q9Y3U8	60S ribosomal protein L36 OS=Homo sapiens OX=9606 GN=RPL36 PE=1 SV=3	0	18.145	9	8	9	7	1	105	12.246	11.59	7.35	8
164	Q96HS1	Serine/threonine-protein phosphatase PGAM5, mitochondrial OS=Homo sapiens OX=9606 GN=PGAM5 PE=	0	18.121	29	6	6	6	1	289	31.985	8.68	5.24	6
165	Q16181	Septin-7 OS=Homo sapiens OX=9606 GN=SEPTIN7 PE=1 SV=2	0	17.985	36	3	5	3	1	437	50.648	8.63	8.11	3
166	P62714	Serine/threonine-protein phosphatase 2A catalytic subunit beta isoform OS=Homo sapiens OX=9606 GN=PPP	0	17.966	51	5	9	5	1	309	35.552	5.43	9.42	5
167	Q9NTJ3	Structural maintenance of chromosomes protein 4 OS=Homo sapiens OX=9606 GN=SMC4 PE=1 SV=2	0	17.894	13	5	7	5	1	1288	147.091	6.79	7.98	5
168	P62891	60S ribosomal protein L39 OS=Homo sapiens OX=9606 GN=RPL39 PE=1 SV=2	0	17.835	13	6	6	6	1	51	6.403	12.56	9.04	6
169	Q7L2E3	ATP-dependent RNA helicase DHX30 OS=Homo sapiens OX=9606 GN=DHX30 PE=1 SV=1	0	17.788	44	5	5	5	1	1194	133.855	8.78	6.41	5
170	P45880	Voltage-dependent anion-selective channel protein 2 OS=Homo sapiens OX=9606 GN=VDAC2 PE=1 SV=2	0	17.758	23	3	4	3	1	294	31.547	7.56	8.31	3
171	P37802	Transgelin-2 OS=Homo sapiens OX=9606 GN=TAGLN2 PE=1 SV=3	0	17.707	18	8	9	7	1	199	22.377	8.25	2.82	8
172	Q9NVP1	ATP-dependent RNA helicase DDX18 OS=Homo sapiens OX=9606 GN=DDX18 PE=1 SV=2	0	17.184	12	4	5	3	1	670	75.359	9.5	7.21	4
173	Q9P035	Very-long-chain (3R)-3-hydroxyacyl-CoA dehydratase 3 OS=Homo sapiens OX=9606 GN=HACD3 PE=1 SV	0	17.022	15	4	4	4	1	362	43.132	8.94	9.33	4
174	P43686	26S proteasome regulatory subunit 6B OS=Homo sapiens OX=9606 GN=PSMC4 PE=1 SV=2	0	16.989	25	5	7	5	1	418	47.337	5.21	11.89	5
175	O75494	Serine/arginine-rich splicing factor 10 OS=Homo sapiens OX=9606 GN=SRSF10 PE=1 SV=1	0	16.977	13	4	25	3	1	262	31.282	11.27	32.1	4
176	Q9P0L0	Vesicle-associated membrane protein-associated protein A OS=Homo sapiens OX=9606 GN=VAPA PE=1 SV=	0	16.886	14	7	7	7	1	249	27.875	8.62	7.41	7
177	P62913	60S ribosomal protein L11 OS=Homo sapiens OX=9606 GN=RPL11 PE=1 SV=2	0	16.818	42	6	6	6	1	178	20.24	9.6	4.7	6
178	P60900	Proteasome subunit alpha type-6 OS=Homo sapiens OX=9606 GN=PSMA6 PE=1 SV=1	0	16.771	3	5	5	1	1	246	27.382	6.76	6.13	5
179	Q14141	Septin-6 OS=Homo sapiens OX=9606 GN=SEPTIN6 PE=1 SV=4	0	16.735	29	5	9	5	1	434	49.685	6.67	8.72	5
180	P15313	V-type proton ATPase subunit B, kidney isoform OS=Homo sapiens OX=9606 GN=ATP6V1B1 PE=1 SV=3	0	16.683	15	5	7	5	1	513	56.797	5.66	8.52	5
181	Q96I99	Succinate--CoA ligase [GDP-forming] subunit beta, mitochondrial OS=Homo sapiens OX=9606 GN=SUCLG	0	16.639	5	8	8	8	1	432	46.481	6.39	1.78	8
182	Q8ND56	Protein LSM14 homolog A OS=Homo sapiens OX=9606 GN=LSM14A PE=1 SV=3	0	16.441	20	5	5	5	1	463	50.499	9.52	9.29	5
183	P12931	Proto-oncogene tyrosine-protein kinase Src OS=Homo sapiens OX=9606 GN=SRC PE=1 SV=3	0	16.395	56	6	10	6	1	536	59.797	7.42	9.48	6
184	Q9HCE1	Helicase MOV-10 OS=Homo sapiens OX=9606 GN=MOV10 PE=1 SV=2	0	16.346	28	2	3	2	1	1003	113.599	8.82	5.28	2
185	P22061	Protein-L-isoaspartate(D-aspartate) O-methyltransferase OS=Homo sapiens OX=9606 GN=PCMT1 PE=1 SV=	0	16.198	10	6	6	6	1	227	24.621	7.21	4.16	6
186	P21912	Succinate dehydrogenase [ubiquinone] iron-sulfur subunit, mitochondrial OS=Homo sapiens OX=9606 GN=S	0	16.109	12	5	5	5	1	280	31.609	8.76	6.06	5
187	Q9HD45	Transmembrane 9 superfamily member 3 OS=Homo sapiens OX=9606 GN=TM9SF3 PE=1 SV=2	0	15.877	16	6	12	2	1	589	67.843	7.21	15.52	6
188	P26639	Threonine--tRNA ligase 1, cytoplasmic OS=Homo sapiens OX=9606 GN=TARS1 PE=1 SV=3	0	15.833	10	6	7	6	1	723	83.382	6.67	3.51	6
189	P47914	60S ribosomal protein L29 OS=Homo sapiens OX=9606 GN=RPL29 PE=1 SV=2	0	15.765	8	5	8	4	1	159	17.741	11.66	4.18	5
190	P38606	V-type proton ATPase catalytic subunit A OS=Homo sapiens OX=9606 GN=ATP6V1A PE=1 SV=2	0	15.72	13	7	7	7	1	617	68.26	5.52	4.14	7
191	Q14683	Structural maintenance of chromosomes protein 1A OS=Homo sapiens OX=9606 GN=SMC1A PE=1 SV=2	0	15.69	33	4	5	4	1	1233	143.144	7.64	7.52	4
192	O43818	U3 small nucleolar RNA-interacting protein 2 OS=Homo sapiens OX=9606 GN=RRP9 PE=1 SV=1	0	15.684	6	6	6	6	1	475	51.809	7.85	5.55	6
193	P10155	60 kDa SS-A/Ro ribonucleoprotein OS=Homo sapiens OX=9606 GN=RO60 PE=1 SV=2	0	15.663	10	3	4	3	1	538	60.631	8.03	4.66	3
194	P28066	Proteasome subunit alpha type-5 OS=Homo sapiens OX=9606 GN=PSMA5 PE=1 SV=3	0	15.477	24	7	8	3	1	241	26.394	4.79	9.39	7
195	P62857	40S ribosomal protein S28 OS=Homo sapiens OX=9606 GN=RPS28 PE=1 SV=1	0	15.432	3	6	8	6	1	69	7.836	10.7	3.85	6
196	P42224	Signal transducer and activator of transcription 1-alpha/beta OS=Homo sapiens OX=9606 GN=STAT1 PE=1 S	0	15.402	15	3	5	3	1	750	87.28	6.05	8.89	3
197	P18077	60S ribosomal protein L35a OS=Homo sapiens OX=9606 GN=RPL35A PE=1 SV=2	0	15.294	21	6	14	3	1	110	12.53	11.06	12.28	6
198	Q15631	Translin OS=Homo sapiens OX=9606 GN=TSN PE=1 SV=1	0	15.167	10	7	7	7	1	228	26.167	6.44	7.85	7
199	P08708	40S ribosomal protein S17 OS=Homo sapiens OX=9606 GN=RPS17 PE=1 SV=2	0	15.093	4	6	6	6	1	135	15.54	9.85	5.02	6
200	P28074	Proteasome subunit beta type-5 OS=Homo sapiens OX=9606 GN=PSMB5 PE=1 SV=3	0	15.077	6	5	5	5	1	263	28.462	6.92	7.03	5
201	P62861	40S ribosomal protein S30 OS=Homo sapiens OX=9606 GN=FAU PE=1 SV=1	0	14.998	7	6	7	4	1	59	6.644	12.15	6.98	6
202	Q13242	Serine/arginine-rich splicing factor 9 OS=Homo sapiens OX=9606 GN=SRSF9 PE=1 SV=1	0	14.867	15	4	5	4	1	221	25.526	8.65	4.15	4
203	P67812	Signal peptidase complex catalytic subunit SEC11A OS=Homo sapiens OX=9606 GN=SEC11A PE=1 SV=1	0	14.862	12	7	7	6	1	179	20.612	9.48	1.76	7
204	P20618	Proteasome subunit beta type-1 OS=Homo sapiens OX=9606 GN=PSMB1 PE=1 SV=2	0	14.667	15	5	6	5	1	241	26.472	8.13	4.37	5
205	O60832	H/ACA ribonucleoprotein complex subunit DKC1 OS=Homo sapiens OX=9606 GN=DKC1 PE=1 SV=3	0	14.659	16	4	5	4	1	514	57.638	9.42	5.47	4
206	P45974	Ubiquitin carboxyl-terminal hydrolase 5 OS=Homo sapiens OX=9606 GN=USP5 PE=1 SV=2	0	14.593	5	6	6	6	1	858	95.725	5.03	0	6
207	Q14151	Scaffold attachment factor B2 OS=Homo sapiens OX=9606 GN=SAFB2 PE=1 SV=1	0	14.578	82	4	9	1	1	953	107.408	6.16	10.58	4
208	Q9UNM6	26S proteasome non-ATPase regulatory subunit 13 OS=Homo sapiens OX=9606 GN=PSMD13 PE=1 SV=2	0	14.57	17	4	4	4	1	376	42.918	5.81	8.04	4
209	Q02543	60S ribosomal protein L18a OS=Homo sapiens OX=9606 GN=RPL18A PE=1 SV=2	0	14.561	23	5	6	4	1	176	20.749	10.71	11.81	5
210	P25788	Proteasome subunit alpha type-3 OS=Homo sapiens OX=9606 GN=PSMA3 PE=1 SV=2	0	14.554	28	6	6	6	1	255	28.415	5.33	4.74	6
211	Q9BWM7	Sideroflexin-3 OS=Homo sapiens OX=9606 GN=SFXN3 PE=1 SV=3	0	14.439	30	5	6	5	1	321	35.481	9.1	8.41	5
212	P35998	26S proteasome regulatory subunit 7 OS=Homo sapiens OX=9606 GN=PSMC2 PE=1 SV=3	0	14.425	9	7	8	7	1	433	48.603	5.95	3.54	7
213	Q14103	Heterogeneous nuclear ribonucleoprotein D0 OS=Homo sapiens OX=9606 GN=HNRNPD PE=1 SV=1	0	14.283	24	4	10	2	1	355	38.41	7.81	6.53	4
214	Q63HN8	E3 ubiquitin-protein ligase RNF213 OS=Homo sapiens OX=9606 GN=RNF213 PE=1 SV=3	0	14.073	14	4	5	3	1	5207	591.03	6.48	4.52	4
215	P25311	Zinc-alpha-2-glycoprotein OS=Homo sapiens OX=9606 GN=AZGP1 PE=1 SV=2	0	13.977	9	5	6	5	1	298	34.237	6.05	6.48	5
216	P55084	Trifunctional enzyme subunit beta, mitochondrial OS=Homo sapiens OX=9606 GN=HADHB PE=1 SV=3	0	13.938	11	3	3	3	1	474	51.262	9.41	7.31	3
217	Q5T750	Skin-specific protein 32 OS=Homo sapiens OX=9606 GN=XP32 PE=1 SV=1	0	13.589	3	5	6	5	1	250	26.219	7.97	11.24	5
218	Q9BQ48	39S ribosomal protein L34, mitochondrial OS=Homo sapiens OX=9606 GN=MRPL34 PE=1 SV=1	0	13.586	38	5	5	5	1	92	10.159	12.25	8.66	5
219	Q9BRL6	Serine/arginine-rich splicing factor 8 OS=Homo sapiens OX=9606 GN=SRSF8 PE=1 SV=1	0	13.463	13	6	6	4	1	282	32.268	11.72	2.46	6
220	P27449	V-type proton ATPase 16 kDa proteolipid subunit OS=Homo sapiens OX=9606 GN=ATP6V0C PE=1 SV=1	0	13.346	18	4	5	4	1	155	15.725	8.44	5.35	4
221	Q9NZ01	Very-long-chain enoyl-CoA reductase OS=Homo sapiens OX=9606 GN=TECR PE=1 SV=1	0	13.32	9	1	5	1	1	308	36.011	9.45	4.5	1
222	P43307	Translocon-associated protein subunit alpha OS=Homo sapiens OX=9606 GN=SSR1 PE=1 SV=3	0	13.318	14	6	7	6	1	286	32.215	4.49	5.8	6
223	P25398	40S ribosomal protein S12 OS=Homo sapiens OX=9606 GN=RPS12 PE=1 SV=3	0	13.248	15	5	9	1	1	132	14.505	7.21	13.75	5
224	O00231	26S proteasome non-ATPase regulatory subunit 11 OS=Homo sapiens OX=9606 GN=PSMD11 PE=1 SV=3	0	13.177	2	4	6	4	1	422	47.434	6.48	6.83	4
225	P28072	Proteasome subunit beta type-6 OS=Homo sapiens OX=9606 GN=PSMB6 PE=1 SV=4	0	13.066	9	6	6	6	1	239	25.341	4.92	4.14	6
226	P49720	Proteasome subunit beta type-3 OS=Homo sapiens OX=9606 GN=PSMB3 PE=1 SV=2	0	13.005	13	6	7	6	1	205	22.933	6.55	6.51	6
227	P57088	Transmembrane protein 33 OS=Homo sapiens OX=9606 GN=TMEM33 PE=1 SV=2	0	12.903	20	6	6	6	1	247	27.96	9.7	3.64	6
228	Q15019	Septin-2 OS=Homo sapiens OX=9606 GN=SEPTIN2 PE=1 SV=1	0	12.866	20	5	6	5	1	361	41.461	6.6	4.08	5
229	P46782	40S ribosomal protein S5 OS=Homo sapiens OX=9606 GN=RPS5 PE=1 SV=4	0	12.812	8	3	3	3	1	204	22.862	9.72	1.96	3
230	Q8WUH6	Transmembrane protein 263 OS=Homo sapiens OX=9606 GN=TMEM263 PE=1 SV=1	0	12.808	9	6	8	6	1	116	11.741	9.32	4.2	6
231	O00487	26S proteasome non-ATPase regulatory subunit 14 OS=Homo sapiens OX=9606 GN=PSMD14 PE=1 SV=1	0	12.8	21	4	9	4	1	310	34.555	6.52	4.35	4
232	P62910	60S ribosomal protein L32 OS=Homo sapiens OX=9606 GN=RPL32 PE=1 SV=2	0	12.582	24	4	4	3	1	135	15.85	11.33	7.38	4
233	Q08257	Quinone oxidoreductase OS=Homo sapiens OX=9606 GN=CRYZ PE=1 SV=1	0	12.576	3	5	5	5	1	329	35.185	8.44	2.02	5
234	P34897	Serine hydroxymethyltransferase, mitochondrial OS=Homo sapiens OX=9606 GN=SHMT2 PE=1 SV=3	0	12.567	3	4	4	4	1	504	55.958	8.53	5.25	4
235	Q06323	Proteasome activator complex subunit 1 OS=Homo sapiens OX=9606 GN=PSME1 PE=1 SV=1	0	12.485	4	4	5	3	1	249	28.705	6.02	2.49	4
236	P62995	Transformer-2 protein homolog beta OS=Homo sapiens OX=9606 GN=TRA2B PE=1 SV=1	0	12.244	10	4	5	4	1	288	33.646	11.25	4.54	4
237	P25789	Proteasome subunit alpha type-4 OS=Homo sapiens OX=9606 GN=PSMA4 PE=1 SV=1	0	12.193	8	6	8	6	1	261	29.465	7.72	5.32	6
238	Q13428	Treacle protein OS=Homo sapiens OX=9606 GN=TCOF1 PE=1 SV=3	0	12.1	22	4	6	4	1	1488	152.015	9.04	7.49	4
239	P62273	40S ribosomal protein S29 OS=Homo sapiens OX=9606 GN=RPS29 PE=1 SV=2	0	12.074	3	3	3	3	1	56	6.672	10.13	0	3
240	P28070	Proteasome subunit beta type-4 OS=Homo sapiens OX=9606 GN=PSMB4 PE=1 SV=4	0	12.067	50	6	11	1	1	264	29.185	5.97	8.88	6
241	O43396	Thioredoxin-like protein 1 OS=Homo sapiens OX=9606 GN=TXNL1 PE=1 SV=3	0	12.047	15	5	6	5	1	289	32.231	4.96	2.1	5
242	Q9BQ52	Zinc phosphodiesterase ELAC protein 2 OS=Homo sapiens OX=9606 GN=ELAC2 PE=1 SV=2	0	11.952	3	5	5	1	1	826	92.16	7.9	4.13	5
243	Q53GQ0	Very-long-chain 3-oxoacyl-CoA reductase OS=Homo sapiens OX=9606 GN=HSD17B12 PE=1 SV=2	0	11.827	22	4	4	4	1	312	34.302	9.32	4.74	4
244	Q68DK2	Zinc finger FYVE domain-containing protein 26 OS=Homo sapiens OX=9606 GN=ZFYVE26 PE=1 SV=3	0	11.81	5	4	5	3	1	2539	284.395	6.39	5.89	4
245	Q9Y5A9	YTH domain-containing family protein 2 OS=Homo sapiens OX=9606 GN=YTHDF2 PE=1 SV=2	0	11.789	3	3	5	3	1	579	62.296	8.79	2.12	3
246	P17019	Zinc finger protein 708 OS=Homo sapiens OX=9606 GN=ZNF708 PE=1 SV=5	0	11.714	15	4	4	4	1	499	57.32	9.39	3.92	4
247	Q16650	T-box brain protein 1 OS=Homo sapiens OX=9606 GN=TBR1 PE=1 SV=1	0	11.713	28	3	3	2	1	682	74.007	7.33	3.04	3
248	Q13868	Exosome complex component RRP4 OS=Homo sapiens OX=9606 GN=EXOSC2 PE=1 SV=2	0	11.677	31	4	6	4	1	293	32.768	7.5	8.78	4
249	O60841	Eukaryotic translation initiation factor 5B OS=Homo sapiens OX=9606 GN=EIF5B PE=1 SV=4	0	11.631	19	4	6	4	1	1220	138.742	5.49	9.14	4
250	P23588	Eukaryotic translation initiation factor 4B OS=Homo sapiens OX=9606 GN=EIF4B PE=1 SV=2	0	11.61	8	4	7	3	1	611	69.11	5.73	6.35	4
251	Q9H4M9	EH domain-containing protein 1 OS=Homo sapiens OX=9606 GN=EHD1 PE=1 SV=2	0	11.581	12	3	3	3	1	534	60.589	6.83	4.9	3
252	Q92769	Histone deacetylase 2 OS=Homo sapiens OX=9606 GN=HDAC2 PE=1 SV=2	0	11.504	7	4	5	4	1	488	55.329	5.91	6.14	4
253	P24534	Elongation factor 1-beta OS=Homo sapiens OX=9606 GN=EEF1B2 PE=1 SV=3	0	11.468	4	4	4	4	1	225	24.748	4.67	5.62	4
254	Q9Y266	Nuclear migration protein nudC OS=Homo sapiens OX=9606 GN=NUDC PE=1 SV=1	0	11.463	6	4	5	4	1	331	38.219	5.38	7.79	4
255	P60228	Eukaryotic translation initiation factor 3 subunit E OS=Homo sapiens OX=9606 GN=EIF3E PE=1 SV=1	0	11.386	33	4	5	4	1	445	52.187	6.04	5.97	4
256	P49321	Nuclear autoantigenic sperm protein OS=Homo sapiens OX=9606 GN=NASP PE=1 SV=2	0	11.34	18	5	5	5	1	788	85.186	4.3	4.84	5
257	Q96A65	Exocyst complex component 4 OS=Homo sapiens OX=9606 GN=EXOC4 PE=1 SV=1	0	11.299	7	3	5	3	1	974	110.429	6.49	4.94	3
258	Q5RKV6	Exosome complex component MTR3 OS=Homo sapiens OX=9606 GN=EXOSC6 PE=1 SV=1	0	11.196	15	4	6	1	1	272	28.218	6.28	4.59	4
259	Q9H6R4	Nucleolar protein 6 OS=Homo sapiens OX=9606 GN=NOL6 PE=1 SV=2	0	11.121	5	3	3	3	1	1146	127.513	7.64	2.16	3
260	P55884	Eukaryotic translation initiation factor 3 subunit B OS=Homo sapiens OX=9606 GN=EIF3B PE=1 SV=3	0	11.111	4	3	3	3	1	814	92.424	5	2.69	3
261	P49736	DNA replication licensing factor MCM2 OS=Homo sapiens OX=9606 GN=MCM2 PE=1 SV=4	0	11.101	5	5	5	5	1	904	101.832	5.52	5.32	5
262	O00303	Eukaryotic translation initiation factor 3 subunit F OS=Homo sapiens OX=9606 GN=EIF3F PE=1 SV=1	0	11.007	11	3	4	2	1	357	37.54	5.45	4.79	3
263	Q04637	Eukaryotic translation initiation factor 4 gamma 1 OS=Homo sapiens OX=9606 GN=EIF4G1 PE=1 SV=4	0	10.971	8	3	5	1	1	1599	175.382	5.33	4.02	3
264	P41091	Eukaryotic translation initiation factor 2 subunit 3 OS=Homo sapiens OX=9606 GN=EIF2S3 PE=1 SV=3	0	10.968	23	3	4	2	1	472	51.077	8.4	2.71	3
265	O14980	Exportin-1 OS=Homo sapiens OX=9606 GN=XPO1 PE=1 SV=1	0	10.948	9	2	4	1	1	1071	123.306	6.06	2.86	2
266	P56537	Eukaryotic translation initiation factor 6 OS=Homo sapiens OX=9606 GN=EIF6 PE=1 SV=1	0	10.916	18	3	3	3	1	245	26.582	4.68	4.11	3
267	P20042	Eukaryotic translation initiation factor 2 subunit 2 OS=Homo sapiens OX=9606 GN=EIF2S2 PE=1 SV=2	0	10.886	19	3	4	3	1	333	38.364	5.8	6.37	3
268	P63241	Eukaryotic translation initiation factor 5A-1 OS=Homo sapiens OX=9606 GN=EIF5A PE=1 SV=2	0	10.846	5	4	4	4	1	154	16.821	5.24	1.89	4
269	P42356	Phosphatidylinositol 4-kinase alpha OS=Homo sapiens OX=9606 GN=PI4KA PE=1 SV=4	0	10.836	7	5	5	5	1	2102	236.678	7.06	2.39	5
270	Q15459	Splicing factor 3A subunit 1 OS=Homo sapiens OX=9606 GN=SF3A1 PE=1 SV=1	0	10.697	8	4	5	4	1	793	88.831	5.22	0	4
271	P09543	2',3'-cyclic-nucleotide 3'-phosphodiesterase OS=Homo sapiens OX=9606 GN=CNP PE=1 SV=2	0	10.62	44	5	8	5	1	421	47.549	9.07	7.8	5
272	P30043	Flavin reductase (NADPH) OS=Homo sapiens OX=9606 GN=BLVRB PE=1 SV=3	0	10.608	9	3	5	2	1	206	22.105	7.65	1.73	3
273	O60658	High affinity cAMP-specific and IBMX-insensitive 3',5'-cyclic phosphodiesterase 8A OS=Homo sapiens OX=	0	10.606	21	3	5	3	1	829	93.245	6.11	8.57	3
274	Q27J81	Inverted formin-2 OS=Homo sapiens OX=9606 GN=INF2 PE=1 SV=2	0	10.573	9	5	5	5	1	1249	135.54	5.38	4.03	5
275	O43772	Mitochondrial carnitine/acylcarnitine carrier protein OS=Homo sapiens OX=9606 GN=SLC25A20 PE=1 SV=	0	10.539	19	4	5	4	1	301	32.922	9.41	5.95	4
276	O14639	Actin-binding LIM protein 1 OS=Homo sapiens OX=9606 GN=ABLIM1 PE=1 SV=3	0	10.45	8	3	3	3	1	778	87.631	8.59	2.32	3
277	P28482	Mitogen-activated protein kinase 1 OS=Homo sapiens OX=9606 GN=MAPK1 PE=1 SV=3	0	10.444	41	6	9	1	1	360	41.363	6.98	0	6

1	**All interacting proteins of EIF1AX-NLS identified by Co-IP/MS**														
2	**Accessior**	**Description**	**Exp. q-val**	**Sum PEP Score**	**Coverage**	**# Peptid**	**# PSMs**	**# Unique I**	**# Prote**	**# AAs**	**MW [kDa**	**calc. pI**	**Score Seq**	**# Peptides**	**Sequest HT**
3	P47813	Eukaryotic translation initiation factor 1A, X-chromosomal OS=Homo sapiens OX=9606 GN=EIF1AX PE=1 SV=4	0	325.1213578	41.67450611	45	82	45	1	144	16.432	5.24	281.046	44	
4	O94832	Unconventional myosin-Id OS=Homo sapiens OX=9606 GN=MYO1D PE=1 SV=2	0	303.9870852	43.0417495	46	84	46	1	1006	116.129	9.39	213.442	46	
5	P38646	Stress-70 protein, mitochondrial OS=Homo sapiens OX=9606 GN=HSPA9 PE=1 SV=2	0	271.6231148	43.44624448	27	60	27	1	679	73.635	6.16	212.101	27	
6	Q9UM54	Unconventional myosin-VI OS=Homo sapiens OX=9606 GN=MYO6 PE=1 SV=4	0	168.026695	24.65224111	26	39	26	1	1294	149.596	8.53	108.119	26	
7	Q9ULV0	Unconventional myosin-Vb OS=Homo sapiens OX=9606 GN=MYO5B PE=1 SV=3	0	123.5129577	16.61255411	24	28	20	1	1848	213.538	7.2	74.531	24	
8	P02545	Prelamin-A/C OS=Homo sapiens OX=9606 GN=LMNA PE=1 SV=1	0	122.4309586	41.56626506	23	26	23	1	664	74.095	7.02	72.0637	23	
9	O00159	Unconventional myosin-Ic OS=Homo sapiens OX=9606 GN=MYO1C PE=1 SV=4	0	116.1827482	37.92599805	47	98	47	1	1063	121.606	9.41	256.932	47	
10	Q9NR30	Nucleolar RNA helicase 2 OS=Homo sapiens OX=9606 GN=DDX21 PE=1 SV=5	0	111.5221386	28.55683269	23	32	23	1	783	87.29	9.28	78.725	32	
11	Q96C19	EF-hand domain-containing protein D2 OS=Homo sapiens OX=9606 GN=EFHD2 PE=1 SV=1	0	106.1145053	51.25	16	29	16	1	240	26.68	5.2	78.0651	16	
12	P09327	Villin-1 OS=Homo sapiens OX=9606 GN=VIL1 PE=1 SV=4	0	105.0514728	32.64812576	23	26	23	1	827	92.637	6.39	70.2751	23	
13	P07900	Heat shock protein HSP 90-alpha OS=Homo sapiens OX=9606 GN=HSP90AA1 PE=1 SV=5	0	104.2705317	29.78142077	21	29	12	1	732	84.607	5.02	72.0598	21	
14	P17480	Nucleolar transcription factor 1 OS=Homo sapiens OX=9606 GN=UBTF PE=1 SV=1	0	101.6366857	25.23560209	3	3	3	1	764	89.35	5.81	35.983	13	
15	O43795	Unconventional myosin-Ib OS=Homo sapiens OX=9606 GN=MYO1B PE=1 SV=3	0	96.3528542	18.92605634	19	21	19	1	1136	131.902	9.38	55.17	19	
16	P12956	X-ray repair cross-complementing protein 6 OS=Homo sapiens OX=9606 GN=XRCC6 PE=1 SV=2	0	88.30963142	25.94417077	13	16	13	1	609	69.799	6.64	49.3992	13	
17	Q9NQX4	Unconventional myosin-Vc OS=Homo sapiens OX=9606 GN=MYO5C PE=1 SV=2	0	85.13611344	13.08840413	20	23	16	1	1742	202.682	7.71	48.628	20	
18	Q9Y230	RuvB-like 2 OS=Homo sapiens OX=9606 GN=RUVBL2 PE=1 SV=3	0	73.81269448	33.26133909	12	14	12	1	463	51.125	5.64	42.0497	12	
19	P13639	Elongation factor 2 OS=Homo sapiens OX=9606 GN=EEF2 PE=1 SV=4	0	69.92134145	15.5011655	12	17	11	1	858	95.277	6.83	42.8492	12	
20	P0CG38	POTE ankyrin domain family member I OS=Homo sapiens OX=9606 GN=POTEI PE=3 SV=1	0	69.17163969	7.162790698	8	48	1	1	1075	121.205	6.21	124.085	8	
21	P19338	Nucleolin OS=Homo sapiens OX=9606 GN=NCL PE=1 SV=3	0	67.00115468	18.45070423	13	14	13	1	710	76.568	4.7	38.7959	13	
22	Q9Y265	RuvB-like 1 OS=Homo sapiens OX=9606 GN=RUVBL1 PE=1 SV=1	0	63.52353343	25.65789474	9	11	9	1	456	50.196	6.42	34.4751	9	
23	O95425	Supervillin OS=Homo sapiens OX=9606 GN=SVIL PE=1 SV=2	0	57.65269238	7.226738934	11	11	11	1	2214	247.593	6.98	29.3656	11	
24	Q9Y4I1	Unconventional myosin-Va OS=Homo sapiens OX=9606 GN=MYO5A PE=1 SV=2	0	53.11306307	7.870619946	13	14	7	1	1855	215.269	8.48	26.4593	13	
25	P52272	Heterogeneous nuclear ribonucleoprotein M OS=Homo sapiens OX=9606 GN=HNRNPM PE=1 SV=3	0	51.63632445	19.04109589	11	14	11	1	730	77.464	8.7	35.0708	11	
26	P61978	Heterogeneous nuclear ribonucleoprotein K OS=Homo sapiens OX=9606 GN=HNRNPK PE=1 SV=1	0	46.51620285	21.81425486	8	10	8	1	463	50.944	5.54	28.8842	8	
27	P17987	T-complex protein 1 subunit alpha OS=Homo sapiens OX=9606 GN=TCP1 PE=1 SV=1	0	46.28286154	21.58273381	10	11	10	1	556	60.306	6.11	26.5127	10	
28	P11940	Polyadenylate-binding protein 1 OS=Homo sapiens OX=9606 GN=PABPC1 PE=1 SV=2	0	41.23368731	15.09433962	9	12	5	1	636	70.626	9.5	23.5787	9	
29	P31943	Heterogeneous nuclear ribonucleoprotein H OS=Homo sapiens OX=9606 GN=HNRNPH1 PE=1 SV=4	0	37.74966568	20.04454343	6	7	4	1	449	49.198	6.3	26.3101	6	
30	P06748	Nucleophosmin OS=Homo sapiens OX=9606 GN=NPM1 PE=1 SV=2	0	37.49300318	26.19047619	7	9	7	1	294	32.555	4.78	24.6367	7	
31	P22087	rRNA 2'-O-methyltransferase fibrillarin OS=Homo sapiens OX=9606 GN=FBL PE=1 SV=2	0	37.37314345	22.14953271	3	3	3	1	321	33.763	10.18	21.6034	7	
32	O00571	ATP-dependent RNA helicase DDX3X OS=Homo sapiens OX=9606 GN=DDX3X PE=1 SV=3	0	33.52885268	12.23564955	7	9	6	1	662	73.198	7.18	21.6222	7	
33	P50991	T-complex protein 1 subunit delta OS=Homo sapiens OX=9606 GN=CCT4 PE=1 SV=4	0	32.9975801	15.21335807	8	9	8	1	539	57.888	7.83	20.5484	8	
34	P67809	Y-box-binding protein 1 OS=Homo sapiens OX=9606 GN=YBX1 PE=1 SV=3	0	32.91984058	25	4	5	2	1	324	35.903	9.88	20.9796	4	
35	P50990	T-complex protein 1 subunit theta OS=Homo sapiens OX=9606 GN=CCT8 PE=1 SV=4	0	32.86431275	16.78832117	8	8	8	1	548	59.583	5.6	13.8576	8	
36	P13010	X-ray repair cross-complementing protein 5 OS=Homo sapiens OX=9606 GN=XRCC5 PE=1 SV=3	0	31.16369671	14.89071038	9	9	9	1	732	82.652	5.81	20.015	9	
37	P62937	Peptidyl-prolyl cis-trans isomerase A OS=Homo sapiens OX=9606 GN=PPIA PE=1 SV=2	0	30.64965319	28.48484848	7	10	7	1	165	18.001	7.81	21.5111	7	
38	P09651	Heterogeneous nuclear ribonucleoprotein A1 OS=Homo sapiens OX=9606 GN=HNRNPA1 PE=1 SV=5	0	30.64129692	17.47311828	6	12	5	1	372	38.723	9.13	24.8495	6	
39	P16615	Sarcoplasmic/endoplasmic reticulum calcium ATPase 2 OS=Homo sapiens OX=9606 GN=ATP2A2 PE=1 SV=1	0	30.44642301	9.404990403	8	9	8	1	1042	114.683	5.34	18.9608	8	
40	P62136	Serine/threonine-protein phosphatase PP1-alpha catalytic subunit OS=Homo sapiens OX=9606 GN=PPP1CA PE=1 S	0	29.40520205	13.33333333	4	6	3	1	330	37.488	6.33	16.3058	4	
41	P52597	Heterogeneous nuclear ribonucleoprotein F OS=Homo sapiens OX=9606 GN=HNRNPF PE=1 SV=3	0	29.3846574	16.38554217	5	5	3	1	415	45.643	5.58	15.3359	5	
42	Q13310	Polyadenylate-binding protein 4 OS=Homo sapiens OX=9606 GN=PABPC4 PE=1 SV=1	0	28.51368748	10.86956522	6	8	2	1	644	70.738	9.26	14.914	6	
43	P15531	Nucleoside diphosphate kinase A OS=Homo sapiens OX=9606 GN=NME1 PE=1 SV=1	0	28.51309384	40.13157895	5	7	2	1	152	17.138	6.19	21.2306	5	
44	P33993	DNA replication licensing factor MCM7 OS=Homo sapiens OX=9606 GN=MCM7 PE=1 SV=4	0	28.27790316	12.65646732	9	9	9	1	719	81.257	6.46	16.8473	9	
45	P40227	T-complex protein 1 subunit zeta OS=Homo sapiens OX=9606 GN=CCT6A PE=1 SV=3	0	28.04552419	20.33898305	9	10	9	1	531	57.988	6.68	17.7421	9	
46	P51991	Heterogeneous nuclear ribonucleoprotein A3 OS=Homo sapiens OX=9606 GN=HNRNPA3 PE=1 SV=2	0	27.51467051	23.28042328	6	9	5	1	378	39.571	9.01	24.174	6	
47	P0C0S8	Histone H2A type 1 OS=Homo sapiens OX=9606 GN=H2AC11 PE=1 SV=2	0	26.76740761	35.38461538	4	17	1	1	130	14.083	10.9	35.4775	4	
48	Q06830	Peroxiredoxin-1 OS=Homo sapiens OX=9606 GN=PRDX1 PE=1 SV=1	0	26.64008098	36.18090452	7	9	6	1	199	22.096	8.13	19.0873	7	
49	P21796	Voltage-dependent anion-selective channel protein 1 OS=Homo sapiens OX=9606 GN=VDAC1 PE=1 SV=2	0	25.18819254	22.96819788	4	4	3	1	283	30.754	8.54	11.8257	4	
50	P25205	DNA replication licensing factor MCM3 OS=Homo sapiens OX=9606 GN=MCM3 PE=1 SV=3	0	25.00396911	11.13861386	7	7	7	1	808	90.924	5.77	13.307	7	
51	O60506	Heterogeneous nuclear ribonucleoprotein Q OS=Homo sapiens OX=9606 GN=SYNCRIP PE=1 SV=2	0	24.7427054	12.03852327	6	6	3	1	623	69.56	8.59	16.1421	6	
52	Q15233	Non-POU domain-containing octamer-binding protein OS=Homo sapiens OX=9606 GN=NONO PE=1 SV=4	0	23.64636621	14.64968153	5	6	4	1	471	54.197	8.95	14.8195	5	
53	O75083	WD repeat-containing protein 1 OS=Homo sapiens OX=9606 GN=WDR1 PE=1 SV=4	0	23.00761066	10.89108911	5	6	5	1	606	66.152	6.65	14.5337	5	
54	P18206	Vinculin OS=Homo sapiens OX=9606 GN=VCL PE=1 SV=4	0	21.08773429	5.291005291	5	5	5	1	1134	123.722	5.66	11.9996	5	
55	P22392	Nucleoside diphosphate kinase B OS=Homo sapiens OX=9606 GN=NME2 PE=1 SV=1	0	20.78938168	30.92105263	4	6	1	1	152	17.287	8.41	17.9672	4	
56	P16989	Y-box-binding protein 3 OS=Homo sapiens OX=9606 GN=YBX3 PE=1 SV=4	0	19.93678692	13.70967742	3	4	1	1	372	40.066	9.77	13.4424	3	
57	P32119	Peroxiredoxin-2 OS=Homo sapiens OX=9606 GN=PRDX2 PE=1 SV=5	0	17.88901559	32.32323232	6	6	5	1	198	21.878	5.97	14.2242	6	
58	P23284	Peptidyl-prolyl cis-trans isomerase B OS=Homo sapiens OX=9606 GN=PPIB PE=1 SV=2	0	17.37094806	25	5	5	5	1	216	23.728	9.41	9.60972	5	
59	P62140	Serine/threonine-protein phosphatase PP1-beta catalytic subunit OS=Homo sapiens OX=9606 GN=PPP1CB PE=1 SV	0	17.34647105	7.339449541	2	3	1	1	327	37.163	6.19	11.289	2	
60	Q92616	eIF-2-alpha kinase activator GCN1 OS=Homo sapiens OX=9606 GN=GCN1 PE=1 SV=6	0	17.17839516	2.283788843	5	5	5	1	2671	292.572	7.47	10.3119	5	
61	Q9UHD8	Septin-9 OS=Homo sapiens OX=9606 GN=SEPTIN9 PE=1 SV=2	0	16.50384007	8.19112628	4	4	4	1	586	65.361	8.97	7.41019	4	
62	Q13838	Spliceosome RNA helicase DDX39B OS=Homo sapiens OX=9606 GN=DDX39B PE=1 SV=1	0	16.0200845	11.44859813	5	5	5	1	428	48.96	5.67	10.6857	5	
63	O95373	Importin-7 OS=Homo sapiens OX=9606 GN=IPO7 PE=1 SV=1	0	15.76266192	4.238921002	4	4	4	1	1038	119.44	4.82	9.67124	4	
64	Q6UWP8	Suprabasin OS=Homo sapiens OX=9606 GN=SBSN PE=1 SV=2	0	15.47386913	9.152542373	2	2	2	1	590	60.505	7.01	9.51932	2	
65	P35637	RNA-binding protein FUS OS=Homo sapiens OX=9606 GN=FUS PE=1 SV=1	0	14.78043949	6.273764259	3	3	3	1	526	53.394	9.36	8.59698	3	
66	Q13347	Eukaryotic translation initiation factor 3 subunit I OS=Homo sapiens OX=9606 GN=EIF3I PE=1 SV=1	0	14.49583184	16	5	5	5	1	325	36.479	5.64	9.12805	5	
67	P84103	Serine/arginine-rich splicing factor 3 OS=Homo sapiens OX=9606 GN=SRSF3 PE=1 SV=1	0	14.21288539	25.6097561	4	4	3	1	164	19.318	11.65	11.2201	4	
68	Q9Y383	Putative RNA-binding protein Luc7-like 2 OS=Homo sapiens OX=9606 GN=LUC7L2 PE=1 SV=2	0	14.13047878	10.45918367	3	3	3	1	392	46.486	10.01	6.49736	3	
69	P78371	T-complex protein 1 subunit beta OS=Homo sapiens OX=9606 GN=CCT2 PE=1 SV=4	0	13.99071687	8.598130841	3	3	3	1	535	57.452	6.46	6.46533	3	
70	P31689	DnaJ homolog subfamily A member 1 OS=Homo sapiens OX=9606 GN=DNAJA1 PE=1 SV=2	0	13.95075431	12.84634761	3	3	3	1	397	44.839	7.08	9.19403	3	
71	P30153	Serine/threonine-protein phosphatase 2A 65 kDa regulatory subunit A alpha isoform OS=Homo sapiens OX=9606 (	0	13.60850088	6.451612903	3	3	3	1	589	65.267	5.11	8.73109	3	
72	P49368	T-complex protein 1 subunit gamma OS=Homo sapiens OX=9606 GN=CCT3 PE=1 SV=4	0	13.51807012	6.055045872	3	3	3	1	545	60.495	6.49	8.1589	3	
73	Q01844	RNA-binding protein EWS OS=Homo sapiens OX=9606 GN=EWSR1 PE=1 SV=1	0	12.8441329	4.420731707	2	2	2	1	656	68.436	9.33	6.32408	2	
74	Q8IWV7	E3 ubiquitin-protein ligase UBR1 OS=Homo sapiens OX=9606 GN=UBR1 PE=1 SV=1	0	12.61789094	2.572898799	4	4	4	1	1749	200.08	6.01	9.12571	4	
75	Q9UQ35	Serine/arginine repetitive matrix protein 2 OS=Homo sapiens OX=9606 GN=SRRM2 PE=1 SV=2	0	12.43392401	1.707848837	4	4	4	1	2752	299.438	12.06	6.90403	4	
76	P11387	DNA topoisomerase 1 OS=Homo sapiens OX=9606 GN=TOP1 PE=1 SV=2	0	11.93353581	5.620915033	4	4	4	1	765	90.669	9.31	5.35838	4	
77	Q09028	Histone-binding protein RBBP4 OS=Homo sapiens OX=9606 GN=RBBP4 PE=1 SV=3	0	11.7400635	5.411764706	3	4	3	1	425	47.626	4.89	3.63819	3	
78	Q15717	ELAV-like protein 1 OS=Homo sapiens OX=9606 GN=ELAVL1 PE=1 SV=2	0	11.66943951	9.509202454	3	3	3	1	326	36.069	9.17	7.77626	3	
79	P31948	Stress-induced-phosphoprotein 1 OS=Homo sapiens OX=9606 GN=STIP1 PE=1 SV=1	0	11.38078431	5.893186004	3	3	3	1	543	62.599	6.8	7.09621	3	
80	O15231	Zinc finger protein 185 OS=Homo sapiens OX=9606 GN=ZNF185 PE=1 SV=3	0	11.21612969	6.095791001	3	3	3	1	689	73.48	7.01	9.18527	3	
81	Q16629	Serine/arginine-rich splicing factor 7 OS=Homo sapiens OX=9606 GN=SRSF7 PE=1 SV=1	0	11.0194815	12.60504202	3	3	2	1	238	27.35	11.82	9.35151	3	
82	P26641	Elongation factor 1-gamma OS=Homo sapiens OX=9606 GN=EEF1G PE=1 SV=3	0	10.92072761	4.576659039	2	3	2	1	437	50.087	6.67	7.16774	2	
83	Q9Y2X3	Nucleolar protein 58 OS=Homo sapiens OX=9606 GN=NOP58 PE=1 SV=1	0	10.43717874	5.671077505	2	3	2	1	529	59.541	8.92	6.92475	2	
84	Q14566	DNA replication licensing factor MCM6 OS=Homo sapiens OX=9606 GN=MCM6 PE=1 SV=1	0	10.39399676	4.384896468	3	3	3	1	821	92.831	5.41	6.59328	3	
85	P29692	Elongation factor 1-delta OS=Homo sapiens OX=9606 GN=EEF1D PE=1 SV=5	0	10.20957724	12.09964413	3	3	3	1	281	31.103	5.01	4.52385	3	
86	Q8IWV8	E3 ubiquitin-protein ligase UBR2 OS=Homo sapiens OX=9606 GN=UBR2 PE=1 SV=1	0	9.734918848	1.424501425	2	2	2	1	1755	200.408	6.24	5.82466	2	
87	Q99832	T-complex protein 1 subunit eta OS=Homo sapiens OX=9606 GN=CCT7 PE=1 SV=2	0	9.327340711	8.47145488	4	4	4	1	543	59.329	7.65	4.86069	4	
88	Q13151	Heterogeneous nuclear ribonucleoprotein A0 OS=Homo sapiens OX=9606 GN=HNRNPA0 PE=1 SV=1	0	8.883977919	7.540983607	2	4	1	1	305	30.822	9.29	8.80959	2	
89	Q92621	Nuclear pore complex protein Nup205 OS=Homo sapiens OX=9606 GN=NUP205 PE=1 SV=3	0	8.865498221	1.789264414	3	3	3	1	2012	227.776	6.19	6.40539	3	
90	B5ME19	Eukaryotic translation initiation factor 3 subunit C-like protein OS=Homo sapiens OX=9606 GN=EIF3CL PE=1 SV	0	8.435058536	2.84463895	2	2	2	1	914	105.407	5.64	4.70502	2	
91	Q07955	Serine/arginine-rich splicing factor 1 OS=Homo sapiens OX=9606 GN=SRSF1 PE=1 SV=2	0	8.173255474	13.30645161	2	2	2	1	248	27.728	10.36	6.67548	2	
92	Q00325	Phosphate carrier protein, mitochondrial OS=Homo sapiens OX=9606 GN=SLC25A3 PE=1 SV=2	0	8.047858632	11.32596685	4	4	4	1	362	40.069	9.38	4.67574	4	
93	Q99729	Heterogeneous nuclear ribonucleoprotein A/B OS=Homo sapiens OX=9606 GN=HNRNPAB PE=1 SV=2	0	8.006467133	6.325301205	2	2	2	1	332	36.202	8.21	3.09701	2	
94	P0C0S5	Histone H2A.Z OS=Homo sapiens OX=9606 GN=H2AZ1 PE=1 SV=2	0	8.001879281	20.3125	3	12	1	1	128	13.545	10.58	16.532	3	
95	Q14152	Eukaryotic translation initiation factor 3 subunit A OS=Homo sapiens OX=9606 GN=EIF3A PE=1 SV=1	0	7.870859896	1.519536903	2	2	2	1	1382	166.468	6.79	5.67748	2	
96	P14866	Heterogeneous nuclear ribonucleoprotein L OS=Homo sapiens OX=9606 GN=HNRNPL PE=1 SV=2	0	7.851031277	5.093378608	3	3	3	1	589	64.092	8.22	5.29689	3	
97	Q01813	ATP-dependent 6-phosphofructokinase, platelet type OS=Homo sapiens OX=9606 GN=PFKP PE=1 SV=2	0	7.544970459	2.933673469	2	2	2	1	784	85.542	7.55	4.60914	2	
98	P55209	Nucleosome assembly protein 1-like 1 OS=Homo sapiens OX=9606 GN=NAP1L1 PE=1 SV=1	0	7.544546031	4.347826087	1	1	1	1	391	45.346	4.46	3.66428	1	
99	Q9Y277	Voltage-dependent anion-selective channel protein 3 OS=Homo sapiens OX=9606 GN=VDAC3 PE=1 SV=1	0	7.446753961	8.127208481	2	2	1	1	283	30.639	8.66	4.63926	2	
100	Q13501	Sequestosome-1 OS=Homo sapiens OX=9606 GN=SQSTM1 PE=1 SV=1	0	6.862013267	5.909090909	1	1	1	1	440	47.657	5.22	5.04992	1	
101	Q14978	Nucleolar and coiled-body phosphoprotein 1 OS=Homo sapiens OX=9606 GN=NOLC1 PE=1 SV=2	0	6.861831875	3.719599428	2	2	2	1	699	73.56	9.47	6.17766	2	
102	P30041	Peroxiredoxin-6 OS=Homo sapiens OX=9606 GN=PRDX6 PE=1 SV=3	0	6.544773601	16.51785714	3	3	3	1	224	25.019	6.38	3.73859	3	
103	P22102	Trifunctional purine biosynthetic protein adenosine-3 OS=Homo sapiens OX=9606 GN=GART PE=1 SV=1	0	6.398026007	2.277227723	2	2	2	1	1010	107.699	6.7	3.91795	2	
104	P63241	Eukaryotic translation initiation factor 5A-1 OS=Homo sapiens OX=9606 GN=EIF5A PE=1 SV=2	0	6.376815673	15.58441558	2	2	2	1	154	16.821	5.24	5.59842	2	
105	P31942	Heterogeneous nuclear ribonucleoprotein H3 OS=Homo sapiens OX=9606 GN=HNRNPH3 PE=1 SV=2	0	6.070127601	7.803468208	2	2	2	1	346	36.903	6.87	5.82147	2	
106	P26599	Polypyrimidine tract-binding protein 1 OS=Homo sapiens OX=9606 GN=PTBP1 PE=1 SV=1	0	5.988362943	8.474576271	4	4	4	1	531	57.186	9.17	3.71467	4	
107	P20042	Eukaryotic translation initiation factor 2 subunit 2 OS=Homo sapiens OX=9606 GN=EIF2S2 PE=1 SV=2	0	5.978371913	5.105105105	2	2	2	1	333	38.364	5.8	3.86015	2	
108	Q9Y3Y2	Chromatin target of PRMT1 protein OS=Homo sapiens OX=9606 GN=CHTOP PE=1 SV=2	0	5.678485558	9.274193548	2	2	2	1	248	26.38	12.23	4.79659	2	
109	Q5SSJ5	Heterochromatin protein 1-binding protein 3 OS=Homo sapiens OX=9606 GN=HP1BP3 PE=1 SV=1	0	5.672714646	3.435804702	2	2	2	1	553	61.169	9.67	4.07545	2	
110	Q14974	Importin subunit beta-1 OS=Homo sapiens OX=9606 GN=KPNB1 PE=1 SV=2	0	5.602922139	2.397260274	2	2	2	1	876	97.108	4.78	2.73728	2	
111	O00567	Nucleolar protein 56 OS=Homo sapiens OX=9606 GN=NOP56 PE=1 SV=4	0	5.602617462	3.198653199	2	2	2	1	594	66.009	9.19	4.53796	2	
112	Q96HS1	Serine/threonine-protein phosphatase PGAM5, mitochondrial OS=Homo sapiens OX=9606 GN=PGAM5 PE=1 SV=	0	5.479952159	6.574394464	2	2	2	1	289	31.985	8.68	4.52282	2	
113	Q15365	Poly(rC)-binding protein 1 OS=Homo sapiens OX=9606 GN=PCBP1 PE=1 SV=2	0	5.313839839	6.179775281	2	2	2	1	356	37.474	7.09	1.75278	2	
114	Q16181	Septin-7 OS=Homo sapiens OX=9606 GN=SEPTIN7 PE=1 SV=2	0	5.301725423	2.745995423	1	1	1	1	437	50.648	8.63	2.37556	1	
115	P62714	Serine/threonine-protein phosphatase 2A catalytic subunit beta isoform OS=Homo sapiens OX=9606 GN=PPP2CB F	0	5.300422409	3.55987055	1	1	1	1	309	35.552	5.43	2.55681	1	
116	Q9NTJ3	Structural maintenance of chromosomes protein 4 OS=Homo sapiens OX=9606 GN=SMC4 PE=1 SV=2	0	5.231361899	0.854037267	1	1	1	1	1288	147.091	6.79	2.39472	1	
117	O60664	Perilipin-3 OS=Homo sapiens OX=9606 GN=PLIN3 PE=1 SV=3	0	5.144943514	6.912442396	2	2	2	1	434	47.046	5.44	4.27512	2	
118	P45880	Voltage-dependent anion-selective channel protein 2 OS=Homo sapiens OX=9606 GN=VDAC2 PE=1 SV=2	0	5.109699952	6.802721088	2	2	2	1	294	31.547	7.56	4.02619	2	
119	P37802	Transgelin-2 OS=Homo sapiens OX=9606 GN=TAGLN2 PE=1 SV=3	0	5.074756411	11.05527638	2	2	2	1	199	22.377	8.25	2.51777	2	
120	Q9NVP1	ATP-dependent RNA helicase DDX18 OS=Homo sapiens OX=9606 GN=DDX18 PE=1 SV=2	0	5.071588784	3.731343284	2	2	2	1	670	75.359	9.5	2.41856	2	
121	Q9P035	Very-long-chain (3R)-3-hydroxyacyl-CoA dehydratase 3 OS=Homo sapiens OX=9606 GN=HACD3 PE=1 SV=2	0	5.06469431	3.867403315	1	1	1	1	362	43.132	8.94	2.81864	1	
122	O75494	Serine/arginine-rich splicing factor 10 OS=Homo sapiens OX=9606 GN=SRSF10 PE=1 SV=1	0	5.034469056	4.198473282	1	1	1	1	262	31.282	11.27	2.71546	1	
123	P56537	Eukaryotic translation initiation factor 6 OS=Homo sapiens OX=9606 GN=EIF6 PE=1 SV=1	0	4.919734373	7.346938776	1	1	1	1	245	26.582	4.68	3.12092	1	
124	Q9P0L0	Vesicle-associated membrane protein-associated protein A OS=Homo sapiens OX=9606 GN=VAPA PE=1 SV=3	0	4.919373513	4.819277108	1	1	1	1	249	27.875	8.62	3.04049	1	
125	Q14141	Septin-6 OS=Homo sapiens OX=9606 GN=SEPTIN6 PE=1 SV=4	0	4.687399561	2.534562212	1	1	1	1	434	49.685	6.67	1.69813	1	
126	P15313	V-type proton ATPase subunit B, kidney isoform OS=Homo sapiens OX=9606 GN=ATP6V1B1 PE=1 SV=3	0	4.597394758	1.949317739	1	1	1	1	513	56.797	5.66	2.71152	1	
127	Q96I99	Succinate--CoA ligase [GDP-forming] subunit beta, mitochondrial OS=Homo sapiens OX=9606 GN=SUCLG2 PE=	0	4.546987609	2.314814815	1	1	1	1	432	46.481	6.39	2.61719	1	
128	Q8ND56	Protein LSM14 homolog A OS=Homo sapiens OX=9606 GN=LSM14A PE=1 SV=3	0	4.48098888	4.75161987	2	2	2	1	463	50.499	9.52	4.59247	2	
129	Q9HCE1	Helicase MOV-10 OS=Homo sapiens OX=9606 GN=MOV10 PE=1 SV=2	0	4.27679069	1.395812562	1	1	1	1	1003	113.599	8.82	2.40174	1	
130	Q09161	Nuclear cap-binding protein subunit 1 OS=Homo sapiens OX=9606 GN=NCBP1 PE=1 SV=1	0	4.233587153	1.392405063	1	1	1	1	790	91.781	6.43	1.86033	1	
131	P21912	Succinate dehydrogenase [ubiquinone] iron-sulfur subunit, mitochondrial OS=Homo sapiens OX=9606 GN=SDHB	0	4.143210711	3.571428571	1	1	1	1	280	31.609	8.76	2.6625	1	
132	O14980	Exportin-1 OS=Homo sapiens OX=9606 GN=XPO1 PE=1 SV=1	0	4.114751892	1.774042951	1	1	1	1	1071	123.306	6.06	2.84928	1	
133	P41091	Eukaryotic translation initiation factor 2 subunit 3 OS=Homo sapiens OX=9606 GN=EIF2S3 PE=1 SV=3	0	4.065804651	2.330508475	1	1	1	1	472	51.077	8.4	3.35058	1	
134	P38606	V-type proton ATPase catalytic subunit A OS=Homo sapiens OX=9606 GN=ATP6V1A PE=1 SV=2	0	3.920947686	3.889789303	2	2	2	1	617	68.26	5.52	4.30629	2	
135	Q14683	Structural maintenance of chromosomes protein 1A OS=Homo sapiens OX=9606 GN=SMC1A PE=1 SV=2	0	3.919012953	1.216545012	1	1	1	1	1233	143.144	7.64	2.5238	1	
136	O43818	U3 small nucleolar RNA-interacting protein 2 OS=Homo sapiens OX=9606 GN=RRP9 PE=1 SV=1	0	3.915781313	2.315789474	1	1	1	1	475	51.809	7.85	2.27556	1	
137	P19388	DNA-directed RNA polymerases I, II, and III subunit RPABC1 OS=Homo sapiens OX=9606 GN=POLR2E PE=1 SV	0	3.784891419	4.761904762	1	1	1	1	210	24.536	5.95	2.78076	1	
138	Q04637	Eukaryotic translation initiation factor 4 gamma 1 OS=Homo sapiens OX=9606 GN=EIF4G1 PE=1 SV=4	0	3.781464495	0.687929956	1	1	1	1	1599	175.382	5.33	2.44457	1	
139	Q99615	DnaJ homolog subfamily C member 7 OS=Homo sapiens OX=9606 GN=DNAJC7 PE=1 SV=2	0	3.658367664	2.631578947	1	1	1	1	494	56.405	6.96	2.36272	1	
140	O00303	Eukaryotic translation initiation factor 3 subunit F OS=Homo sapiens OX=9606 GN=EIF3F PE=1 SV=1	0	3.626904013	3.081232493	1	1	1	1	357	37.54	5.45	2.93211	1	
141	P49736	DNA replication licensing factor MCM2 OS=Homo sapiens OX=9606 GN=MCM2 PE=1 SV=4	0.00116	3.619426997	1.327433628	1	1	1	1	904	101.832	5.52	2.45152	1	
142	P18077	60S ribosomal protein L35a OS=Homo sapiens OX=9606 GN=RPL35A PE=1 SV=2	0.00116	3.561141341	9.090909091	1	1	1	1	110	12.53	11.06	2.70852	1	
143	Q15631	Translin OS=Homo sapiens OX=9606 GN=TSN PE=1 SV=1	0.00116	3.532836034	4.385964912	1	1	1	1	228	26.167	6.44	2.53268	1	
144	P08708	40S ribosomal protein S17 OS=Homo sapiens OX=9606 GN=RPS17 PE=1 SV=2	0.00216	3.503347061	7.407407407	1	1	1	1	135	15.54	9.85	2.48268	1	
145	P28074	Proteasome subunit beta type-5 OS=Homo sapiens OX=9606 GN=PSMB5 PE=1 SV=3	0.00216	3.477164686	5.323193916	1	1	1	1	263	28.462	6.92	0	1	
146	P55884	Eukaryotic translation initiation factor 3 subunit B OS=Homo sapiens OX=9606 GN=EIF3B PE=1 SV=3	0.00216	3.475733731	1.228501229	1	1	1	1	814	92.424	5	2.65536	1	

147	P62861	40S ribosomal protein S30 OS=Homo sapiens OX=9606 GN=FAU PE=1 SV=1	0.00216	3.394694954	16.94915254	1	2	1	1	59	6.644	12.15	3.83875	1
148	Q13242	Serine/arginine-rich splicing factor 9 OS=Homo sapiens OX=9606 GN=SRSF9 PE=1 SV=1	0.00216	3.356251315	4.524886878	1	1	1	1	221	25.526	8.65	2.33389	1
149	P20618	Proteasome subunit beta type-1 OS=Homo sapiens OX=9606 GN=PSMB1 PE=1 SV=2	0.00216	3.284247283	4.149377593	1	1	1	1	241	26.472	8.13	2.27809	1
150	Q9H6R4	Nucleolar protein 6 OS=Homo sapiens OX=9606 GN=NOL6 PE=1 SV=2	0.00216	3.234108024	1.308900524	1	1	1	1	1146	127.513	7.64	0	1
151	O60832	H/ACA ribonucleoprotein complex subunit DKC1 OS=Homo sapiens OX=9606 GN=DKC1 PE=1 SV=3	0.00216	3.219106891	1.945525292	1	1	1	1	514	57.638	9.42	2.34984	1
152	P45974	Ubiquitin carboxyl-terminal hydrolase 5 OS=Homo sapiens OX=9606 GN=USP5 PE=1 SV=2	0.00216	3.177308989	1.398601399	1	1	1	1	858	95.725	5.03	1.78106	1
153	Q5RKV6	Exosome complex component MTR3 OS=Homo sapiens OX=9606 GN=EXOSC6 PE=1 SV=1	0.00216	3.175483672	5.882352941	1	1	1	1	272	28.218	6.28	1.97494	1
154	Q14151	Scaffold attachment factor B2 OS=Homo sapiens OX=9606 GN=SAFB2 PE=1 SV=1	0.00216	3.12395545	0.944386149	1	1	1	1	953	107.408	6.16	2.18446	1
155	Q9UNM(	26S proteasome non-ATPase regulatory subunit 13 OS=Homo sapiens OX=9606 GN=PSMD13 PE=1 SV=2	0.00216	2.990549104	2.393617021	1	1	1	1	376	42.918	5.81	1.68519	1
156	Q16625	Occludin OS=Homo sapiens OX=9606 GN=OCLN PE=1 SV=1	0.00216	2.879097182	1.915708812	1	1	1	1	522	59.106	6.09	0	1
157	Q99497	Parkinson disease protein 7 OS=Homo sapiens OX=9606 GN=PARK7 PE=1 SV=2	0.00216	2.834397581	11.64021164	2	2	2	1	189	19.878	6.79	1.68979	2
158	Q02543	60S ribosomal protein L18a OS=Homo sapiens OX=9606 GN=RPL18A PE=1 SV=2	0.00216	2.825940192	5.681818182	1	1	1	1	176	20.749	10.71	2.46711	1
159	Q96A65	Exocyst complex component 4 OS=Homo sapiens OX=9606 GN=EXOC4 PE=1 SV=1	0.00216	2.785421046	1.232032854	1	1	1	1	974	110.429	6.49	1.94345	1
160	P25788	Proteasome subunit alpha type-3 OS=Homo sapiens OX=9606 GN=PSMA3 PE=1 SV=2	0.00216	2.72607322	4.705882353	1	1	1	1	255	28.415	5.33	2.83372	1
161	Q9BWM'	Sideroflexin-3 OS=Homo sapiens OX=9606 GN=SFXN3 PE=1 SV=3	0.00216	2.721246399	2.803738318	1	1	1	1	321	35.481	9.1	0	1
162	P35998	26S proteasome regulatory subunit 7 OS=Homo sapiens OX=9606 GN=PSMC2 PE=1 SV=3	0.00216	2.688458042	2.540415704	1	1	1	1	433	48.603	5.95	2.59104	1
163	Q14103	Heterogeneous nuclear ribonucleoprotein D0 OS=Homo sapiens OX=9606 GN=HNRNPD PE=1 SV=1	0.00216	2.675511767	2.253521127	1	1	1	1	355	38.41	7.81	2.12814	1
164	Q63HN8	E3 ubiquitin-protein ligase RNF213 OS=Homo sapiens OX=9606 GN=RNF213 PE=1 SV=3	0.00317	2.660548559	0.345688496	1	1	1	1	5207	591.03	6.48	0	1
165	P25311	Zinc-alpha-2-glycoprotein OS=Homo sapiens OX=9606 GN=AZGP1 PE=1 SV=2	0.00317	2.659555885	3.355704698	1	1	1	1	298	34.237	6.05	2.70244	1
166	P55084	Trifunctional enzyme subunit beta, mitochondrial OS=Homo sapiens OX=9606 GN=HADHB PE=1 SV=3	0.00317	2.606951534	2.742616034	1	1	1	1	474	51.262	9.41	2.15863	1
167	Q5T750	Skin-specific protein 32 OS=Homo sapiens OX=9606 GN=XP32 PE=1 SV=1	0.00317	2.590912631	3.2	1	1	1	1	250	26.219	7.97	2.15693	1
168	P49321	Nuclear autoantigenic sperm protein OS=Homo sapiens OX=9606 GN=NASP PE=1 SV=2	0.00317	2.542723814	1.269035533	1	1	1	1	788	85.186	4.3	2.16368	1
169	Q9BQ48	39S ribosomal protein L34, mitochondrial OS=Homo sapiens OX=9606 GN=MRPL34 PE=1 SV=1	0.00317	2.534319788	10.86956522	1	1	1	1	92	10.159	12.25	1.7021	1
170	Q9BRL6	Serine/arginine-rich splicing factor 8 OS=Homo sapiens OX=9606 GN=SRSF8 PE=1 SV=1	0.00387	2.50751899	2.482269504	1	1	1	1	282	32.268	11.72	2.05936	1
171	P27449	V-type proton ATPase 16 kDa proteolipid subunit OS=Homo sapiens OX=9606 GN=ATP6V0C PE=1 SV=1	0.00387	2.472241247	11.61290323	1	1	1	1	155	15.725	8.44	2.89658	1
172	P60228	Eukaryotic translation initiation factor 3 subunit E OS=Homo sapiens OX=9606 GN=EIF3E PE=1 SV=1	0.00387	2.462306806	2.247191011	1	1	1	1	445	52.187	6.04	2.39186	1
173	O75746	Calcium-binding mitochondrial carrier protein Aralar1 OS=Homo sapiens OX=9606 GN=SLC25A12 PE=1 SV=2	0.00387	2.397722916	1.179941003	1	1	1	1	678	74.715	8.38	1.98432	1
174	Q9NZ01	Very-long-chain enoyl-CoA reductase OS=Homo sapiens OX=9606 GN=TECR PE=1 SV=1	0.00387	2.371917739	2.272727273	1	1	1	1	308	36.011	9.45	1.94596	1
175	P43307	Translocon-associated protein subunit alpha OS=Homo sapiens OX=9606 GN=SSR1 PE=1 SV=3	0.00387	2.278684119	2.797202797	1	1	1	1	286	32.215	4.49	0	1
176	Q9Y266	Nuclear migration protein nudC OS=Homo sapiens OX=9606 GN=NUDC PE=1 SV=1	0.00387	2.233512794	3.32326284	1	1	1	1	331	38.219	5.38	1.82332	1
177	P25398	40S ribosomal protein S12 OS=Homo sapiens OX=9606 GN=RPS12 PE=1 SV=3	0.00387	2.078938109	7.575757576	1	1	1	1	132	14.505	7.21	2.12844	1
178	P24534	Elongation factor 1-beta OS=Homo sapiens OX=9606 GN=EEF1B2 PE=1 SV=3	0.00387	2.055616042	4	1	1	1	1	225	24.748	4.67	0	1
179	O00231	26S proteasome non-ATPase regulatory subunit 11 OS=Homo sapiens OX=9606 GN=PSMD11 PE=1 SV=3	0.00387	2.03263926	1.658767773	1	1	1	1	422	47.434	6.48	1.6161	1
180	Q92769	Histone deacetylase 2 OS=Homo sapiens OX=9606 GN=HDAC2 PE=1 SV=2	0.00387	2.028075051	2.459016393	1	1	1	1	488	55.329	5.91	0	1
181	P28072	Proteasome subunit beta type-6 OS=Homo sapiens OX=9606 GN=PSMB6 PE=1 SV=4	0.00387	2.003050752	4.184100418	1	1	1	1	239	25.341	4.92	2.34294	1
182	P49720	Proteasome subunit beta type-3 OS=Homo sapiens OX=9606 GN=PSMB3 PE=1 SV=2	0.00474	1.960585881	3.414634146	1	1	1	1	205	22.933	6.55	1.74416	1
183	P57088	Transmembrane protein 33 OS=Homo sapiens OX=9606 GN=TMEM33 PE=1 SV=2	0.00474	1.952725133	3.643724696	1	1	1	1	247	27.96	9.7	2.05533	1
184	Q15019	Septin-2 OS=Homo sapiens OX=9606 GN=SEPTIN2 PE=1 SV=1	0.00474	1.915572444	5.540166205	2	2	2	1	361	41.461	6.6	0	2
185	Q9H4M9	EH domain-containing protein 1 OS=Homo sapiens OX=9606 GN=EHD1 PE=1 SV=2	0.00474	1.914352712	1.685393258	1	1	1	1	534	60.589	6.83	2.07053	1
186	P46782	40S ribosomal protein S5 OS=Homo sapiens OX=9606 GN=RPS5 PE=1 SV=4	0.00474	1.904830649	4.901960784	1	1	1	1	204	22.862	9.72	2.71465	1
187	Q8WUH(	Transmembrane protein 263 OS=Homo sapiens OX=9606 GN=TMEM263 PE=1 SV=1	0.00474	1.90274269	8.620689655	1	1	1	1	116	11.741	9.32	1.78312	1
188	O00487	26S proteasome non-ATPase regulatory subunit 14 OS=Homo sapiens OX=9606 GN=PSMD14 PE=1 SV=1	0.00474	1.859806321	2.258064516	1	1	1	1	310	34.555	6.52	0	1
189	P62910	60S ribosomal protein L32 OS=Homo sapiens OX=9606 GN=RPL32 PE=1 SV=2	0.00474	1.853871964	5.925925926	1	1	1	1	135	15.85	11.33	1.69686	1
190	O75475	PC4 and SFRS1-interacting protein OS=Homo sapiens OX=9606 GN=PSIP1 PE=1 SV=1	0.0073	1.756465898	2.075471698	1	1	1	1	530	60.067	9.13	0	1
191	P23588	Eukaryotic translation initiation factor 4B OS=Homo sapiens OX=9606 GN=EIF4B PE=1 SV=2	0.0073	1.741123371	2.127659574	1	1	1	1	611	69.11	5.73	2.25206	1
192	Q08257	Quinone oxidoreductase OS=Homo sapiens OX=9606 GN=CRYZ PE=1 SV=1	0.0073	1.652279783	3.343465046	1	1	1	1	329	35.185	8.44	1.84381	1
193	P34897	Serine hydroxymethyltransferase, mitochondrial OS=Homo sapiens OX=9606 GN=SHMT2 PE=1 SV=3	0.0073	1.629116983	1.587301587	1	1	1	1	504	55.958	8.53	1.63379	1
194	O60841	Eukaryotic translation initiation factor 5B OS=Homo sapiens OX=9606 GN=EIF5B PE=1 SV=4	0.0073	1.623240605	0.819672131	1	1	1	1	1220	138.742	5.49	0	1
195	Q06323	Proteasome activator complex subunit 1 OS=Homo sapiens OX=9606 GN=PSME1 PE=1 SV=1	0.0073	1.570893992	2.81124498	1	1	1	1	249	28.705	6.02	0	1
196	P62995	Transformer-2 protein homolog beta OS=Homo sapiens OX=9606 GN=TRA2B PE=1 SV=1	0.0073	1.504594437	2.430555556	1	1	1	1	288	33.646	11.25	1.7463	1
197	P25789	Proteasome subunit alpha type-4 OS=Homo sapiens OX=9606 GN=PSMA4 PE=1 SV=1	0.0073	1.423428316	3.448275862	1	1	1	1	261	29.465	7.72	1.99006	1
198	Q13868	Exosome complex component RRP4 OS=Homo sapiens OX=9606 GN=EXOSC2 PE=1 SV=2	0.00907	1.361111575	3.754266212	1	1	1	1	293	32.768	7.5	2.2131	1
199	Q13428	Treacle protein OS=Homo sapiens OX=9606 GN=TCOF1 PE=1 SV=3	0.00907	1.32670256	0.60483871	1	1	1	1	1488	152.015	9.04	1.7287	1
200	Q15366	Poly(rC)-binding protein 2 OS=Homo sapiens OX=9606 GN=PCBP2 PE=1 SV=1	0.00907	1.31354249	3.561643836	1	1	1	1	365	38.556	6.79	1.85322	1
201	P62273	40S ribosomal protein S29 OS=Homo sapiens OX=9606 GN=RPS29 PE=1 SV=2	0.01693	1.172953983	14.28571429	1	1	1	1	56	6.672	10.13	1.67537	1
202	P28070	Proteasome subunit beta type-4 OS=Homo sapiens OX=9606 GN=PSMB4 PE=1 SV=4	0.0248	1.081812339	5.681818182	1	1	1	1	264	29.185	5.97	1.73977	1
203	O43396	Thioredoxin-like protein 1 OS=Homo sapiens OX=9606 GN=TXNL1 PE=1 SV=3	0.02564	1.077067043	3.806228374	1	1	1	1	289	32.231	4.96	0	1
204	Q9BQ52	Zinc phosphodiesterase ELAC protein 2 OS=Homo sapiens OX=9606 GN=ELAC2 PE=1 SV=2	0.03086	1.048128443	1.331719128	1	1	1	1	826	92.16	7.9	1.61717	1
205	Q53GQ0	Very-long-chain 3-oxoacyl-CoA reductase OS=Homo sapiens OX=9606 GN=HSD17B12 PE=1 SV=2	0.04891	0.900664722	3.846153846	1	1	1	1	312	34.302	9.32	1.62007	1
206	O43772	Mitochondrial carnitine/acylcarnitine carrier protein OS=Homo sapiens OX=9606 GN=SLC25A20 PE=1 SV=1	0.04944	0.884722409	2.657807309	1	1	1	1	301	32.922	9.41	0	1
207	O14639	Actin-binding LIM protein 1 OS=Homo sapiens OX=9606 GN=ABLIM1 PE=1 SV=3	0.04944	0.869666232	2.570694087	1	1	1	1	778	87.631	8.59	0	1
208	Q15459	Splicing factor 3A subunit 1 OS=Homo sapiens OX=9606 GN=SF3A1 PE=1 SV=1	0.04944	0.863913903	1.134930643	1	1	1	1	793	88.831	5.22	0	1
209	P09543	2',3'-cyclic-nucleotide 3'-phosphodiesterase OS=Homo sapiens OX=9606 GN=CNP PE=1 SV=2	0.04944	0.853871964	2.137767221	1	1	1	1	421	47.549	9.07	0	1
210	P42356	Phosphatidylinositol 4-kinase alpha OS=Homo sapiens OX=9606 GN=PI4KA PE=1 SV=4	0.04944	0.834755674	0.475737393	1	1	1	1	2102	236.678	7.06	0	1
211	Q68DK2	Zinc finger FYVE domain-containing protein 26 OS=Homo sapiens OX=9606 GN=ZFYVE26 PE=1 SV=3	0.05181	0.782516056	0.315084679	1	1	1	1	2539	284.395	6.39	0	1
212	P30043	Flavin reductase (NADPH) OS=Homo sapiens OX=9606 GN=BLVRB PE=1 SV=3	0.05181	0.782516056	4.368932039	1	1	1	1	206	22.105	7.65	0	1
213	O60658	High affinity cAMP-specific and IBMX-insensitive 3',5'-cyclic phosphodiesterase 8A OS=Homo sapiens OX=9606 (	0.05331	0.747632486	2.774427021	1	1	1	1	829	93.245	6.11	0	1
214	Q27J81	Inverted formin-2 OS=Homo sapiens OX=9606 GN=INF2 PE=1 SV=2	0.06014	0.695940534	0.880704564	1	1	1	1	1249	135.54	5.38	1.62824	1
215	Q9Y5A9	YTH domain-containing family protein 2 OS=Homo sapiens OX=9606 GN=YTHDF2 PE=1 SV=2	0.06911	0.566230166	2.07253886	1	1	1	1	579	62.296	8.79	0	1
216	Q16650	T-box brain protein 1 OS=Homo sapiens OX=9606 GN=TBR1 PE=1 SV=1	0.0724	0.547140664	1.759530792	1	1	1	1	682	74.007	7.33	1.79248	1
217	P17019	Zinc finger protein 708 OS=Homo sapiens OX=9606 GN=ZNF708 PE=1 SV=5	0.08333	0.508638306	1.803607214	1	1	1	1	499	57.32	9.39	0	1

1	**All interacting proteins of EIF1AX-NLS-N11A+D142A identified by Co-IP/MS**														
2	**Accession**	**Description**	**Exp. q-val**	**Sum PEP !**	**Coverage**	**# Peptides**	**# PSMs**	**# Unique I**	**# Protein**	**# AAs**	**MW [kDa**	**calc. pI**	**Score Seq**	**# Peptides**	**Sequest HT**
3	O15231	Zinc finger protein 185 OS=Homo sapiens OX=9606 GN=ZNF185 PE=1 SV=3	0	346.552	78	39	339	39	1	689	73.48	7.01	585.89	39	
4	P62277	40S ribosomal protein S13 OS=Homo sapiens OX=9606 GN=RPS13 PE=1 SV=2	0	200.537	22	45	53	45	1	151	17.212	10.54	83.17	45	
5	P56537	Eukaryotic translation initiation factor 6 OS=Homo sapiens OX=9606 GN=EIF6 PE=1 SV=1	0	135.723	19	38	46	38	1	245	26.582	4.68	48.95	38	
6	P08865	40S ribosomal protein SA OS=Homo sapiens OX=9606 GN=RPSA PE=1 SV=4	0	127.885	48	21	48	17	1	295	32.833	4.87	79.82	21	
7	Q8TDD1	ATP-dependent RNA helicase DDX54 OS=Homo sapiens OX=9606 GN=DDX54 PE=1 SV=2	0	111.992	15	12	21	12	1	881	98.534	10.02	56.5	12	
8	Q16625	Occludin OS=Homo sapiens OX=9606 GN=OCLN PE=1 SV=1	0	103.526	16	31	39	31	1	522	59.106	6.09	34.05	31	
9	P05387	60S acidic ribosomal protein P2 OS=Homo sapiens OX=9606 GN=RPLP2 PE=1 SV=1	0	98.209	24	26	38	26	1	115	11.658	4.54	44.37	26	
10	Q13200	26S proteasome non-ATPase regulatory subunit 2 OS=Homo sapiens OX=9606 GN=PSMD2 PE=1 SV=3	0	95.686	29	22	33	16	1	908	100.136	5.2	41.18	22	
11	P62891	60S ribosomal protein L39 OS=Homo sapiens OX=9606 GN=RPL39 PE=1 SV=2	0	85.633	11	22	28	22	1	51	6.403	12.56	35.9	22	
12	Q9H4M9	EH domain-containing protein 1 OS=Homo sapiens OX=9606 GN=EHD1 PE=1 SV=2	0	84.959	17	19	23	19	1	534	60.589	6.83	29.08	19	
13	P55036	26S proteasome non-ATPase regulatory subunit 4 OS=Homo sapiens OX=9606 GN=PSMD4 PE=1 SV=1	0	81.303	25	20	29	20	1	377	40.711	4.79	35.49	20	
14	Q04637	Eukaryotic translation initiation factor 4 gamma 1 OS=Homo sapiens OX=9606 GN=EIF4G1 PE=1 SV=4	0	77.357	22	17	20	9	1	1599	175.382	5.33	38.89	17	
15	P23284	Peptidyl-prolyl cis-trans isomerase B OS=Homo sapiens OX=9606 GN=PPIB PE=1 SV=2	0	67.545	25	17	21	17	1	216	23.728	9.41	18.75	17	
16	Q9Y4I1	Unconventional myosin-Va OS=Homo sapiens OX=9606 GN=MYO5A PE=1 SV=2	0	66.072	26	14	23	14	1	1855	215.269	8.48	28.51	14	
17	P11940	Polyadenylate-binding protein 1 OS=Homo sapiens OX=9606 GN=PABPC1 PE=1 SV=2	0	56.705	24	10	16	1	1	636	70.626	9.5	42.23	10	
18	Q8NFJ5	Retinoic acid-induced protein 3 OS=Homo sapiens OX=9606 GN=GPRC5A PE=1 SV=2	0	54.775	17	15	20	13	1	357	40.225	8.15	24.26	15	
19	P49736	DNA replication licensing factor MCM2 OS=Homo sapiens OX=9606 GN=MCM2 PE=1 SV=4	0	54.733	19	15	18	15	1	904	101.832	5.52	20.25	15	
20	P63104	14-3-3 protein zeta/delta OS=Homo sapiens OX=9606 GN=YWHAZ PE=1 SV=1	0	50.765	32	12	16	8	1	245	27.728	4.79	20.32	12	
21	P62241	40S ribosomal protein S8 OS=Homo sapiens OX=9606 GN=RPS8 PE=1 SV=2	0	45.917	18	14	17	13	1	208	24.19	10.32	17.46	14	
22	P17980	26S proteasome regulatory subunit 6A OS=Homo sapiens OX=9606 GN=PSMC3 PE=1 SV=3	0	45.257	24	10	18	1	1	439	49.172	5.24	23.6	10	
23	Q8IWV8	E3 ubiquitin-protein ligase UBR2 OS=Homo sapiens OX=9606 GN=UBR2 PE=1 SV=1	0	44.92	13	9	11	9	1	1755	200.408	6.24	16.51	9	
24	Q16650	T-box brain protein 1 OS=Homo sapiens OX=9606 GN=TBR1 PE=1 SV=1	0	44.544	9	14	15	14	1	682	74.007	7.33	13.4	14	
25	Q99729	Heterogeneous nuclear ribonucleoprotein A/B OS=Homo sapiens OX=9606 GN=HNRNPAB PE=1 SV=2	0	43.95	18	10	20	7	1	332	36.202	8.21	31.56	10	
26	P60228	Eukaryotic translation initiation factor 3 subunit E OS=Homo sapiens OX=9606 GN=EIF3E PE=1 SV=1	0	42.91	55	11	19	10	1	445	52.187	6.04	28.43	11	
27	Q92769	Histone deacetylase 2 OS=Homo sapiens OX=9606 GN=HDAC2 PE=1 SV=2	0	40.463	14	8	10	8	1	488	55.329	5.91	9.89	8	
28	Q9Y3U8	60S ribosomal protein L36 OS=Homo sapiens OX=9606 GN=RPL36 PE=1 SV=3	0	37.794	39	12	18	1	1	105	12.246	11.59	21.98	12	
29	Q8IWV7	E3 ubiquitin-protein ligase UBR1 OS=Homo sapiens OX=9606 GN=UBR1 PE=1 SV=1	0	33.638	5	10	10	6	1	1749	200.08	6.01	8.4	10	
30	P32119	Peroxiredoxin-2 OS=Homo sapiens OX=9606 GN=PRDX2 PE=1 SV=5	0	32.673	27	6	8	6	1	198	21.878	5.97	19.09	6	
31	P47813	Eukaryotic translation initiation factor 1A, X-chromosomal OS=Homo sapiens OX=9606 GN=EIF1AX PE=1 SV	0	30.069	7	6	10	6	1	144	16.432	5.24	10.99	6	
32	O14980	Exportin-1 OS=Homo sapiens OX=9606 GN=XPO1 PE=1 SV=1	0	29.009	64	8	8	8	1	1071	123.306	6.06	9.45	8	
33	P23588	Eukaryotic translation initiation factor 4B OS=Homo sapiens OX=9606 GN=EIF4B PE=1 SV=2	0	28.368	8	10	10	10	1	611	69.11	5.73	8.11	10	
34	Q15365	Poly(rC)-binding protein 1 OS=Homo sapiens OX=9606 GN=PCBP1 PE=1 SV=2	0	26.6	29	6	6	6	1	356	37.474	7.09	7.21	6	
35	P26373	60S ribosomal protein L13 OS=Homo sapiens OX=9606 GN=RPL13 PE=1 SV=4	0	25.817	15	6	8	6	1	211	24.247	11.65	10.8	6	
36	P61313	60S ribosomal protein L15 OS=Homo sapiens OX=9606 GN=RPL15 PE=1 SV=2	0	25.705	27	6	7	6	1	204	24.131	11.62	8.33	6	
37	P55884	Eukaryotic translation initiation factor 3 subunit B OS=Homo sapiens OX=9606 GN=EIF3B PE=1 SV=3	0	25.694	24	6	12	6	1	814	92.424	5	8.51	6	
38	P62913	60S ribosomal protein L11 OS=Homo sapiens OX=9606 GN=RPL11 PE=1 SV=2	0	25.561	12	8	9	8	1	178	20.24	9.6	8.92	8	
39	Q15233	Non-POU domain-containing octamer-binding protein OS=Homo sapiens OX=9606 GN=NONO PE=1 SV=4	0	23.865	16	7	13	7	1	471	54.197	8.95	9.77	7	
40	P09327	Villin-1 OS=Homo sapiens OX=9606 GN=VIL1 PE=1 SV=4	0	21.819	8	8	11	8	1	827	92.637	6.39	7.62	8	
41	P61247	40S ribosomal protein S3a OS=Homo sapiens OX=9606 GN=RPS3A PE=1 SV=2	0	20.563	4	7	8	7	1	264	29.926	9.73	13.77	7	
42	P21589	5'-nucleotidase OS=Homo sapiens OX=9606 GN=NT5E PE=1 SV=1	0	20.139	17	8	11	6	1	574	63.327	7.03	7.17	8	
43	Q9NQX4	Unconventional myosin-Vc OS=Homo sapiens OX=9606 GN=MYO5C PE=1 SV=2	0	20.118	14	7	9	6	1	1742	202.682	7.71	4.31	7	
44	Q96A65	Exocyst complex component 4 OS=Homo sapiens OX=9606 GN=EXOC4 PE=1 SV=1	0	19.86	39	8	15	8	1	974	110.429	6.49	8.89	8	
45	P61513	60S ribosomal protein L37a OS=Homo sapiens OX=9606 GN=RPL37A PE=1 SV=2	0	19.369	12	4	5	4	1	92	10.268	10.43	4.14	4	
46	P61978	Heterogeneous nuclear ribonucleoprotein K OS=Homo sapiens OX=9606 GN=HNRNPK PE=1 SV=1	0	19.287	13	8	8	8	1	463	50.944	5.54	5.17	8	
47	P14866	Heterogeneous nuclear ribonucleoprotein L OS=Homo sapiens OX=9606 GN=HNRNPL PE=1 SV=2	0	19.164	3	7	9	2	1	589	64.092	8.22	9.07	7	
48	P62244	40S ribosomal protein S15a OS=Homo sapiens OX=9606 GN=RPS15A PE=1 SV=2	0	19.145	13	5	5	5	1	130	14.83	10.13	6.48	5	
49	P21796	Voltage-dependent anion-selective channel protein 1 OS=Homo sapiens OX=9606 GN=VDAC1 PE=1 SV=2	0	18.383	6	8	9	8	1	283	30.754	8.54	5.42	8	
50	P46779	60S ribosomal protein L28 OS=Homo sapiens OX=9606 GN=RPL28 PE=1 SV=3	0	18.335	24	4	9	4	1	137	15.738	12.02	12.66	4	
51	P42766	60S ribosomal protein L35 OS=Homo sapiens OX=9606 GN=RPL35 PE=1 SV=2	0	17.927	5	8	10	8	1	123	14.543	11.05	2.34	8	
52	Q13868	Exosome complex component RRP4 OS=Homo sapiens OX=9606 GN=EXOSC2 PE=1 SV=2	0	17.572	6	4	4	4	1	293	32.768	7.5	4.51	4	
53	O43818	U3 small nucleolar RNA-interacting protein 2 OS=Homo sapiens OX=9606 GN=RRP9 PE=1 SV=1	0	17.41	13	5	5	5	1	475	51.809	7.85	5.27	5	
54	P62851	40S ribosomal protein S25 OS=Homo sapiens OX=9606 GN=RPS25 PE=1 SV=1	0	17.372	15	7	8	7	1	125	13.734	10.11	9.27	7	
55	P62854	40S ribosomal protein S26 OS=Homo sapiens OX=9606 GN=RPS26 PE=1 SV=3	0	17.298	12	6	6	6	1	115	13.007	11	6.49	6	
56	P61254	60S ribosomal protein L26 OS=Homo sapiens OX=9606 GN=RPL26 PE=1 SV=1	0	17.156	6	5	5	5	1	145	17.248	10.55	5.66	5	
57	P62917	60S ribosomal protein L8 OS=Homo sapiens OX=9606 GN=RPL8 PE=1 SV=2	0	16.604	10	6	6	6	1	257	28.007	11.03	2.01	6	
58	P62333	26S proteasome regulatory subunit 10B OS=Homo sapiens OX=9606 GN=PSMC6 PE=1 SV=1	0	16.494	6	7	9	7	1	389	44.145	7.49	7.33	7	
59	P05388	60S acidic ribosomal protein P0 OS=Homo sapiens OX=9606 GN=RPLP0 PE=1 SV=1	0	16.398	16	5	7	4	1	317	34.252	5.97	5.45	5	
60	P50914	60S ribosomal protein L14 OS=Homo sapiens OX=9606 GN=RPL14 PE=1 SV=4	0	15.264	6	7	7	7	1	215	23.417	10.93	0	7	
61	Q06830	Peroxiredoxin-1 OS=Homo sapiens OX=9606 GN=PRDX1 PE=1 SV=1	0	15.162	9	4	7	4	1	199	22.096	8.13	7.06	4	
62	Q15717	ELAV-like protein 1 OS=Homo sapiens OX=9606 GN=ELAVL1 PE=1 SV=2	0	14.755	3	6	6	6	1	326	36.069	9.17	10.47	6	
63	P07900	Heat shock protein HSP 90-alpha OS=Homo sapiens OX=9606 GN=HSP90AA1 PE=1 SV=5	0	14.722	8	5	5	5	1	732	84.607	5.02	3.78	5	
64	P84098	60S ribosomal protein L19 OS=Homo sapiens OX=9606 GN=RPL19 PE=1 SV=1	0	14.421	21	4	6	1	1	196	23.451	11.47	10.14	4	
65	P62081	40S ribosomal protein S7 OS=Homo sapiens OX=9606 GN=RPS7 PE=1 SV=1	0	13.158	11	3	4	3	1	194	22.113	10.1	4.6	3	
66	P46777	60S ribosomal protein L5 OS=Homo sapiens OX=9606 GN=RPL5 PE=1 SV=3	0	13.06	7	4	6	4	1	297	34.341	9.72	10.78	4	
67	Q68DK2	Zinc finger FYVE domain-containing protein 26 OS=Homo sapiens OX=9606 GN=ZFYVE26 PE=1 SV=3	0	12.233	18	4	4	4	1	2539	284.395	6.39	0	4	
68	O75475	PC4 and SFRS1-interacting protein OS=Homo sapiens OX=9606 GN=PSIP1 PE=1 SV=1	0	11.981	6	5	5	5	1	530	60.067	9.13	3.88	5	
69	P62753	40S ribosomal protein S6 OS=Homo sapiens OX=9606 GN=RPS6 PE=1 SV=1	0	11.875	7	5	6	5	1	249	28.663	10.84	9.44	5	
70	P60866	40S ribosomal protein S20 OS=Homo sapiens OX=9606 GN=RPS20 PE=1 SV=1	0	11.822	4	5	5	5	1	119	13.364	9.94	3.65	5	
71	P23396	40S ribosomal protein S3 OS=Homo sapiens OX=9606 GN=RPS3 PE=1 SV=2	0	11.605	5	2	4	2	1	243	26.671	9.66	7.92	2	
72	P42765	3-ketoacyl-CoA thiolase, mitochondrial OS=Homo sapiens OX=9606 GN=ACAA2 PE=1 SV=2	0	11.378	15	5	7	4	1	397	41.898	8.09	4.01	5	
73	P62195	26S proteasome regulatory subunit 8 OS=Homo sapiens OX=9606 GN=PSMC5 PE=1 SV=1	0	11.373	8	4	4	4	1	406	45.597	7.55	5.69	4	

74	Q04917	14-3-3 protein eta OS=Homo sapiens OX=9606 GN=YWHAH PE=1 SV=4	0	11.296	5	3	3	3	1	246	28.201	4.84	4.69	3
75	Q12931	Heat shock protein 75 kDa, mitochondrial OS=Homo sapiens OX=9606 GN=TRAP1 PE=1 SV=3	0	11.218	12	3	3	3	1	704	80.06	8.21	2.74	3
76	Q13151	Heterogeneous nuclear ribonucleoprotein A0 OS=Homo sapiens OX=9606 GN=HNRNPA0 PE=1 SV=1	0	10.993	16	3	3	3	1	305	30.822	9.29	2.27	3
77	P24534	Elongation factor 1-beta OS=Homo sapiens OX=9606 GN=EEF1B2 PE=1 SV=3	0	10.831	12	5	5	5	1	225	24.748	4.67	4.48	5
78	P02545	Prelamin-A/C OS=Homo sapiens OX=9606 GN=LMNA PE=1 SV=1	0	10.723	27	3	3	3	1	664	74.095	7.02	4.04	3
79	P27449	V-type proton ATPase 16 kDa proteolipid subunit OS=Homo sapiens OX=9606 GN=ATP6V0C PE=1 SV=1	0	10.694	5	3	4	3	1	155	15.725	8.44	7.22	3
80	P18124	60S ribosomal protein L7 OS=Homo sapiens OX=9606 GN=RPL7 PE=1 SV=1	0	10.525	18	3	5	3	1	248	29.207	10.65	6.69	3
81	P62979	Ubiquitin-40S ribosomal protein S27a OS=Homo sapiens OX=9606 GN=RPS27A PE=1 SV=2	0	10.514	3	5	5	5	1	156	17.953	9.64	4.28	5
82	P15313	V-type proton ATPase subunit B, kidney isoform OS=Homo sapiens OX=9606 GN=ATP6V1B1 PE=1 SV=3	0	9.463	5	4	4	4	1	513	56.797	5.66	1.76	4
83	P31942	Heterogeneous nuclear ribonucleoprotein H3 OS=Homo sapiens OX=9606 GN=HNRNPH3 PE=1 SV=2	0	9.44	4	2	2	2	1	346	36.903	6.87	3.45	2
84	P62280	40S ribosomal protein S11 OS=Homo sapiens OX=9606 GN=RPS11 PE=1 SV=3	0	9.303	5	4	4	4	1	158	18.419	10.3	3.62	4
85	P62701	40S ribosomal protein S4, X isoform OS=Homo sapiens OX=9606 GN=RPS4X PE=1 SV=2	0	9.245	3	4	4	4	1	263	29.579	10.15	6.62	4
86	P46778	60S ribosomal protein L21 OS=Homo sapiens OX=9606 GN=RPL21 PE=1 SV=2	0	8.82	7	3	3	3	1	160	18.553	10.49	4.64	3
87	Q13310	Polyadenylate-binding protein 4 OS=Homo sapiens OX=9606 GN=PABPC4 PE=1 SV=1	0	8.652	23	3	5	1	1	644	70.738	9.26	5.97	3
88	P15880	40S ribosomal protein S2 OS=Homo sapiens OX=9606 GN=RPS2 PE=1 SV=2	0	8.33	6	4	4	4	1	293	31.305	10.24	1.97	4
89	P27348	14-3-3 protein theta OS=Homo sapiens OX=9606 GN=YWHAQ PE=1 SV=1	0	8.324	14	3	3	3	1	245	27.747	4.78	3.82	3
90	Q9Y5A9	YTH domain-containing family protein 2 OS=Homo sapiens OX=9606 GN=YTHDF2 PE=1 SV=2	0	8.087	16	3	4	3	1	579	62.296	8.79	4.19	3
91	P0CG38	POTE ankyrin domain family member I OS=Homo sapiens OX=9606 GN=POTEI PE=3 SV=1	0	8.04	21	4	6	1	1	1075	121.205	6.21	5.97	4
92	Q9H6R4	Nucleolar protein 6 OS=Homo sapiens OX=9606 GN=NOL6 PE=1 SV=2	0	7.998	8	2	2	2	1	1146	127.513	7.64	2.39	2
93	P55209	Nucleosome assembly protein 1-like 1 OS=Homo sapiens OX=9606 GN=NAP1L1 PE=1 SV=1	0	7.894	17	2	3	2	1	391	45.346	4.46	2.61	2
94	P62847	40S ribosomal protein S24 OS=Homo sapiens OX=9606 GN=RPS24 PE=1 SV=1	0	7.512	1	3	3	3	1	133	15.413	10.78	0	3
95	Q9NZ01	Very-long-chain enoyl-CoA reductase OS=Homo sapiens OX=9606 GN=TECR PE=1 SV=1	0	7.427	12	3	3	3	1	308	36.011	9.45	0	3
96	Q63HN8	E3 ubiquitin-protein ligase RNF213 OS=Homo sapiens OX=9606 GN=RNF213 PE=1 SV=3	0	7.074	7	3	3	3	1	5207	591.03	6.48	3.63	3
97	P62937	Peptidyl-prolyl cis-trans isomerase A OS=Homo sapiens OX=9606 GN=PPIA PE=1 SV=2	0	6.893	3	2	2	2	1	165	18.001	7.81	1.61	2
98	P62258	14-3-3 protein epsilon OS=Homo sapiens OX=9606 GN=YWHAE PE=1 SV=1	0	6.793	3	3	3	3	1	255	29.155	4.74	0	3
99	P11021	Endoplasmic reticulum chaperone BiP OS=Homo sapiens OX=9606 GN=HSPA5 PE=1 SV=2	0	6.371	4	3	5	3	1	654	72.288	5.16	0	3
100	P26599	Polypyrimidine tract-binding protein 1 OS=Homo sapiens OX=9606 GN=PTBP1 PE=1 SV=1	0	6.371	8	3	3	3	1	531	57.186	9.17	0	3
101	P30050	60S ribosomal protein L12 OS=Homo sapiens OX=9606 GN=RPL12 PE=1 SV=1	0	6.289	6	2	2	2	1	165	17.808	9.42	4.51	2
102	Q9Y266	Nuclear migration protein nudC OS=Homo sapiens OX=9606 GN=NUDC PE=1 SV=1	0	6.063	5	1	1	1	1	331	38.219	5.38	0	1
103	O00159	Unconventional myosin-Ic OS=Homo sapiens OX=9606 GN=MYO1C PE=1 SV=4	0	6.057	6	2	2	2	1	1063	121.606	9.41	0	2
104	O43242	26S proteasome non-ATPase regulatory subunit 3 OS=Homo sapiens OX=9606 GN=PSMD3 PE=1 SV=2	0	5.754	6	2	2	2	1	534	60.939	8.44	1.87	2
105	P51571	Translocon-associated protein subunit delta OS=Homo sapiens OX=9606 GN=SSR4 PE=1 SV=1	0	5.689	4	4	4	3	1	173	18.987	6.15	3.4	4
106	P08238	Heat shock protein HSP 90-beta OS=Homo sapiens OX=9606 GN=HSP90AB1 PE=1 SV=4	0	5.668	5	2	2	2	1	724	83.212	5.03	2.47	2
107	P18206	Vinculin OS=Homo sapiens OX=9606 GN=VCL PE=1 SV=4	0	5.654	3	2	3	2	1	1134	123.722	5.66	1.73	2
108	P41091	Eukaryotic translation initiation factor 2 subunit 3 OS=Homo sapiens OX=9606 GN=EIF2S3 PE=1 SV=3	0	5.649	2	2	2	2	1	472	51.077	8.4	1.74	2
109	O43795	Unconventional myosin-Ib OS=Homo sapiens OX=9606 GN=MYO1B PE=1 SV=3	0	5.57	4	1	1	1	1	1136	131.902	9.38	0	1
110	P27635	60S ribosomal protein L10 OS=Homo sapiens OX=9606 GN=RPL10 PE=1 SV=4	0	5.553	3	1	1	1	1	214	24.588	10.08	2.67	1
111	P0DMV9	Heat shock 70 kDa protein 1B OS=Homo sapiens OX=9606 GN=HSPA1B PE=1 SV=1	0	5.379	2	2	2	2	1	641	70.009	5.66	1.7	2
112	P22314	Ubiquitin-like modifier-activating enzyme 1 OS=Homo sapiens OX=9606 GN=UBA1 PE=1 SV=3	0	5.33	4	3	3	3	1	1058	117.774	5.76	5.47	3
113	P25311	Zinc-alpha-2-glycoprotein OS=Homo sapiens OX=9606 GN=AZGP1 PE=1 SV=2	0	5.163	13	1	1	1	1	298	34.237	6.05	2.7	1
114	P62829	60S ribosomal protein L23 OS=Homo sapiens OX=9606 GN=RPL23 PE=1 SV=1	0	5.108	4	1	2	1	1	140	14.856	10.51	2.09	1
115	P62906	60S ribosomal protein L10a OS=Homo sapiens OX=9606 GN=RPL10A PE=1 SV=2	0	5.021	5	2	3	2	1	217	24.816	9.94	2.8	2
116	Q07020	60S ribosomal protein L18 OS=Homo sapiens OX=9606 GN=RPL18 PE=1 SV=2	0	5.004	8	3	3	3	1	188	21.621	11.72	0	3
117	Q5RKV6	Exosome complex component MTR3 OS=Homo sapiens OX=9606 GN=EXOSC6 PE=1 SV=1	0	4.896	3	1	1	1	1	272	28.218	6.28	1.67	1
118	P60842	Eukaryotic initiation factor 4A-I OS=Homo sapiens OX=9606 GN=EIF4A1 PE=1 SV=1	0	4.679	7	3	3	1	1	406	46.125	5.48	1.76	3
119	P10809	60 kDa heat shock protein, mitochondrial OS=Homo sapiens OX=9606 GN=HSPD1 PE=1 SV=2	0	4.537	2	2	2	2	1	573	61.016	5.87	0	2
120	P46776	60S ribosomal protein L27a OS=Homo sapiens OX=9606 GN=RPL27A PE=1 SV=2	0	4.407	5	1	1	1	1	148	16.551	11	2.28	1
121	Q9NVP1	ATP-dependent RNA helicase DDX18 OS=Homo sapiens OX=9606 GN=DDX18 PE=1 SV=2	0	4.314	2	1	1	1	1	670	75.359	9.5	2.12	1
122	P62899	60S ribosomal protein L31 OS=Homo sapiens OX=9606 GN=RPL31 PE=1 SV=1	0	4.055	6	2	2	2	1	125	14.454	10.54	0	2
123	P42677	40S ribosomal protein S27 OS=Homo sapiens OX=9606 GN=RPS27 PE=1 SV=3	0	4.053	1	2	3	2	1	84	9.455	9.45	2.39	2
124	P18621	60S ribosomal protein L17 OS=Homo sapiens OX=9606 GN=RPL17 PE=1 SV=3	0	3.98	3	1	1	1	1	184	21.383	10.17	2.62	1
125	P61353	60S ribosomal protein L27 OS=Homo sapiens OX=9606 GN=RPL27 PE=1 SV=2	0	3.66	3	1	1	1	1	136	15.788	10.56	0	1
126	Q14103	Heterogeneous nuclear ribonucleoprotein D0 OS=Homo sapiens OX=9606 GN=HNRNPD PE=1 SV=1	0	3.55	2	2	2	2	1	355	38.41	7.81	1.92	2
127	P62424	60S ribosomal protein L7a OS=Homo sapiens OX=9606 GN=RPL7A PE=1 SV=2	0	3.427	5	1	1	1	1	266	29.977	10.61	2.04	1
128	Q9P035	Very-long-chain (3R)-3-hydroxyacyl-CoA dehydratase 3 OS=Homo sapiens OX=9606 GN=HACD3 PE=1 SV=	0	3.339	12	2	2	2	1	362	43.132	8.94	0	2
129	P62888	60S ribosomal protein L30 OS=Homo sapiens OX=9606 GN=RPL30 PE=1 SV=2	0	3.316	5	2	3	2	1	115	12.776	9.63	0	2
130	P62750	60S ribosomal protein L23a OS=Homo sapiens OX=9606 GN=RPL23A PE=1 SV=1	0	3.268	9	2	2	2	1	156	17.684	10.45	1.98	2
131	P51991	Heterogeneous nuclear ribonucleoprotein A3 OS=Homo sapiens OX=9606 GN=HNRNPA3 PE=1 SV=2	0	3.253	4	1	1	1	1	378	39.571	9.01	1.77	1
132	P62249	40S ribosomal protein S16 OS=Homo sapiens OX=9606 GN=RPS16 PE=1 SV=2	0	3.179	1	2	2	2	1	146	16.435	10.21	1.84	2
133	P11142	Heat shock cognate 71 kDa protein OS=Homo sapiens OX=9606 GN=HSPA8 PE=1 SV=1	0	3.121	2	1	1	1	1	646	70.854	5.52	1.67	1
134	P09651	Heterogeneous nuclear ribonucleoprotein A1 OS=Homo sapiens OX=9606 GN=HNRNPA1 PE=1 SV=5	0	3.096	2	1	1	1	1	372	38.723	9.13	0	1
135	Q15366	Poly(rC)-binding protein 2 OS=Homo sapiens OX=9606 GN=PCBP2 PE=1 SV=1	0	3.092	1	1	1	1	1	365	38.556	6.79	1.71	1
136	Q9BQ52	Zinc phosphodiesterase ELAC protein 2 OS=Homo sapiens OX=9606 GN=ELAC2 PE=1 SV=2	0	2.897	12	1	1	1	1	826	92.16	7.9	0	1
137	P40429	60S ribosomal protein L13a OS=Homo sapiens OX=9606 GN=RPL13A PE=1 SV=2	0	2.812	7	1	1	1	1	203	23.562	10.93	0	1
138	P45880	Voltage-dependent anion-selective channel protein 2 OS=Homo sapiens OX=9606 GN=VDAC2 PE=1 SV=2	0	2.761	12	1	1	1	1	294	31.547	7.56	0	1
139	O00303	Eukaryotic translation initiation factor 3 subunit F OS=Homo sapiens OX=9606 GN=EIF3F PE=1 SV=1	0	2.758	6	2	2	2	1	357	37.54	5.45	0	2
140	Q6P5R6	60S ribosomal protein L22-like 1 OS=Homo sapiens OX=9606 GN=RPL22L1 PE=1 SV=2	0	2.678	2	1	1	1	1	122	14.598	9.38	0	1
141	Q9P0L0	Vesicle-associated membrane protein-associated protein A OS=Homo sapiens OX=9606 GN=VAPA PE=1 SV=3	0	2.65	1	1	1	1	1	249	27.875	8.62	2.32	1
142	Q9ULV0	Unconventional myosin-Vb OS=Homo sapiens OX=9606 GN=MYO5B PE=1 SV=3	0	2.618	5	1	1	1	1	1848	213.538	7.2	0	1
143	P62857	40S ribosomal protein S28 OS=Homo sapiens OX=9606 GN=RPS28 PE=1 SV=1	0	2.558	2	1	1	1	1	69	7.836	10.7	1.62	1
144	P62266	40S ribosomal protein S23 OS=Homo sapiens OX=9606 GN=RPS23 PE=1 SV=3	0	2.474	1	1	1	1	1	143	15.798	10.49	1.75	1
145	P46781	40S ribosomal protein S9 OS=Homo sapiens OX=9606 GN=RPS9 PE=1 SV=3	0	2.438	2	1	1	1	1	194	22.578	10.65	0	1
146	P62841	40S ribosomal protein S15 OS=Homo sapiens OX=9606 GN=RPS15 PE=1 SV=2	0	2.435	1	1	1	1	1	145	17.029	10.39	0	1
147	P38606	V-type proton ATPase catalytic subunit A OS=Homo sapiens OX=9606 GN=ATP6V1A PE=1 SV=2	0	2.398	5	1	1	1	1	617	68.26	5.52	0	1
148	O94832	Unconventional myosin-Id OS=Homo sapiens OX=9606 GN=MYO1D PE=1 SV=2	0	2.344	1	1	1	1	1	1006	116.129	9.39	0	1
149	Q92614	Unconventional myosin-XVIIIa OS=Homo sapiens OX=9606 GN=MYO18A PE=1 SV=3	0	2.339	1	1	1	1	1	2054	232.971	6.3	1.88	1
150	P46783	40S ribosomal protein S10 OS=Homo sapiens OX=9606 GN=RPS10 PE=1 SV=1	0	2.227	2	1	2	1	1	165	18.886	10.15	0	1
151	P62263	40S ribosomal protein S14 OS=Homo sapiens OX=9606 GN=RPS14 PE=1 SV=3	0	2.184	2	1	1	1	1	151	16.263	10.05	0	1
152	P22392	Nucleoside diphosphate kinase B OS=Homo sapiens OX=9606 GN=NME2 PE=1 SV=1	0	2.151	2	1	1	1	1	152	17.287	8.41	1.71	1
153	P17019	Zinc finger protein 708 OS=Homo sapiens OX=9606 GN=ZNF708 PE=1 SV=5	0	2.029	2	1	2	1	1	499	57.32	9.39	0	1
154	P43686	26S proteasome regulatory subunit 6B OS=Homo sapiens OX=9606 GN=PSMC4 PE=1 SV=2	0.001	1.891	2	1	1	1	1	418	47.337	5.21	2.04	1
155	Q7L2E3	ATP-dependent RNA helicase DHX30 OS=Homo sapiens OX=9606 GN=DHX30 PE=1 SV=1	0.001	1.837	3	1	1	1	1	1194	133.855	8.78	0	1
156	Q02878	60S ribosomal protein L6 OS=Homo sapiens OX=9606 GN=RPL6 PE=1 SV=3	0.001	1.794	1	1	3	1	1	288	32.708	10.58	0	1
157	P39023	60S ribosomal protein L3 OS=Homo sapiens OX=9606 GN=RPL3 PE=1 SV=2	0.001	1.786	3	1	1	1	1	403	46.08	10.18	0	1
158	P47914	60S ribosomal protein L29 OS=Homo sapiens OX=9606 GN=RPL29 PE=1 SV=2	0.001	1.765	3	1	1	1	1	159	17.741	11.66	0	1
159	Q9NQC3	Reticulon-4 OS=Homo sapiens OX=9606 GN=RTN4 PE=1 SV=2	0.001	1.746	3	1	1	1	1	1192	129.851	4.5	0	1
160	P83731	60S ribosomal protein L24 OS=Homo sapiens OX=9606 GN=RPL24 PE=1 SV=1	0.002	1.67	1	1	1	1	1	157	17.768	11.25	1.93	1
161	P63173	60S ribosomal protein L38 OS=Homo sapiens OX=9606 GN=RPL38 PE=1 SV=2	0.002	1.65	4	1	1	1	1	70	8.213	10.1	0	1
162	P32969	60S ribosomal protein L9 OS=Homo sapiens OX=9606 GN=RPL9 PE=1 SV=1	0.002	1.647	1	1	1	1	1	192	21.85	9.95	0	1
163	Q9UM54	Unconventional myosin-VI OS=Homo sapiens OX=9606 GN=MYO6 PE=1 SV=4	0.002	1.627	0	1	1	1	1	1294	149.596	8.53	0	1
164	O00571	ATP-dependent RNA helicase DDX3X OS=Homo sapiens OX=9606 GN=DDX3X PE=1 SV=3	0.002	1.593	2	1	1	1	1	662	73.198	7.18	0	1
165	P31946	14-3-3 protein beta/alpha OS=Homo sapiens OX=9606 GN=YWHAB PE=1 SV=3	0.002	1.593	1	1	1	1	1	246	28.065	4.83	0	1
166	Q14254	Flotillin-2 OS=Homo sapiens OX=9606 GN=FLOT2 PE=1 SV=2	0.002	1.553	1	1	2	1	1	428	47.035	5.25	0	1
167	P52597	Heterogeneous nuclear ribonucleoprotein F OS=Homo sapiens OX=9606 GN=HNRNPF PE=1 SV=3	0.002	1.533	3	1	1	1	1	415	45.643	5.58	0	1
168	P30041	Peroxiredoxin-6 OS=Homo sapiens OX=9606 GN=PRDX6 PE=1 SV=3	0.003	1.482	4	1	1	1	1	224	25.019	6.38	0	1
169	Q14974	Importin subunit beta-1 OS=Homo sapiens OX=9606 GN=KPNB1 PE=1 SV=2	0.003	1.473	4	1	1	1	1	876	97.108	4.78	0	1
170	P34932	Heat shock 70 kDa protein 4 OS=Homo sapiens OX=9606 GN=HSPA4 PE=1 SV=4	0.004	1.416	2	1	1	1	1	840	94.271	5.19	0	1
171	P61981	14-3-3 protein gamma OS=Homo sapiens OX=9606 GN=YWHAG PE=1 SV=2	0.004	1.413	1	1	1	1	1	247	28.285	4.89	0	1
172	P05386	60S acidic ribosomal protein P1 OS=Homo sapiens OX=9606 GN=RPLP1 PE=1 SV=1	0.004	1.401	5	1	1	1	1	114	11.507	4.32	0	1
173	P15531	Nucleoside diphosphate kinase A OS=Homo sapiens OX=9606 GN=NME1 PE=1 SV=1	0.004	1.389	0	1	1	1	1	152	17.138	6.19	0	1
174	Q5SSJ5	Heterochromatin protein 1-binding protein 3 OS=Homo sapiens OX=9606 GN=HP1BP3 PE=1 SV=1	0.006	1.367	2	1	1	1	1	553	61.169	9.67	0	1
175	Q99460	26S proteasome non-ATPase regulatory subunit 1 OS=Homo sapiens OX=9606 GN=PSMD1 PE=1 SV=2	0.006	1.346	3	1	1	1	1	953	105.769	5.39	0	1
176	Q99497	Parkinson disease protein 7 OS=Homo sapiens OX=9606 GN=PARK7 PE=1 SV=2	0.008	1.328	0	1	1	1	1	189	19.878	6.79	0	1
177	P49321	Nuclear autoantigenic sperm protein OS=Homo sapiens OX=9606 GN=NASP PE=1 SV=2	0.008	1.32	1	1	1	1	1	788	85.186	4.3	0	1
178	P36578	60S ribosomal protein L4 OS=Homo sapiens OX=9606 GN=RPL4 PE=1 SV=5	0.009	1.288	5	1	1	1	1	427	47.667	11.06	0	1
179	Q9Y277	Voltage-dependent anion-selective channel protein 3 OS=Homo sapiens OX=9606 GN=VDAC3 PE=1 SV=1	0.011	1.249	3	1	1	1	1	283	30.639	8.66	0	1
180	Q53GQ0	Very-long-chain 3-oxoacyl-CoA reductase OS=Homo sapiens OX=9606 GN=HSD17B12 PE=1 SV=2	0.011	1.246	2	1	1	1	1	312	34.302	9.32	0	1
181	O60664	Perilipin-3 OS=Homo sapiens OX=9606 GN=PLIN3 PE=1 SV=3	0.011	1.214	0	1	1	1	1	434	47.046	5.44	0	1
182	O95373	Importin-7 OS=Homo sapiens OX=9606 GN=IPO7 PE=1 SV=1	0.014	1.161	1	1	1	1	1	1038	119.44	4.82	0	1
183	Q9HD45	Transmembrane 9 superfamily member 3 OS=Homo sapiens OX=9606 GN=TM9SF3 PE=1 SV=2	0.017	1.116	9	1	1	1	1	589	67.843	7.21	0	1
184	P49755	Transmembrane emp24 domain-containing protein 10 OS=Homo sapiens OX=9606 GN=TMED10 PE=1 SV=2	0.02	1.072	4	1	1	1	1	219	24.96	7.44	0	1
185	Q9HCE1	Helicase MOV-10 OS=Homo sapiens OX=9606 GN=MOV10 PE=1 SV=2	0.021	1.04	1	1	1	1	1	1003	113.599	8.82	0	1
186	P62269	40S ribosomal protein S18 OS=Homo sapiens OX=9606 GN=RPS18 PE=1 SV=3	0.021	1.023	1	1	1	1	1	152	17.708	10.99	0	1

Supplementary Table 2. The genes and sgRNAs information of CRISPR library.

A2M	TGAAATGAAACTTCACACTG
A2M	ACTGCATCTGTGCAAACGGG
A2M	ATGTCTCATGAACTACCCTG
A2M	TTACTCATATAGGATCCCAA
A2M	AAGCCTGTGAATCTTCACCG
AADAC	ATGTATATAATGCCTTAAGG
AADAC	ATTTCTATCCAAATCACTCA
AADAC	AAGTCTGAAGCACTAAGAAG
AADAC	GGTATTTCTGGAGATAGTGC

AADAC	TGAGGATCCCCACAATCAGA
AARS	TTGGTGCTGAGGATGCCGAT
AARS	TCAGAATGTGCGTAGCTGTG
AARS	GGAGGCCAGATCTATGACGA
AARS	CCAAGAAAAGCATTGACACA
AARS	GTAGGCCATGTCAATCCCAT
AATK	CTCCTCAAGTCCACAGACGT
AATK	AGCACCGGAACCACGCCCGG
AATK	GTGTCAGCCAACAACAACAG
AATK	GCATAGCAACCTGCTCGTCG
AATK	GCTGGCTGCAGCCCGAGCAG
ABAT	GTGAATGGCTTTAGAGTGCG
ABAT	GAAAGAAGTCATCGGATGCG
ABAT	TGCAATTACGAAGAGAGCCG
ABAT	CCTTAAAGACCATCTTCATG
ABAT	TGATGAAGACGGAAGTCCCA
ABCA1	GTGGCATGGCAGGACTACGT
ABCA1	AATGCAGAGAAAGCTATCTG
ABCA1	GGACACGCCCAGCTTCAAGT
ABCA1	TTGGACAGCCCAAGACATCG
ABCA1	TGTCAAGTACAAGATCCGAA
ABCA2	CATGTACGTGGCGATCCGAG
ABCA2	TAGGGGATGAAATTGCCACG
ABCA2	GATGTCGTGCCCGTAGATGG
ABCA2	CTTGTGTGGCAACAACCGGT
ABCA2	GGTCTGGCTCAACATCTCGG
ABCA3	ACCTACGGTTCAGTTACACA
ABCA3	CCTCCTGAGTCCCGTCAACG
ABCA3	AATGCGCATTTACTGACCGA
ABCA3	AGTGGACGGGCATACATCAG
ABCA3	AGAGTCATCCAGTTGTACCG
ABCA4	GATCCGAATGGACATAGACG
ABCA4	AGCACCATACCCGCAAACAG
ABCA4	AGGGTGTTGAGTTGCCACAG
ABCA4	AAAGACCGAGCCCCTAACAG
ABCA4	TTGTCCTTACAGCTTGGCAA
ABCA5	GAAATAGATACCTTTCCCCG
ABCA5	TGACAGTGACTATGTATCCG
ABCA5	AAAATGAATGATACCTCAGG
ABCA5	AAATTATGAGGAGTTATCAG
ABCA5	GATCAAGATAGACAGCCAAG
ABCA7	CTGGCCTAGACGTAACCCTA
ABCA7	TGGACATTGACGTGGTCACG

ABCA7	AGTACGGGATCCCTGAACCA
ABCA7	TCAAACAGCACGTTGTACTG
ABCA7	GCAGCAGCTCCCAAATACCG
ABCA8	CTGTTAATGTCACAAGAGAG
ABCA8	GCAAATCTGCGCAATTGCAA
ABCA8	GACACCCCCAAAAGACAGTG
ABCA8	AAATTATCAGCCAAAAGCGA
ABCA8	GCATTTCCTCATCCATCGGA
ABCA9	ATATCAGAAAAGTTACCCGT
ABCA9	TGGCAGGAAGAACAATCATG
ABCA9	CTTGATAGATGTTCTAACCA
ABCA9	TTATTTCTAACTAGACAGGA
ABCA9	CAGGCCATAGAGGAGAAAGA
ABCB1	TTTATAGTAGGATTTACACG
ABCB1	CTGGAGAGATCCTCACCAAG
ABCB1	TAGGTGATATCAATGATACA
ABCB1	ATTGACAGCTATTCGAAGAG
ABCB1	ACTGATGGCCAAAATCACAA
ABCB10	CTCAGTGCCGTGTTTCTGTG
ABCB10	ACAGCAATGATTGACACTGG
ABCB10	GAGTTCACCCACGGTCATGT
ABCB10	TATCGCCGTTCACTGGCCTG
ABCB10	GATCTTCCCCAGGAAGAAAG
ABCB11	GATTCTTTACTGGATTCGTG
ABCB11	TCAACATGGTCATTAAACCA
ABCB11	TTTCAGAATCCTCCTAACTG
ABCB11	GAGCTCCCCAGATTTAGCAA
ABCB11	CATTTCATCAATGAGAACAG
ABCB4	CATTTCGGACCGTAGACAGT
ABCB4	ACACCACTGAACTCAATACG
ABCB4	CCCTTCTCGAGCTAACGTCA
ABCB4	TGGCCGTGGAAATGTAACCA
ABCB4	GCTGATGGCCATTATCACAA
ABCB5	CAAAGGTCGGACTACAATCG
ABCB5	GAAACCTTCGCAATAGCCCG
ABCB5	AGACGTGGATAGAGTCACTA
ABCB5	TTTGGATTGGCAGATCATGG
ABCB5	AGAGATGGAGAGAGCAGCAA
ABCB7	TCACAGTTGCAGTCACACGG
ABCB7	AGTCATTAAAAAGTATCACA
ABCB7	GATTAAATACCAAAGCACTC
ABCB7	CACACCACAGACAGCTACCG
ABCB7	GATGCATTCTTGGCGCTGGG

ABCB8	AAGTACACAAGGGACCACGT
ABCB8	TCTTTGACGCCAATAAGACA
ABCB8	CCTGCCCAAACAGGACAGAG
ABCB8	CTTGGAACAAGGCGATGCCG
ABCB8	GGTCGTGGCCAAGTACACAA
ABCB9	TGTCCTGGGAGATTGTATGG
ABCB9	TTTGCCGCAGGTATTCGGGG
ABCB9	GCCCCAGACGTAGTACATGT
ABCB9	TGGACACCATCATGATGATG
ABCB9	AGTGGCGGATGTCCTCCAGG
ABCC1	AAAATGTGATTGGCCCCAAG
ABCC1	AACCTGACAGCATCGAGCGA
ABCC1	AGTACACGGAAAGCTTGACC
ABCC1	TCTGCTTCGTCAGTGGCATG
ABCC1	ATAGACAGCCCCAATGACAG
ABCC10	GGGCCCTGAGAATCCCAACA
ABCC10	CGTGTACCAAGCTTACTGGA
ABCC10	CAAGATGAGACCACCCACGA
ABCC10	AAGGGTGTCACCCTTAGCGG
ABCC10	GGAGACCATTGATCACCCAA
ABCC12	AAAGACCATTAGGATCGGGA
ABCC12	ACGTACATTAAGGCTTCTGG
ABCC12	GCCAACCCAGACTTTCCCAG
ABCC12	GAGTCATATGTCGACAATGG
ABCC12	GCATCATCATGGCAGCCATA
ABCC2	GATTGGTATATCGAACAGCA
ABCC2	CAGAATTCATCACAAACGCA
ABCC2	CAGGGTATAAATCTTAGTGG
ABCC2	CATTATGGCAGGCCAACTTG
ABCC2	AGAGTCTTCTGTGAGTACAA
ABCC3	GCAGGGCGTACAGTCTTCGG
ABCC3	CCACGTACACGTACACCCAG
ABCC3	GGCCACATGAGAGTCCACCG
ABCC3	TGAACCACCAGAAAAACAGG
ABCC3	ATACAGTATGAGCGGCTGCA
ABCC4	CAGATTGACTATCTGGCCTG
ABCC4	AAGCACGGGTAAACCTTGCA
ABCC4	GAAGGTACGATTCCTTAGTG
ABCC4	GCGTCACTGCCACGAACACG
ABCC4	AGAAGAACACGCGTGAGCAG
ABCC5	TTTATTCATAGAAATCCGCG
ABCC5	TGTTAAGACTAGAGAGACTG
ABCC5	TGAAAAGGGCCATAATAACC

ABCC5	TACGGAAAGAGGCACCCATG
ABCC5	CTGGATGAGGAGCATCCCAA
ABCC6	CAGACCACGATCCAGACGAG
ABCC6	GGACAGCATCCAATACGGCA
ABCC6	ACTATGATCCAATCAGCCTG
ABCC6	GAGAACTGTGAGAGTCACAA
ABCC6	GTGCTTCCTGAGAACAGCAG
ABCC8	CAGACCAACGAGATGCTCCG
ABCC8	TCAACGCCAGCGAATCAGTG
ABCC8	GATGTTGTGATAGTAGACCA
ABCC8	CTACCGTCAAAGCTCTAGTG
ABCC8	TGATGACATTGACCTCCACG
ABCC9	GACTGCAAATCTGACCACCG
ABCC9	ATACACTATCGATGTTGTAG
ABCC9	AGAGAGGAAAACTCTCCGAC
ABCC9	TAAAGTACTGAATTGGCGCA
ABCC9	ATACCAGCAGAGCTCCACGG
ABCE1	CAATTGATAAGGCGCCAAAG
ABCE1	CCAAAACTTCACCTGGACGA
ABCE1	TTTCAGATATATCATTGTGG
ABCE1	AGTTGTCCTGTAGTTCGAAT
ABCE1	AATGTTGAAGATCTTTCAGG
ABCG1	GAGGCCGATCCCAATGTGCG
ABCG1	ATGAAAGGGCTCGCTCAAGG
ABCG1	GACCACAAGAGAGACCTCGG
ABCG1	CTTCAGGAACCGAATAGGAA
ABCG1	CTACCTTTCTTCCTCCACCA
ABCG2	AAAAAACGAACGGATTAACA
ABCG2	GGAAGGCTCTATGATCTCTG
ABCG2	GAATTACATCAACTTTCCGG
ABCG2	TAACTTAGGATGTCTAAGCA
ABCG2	TTTCTCCTCCAGACACACCA
ABCG8	GAGTCCTACGAAGATGCCTG
ABCG8	CAGTGCGCTGACACCCGCGT
ABCG8	CACCCCCATCTACTTAGGGG
ABCG8	CTCAAACCAAGGGACCTGAG
ABCG8	CTAGATGTGATCACTGGCCG
ABL1	TCAGTGATGATATAGAACGG
ABL1	GGTTCATCATCATTCAACGG
ABL1	TTGCTCCCTCGAAAAGAGCG
ABL1	CTTAGGCTATAATCACAATG
ABL1	GCTAGAGAAGGACTACCGCA
ABL2	AACCTCTGTAATGACGACGG

ABL2	TGTACACCATCACTCCACAG
ABL2	TATCGAATGGAACAGCCTGA
ABL2	GGTTCAACATCACAACCATA
ABL2	CTGCTGCCCCGGATCCCGCG
ACACA	TATTTGCCTAGGTACCGAAG
ACACA	AATGCATGCGGTCTATCCGT
ACACA	TGATGGGTCCATGATAACCG
ACACA	CCAGAGTCCCACATTCCCTG
ACACA	TCAATGGGAGAATCACCCCA
ACACB	ACAACGACATCGACACCGGG
ACACB	AAAGCGTGTGACAAACTCAG
ACACB	GAACTCGAATATGGCCAGCG
ACACB	GTCGGAGTCCATAATCCACA
ACACB	GCACGTTCTGAACATCGCAT
ACADM	AAGATGTGGATAACCAACGG
ACADM	ATTGTGGAAGCAGATACCCC
ACADM	AATTGGCTTATGGATGTACA
ACADM	GTATTTGGGGAGAATGACTG
ACADM	ATTGGCTTATGGATGTACAG
ACADS	GACACTCATGATGACTCCGG
ACADS	ACACACCATCTACCAGTCTG
ACADS	GGCAGGTACCTGGTTCGCTG
ACADS	TAAGGAACATCTCTTCCCAG
ACADS	GCTGATCTCCTCCATGGCGA
ACAT1	GAGGATCAACACCATATGGT
ACAT1	GAAGTGAAAGAAGCATACAT
ACAT1	TTCTGCGCAGGATGTGATGG
ACAT1	CTGCCTAAAAAAGATCCAAT
ACAT1	GTTCCATATGTAATGAACAG
ACE	GGTGTGGAACGAGTATGCCG
ACE	GTTTCGTTTCGGGTAACAGG
ACE	GGAAAACATCTACGACATGG
ACE	GTTGTAGAAGTCCCAAGCCG
ACE	ATATGACCGGACATCCCAGG
ACE2	CCAAAGGCGAGAGATAGTTG
ACE2	CAGGATCCTTATGTGCACAA
ACE2	TGCACAGAGAATATTCAAGG
ACE2	AACATCTTCATGCCTATGTG
ACE2	CATCTTCAATCAACTGGCCG
ACHE	GGTGTCCATGAACTACCGGG
ACHE	TATGTGGACACCCTATACCC
ACHE	TGTCCTCGTCTGGATCTATG
ACHE	TGGTGGGAATGACACAGAGC

ACHE	TCTCGGTGCCCTCAAAACCT
ACPP	ACTCCTTGGCTAGTACACTT
ACPP	AAAGGCAGGTATAGCAACTG
ACPP	CTACGACCCTTTATATTGTG
ACPP	GCCATGAGGATTCCTTTATG
ACPP	GACACCTTCTGGGGGAAACA
ACR	GAAAGCGCCTATGTGGTCGT
ACR	CCCCGACACTTACTCACACG
ACR	AAGCGCCCACACGAAATGGG
ACR	TAATGTGCATGACTGGAGAC
ACR	GCAGCTTGCTGAATTCACGA
ACSL1	CAAGAGCCATCGCTTCAGCG
ACSL1	GTTTCCGAGAGCCTAAACAA
ACSL1	ATCACGTACATAGTCAACAA
ACSL1	GATGCCAATGAACTGATCTG
ACSL1	TCAGCAGTCTTGGAACCACG
ACSL3	GTGGTGAAGAGTAACCAATG
ACSL3	TATCTAAAGTATCACATCCA
ACSL3	ATGATTACTGCAATATCTGA
ACSL3	GAAAGTTCGAAGCTTGCTAG
ACSL3	GGATCCACAGGACTTCCAAA
ACSL4	GTGTGTCTGAGGAGATAGCG
ACSL4	GCATCATCACTCCCTTAGGT
ACSL4	ACCTGGTCAGAGAGTGTAAG
ACSL4	AAGCCCACTTCAGACAAACC
ACSL4	TGATGCATCATCACTCCCTT
ACSS2	GGAACCAAGGGATTGACTTG
ACSS2	CAGTGGCCCATAGGTGACGT
ACSS2	GCATTGTGGTCAAGCACCTG
ACSS2	CAGGAAGGGGAGTCAACATG
ACSS2	TTACCTGCAGGCATTCAGAA
ACTL6A	ACCACCATACCAATAGCTGT
ACTL6A	TGTTCCGAGGGAGAATATGG
ACTL6A	AGAAGTTGCCTCAGGTTACG
ACTL6A	ACTGCAATTCCAGTCCACGA
ACTL6A	CTAATGCTCTGCGTGTTCCG
ACVR1	GCCATCGTTGATGCTCAGTG
ACVR1	CTGGTGTAACAGGAACATCA
ACVR1	CCATGACTTCTCATCACGGG
ACVR1	ATTACACTGTTGGAGTGTGT
ACVR1	TTACCTTCCATACTAGGGGA
ACVR1B	CCGGTTCAGATAATCAAACA
ACVR1B	ACTTGACTCAGGTCACCTCA

ACVR1B	CTACACGTGTGAGACAGATG
ACVR1B	AAGAGATTATTGGCAAGGGT
ACVR1B	GGAAGCAGAGATATACCAGA
ACVR1C	ATTGTCCTTTGAACCAACAG
ACVR1C	TGTAGGAGCACTGTCGACCC
ACVR1C	CTGGCGCTCTCAATTGCTAG
ACVR1C	CCAAGATCTTTCATCTCTGG
ACVR1C	GCATCAGTCATGCTAACCAA
ACVR2A	ATTGCAGAAACCATGGCTAG
ACVR2A	CCAAAGATCCACATCAACAC
ACVR2A	CCAGTTGCTTAACGAATATG
ACVR2A	AGTGGTTTCAAACCTAGTAA
ACVR2A	GAGTTGGAACAAGTACAGGA
ACVR2B	ATGTCCACATGACCGTAGGG
ACVR2B	ATGACTTCAACTGCTACGAT
ACVR2B	ACAAGCCGTCTATTGCCCAC
ACVR2B	CTGGAGCGCACCAACCAGAG
ACVR2B	ACAGCAGCAGAAGTACACCT
ACVRL1	CTGCGTGCTCGAGTTGCGGG
ACVRL1	CCAGGACTGTTCATCCCTCG
ACVRL1	TGGACAGTGACTGCACCACA
ACVRL1	AGGCACCCCCAGGAACATCG
ACVRL1	CCTCGAGGAGAAGATCTTGA
ACY1	CCATGCCTCACGCTTCATGG
ACY1	TAGCTTAGTCAGGTTCACGG
ACY1	GCTGGACAGTGCGGATACGC
ACY1	AGGCACAAAGGTCATGTGGA
ACY1	CATCTTGCTCAACTCCCACA
ADA	TCACCGTACTGTCCACGCCG
ADA	TGGACATACTCAAGACAGAG
ADA	CACAGACTGGTCCCCCAAGG
ADA	TCCCAGCTAACACAGCAGAG
ADA	GTAGAGATGAAGGCCAAAGA
ADAM10	CCCATAAATACGGTCCTCAG
ADAM10	TTTCAACCTACGAATGAAGA
ADAM10	TTCCATCAATAACAGACCCA
ADAM10	GGAAATGGAATGGTAGAACA
ADAM10	GGAAATCTAGACGTAAAAAT
ADAM12	GGTGATCCTTATGGCAACTG
ADAM12	AAGCAATGGTACTTGCCCGG
ADAM12	ACTGAACATTCGGATCGTGT
ADAM12	TGCTGAATATTCGACTACAA
ADAM12	GCATTGTCATGGGATTTGCG

ADAM15	TGGATTCGCGAAATAATGGG
ADAM15	AGTGCCACTCGTACATTCAG
ADAM15	CTCCAGACAGCTAATACTCG
ADAM15	CTGTTGTCAAAATTGCCAGG
ADAM15	AGAGTCAGAGAAGGCCCCGA
ADAM17	AATCAGAATCAACACAGATG
ADAM17	TGGTGAAAAGCACTACAACA
ADAM17	CATCGCTTCTACAGATACAT
ADAM17	ACAAAATTTCAAGGTCGTGG
ADAM17	GTAGACAGAGAACCACCTGA
ADAM2	TTGAAAAAAGGATCTAAGCG
ADAM2	GACCTCCCTGAATATTGCAA
ADAM2	CTTTAAATTACAAAGCGTAG
ADAM2	ATGTGTGATGCAAACTATGC
ADAM2	AAGCCAACTGAAGACTCCAG
ADAM8	AGTCCGGGTACCCCTCTACG
ADAM8	CCACATACAGCTCCACGTAG
ADAM8	CAAACAGGTTCCCACACACG
ADAM8	CCAGCTTAGACTCACCAAGT
ADAM8	GGACACCCTGGCAAACCCCA
ADAMTS1	CTATGTGGAAACCATGCTTG
ADAMTS1	GAGGAGTCCAGTACACGATG
ADAMTS1	GAAAGCGGAGACCGAAGACG
ADAMTS1	GTAATCATGTAGGCACTGCA
ADAMTS1	AATGCTTTAGACCACTGCCG
ADAMTS4	AGCGCTTTAGCCCCGCACCG
ADAMTS4	CATACCCAGCGTGTCGCAAG
ADAMTS4	AGGGGCCATGACATGGCGAG
ADAMTS4	CCAGACCAAACACTCGCCCT
ADAMTS4	GTGTTTCCAGAGAAGCTCAA
ADCY1	CATGTTCAGATCCACCTCGG
ADCY1	AACCGGGTTACGGACATGAG
ADCY1	CGGGGTGGTGTAGACAACGT
ADCY1	CCCAGACGAAAGGACCACCA
ADCY1	GAGATGGCGGGGGCGCCGCG
ADCY2	TGAAGCGGCATACAAACGTG
ADCY2	ACTCCAAGTACCGGGTCATG
ADCY2	CCTGTAATTGCATCAAGTCG
ADCY2	CAGGCAGACGCTAAGCACGA
ADCY2	TATCGCAAAGAAAATCGCCA
ADCY3	GTCGATGAGTATCTCGACCA
ADCY3	CCACGGAGATGATCACGATG
ADCY3	AGCTGTCTCCAGTACTACAC

ADCY3	TCTCTTCTAGGTAATCACAG
ADCY3	CATGGGTCCGGCCCACCCCG
ADCY6	TTGGCCTGGCAACTTAACCG
ADCY6	ATAGATCCCAAGCATCAGGG
ADCY6	GATGACGCCTTCATCCGGAG
ADCY6	AACACTGCAGTACCTGAACG
ADCY6	ACTGGAACACCTGCACCAGA
ADCY7	TGTACGTCGAGTGTCTCCTG
ADCY7	AGAGGCACCAGAATGTCAGG
ADCY7	GCGTGGGTAGGCAGCGACAC
ADCY7	CCGTAGCCGGGTGCACATCA
ADCY7	TCAAAGAACCGAGCACCAGG
ADCY8	CAAAACACGATGTTGACATG
ADCY8	ATTTCCAGGACAACAAACCG
ADCY8	GCTGCAGGTCATCCTCCAAG
ADCY8	CGATATTTAAGATGGCACCC
ADCY8	TGGGGGCTATAGCTACCGAG
ADCY9	GCTACCACGTCACTGCATCG
ADCY9	GGGAGACTGTTACTACTGCG
ADCY9	TGTACGCCCGGCATTACGCG
ADCY9	GATATCGGGTCAGAGAGCCA
ADCY9	GCTGCATCACCACAGCACCG
ADCYAP1R1	GGTGAGCCGGAACTGCACGG
ADCYAP1R1	TGTGGGACAACATCACGTGT
ADCYAP1R1	TGAATATGAATCTGAGACTG
ADCYAP1R1	CGAATCTTCAACCCAGACCA
ADCYAP1R1	GTGCCTGGAGAAGATCCAGA
ADGRB1	CAGGATGAGTACCGGCAGTG
ADGRB1	CCCACGATGAGCGTCACCGA
ADGRB1	CCTGTGGGGCGAATGCACGC
ADGRB1	GAAGCATCCGTGTTTGTGGT
ADGRB1	GCTGGAGCACACGCTCCACG
ADGRB2	ATCATCAGCCGAGGGCACGT
ADGRB2	CAGCCACTGATAGCAAGTGG
ADGRB2	TGAGCAAGACCTCGACAAAG
ADGRB2	CTCTCTCGTAGGCGACCCGG
ADGRB2	GGTGGGGTCAGGGTTCTCCA
ADGRB3	CCAAGTCTGCAATCTTACCA
ADGRB3	AGGCGAATAAGGACCTGTCA
ADGRB3	TACAGTACACGGAGTATGGG
ADGRB3	AGGGTCAATAGAGTTAATGC
ADGRB3	GTTGATGTCTGTTAGAACAG
ADGRE5	ACCGTCACAAGTCTCCGTCG

ADGRE5	CCTTATGGCTCATTATGACG
ADGRE5	GGCCTCACCTGTGTTCGAAG
ADGRE5	CCCATCGGAGGACTCAAGGT
**ADGRE5**	AGTACTTACCGAGAAAGACG
ADH1A	CACACTGAGGAATAGCGAGT
ADH1A	CCATGGTACCACTAACCACG
ADH1A	ACAGACTTTCTCTAGAGGCG
ADH1A	CCAGTGTAAGCAATCCTCAG
ADH1A	CACCTTCTCACAGTACACAG
ADH4	AAATTTAGAATCGATAACAG
ADH4	TACATTCTCTCAGTACACTG
ADH4	TCAACCTCTTCAATGCAAAG
ADH4	TTCCCACACAAATTTGTGAG
ADH4	GGCATCAGTATGGCACAGAG
ADH5	TCAGGGTATAGGCATCGGTG
ADH5	TGCTTCCCGGATCATTGGTG
ADH5	TGGGTTTACTAAAATCCTGA
ADH5	GCTGGAATTGTGGAAAGTGT
ADH5	GGGAAAGGATTAATGCCAGA
ADH7	TACATTTACCGAGTACACAG
ADH7	CCTACTCGCTCCTAATGCAA
ADH7	CTTGGGACTGATACACTCAG
ADH7	AAGACGACGCAAGTGGAACC
ADH7	TCTATTTCCTCAATGGAGAA
ADK	ACAGCAGAGATGTCAAGCAG
ADK	AAAGTCGAATATCATGCTGG
ADK	TCTGGAGAAAAACTGGATGT
ADK	GTAGTAATGAGCATCCACAT
ADK	CATGCAGCACAAGTTCCTGT
ADORA1	AGGGGTCAGTCCCACCACGA
ADORA1	GAGGACCATGAGGAGAAGCG
ADORA1	GGAGAGGGATCTTGACCCGG
ADORA1	GGCAGCCAACGGCAGCATGG
ADORA1	GATGGTGGTGACCCCCCGGA
ADORA2A	GCGGCGGCCGACATCGCAGT
ADORA2A	TGGCTTGGTGACCGGCACGA
ADORA2A	ATGCTAGGTTGGAACAACTG
ADORA2A	AAGCAGTTGATGATGTGTAG
ADORA2A	CTATTTGCGGATCTTCCTGG
ADORA2B	TCTCAAAGAGACACTTCACA
ADORA2B	GGTATAAAAGTTTGGTCACG
ADORA2B	CACCAGCATTATAAGCAGTG
ADORA2B	TGGAGTCAATCCGATGCCAA

ADORA2B	GGGGATGGCGAAGAGCCCCA
ADORA3	GATGCCCAGGCTGACAACAA
ADORA3	ATGGCGCACATGACAACCAG
ADORA3	GACATTTCTGTGGTACTCTG
ADORA3	ATTGGACTCTGCGCCATAGT
ADORA3	CCTTGGCTCTCATTTCAGGT
ADRA1A	CCAAGACGCACTTCTCAGTG
ADRA1A	TGGCCTCAAGACCGACAAGT
ADRA1A	GGGTCCAATGGATATGACCA
ADRA1A	ATCTCCATCGACCGCTACAT
ADRA1A	GGTCTTCTGCAACATCTGGG
ADRA1B	TCTTGGCCACTATATAGACA
ADRA1B	CCATTCCAAGAACTTTCACG
ADRA1B	CTCCATCGATCGCTACATCG
ADRA1B	CATCCTAGTCATCTTGTCTG
ADRA1B	GCCCCAGCTGGACATCACCA
ADRA1D	TGCGCGCGACCACGTACACG
ADRA1D	ATCTCCGTGGACCGGTACGT
ADRA1D	TACCGCAGAAGCGCTCGTCA
ADRA1D	CGGCGACGTGAATGGCACGG
ADRA1D	TTTCATCGTGAACCTGGCCG
ADRA2A	ATGATGGCCTTGATGCGGCG
ADRA2A	TGGTCGTTGATCTCGCAGCG
ADRA2A	TCCAGCGCGTCGGTGTCGCG
ADRA2A	GACGAGCACGTTGCCGAACA
ADRA2A	GACGGCCGAGATGACCCACA
ADRA2B	TGGAGTTGTACTCCAGCGCG
ADRA2B	CGGACACTCGAAGTCCACTG
ADRA2B	TGGTCGCCCTTGTAGATGAG
ADRA2B	CAGCCCCGACCCGACCATGG
ADRA2B	TCGCCCTTGTAGATGAGGGG
ADRA2C	ACACAGATGCACGATCGACG
ADRA2C	CTACGCGCGCATCTACCGAG
ADRA2C	TGTACCAGGTCTCGTCGTTG
ADRA2C	ATGGTGGCCTTGACGCGGCG
ADRA2C	CACCTGCCCGAAGTACCAGT
ADRB1	GTAGAAGGAGACTACGGACG
ADRB1	TGGCCATCGCCAAGACGCCG
ADRB1	CGCACGCCCGTTGGCCAGCG
ADRB1	TGGCCCACACGGTGCACACG
ADRB1	AGAAGGAGCCGTACTCCCAG
ADRB2	ATCCACTGCGATCACGCACA
ADRB2	CAGACGCTCGAACTTGGCAA

ADRB2	CCCTTTCCTGCGTGACGTCG
ADRB2	GGGAACGTAGAAGGACACGA
ADRB2	GCTGACCAAGAATAAGGCCC
ADRB3	CAGCGAAGTCACGAACACGT
ADRB3	GATGGGCGCAAACGACACCG
ADRB3	CCAATACCGCCAACACCAGT
ADRB3	ACCAGTGCGCCGTAACGCAG
ADRB3	AGAACAGCTCTCTTGCCCCA
ADRBK1	CCAGGTCCGAGATCCGCACG
ADRBK1	TCTGGAACACGTCCCCTCGG
ADRBK1	CATCGCATCATTGGGCGCGG
ADRBK1	CTCCTCATAGAATTCCACCA
ADRBK1	GATTTGTCAAAACCTCCGAG
ADRBK2	ACTACTTACACACTCGCATG
ADRBK2	TCTTCATAAAACTTCACCTG
ADRBK2	GATGAAGCAGAGTTTATCTG
ADRBK2	TATTGGACGAGGAGGATTCG
ADRBK2	ACACACCGTGTTGTGAAAGG
ADSL	AAATGTGTGAAACCTAATGT
ADSL	CAGCAACGTTCTGAACGCAT
ADSL	GGTATAAATTCCGGACATGG
ADSL	TATTGAAGTACTGTCTGTGC
ADSL	GGAGAACATCGACTTCAAGA
AEBP1	CCAGACCTTTCATGGGAACG
AEBP1	GCACCAGGCTGCGCACACGT
AEBP1	GCGCCGAATGTACTCAACTG
AEBP1	GTGTGACTCCATCCCAATGG
AEBP1	GCTAGAACCTGAGCCCCGGG
AFM	AGAGAATATTCACCGTGTCA
AFM	TATAACAAGAAATCTGATGT
AFM	AAAAAATATGTGCTATGGAG
AFM	AGTCAACTGCCTTCAAACAA
AFM	GCATTTCTCTTCGGGATCCA
AFP	ACATTGACCACGTTCCAGCG
AFP	AGTGGCTTCTTGAACAAACT
AFP	ATTGTAGGTGCATACAGGAA
AFP	AGTTGAATGCTTCCAAACAA
AFP	GAAGACTGTTCATCTCCAGT
AGT	AGAGCGTGGGAGGACCACAG
AGT	CTTACCTTGGAAGTGGACGT
AGT	TGTCCTTCCAAGGAACACCC
AGT	TTGACACCGAAGACAAGTTG
AGT	TCCCAGATAGAGAGAGGCCA

AGTR1	GCTGTGTAGACAGCCCATAG
AGTR1	CAAGCATTGTGCGTCGAAGG
AGTR1	GTAGAAACACACTAGCGTAC
AGTR1	TCAGGCCCAGCCCTATCGGG
AGTR1	TCATTGGGTGAACAATAGCC
AGTR2	ACAGGCCATACACCAAACAA
AGTR2	GTACCTATCAACACTCATGC
AGTR2	ATTGGGCATATTTCTCAGGT
AGTR2	GGAGTAAATCAGCCACAGCG
AGTR2	ATAGAGGAAGAGTAGCCAAA
AGXT	GCTGCTGTTCTTAACCCACG
AGXT	CCCCTTTACATGGACCGGCA
AGXT	CCGATGACCAAGGACCCTGG
AGXT	GTCACTGAAGGAGATGAGCG
AGXT	CTCGCATCATGGCAGCCGGG
AGXT2	ACACTTGGCTTGACAAACGT
AGXT2	GCACTGGAGAATCTCGACAG
AGXT2	CAGGAGAAAGATGTTCCTTG
AGXT2	AACACTGACAGTAACAATCC
AGXT2	GCAGTGGCACAAAAGCAGCT
AHCY	AACATGATTCTGGACGACGG
AHCY	CTCTGAGGAGACCACGACTG
AHCY	GCGGGCTCCGAAACCCCGCA
AHCY	GATGATGTCAATACAGCCTG
AHCY	GTATGCCTGGAAGGGCGAAA
AIMP1	ACCATAGAATTAGCGTGCAG
AIMP1	GCCCCAAGGACAGTTGTCAG
AIMP1	GCAGTAACAACCGTATCTTC
AIMP1	AACCAATTCGAAGATCCAGA
AIMP1	GCGTGCAGTGGAGTACCAGA
AK1	GATTGATGGCTACCCGCGGG
AK1	AGTATTGACTTTGGCCACCA
AK1	GCCCTGAGACCATGACCCAG
AK1	AAGAGTTTGAGCGACGGGTA
AK1	CTACACCCACCTCTCCACCG
AKR1A1	GTGGCGGTAGCCTACGCTAA
AKR1A1	CAAGACAGCTGGACGCACGG
AKR1A1	TGTTTGTGACATCCAAGCTG
AKR1A1	TAGTCCCATCAGCATTCTTG
AKR1A1	GTCAGCCAGAGTCTTCCGGA
AKR1B1	CACCAACATTCTGGACACGT
AKR1B1	TGGTGCACGTACCATGAGAA
AKR1B1	CATGGGCACAGTCGATGTGG

AKR1B1	TGTTTAAGATCATCTCCACC
AKR1B1	TCTCCTTTCAGTCCCCTCCA
AKR1B10	TCTTAAACCTTGAATCCCTG
AKR1B10	GATAAAGGTAATGCCATCGG
AKR1B10	AGAGAAGGCTGTGAAGCGGG
AKR1B10	GTGTCACCCATACCTCACAC
AKR1B10	GCTTCTTTCACTTTGCCAAG
AKR1C1	AATGAGCAGAATCAATATGG
AKR1C1	CCAAACACTCACCTCCCATG
AKR1C1	TGGCACCTATGCGCCTGCAG
AKR1C1	CTGGAAAATGAATAAGGTAG
AKR1C1	GGATCTCTGTGCCACATGGG
AKR1C2	CATGTGCAGAATCAATATGG
AKR1C2	GGATCATCTCCAGCAGCCTG
AKR1C2	AGTGGATCTCTGTGCCACAT
AKR1C2	CTGGAAAATGAATAAGATAG
AKR1C2	GGCTTCTATTGCCAATTTGA
AKR1C3	AGAAATCTAGCAATTTACTC
AKR1C3	AATGAGCAGAATCTATATGG
AKR1C3	GGGTGTCAAACTTCAACCGC
AKR1C3	GGATCTCTGTACCACCTGGG
AKR1C3	CCAAGCACTCACCTCCCAGG
AKR1C4	AAAAGTAATATTCGACACAG
AKR1C4	AATAAGCAGAATCAATATGG
AKR1C4	ATAGGTGCCAAATCCCAATA
AKR1C4	TCCCTACCTTGAGAGCCATT
AKR1C4	CTATCTTCTTCATTTCCCAA
AKR1D1	GATCCACATAATCTAGCTGG
AKR1D1	TGAAGGTTGCTATTGACACA
AKR1D1	TAGGTACCAAGTCCGATGAT
AKR1D1	TTGGCTGGGTGAAATACGGA
AKR1D1	TGTAGGCCCCATCAATATGT
AKT1	GAAGGTGCGTTCGATGACAG
AKT1	CCTGCACTCGGAGAAGAACG
AKT1	TGTTGAGGGGAGCCTCACGT
AKT1	TGTCATGGAGTACGCCAACG
AKT1	GCTCCTCAGGAGTCTCCACA
AKT2	TCTCGTCTGGAGAATCCACG
AKT2	GACCCCATGGACTACAAGTG
AKT2	GGGGGGTAGAGTCTGATCAG
AKT2	CTCTTGAGTACTTGCACTCG
AKT2	CATCGAGAGGACCTTCCACG
AKT3	CTGCACCATAGAAACGTGTG

AKT3	ATTTCATGTAGATACTCCAG
AKT3	ACAAATTGATAATATAGGAG
AKT3	TATTTGAAACTACTAGGTAA
AKT3	AACCCAACCTTCTTTCACAA
ALAD	ATGGATTGCCTCAATAGCTG
ALAD	GGACATGATGGATGGACGCG
ALAD	AGACTCACGTGACAAAGATG
ALAD	CACACAGGTATGGTGTGAAG
ALAD	GCCAGGCCCGAAGTAGTGGG
ALB	CCTCTGGTCTCACCAATCGG
ALB	ACTTTGGCATAGCATTCATG
ALB	TCTTATCTACTTACATGCCC
ALB	GCAACTCTTCGTGAAACCTA
ALB	CTTTAGCTCGGCTTATTCCA
ALDH18A1	ACTTGCCATGTGTACGACTG
ALDH18A1	GCAGCTTTGGCTATCGCAAG
ALDH18A1	TGCCAATGGAACCCACCCAA
ALDH18A1	CTAACAGGATCTCATCACGC
ALDH18A1	GTACATACGAGATCTGAAGA
ALDH1A1	AGCATCCATAGTACGCCACG
ALDH1A1	AGCCTTCACAGGATCAACAG
ALDH1A1	GAATGGCATGATTCAGTGAG
ALDH1A1	TGTTGAATTTGCACACCATG
ALDH1A1	GGTTGGGCTGACAAGATCCA
ALDH1A2	CTTGATTGAAGAACACACCC
ALDH1A2	TTAATTTCGAGATTGGGCGT
ALDH1A2	CCAGCCTGCGTAATATCGAA
ALDH1A2	AAAATATTGATGACCCCGGG
ALDH1A2	GCTGGGCTGATAAAATTCAT
ALDH1A3	TGGATGCCCTGAGTCGTGGG
ALDH1A3	CAGAGAGCCGAGATAAAGGG
ALDH1A3	CGTCCGCACACACGATGCAG
ALDH1A3	GAATGGCACGAATCCAAGAG
ALDH1A3	GGGTGGGCAGACAAAATCCA
ALDH1B1	GTGCCCAATGACCTCCCCGG
ALDH1B1	ATTGTCCAAGGTCTCGAGTG
ALDH1B1	GGCATCCATCCGGCGCCATG
ALDH1B1	TCCAACCCTGCATGACCAAG
ALDH1B1	GCTCCACTAGGTCTGCCAGG
ALDH1L1	CTGGCCCTGAAGACCCACGT
ALDH1L1	TTGCTCCCCACAGATCAACT
ALDH1L1	TCATCAGGGGATAGTTCCAG
ALDH1L1	ATGCCTTTGAGAATGGACGG

ALDH1L1	GATCCTCCCCAAAGTCCTGG
ALDH2	TTCCACGGCCCAATCCACTG
ALDH2	CGCATGGACGCATCACACAG
ALDH2	CAGTGGACGGATTGACGGTG
ALDH2	AGCCTTGGCAACTGGAAACG
ALDH2	GGACCATGTCCAAATCCACC
ALDH3A1	CTGTACCCAGTAATCAATGG
ALDH3A1	AGGGAAGAGTCCCTGCTACG
ALDH3A1	GGAGGTGGTGTACGTCCTAG
ALDH3A1	TTCTCCACAGGAGTTCTACG
ALDH3A1	GGACGAGCTCTACATCCACT
ALDH5A1	TAATGTCTCCGTAAACACGG
ALDH5A1	AGTATTTGACAGTGCCAACG
ALDH5A1	GGGCATGGTAGCCGACTGCG
ALDH5A1	GGCACTGGGGAAATTCCACT
ALDH5A1	GAACCACTCTAGGAAAAAGG
ALDH7A1	ACTCACCAGTCGCCTCGCAG
ALDH7A1	GGGCTCCGCGAGGAAAACGA
ALDH7A1	ATTGAGCAGTGGAATCCCGT
ALDH7A1	ATTCTTAATTTAGGCCAGTG
ALDH7A1	TCAATCAGCCCCAGTATGCG
ALDH8A1	TTCTTACGACCCATCAACAG
ALDH8A1	CAGGCCGAGTCTAAAGACCA
ALDH8A1	TGCACGCAGATGGACCACCT
ALDH8A1	CAGCTGGGTGATCCGCTCAG
ALDH8A1	GCACTCACCGACTCCCACCG
ALDH9A1	TCTGTGTCAGCATCCCGATG
ALDH9A1	ATATGAACAATGCTGTAAAG
ALDH9A1	GAGCGGCTGCGACACGACGA
ALDH9A1	GTATAACCAAACGATCCACC
ALDH9A1	GCTTTACCCAAAGGAACGGG
ALK	CCATACCTTAAATACGTAGG
ALK	CTGTAGCACTTTCAGAAGCG
ALK	TCCAGACAACCCATTTCGAG
ALK	CTCTATTGCAGTTAGCGGAG
ALK	GATGCCCGAGAAGAAGGCGT
ALOX12	TCCATCTTCAGCATAACGAG
ALOX12	CCCCCATATCCGCTACACCA
ALOX12	TCCAAATATGAGATTCCATG
ALOX12	TGAAGCTCTTCCATCCCCGA
ALOX12	GGGCCGCTACCGCATCCGCG
ALOX12B	CAAGACGTGGGTACGCTATG
ALOX12B	CCAGTTACCGCCCTCCGGTG

ALOX12B	GTTCCCCGTCACAGACGACA
ALOX12B	TGCACATAGTTGCAGTACCA
ALOX12B	GCACTGGCTTTCAAAGTCCG
ALOX15	CAAACTATATGACCTCCCTG
ALOX15	AAGCGACTGTGGCCCGCACG
ALOX15	CAATTTCAGCATGACTAGAG
ALOX15	TTCCAGGGTGTATCGCAGGT
ALOX15	CCAGAAATCGCTCATCCACA
ALOX5	GGATTCATACGACGTGACTG
ALOX5	AACTCGATGTAGTCCCCGTG
ALOX5	CATCGATGCCAAATGCCACA
ALOX5	TAGAGCGGGTCATGAATCAC
ALOX5	GCTGCACTCTACCATCTCCG
ALOXE3	GTACAAGGTGCGTTGCACAG
ALOXE3	ACGACAACTTGTGTAGACCA
ALOXE3	CTTCAATCCACTGATAGCAG
ALOXE3	CGAGGGCTGTTGGATCGCAA
ALOXE3	TGTTGACGTCTACCATGCAG
ALPI	CCGTTCGCAGACATACAATG
ALPI	CATGATGACCATGGTCGATG
ALPI	CAACACGACACGCGGCAATG
ALPI	GATCCACCGAGACCCCACAC
ALPI	GCTGCCCCCAAGACATGCAG
ALPL	GGCATGGTTCACTCTCGTGG
ALPL	CATTGGCCTTCACCCCACAC
ALPL	GATGACATTCTTAGCCACGT
ALPL	AGCTCTTCCAGGTGTCAACG
ALPL	CCACTTCATCTGGAACCGCA
AMBP	TGCAGAATGGAACATAACCA
AMBP	TAGAGCTTGGCAGTAATGGT
AMBP	TTCTAGGAAAGGTGTCTGTG
AMBP	CCTAGGTGAATGTGTCCCTG
AMBP	GCAGCAAGAGCAGGGCCCCG
AMD1	CCCACAAGGATTGAACATTG
AMD1	AAGCTTGCTAGGGATTACAG
AMD1	ATCTTCGCACTATCCCAAGG
AMD1	AATTCCGGTGTGGGTACCCT
AMD1	CCATTCATCGAATACCCACA
AMN	GCAGACGTTCACGCGCGACG
AMN	GCGGAAAGAAGACGTCGTCG
AMN	GGGCTGCGTCTGCGGCAACG
AMN	GGCGGGGGCGCTTACCACAG
AMN	CGGCGCCGTTGAGTTCCCGG

AMPD1	ACAGTAAGACCTATACCCAC
AMPD1	GCAGGTACATACTAGATCCT
AMPD1	CAAGGACCAACTCACCCCAG
AMPD1	TATCACCTCAAAATGAAGGA
AMPD1	CCACCTCCACAGAAGCCAGG
AMPD2	TGCCCCGTATGAGTTCCCCG
AMPD2	TGCCAAATACAACCCTATTG
AMPD2	GAGACCCGGACCTATGAACA
AMPD2	GGTGCACGTCTACACCCGCA
AMPD2	GGTGGGGCAGAAGCTCTGCA
AMPD3	TGATCCGGGAGAAGTATGCG
AMPD3	TCACGTTATAGAAGTCCCGG
AMPD3	CACCTCACTTACTAAATCCG
AMPD3	AAAACTGAAAACTATCTGGG
AMPD3	GGACTCACTGGATGTCCACG
AMT	ACTGTCGCTGTTTACCAACG
AMT	AGAGGGGGTATACCTGCATG
AMT	CCAGGTACTACAGGCCGGCG
AMT	TGTAGCCACAGCGGGTCACG
AMT	GTTGCAGGCGACGATGCAGA
ANG	AATGTGTTGATGTCTTTGCA
ANG	ATCCTGAGCCAGGGTCGGTG
ANG	GATACTGTGAAAGCATCATG
ANG	CACTATGATGCCAAACCACA
ANG	AGACCAACAACAAAACGCCC
ANGPT1	TTGCAATATGGATGTCAATG
ANGPT1	GCAGCTTGAGAATTACATTG
ANGPT1	AGATATAACCGGATTCAACA
ANGPT1	GCAGAGAGATGCTCCACACG
ANGPT1	GAATGCAGTTCAGAACCACA
ANGPT4	GGTGCCCAGCTCTAGCATGG
ANGPT4	GCCCTCACCAACATCGAGCG
ANGPT4	CTGGATGGTGTAGACACCAC
ANGPT4	GCTTCAGGGCCAAAACAGGT
ANGPT4	GCAAATGGCCCAGAATCAGA
ANGPTL1	TACCTGAACAAAGAATAACA
ANGPTL1	GGGAGGTAACGAGATTCAGA
ANGPTL1	GAAAACAGTTTGGACCCTGG
ANGPTL1	GAAGATGGCAACAAGATACA
ANGPTL1	GATGGACCTTGAAAACCTGA
ANGPTL2	TGCGACCAGAGACACGACCC
ANGPTL2	CCTAAACAGGTACAAGCGGG
ANGPTL2	CTTACCCGACGGCTTGTCGG

ANGPTL2	TGACCCGCGAGTTCATGTTG
ANGPTL2	GCTTGAGGAGCACTGCCAGA
ANGPTL3	AGACTTTGTCCATAAGACGA
ANGPTL3	TGTACCACCATTTATAACAG
ANGPTL3	ACAAAACTTCAATGAAACGT
ANGPTL3	CAAAGACCTTCTCCAGACCG
ANGPTL3	GACTTTGTCCATAAGACGAA
ANGPTL4	GTGCTACTGAGCGCTCAGGG
ANGPTL4	TTGCAGTTCACCAAAAATGG
ANGPTL4	GAGATGAATGTCCTGGCGCA
ANGPTL4	CCACAAGCACCTAGACCATG
ANGPTL4	GCAGGCTGTGAAGGACCTCA
ANGPTL6	GGAGTGTATGAACTGCGAGT
ANGPTL6	GTAGCACCAGTGACACCAGT
ANGPTL6	CGCGTAACAGCTCCTCGTGG
ANGPTL6	GCGCCCGTGAACTTCTGCGG
ANGPTL6	GGAGCGGCCCCGCATCCACG
ANGPTL7	GGAGAGGGACTGGGTCAGCG
ANGPTL7	CCTTTGTCAGCCACCCAGCG
ANGPTL7	CAAAGCGGCCAACTGCTGTG
ANGPTL7	GACTGGAAGCAGTACAAGCA
ANGPTL7	GAGTGAACTGAACAAGAAGC
ANPEP	CGTTCAGGGCATAATCGCCG
ANPEP	TCACGGTGGATACCAGCACG
ANPEP	CATCACGCTTATCCACCCCA
ANPEP	CCTTGGACCAAAGTAAAGCG
ANPEP	ATACTGGCTGTCCTTCACCA
ANXA1	AGAAATCAGAGACATTAACA
ANXA1	TCACACCAAAGTCCTCAGAT
ANXA1	AAGGCAGCGACATCCGAGGA
ANXA1	CCTTACAGGTCACCTTGAGG
ANXA1	ATGCAAGGCAGCGACATCCG
ANXA2	ACAGGGGCTGGGAACCGACG
ANXA2	CAGCCATCAAGACCAAAGGT
ANXA2	TGGGAAACCAACCTTTGCCA
ANXA2	GGTCCTTCTCTGGTAGGCGA
ANXA2	ATACTAACTTTGATGCTGAG
ANXA3	CTGAGAGGTCAAATGCACAG
ANXA3	TTTGCATCAAAGACTGCTGG
ANXA3	GAGGAAAGCTCCTTAAACAC
ANXA3	GAATAGATTCTCTATAAAGC
ANXA3	GATAATCTCTTACTGTTCCT
ANXA5	AGGCTGGAGAACTTAAATGG

ANXA5	ATAAGCATCATAAAGCCGAG
ANXA5	AATCTCCACGCAATACCTGA
ANXA5	ACTCTTCGGAAGGCTATGAA
ANXA5	ACTTCCCTGGATTTGATGAG
AOX1	GGGGTTCTACATACCCACAG
AOX1	GAGTTATGCATCTCATATGG
AOX1	CTGGTGTGAAGCATGCCACG
AOX1	CATATATAACCTACCACCAA
AOX1	GCAGTTCCTTCAGGGTCACG
APCS	ACTATAGGCTCGAAAACACA
APCS	GGAGAGTATAGTCTATACAT
APCS	CTCCTACAATACCCAAGGCA
APCS	GTGAAAAAGGGTCTGCGACA
APCS	TGTGAGCAAAGGCTTCCAGG
APEX1	ATGCCGTAAGAAACTTTGAG
APEX1	CCAAGAGCAGATCTTGAGTG
APEX1	ACAGCATATGTACCTAATGC
APEX1	CTGGTCAGCTCCTTCGGACA
APEX1	AGAGGCCAAGAAGAGTAAGA
APOB	AAGTCCATGAGTTAATCGAG
APOB	AAACTCACTTGTTGACCGCG
APOB	GACATGACTTTCCGGCACGT
APOB	GGTCTCTACCACCAAAACGG
APOB	GCTGCGAGAGATCTTCAACA
APOE	GGCCTACAAATCGGAACTGG
APOE	GCGGACATGGAGGACGTGTG
APOE	CATGCTCGGCCAGAGCACCG
APOE	AGCTGCGCCAGCAGACCGAG
APOE	GCATGGCCTGCACCTCGCCG
APP	GCTGCAGCGAGACCTACCCG
APP	CAAGTATCTCGAGACACCTG
APP	ACATCCGCCGTAAAAGAATG
APP	CGGAACTTGTCAATTCCGCA
APP	CGGAATTGACAAGTTCCGAG
APRT	CGCCTGCGATGTAGTCGATG
APRT	GCTGCGTGCTCATCCGAAAG
APRT	TCTTACCTTCCCGTACTCCA
APRT	TCGCGCCAGGAGGCCGATGG
APRT	AGCTGCAGCTGGTTGAGCAG
AQP1	GCGTGCTGGCTACTACCGAC
AQP1	ACACCCCACTCACGTCATTG
AQP1	ACTGGGCGATGATGTACATG
AQP1	CTGGGCTTCAAATACCCGGT

AQP1	GCCCCTGACTCACAGCCAGG
AQP2	CACGCCAGCAGACATCCGCG
AQP2	CATGGCAATCTGTAGCACAG
AQP2	GGCAGGCCACAGTCACGGCA
AQP2	TCTCCGAGCCGCCTTCTACG
AQP2	CATTGACAGCCAGGTCCCCG
AQP4	AACCAGGAGACCATGACCAG
AQP4	AAAGCCTTTACCGGTCGACA
AQP4	CAGTGCTTTGGCCATATCAG
AQP4	TGCGATGTAGAAGACAGACT
AQP4	CCATGGCCACAGTCACTGCA
AQP5	GCTCAACAACAACACAACGC
AQP5	GACAGACAGGCCAATGGACA
AQP5	GGTGCGGCGGGAGTCAGTGG
AQP5	GGCTGGCATCCTCTACGGTG
AQP5	GGGCAATCTGGCCGTCAACG
AR	AGGGTACCACACATCAGGTG
AR	CCTTAAAGACATCCTGAGCG
AR	GGACGCAACCTCTCTCGGGG
AR	TCCAGCTTGATGCGAGCGTG
AR	TGGTAATCTGAAACTACAGG
ARAF	GCCCAACAAGCAACGCACGG
ARAF	GTAGTGATGGAACCCCCCGG
ARAF	TGGTCTACCGACTCATCAAG
ARAF	AGTGTCCAGGATTTGTCCGG
ARAF	GGCTCCATGGAGCCACCACG
ARF1	TGGAAGTAGTGGCGCCACAG
ARF1	AGGCTTCAACGTGGAAACCG
ARF1	TGACAGAGAGCGTGTGAACG
ARF1	CACCCAGCTTAAGCTTGTAG
ARF1	GCTGATGTTCTTGTACTCCA
ARG1	AGATATACAGGGAGTCACCC
ARG1	AGGCCCTACAGTATTGAGAA
ARG1	GGACAGACTAGGAATTGGCA
ARG1	ACTCCACTGACAACCACAAG
ARG1	GCGCCAAGTCCAGAACCATA
ARSA	TGGAAAGGGAACGACCTACG
ARSA	TGCCGACGACCTCGGCTATG
ARSA	TTACGTGAGAGGCATAGTAC
ARSA	ACGGGATGCCTAGAAATCGA
ARSA	GGGAGTCCCCAAATGGCCCG
ARSB	GACCTAGGCTGGAACGACGT
ARSB	TACTGTGTTTCAGATAACGG

ARSB	ATGGCTTCAAGTATTCCTCA
ARSB	GTAGGTATCAAATCCTCGGC
ARSB	GGCAAGCTCGCCGCGCCGCG
ASAH1	TGCGCGACGGCACAGCTGA
ASAH1	CTTACCACCCTACAAAAGA
ASAH1	TTCAGTGCCACGAAAAGGG
ASAH1	ACTCCCCGGACTCCAGCAG
ASAH1	TCGGTCCGACTATTGCCCG
ASAH2	ACATAGGCATGGTATCACAA
ASAH2	TAGCACTTGTCCCATTGGTG
ASAH2	GGGACCATTGAAAACCACAA
ASAH2	CCAGGAGGTAACAGGACCAC
ASAH2	TCTCCTGTGTTGATGCAACG
ASGR2	GTCAACTGGGTGGAGCACCA
ASGR2	CTCCTCGAGCACCCTGACGG
ASGR2	GTGCTTCAGATGGAAGAGCA
ASGR2	GGTGACAAGATCACATCCCT
ASGR2	CCATCTGAAGCACTTCCCCG
ASL	CAAACTGAACTCCAATGATG
ASL	TCCTAATGAGCTCCCAGAGG
ASL	CACAAAGTCCCGCTCACTAG
ASL	CTACGACCGGCACCTTTGGG
ASL	GCATGGATGCCACTAGTGAG
ASNS	TTGTCATAGAGGGCGTGCAG
ASNS	CTCCATATGTATCTCTACCC
ASNS	TTCTAGCAGCCAGTAAATCG
ASNS	AACGTTTGATGACAGACAGA
ASNS	ATTTGAATACCAGACCAAAG
ASPA	GAGGACCAACTTCTATACCT
ASPA	ACAACACCACCTCTAACATG
ASPA	ATTTGCCATATGAAGTGAGA
ASPA	GAGTATTTCTGGTTAAGCAT
ASPA	CTATCTTTGGAGGAACCCAT
ASPH	AACCGAGCATAGTTACCACG
ASPH	TCAAGAGGTATCCCACGCCA
ASPH	AGAAGTAATGAGGTGCTACG
ASPH	TCTACAGGATTATCCTCAGG
ASPH	TCTGACCTCTCCGGGCCCCG
ASS1	TGACCTGATGGAGTACGCAA
ASS1	ACATCCTACCTTTATCTGGG
ASS1	TGGAATCCTGGAGAACCCCA
ASS1	ACACCTCGTGCATCCTCGTG
ASS1	GCCACTGTAGGCCAGAACCA

ATIC	ACACCACTGTCACTCGAGCG
ATIC	AGTTGGTACATCACAAACAT
ATIC	TGAATCTGGTCGCTTCCGGA
ATIC	GCACAAGTTTATAAATCCAG
ATIC	GGATTTCCTGAAATGTTGGG
ATM	GACCTACCTGAATAACACAC
ATM	CCAAGGCTATTCAGTGTGCG
ATM	TCATCACCAAGTTCGCATGT
ATM	TCTACCCCAACAGCGACATG
ATM	TATGGACTCTGAGAACACAA
ATP12A	TGGGTAAACTCAGAGGAGCG
ATP12A	ACATCGCCTCAACATTGCTG
ATP12A	CACCATCATGATCAACGGCG
ATP12A	GTCGCTGACAGCAAAACGGA
ATP12A	GGATAACTCATCTCTCACGG
ATP1A1	GCAACCAGTTATGATTACAA
ATP1A1	TGGTATTGTTGTCTACACTG
ATP1A1	CCTGCCTCTTACCGTGACAG
ATP1A1	ATCCATTCAGGAGTAGTGGG
ATP1A1	ACAAAGCGTCTGGTACCTGA
ATP1A2	GTTCGACAACCAAATCCATG
ATP1A2	CTGAGGCGAGAGTAGCTATG
ATP1A2	TCGGGGCTTCAAATTCGACA
ATP1A2	CATGAGAAGAGATGATCCGG
ATP1A2	GCAGAACTTGACCCACTCAG
ATP1A4	GAGCATCGTACTGTCCGTCG
ATP1A4	CGCCAGGCCTGACGTCAGGG
ATP1A4	AAGGAAATCCTGACTCGAGG
ATP1A4	TGATGAAGGGTGCTCCGGAG
ATP1A4	TGTGGAGTTCACATGCCAAA
ATP1B1	TCCAAGGACTCATTCTTGGG
ATP1B1	CTTGGGATCATTAGGACGAA
ATP1B1	TGTGGGCTTAAATTCACTGA
ATP1B1	CCCAGTGAACCGAAAGAACG
ATP1B1	GTACAAAGATTCAGCCCAGA
ATP1B2	GTTCGTAATAGCGTCCAGGG
ATP1B2	CAGCATCACCCACATGGTGA
ATP1B2	AGGAGTTCGTGTGGAACCCG
ATP1B2	AGTGCTGTAACCATAGTGGG
ATP1B2	TGAGGAACCCATAAAAAACG
ATP1B3	ACTGAATGTATATTCCAATG
ATP1B3	CGACCACCGGAGAATTCCTG
ATP1B3	CCAGGAACCCATAAAAAACT

ATP1B3	GCTTCAGACTCTCAACGATG
ATP1B3	GTAGATGAAGAGCTTCCACT
ATP1B4	GATCATGAGTGAATACCTGT
ATP1B4	AAGTTAAGGCTATGGGCGAA
ATP1B4	TGAATGTAGATTGTCCCCCG
ATP1B4	GACGGTGGTGCCCAAATCGG
ATP1B4	GTAGCGATAACTGTAAGGAA
ATP2A1	TTCAACGACCCCGTCCATGG
ATP2A1	TCACCCGGATCCCGGCGTCA
ATP2A1	GCGGTCGATGACGCCCTCAG
ATP2A1	GGTGGTGAGTGCTGTCTCGG
ATP2A1	TGGGGGCTCCTGGTTCCGCG
ATP2A3	GGGTACCACGTATACCCCCG
ATP2A3	ATGATCACGGGGGATAACAA
ATP2A3	GCCGTGTGGGTCATCAACAT
ATP2A3	CCTACTTACAAAGGAGACAA
ATP2A3	GCACGCCCTTGCGGTCCGAG
ATP2B1	GGTGGATTACCTCGTCACGT
ATP2B1	AGAGGGCTGGAATTACTGTG
ATP2B1	GGCAAAAGTATTCAGACCAA
ATP2B1	ATTGATGAAAGCTCATTGAC
ATP2B1	AGTGAGATCGTGACTGCAAG
ATP2B2	GCAGGAACAGAAATTTACCG
ATP2B2	TGGGCTCCGCACTATCTGCG
ATP2B2	CTGTACCACTGTCATGCGAT
ATP2B2	CCCAACGGACAAGCATACCC
ATP2B2	TCGTGGTCAACAAGAAGCCG
ATP2B3	AACAGCAATGGCGAACTCCG
ATP2B3	CTGGACCACGGTCATACGGT
ATP2B3	TCCCACCGACAAGCACACAC
ATP2B3	TGTCTGGGAATTCACGCCAA
ATP2B3	CCTTCACTCTCCTCTCCTGG
ATP2B4	CGATACAGGTCAGTTCGGTG
ATP2B4	TGTGCGTAATGAAGTGCCCG
ATP2B4	TAGACTGAAAACCTCCCCTG
ATP2B4	CCAGGAGGGAATCGACAATG
ATP2B4	GCAAAGAGAAGCAATTCCGG
ATP2C1	GGAGCTGTCACCTTAGAACA
ATP2C1	CAACAAGAGAAGGCACGCAT
ATP2C1	AGTTGGCTATAATCAATTTG
ATP2C1	GCTTTCACAAATAGTACCAA
ATP2C1	TGATGCCGTCAGTATCACTG
ATP4A	GGGATGGGCCCAACGCACTG

ATP4A	GATCAACGCTGACCAACTGG
ATP4A	GGGGGCTTCACAGACCCTCA
ATP4A	TGGCTATGCCTTCGACGTAG
ATP4A	CGATGTTGCGGGTCTCCAGA
ATR	TGACGTGCGAAAACAAGATG
ATR	GCCCAGGTCACCAATTGTGG
ATR	CTGTGTGAGATGGTCAAGCA
ATR	GATGCTTTGATTTATATGCA
ATR	GTATTCAAGGGAAATCTGAA
ATRX	GAAAATCTCAAAAAACGCGG
ATRX	CTTACCAAGGCCCATACAGT
ATRX	ATGATTTAAAGACTCAGGCG
ATRX	AATTAGTGCGGAATAAGAGT
ATRX	GAGTTCAGTTGATCATCAAG
AURKA	CTTCGAATGACAGTAAGACA
AURKA	CCATATAGAAAATAATCCTG
AURKA	CCTGAAAACTCACCGAAGGT
AURKA	TGCTTGCAAAGGAATGCGCT
AURKA	GCTTGTCTCCAGTCACAAGC
AURKB	ATTCTAGAGTATGCCCCCCG
AURKB	TCTTTCCGGAGGACTCGCTG
AURKB	CATCAACCCATACTGCAGGT
AURKB	TGACGAGCAGCGAACAGCCA
AURKB	GCTCCTTGTAGAGCTCCCCG
AURKC	CTAGGAGGAAGACAATGTGT
AURKC	AGATGAACAGCGCACAGCCA
AURKC	ATTCTGGAATATGCTCCAAG
AURKC	GGAAATAGTTATACAGGCGC
AURKC	CCAAATTTCCCCTTGCCCAG
AVP	GCAGTTCTGGAAGTAGCACG
AVP	GTCGTTGCAGCAAACGCCGA
AVP	CAAGAGGGCCATGTCCGACC
AVP	TCGTCCGCGCAGCAGATGCT
AVP	CTACTTCCAGAACTGCCCGA
AVPR1A	GACAGCCGACCGCTACATCG
AVPR1A	ACCGAGGGACGTGCGCAACG
AVPR1A	TGCATGCGGGACGTCTTGCG
AVPR1A	GACATCACCTACCGCTTCCG
AVPR1A	GCTGGCGGTGACTTTCGCGG
AVPR1B	CAGAACGAAGATAGCCAGGG
AVPR1B	AAACCTAAAAGTCAAGACAC
AVPR1B	GCAGCATCAACACCATCTCA
AVPR1B	AAAAAATGAAGACTTGAGGG

AVPR1B	CAGCACCTGGAAGAGCGCCA
AVPR2	CAGGCGGCGATACCCAGGGT
AVPR2	TGGCCAATGAAGACGTGTAT
AVPR2	ATACTTCACGGCCCGACACA
AVPR2	GGCGTACCGCCATGGAAGTG
AVPR2	AGACGTGTATGGGTGCCCAG
AXL	CTGAGAACATTAGTGCTACG
AXL	CGAAGCCCATAACGCCAAGG
AXL	CCTAGCAGTACATACCACCA
AXL	CCCGAAGCCAATGTACCTCG
AXL	CACCCCTTATCACATCCGCG
AZIN1	ACTTGATGTCCAAATAATTG
AZIN1	GGAAAGTAGATATCCAACAG
AZIN1	AATGGCTTTAGTGCAAGAGT
AZIN1	TGCTCGATGTGTGTTTGACA
AZIN1	CTATGTTTATGAACATACCC
AZIN2	GGGGCGCTACTACGTGACCT
AZIN2	AAATGCGAAGAAGCACCATG
AZIN2	GTCCAAGGCTGAGTTGATCA
AZIN2	ACTTGACAGCATAAAAGGGC
AZIN2	ACTTCTTGTCATTCAGGACG
B4GALT1	CGAGTCCTTACCAAGCAGCG
B4GALT1	TCTATGTTATCAACCAGGTG
B4GALT1	CACGTCACTAAACACAAAGC
B4GALT1	GACCGAGGTCAAGTTGCTAG
B4GALT1	GCCAGCCAGGTAGTAAACGA
BACE1	CCTCATAATACCACTCCCGC
BACE1	GAACTTGTCTGATTCAGTGA
BACE1	TACTACGTGGAGATGACCGT
BACE1	GTGTATGTGCCCTACACCCA
BACE1	ATAATACCACTCCCGCCGGA
BAX	TCGGAAAAAGACCTCTCGGG
BAX	GTTTCATCCAGGATCGAGCA
BAX	AGTAGAAAAGGGCGACAACC
BAX	GGACGAACTGGACAGTAACA
BAX	GGGGGAGTCTGTGTCCACGG
BCAT1	GTTTCAGCCAAACCTCAACA
BCAT1	TTGAAAAATAAGGTCCCACT
BCAT1	CACGGATCATATGCTGACGG
BCAT1	ACCTCAGTTCCAATGAATGT
BCAT1	AGTGCAAAGCTGATGAGCCA
BCAT2	TCGGCAACTACAAGTTAGGT
BCAT2	ACGAACAGGAGCGCGCGCGT

BCAT2	AAATGTCTTCCCAAACACCA
BCAT2	GGTCGGGCCCATACAGCCAG
BCAT2	GTCGGCCAGGAGGGAGACCG
BCHE	AGTGTCAATGAACTATAGGG
BCHE	AGTAAACTTTGGTCCGACCG
BCHE	TTGAATCGAAGTCTACCAAG
BCHE	GATATAAACAGTCTTCACTG
BCHE	GTATTGATATGGATTTATGG
BCL2	TGTCGCAGAGGGGCTACGAG
BCL2	CTGACGCCCTTCACCGCGCG
BCL2	GGCCTTCTTTGAGTTCGGTG
BCL2	TGGACATCTCGGCGAAGTCG
BCL2	GGTCCACCTGACCCTCCGCC
BCL2L1	CAGGCGACGAGTTTGAACTG
BCL2L1	GACCCCAGTTTACCCCATCC
BCL2L1	CAGTGGCTCCATTCACCGCG
BCL2L1	CTCCGATTCAGTCCCTTCTG
BCL2L1	GCACCTGGCAGACAGCCCCG
BCL2L10	GTGAATCTGCCGTAACCTGG
BCL2L10	TGACGCTCGTGACCTTCGCA
BCL2L10	TGTTGCTGGCCGACTACCTG
BCL2L10	CCCTCCTGCTCCTTTAGCCG
BCL2L10	GTAGTCGGCCAGCAACAGCT
BCL2L2	GTGTGCTGAGAGTGTCAACA
BCL2L2	AGCCCAACAACGCTTCACCC
BCL2L2	ACTTTGTAGGTTATAAGCTG
BCL2L2	AGACAAAGAAGGCTACAAGG
BCL2L2	GCCCCCTTGAAAAAGTTCAT
BDH1	CCGTCGGACTTATGCCAGTG
BDH1	CGGCATCTCAACGTTCGGGG
BDH1	CTTTCTCCCCCTCATCCGAA
BDH1	GTACTGCATCACCAAGTTCG
BDH1	GCATAAGTCCGACGGCCAAT
BDKRB2	TGGGTAGCTGATGACACAAG
BDKRB2	CCAGAGCAAATGCCCCCAAG
BDKRB2	AGATAATGGCATTCACCACG
BDKRB2	TGAAAACCATGTCCATGGGC
BDKRB2	GCACAAGAGCAGCTGCACGG
BHMT	GATAGCATCCGGTAAACCTG
BHMT	TCAGGAGTGTGGTAAGCCAA
BHMT	AAGAGGGGCTACGTAAAGGC
BHMT	TGACGTTTGAGCCAGCTCTG
BHMT	AGATTGTGATTGGAGATGGA

BID	TGGGAAGAATAGAGGCAGGT
BID	ACATCATCCGGAATATTGCC
BID	CAACAACGGTTCCAGCCTCA
BID	CAGGAACACCAGCCGGTCGG
BID	GCTCAGGAACACCAGCCGGT
BLK	CAGGTCCCGATCATTCATAG
BLK	GCTGGTCCGACTCTACGCAG
BLK	GCTTCTTGCTCCAATCAACA
BLK	ACTCGGGCCACAAAGTTACT
BLK	ACTAGAATAGTGCTGCACCA
BLM	CGTGCAACAACTTACACCTG
BLM	TCTACGATAAGTGATCTCAA
BLM	CCAACACCACAAATCAGCAA
BLM	GCCTATCAACCCATCAAGGA
BLM	TGACACCTCTGACAGAAAAG
BMPR1A	TAACCTACCTATGACAACAG
BMPR1A	CAACTGGACAGGTTTCATAG
BMPR1A	ATAGCACTTTAAAAAAGGCA
BMPR1A	ATGGCGTGGCGAAAAAGTGG
BMPR1A	TTACTTGTAACAAAAGCAGC
BMPR1B	TCTAACCCAATGCTGTATCG
BMPR1B	TGATGGACCTATACACCACA
BMPR1B	GATTGGAAAAGGTCGCTATG
BMPR1B	CATTGATTTAGCGTCTAGGG
BMPR1B	CTGCCTCCATTGAAAAACAG
BMPR2	GTGACTTTGGACTGTCCATG
BMPR2	CCTTTGGGAGAAATCAAAAG
BMPR2	CAGCACACCTTTGACTATAG
BMPR2	AGCAACTGGACGCTCATCCA
BMPR2	CTTCACACAGAATTACCACG
BMX	GGTTAGAAATTCGAGCCAAG
BMX	GGGAAGACTTCCCTGACTGG
BMX	TGTTGGCCTTTGTTGACACA
BMX	GGGGTTACCCCTTATCTCTG
BMX	TTGAGAACAGGAACTGCCCG
BOC	AATCCAGGTCACGTACACTG
BOC	ACTGGTACCGTCCCACAGTG
BOC	CGTCAAACAAGAGTGGCTGG
BOC	CAGACTCATATGAACTGGTG
BOC	CTATGTGGTGAAACACCGCA
BPI	TCACTGTAAAACTCCCCCTG
BPI	GATGACCCTTAGAGATGACA
BPI	AGAGGCCCAGGTATACCATG

BPI	CTGGAAATAAGGTTGCAGCT
BPI	CTTCAGCTCCTTCTGCAGAG
BRAF	ATACCCAATAGAGTCCGAGG
BRAF	TCATAATTAACACACATCAG
BRAF	ACAAATGATTAAGTTGACAC
BRAF	GGGGGTAGCAGACAAACCTG
BRAF	TCTTCCTGCCCAACAAACAG
BRD4	AGTCGATTTCAATCTCGTCG
BRD4	AGTCGAACTGTCACTGTCCG
BRD4	CCAGACCCCTGTCATGACAG
BRD4	CACCAAACTCCTGAGCATCA
BRD4	CCAACCCTAACAAGCCCAAG
BRSK1	TCACCAACATCATTCCGGGG
BRSK1	CTCTGGACACGCATAATGGG
BRSK1	CCATGCTCTCTAGGACGTCG
BRSK1	TCTCGGGCTTTAGGTCTCTG
BRSK1	AGATTGCCAGAGCCTCCTGA
BTK	TATGAGTATGACTTTGAACG
BTK	GATGGTAGTTAATGAGCTCA
BTK	ATAAGGAGTTACCGTATCCC
BTK	CTGTGTTTGCTAAATCCACA
BTK	GATGCTCTCCAGAATCACTG
C1R	TTCTTCACAGATGAGTCGG
C1R	AAGCAGGACGCCTGCCAGG
C1R	GAGTCCTACAATTTTGAGG
C1R	AGCTTCACCCTGTATCCCG
C1R	ATCAAGAAAGAGATGGAGG
C1RL	CTATAGTCAGCCCATCAGCG
C1RL	TCGGATCCAAGCCAGTTCTG
C1RL	CACCAGTATCCACGGCCGTG
C1RL	GGAGCCCTATTATCAGGCCG
C1RL	ATACGGCTCTGGGTACCCGG
C1S	TCTTCCAAACTGATCTAACA
C1S	GATGACATGAAGAATTGCGG
C1S	CTGTGCGTATGACTCAGTGC
C1S	CACGCACAACTAAACTGTCA
C1S	CATGGAGGAAATATTCCGGG
C3AR1	TACGAGTCTGAGCTACCACA
C3AR1	ACAGACAACCATAATAGATG
C3AR1	AAAGCATCAGAGTTATCCAG
C3AR1	TGGAGGGGATGAGCTTGCAT
C3AR1	CTATCATTCATTTCTCCAGG
C5AR1	CTGGTGCCAGAACTTCCGAG

C5AR1	ACTACAGCCACGACAAACGG
C5AR1	AGTCGGCTACCGCCAAGTTG
C5AR1	CCATTGTACAGCATCACCAC
C5AR1	CCAGTGGTGATGCTGTACAA
CA1	GACACTAATAGGTTTCAGAG
CA1	TGGGGCAGTACAAATGAGCA
CA1	GAGGACAACGATAACCGATC
CA1	TGGAGTCAAATATTCTGCCG
CA1	GCATGGTTCAGAACATACAG
CA11	GCAGCTCATTCACTTCAACC
CA11	GCCACCGACTCAGTGAACTG
CA11	CCAGTGCTGAGCCTTAATGG
CA11	TGTAGCTCCACCAGTCCTCG
CA11	TCTTCAGCTCCACATCCACG
CA12	GGGAACCCGAATGACCCGCA
CA12	TTGTAGCCTTGGAACTCGAG
CA12	TGCAGCAGGCCCCCACACGA
CA12	TGGCACTGTAGCGAGACTGG
CA12	GTTCCTTTAAGATCACCAGC
CA13	TGTTGTTGTTTAGTTCTGCG
CA13	TGGGGGTCCGCTGATGACCA
CA13	GAGCTGCCTCAACAAAGCTG
CA13	TGGAGTGAGCTATGCTGCAG
CA13	GCTAAAATCATCAGCAACAG
CA14	CCAGGCTGGTCATATCCGTG
CA14	TTCCTATGACAGCTTGAGTG
CA14	CTGGTGTTCTGACCCCCCTG
CA14	AGCTCTCTTAGGTTGAAGGG
CA14	GGGAAGTCCACCCAGATACA
CA2	CAATGGTCATGCTTTCAACG
CA2	TATGAGTGTCGATGTCAACA
CA2	TGATCATAGGAAACAGACAG
CA2	TCACTGGAACACCAAATATG
CA2	ATGAGTGTCGATGTCAACAG
CA4	AGAGTCACACTGGTGCTACG
CA4	CTCAGAGATGAGCACTACGA
CA4	GCTACGATAAGAAGCAAACG
CA4	CGAGCCCTTATATGGCAAGT
CA4	ACTGGCCGATGGCCGCGCCG
CA5A	AGACCGGGACCGTCCAGAGT
CA5A	CGGCCACGCGTACCCCGCAG
CA5A	GGAATTTGACGATGCCACCG
CA5A	AGTCTCCTATTAACATCCAG

CA5A	GTCCCGGTCTCCGTGCCAGG
CA7	TGGGGCAAGAAGCACGATGT
CA7	ATACAGCTTGTGCCAATGCG
CA7	TGAAACTGCTTGAGGCGGTA
CA7	GCCTCATAGGAAAGCTCCAG
CA7	TGTGGGTTCTGAGCACACGG
CA8	GCAATGATGGCGATTCCGTG
CA8	TCGGCACACCACATAATTTG
CA8	TATTAACCTAAACTCAAGAG
CA8	GTACGAAGTGAGATTTCACT
CA8	TTCCTTCTCGGGGAAGGCGA
CA9	CTGGGGGCGGATATCCACCG
CA9	ATCTGGTACCTCCAGAAAGG
CA9	GGGAGACCCCCTCACCACTG
CA9	TCCGGGCTCGGAGCACACTG
CA9	GGAAGCCCAGGAGTTCCAGG
CACNA1A	TCTCACCTTGTACGACGGTG
CACNA1A	CGTCAGTTTCATCCTCGGCG
CACNA1A	GTTTGACCTACGGACGCTGA
CACNA1A	GCGCTCCAGCCACGTACGAG
CACNA1A	AGAAGAGGTGATCCTCGCCG
CACNA1B	AGTCGTTCCAGTATAAGACG
CACNA1B	TATCACGGGATCGAATCGCA
CACNA1B	ATACTCACTGGGAACGACCG
CACNA1B	GTGCCACCTACCGTCCAAAG
CACNA1B	GTACAGCGAGATGGACCCCG
CACNA1C	TGTTGATATAGCAATCACCG
CACNA1C	CATTAGACTTGACTGCGGCG
CACNA1C	AGATCGTGTCATTGACAATG
CACNA1C	GATGGGGCAAACGCTCTCGG
CACNA1C	TGTCATTCAGATCCTGACCG
CACNA1D	AATCGGCAGCATTATAGACG
CACNA1D	ACTCCTGACACTAGTCGAAG
CACNA1D	GACTCGAATGGAGAGAACAT
CACNA1D	TGCTTGAGGGAAATTGTCAA
CACNA1D	ACAGCGATAGAACTTCCCCT
CACNA1E	GTCCACGTGAGTATTGAAGT
CACNA1E	CCACGGTTGAGTCCACCAAG
CACNA1E	AAGACACCACATGTCGATGT
CACNA1E	AAGGTATCAAAATTTGCCGA
CACNA1E	GTAGGCGGCCGCCTGCCCCG
CACNA1F	TGGTATGCAGCGTCAATGTG
CACNA1F	CCAGGGCAGTTCATACCCCA

CACNA1F	GCCCCTCCGAGCCATCAACA
CACNA1F	TGGAATTGAGCACTATGTGC
CACNA1F	CATTGGACTTCACTGCCCGA
CACNA1G	TCATAGTCCAGACCGCAAGG
CACNA1G	CTGACCAGGAGTCTCGCTCG
CACNA1G	CAATAAATCGGACTGTGCCG
CACNA1G	GGGATGCCAATGGGTCCCGC
CACNA1G	CTATTACCAGACAGAGAACG
CACNA1H	TGGCGTCTATGAATTCACGC
CACNA1H	TGTACTTGCGTCGCACCCAG
CACNA1H	CGATGCCAACAGATCCGACA
CACNA1H	GCGGCCGTACTACCAGACGG
CACNA1H	GCACGGTCCTGATAGCCGAG
CACNA1I	GGGCAATAATGGTCTGACAC
CACNA1I	TGTATTTGTGATGGACCCAG
CACNA1I	TGGCAAGAAGTGCTACCTCG
CACNA1I	ACGGAGCCTCCTCAGAACTG
CACNA1I	TGACGATGAAGAAATCCAGG
CACNA1S	GGAAGTCAAACACATTCCAG
CACNA1S	CTGGTGGAATAAGAAGCCGT
CACNA1S	AGAGCTGCGTCACCGCGAGT
CACNA1S	CCAGGGCCACTCATTCCCGA
CACNA1S	GAGCCATCAACAGAGCCAAG
CACNA2D1	ATAATATCGAGCTAGGCCAG
CACNA2D1	TTTCATCTTACTGTAAAACG
CACNA2D1	AGTTGTCTACTACAATGCAA
CACNA2D1	GCTATACATTCATAGCACCA
CACNA2D1	CGACTTACGTGACGGCCGAA
CACNA2D2	GGTGACACGGAACTACACCT
CACNA2D2	TCACTCGCTACTACCCGGGT
CACNA2D2	CATCCCTAGAGAGTACTGCA
CACNA2D2	CAGTGCATCCTCATACACGT
CACNA2D2	GTACAGGTCGATCTTCTTGG
CACNB1	AGACCCCCTAGAGTTAGAGG
CACNB1	ACCGTCGCTCAAGGGCTACG
CACNB1	AAGGGGATGCGTTTGCCATG
CACNB1	GCTGTTGGATGTGGTATCCG
CACNB1	GCATGTGCCCCCCTATGACG
CACNB2	TAGAGAACGTGGCTCCCGCG
CACNB2	GACAAATGTCAGCTACAGTG
CACNB2	ATACATCGGAATCGGATGGA
CACNB2	ATAGGGCGATTGGTAAAAGA
CACNB2	ATGAACAGAGAGCCAAGCAA

CACNB3	ACATGTTCCCCCATATGACG
CACNB3	GTCCAACACTACTAGCTGCA
CACNB3	TGCTCAACAATCCGGGCAAG
CACNB3	CCTGGCTCTCTACTTCACGC
CACNB3	GTAGGAGTCGTCATACATGG
CACNB4	TGTACCGTCAATGCGTCCGG
CACNB4	CAGCGGTTGATTAAATCTAG
CACNB4	GGACCGGGAAGCAATTCGAC
CACNB4	TAATTGAACGTTCGAACACC
CACNB4	CTACGCCAAGAACGGGACCG
CACNG1	GCTTGGTACAAATCCGCCAG
CACNG1	AAAGGCATAGAACATGGACG
CACNG1	TTCGAATTCACCACTCAGAA
CACNG1	AGCCGTGGTAACCGACCACT
CACNG1	AGATCTCCGAGCTCTCGCCG
CALCA	AGACCCGGCCACGCTCAGTG
CALCA	ACCTGAATGGTGCTGCATGG
CALCA	GAGAGAGAGGGCTCCAGGTG
CALCA	GATGCTGAGAGCCAGGAAGG
CALCA	AGATGCTGAGAGCCAGGAAG
CALCR	CAGCAGTTACCCGCATACCA
CALCR	TTACTGAGAAGCAACGCTTG
CALCR	GCACATAGTATAGTTGGACC
CALCR	CATAATCCATGGACCTGTCA
CALCR	GAAAAACACGAAAATCCCCA
CALCRL	GTGGGTGTTAACATTACACT
CALCRL	AGAATTAGAAGAGAGTCCTG
CALCRL	TTACCTACACACACTCATTG
CALCRL	CGTTTACTGCAACAGAACCT
CALCRL	TTAGGAGCCTAAGTTGCCAA
CALR	TAATCCCCCACTTAGACGGG
CALR	CATGAGCAGAACATCGACTG
CALR	CCTCGGGCTTCTTAGCATCA
CALR	GGCCACAGATGTCGGGACCT
CALR	GTGTTTGGATTCGATCCAGC
CAMK1	TGTGGAACTCCGGGATACGT
CAMK1	TGTGGAAAAAGGCTTCTACA
CAMK1	GATCCCGGTGTACAATGCCC
CAMK1	CATCCAGGGCTACAATGTTG
CAMK1	ACTCTCATAGATGTCATCCA
CAMK1D	GGTCCCCACTTACGTTCCGA
CAMK1D	GATGTAGGCAATCACTCCGA
CAMK1D	TATGGGAGACCTACCAGAGT

CAMK1D	TTTGGATTGTCAAAAATGGA
CAMK1D	GCTTTCATAAATGTCTTCCA
CAMK1G	ACTTTGCTAAGAGCAAGTGG
CAMK1G	TTGAGAAGGATCCGAACGAG
CAMK1G	GGACGATGCCATTCTCATGT
CAMK1G	AGCGTCTACTCACAATATGT
CAMK1G	GCTCTCATAGATGTCCTCCA
CAMK2A	GGCCCGGGAGTATTACAGTG
CAMK2A	GTGCTGCGGAAGGACCCGTA
CAMK2A	CCTCCAGCACCGCTCCACCG
CAMK2A	GACACTCACATCATAGGCGC
CAMK2A	ACTACATGACAGCATCTCAG
CAMK4	AGATACTTCATCCCACCAGG
CAMK4	GTCCCGGATTACTGGATCGA
CAMK4	GAGCATACCGCAGTACCCTG
CAMK4	TGCCGTTAAACAAATCCTGG
CAMK4	GCAGCACCTGAAATTCTTAG
CAMKK1	TCAGTTTGACCACATTCACG
CAMKK1	GGCCAGCCTCACCACACCGT
CAMKK1	AGAGCCTACTAGAAACGGTG
CAMKK1	AGGCTGGGCCTTATGCCACG
CAMKK1	AGTACTGCCAGGGATCACAG
CAMKK2	TGGAAGGTTTGATGTCACGG
CAMKK2	ACGTGGTGAGACTCCACTGT
CAMKK2	GAGACAGCTTGCGACCGGAG
CAMKK2	TGTTCGAACTGGTCAACCAA
CAMKK2	TCAGGTCGCCCTCCACCCCG
CAMKV	AAGAGGCCATCTCCCATGAG
CAMKV	TGGCAGCTTTCCGCACCTTG
CAMKV	GATCGTGCACAGGAATCTCA
CAMKV	AGATACTCGGGGGTCCCACA
CAMKV	GAGTGAGTGCAAATAGGCCA
CAMLG	ACAGCGGACTCGGTCCAGAG
CAMLG	TTGAAACGGAAGGAACGCTG
CAMLG	CAGTTGGGTCCCCTTTACCT
CAMLG	TTGACATCACTACTGCACTC
CAMLG	GGAACAACTGACCAGCAGGG
CANX	ACCAAACATAATCGTATAAG
CANX	TCCAGACGCAGAGAAACCTG
CANX	TTACTGAACAATGAGAGGCT
CANX	TTGTCTTGTAGGAAAGTGGG
CANX	ACAAAGCTCCAGTTCCAACA
CAPN1	CTCCTCAGAGTGGAACAACG

CAPN1	TCACAGGCGGGGTTACCGAG
CAPN1	CTCCAGCGTTCTAGACATGG
CAPN1	TGGAACACCACACTCTACGA
CAPN1	CATCAAGTGGAAGCGTCCCA
CAPNS1	TGACACATGTCGCAGCATGG
CAPNS1	GACTCCGCCTAGGATGCGCA
CAPNS1	GTTGGAGTAATGTGTGCGTG
CAPNS1	GCTTGGAGGCCTGATCAGCG
CAPNS1	GTTAACTCGTTCTTGAAGGG
CARM1	TGGAGCACGGAAAATCTACG
CARM1	TAGAGCTGTTCATCCGTGAA
CARM1	TCGCGTCGCCGATGGTGAGG
CARM1	TTGAAGAGCATGTAGCCCAT
CARM1	AAGAAGTACCTGAAGCCCAG
CARS	CTTCGATGGACATTCACGG
CARS	CACTGGTCAGTTTGAGAAG
CARS	AATGAGCTGGCACAGTCGG
CARS	CCCGGGTTAAGACATCTGG
CARS	CAGAAAGCCCTTCAAGAAG
CARS2	GTTACCCTCAGGTACACCG
CARS2	CTCCCGGGTCCCCAGGGAG
CARS2	GTACAACAGCCTCACCGGG
CARS2	TTTGCGCTGGCCATGCCCG
CARS2	GACATGTTGAGGACTACGCG
CARTPT	TGGGCACGGGTACCCAACAG
CARTPT	ACCGACCAGCTCCTTCTCGT
CARTPT	CCTGGACATCTACTCTGCCG
CARTPT	GAGTAGATGTCCAGGGCTCG
CARTPT	AGGTAGCATCAGCAGCAGGG
CASP1	TACCATGAGACATGAACACC
CASP1	ATGGAAACAAAAGTCGGCAG
CASP1	CTTAATATGCAAGACTCTCA
CASP1	ACAGACAAGGGTGCTGAACA
CASP1	ATAAAAACAGAGCCCATTGT
CASP3	ATTGTGGAATTGATGCGTGA
CASP3	GGAAGCGAATCAATGGACTC
CASP3	GTCCAGTTCTGTACCACGGC
CASP3	CAAGGAATGACATCTCGGTC
CASP3	AGTTTCTGAATGTTTCCCTG
CASP6	GTTGGACACCAACATAACTG
CASP6	CTTGTCTTGTAGGCATGTCG
CASP6	GTAGATAAAACTACTACCTG
CASP6	TGCTCAAAATTCATGAGGGT

CASP6	GTGTGTGTCTTCCTGAGCCA
CASP7	GAAGAGGGACGGTACAAACG
CASP7	TGTACTGATATGTAGGCACT
CASP7	TTTGATATTTAGGCTTGCCG
CASP7	TTTGACAGCCCACTTTAGGG
CASP7	AGGGCTGTATTGAAGAGCAG
CASP8	CTACCTAAACACTAGAAAGG
CASP8	TCTACTGTGCAGTCATCGTG
CASP8	AGGGGACTCGGAGACTGCGA
CASP8	GCCTGGACTACATTCCGCAA
CASP8	GGAACTTCAGACACCAGGCA
CASP9	CTCTGGTCTGAGCACCACTG
CASP9	CAATCTTCTCGACCGACACA
CASP9	ACACCCAGACCAGTGGACAT
CASP9	TGGATGTCCTCGATCATATG
CASP9	TCATAGATCTGGAGACTCGA
CASR	CATGCCTCAGTACTTCCACG
CASR	AAACTTTACAAACAATATGG
CASR	TTGAGCAACAAAACTCAGGG
CASR	GAGGGTGAGTGCGATCCCAA
CASR	GGGGATTGAGAAATTCCGAG
CAT	CTGGATGTAAAAAGTCCAGG
CAT	ACATCTGAAGGATCCGGACA
CAT	TTATTACAGTAGGGCCCCGT
CAT	GTGGAGAACCGAACTGCGAT
CAT	ATTTCACTGCAAACCCACGA
CATSPER1	AACTGGAATGATACGCGCCG
CATSPER1	GATGGTGCAACTCGTAATGG
CATSPER1	TATCACGTAGCACACCCACG
CATSPER1	GATAGTCAGATATCCCACGC
CATSPER1	GGAAGTCTTGGAACTCCGGA
CBL	GTAGATCCGTTTGATCCTAG
CBL	GCAGGTCTAAGATATAAGGT
CBL	GGTGGAAGATCTCGAAGTGT
CBL	AGACCATATCAAAGTGACCC
CBL	TCATCATCATAATTTGGGGA
CBR3	ACTGTGCCGACAGTTCTCTG
CBR3	CAACGTACTGGTCAACAACG
CBR3	CTGCCGATAATGAAACCTCA
CBR3	GCAAGTCGTCGATGTCCAGT
CBR3	GCACCTACTCTTGAAGGCGA
CBS	CAAGTGTGAGTTCTTCAACG
CBS	TCATTGGGGTGGATCCCGAA

CBS	AGATATTCTGAAGAAAATCG
CBS	ACTCCCCGGAGTCACACGTG
CBS	GGTGGTGTCGTAGTGAGCCA
CCK	AGGGTATCGCAGAGAACGGA
CCK	ACGATGGACATTCGTCCAGA
CCK	GCTGGCAAGATACATCCAGC
CCK	GATACCCTCAGCTGCCTACG
CCK	GCTGAGGGTATCGCAGAGAA
CCKAR	GCACCAAGTTGCTATAAATG
CCKAR	ATCTCTTTGGAACTCTACCA
CCKAR	TGCAAAAGACCAGGCCCCCG
CCKAR	CCGTGCTGATTCGGAACAAG
CCKAR	GTAGGTGGTGGTCTTGCAAA
CCKBR	CTCGCGAGAGATAAGCCCGT
CCKBR	CCGGCTCCGCGAATGCGAGG
CCKBR	TCGTGGCCATCGCACTGGAG
CCKBR	CTCGCGTGATTGTAGCCACG
CCKBR	CTACAATCACGCGAGCCGCG
CCL11	AGCTACAGGAGAATCACCAG
CCL11	TAGCTCTCTAGTCGCTGAAG
CCL11	GGGGTATCTTCCTATTGGCC
CCL11	CAGCAGCCACAGAAGTGCTG
CCL11	GTAGCTCTCTAGTCGCTGAA
CCL2	CAGCCACCTTCATTCCCCAA
CCL2	ATTGGTGAAGTTATAACAGC
CCL2	CCACAATGGTCTTGAAGCTG
CCL2	ACTGGGGCATTGATTGCATC
CCL2	GCTGTTATAACTTCACCAAT
CCL20	TACCTTCTGATTCGCCGCAG
CCL20	AGCAGCCAGGAGCAAACTCT
CCL20	ACACGGCAGCTGGCCAATGA
CCL20	CTTCATCCTAAATTTATTGT
CCL20	GTGCTGCTACTCCACCTCTG
CCL5	AAGGAGTATTTCTACACCAG
CCL5	GTAGAAATACTCCTTGATGT
CCL5	TCAAGACCAGGACTTACATG
CCL5	GCAATGTAGGCAAAGCAGCA
CCL5	AGGTACCATGAAGGTCTCCG
CCL7	ATAAGAAAATCCCTAAGCAG
CCL7	ACATACATTACAGCTTCCCG
CCL7	CCGGGGACAGTGGCTACTGG
CCL7	ATTGATAAATCTGTAGCAGC
CCL7	GCAGCTGCTTTCAGCCCCCA

CCNB1	CATCAGAGAAAGCCTGACAC
CCNB1	GTCAGACCAAAATACCTACT
CCNB1	GAGGCCAAGAACAGCTCTTG
CCNB1	ATATTTGCTTGCAATAAACA
CCNB1	CATGGCGCTCCGAGTCACCA
CCND1	GTGTTCAATGAAATCGTGCG
CCND1	GGTTGGCATCGGGGTACGCG
CCND1	CGTGCCTCCGTAGGTCTGCG
CCND1	AGAGGCCACGAACATGCAAG
CCND1	GGTGGCGACGATCTTCCGCA
CCR1	AAATACCAAGGAGTACAGAG
CCR1	AACTTGTAGTCGATCCAGAA
CCR1	GAAACAGCTTCCACTCTCGT
CCR1	CTGTGTAATAAAACCCAGAG
CCR1	CATGGAAGCCAAGATGGCCA
CCR2	TGGAAATTATTCCATCCTCG
CCR2	GACAAGTGTGATCACCTGGT
CCR2	ATTTGACGTGAAGCAAATTG
CCR2	TTCACAGGGCTGTATCACAT
CCR2	GTAATCATAATCAAAAAAGG
CCR3	ACATCCTACTATGATGACGT
CCR3	TCTGGATCCACTATGTCAGG
CCR3	GACACCAAAAGTGACAGTCC
CCR3	TTGCAGTGCTCTTTACCCAG
CCR3	TGATCCTCATAAAATACAGG
CCR4	ACTCTCTCAACTCCACGACG
CCR4	AAAAACCAAGGAATACAGTG
CCR4	ATACCTGGCAATTGTGCACG
CCR4	AAAACAGCATGATCCCTAAG
CCR4	ATAGTAGCCCCAAAAAGGGA
CCR5	CATTAAAGATAGTCATCTTG
CCR5	GGTGACAAGTGTGATCACTT
CCR5	CAATGTGTCAACTCTTGACA
CCR5	TCATCCTCCTGACAATCGAT
CCR5	AACACCAGTGAGTAGAGCGG
CCR7	GAGCAGGTAGGTATCGGTCA
CCR7	ACGCAACTTTGAGCGCAACA
CCR7	TAGTATCCAGATGCCCACAC
CCR7	AGCTGAGACAGCCTGGACGA
CCR7	GCTTGCTGATGAGAAGGACG
CCR8	TGGGACTGTAATGTGCAAAG
CCR8	AAGGTGAGGACGATCAGGAT
CCR8	GGTACCTGTCCACACTCATG

CCR8	CTTCTCAAGCCCCTGTGATG
CCR8	GTACCTGTCCACACTCATGA
CD163	CAAAGACGATGAATTGCACG
CD163	GTGAAGCATGGTGACACGTG
CD163	ACTGGCGTTAACTCGACCAA
CD163	TCTGTGATTTGTAACCAGCT
CD163	AGTGGAAGTGAAAGTCCAGG
CD1D	TTATCGAAGCAGCTTCACCA
CD1D	GGCCAAGTTTACCCAAAGTG
CD1D	CCGACTTCCCTGACTCAAGG
CD1D	GCACAGCTGGAGCAACGACT
CD1D	AGTTATTTGAGGCGTTCCCA
CD2	CTTGTAGATATCCTGATCAT
CD2	CTTGATACAGGTTTAATTCG
CD2	AGAGGGTCATCACACACAAG
CD2	GTGCCACAAAGACCATCAAG
CD2	CTGACCTGTGAGGTAATGAA
CD22	ATTCATACCGGGTAACACTG
CD22	GAAGTGACCAAGGACCAGAG
CD22	GCTGCACCGTGTCATTGGAG
CD22	GGTATCCGATCCAATTGCAG
CD22	GGGACTCTTGAATTTCTGGA
CD248	CTGCGAACACGAATGTGTGG
CD248	ATGTGTGTCAACTACGTTGG
CD248	CGGCTTCACGTGGACCACAG
CD248	GAAGCTCGGTCTATAGGCCA
CD248	ACAGCGGCCACAGAGCCGAA
CD3E	GATGGAGACTTTATATGCTG
CD3E	GATGTCCACTATGACAATTG
CD3E	CAACACAATGATAAAAACAT
CD3E	TGAGGATCACCTGTCACTGA
CD3E	TATTATGTCTGCTACCCCAG
CD40LG	ACGATACAGAGATGCAACAC
CD40LG	GAGCAACAACTTGGTAACCC
CD40LG	TGCGGCACATGTCATAAGTG
CD40LG	AAGTGCTGACCCAATCATCT
CD40LG	GCTTTGAAATGCAAAAAGGT
CD44	CATCACGGTTAACAATAGCT
CD44	AAGACTCCCATTCGACAACA
CD44	TGCTACTTCAGACAACCACA
CD44	TCGCTACAGCATCTCTCGGA
CD44	CGTGGAATACACCTGCAAAG
CD5	CAGCATCTGTGAAGGCACCG

CD5	CGGCTCAGCTGGTATGACCC
CD5	AAGCGTCAAAAGTCTGCCAG
CD5	TTTCCTGAAGCAATGCTCCA
CD5	GGCGTGGTGGAGTTCTACAG
CD59	ACGACGTCACAACCCGCTTG
CD59	AAGGAGGGTCTGTCCTGTTC
CD59	AAAATCAGATGAACAATTGA
CD59	CTGAATGGCAGAAGACAGCC
CD59	TCACAATGGGAATCCAAGGA
CD80	TGACGTTATCAGTCAAAGGT
CD80	AGGTGTTATCCACGTGACCA
CD80	CGTATGTGCCCTCGTCAGAT
CD80	AGGCTCTGGAAAACCTCCAG
CD80	GCTCTGCGCCCATCTGACGA
CD86	TGTCCGAATCAAAACTTGTG
CD86	AAAATACTACTAGCTCACTC
CD86	ACAGTTCAGAATTCATCTGG
CD86	TTGACCTGCTCATCTATACA
CD86	GTTCTTACCAGAGAGCAGGA
CDC25A	TTTGTAGTTCTCATGACGAG
CDC25A	GTAAAGATCTCTTCACACAG
CDC25A	TGATTATGAGCAACCACTGG
CDC25A	CATGGTCAAGAGAATCAGAA
CDC25A	AAAGAGATAGCAGTGAACCA
CDC25B	TGGATGTACCATCAGGTCGG
CDC25B	GGCACTTGCTGTACATGACG
CDC25B	CGCCCGTGCAGAATAAGCGG
CDC25B	TGGTGATGTTCCGAAGCACG
CDC25B	ACAGGGATAGGTGCGTCAGG
CDC25C	TGCTAAGATTCGAAAGATCG
CDC25C	CAAGTTCTCTGGCATCGACG
CDC25C	GATGTCCCTAGAACTCCAGT
CDC25C	CTGGAGGAAGATTCTAACCA
CDC25C	ACTCTTCTCATCCACAAGAG
CDC42	ACAGTCGGTACATATTCCGA
CDC42	AGAAAGGAGTCTTTGGACAG
CDC42	GCAGTCACAGTTATGATTGG
CDC42	AAGTGTGTTGTTGTGGGCGA
CDC42	TCTGTTTGTGGATAACTCAG
CDC42BPA	TTCTGAGGATCTATACCCAG
CDC42BPA	TGTAGCAGAGAACATCGACT
CDC42BPA	CAACATAATAATCCATAACC
CDC42BPA	AAAACTGTAGGAACTAGTCC

CDC42BPA	GGAAATGCTGAAAAGAGCTG
CDC42BPB	GATATGGCGAGGTTCTACAT
CDC42BPB	TGCAGATGGAAGCTTACGAG
CDC42BPB	AGAGGAATTGGTCAGACGTG
CDC42BPB	CTGGGGTCAAGAACACTGGT
CDC42BPB	TGACACATCCAACTTCGACG
CDC7	TTAAACACATTACTAAGCTG
CDC7	TAGGGGGCAAGATAATGTCA
CDC7	GGCCAAACCAAAGTCTACCA
CDC7	TCCTGGTGTACCTGCCCTAG
CDC7	TTAGGTACTGGGCCACTCAG
CDCP2	AATGGGGCCTCACCAGACAA
CDCP2	CACGAACACCAGCTTGACGT
CDCP2	GAGGTATACATGGCCATGCG
CDCP2	GTTGTTGGGATACTCAGGAC
CDCP2	ATGGAAGGTGAGCAGCACCG
CDK1	GACAAAACACAATCCCCTGT
CDK1	GTATTCCAAAAGCTCTGGCA
CDK1	ACCCTTATACACAACTCCAT
CDK1	GATCTCCAGAAGTATTGCTG
CDK1	AATCAGACTAGAAAGTGAAG
CDK10	CCTGCGTCATCCGAACATCG
CDK10	TATGCCAACACCCTTCTCGG
CDK10	GAAGCTGAACCGCATTGGAG
CDK10	ACAGGAACTTCATTATCCAC
CDK10	GTGTTGGCATATTCTCCAGG
CDK12	ATTCACCAGTTCAGTATCTG
CDK12	ACTGACCGACTGCCTTCTCG
CDK12	CTAGCAGTCCCATTAAGTCA
CDK12	TGGCCTTCAAACTAGACCGA
CDK12	GCTTGTGCTTCGATACCAAG
CDK13	AGGTAACGGTGGTAATGTAG
CDK13	TAGTCGGCCGTATACTAACA
CDK13	GGTTCCTTGTAGGCCGAAGG
CDK13	AGAATATGTGGGCCTCGCTA
CDK13	ACACTTCTACACCTACCAAG
CDK14	CCAACGAAGTGGTTACCTTG
CDK14	ACCTTCTGATCAGTGACACG
CDK14	AAGAGTCACCTAAAGTTAGG
CDK14	GTGTCACAAAGATGTCTACA
CDK14	GTACATGGACAAGCACCCTG
CDK17	ACATAGACGGATCTCAATGG
CDK17	CTTTCAAGAAGCCCCCATTG

CDK17	CATGAGTATGCACAACGTAA
CDK17	CCCTGCACAGCTATAAGAGA
CDK17	GCAGACATCAGAATACCTGA
CDK2	CATGGGTGTAAGTACGAACA
CDK2	CAAATATTATTCCACAGCTG
CDK2	TCTGAGGTTTAAGGTCTCGG
CDK2	AAGCAGAGAGATCTCTCGGA
CDK2	CTTCATGGAGAACTTCCAAA
CDK4	CCAGATGGCACTTACACCCG
CDK4	AGTGTGAGAGTCCCCAATGG
CDK4	GTCCACATATGCAACACCTG
CDK4	GTCTACATGCTCAAACACCA
CDK4	CCAGTGGCTGAAATTGGTGT
CDK5	TAGCCGCAATGTGCTACACA
CDK5	CCTTTACAATCTCAGGATCG
CDK5	CGTCCGCTGTTACTCAGCTG
CDK5	CCGGGAGACTCATGAGATCG
CDK5	GAGTAGGCAGATCTCCCGGA
CDK6	GCCCGCGACTTGAAGAACGG
CDK6	AACACTCCAGAGATCCACGG
CDK6	TGGCTCACCTGACCACGTTG
CDK6	CATTGCAGGTCGTCACGCTG
CDK6	GCCGCTCTCCACCATCCGCG
CDK7	AGCTCCAAATAGTAACTCGG
CDK7	ATCTCTGGCCTTGTAAACGG
CDK7	TTTCCATAAAATCAAAGACA
CDK7	TTAAAAACCTTACCCTATGT
CDK7	TGAGAAGCTGGACTTCCTTG
CDK8	CTCTCACTTTCTTCAACGG
CDK8	TCTGATGTGAGTACTGTGG
CDK8	AGCGAGGAAGAGTAAAAAT
CDK8	GCTCCAAGAAGTAGTTCAG
CDK8	GAGGGCTGCAAAGTTGGCCG
CDK9	CCAGAGTGTCACCACACGGT
CDK9	TCTCCCGCAAGGCTGTAATG
CDK9	GCGGTTATAGGGGGAAGCTG
CDK9	GCTGACTGATGAGGGCGAGT
CDK9	GGTATATACTACCCTTGCAG
CDKN1A	CCATTAGCGCATCACAGTCG
CDKN1A	TCAGAACCCATGCGGCAGCA
CDKN1A	AGTCGAAGTTCCATCGCTCA
CDKN1A	GTCACCGAGACACCACTGGA
CDKN1A	GATGTCCGTCAGAACCCATG

CDKN1B	AGTTCTACTACAGACCCCCG
CDKN1B	TGGACCACGAAGAGTTAACC
CDKN1B	AATCGAAATTCCACTTGCGC
CDKN1B	GGGCAAGTACGAGTGGCAAG
CDKN1B	TCAAACGTGCGAGTGTCTAA
CEL	CACACGCGGAAACGTCATCG
CEL	CAGGCAGTCTTCATCCCCGT
CEL	CAGACACTGGGCCATCCTGG
CEL	GAATATCGCGGCCTTCGGGG
CEL	GTGAGTTCACAATCACCAAG
CELA2A	CCACCCTAGTCACATAAGGT
CELA2A	AGTACAGCTCCAATGGCAAG
CELA2A	CTGATCTCATTCAGCCAACG
CELA2A	CCGGCACAACCTCTACGTTG
CELA2A	ACTCCAACCAAATCTCCAAA
CELA3B	GGAAGCTTCTACCACACCTG
CELA3B	CATCCTCACCATTGACAACG
CELA3B	CCAGCTCGCCTCACTCCCTC
CELA3B	GAGCTCCCGGACCTACCAGG
CELA3B	TCCAGAGTGGATGCACAAAG
CES1	ATGCCGCAGCTTACATAGGA
CES1	ATCTTTGGAGAGTCAGCGGG
CES1	TTCTCTTCCTCACAGCACAG
CES1	TGAGCTATCCACTCTCCGAA
CES1	GAAGGACTCACCCCAAGCCG
CFB	TATGACGGTTACACTCTCCG
CFB	CATGTACGACACCCCTCAAG
CFB	TCTGCAGGATTGCACAACAT
CFB	TGTCTGATCCATCTAGCACC
CFB	GAGGGGGTAGAGATCAAAGG
CFD	ACCGGGAACTCTCTGCGACG
CFD	CCCCGTGGTCGGATCCTGGG
CFD	ACGTCGTACAGGCGCTTGGA
CFD	ATGGCGTCGGTGCAGCTGAA
CFD	GAGAACCTGCACCTTCCCGT
CFI	TGAGGTGGACTGCATTACAG
CFI	AGAAGACTTTATCGCAGGAG
CFI	TGTCTACATGTGCATTGCCG
CFI	GATGCCAGTGGAATCACCTG
CFI	GGTCACTTATACATCTCAAG
CFTR	TGTGGACAGTAATATATCGA
CFTR	ATTCTTCAGAGGTCTACCAC
CFTR	CCACGCTTCAGGCACGAAGG

CFTR	GCTATTGAAGTATCTCACAT
CFTR	CTATGACCCGGATAACAAGG
CHAT	AGCAAAAACTCCCAGCAGTG
CHAT	GTGCTGCCGAGCAAAGATCA
CHAT	GGAGCTCAGCGACACCCACA
CHAT	ACATCCAAGACAAAGAACTG
CHAT	GTATGCCTGGACGCGCCAGG
CHD1	TTAATTCGCCTAAGAGAACG
CHD1	TTCCGATGACTCATCAAGTG
CHD1	AAGCAGCCATCCTATATTGG
CHD1	ATGCCCAATTTAGACCTCCA
CHD1	GGACGCATCATCAGACCAAA
CHEK1	ACACCACCTGAAGTGACTCG
CHEK1	TGGTATTGGAATAACTCACA
CHEK1	CTTACTGCAATGCTCGCTGG
CHEK1	TTTCTGGAGTACTGTAGTGG
CHEK1	CTTCCATCAACTCATGGCAG
CHEK2	GGGCCCATAATCGAGCCCAG
CHEK2	AGGTAAAGCTGGCTTTCGAG
CHEK2	GCATACATAGAAGATCACAG
CHEK2	AGAGCTGTTTGACAAAGTGG
CHEK2	GTGTAGTACCTTCATGAAAA
CHKA	CATAACGCTCTCCAGAACCA
CHKA	CCGGGATGAACTGCTCCAGT
CHKA	CTTGGACTCGAGGTCGCTGG
CHKA	TTTCCGAGGCTCATCACCAA
CHKA	TGTGGAACTCGTCCTCGCGG
CHKB	TGTTCGCCATACTTGCGGAG
CHKB	GCAGAAGACGACTGGCGATG
CHKB	GGCGTCACGCGACAGCGACG
CHKB	AGCTGAGGGTTTACCCCGTG
CHKB	CGGACACTACCCCAAAACGG
CHRM1	GCGTGCACATGACTGTGACA
CHRM1	CGCTTGGCACGGTAGCTCAG
CHRM1	TCACTGTGGCTAGCGACAGG
CHRM1	GTGGCCCATGAGCAGGTACG
CHRM1	TGAAAGAGATGAGTACCAGC
CHRM2	CGATAATGGTCACCAAACTG
CHRM2	GGCTGCAATAGCCGTACCAA
CHRM2	AAGCGGACCACAAAAATGGC
CHRM2	AGAGGCAACAGCACTGACTG
CHRM2	GTACCAGACTTGGAGAAACG
CHRM3	AAATGAGTGACGGTTCCCGG

CHRM3	AAGTAATGGTGGGCTCACTG
CHRM3	GACCAGAAGATTCATAACAG
CHRM3	CTGTGCCGATCTGATTATCG
CHRM3	GTAGCTGCCGAAATGAGTGA
CHRM4	GATGAGAAGGTTCATGACGG
CHRM4	GAGCACGAAGGACAGTACCC
CHRM4	CGTCAAGAAGCCCCCGCCCG
CHRM4	GCTCAGGGAGCCTGTCACTG
CHRM4	GTAGCCCTTGATGATGTACA
CHRM5	TGGTCACAGAGTCAGAACCC
CHRM5	CAAGTGGTCTACAAGAGTCA
CHRM5	TTGGCTTGCACTGGACTACG
CHRM5	GACCAAGACATTGCCCACAA
CHRM5	GGATGGTCATGACAGAAACA
CHRNA1	GGCCACGACAGAGCCGTCGT
CHRNA1	GCTGATACAGCTCATCAATG
CHRNA1	ATTTAAAGACTACAGCAGCG
CHRNA1	TGGGTGATCAAGGAGTCCCG
CHRNA1	CCAGGTCGTGGAGGTCACCG
CHRNA10	GGACAGATGCCTACCTACGA
CHRNA10	GTGCTGGGCGTCGAACGGGA
CHRNA10	ATCGTGGCGCAGGACCACGT
CHRNA10	CAGCAGCAGGTTGCACACGT
CHRNA10	GGAAGGCTGCTACATCCACG
CHRNA2	GGTGCCCAACACTTCAGACG
CHRNA2	TCGGTATGCGAGCCTCCCTG
CHRNA2	TGATGTTGCCAAAATCAGTG
CHRNA2	CGAAGGCGTAGGTGACGTCG
CHRNA2	GGATCCAGATCATCTCAGAA
CHRNA3	ACAGCGAGTATGTGATGTCG
CHRNA3	CTGAGCACCGTCTATTTGAG
CHRNA3	CTTACTCAAGTACACTGGGG
CHRNA3	CTCTGCCCCACCATAGTCAG
CHRNA3	CATCAAGTACAACTGCTGCG
CHRNA4	TGGTCAAGGACAATTGCCGG
CHRNA4	GGTGAACATGCACAGCCGCG
CHRNA4	GATGATGACCACGAACGTAT
CHRNA4	TGGTGTGCGTGCGTGGCGAG
CHRNA4	AGAAGTCCAGCTGGTCCACG
CHRNA5	ACAGGGTATTATAAGGAACA
CHRNA5	GGGAGATTGTGAGTGCAACA
CHRNA5	AAAACAGTCATCAGGTACAA
CHRNA5	TGATCTCTTCAATAACCAGA

CHRNA5	GCTGGACCAAGAGCAGCAGG
CHRNA6	GGACCACCAGAGATGTGGAT
CHRNA6	GAATGATTATAAATTGCGCT
CHRNA6	AAACGTTTCCGACCCTGTCA
CHRNA6	AAAGCTCTTCTTAAATACAA
CHRNA6	AGATTAATCGTGTAAAACAT
CHRNA7	GGACAGATCACTATTTACAG
CHRNA7	AGAACTACAATCCCTTGGAG
CHRNA7	TCACTGTGAAGGTGACATCG
CHRNA7	CTAGGTCCCATTCTCCATTG
CHRNA7	GGAATGTGTCAGAATATCCA
CHRNA9	TGGGACCGAGATCAGTACGA
CHRNA9	TAAATACTACATAGCCACGA
CHRNA9	TGAACACCAATGTGGTCCTG
CHRNA9	CATTAGCTGAAATACAGTCA
CHRNA9	GCACGATGCCTATCTCACGT
CHRNB1	CGATTCGCTCCGCATCACGG
CHRNB1	CTGCTGCGATAGATGCCCGG
CHRNB1	CTGGCTATGATAGCTCCGTG
CHRNB1	TGGAGGCTGGATTAGCCGAG
CHRNB1	GGTAGAAGAGAGGCTTGCGG
CHRNB2	GGGGTACGGATACAGAGGAG
CHRNB2	TGCGGTCGTAGGTCCACGAA
CHRNB2	CAGTGCTGACGGCATGTACG
CHRNB2	CCACGTACGTAGAGTCGTCG
CHRNB2	GTCCTTCTATTCCAATGCCG
CHRNB3	GATCAAAATGAGGTCAACCA
CHRNB3	CAGGATTCCAGCGTAACTTG
CHRNB3	TCATCAGGGAGCCTTCGAAG
CHRNB3	GGGAAATACTGAACGCAAAG
CHRNB3	CATTAGAATGTAATACAGGG
CHRNB4	GTTGGACCGGACTATCAAGT
CHRNB4	GGATGTTCACACCCTCGTAG
CHRNB4	TGTGGTCATAGGTCCAGGAG
CHRNB4	GCCGATGAGAGGCACATCGA
CHRNB4	TTTCTCAGGGAACTGCCGCG
CHRNE	AACGTGCTCGTCTACGAGGG
CHRNE	TACAGCAAGGACGACTTTGG
CHRNE	TCAGACGTACAATGCCGAAG
CHRNE	GCAATTCATGACAATGAGCG
CHRNE	GCAGCCACACGAGTTCTGAA
CHRNG	AAAACTACGACCCCAACCTG
CHRNG	GCGAGACTACGAAGGCCTGT

CHRNG	TCTCGGTCATGGATAGCTGG
CHRNG	TTGTGCTCAATGTCTCCTTG
CHRNG	GCACCCCCAGAACGAGCGAG
CHUK	AAAGCTCCAATAATCAACAG
CHUK	TATACAGCTGCGTAAAGTGT
CHUK	AGGCCTTTACAACATTGGCA
CHUK	TCCATCTAGAACACATTGAG
CHUK	GTACCAAAAACAGAGAACGA
CIT	GCAGCTAAACCAGCTGACCG
CIT	GCAGCCGATTCTTGTCACTG
CIT	AGACTCGGTGATCCGCTGAT
CIT	CCAGCTTGATGTGTCCTGTG
CIT	GGTAAGAGAGAAAGCAACCG
CKB	GCATGGAGATGACCCGCAGG
CKB	CCACGGTCATGATGTACGGG
CKB	ATCATCGAGGACCGGCACGG
CKB	GGACACGCACCAGATACCGC
CKB	CGATGGCGCGGCGCTCCCCG
CKM	ATCATCTCGGATCGCCACGG
CKM	CAGCAAACATAACAACCACA
CKM	GTAGACGATGTCATCCAGAC
CKM	AGCCTGACGGGCGAGTTCAA
CKM	TCGTCTACAGTGAAGCCAGA
CKMT2	GTCATCAAACTAAGACACAA
CKMT2	CCAGTGCATCCAGACTGGAG
CKMT2	AAATGAGGAGGATCACACCA
CKMT2	CCCAGCACATGTTAATAAAG
CKMT2	CTGCTTGGCAGATCACCCAA
CKS1B	ACGACGAGGAGTTTGAGTAT
CKS1B	TTCGGACAAATACGACGACG
CKS1B	TTCAGATTCAGACATCAGAT
CKS1B	CAGACATGTCATGCTGCCCA
CKS1B	GCACAAACAAATTTACTATT
CLCA1	TATAAGTGGGTGCTTTAACG
CLCA1	GTAGTAAAGAAGTGTCAGGG
CLCA1	CGCACGGGACGTGACAGTCA
CLCA1	GAACCCTACACTGAGCAGAT
CLCA1	TGACAAATCTGGAAGCATGG
CLCN1	AGGAGTGCTATTTAGCATCG
CLCN1	ATGATGACAACGTTGACCCG
CLCN1	AATGAAGACAATACTTCGTG
CLCN1	GACAGGCTCCAGTTCTACCG
CLCN1	CTACTACTCTGATATCCTGA

CLCN2	CCTGTTTGACAATCGGACGT
CLCN2	GCCCCCAGGCACAATCCGGT
CLCN2	ACTCTCGCCAGAATGAATCC
CLCN2	AGTGATGAGGACAACAGGGT
CLCN2	GCAGTTACCTCTTTGCCAAG
CLCN3	GCAACAGGCAACATGTACCA
CLCN3	TGGAGCAAATACCTTTACCA
CLCN3	CAAAAAGTTTGTATGATGCG
CLCN3	AACAGTTCAAAAAGGTACCA
CLCN3	CTTTAGGTCAGTCATCCAAT
CLCN5	TCATCCGAGTGTATTCATTG
CLCN5	TGGGAGTCTACAGTGCAATG
CLCN5	GAGCTCAAAGAGATGCCATG
CLCN5	GAATGAAGCCAAGCGCAGAG
CLCN5	GATAACAGAGGCTTTCAGCA
CLCNKA	ACCTCGGACCACACACCCGA
CLCNKA	CTGACCATACCACTTTGCCC
CLCNKA	GGACGCCGCAGATGCCACTG
CLCNKA	GTAATCCCGGACAGAGAAGT
CLCNKA	TCAGCTTCATCAAGACCAAT
CLCNKB	GCTGTTGAGAGTGTGGTCCG
CLCNKB	CTGCCCATACCACTTTCCCG
CLCNKB	GACTGTGGACTCACCTCCAG
CLCNKB	GAGAACCAAGGTGGCCAGAG
CLCNKB	GTAATCCCAGACAGAGAAGT
CLK1	TTCACATCGTCGTTCACATG
CLK1	ATACTTACAAAGTACTGTTG
CLK1	GCCAAAGAGACCATGAAAGC
CLK1	GAGATCACATAGCAGTGCCC
CLK1	ATTCCAACATCTGGACACAG
CLK4	TATCACAGAGACATTGAAAG
CLK4	ATGAGCGAGATTATCGGGAC
CLK4	CCTCACTCTAGATGAAATCG
CLK4	AACGAGCTGCTTCACGGTAA
CLK4	GCTATCGTGGAAGTCACAAG
CMA1	TGGAGCCCATAACATAACAG
CMA1	CCTTTAGTAACATGATATCG
CMA1	AATTCAACTTTGTCCCACCT
CMA1	AAGACGGAACTTTGTGCTGA
CMA1	GCACAAGAGAAAGAGCAGCA
CNGA1	GTTGTAATATGTGTTTCCCG
CNGA1	GTGAAACACCCCCTCCCGTG
CNGA1	TCGATATGTTTGTACGAACA

CNGA1	CTAAAGGAACCCCTTGCATG
CNGA1	GAAAGGGAGGACCATCACAG
CNGA2	CAGGCCCACGAAAACGCTCG
CNGA2	AGTGATGTTTGGGTAAACCC
CNGA2	CTCAGATGTGGTCTACATTG
CNGA2	ACACTACATGCAGTTCCGAA
CNGA2	GGCCAACAAGAATTTCCGAG
CNGA3	AAAGAAGGATGCGATCGTGG
CNGA3	GGGATCGCCATGGAGACCAG
CNGA3	GCTGGACACCTCCTTAAGCT
CNGA3	TCCCAATCCTGAACATATTG
CNGA3	CCAGTTATAGAAGACAGGCA
CNGB1	GCAGTCCTACCTTTAAACAG
CNGB1	AGGTAACCTGTGTGTCCCCG
CNGB1	GTGCACAGATGAACCCAATG
CNGB1	GGAAGTAGATGAGGTCGCAT
CNGB1	GTACTGACCTGGCTCATGAA
CNR1	GTTTGAACAGAAACACGTTG
CNR1	ACAGGTACATATCCATTCAC
CNR1	ATGAGGAGAACATCCAGTGT
CNR1	GATGAACAGAAGCAGTACGC
CNR1	GGATGACGCACAGCACCAGG
CNR2	GCAGAGGTATCGGTCAATGG
CNR2	GCTGCATGCAAAGACCACAC
CNR2	GACAGCAAGTCCATCCCATG
CNR2	AAGGATTACATGATCCTGAG
CNR2	GAGCACAGCCACGTTCTCCA
CNTNAP1	AGTGGCGCCGACCTTATGTG
CNTNAP1	TGGACACCGTTCTACCAGCG
CNTNAP1	CTCCCGCAGATGTTCATCGG
CNTNAP1	GGCTTCGGCTGACCCCTCGC
CNTNAP1	ATAGGCCAGGTTCTTCCGCG
COL1A1	ATACTTACGACAGCGCCAGG
COL1A1	TGTGTCCCTTCATTCCAGGG
COL1A1	GGGGTCCTTGAACACCAACA
COL1A1	CCAAGAAACCACCGGCGTCG
COL1A1	TCATCTCCATTCTTTCCAGG
COL1A2	TACTTACAGGAGGTCCAACG
COL1A2	CAGGACCAGCAAATCCATTG
COL1A2	AGGAAAGAGAGGCCCTAATG
COL1A2	CAGGGCTTAATGGGACCTAG
COL1A2	ACAGGGCCAGGAATACCGCG
COL2A1	GGATGAAATGAACTTACCGG

COL2A1	GAAGGGCCAACTTTGCCTTG
COL2A1	CGGGAGAGCCACGTTCACCT
COL2A1	TCACTTACATTGGAGCCTGG
COL2A1	GGTTCTCCATCTCTGCCACG
COL3A1	AGGATGACCAGATGTACCAG
COL3A1	TTACTTACATTACTACCAGG
COL3A1	CCAGGACTACCATTAATCCC
COL3A1	ACTCGCCCTCCTAATGGTCA
COL3A1	GGATGACCAGATGTACCAGG
COL6A1	AACAACGACATTGCACCCCG
COL6A1	GGGCGTCAAAGTCTTCTCGG
COL6A1	CGACGCACTCAAAAGCAGCG
COL6A1	AAGTGACTTACGTCAAACCC
COL6A1	GCACAAAGAACAGGTCCACG
COL7A1	CGACGTTCTACGGATCACCT
COL7A1	AAGACTCACATTCGGCCCAG
COL7A1	AGAGTGTGCACGACTGCTGG
COL7A1	TCCAAGCATTGAACTACGTG
COL7A1	CTACTGCCACAGACATCACA
COMP	GGTCGCGACACTGACCTAGA
COMP	ATGCAGCAGTCAGTACGCAC
COMP	GAACCCAGACCAGCGCAACA
COMP	GACAGCGATCAAGACCAGTA
COMP	GGAACTGCAGGAAACCAACG
COMT	CTGGGACGCTCCAACCACAA
COMT	ACTGTGCCGCCATCACCCAG
COMT	CACAGCTGAGTAGCCACAGT
COMT	CCTGCTCATGGGTGACACCA
COMT	AGCGAAATCCACCATCCGCT
CORIN	CACAACAGAGCATCGCTGCG
CORIN	AATCAAGGCAGGGATTGTAG
CORIN	GCCTCACAGAAGGACCTACA
CORIN	AGCACACAGTGATCTGCGAT
CORIN	GCAGCGCTCTTCCGGAGCGA
CP	CCAATCGGCTCAATACTGAG
CP	GAGATGGCAATTGTGTGACT
CP	CACCATATAAGCATCAAACA
CP	AAAGTATATTCCATGTACAT
CP	GTGAAGATGTCTATACCAGA
CPA1	GGTGGCGTAGTTAAAAGTGT
CPA1	TGGGGTCCACGCCAATACAG
CPA1	GATCACTCAAGACTACGGGC
CPA1	CCAGCCATCTGGATCGACAC

CPA1	GCCCTTCCCCAGCATCCAGG
CPA2	ACTGTCTAGAAACATTTGTG
CPA2	TCACTTTGCTCACTAGACCA
CPA2	TGTGGATCCTAACCGGAACT
CPA2	AGAGTGGGTTACACAAGCTA
CPA2	GGAAATCACCCACCACCCCA
CPB2	ATACTGTTCATAGTACGATG
CPB2	TAGAACTTGAACTTGCCTAG
CPB2	ATACCTTTAAAACATAGAGT
CPB2	ACAGTAGGTTTCCGAGCATG
CPB2	CTGTTCATAGTACGATGCGG
CPD	CTGGGGTCGCTAATCCCTGA
CPD	CGCGGCCGCGACTATTGTCG
CPD	AATCTCAGCAACACTAATGG
CPD	AGTAATCCCTGACACGACAG
CPD	AAGTTCCAGGAACAAGTAAT
CPE	CATCAGCAGGATTTACACGG
CPE	TCATCATTCTTGCGACATGG
CPE	GGGAAGGCATGATGTGAATG
CPE	GTGGGTCGAAGCAATGCCCA
CPE	TACCAGGCTCATGGACGCCA
CPM	GATTCCAGAGTTCAAATACG
CPM	ACTGTTATTACAGCATCGGA
CPM	AAATGGGTAACTGGCCACGA
CPM	ATGCTGTAAATATCCTCGTG
CPM	TGTGGGTTCTTGTTGTGGGG
CPN1	TACTATAACGAGAAGTACGG
CPN1	CGAATGCCCCGGCATCACGC
CPN1	GCTGTCGGAGTTTCTGTGCG
CPN1	GCTCAAAGGACTTGTCATAC
CPN1	CCTCCTCCTTCTCTTCAAGT
CPT1A	CACATCGTCGTGTACCATCG
CPT1A	GCTCAGTGAACATCCACCCG
CPT1A	TACGCCAAATCTCTACTACA
CPT1A	ACATCTACCTCCGAGGACGA
CPT1A	TTGCAATTATTCCTAACGAG
CPT2	GATAGGTACATATCAAACCA
CPT2	AACTGGCCATCATTCAAGAG
CPT2	AAATACTGGGACATATCCAG
CPT2	TAAAGGATTTATCAAACCAG
CPT2	GATGTGCCTGGATTTCCGAG
CPXM1	ATCACAATTACAAGGCCATG
CPXM1	AGGGACTCCAGACCCAAAGG

CPXM1	GTCCACCTGAAACCATGGAT
CPXM1	CCGCCGGCGGAGACAGCTAA
CPXM1	TTTGGTCTTGGACCACACCG
CPXM2	CAGGCCATAGCGCTTCACCG
CPXM2	CTGGTGTCATCACTCAAGGG
CPXM2	GCTACGAGAAGGCCTACGAA
CPXM2	GCCCGAGTTCCACTACATCG
CPXM2	GTAATAATCAGGGTCCTCGA
CPZ	CCACCGCTACTTCACGAGAG
CPZ	CACACGCACCATCTGGGCGT
CPZ	CGGCAACATTCATGGCAACG
CPZ	ACCTGACGTCCGAGTACTAC
CPZ	GGGTGCTTGGAGAAGTCGAA
CR2	GCACTTCCTATGATCCACAA
CR2	TTGCAAAGCTGATAACACCT
CR2	TAGATGTTCCAGGGTCAAAG
CR2	TCTGACTATCAACTGTACAA
CR2	TCTTGGCTCTCGTCGCACCG
CRABP1	TCCTGGCGGATCTCCACGTG
CRABP1	CAGGTGTGAACGCCATGCTG
CRABP1	CCTGCAGAGTTTAGCCACTT
CRABP1	CTACATCAAGACATCCACCA
CRABP1	AGAACTGATCCCCGTCCTGG
CRABP2	CAACTGGAAAATCATCCGAT
CRABP2	AACCTTGAAGTTAATCTCTG
CRABP2	GATGCTGAGGAAGATTGCTG
CRABP2	AGCAGTGGAGATCAAACAGG
CRABP2	GCAGTGGAGATCAAACAGGA
CRAT	CACCTGGTAGTTGTGTACCA
CRAT	GCAGCGTCTTGTCGAACCAG
CRAT	CTCATCCCTACAGACCAACA
CRAT	GGGCTCGAGTAGATGACCAC
CRAT	AGACGTGTACCGCAGCCACG
CREB1	GGCTAACAATGGTACCGATG
CREB1	AGCTGTACTAGAGTTACGGT
CREB1	TGGAGTTGGCACCGTTACAG
CREB1	TGTGGAGACTGAATAACTGA
CREB1	AACTGATTCCCAAAAGCGAA
CREM	GCATATATCAGACTAGCACG
CREM	ACAATCCAGATTTCTAACCC
CREM	TACTGTACAATTGTAGCACC
CREM	TGCCTACCTGAGCTAAAGCA
CREM	CCTACTGCTGCTTTGCCACA

CRH	GGGCTGTCGAGCGAGCGCCG
CRH	AGGAGTACTTCCTCCGCCTG
CRH	CAACACGCGGAAAAAGTTGG
CRH	TGGAAGAAATCCAAGGGCTG
CRH	ACTCCCGCGGACACAAGCAG
CROT	GATTGCAGCATTAACTAGTG
CROT	GGAGATCCAACAGTACGCTG
CROT	CTCGACACAGCACTACAATG
CROT	TGGTATACGAACATCCAGAT
CROT	AGTGAAGGAACAGGCAGTGA
CRYZ	GAAAAACTGGACTTTAAACA
CRYZ	CTAATCAAGGTCCATGCATG
CRYZ	GCTGGGGTGATAGAAGCTGT
CRYZ	CACTAGCAGCACGATCTCTG
CRYZ	ACTCTCTCCAGCTTTCACAC
CSF1R	ACGCTACCTTCCAAAACACG
CSF1R	GTTGGAAATCTACTTGATCG
CSF1R	GCTGCCTTACAACGAGAAGT
CSF1R	GCTTGCTAATGCTACCACCA
CSF1R	ATATGACGCTTACCTCTGGG
CSF2RA	AAAAATACTGGACGTCACGG
CSF2RA	AAACCTCACATTGAGACTAG
CSF2RA	TGGGCTGTTTCCACCGTACG
CSF2RA	CCTTATTACATACAAGACTC
CSF2RA	TACCTCTTCCCAGGAAGGGA
CSF2RB	GGTGTTCCCTGTAGGATTCG
CSF2RB	GGTCCGTACTCGGGCCACGT
CSF2RB	ACACATTTGAGATCCAGTAC
CSF2RB	GGGCTTGTAGAATAGGCCAA
CSF2RB	GTACCTGGTGGAGGGCTCCA
CSF3R	GAGCTGAGAACTACCGAACG
CSF3R	CATCACCCACCAACATCCAT
CSF3R	CGGGACCTCTCGTCCCACTC
CSF3R	AAAGCACATTGGCAAGACCT
CSF3R	TGGAGCTGAGAACTACCGAA
CSK	TCTTGCCGTGGAACCAACTG
CSK	CGCACAGCGTGTAGTCTCCG
CSK	TGATGCTGGGCGATTACCGA
CSK	CATTAAACCAAAGGTCATGG
CSK	GCTCCCGTGTGATCTTGCCG
CTGF	AAGACTCGACTCACCCGCGA
CTGF	GGTGGTACGGTGTACCGCAG
CTGF	AAGGGCCTATTCTGTCACTT

CTGF	GGCGTTGTCATTGGTAACCC
CTGF	CGAGCCCAAGGACCAAACCG
CTLA4	TACCCACCGCCATACTACCT
CTLA4	TTCCATGCTAGCAATGCACG
CTLA4	CCTCACTATCCAAGGACTGA
CTLA4	GCAGATGGAATCATCTAGGA
CTLA4	TGGCTTGCCTTGGATTTCAG
CTNS	CTGAAGCTCGTAGAGAAATG
CTNS	GATTTCAAAAGTGATCACCA
CTNS	TGGCAGGCCAGGACACGCGC
CTNS	GAAATCACTCCAATCAGACC
CTNS	CCTGTCGTAAAGCTGGAGAA
CTRC	AGTACCTCAAGAACGACACG
CTRC	GGTGTCCACACCCACAAACA
CTRC	CAACACCCGGACCTACCGTG
CTRC	CTGTCACTCACCGCAACAGG
CTRC	TGTTCTTTCCCACGGCCACA
CTRL	CAGCCAGAGGATTGTCAACG
CTRL	GTTCCAGCTAGGGTGTGTAA
CTRL	ATGATCGGTCATACTCGCCC
CTRL	TCAAACGAGGCTCTGACTGA
CTRL	ACTCACCCCAGGAGGAGCCG
CTSB	TCAACAAACGGAATACCACG
CTSB	CACCAATGCGCACGTCAGCG
CTSB	GGGGACACACTTACCTACAT
CTSB	GTCCTCGGTAAACATAACTC
CTSB	ATCCAGAGTTATGTTTACCG
CTSC	ACTGCAACGAGACAATGACT
CTSC	CCAGCGCGATGTCAACTGCT
CTSC	TCTCCTAATCATATCTCCCA
CTSC	TACAAGACACAACCTCCTGA
CTSC	ACCACAAGAAAAAAAAGTAG
CTSD	GCCATAGTGGATGTCAAACG
CTSD	ACGTTGTTGACGGAGATGCG
CTSD	ACAGACTCCAAGTATTACAA
CTSD	AAGACGACTGTGAAGCACTG
CTSD	CTGCCAGGCCCAGTACTACG
CTSF	GCGCTGGAGATGTTCAACCG
CTSF	GGCCAGTGGTTTCTCAACCA
CTSF	GAGTATTCAGGTAGATAGTG
CTSF	ACCATGGGGTCGTTGCAGGG
CTSF	GCGTCCCCGCAGCCCGGCCG
CTSG	GGGAACAGATACACTCCGAG

CTSG	ATGGTCCGCTGATTATATTG
CTSG	TCTGAAGATACGCCATGTAG
CTSG	GAATCGAAACGTGAACCCAG
CTSG	GCCACCCTCAATATAATCAG
CTSK	CAATATGTGCAGAAGAACCG
CTSK	GGACACCAAGAGAAGCCTCA
CTSK	CAGGAAGCAATATAACAACA
CTSK	ATTACTGCGGGAATGAGACA
CTSK	ACTGGCTATGAACCACCTGG
CTSS	GTTGCATAAAGATCCTACCC
CTSS	TTTCAGTTGAGCAATCCACC
CTSS	CCACTGGCTGGGAACTCTCA
CTSS	GATCTGGGCATGAACCACCT
CTSS	GAGATGCCAGTGGTGATCCA
CUBN	ACGGTTCTATTAAGTCTCCG
CUBN	CAAATGCAATCAGCGTGCGG
CUBN	CAAGTACAGTTGATACCGTG
CUBN	GTATTCACACACTCAACGGG
CUBN	TTAAGAAGCCTTCATAAGCG
CXCL10	ACTCACATGATCTCAACACG
CXCL10	GTAATCAACCTGTTAATCCA
CXCL10	CAGCGTACAGTTCTAGAGAG
CXCL10	CGTGGACAAAATTGGCTTGC
CXCL10	GATAAGGCAGCAAATCAGAA
CXCR1	CAGAACAGCATGACAAACAG
CXCR1	GAAATGACACAGCAAAATGG
CXCR1	CAGGCTCAGCAGGAACACTA
CXCR1	ACTGACCCAGAAGCGTCACT
CXCR1	CTACCTGCTGAACCTGGCCT
CXCR2	GGCGGCATCTAGTAGAAAAG
CXCR2	TAAGATGACCAGCATCACGA
CXCR2	CAGTGGCACGATGAAGCCAA
CXCR2	CCAGCCTGCTATGAGGACAT
CXCR2	ATAAGATGACCAGCATCACG
CXCR3	CTGCCAATACAACTTCCCAC
CXCR3	GCGTGTCTGCTACAGCTAGG
CXCR3	CCACCCAGCTCTACCGCCGG
CXCR3	AGAGCCAAAGACCCACTGGA
CXCR3	CCAAGTGCTAAATGACGCCG
CXCR4	TGACATGGACTGCCTTGCAT
CXCR4	TCTTCTGGTAACCCATGACC
CXCR4	CATCTTTGCCAACGTCAGTG
CXCR4	ACACCGAGGAAATGGGCTCA

CXCR4	CACTTCAGATAACTACACCG
CYP11A1	AGTGTTCACCACGATTACCG
CYP11A1	CGGGCTCCGGAAATTACTCG
CYP11A1	TACTGGTGATAGGCGACCCA
CYP11A1	TAAACCTGTACCATTTCTGG
CYP11A1	GGAGGAAGTAGTGAACCCCG
CYP11B1	TGCTGGTGTACTGTTGAGGG
CYP11B1	CACCTGTTGCCTGGACGCCG
CYP11B1	ACCAGACCTTCCAGGAACTA
CYP11B1	TGTCGCCCAACGCTGTGCAG
CYP11B1	CACTGTCCTGGGGACCCGGG
CYP11B2	AAACGGCAGCACCGTCCTAG
CYP11B2	TGGCCCCACAGGTACAACTT
CYP11B2	TCAACACTACACAGGCATCG
CYP11B2	CATCGGGAGGAACCTCTGCA
CYP11B2	GTCTCGCTGGATCAGCCCCA
CYP17A1	CCATACGAACCGAATAGATG
CYP17A1	ATCGCGTCCAACAACCGTAA
CYP17A1	TATGGACTGTCCGTTGTGGG
CYP17A1	CAATACCTCCTACAAGAATG
CYP17A1	GTCACTCCGGAATTTCTCCT
CYP19A1	TGATAGCAGAAAAAAGACGC
CYP19A1	CAGCATGACACGACGCAGAA
CYP19A1	GAGGGCACATCCTCAATACC
CYP19A1	GCATGAATTCTCCATATACC
CYP19A1	TGACCATACGAACAAGGCCG
CYP1A1	CCTGAATAATAATTTCGGGG
CYP1A1	GTTGTGACTGTGTCAAACCC
CYP1A1	AATTGGCTCCACACCCGTGG
CYP1A1	GCACTACAAAACCTTTGAGA
CYP1A1	AAGGCCTGAAGAATCCACCA
CYP1A2	GCACGGGCGTGGAGCCAATG
CYP1A2	CTTCGACCCTTACAATCAGG
CYP1A2	GTCAGCACATGCCCGAGCAA
CYP1A2	CAGCGGCAACCTCATCCCAC
CYP1A2	GGATGGGGAAGAAGTCCAGG
CYP1B1	TGCGCCCGAACTCTTCGTTG
CYP1B1	GCCACTGATCGGAAACGCGG
CYP1B1	GCGGCCGGGGACTCGCACGG
CYP1B1	ACGGCGCCTTCCTCGACCCG
CYP1B1	GCGCCAGGCGAGCGAACGAG
CYP24A1	ATCTCTTCTCATACAACACG
CYP24A1	GCTGGACAACAAAATCAATG

CYP24A1	TAGTCGCGATAGGCCTTCCA
CYP24A1	TGGTGGCGAGACTCAGAACG
CYP24A1	GTAGATGTCACCAGTCTCGG
CYP26A1	CGTAGCATTCGAGTGCCTCG
CYP26A1	CAGGTAAGTGATCAGAGATG
CYP26A1	ACAAGACGCATCTGTTCGGG
CYP26A1	GGGAAGCCCATAGTCCCGGG
CYP26A1	CTACGTGCCGGTGATCACCG
CYP27A1	CCTGCAGCGATCCATCCCCG
CYP27A1	GTACCCAGTACGGAACGACA
CYP27A1	CCTAAGTAGGACATCCACAT
CYP27A1	AAGCGCAGCTGTCCTAGACG
CYP27A1	CATCACTTGCTCCAAGAGCG
CYP27B1	TGTTCAGGGTTCCGGCGTAG
CYP27B1	GACAGTGCGCACCGTGTACG
CYP27B1	CTCCCGACAAGGCATCGCCG
CYP27B1	CCACGCAGCTCAGAGGCACG
CYP27B1	GCGCGAGCCGAGCAGAACCG
CYP2A13	AGTTCACGGCAACCTCCACG
CYP2A13	CACGCCAAAACCCCTTAGGG
CYP2A13	CAGGAAACCGTAGCGCAGGG
CYP2A13	TGGGATGTACCTCCTGCATG
CYP2A13	CATCGAGGAACGCATCCAGG
CYP2A6	CCCAATGAAGAGGTTCAACG
CYP2A6	AGGCGTGGTATTCAGCAACG
CYP2A6	CTTTGTCCTTATAGTCAAAG
CYP2A6	GGAGTCAATGAAGTCCCGTG
CYP2A6	GCCCACCCCGAAGTCCCGCA
CYP2A7	GGAGTCGGGCTTCCTCATCG
CYP2A7	GTTTCCAATGAAGGGCAGTG
CYP2A7	GGAGTCGATGAAGTCCTGTG
CYP2A7	CTTCATTGCAGGCACCGAGA
CYP2A7	GCCCACCCCGAAGTCCCTCA
CYP2B6	ATGGTCGACCCATTCTTCCG
CYP2B6	CTTCTGCAGATGGATAGAAG
CYP2B6	GCAAGTTTACAAAAACCTGC
CYP2B6	AGAAGAGCGAGAGCGTGTTG
CYP2B6	GCTGAGGCCTTCTCTGGCCG
CYP2C18	CACTGTAACTGATATGTTTG
CYP2C18	GTTTCTGCCTCATGACTCTG
CYP2C18	CAAAATACACAGTGAACACA
CYP2C18	GGAGATAATCGATGAGAGCA
CYP2C18	ATATTTCCAATAATCGGGAG

CYP2C19	AGCAATCAATAAAGTCCCGA
CYP2C19	GGTGCTGCATGGATATGAAG
CYP2C19	TCAGGATTGTAAGCACCCCC
CYP2C19	CAGCTGACTTACTTGGAGCT
CYP2C19	TGGCCTTACCTGGATCCAGG
CYP2C8	GTTTCTCCCTCACAACCTTG
CYP2C8	GGGATTCATGCCAAAATACA
CYP2C8	AAGCAATCGATAAAGTCCCG
CYP2C8	ATATTTCCAATAATAGGAAG
CYP2C8	TCAGGATTCTGAACTCCCCA
CYP2C9	AATGGACATGAACAACCCTC
CYP2C9	TGTCCTTAATACCTATCTGT
CYP2C9	GTAATTTGTTGTGAGTTCCC
CYP2C9	AAACCCATAGTGGTGCTGCA
CYP2C9	TCTACTTTCCTAGCTCTCAA
CYP2D6	CCAGCGCTGGGATATGCAGG
CYP2D6	GGCGCTGGTGACCCACGGCG
CYP2D6	CCGGATGTAGGATCATGAGC
CYP2D6	CTTTGTCCAAGAGACCGTTG
CYP2D6	CCAGCAGCCTGAGGAAGCGA
CYP2E1	GGTGATGCACGGCTACAAGG
CYP2E1	TGTCCCCGCAAAGAACAGGT
CYP2E1	CATTGTAGTCAAAATGCTTG
CYP2E1	GAAGCACTCAGGAAGACCCA
CYP2E1	CCCCATCCCATAGTTCCGGA
CYP2F1	TCCATGTACACAGTGCACCT
CYP2F1	AAAAGGCAGGGTAGTCACCG
CYP2F1	AGGTTTCCCAGGATTGAGAG
CYP2F1	AAGCACTGGATGAAGTCCCG
CYP2F1	TGGTAAAGTTGAAAAAGGCA
CYP2J2	GCGCCCAAAGAACTACCCGC
CYP2J2	TTGCTGAAGAGAGTTTGGTG
CYP2J2	CATTGTTGATCTTGAAATGA
CYP2J2	GGTTCACTCTGACAGCACTA
CYP2J2	GCGACTGCTCGAAGTCCACA
CYP2R1	AATCGCCCACCGTAGCACAT
CYP2R1	TCTTGGAGGCATATCAACTG
CYP2R1	CAGGATGCCAATCCATGGAA
CYP2R1	CTCAATTCCAGATATGGCCG
CYP2R1	GCTTCCCCCCGGGGCCGCCG
CYP2S1	CAGGAGGGTATAGCCGACCG
CYP2S1	GAACCAGGAGAACATCTCGT
CYP2S1	GCTGAGTAAGAAGTACGGAC

CYP2S1	GGCATCGACAAGGTCACGTG
CYP2S1	AGTCCCTTCCAGCATCGCTA
CYP2U1	GTCCCGTCTGGAGACAACAA
CYP2U1	GGAGCAAATGATGTTAGAGA
CYP2U1	AAGGGAAGGTAATAAAGCCA
CYP2U1	TCTGGATAGAGAGAACCCTC
CYP2U1	GCAGCAGGACCAGGGCCGCA
CYP2W1	CCTGCTGGGTCTCATCGATG
CYP2W1	GAGGCCCCACGTGTGCCCGG
CYP2W1	AAGTGCAGGTTCCCGACGAG
CYP2W1	GGTGCTGACGGGGTTCGAGG
CYP2W1	GGTCCCGGTAGTCAAATCGG
CYP3A5	TAGCACTGTTCTGATCACGT
CYP3A5	TCTTAGTGCTCTCCACAAAG
CYP3A5	AGATATGGGACCCGTACACA
CYP3A5	GGCCAGTTCATATAAAGTGA
CYP3A5	TTAAGAGACTGGGAATTCCA
CYP3A7	AAAAGTATAGAAAAGTCTGG
CYP3A7	AGATATGGAACCCGTACACA
CYP3A7	TCGCCTCAAAGAGACACAAA
CYP3A7	ACTCTAGCCTTTCGGGCCAG
CYP3A7	TCTCATCCCAAACTTGGCCG
CYP4B1	CACATCGCAGAAGATGTCAA
CYP4B1	TGATGTGCTGAAGCCCTATG
CYP4B1	GGCATGTCCAAAAAGCCAGT
CYP4B1	GTGGCCCATGACCATACAGG
CYP4B1	GGGCATGTCCAAAAAGCCAG
CYP4F2	GTTTCCCACAACCCCCAAGA
CYP4F2	TGTCGGGGTGGCACAAACTG
CYP4F2	CAAGAACTTACTCCTGACAA
CYP4F2	ACAGGAAGTCAATATGCAGG
CYP4F2	GCAGTTGTCATAGAAGGCGT
CYP4F3	CCAGCAGCACATATCACCGA
CYP4F3	ACAGGAAGTCTATGTACAGG
CYP4F3	AAGAACCAATTCCGTTTCGG
CYP4F3	TGGGAAGAAGTTGTCCGATG
CYP4F3	CAGCAGCAGGAGCAGCCACG
CYP51A1	TACGATGTGCCTAATCCAGT
CYP51A1	GAATGTACGTACCACCCCTG
CYP51A1	TCAACTACTAGTGCTTGGAT
CYP51A1	TTTCCTTTCCATGCAAACAA
CYP51A1	CCATACCTGAAACTAGGCAA
CYP7A1	AACACCTTATGGTATGACAA

CYP7A1	TGTCATTGAGAAACATGCGC
CYP7A1	TTTCCATCATGCTTTCCGTG
CYP7A1	ATTAATCCATTCTCTAGAGG
CYP7A1	AGTTCCTCAGAGCAAATCAA
CYP7B1	GGAGAAATATTATGTGCACG
CYP7B1	AAAAACCACAAGTTGGGACA
CYP7B1	AGCCTCCATTGATAAAAGGT
CYP7B1	AAAGTACATAACATTTATCC
CYP7B1	GCAGAGGGCCAGGAGCAGCA
CYP8B1	TTTGGACGTCAGCATTACAA
CYP8B1	GAGCTTGTTCGGCTACACGA
CYP8B1	GTGTTCACAGTGCAGCTAGG
CYP8B1	TGGATACCGTTCAGTGCAAG
CYP8B1	CATGGTCCCCTTGCACTGAA
CYR61	CTCCAGAATCTACCAAAACG
CYR61	TAGTATCAAGGACCCCATGG
CYR61	CTGCGCCAAGCAGCTCAACG
CYR61	GAACTATCTCTCCCCAACTT
CYR61	AATGAGCTCCCGCATCGCCA
CYSLTR1	AGGTGGAATACACTTGATTG
CYSLTR1	TGTGAACATAATAGACCACA
CYSLTR1	CATACAAAGCATAGGTGCTG
CYSLTR1	AATACCTACACACACAAACC
CYSLTR1	GTTGTAGGCTTCTTTGGCAA
CYSLTR2	GAAACGCACAACACTCAGCA
CYSLTR2	AGATAATAGTCAGCCCTGAA
CYSLTR2	GACCATGAACTATATTGCCT
CYSLTR2	TCAATAATGCTCCTGGACAG
CYSLTR2	ATTATCTTAGAGGCTCCAAT
DAO	AGGGAAAGAACTATCTACAG
DAO	ACTGGACATAAAGGTCTACG
DAO	CGTGATTGTCAACTGCACTG
DAO	ATGGCTCAGGAGATAGTCAA
DAO	GCGTAGACCTTTATGTCCAG
DAPK1	CATGCTCAGGATACGCACCG
DAPK1	ACAACAACATGGATTGACGT
DAPK1	TTGGCACGGCTATTACTCTG
DAPK1	AAGTCAATGATCTTGATCCG
DAPK1	GTTCTCATAGACCTCGTGCA
DARS	CATTAAACTGCTGCTGACGT
DARS	CATCAACAAAGAGAGCATTG
DARS	ATACAATCACAAGAAAAACC
DARS	AGGAAGAGCTACTGTTAACC

DARS	GCAAGAGTCAGGAGAAGCCG
DBH	GGCCACCCCTTACTTCAATG
DBH	CGCACTGGAAGACTTCCATG
DBH	CGTGGTGCTCTGGACCGATG
DBH	CAGTGATGGCCATTCCACCA
DBH	GGTTTCATCTTGGAGTCGCA
DBI	GCACCTTAAGACCAAGCCAT
DBI	TTAAGAACGGCCCGGGATGT
DBI	GATGTTGGACTTCACGGGCA
DBI	ACTGTGGGCGACATAAATAC
DBI	CCACAGTTGCTTGTTTGTAG
DCK	TGTATGAGAAACCTGAACGA
DCK	TTTGAGCTTGCCATTCAGAG
DCK	TCAAGAAAATCTCCATCGAA
DCK	TTCCTGAACCTGTTGCCAGA
DCK	ATCTCCATCGAAGGGAACAT
DCLK1	AAAAGATCGGAACCGGTCTG
DCLK1	CTACAGAGCTCTCAGCATGG
DCLK1	ATAACAGAACGATATAAAGT
DCLK1	CATCTAGCAAGAAATCATCC
DCLK1	TCTCTGGCTTGATATCACGG
DCN	AGATGTAATTCCGTAAGGGA
DCN	GCTGGACCGTTTCAACAGAG
DCN	GGTCTAGCAGAGTTGTGTCA
DCN	AACACTGGACCACTCGAAGA
DCN	TCTGCTTGCACAAGTTTCCT
DCXR	ACACCTGGATGACCGCACGC
DCXR	GGCACTCACAGTAGACGCTA
DCXR	GGCAGGTATAGGGCGCGGCA
DCXR	GAACCCGTGTGCGTGGACCT
DCXR	TCTTGACAGCCTTGTCCGCG
DDAH1	TCCAAAAGGACAAATCAACG
DDAH1	GGCCCTCATCACCCGACCCG
DDAH1	GACCCAATTGCGATCAGGTT
DDAH1	CGGGCGCTACCCGAGTCGCT
DDAH1	TGCAGTCTCCACAGTGCCAG
DDAH2	AGAGGCCTACGAAAAACTCC
DDAH2	GGGACACGGCCCTAATCACG
DDAH2	GGGACCCGAGACTGGCACAG
DDAH2	CAAAGCTCAAAGGGAGCACG
DDAH2	GGATCTGGCCAAAGCTCAAA
DDC	CTTGGATACACACTTACCCC
DDC	ATGGATCACTTTGGTCCGAG

DDC	TAACCCAGCTCTTTCCACTG
DDC	GACTTCTAAGAGATTGTCAA
DDC	AAACGTGTCTGGCTCCTGAG
DDR1	ACTTACGCCCGTCCCCCTCG
DDR1	CCCATGCGCCACAACCTAGG
DDR1	ACCTATGACGGACATACCGT
DDR1	GGGGGTAGAAGCGAACCAGT
DDR1	TCATGACCCGGTCAGCCCGG
DDR2	CCGTGACAAACCGAGCACTG
DDR2	GGGCTAGGCCAATTGACCGA
DDR2	GTAATTGATCTTGTACATGG
DDR2	AAGTTGATGACAGCAACACT
DDR2	GCACTCGCTGGATCTCTTGG
DES	GCGCGACACCTGGTACACGC
DES	CAGAATTGAATCTCTCAACG
DES	TAGAGCTCGGCCACTCGCGT
DES	AGAAATGTTCTTAGCCGCGA
DES	CTACCGCCGCACCTTCGGCG
DGKA	CAGCACTAGCAGTGGCACGG
DGKA	CTCCCAAACTGACCTCTAGG
DGKA	TTATAGGCCATTGGGTACGA
DGKA	GATTGACAAAGACGAGAAGT
DGKA	TATCCTACAGATGATGCGAG
DGKB	TTTGCAGGAGAAGTAGTCCG
DGKB	GAAAGACTGTAAACCTGACG
DGKB	TGAACATGCTGATTGGCGTG
DGKB	AGAGGCAGAATCGCAACTGG
DGKB	AAGCCTGCTCTCCTATCAGG
DGKD	TGAGGCTGTCGATTTCGGAG
DGKD	CTCACGGCACACATTGCAGT
DGKD	TGCACTTGGCGCTCACAGGT
DGKD	CGTCACCGAAGACTTCAGCG
DGKD	AAGTCGTTCCCTGTGCCGAG
DGKG	GCTACCAGAGTGTCACCGCG
DGKG	GGGATGGACTACGACCGGGA
DGKG	CAAAGACTTACCAGTATCAG
DGKG	CTCCACCACAGGCCAAAACA
DGKG	CGAGACCTCTGACCACCCGA
DHFR	CGGCCCGGCAGATACCTGAG
DHFR	AACCTTAGGGAACCTCCACA
DHFR	GTCGCTGTGTCCCAGAACAT
DHFR	GACATGGTCTGGATAGTTGG
DHFR	GAGAAGAATCGACCTTTAAA

DHODH	CATCTTATAAAGTCCGTCCA
DHODH	GTGACTCCAAAACCTCAGGA
DHODH	GAGTCTTGAAATCTGGCCCG
DHODH	ATCTCCCGTGGCCATCAGGT
DHODH	GGATGCTGTGATCATCCTGG
DHRS13	TGGATGAGGATGTCCAACCG
DHRS13	GGCATGTGCCCCTAGCCGCG
DHRS13	TGCATAGCAGGTGACGCCAG
DHRS13	CACCCGTGACCACGGCCGTG
DHRS13	CTGAGGCTACCACCACCACG
DHRS9	CACAGTACGAAGTCTCTCTG
DHRS9	GTGGGTGAAGAACCAAGTTG
DHRS9	TCGCCTTGCAATCGTTGGAG
DHRS9	TCACACTGATGAGTCCAAAC
DHRS9	GAGTCCACAGAAAACCACAG
DIABLO	GTGCAATAGGAACCGCACAC
DIABLO	AGCTTCAATCAACGCATATG
DIABLO	ACCAACTGCAGTCATCCAAG
DIABLO	TTAGTAGTGAAGCATTGATG
DIABLO	TTGTGGCTAACTTTAAGAAG
DLD	ACTGCTACGAAAGCTGATGG
DLD	AATTCTTAGTAAAGGGTCGT
DLD	GCAGTAAAAGCTTTAACAGG
DLD	CCTTACCTTGAAGCCTAACT
DLD	TGATCTGCGTAAGTTCTCAG
DLG2	TGGACCAGCAGACCTAAGTG
DLG2	CCTTTGCCTACCTGACGTAG
DLG2	ATTACGAAGATTATACCAGG
DLG2	GTGCTGTAAACAGGTTCCGG
DLG2	AACCGTCGTCACCTAATCCG
DLG4	AGGCGAATTGTGATCCACCG
DLG4	ATGGGTCGTCACCGATGTGT
DLG4	CATGCAGCACATCCCCAAAG
DLG4	ATGTAACAAAGATCATCGAA
DLG4	GCCACTGGAGAGTAGCGCCG
DMBT1	ACCTTACCTGCATCGACAGT
DMBT1	CAGCTGCCTACAGACCACGT
DMBT1	CAGGACATGAGTCCTATCTG
DMBT1	GGCTGGCTCACCCACAACTG
DMBT1	GGAAATGCCTGGTTTGGCCA
DMPK	CGGACGCGGGGCGTTCAGCG
DMPK	ACACTGTCGGACATTCGGGA
DMPK	TCGAAATCCGGTGTAAAGGG

DMPK	CTTCTACGCGGATTCCACGG
DMPK	GCAGGTGTCGTGCTTCCGTG
DNMT1	GATTTCTGATGAAAAAGACG
DNMT1	GCTCTACTGGAGCGACGAGG
DNMT1	GAGGCAAAAAGAAATCCCCA
DNMT1	TCACCCAAAAAAATGCACCA
DNMT1	GCAGGTGGAGAGTTATGACG
DPP4	GGATTCCAAACAACACACAG
DPP4	CTGCTGTGTAGAGTATAGAG
DPP4	CTACTTGTGTGATGTGACAT
DPP4	GAATATAAAGGAATGCCAGG
DPP4	TGCTCGGCTTGCAGACACCG
DPYD	TCTCCATTGCCATCGATACG
DPYD	TCAATAGGAGTGTAAAAGAG
DPYD	CACACGACTCTTGGTGAGCG
DPYD	GGAAGGTTATAGTAAAAGGT
DPYD	TGTGCTCAGTAAGGACTCGG
DRD1	GAAACAAATACGGCGCATTG
DRD1	GATGTAAAAGCTTATTACAG
DRD1	CAGGTGTCGGAACCTGATAA
DRD1	CAACCTCTGTGTGATCAGCG
DRD1	TGTCCACGCTGATCACACAG
DRD2	TAGCGCGTATTGTACAGCAT
DRD2	CCTGATCGTCAGCCTCGCAG
DRD2	CTCTTCGGACTCAATAACGC
DRD2	GTGGCATAGTAGTTGTAGTG
DRD2	CGAGGAGGTCGGCCACTGCG
DRD3	TGTACACATCATGACATCCA
DRD3	ACTGACTGTTCTGTCGAGTG
DRD3	GAGAGGGCATAGTAGGCATG
DRD3	CACCTCCAGGTATACCACCC
DRD3	GTAGTTGGTGGTAGTCTGCA
DRD4	ACGAGTAGACCACGTAGTCG
DRD4	GCCGCTCTTCGTCTACTCCG
DRD4	CTGCGCTACAACCGGCAGGG
DRD4	CTCTACTGGGCCACGTTCCG
DRD4	CATGGGGAACCGCAGCACCG
DRD5	TCACGATCATGATGGCAACG
DRD5	CGCCACGAAAAGGTCTGACA
DRD5	ACAGTTCTCTGCATTCACGT
DRD5	GACGTCGCAGAACGCTCCAA
DRD5	GCAACGGCACCGCGTACCCG
DSCAML1	CGGATCGCCCACCTAAACCG

DSCAML1	GGTTGCACCACAAATCGAGG
DSCAML1	GAGGATCAAAGGTCAATGCG
DSCAML1	GCTGCAAGGTAACATCCATG
DSCAML1	TCAGTGAGAACTGCTCCCCG
DUSP10	AAGGCAAGGACTCTTTCAAG
DUSP10	GGTAACTAGAGTCTAAACAA
DUSP10	CACTGTGGCAACCTACGACA
DUSP10	TCTTCTTTGCCAAGTCATTG
DUSP10	AAGGCCTGTTCAACTACAAG
DUSP12	CATCACGGCCGTGCTAACAG
DUSP12	CCAGGCAATGGGATACGAAG
DUSP12	ACCGGTCCAGATGGCTGAGT
DUSP12	GACCCAACTACCGTTTCACA
DUSP12	GGAGGAGCCCAGCTTCAAGG
DUSP2	CAGGGCTCCTGTCTACGACC
DUSP2	TGCTGCACGAGACCCGCGCG
DUSP2	GTCACTCGTCAGACCTGCAG
DUSP2	GGACGAGGGCAGTGCCTCGG
DUSP2	GCTGCTGCACGAGACCCGCG
DUSP5	GGGTAGGCAAGCGAGTAGCG
DUSP5	TGTCTCCCGACGGACCTCCG
DUSP5	GGGATATGAGACTTTCTACT
DUSP5	CTACAGGCCAGCTTATGACC
DUSP5	GGAATATCCTGAGTGTTGCG
DUSP6	GACTGGAACGAGAATACGGG
DUSP6	CATTGCGAGACCAATCTAGA
DUSP6	CGAGTCGTCGCACATCGAGT
DUSP6	TTCCTCCAACACGTCCAAGT
DUSP6	CATCGAGTCGGCCATCAACG
DUSP7	GACGACTCGAAGAGCTCGTG
DUSP7	CACCGTGCTGCTCTACGACG
DUSP7	CTACAGAAGCTGCGCGACGA
DUSP7	AGCAGTGCCACCGAGTCAGA
DUSP7	GCTGCTCTACGACGAGGCCA
DUT	CCTGTACAGGTCGTAGCCCG
DUT	CCGCCATTTCACCCAGTAAG
DUT	ACTCTTCCATAACACCCAGA
DUT	TTTGCAGCCAAGCCTGACCG
DUT	CCGGCTCTCCGAGCACGCCA
DYRK1A	TTCAACCAAAATACACCCGA
DYRK1A	TCAGCAACCTCTAACTAACC
DYRK1A	TGAGAAACACCAATTTCCGA
DYRK1A	TTACAGGAGTACAAACCACC

DYRK1A	GAGAAACACCAATTTCCGAG
DYRK1B	AGGCTCGCAAGTACTTTGAA
DYRK1B	GATGAAGTACTATATAGGTG
DYRK1B	CACAGAGAGCTTACGCAGCG
DYRK1B	CATGACTACATCGTGCGCAG
DYRK1B	CCAGGATTCGAGCAACAAGA
DYRK2	TTGAGGATAACAGTAACAAG
DYRK2	CTAAATGCTAAGAAGCGCCA
DYRK2	ACAGCATTCATAGACGGCAG
DYRK2	CAAGCACTGCAGAATCGAGT
DYRK2	GCAGGTGCCCCACGATCACG
DYRK3	CCACGGGCGCAGTTCAACCA
DYRK3	TTGGTGGTCCCAATAATGGA
DYRK3	GGCATCCAAAGATTGCAAGA
DYRK3	TGCAGAGTACATTTGAACAG
DYRK3	CCTCATATCGATAAGCTAGA
ECE1	AGTGACACAGAAAACAACCT
ECE1	GAGGGCGACGCATACCCCAA
ECE1	TGGTCTCGTTCATGCACGCA
ECE1	GGAAACCCGAAAATCAGCCA
ECE1	GCTCATCTACCACAAAGTGA
ECE2	GTTTAAGTAGTAATCCCGAG
ECE2	TGTGGACTACTCATCAGTCG
ECE2	TTGGTGGTTGGAACATTACG
ECE2	CTTGTGCAGACTCAAAGCGT
ECE2	GGACTACTCATCAGTCGTGG
EDNRA	TGTCAACACTAAGAGCGCAG
EDNRA	CACATAGATAAGGTCTCCAA
EDNRA	TGAAGCGATTGGCTTCGTCA
EDNRA	CAACATCTCACAAGTCATGA
EDNRA	TGTTCACCCCTATATTCAAA
EDNRB	GGGTGGCGTCATTATCTCTG
EDNRB	CCCCAGCACGAACACAAGGC
EDNRB	GCACCTGCGGAGGTGCCTAA
EDNRB	CAATATCTTGATCGCCAGCT
EDNRB	TTACACATCTCAGCTCCAAA
EGF	GAGAACATCTCTCAACCACG
EGF	GAAGCAGAACAAATCCTACA
EGF	ACCGTATCTTCTATTCAACA
EGF	CTTTATAGAGCAGATCTCGA
EGF	GGATGAACAGCAATTCCTCG
EGFR	TGTCACCACATAATTACCTG
EGFR	GTGGAGCCTCTTACACCCAG

EGFR	GTCTGCGTACTTCCAGACCA
EGFR	TCTTGCCGGAATGTCAGCCG
EGFR	CTCTTCTTAGACCATCCAGG
EHMT1	GGGCCGGTGCACAAACAGCG
EHMT1	TTCGGCTGCTTCCATCAACG
EHMT1	ACTTATACGACTCAGAACCT
EHMT1	CAACACACTAACTCGGATAG
EHMT1	GCACACTCAGGACAGCGCAA
EHMT2	CAAGAGGTGACCATCCCCCG
EHMT2	CTCCAGGTGGTTGTTCACCA
EHMT2	CGGACAGGTACAACTGCCGA
EHMT2	CTCTCCGTCCACACTCTCAG
EHMT2	GATGAATCTGAGAATCTTGA
EIF2AK1	ATAGTCGAGAGAAACAAGCG
EIF2AK1	GGCCCGGACCCCGAATATGA
EIF2AK1	TTGTTGGCTATCACACCGCG
EIF2AK1	AACACCTGTCTTGAACGAAG
EIF2AK1	ATGAACATGTTCTATCCACG
EIF2AK2	ATTATGAACAGTGTGCATCG
EIF2AK2	GCAACCTACCTCCTATCATG
EIF2AK2	ATGGTCTCAGAAATAATCAA
EIF2AK2	AAAGGCAATACGTACCACTG
EIF2AK2	GATGGAAGAGAATTTCCAGA
EIF2AK3	GAATATACCGAAGTTCAAAG
EIF2AK3	GTACCACCCATTACCTATTG
EIF2AK3	GAGACAGAGTTGCGACCGCG
EIF2AK3	TTATCTACCATACTACAAGA
EIF2AK3	GCAGCCCCTCACCTGCCGCG
EIF2AK4	GCAAGACGACTCCATCGTGG
EIF2AK4	AGGATGACCGAGCTGCACGC
EIF2AK4	GTAGGCCTTCCCATCCACGT
EIF2AK4	ACTGGCCAAGAAACACTGTG
EIF2AK4	ATGCTCTGCCTTATAAAACG
ELN	CCCCCGGAAAGGTAACTGCG
ELN	TGGAGGCATTCCTACTTACG
ELN	GCAAGGCTGGTTACCCAACA
ELN	ACGCCACCTGGGTATACACC
ELN	ATTCCTGGTGGAGTTCCTGG
ENO1	AGGTCCTACCTTGCTAACCA
ENO1	ACAGCAGCTCTGAAGAGACC
ENO1	GGTCATCGGCATGGACGTAG
ENO1	GGAAAGATGCCACCAATGTG
ENO1	ATACCAGTTGAAGCACCACT

ENPEP	GGTTCTATAAAATCCCACGA
ENPEP	TATCCTGTGCTTAACGTGAA
ENPEP	CTCATCAAACCAACAGAGGG
ENPEP	ACACCAGGTACGTGCTCATG
ENPEP	AGCCTCATCAAACCAACAGA
ENPP1	TATGGACCTGGATTCAAGCA
ENPP1	GCAGTTTCCAAGCTCAACAC
ENPP1	CACCTATAAAGTACTCTCGC
ENPP1	AACTACAGTTCTGTGTGTCA
ENPP1	GTAGGAGGCGTTTCAAACCT
ENPP2	GCACACACTCTCCCTACATG
ENPP2	TCACCCTGCCAGATCATGAG
ENPP2	CTGCTGTACCAATTACCAAG
ENPP2	GGGAAATCGACAAAATTGTG
ENPP2	TTAATATTCTCCGCTCGTGA
ENPP3	CTTTACATGCCACATCACAC
ENPP3	ATTCAACCAGCACCAAACAA
ENPP3	AATGGTGTAATGATTTGGGA
ENPP3	ATTATGAGCTCGGATGCGGG
ENPP3	GATGTGGCATGTAAAGACCG
EP300	GGTACGACTAGGTACAGGCG
EP300	ATGGTGAACCATAAGGATTG
EP300	GTGGCACGAAGATATTACTC
EP300	CTGTAATAAGTGGCATCACG
EP300	TGGTGACTCCAGTTGCCCAA
EPHA1	GGTCCGAAACTCATGATCAG
EPHA1	CGACTCACCTCGATCCACAT
EPHA1	CTCCAATTGGATCTACCGCG
EPHA1	AAGCCCAAAATGGAGTGTCA
EPHA1	GACTGGTGAAGAAAGAACCG
EPHA10	GCGAGTAGGTGACGTCCGAG
EPHA10	CGAGATCCGATACTACGAGA
EPHA10	CCGCAAAATCGACACGATCG
EPHA10	TTTACAAGGTGTCCCCGCGG
EPHA10	AGTCTTCTCACCTTCCTCGG
EPHA2	GAAGCCCCTGAAGACATACG
EPHA2	CACACACCCGTATGGCAAAG
EPHA2	TCACGGAGAAACCCTCGGTG
EPHA2	CTGGTGCGGGTCAGTCCGTG
EPHA2	CATGAACTACACCTTCACCG
EPHA3	TGGGATCATATTGGACTACG
EPHA3	ACTCCAGTCCAGGATAACTG
EPHA3	GTAGGTCCTGTCAACAAGAA

EPHA3	ATTGCAGGAACACTTGCCAA
EPHA3	AGACCCTCTAACCTCCACCA
EPHA4	TTGGAGGTTACAACCAACAC
EPHA4	TGTGCCAAAAATGTACTGTG
EPHA4	TGTAATTGGTATGAGCTAGG
EPHA4	TGGGATCTTCGTACGTAAAG
EPHA4	GTTTCCTGACACCATCACAG
EPHA5	GGTGAGGCTTTGAAGAACCC
EPHA5	ACATTTGAGATGGCATTCCG
EPHA5	TAACCATATTAGGCTTGGAT
EPHA5	TAGAACTCAAATTTACCCTG
EPHA5	GGAGGCTCGGAGAAGATGCG
EPHA6	ACAGTGACCACGGATCAAGA
EPHA6	AGAACTGCGACAGGATACAG
EPHA6	TGGTACATGCCATAGAAGGT
EPHA6	GAAAACATATCCATTAAATG
EPHA6	CTCTCAATACGAATTCTTGA
EPHA7	CTGACTTAAGCCGATCCCAG
EPHA7	CACCTGGTATGTTCGTATCG
EPHA7	TTGTGCACGCAACGTATGGT
EPHA7	AGCTGTCCATCAATTCGCCA
EPHA7	GTACCGAGTTTGAAAAACCA
EPHA8	CCTCAAAATCGACACCATTG
EPHA8	CCCAGAGCCCCAGTTCTATG
EPHA8	GCTCACGTATCCGGCTCATG
EPHA8	CGTCAGGCAGATCCAGACAA
EPHA8	GGACACGTCGACCATCCACG
EPHB1	TCGGACCGGTTATTACCGAG
EPHB1	AGGATCCTGGGACATTAGGG
EPHB1	TGAGGAGCATCACCTTGTCA
EPHB1	GCTGGCTACGGCAAGTTCAG
EPHB1	GTTTCCAGAGACTATGACAG
EPHB2	AAGCTGCAACACTACACCAG
EPHB2	AGGTCACTGATGTAAATGCG
EPHB2	ACCAAGTTTATCCGGCGCCG
EPHB2	CTTGCGGTAGAAGACACGCA
EPHB2	GTTGGTGGTGATGTTCACAG
EPHB3	TTCACGGCCGCATAACGAGG
EPHB3	AGAGGTGAGTGGCTACGATG
EPHB3	TTGGAGATCACACCTCGGGG
EPHB3	CTGTAGCGGAGCCCACGATG
EPHB3	GCACCCCCAGAGCGGCCCAA
EPM2A	GTTACATGTTCCACCTGACG

EPM2A	AATGAAAACAACTTGGTGGA
EPM2A	GTACAATATCCCATTCAGTC
EPM2A	ATCATAGTGTCTGGAGTCAT
EPM2A	TGTACCAGAACGTGTCCACG
EPOR	TCGGCTCAGCCATACGCGCG
EPOR	GGGCACGAAGCTCGACGTGT
EPOR	GGAGCCAGCGCAACACTACG
EPOR	CGGATCGGACTCACTCGAGC
EPOR	GGGCGCATAGGGATGAGCCA
EPRS	AATGTATATCAAAACACACG
EPRS	CTCAGTACACCACGAACCGT
EPRS	ACTTTGGGACTATACCACAC
EPRS	AGTAGAGTATAAGCCTGTGT
EPRS	GATTTCCTCCAGAGGCCAGT
EPX	AGTACCGCACCATCACTGGA
EPX	TGGAGACAAACTGTACAATG
EPX	CTCCAATGTGGACCCACGGG
EPX	GAGAGACTGACCTCCGACCG
EPX	CCTGTCCTACTTCAAACAAC
ERBB2	AACTACCTTTCTACGGACGT
ERBB2	TTGGGATCCTCATCAAGCGA
ERBB2	TCATCGCTCACAACCAAGTG
ERBB2	GTTACCTATACATCTCAGCA
ERBB2	GAGTCCATGCCCAATCCCGA
ERBB3	ATGAGGCGATACTTGGAACG
ERBB3	TGTCGAAATTATAGCCGAGG
ERBB3	ATCATGTGAGACAACACCGG
ERBB3	ACCATTGCCCAACCTCCGCG
ERBB3	GCTCTACGAGAGGTGTGAGG
ERBB4	CTGGTGTGTCCAGATAGCTA
ERBB4	AGCGGCGACACGACAGACAT
ERBB4	ATGTCCAGATGGCTTACAGG
ERBB4	ATAGAGTACTCTTCCACCAA
ERBB4	GTGTGTGCAGAACAATGTGA
ERCC6	GGGTGAAGGAATTTCACACG
ERCC6	ACTGATTACGAGATACAATG
ERCC6	AGACAGAATGATCCGATGAG
ERCC6	AGTGATGCTGAATTTGACGA
ERCC6	TCATACCTGTTTGCAAGCAA
ESR1	TCAGATAATCGACGCCAGGG
ESR1	CTGACCGTAGACCTGCGCGT
ESR1	TACTCGGAATAGAGTATCGG
ESR1	TCCAGGTACACCTCGCCCAG

ESR1	GTAGACCTGCGCGTTGGCGG
ESR2	CCAGTTATCACATCTGTATG
ESR2	TCAGCCTGTTCGACCAAGTG
ESR2	CCCCAGTGCGCCCTTCACCG
ESR2	AGCAGGGCTATAGAATGTCA
ESR2	TTGAACCTGGACCAGTAACA
ESRRA	AGACACCAGTGCATTCACTG
ESRRA	CTCCGGCTACCACTATGGTG
ESRRA	GTGGGCGGCAGAAGTACAAG
ESRRA	CCACAATCTCTCGGTCAAAG
ESRRA	GCCAGCCCTGACAGTCCAAA
ESRRB	TGGCGTCGGACGAGCCACTG
ESRRB	AGTGCGAGATCACCAAACGG
ESRRB	CCCATGCCGCAAGAGCTACG
ESRRB	CTCTGGCTACCACTACGGCG
ESRRB	ACGAGTGCGAGATCACCAAA
ESRRG	GTACAAGCGCAGGATAGATG
ESRRG	AAGAGACTGTGTTTAGTGTG
ESRRG	GTTGACGCTGTCCGTCAGGG
ESRRG	GATAACCACCAACTCTCGGT
ESRRG	ATAGGAGCAGAAGGGTAGAG
ETV1	TCCTTTGCCGACGATGCCAA
ETV1	TGTCATCATAAAACTGCCTG
ETV1	TAAGTCAATTACAGGAAACA
ETV1	TTGGAGATGCATGATGCAGT
ETV1	AGTGTATGAACACAACACCA
EXOG	GGTGATTGGCGAGGACAACG
EXOG	TGTCTTATGATCAGGCAAAG
EXOG	AAGATTATGTTGGAAGTGGG
EXOG	ATCGCCTCTAGTCTGAGGTA
EXOG	CTGACTCCGGAAGAACTGCA
EZH2	ATGTTGGGGGTACATTCAGG
EZH2	AGAAGGGACCAGTTTGTTGG
EZH2	TTATGATGGGAAAGTACACG
EZH2	CTTCTGTGAGCTCATTGCGC
EZH2	TTATCAGAAGGAAATTTCCG
F10	CAAATGTAAAGACGGCCTCG
F10	CCACAGAGTTCTGTTCCTCG
F10	CAAGCACAACCGGTTCACAA
F10	GCTCATCAATGAGGAAAACG
F10	CCTTCCAGTGTTCATCCGCA
F11	TATGTGGACCTAGACATGAA
F11	CATCTGAGGATCCCACCCGA

F11	AAGGTAAAAAACAAGCAACC
F11	TACTGAAGCACACCCAAACA
F11	AGAATGCCAAGAAAGATGCA
F11R	TCAAACTTCCACTCCACACG
F11R	TGTAACACTGCCCAATGCCA
F11R	GGAAGGCGGCAACAGCTATG
F11R	GACTTGAAGGTGATACCAGT
F11R	GGTCAAACTTCCACTCCACA
F12	TCATCGAAGACAGACTCTTG
F12	CTCGGCAGTCACGTTCCGGT
F12	ATGAAGCCTAGGGGACACCG
F12	TACCACAAATGTACCCACAA
F12	GCGTGGTCCTGGCCAGGCCG
F13A1	TATTGTCCCAGGATCCAACG
F13A1	TAAGAAAGATGGCACTCATG
F13A1	TGAGAGAGGACAGGTCTGTG
F13A1	GGAGAGATGGGACACTAACA
F13A1	GCTGCATTAGAGTTATTGGG
F2	GGTCTGGGTACGAACTACCG
F2	GCAGCTCACCACAATAGTTG
F2	GCCTGGCGGTGACCACACAT
F2	ACCAGACTTTCTTCAATCCG
F2	GGGTCTGTAGTGTAGCACCA
F2R	GGAGCTGGTCAAATATCCGG
F2R	AAATGACCGGGGATCTAAGG
F2R	TGGCCATGATGTTTAGTGGG
F2R	CACAAACAGCACATCTGCCG
F2R	TTCCTGAGAAGAAATGACCG
F2RL1	TGTGATAGGCAATCTTCAAG
F2RL1	GGGTTTGCCAAGTAACGGCA
F2RL1	CCCAATACCTCTGCACACTG
F2RL1	GTCTGCTTCACGACATACAA
F2RL1	TGACCTGCCTCAGTGTGCAG
F3	CCTTCACAATCTCGTCGGTG
F3	TAAAGGCACTACAAATACTG
F3	CAGGTAAGGTGTGAACTCTG
F3	CAGGAGCGTCCGAGCGACGG
F3	GCAGGGAATGTGGAGAGCAC
F5	AAGACCATACTACAGTGACG
F5	TGATCGAGGATTTCAACTCG
F5	GGGAAAGATCTGTCTCACCA
F5	ACGGTCACAATGGATAATGT
F5	CCTTGACCACACATTCCCTG

F7	GAACTGGAGGAACCTGATCG
F7	TTTCCCAGTCTTCGTAACCC
F7	CAGTACTGCAGTGACCACAC
F7	CAAACCCCAAGGCCGAATTG
F7	ATCTGTGTGAACGAGAACGG
F8	ATACTAGTAGGGCTCCAATG
F8	TCAGATAAGGATAGCCCATG
F8	ACTTCCTACCAATCCGCTGA
F8	TAGACCTATATATCTGACCA
F8	GCTTTACTCTCCATTCCCAA
F9	AAGTCGATATCCCTCAGTAC
F9	AGGAAAAACAGTCTCAGCAC
F9	TAACACCAGTTTCAACACAG
F9	TTTAAATGGCGGCAGTTGCA
F9	TTTCTAGTGCCATTTCCATG
FAAH	GGAACATTGGTGTGCACGAA
FAAH	TCTCATAGTACCCCACACGC
FAAH	CAGGCCTGGGAAGTGAACAA
FAAH	GAAGAGCCCACCTGTTGACA
FAAH	GCGCTGGTCCGGGCGCCGGA
FABP4	CATGATCATCAGTGTGAATG
FABP4	ACTGAGATTTCCTTCATACT
FABP4	TTATATGAAAGAAGTAGGTA
FABP4	GACACCCCCATCTAAGGTTA
FABP4	CATGCCAGCCACTTTCCTGG
FAM57A	GCGACGACGTGATCACCGGC
FAM57A	AAGTTTCGAAGAGTGAGGGA
FAM57A	AGCCGACAAAGAAGTCCCCA
FAM57A	CCTCATGATCACACATCATG
FAM57A	GATGCTGCTGACGCTGGCCG
FANCA	AGACGCATACTGACCACTCG
FANCA	CCTCCACTCACAAGATCGTG
FANCA	GACACACAGAACCTTCCGAG
FANCA	GAGCCACGGGAACACATGGT
FANCA	GCTCCACAGTCAGCAGCACA
FAP	ATGCACTTGTCTGCTACGGT
FAP	ATGAACGAGTATGTTTGCAG
FAP	ATTACGGCTTATCACCTGAT
FAP	ATCTATTTGAAACAAAGACC
FAP	CCTGGGGACCTACATACGCA
FASN	CGAGACCCCGAGACACTCGT
FASN	GATGTATTCAAATGACTCAG
FASN	CATCTCCCCACTCATCAAGT

FASN	TTCCATCCTACGCTCCGATG
FASN	GAGCATGCTGAACGACATCG
RPL7	TTCAGCTTCGAAAGGCAAGG
RPL7	CGTTTGTCATCAGAATCAGA
RPL7	TAGAGCCATATATTGCATGG
RPL7	GAACTGAAATTCGAATGGCG
RPL7	ACAGAACCTCACCTGTTTGG
FBN1	TTTCTCCTTACCGATACACG
FBN1	TATTTGGATATTCGACCTCG
FBN1	ACGTACCAATACACTCCCCA
FBN1	AGTGCATGCACATCGATTTG
FBN1	GGAGAGGTGTAAAAACCAGG
FBP1	ATGTTGGAAGATCCATCAAG
FBP1	AAAATCTACAGCCTTAACGA
FBP1	TTCTGACACGAGAACACACG
FBP1	GCTGGTTCTACCAACGTGAC
FBP1	GTGTTGACGTCCGTGTCGAA
FCGR2B	TGGAGCACGTTGATCCACTG
FCGR2B	AGGGAGAAACCATCGTGCTG
FCGR2B	CACAGAAGCATATGACCCCA
FCGR2B	TGGAGAAGTTGGGATCCGAA
FCGR2B	CTGTGACTCTGACATGCCGG
FCN1	CTGGAAAGGCAGGACCAGTG
FCN1	GACTGGGCCGCATACAAGCA
FCN1	GCAAGGACCTGCTAGACCGG
FCN1	ATCCACGCCCTGACTGCCCA
FCN1	CCATTCTCCGAGGCTGCCCG
FDPS	GTATAACCGGGGTTTGACGG
FDPS	GGTGGCTGGGTTCCCTACGG
FDPS	AAGCGTGGACAGAGGAACCT
FDPS	GATTCATCCCTTACCCGCCG
FDPS	ATTGGAGGCAAGTATAACCG
FDX1	GTCCACTTTATAAACCGTGA
FDX1	GCAGGCCGCTGGATCCAGCG
FDX1	GTGACAGGTTGAACAAGCCA
FDX1	GGTCGCTGAGCGTGTCGGCG
FDX1	GCTGTCCTCGGCGGCCCGGC
FES	CATCGAACGTGACACAGGGT
FES	GACTGGATGATGAAGTGCCG
FES	GGCAGACAAGGACCGTGACA
FES	TGAGATCACCAGCCAAACTG
FES	GCATTTGCTGCAGGACCCCG
FFAR1	CGGATGGCCAGGACGTTGAG

FFAR1	CGGAAGGCTTGGTAGCCCAA
FFAR1	AGCCCCCGCCGGCATAGAGT
FFAR1	CAGACCGGAGAGCCGTTGAC
FFAR1	GGAATAGCACGGCCTCCGGA
FGA	ACAGTCAGAACCATCTTCGG
FGA	GGAAATGAGATTACTCGAGG
FGA	GGTTGATATGAAACGACTGG
FGA	TAAGTGTTGCCTATCTCTAG
FGA	TTAAGATCCGATCTTGTCGA
FGB	GGATTGAACGAAGCACACGA
FGB	TGTCAGTTGCAATATTCCTG
FGB	TGATGAGTTAAATAACAATG
FGB	CCAAGGTGTCAACGACAATG
FGB	TCTTCTCTCTTCTTGTCAAG
FGF1	TGAGCCGTATAAAAGCCCGT
FGF1	CTCCTCTACTGTAGCAACGG
FGF1	TTCCTGAGGATCCTTCCGGA
FGF1	AGAAGTTTAATCTGCCTCCA
FGF1	GGCTTCTTGTAATTCCCTGG
FGF2	TCTCCCGGACCCCGTCAACT
FGF2	GCCACTTCAAGGACCCCAAG
FGF2	GGGTGCCAGATTAGCGGACG
FGF2	TTCACGGATGGGTGTCTCCG
FGF2	CGGACAGAAGAGCGGCCGAG
FGF4	ACTACCTGCTGGGCATCAAG
FGF4	TGCACCCAACGGCACGCTGG
FGF4	GGCCGCGCCACTCACTGTCG
FGF4	GCGCCAACGAGAGCGCCACC
FGF4	GCGCCACCAGGCTCTCCCAG
FGFR1	GTTGCCCGCCAACAAAACAG
FGFR1	CTGGTCTTAGGCAAACCCCT
FGFR1	AGTTCAAATGCCCTTCCAGT
FGFR1	ACAGTGTGTACCTTCCAGAA
FGFR1	TCTTACCCACGACATCCAGC
FGFR2	CTTAGTCCAACTGATCACGG
FGFR2	TGTGTCTGTTCTAGCACTCG
FGFR2	GCCGGCAAATGCCTCCACAG
FGFR2	GATAGCCATTTACTGCATAG
FGFR2	ACTCACCCACAACATCCAGG
FGFR3	CATCCGGCAGACGTACACGC
FGFR3	GGTGCTGAATGCCTCCCACG
FGFR3	AAGAACGGCAGGGAGTTCCG
FGFR3	CCCGAGACAGCTCCCATTTG

FGFR3	GCTGCCGGCCAACCAGACGG
FGFR4	GAGGTAGATCTAGACTCACG
FGFR4	TTGCACATAGGGGAAACCGT
FGFR4	GGTAACTGTGCCTATTCGAG
FGFR4	TGGTGGCCACTGGTACAAGG
FGFR4	TGGAGCCTCGTGCCAGGCAG
FGG	TTATCAAACCAAAGTAGACA
FGG	CATCCCATATGCATTAAGAG
FGG	AATAAGGGAGCTAAACAGAG
FGG	ATTTGCACCGTGTCTTTGCA
FGG	AGTAGAGAATTAAATTCCGG
FGL2	GGGAGCTGAATAGTCAAGGG
FGL2	GAACAAATACAGTCACGTCC
FGL2	ACATGGCAAGACTACAAAGC
FGL2	GTTACCCAGTACAGGAGCCC
FGL2	GTTATCACCAACCTCTCCCG
FGR	CGAGTTGAACTGAACCCGTG
FGR	AGGGGACTTCAGAAGCTACG
FGR	AGCTTGGATTGAGTCAACAG
FGR	GTGACCGAGTTCATGTGTCA
FGR	GGTCTGATCCCAGTCCCGGA
FIBCD1	AGGACGTCCAGACAGTCTCG
FIBCD1	GGCGTGCGAAGCTGTCGGTG
FIBCD1	GGTGTACTGTGACATGCGCA
FIBCD1	CCAGCGCCCGAGCGTACCTG
FIBCD1	CGTACCTGCGGCTTGTCGCG
FKBP1A	GGGCGCACCTTCCCCAAGCG
FKBP1A	ACCGGTGTAGTGCACCACGC
FKBP1A	GGCAAGCAGGAGGTGATCCG
FKBP1A	GAAACCATCTCCCCAGGAGA
FKBP1A	GTTTATGCTAGGCAAGCAGG
FKBP1B	CAAGAAGGGCCAAACGTGTG
FKBP1B	GAGACCATCTCCCCCGGAGA
FKBP1B	TGACAGGAATGCTCCAAAAT
FKBP1B	ATCCCTGCTAGATGAGCTTG
FKBP1B	GGAAGGACATTCCCCAAGAA
FKBP4	AGCACCATAGTGAAAGAGCG
FKBP4	AGGCTATGCTAAGCCCAATG
FKBP4	AGGACTGCCTGCTGAACCGT
FKBP4	CCCCCTCGCCAATCTCAAAG
FKBP4	GATGAAGGCGACCGAGAGCG
FLT1	ACAGCCACAGTCCGGCACGT
FLT1	AGGTTGAGGGATACCATATG

FLT1	CTTACCATATATATGCACTG
FLT1	TGGCCACTGTGTGATCACTG
FLT1	GGTCAGCTACTGGGACACCG
FLT3	AAAGCTGTTCATGTGAACCA
FLT3	GGTGCTTTGCGATTCACAGG
FLT3	GTAACCAAAGCTGATTGACT
FLT3	GGGGTCTCAACGCACACCCG
FLT3	AGATACATCCACTTCCACAG
FLT4	GCCCTCCAGTCACGGCACTG
FLT4	CTCACCTCTCACGAACACGT
FLT4	CATCGAATCCAAGCCATCCG
FLT4	CATACCATGCACAATGACCT
FLT4	GCGATTTCGGGAGAGCACCG
FMO3	ACTGTGTAATCTTTGCAACA
FMO3	ACATTCACAGGACCATGCAG
FMO3	CTCCCAGCAAGCATTCTGTG
FMO3	CTGTCGGTAAATTGTTCTTG
FMO3	GTAGTACCTGTTCTGCTGTG
FMO5	TTGGATCCTGAATCGTGTAG
FMO5	GGTTCTTATAGTCTCGACTG
FMO5	AAAGTCAAAGCTATAGCCTG
FMO5	GAAAGGACTGATGACATCGG
FMO5	AAGTGGTCACTGAATCTGAA
FN1	TATTCCACCTTACAACACCG
FN1	GATGCGGTACCCAATAATGG
FN1	CTGGGACTGTACCTGCATCG
FN1	AAGCCTGGTGTGGTATACGA
FN1	GCTTTGACTGACAGCCACCG
FOLR2	AACTTTAACTGGGACCACTG
FOLR2	AGAGGACTGTCAGCGCTGGT
FOLR2	ACTGGCACAGAGGATGGGAC
FOLR2	AAGCACCACAAGACAAAGCC
FOLR2	GCGCCAGCTCTGATTCACCT
FPR1	GGACCAACGACCCTAAAGAG
FPR1	CTACAGTACCTGGTAAAACG
FPR1	CTGACAGCAACGATGGACAT
FPR1	TGGTAAAGACGAATTTGCAC
FPR1	GATGCAGGACGCAAACACAG
FRK	CTATATTCCTTCTAACTACG
FRK	AGTTGCGGTCTATCTCCCAT
FRK	GCAGTGAAAACATTAAAACC
FRK	CACTACACCAAGACAAGTGA
FRK	TTTGCTCTCCCCAGTCACAG

FSHR	TCTAGGTTAATATCCAACAC
FSHR	GATTATATGACTCAGGCTAG
FSHR	TTGAAAGAAATTCTTTCGTG
FSHR	GTTGAATGCATCTGGCTTAG
FSHR	GATCTGTCACTGCTCTAACA
FST	AAGGAGTCCTACCTTTACAG
FST	TGGCTCCGTCAAGCGAAGAA
FST	GAGTGCACATTCATTGCGGT
FST	GGTGATGTTGGAACAATCCG
FST	TCTTGTACAGGACCTGGCAG
FTH1	TGTTCACCTTGATATCCTGA
FTH1	CACCATGGACAGGTAAACGT
FTH1	GGAGAGCGGGCTGAATGCAA
FTH1	TCTTCAAAGCCACATCATCG
FTH1	GGTGCGCCAGAACTACCACC
FURIN	GCCACGGCGATTATAGGACA
FURIN	AAAGCCCGTAGCCATATGAG
FURIN	CAACGTGCCGTGGTACAGCG
FURIN	TATTACCACTTCTGGCATCG
FURIN	TCATTCATCTGTGTGTACCG
FXYD2	ACTGGGTTGTCGATGGACGG
FXYD2	CTATGAGACCGTTCGCAATG
FXYD2	CTGGACTGGCCTTCATCGTG
FXYD2	CCAGGCTTACCATAGTAGAA
FXYD2	GATCAGGCCCCCATTGCGAA
FYN	ACGGGGACCTTGCGTACGAG
FYN	TGGATACTACATTACCACCC
FYN	GTCCCCCGAATCATTCCTTG
FYN	TTGTCCTTTGGAAACCCAAG
FYN	AAACTGACGGAGGAGAGGGA
G6PD	CTTGAAGGTGAGGATAACGC
G6PD	AGAGGTGCAGGCCAACAATG
G6PD	TGCCCGTTCCCGCCTCACAG
G6PD	ACGGCCTCGTAGACGGTCGG
G6PD	GATGGGGCCGAAGATCCTGT
GAA	GAGGCCTGTGATATACTGCG
GAA	CTGGGTGGGAAGAAGCACCA
GAA	TCCAGCTAACAGGCGCTACG
GAA	CCACGATCATCATGTAGCGC
GAA	CTACAGCGTGGAGTTCTCCG
GABBR1	ACGGCGCGCAGTGTACATCG
GABBR1	CTGAGGTCTTCACTTCGGTG
GABBR1	AGATTGAGTATGTGTGCCGG

GABBR1	CATTACCGACCAAATCTACC
GABBR1	GGAGGACGTGAATAGCCGCA
GABBR2	GACCGTCCCATCAGACAATG
GABBR2	GGTGGGAGAGTACAACGCTG
GABBR2	GAAACTGCTTGTGATCGTGG
GABBR2	GGCGCAGGAGTGACTCGTTG
GABBR2	GGCGCGGCATGGCTTCCCCG
GABRA1	CCATTAGGTTATTTAACCGG
GABRA1	CAAACTCCTGCGGATCACAG
GABRA1	GAAGATATCAGTCTTCACTT
GABRA1	GCTACAACCACTGAGCGTGC
GABRA1	GCATTTGGAGGACTTCCCTA
GABRA2	GCTGGGCCAATCAATCGGAA
GABRA2	CCATATCTGTATCTGAGACA
GABRA2	AGATGCATATACAACTTCAG
GABRA2	TGTTAAGCCAGAATGAAACT
GABRA2	ATTCTTGACAGACTTCTGGA
GABRA3	CCACAATGGCAAGAAATCAG
GABRA3	CCCTGTGTCAGACACTGACA
GABRA3	TCTCGGAAAGAACAAATCCG
GABRA3	TCTGGCACTGATACTCAAGG
GABRA3	TCTTGATTCCCCTTGACCAG
GABRA4	GGAGTGTCCCATGAGATTGG
GABRA4	TCAATCTCAAAATTTCAATG
GABRA4	ATTACCAAATACAGTCCTAG
GABRA4	AATAACCCATCTTCCGTCTG
GABRA4	ATTGTCCTAGGAATACACAA
GABRA5	ACAACATCACGATATTTACC
GABRA5	TCACGGTCATTATGCAGGGA
GABRA5	TCTTTCAGAGCGCATCACTC
GABRA5	TGAACCAGTACCACCTGATG
GABRA5	GCAGCTTGAGGACTTCCCGA
GABRA6	AAGGCTATGACAATCGGCTG
GABRA6	TTTCTCTTAGGAGTATACGA
GABRA6	TCAATGCTGACTGTCCCATG
GABRA6	AAAGTGAAATCATATATACG
GABRA6	TTCAACTTCGAGTTTCCCTA
GABRB1	AAGGATATGACATTCGCTTG
GABRB1	CTCACAACTTTCGATCTCCA
GABRB1	GAACAGTTCTCTATGGACTC
GABRB1	TAGGGTAGCTGACCAACTCT
GABRB1	GGATGAGCAGAACTGCACCC
GABRB2	TAACCAGCGACATATTACTA

GABRB2	TTAAAGCTGAGGGATAACCT
GABRB2	GGATGAACAAAACTGCACCT
GABRB2	CACAATCAACACCCACCTCC
GABRB2	TTACCTCTGCGCACAGACAG
GABRB3	CGCCTAAGACCCGACTTCGG
GABRB3	GACCAGACGGTGCTCCACGA
GABRB3	CTTGGCAGATGGCTACACCA
GABRB3	CATACAGCACTGTCCCATCA
GABRB3	GTAAAACTCAATGTCATCCG
GABRD	TCCAGACGGTTACTCATCGG
GABRD	CGTACCTAGAGACACCCTGG
GABRD	CCCAGAGCGATGAATGACAT
GABRD	CAGCATCGACCACATCTCAG
GABRD	GGACCTGGCCAAATACCCCA
GABRE	ACCATGTCTAGGATAGAGAG
GABRE	ACGAGAAAAGGTGCCCAACG
GABRE	CCTGGCACGCTTACCAACTG
GABRE	GGAAACCCAGGAGAGCATCG
GABRE	GCTCCTCACCTCCAATGCCA
GABRG1	TTTAACGGTGAACAAAACCT
GABRG1	GGATCAGCCACTTCTACGGA
GABRG1	ACATCAGTTTCAATTACTGT
GABRG1	AGAACAACTGTCAGAATGCA
GABRG1	TCTGCGGAGTCAAAGTAGAG
GABRG3	CCGCAATTCTAAAACCGCAG
GABRG3	AGACGTTGTCACGATTTCTG
GABRG3	GTTGAGAATAAGAGTCACGT
GABRG3	AAAACCACAGTCAGTATACA
GABRG3	GCAAGCCCGAGAACAGGCAG
GABRP	GGGGAGTCAGTTCAACGTCG
GABRP	TTGCTCAGTACACCATAGAG
GABRP	GCGCTGTCGGAGGTATATGG
GABRP	ATAACACGGTCGTCACTCCT
GABRP	GCACCCAGAGGAACTCCACG
GABRQ	AAGGACGATTACTAGCAAGG
GABRQ	AAAGGATGGCCTGATTAACG
GABRQ	ACACAAAGAACAAGCACACG
GABRQ	TCCAAGAGGTAATAGTGGTG
GABRQ	GATGAGCAGCAGGATCACTG
GABRR1	GAGTTTGGATAGCATCTCAG
GABRR1	GCATGACCATCAGGGTAGCG
GABRR1	GAGCACGTTTGTGTGTCCAA
GABRR1	ATGACCATGATTTCAGCATG

GABRR1	GTAGAAAGCCAGTTTGGTGG
GABRR2	GGGTGTTGCAGGTATCACGA
GABRR2	ACGTGAAGTTAATGTACAGA
GABRR2	CTGAATCAGAAACTGAGACA
GABRR2	ACAGTCCTACCTCCGAAGGC
GABRR2	TGGAGAGCAGAAAACCCAAG
GAD1	CTAGCGTACGATACCTGGTG
GAD1	TTACATCGACATGCAACCAA
GAD1	CTATTCCATAAAGAAAGCTG
GAD1	GACATTTGATCGCTCCACCA
GAD1	GTACGATACCTGGTGCGGCG
GAD2	GTGGCTCAGAAGTTCACGGG
GAD2	TCTTGCAGAAACGCCAAAGT
GAD2	GGGGTCAAATGCTCCGTACA
GAD2	AAATGAGAGAAATCATTGGC
GAD2	TCCACTTTGGAGCAGCTGCA
GALNS	TGTTGTCATAAGGTCCAAAG
GALNS	TCAGCAAGATTGTCGGCAAG
GALNS	GGACGGCTACCCATCCGCAA
GALNS	AGCCATCCGGTCCAAATTCG
GALNS	GCCCGAGGCCCCCATCCCCG
GALR1	GGAGCGCCGCGAGTGCACGA
GALR1	CGTGGAGAACTTCGTCACGC
GALR1	GACGAAGGTGCACACCACGT
GALR1	GGTGGGCAGCGCGTACACGG
GALR1	GATGTCCGTGGACCGCTACG
GALR2	CTGGCGCGCCGTCGACCCGG
GALR2	GACACGCATGATCCTCATCG
GALR2	GGGGCACGATGACCGCCTCG
GALR2	GCCGAACACCCAGCCGTCCA
GALR2	GCACAGGATGAAACACAGGT
GALR3	GTAACGCCCGCGCCGCAGTG
GALR3	GTACGCCAGCAGCTTTACGC
GALR3	TACTACGGCACCGTGCGCTA
GALR3	CCAGGCCACCATCTACACGC
GALR3	GCAGGAGCCTGGCAGCACCA
GALT	GGCCATCCGAGCCAACGGAG
GALT	GCAGCGTTACATCCGACCAG
GALT	TGTCAGCTCACCGCATGAAG
GALT	CCTCACAAACCTGCACCCAA
GALT	CCTTCCTGAGTAGCTCCTGG
GAMT	CGACACGTACCCACTCTCGG
GAMT	CACCTTGTGTGTCTGCCGTG

GAMT	GGCCAGCGCGTGCATATAGG
GAMT	ATCAAAGTGACCGTCAGGCA
GAMT	GTCCGCTGCGTCGTAGGCCG
GAPDHS	ATGAGATCTCTGTCTACCAG
GAPDHS	GAATGATCCATTCATTGACC
GAPDHS	AGAGCCCACACCAGTCAGGG
GAPDHS	CTGGCCAGATTTGGACGCAT
GAPDHS	TCTTAGGAGGAGGAGTAGCG
GAS6	CCTCGAAGACCTGAAAGGCG
GAS6	TCTCCGTACACCAAAAACTC
GAS6	CAGTACTCACAGGCTGCACG
GAS6	AGCGGCCCGGTCATCAACCA
GAS6	GTATCATCTGAACCTGACCG
GATM	ACAACTATCAGGATGTCTCG
GATM	ACTTCAATGACCAGTCAATG
GATM	ATCAAAGACTACTTCCACCG
GATM	AAGTCAGCAGCATCAAAGCA
GATM	ATTCGTTGTAAGAAGAGACA
GBA	CGCTATGAGAGTACACGCAG
GBA	TTGGCTCAAGACCAATGGAG
GBA	AATCGGATATAACATCATCC
GBA	TGTGGTGAGTACTGTTGGCG
GBA	GTGGTGAGTACTGTTGGCGA
GCG	GAGGACAAGCGCCATTCACA
GCG	AGTGGTTGATGAATACCAAG
GCG	TCTGATCAGGATCACTGAGT
GCG	ACGTTCCCTTCAAGACACAG
GCG	TGATCCTGATCAGATGAACG
GCGR	AGGACGCAAACAGATTCGCG
GCGR	GCCATATTGCATGAACACCG
GCGR	CCAGCAGGAATACTTGTCGA
GCGR	CGCTTCGTGTTCAAGAGATG
GCGR	GCATGAACACCGCGGCCACA
GCH1	TCCCCGAGCGGGATCCGCCG
GCH1	TGATGAGATGGTGATTGTGA
GCH1	ACTGACCTGAGATGGTCTCC
GCH1	GCGAGAACCCCCAGCGGCAA
GCH1	GCACCGGCGGAGAAGCCGCG
GCLC	AGGCCAACATGCGAAAACGC
GCLC	CAATGTCTGACACATAGCCT
GCLC	ATTGCCCATTCCAAATCCCA
GCLC	AGAAATATCCGACATAGGAG
GCLC	CCCATACTCTGGTCTCCAAA

GCLM	ACGGGGAACCTGCTGAACTG
GCLM	ACTAGAAGTGCAGTTGACAT
GCLM	AATCAACCCAGATTTGGTCA
GCLM	TGATCTAGACAAAACACAGT
GCLM	CATGGGCACCGACAGCCGCG
GGCX	GCCTGCACGATGTCCACACG
GGCX	TGTGTGTATAAGAGGAGCCG
GGCX	GTCCGTTGGTCGATTCAGCA
GGCX	GCCAGTGCGGCCATCACGGT
GGCX	GAGCACTGCATAGTTCCAAA
GGH	TAGCATAATCTGAGCGTCTG
GGH	TGCCACAGATACTGTTGACG
GGH	AGTGTGATACCTTCACGGAG
GGH	AGACTCCAAGTACTTTACAT
GGH	GCTGGGCCTGCTACTCTGCG
GHR	TTCTGAATATCTGCATTGCG
GHR	AAATTATGGCGAGTTCAGTG
GHR	CATACATGAGGGTACCTCAG
GHR	AGTCTGCAAAGTGTTAATCC
GHR	GATACAATAAGGTATCCAGA
GHRHR	GTGAAGATTATCTACACCGT
GHRHR	GATAAAAGTGGTGAACAGCT
GHRHR	AGTCACATTCTGGGTGCATG
GHRHR	CGTGCTCTTCACTGGCACGT
GHRHR	GCAGCCCATCCCAGGTCGCA
GHSR	GGTACTGCCAGAGGCGAACG
GHSR	CATCTTCGTGCTAGTCGGGG
GHSR	AGAGCGCACCGCAAACTCGG
GHSR	CACCACGAAGAGTGCCACGC
GHSR	CGACGCCCAGCGAAGAGCCG
GIPR	GGGAATGACGAAAAGCGCGG
GIPR	GCGTGGGTGCCAACTACACG
GIPR	CCAGCAGACGTACATATCGA
GIPR	CTGGGAGCGCAACGAAGTCA
GIPR	GCGCTGGGAACGGTACCGCA
GJA1	TGGTAAGGTGAAAATGCGAG
GJA1	TGAGCCAGGTACAAGAGTGT
GJA1	AAGCCTACTCAACTGCTGGA
GJA1	GGAAACAGTCCACCTGATGT
GJA1	AGAGAAACTGAACAAGAAAG
GLDC	AGGGTAACTTCAGCTCAGTG
GLDC	TCATTCCGAAATCAGCACAT
GLDC	AAACCTGTTGAACACTTGAT

GLDC	TGGCAGCCATATTCGCCAAG
GLDC	CTTGGTGTAGAGATGCCACT
GLP1R	CTCTACGTGAGCATAGGCTG
GLP1R	GAAGCCGAGGAGGATCGCAG
GLP1R	CCAGCAGGCGTATTCATCGA
GLP1R	GAGTGCGAGGAGTCCAAGCG
GLP1R	GCATGAGCAGAAACACCAGG
GLP2R	TTATTACCTTCACTCCACCA
GLP2R	AGGGTACAACAAATAGCACA
GLP2R	TTGCAGCTGATGTACACCGT
GLP2R	ATGTCTGAGAGACTTACTCA
GLP2R	GCAGACGATAGAGAACGCCA
GLRA1	GATCCTGGCATCATATCCGG
GLRA1	TTCCATTGCTGAGACAACCA
GLRA1	CCAGGTCCAGAGAGTCGTCA
GLRA1	TCCAGAAGGAGATCCATGAG
GLRA1	GGACTTGAAGAATTTCCCCA
GLRA2	ATTCATCGTGTACCCAACTG
GLRA2	ATCAGTCACAGAAACGACCA
GLRA2	GGTGGTCATCGTTAAGACTG
GLRA2	TCTGCAAAGACCATGACTCC
GLRA2	GTACAGGTCTGGACATCCAT
GLRA3	GATATTCACTGTACGCGAGG
GLRA3	TGTAGTCATCGTTAGCACAG
GLRA3	AGATGAGGCACCCGTACAAG
GLRA3	AGAATAACAATCAGGAGACT
GLRA3	TATTCACTGTACGCGAGGCG
GLRB	AACAACAATGAGCAGAGTTG
GLRB	TTGCAACGTTGTGTATCCAT
GLRB	GGCAAGGGTTGTGCACTCAG
GLRB	CCTGTTCAAGATATTGCTAG
GLRB	ATAGGCTTCTTCCACCCACA
GLS	AAATTCAGTCCCGATTTGTG
GLS	GAGCACGCATCCGCAGCCCG
GLS	GATTGCGAACGTCTGATCCC
GLS	ATTGCTCCAGCATTTACCAT
GLS	CCACGGGTGGAGTCGCGCGG
GLS2	ATGGCTGGGAATGAATACAT
GLS2	AGGAATCCCCCATAACCCCA
GLS2	GGAAGAGATCTCGGTCCAAG
GLS2	CCTCAAAGATGCGATCCACA
GLS2	CATGAGCGAGATGCACCGCG
GLUD1	CCTGCTCCAGACATGAGCAC

GLUD1	CCGGCGCCACTACAGCGAGG
GLUD1	TCAATCAATGGCAGCAACAT
GLUD1	TCAGTGCTGTAACGGATACC
GLUD1	GCGCCACTACAGCGAGGCGG
GMPR	ATGTCCTGCTCCGACCTAAG
GMPR	AGGCAAATAAACTTAACCTG
GMPR	TGTGTTCAGGAAATTTGGCA
GMPR	TCAATGACGGCACTCAGCTG
GMPR	GCTTGAGGTCCGCATCTATG
GMPR2	GCTGCCAATATGGATACTGT
GMPR2	TACCCTTAAGTCTCGAAGTG
GMPR2	AGGCATATATACTTCACCTG
GMPR2	AGCACTATAGCCTCGTTCAG
GMPR2	GAGTAGTACACACAGAGCCT
GMPS	AATATTGCTGGATCAAACCA
GMPS	AAGTCATAGACCGAAGAGTG
GMPS	CCACCACTGAGTAAAACCTA
GMPS	GCCGTAAAGTACCTTGGGCA
GMPS	CTCAAGTTCTCTGTTCTGCA
GNMT	CTGCATACCCCACTTGTCGA
GNMT	CAGCCATGCCTTGTACTCGG
GNMT	AGGGCTCCCGGACCAGTACG
GNMT	TACGACCACATCCTCAGTAC
GNMT	GTACTGGTCCGGGAGCCCTT
GNRHR	GTCCTGCAAAGACACTACTG
GNRHR	GGAAGAAAGTAACCGTCACT
GNRHR	TGTGGAACATTACAGTCCAA
GNRHR	AGTCTCCAACAGGTTGGCTA
GNRHR	TGACAATCAGAGTCTCCAAC
GOT1	GGAGGTGTGCAATCTTTGGG
GOT1	AACTGGGATTGACCCAACTC
GOT1	CCATTCTCCAGCATATCGCA
GOT1	GTTGGTGAGGACACATAGAC
GOT1	GCTCACTGCCGACTTCAGGG
GP9	AGGGGTTCTGCGTCACATCG
GP9	GCCGCGCCCTGGAAACCATG
GP9	AGGTGGTCAAAGGCTCCCGG
GP9	GCCAGAGGCGCAGATAGGTG
GP9	GCAGTCCACCCACAGCCCCA
GPI	TTGGAGACATACCAGACGCG
GPI	TGGGAGGACGCTACTCGCTG
GPI	TGAGAAGATCAACTACACCG
GPI	TGACCCTCAACACCAACCAT

GPI	ATTGAACATCCGCTCCCGGG
GPR1	AGAAGACAGTCACCACTCTG
GPR1	CCTGTACTTCCGGGACACTG
GPR1	ACTGGCAAACATGTTCAACT
GPR1	CAGGCACCATGTTCTGACTT
GPR1	GCCACATAGGAGATGTACAG
GPR132	GGTAGTGAACGATCCCGACG
GPR132	GCTCCTGAAAATCCGGTGGT
GPR132	GGTGAAGCACTCGGCCATCG
GPR132	GTGTACGCGCTGGAGAGTCG
GPR132	CCTGGTCGTGGTGTACAGCG
GPR135	ACGCGCGCGTAGGTGTTCAC
GPR135	CATCGTGTCCACGCTCAGCG
GPR135	GGGAACCTGAGCGACGCAAG
GPR135	GCGCCGTGAGCAGATCCGAT
GPR135	GTCTAGCCTTGGCAACTGCG
GPR17	CAGATGCATCAGGAACACGT
GPR17	AAGTTGGTGATCAGACCTGG
GPR17	GCTGCAGGCAGACCACCGTG
GPR17	CACAATGGCCAGGAAACGGT
GPR17	GATGAAAAGCCACAGAGCCA
GPR3	GTCCTGCGAGAATGCGCTAG
GPR3	TGGATAAACCACGCCACATG
GPR3	CATTGTACAGAGAAAGGTAG
GPR3	CACTAAGGCCAGCATCACAT
GPR3	TGTCTCTGAATAGTAGGTGA
GPR39	TGGACTGGAACACGGTCCAG
GPR39	ACTCCATGGGCATGCCGATG
GPR39	CATGGGTACTGAGTACCCCC
GPR39	CAGCACCTGGGTGACCCGAA
GPR39	GTACCCATGGCAAACAGCAA
GPR50	GGTGCGAGGATCGTACTCGA
GPR50	AACATTCCGGACATTCATCG
GPR50	GGTAGAGGCAGATTTGCGAT
GPR50	TGAAGATCCGTTCGTACTGG
GPR50	GTAGATGGCCACCAGCATAT
GPR65	TGTGCGGCACAATAAAGCCA
GPR65	AACAGCCAAATACCGATCAA
GPR65	TTTCCAATATCCAGATGGAC
GPR65	GCACTCCCTTTGCACAAGGC
GPR65	GATGGTTTCCAATATCCAGA
GPR84	GCTCATAGCCAACCTCACAC
GPR84	GACAGCAGGTTAGCATCAGG

GPR84	CTTGGGGAAAAAGCTTAGGG
GPR84	CCAGGATATAAATAGGCCAG
GPR84	TTATGTTGCAGTTAGCTGGG
GPR87	GTATGCCATTTGACCCCCAA
GPR87	AGAATAAACTTGAAGTACCA
GPR87	AATATAATGAGATAAAGCAC
GPR87	CATTGATCGCTATCTGAAGG
GPR87	TATCAGCCCAAGGAACACGA
GPRC5A	ACAAAGTCTTCATTGCGACG
GPRC5A	GGGCATCGTCCTAGAAACGG
GPRC5A	CAGACCCAGAATCACCAACA
GPRC5A	CTTCGCCTTCATCATCGGAC
GPRC5A	AGTGAGCATGAAGGCCACCG
GPRC5B	AGGCAAACGTCAGCCCAAAG
GPRC5B	GGTGCAAGTCATCATCGCTG
GPRC5B	GATGGCGTCCAGGTCGCACA
GPRC5B	GCAGTACCATGTCGTAGATG
GPRC5B	GAAAACGCCAGCACATCCCG
GPRC5C	TCACCACACAGGCAAACACG
GPRC5C	AGGTCATCATCAATACAGAG
GPRC5C	CACCACCCATATGGCAACGG
GPRC5C	CAGTCCCAGGCACATCACCA
GPRC5C	GTGAAGATCACCCAGCCCCG
GPRC5D	AAAGAGAAAGTAGCGTACGG
GPRC5D	AGTCTTGGATCTTTCGCATG
GPRC5D	GACACCCTGCCAGCTCAATG
GPRC5D	GACTCCAGAATGATGCCCCA
GPRC5D	GTGACTCTCATCATGACCAG
GPT	TGAGTGGAACTGCGAACCCG
GPT	TGACACAGAGCGCACGAGGG
GPT	CCATGCCCAGCCTACCACGA
GPT	CCAGCGTGGCCGAGTAGAGT
GPT	TGACGATGCCAAGAAAAGGG
GPX4	AGAGATCAAAGAGTTCGCCG
GPX4	GAGCTGAGTGTAGTTTACTT
GPX4	CTTGGCGGAAAACTCGTGCA
GPX4	TTAACCTGGACAAGTACCGG
GPX4	GCTTCAGTAGGCGGCAAAGG
GRB2	AAGAAATGCTTAGCAAACAG
GRB2	AGTACTTCCCGGCTCCATCT
GRB2	ACGAGCTGAGCTTCAAAAGG
GRB2	GAAATACTTACTTGACAGAG
GRB2	AATTGAACTTCACCACCCAG

GRIA1	GAATTGATATATCTCGCCGG
GRIA1	TACATTTATGATGCCGACCG
GRIA1	AGCCCGAGACCCTGACACGA
GRIA1	CCTGGCCCAAGATAGCATTG
GRIA1	CATCACTGACAATCTCCAGA
GRIA2	GAATTGAAATCTCCCGAAGG
GRIA2	CAGATGAGACCCGACCTCAA
GRIA2	TGATCATGATAGATATCCCG
GRIA2	CCTCAGTGTGCCACTCGTAG
GRIA2	GGTCTCATCTGAATGACAAA
GRIA3	AGGAATCCAAGTGATCTACG
GRIA3	TACCTCTATGACACAGAACG
GRIA3	ATCCTAGGGAAACACTCAAG
GRIA3	ATATTTCCCGTCACCAACGA
GRIA3	GGACTAAAAAGAAGACCGCC
GRIA4	AATTGACTCTACGTCCATAG
GRIA4	AATCACTTTGGTACGAGAGG
GRIA4	GTTTGCTGGATCACTACGAA
GRIA4	GACCACGCTGACACCAATGT
GRIA4	TGGACTCTATGATAAGAGGT
GRIK1	AATCTTTGTTGTCCACCGAG
GRIK1	AATGACTTTCTCCCGCACGT
GRIK1	AAAATAGTTACCTCTCCGCG
GRIK1	ACAGTGGCGTAAACATGACC
GRIK1	TCTGAGGGAGGATATAGCAG
GRIK2	GTTGAGCCCTACCGATACAG
GRIK2	AATATACATCCAGATATCAG
GRIK2	TTTATGTCGATTACACTGCA
GRIK2	TTTGGATGTGATCAGTCTGA
GRIK2	GCACCAGGTCTAAAATGGCA
GRIK3	GCGAGTCGTCAGAGTCGATG
GRIK3	GCACACGGACACGATATGGA
GRIK3	CCAGAGGTACATTACCAGAG
GRIK3	CCTTGACCTATGACATACAG
GRIK3	GGAATCTTCGAGTATGCGGA
GRIK4	GGAAGGCAATGACCGCTACG
GRIK4	TGTTGACACGATCATCCACA
GRIK4	TCAGATCGGCCAGTGGCACG
GRIK4	GCATCAACCGCGCTCCTGAG
GRIK4	GGTGTCATCCAGCATGCGGA
GRIK5	CACGGCGTCAAACATCAGGG
GRIK5	TCCACCGTGAGCCATATCTG
GRIK5	GGAACCATACCATGTGCACT

GRIK5	TGGGTCTGCTCACCTCACGG
GRIK5	CCAAGGCCAGACGCTCACCG
GRIN1	CTGTCCTATGACAACAAGCG
GRIN1	CAAAAGCCGTAGCAACACTG
GRIN1	CATGTCCATCTACTCGGACA
GRIN1	CGGGCAGGCAGACATGATCG
GRIN1	GCACGAGCAGATGTTCCGCG
GRIN2A	TGAGGAACACCGTGCCATGT
GRIN2A	CATGATCTTCAGCATGACCG
GRIN2A	GCTCTACTGTTCCAAAGACG
GRIN2A	ATTGTAAAAGAAGGCCCATG
GRIN2A	GAAGTTCTCCTACATCCCCG
GRIN2B	CATAGACGGATGACTCCCGT
GRIN2B	TCTGTGCTGAAATGAAATCG
GRIN2B	TGACTGGCTATGGCTACACG
GRIN2B	AGACACCCATAAAGCAATGT
GRIN2B	TGATTTCCACCATCTCTCCG
GRIN2C	CTGCGCCAGAAGGTGCGCGA
GRIN2C	CAGTGCCCATCGAGAACCCG
GRIN2C	GTAGGAGAATTTGACCACTC
GRIN2C	CAAGTTCAACCAGCGCTCGG
GRIN2C	TGTCCTCAAAGACAATGCCG
GRIN2D	CTGCCACAATGACAAAATCG
GRIN2D	GAGCCTGCAGACCCTATCAG
GRIN2D	ACCTATGCAGACTCTCGCCG
GRIN2D	CACCGAATTATTGAACACCA
GRIN2D	GTACTTCATGAACATCACGT
GRIN3A	TTACTCTGATGGAACCACTG
GRIN3A	ACTGCTCCATGGCGAACTAG
GRIN3A	CATTTGGTTTGACTCCCAAG
GRIN3A	AGCACCCAACGAAGTTCAGG
GRIN3A	GCAAAGTGTGTGCCATACCG
GRIN3B	GGGGCTACGCCACTCGTACA
GRIN3B	CCATGACATTGTGCAACTGG
GRIN3B	GTGCCGAGACATGTGTACCT
GRIN3B	AGGTACAGCTCGAAGTCGAA
GRIN3B	GCTGCAGCTGCACTTCCTGG
GRK1	ATAGTGGCGAAGTTTAAGG
GRK1	CAAGAAGCTGAACAAGAAG
GRK1	CCCAGGTACTCCTGGAAGG
GRK1	GGAGCACCTGCACCAGAGG
GRK1	AAGGCCACACCTTTGACGG
GRK4	TGCCCATAACTACCTAAGAG

GRK4	GCGGCTTGGAAGATTTACAG
GRK4	GTTGGAACAGTCGGCTACAT
GRK4	CTTTGTGACAAGCAACCGAT
GRK4	CATTCTGTCACAACATCTGG
GRK5	GTGACAAGCAGCCAATCGGG
GRK5	GGCCTGAGCCCCGACTACTG
GRK5	TATGCGGCAGAGATCCTCTG
GRK5	TTGGCTGTGAAGATCCCCGA
GRK5	GCTTACCTATGGTCCTTCGG
GRK6	GGCCACGCTCAGGTACTCGT
GRK6	AAGTGACCCCGGATGACAAG
GRK6	GTGCTGACACTGATGAACGG
GRK6	CTGCGATCATCTCGTACAGG
GRK6	GCAAGAAGCTAGAGAAAAAG
GRM1	TGAGGCTGGACACTAACACG
GRM1	CGGCCGAGAAAGTGCCCGAG
GRM1	AAAGATTAGGGTGACAAACA
GRM1	GATTAGTGTGCAACTAACCC
GRM1	GGAGCAGTATGGCATCCAGA
GRM2	GGGTCGCATAAGAGCCGTCG
GRM2	CCAGCCCTATGAGTACCGAT
GRM2	GTCGCCCTCAGACGCCACAG
GRM2	TGGGCGCTATCGCTACCAGA
GRM2	CCACACCCTCAAAGGCCGCG
GRM3	TCATCAGCCAGGTATTCGTA
GRM3	TGACCCCTGCAATGAGAAGT
GRM3	TTCATCAATAGCAAACAACA
GRM3	CCAGTGACCAACTTTCAAGT
GRM3	GGGCGAATCAATGAAGACCG
GRM4	GGATCGCATCAACAACGACC
GRM4	CCCAAGAGGATGTCCGTACG
GRM4	AGACGTGTCCCTATGACATG
GRM4	CCACCCTTACAGGCATCGCA
GRM4	CTACGACTTCTTCTCCCGCG
GRM5	CCAGTACAGTATCTTCGATG
GRM5	CCATGAAGCCAATTGATGGA
GRM5	ATCTGTAACACCACCAACCT
GRM5	CAAAGACCAACCGTATTGCA
GRM5	GCTCCAATAATGATGTCACC
GRM6	CTTCGTTCAGATCTCCCGAG
GRM6	GGAGAAGAAGTCATAGCGTG
GRM6	GCTCGGGGTCGGCGTTGACG
GRM6	CTCACCCATCTTGAGCCTGG

GRM6	AGGACAGGCGAGGAACGCAT
GRM7	TCCGGCTTGACGAAAACCGG
GRM7	GATCTCTGTGCTGACTACCG
GRM7	TGGTACTGGTAACCATCGCA
GRM7	TTATGTGTCTACCCTCGCAT
GRM7	ATAACTTCCTTCCGATGCGA
GRM8	GGTGTTATCACTTAGCTCTG
GRM8	TGGTACTCATTCGTGGACAA
GRM8	TGTCCCTAGAGCACGTGTCG
GRM8	AGCACAGAGTACAAAGTCAT
GRM8	GTATGCCCATTCCATACGGG
GRN	TTTACGTGTGACACGCAGAA
GRN	ATCGACCATAACACAGCACG
GRN	CTGCTGCCGTCTACAGTCGG
GRN	CCCTGCCCAGAGGACTAACA
GRN	GGTCCAGGCAGCAGGCCACA
GRPR	ACGATGACTGGTCCCACCCG
GRPR	CTGGAGCACACGTTATTAGG
GRPR	TAAACAGAGATGATCGACAG
GRPR	GAGTGCTTACAATCTTCCCG
GRPR	TGATAACCCCATAAACTGCA
GSK3A	CCTCCCATAACTCTGACCGA
GSK3A	GCCCGAGACAGTGTACCGGG
GSK3A	TCAGCCTCACAATATTGCAG
GSK3A	AGGAGACATTGGGCTCCCCT
GSK3A	GGAAGCTAGTGCCTGCGCCG
GSK3B	ATACCTTGACATAAATCACA
GSK3B	CAGTATCAGGATCCAACAAG
GSK3B	GTGGCTCCAAAGATCAACTC
GSK3B	AGGTCCTGGGAACTCCAACA
GSK3B	CTTCCTTTAGGAGACAAGGA
GSR	TGGGCGGGTCCCGAATACCA
GSR	GGGCTTGGGATCACTCGTGA
GSR	TATTTGCCAATAGGTCAAGG
GSR	TCAGTGATGTCTTAGAACCC
GSR	GTACACATCCAACATTCACC
GSS	ACCTGTAGACTGTACTGACG
GSS	GCTACTGATTGCTCAAGAGA
GSS	TGAGGGGAAGAGCGTGAATG
GSS	AGATATCTTCAAATGTTCGT
GSS	GGAAAGAGTTTGCTCCAGGA
GSTM1	ATGGACAACCATATGCAGCT
GSTM1	GCGGGCAATGTAGCACAAGA

GSTM1	AGCTCTACTCAGAGTTTCTG
GSTM1	ACTGAAGCCAAAGTACTTGG
GSTM1	GCGGACCCTCGCTCACCCCG
GSTO1	TGAAGGCCAAGGGAATCAGG
GSTO1	ACCTGGATGAAGCATACCCA
GSTO1	AGAATTTACCAAGCTAGAGG
GSTO1	GTCTAGGTGCCATCCTTGGT
GSTO1	AGAACTGGCACCAGACCAAA
GSTP1	CATGGTGAATGACGGCGTGG
GSTP1	AATACCATCCTGCGTCACCT
GSTP1	GGCATGGTCACTTACGCAGG
GSTP1	TCAAAAGGCTTCAGTTGCCC
GSTP1	GCATGCGCAGGGCCGCGCAG
GSTZ1	GAAAGGCATCGACTACGAGA
GSTZ1	GTCAGAAATCATACGCACGC
GSTZ1	CTGTCTGTCCTGAAGCAAGT
GSTZ1	CTGAGGCAGAAGTCGCGGAG
GSTZ1	GTACAAGTGCCACACCTGCA
GUCY1A2	CTTGGTTCTAATCACAAACG
GUCY1A2	AGAGTCTGCTGTATCGTCTG
GUCY1A2	GCAAACCCCACAATATGGTG
GUCY1A2	AACCAAAATGACATCTCGGG
GUCY1A2	CTACCTGGAGACCAGCCCGG
GUCY1A3	CGATGTGGGAATCACCAGCG
GUCY1A3	TGTGAATTGGGATGTCTGAG
GUCY1A3	CCAAAGTGTGAAGATAGACT
GUCY1A3	CTTATCCAGGCATAGAATGG
GUCY1A3	CAATCATTATGGAAGCAGGG
GUCY1B3	TGAACCTGGACGATTTGACA
GUCY1B3	ACAGTGGCACAACAAATCCA
GUCY1B3	CACCTTGCTACCATCTACCC
GUCY1B3	GACCTAGTGGTCACTCAGTG
GUCY1B3	GATCCGCAATTACGGCCCCG
GUCY2D	TTTGGATCGAACCAGCACGA
GUCY2D	AGCATGTCAGACATTCGCAG
GUCY2D	GCGTAGTGGATCGTGTCGAA
GUCY2D	GAAGTCACGGAACTGCATAG
GUCY2D	GCAGGTCCCTGGCTTCTGCG
GUCY2F	TGGAGCTGCTACGTTTCGGA
GUCY2F	TGCAGTGTTGACCATTACAG
GUCY2F	TGGAGCTCTCAACAGTTCAG
GUCY2F	TTTATTGGACCTACCAACCC
GUCY2F	CGACCCCTACAGGTAAGCCG

GUK1	TCTCTCTGCTAGATACCACG
GUK1	ACATCAAGGCCACCGATCTG
GUK1	GGCATGCTCGATGAAGTCGC
GUK1	CGGGGAACCTGTATGGCACG
GUK1	AGGCTGCTCCAGGAGCACAG
GZMH	ACAATATCAAGGAACAGGAG
GZMH	CTTTCAGAGGAGATCATCGG
GZMH	ACTGAACAAAGGCCATGTAG
GZMH	GCACAAAGTCCTTTCTCACT
GZMH	TCAGAAGAAAGGCCAACAGG
GZMK	AGTAACAGTGACTTCTCGCA
GZMK	TGTTCTGATTGATCCACAGT
GZMK	TTTGGTTCCAGATCTAAGAG
GZMK	GTGTGCGCCTAAAACCACAG
GZMK	TGAGGCCTCCAAACAAACAC
GZMM	CAGGACACCCCCGCACAGGT
GZMM	TTTGGGACCCAGATCATCGG
GZMM	TGCACACCTGAAGCAGCGCG
GZMM	CAGCCGGACCATCCGGCCGT
GZMM	TGATGTGGAAGGTGAGACCG
HABP2	GCGATGGCTACTCTTACCGA
HABP2	CACAGACGTTGCCTACCCAG
HABP2	AAGATCAAGAGAATCTATGG
HABP2	TACAGTGCAAAATACGTGCA
HABP2	GTAACTTTAATAAAGCACCA
HAL	AGATGGAGACCGTCTGACCA
HAL	AAGCAACTTCAATTTGCCCA
HAL	GAAGTGCGCGTCATCCACGG
HAL	TTGTATCATTCACCACACCA
HAL	CTATAGGTGCTAGAAGCCCA
HAS1	GGACCCCGCCACGTACGTGT
HAS1	CACCAACAGCCCCTACCCGG
HAS1	CGCTGATGCAGGATACACAG
HAS1	CCGCTGGCCTCCGATCGCTA
HAS1	CATGGTCGACATGTTCCGCG
HCAR2	AAGATGCCGATCCAGAATGG
HCAR2	ATGTTGGCTATGAACCGCCA
HCAR2	TCCTGATGGACAACTATGTG
HCAR2	CAATAGTGATGCCCCACAGA
HCAR2	TGAAGAGCATCAGCCGGCAA
HCK	ATCCGGACCCTGGACAACGG
HCK	AATGGCCTCGTAATCATACA
HCK	ATGTATTGCCTCCGACCTGG

HCK	CCAGCTTGAGGGATTCCCGA
HCK	CTGTCTCCAGAGAGTCAACG
HCN1	AAGACACCCAGCAATCTGGT
HCN1	ATGAAGCCGTACTGCCGCCG
HCN1	CTTCAACTGTCGGAAACTGG
HCN1	CAGAGACTGGATTAAAGCGG
HCN1	CTTCATCTCATCCATCCCAG
HCN2	CACGTTGAACACGATCCACG
HCN2	TCTGCGCACGTGGTTCGTGG
HCN2	GGTGACTACATCATCCGCGA
HCN2	AGTACAGTTCACTCCACGAG
HCN2	AAGCTGGCCTGGCTGCCGCG
HCN3	GTGGCTGCCGAACACCCGAA
HCN3	TCATCCGCTACATACACCAG
HCN3	CAAAGTAGGATCCATCGGTG
HCN3	CAACTTCCGAACGGGCATCG
HCN3	CTAGGAAGATGTAATCCACA
HCN4	TCATTCGATATATTCACCAG
HCN4	CGGACACCTGCATGACTCCG
HCN4	CGAACAGGAGAGGGTCAAGT
HCN4	GGAGTACCCCATGATGCGAA
HCN4	TTTCATTTCCTCCATCCCCG
HCRTR2	AGTGATATCCACGACCAGTG
HCRTR2	ATCACACACCGTAAAGAGGG
HCRTR2	AATTAGTTTGTGTGGCAGTG
HCRTR2	GAAGTCCCGGATGAGCGCTG
HCRTR2	GATTGCATACCACCGATCCA
HDAC1	CATCCGTCCAGATAACATGT
HDAC1	TGAGTCATGCGGATTCGGTG
HDAC1	GCACCGGGCAACGTTACGAA
HDAC1	GGAGATGTTCCAGCCTAGTG
HDAC1	GCACCATGCAAAGAAGTCCG
HDAC10	AGTCAGATGCAGACGCAGTG
HDAC10	AGGATTTGACTCAGCCATCG
HDAC10	CCGCAGCCCTGGATCGCCTG
HDAC10	GACAACGCCGGATATCACAT
HDAC10	GGGCAGCTCTCACCTCACCA
HDAC2	GATGTATCAACCTAGTGCTG
HDAC2	TACAACAGATCGTGTAATGA
HDAC2	CCTCCTCCAAGCATCAGTAA
HDAC2	TCAAAGAGTCCATCAAACAC
HDAC2	TGGGTCATGCGGATTCTATG
HDAC3	TCATCAATGCCATCCCGCAG

HDAC3	ACCTGGAGCACAATGCACGT
HDAC3	TGGGTCAATGCCAGGCGATG
HDAC3	GTCAGCCCCACCAATATGCA
HDAC3	CTATTCCCATACCTGTGCCA
HDAC4	CTTACCCGTACCAGTAGCGA
HDAC4	GCATCAGCGTGTCATACACG
HDAC4	GGGGCTGACTTACCGCAGAG
HDAC4	GGAGCCCATTGAGAGCGATG
HDAC4	GTCGACCTCCTATAACCACC
HDAC5	ACGTTCACCCGTCACTAGTG
HDAC5	TATGCCCTGTACTTACAGTG
HDAC5	GCCGGGTGCGCTGTTACACA
HDAC5	AGGCCTGCTTAGCAAGTGCG
HDAC5	GAGCCCCCCGTAGCTCCACA
HDAC6	TGTGCTGAGTTCCATTACCG
HDAC6	AGGACACGCAGCGATCTAGG
HDAC6	GCTTCCAGTGCTGAGTACGT
HDAC6	CCTCTAGGATAAGGATAATG
HDAC6	CTATTGCATGTTCAACCACG
HDAC7	TGCAGTCGGTCCACTCTGAG
HDAC7	GTTACCTGTAGGGAATGCCG
HDAC7	AAGGACTGGGCAAAGTGGAA
HDAC7	GACCTGGAGACAGATGGCGG
HDAC7	AGTCCTTAATGACCACCGAG
HDAC8	ATTTGAGCGTATTCTCTACG
HDAC8	ATAGTCAAATATCCCTTCAG
HDAC8	GTAAATGTGCCCATTCAGGA
HDAC8	GCTGCAGATAAGCATCAGTG
HDAC8	GGAGGAGCCGGAGGAACCGG
HDAC9	AGCTTTGATCCAATGATGTG
HDAC9	AACAGCATGAGAACTTGACA
HDAC9	TTTCCCTCTAAAGTAACATG
HDAC9	GAGAGCGCACGTGTGTGCGT
HDAC9	CTATCTTTGCCTCTGAGAGG
HDC	GATGACAACTTCTCACTCCG
HDC	GTTTAACAGAGCGAAACCGT
HDC	CTGCATAAGCAGCATCGATG
HDC	GGTAGTAGGCGTGCATATGG
HDC	TCACCTTTAATCCTTCCAAG
HEPH	AGCCACTCGAGTCTACTACC
HEPH	TTCTCATAAAAGACCCCATG
HEPH	GGACAGATGCTGACTACCCG
HEPH	GGCACAGAGACTGATGTGCA

HEPH	AGGGCTTCCAAGACTCCAAT
HEXB	AGAGTTTGGGGAGCATTACG
HEXB	ATTGAATATGCCAGATTACG
HEXB	AGGGGCCCGCCGTGGAATTG
HEXB	ATTAAACTTATTAAAAGCCA
HEXB	GCTGATGTAGAAGTTCTCCG
HGF	TCTTTACCCCGATAGCTCGA
HGF	AATGTGCTAATAGATGTACT
HGF	CTGGACTAACATGTTCAATG
HGF	TGAATGCATGACCTGCAATG
HGF	GAGGACATGCTGCAGCAGCA
HGFAC	GGAATCCGGACAATGACGAG
HGFAC	GAGGAGCCGCCGATGATACG
HGFAC	AGCACTCGCACTGTTCCACG
HGFAC	CACAACTCACAACTACGACC
HGFAC	CTGCTCCTCCTAGAACCGTA
HIPK2	TGGACTCAAGCGTAAGAGCG
HIPK2	ACTGGGCGAATGTATTTGAG
HIPK2	TCGGGTGAATATGTATGACA
HIPK2	TAAACCAAGGATGATCTCAG
HIPK2	CATCACCTACCGGCAGCAG
HIPK3	ATACCATTTAATAGACCTCG
HIPK3	TAAAGTAATAGACTTTGGGT
HIPK3	GCAATGTTGCAAACCAACAT
HIPK3	TAGAAGTGAGCATATTAGCA
HIPK3	CTACCATTCACATAGGTCCG
HLCS	TCAGGGATCCTCTTATGCAG
HLCS	TTGGTGAAGAACCCAAACAA
HLCS	GGGAGTCGGAGCCCACATAG
HLCS	ACAGTGCTCTCAGAGACCCG
HLCS	GTACCAGTCCATTATCCATG
HMCN1	TGGTGGTTAATAACCCGGTG
HMCN1	ATAGTTACACCTACAATTAG
HMCN1	TGTTTATCGTGGGTTCACAC
HMCN1	AATGTTGAACTTCTAGACAG
HMCN1	AGAGCTGAGGAAATTCCCGA
HMGCR	AGAGCATCGAGGGTAAACGT
HMGCR	GCCAAATTGGACGACCCTCG
HMGCR	ACAGATACTTGGGAATGCAG
HMGCR	TTGTAGACGTGAACCTATGC
HMGCR	AGAGAGATAAAACTGCCAGA
HMGCS1	AGCAGCGGTCTAATGCACTG
HMGCS1	GGAAATGCTAGACCTACAGG

HMGCS1	TAATAGCTCCTCTTACCCAA
HMGCS1	ACAACTAATGCATGCTATGG
HMGCS1	GCAAAAAGATCCATGCCCAG
HMOX1	GGGATGACCTCCTGCCAGCG
HMOX1	GGCCCCCAGACAGGTCACCC
HMOX1	CAGAGAATGCTGAGTTCATG
HMOX1	GGCCACATAGATGTGGTACA
HMOX1	CTATGTGAAGCGGCTCCACG
HNF4A	GGGACCGGATCAGCACTCGA
HNF4A	CAGGTGTTGACGATGGGCAA
HNF4A	CAGCTCGTCAAGGATGCGTA
HNF4A	CCAAGGGGCTGAGCGATCCA
HNF4A	GCTTCTTCCGGAGGAGCGTG
HNF4G	ACAGTTGACACCGTTGTCTG
HNF4G	CAGCTCATCTAGAACACGAT
HNF4G	ATGTGTACCTGATCATCCAA
HNF4G	TCGGCAATGTGTTGTTGACA
HNF4G	TTATGTCAGTGCTTGACCCA
HNMT	ATTAAGATTCTAAGCATAGG
HNMT	AGCTGCCAGGCATAATAGGA
HNMT	AATAATGAGCATCTTAGCAT
HNMT	AAGCAAACTTTACGTTCTCG
HNMT	AATATGTTGAATCTTTCCGG
HPD	TCAGCCATGTAATCAAACAA
HPD	GTGGAGAAGATGAACTACAT
HPD	AGATGGTGTCCGCCTCCGAA
HPD	CTTGCTGCAGTAGAATGACG
HPD	GTAGTTCATCTTCTCCACCA
HPN	AGAGCAGGGATCCCCCACAG
HPN	GAAAGGGAAGATAGCCCCCG
HPN	TGGTCTTTGACAAGACGGAA
HPN	GGCGGGCGCCAATGGCACGT
HPN	GATGGCTGTCAGAAGTAGCA
HPRT1	AATAAATCAAGGTCATAACC
HPRT1	CTGTCCATAATTAGTCCATG
HPRT1	ACTAGAATGACCAGTCAACA
HPRT1	CACAGAGGGCTACAATGTGA
HPRT1	TTATGCTGAGGATTTGGAAA
HRH1	CGATACTTAAGGTACCTGAG
HRH1	TGTATGCCGTACGGAGTGAG
HRH1	CTTGTCCTCTCGGCGCACCG
HRH1	GTCACCATCCCAAACCCCCA
HRH1	CGATCAAGTCCGCCACCGAG

HRH2	AGAGATGGCGACCCGAACTG
HRH2	TGCACATGATCAGTAGCGGG
HRH2	TCTTCATGATCAGCCTCGAC
HRH2	GAGCAGGTCAGTGATAGCCA
HRH2	GCTATCACTGACCTGCTCCT
HRH3	GGGCTGGGGATTTACCGACG
HRH3	GAGGCTCGAGTCGGCCACGA
HRH3	GGGCGTAAAGAACTCCAGGG
HRH3	GTGACACGCGGCGGGCAGTG
HRH3	GCAGCCCAGGACTCACCGCT
HRH4	TTATAGAACTCAACATACTG
HRH4	CCCATTCGAACAGCGTGTGA
HRH4	TGAGATGATCACGCTTCCAC
HRH4	TCTAGTTTCAGAGTCTTGGA
HRH4	GCCATCTCTGACTTCTTTGT
HS3ST1	GGGCGACGTGAAATACGCGG
HS3ST1	CAAGCCCTACCCGTCCATCG
HS3ST1	CGTTTGGGGCCACGCCATCG
HS3ST1	CGAGGTCCACTTCTTCGACT
HS3ST1	TGAGGTACCAGCCCAAGCCG
HSD11B2	CTGGCACAGCCAGTCGAGCG
HSD11B2	GGCCTATGGAACCTCCAAAG
HSD11B2	GCTGTTCAACTCCAATACGG
HSD11B2	CCGCGTGCTAGAGTTCACCA
HSD11B2	CCTGCCGGTGGCCACTCGCG
HSD17B10	CTGGTGGCGGTAATAACCGG
HSD17B10	CAACTGTGCAGGCATCGCGG
HSD17B10	CCACGGCGGAGCGACTTGTG
HSD17B10	TGTTGATGATGACCCCACGT
HSD17B10	AGTTGACAGCTACATCCACA
HSD17B6	ATGTGAGTGGCTGAACACTG
HSD17B6	CCTTTGGTGAGGAGAGCACG
HSD17B6	AAAATCAGCATAGTTGAACC
HSD17B6	TAAAGACATACTTGTCTTGG
HSD17B6	GCATCGCTGCAGCTACTCAG
HSD3B2	AAGACCCACATATATCTATG
HSD3B2	CCTTCTCTTCCACCAACAGG
HSD3B2	GGCAGGTACCCAGCTACTGT
HSD3B2	CTTCAGACCAGAATTGAGAG
HSD3B2	GTTCCTTCTCTTCCACCAAC
HSP90AA1	GATCTGTCAAGCTTTCATAC
HSP90AA1	TCTCACGGGATATGTTTAGA
HSP90AA1	CAGTGAGGACAGACACAGGT

HSP90AA1	GATCAAAAGGAGCACGTCGT
HSP90AA1	TCTCTTGCAGGTGAACCTAT
HSP90AB1	CATTAGAGATCAACTCCCGA
HSP90AB1	CTCACACCTTGACTGCCAAG
HSP90AB1	ACTCTTCTAGGTGAGCCCAT
HSP90AB1	CACTTTCTCTGCCACCAAGT
HSP90AB1	ACTTTGGTACCCCTGCCAAT
HSP90B1	TCTCGCGGGAAACATTCAAG
HSP90B1	CTTATCTGCTACAAGGAAGG
HSP90B1	AGACCACGTGGAGCAGATGT
HSP90B1	TTCTCTGGTCATTCCTACAC
HSP90B1	GTACCCACATCTGCTCCACG
HSPA5	CAGACGGGTCATTCCACGTG
HSPA5	AATGGCAAGGAACCATCCCG
HSPA5	GGTGAGAAGAGACACATCGA
HSPA5	CGACATAGGACGGCGTGATG
HSPA5	CGTTGGCGATGATCTCCACG
HSPG2	ATTGTGCGCTGTGACGAGCG
HSPG2	TGACTCGATCCGGACCGTGG
HSPG2	GCGAAGGCACAAATCCACGG
HSPG2	ATCACATGGTACAAGCGTGG
HSPG2	GGAGCGAGTGAAATTCACCA
HTATIP2	GCTGGCGCCCAAAATAAAGA
HTATIP2	CCTTGCCTAGAATCAAGAAG
HTATIP2	TACCACCAGAGGGAAAGCTG
HTATIP2	AAGTCACGCTCATTGGCCGG
HTATIP2	GAGGAAGCTCACCTTCGACG
HTR1A	TTGGTAGCTGACGGTCACGT
HTR1A	TGCTAATGGTGCATGCGTCG
HTR1A	AGGTGCAGCACAGCACGTCG
HTR1A	GTTGAGCACCTGATACAGCG
HTR1A	GGGCACGCTCATCTTCTGCG
HTR1B	GAAGTAGAAAGCACCCACCG
HTR1B	GGCGATCAGGTAGTTAGCCG
HTR1B	CAGCTAAAAGGACTCCCAAG
HTR1B	GCAGAGGATAAGTTGGCTTG
HTR1B	GTACACTGTGGCAATCACAA
HTR1D	AAGCGCTTCCCATAGAGTGA
HTR1D	CCCTGGAATACAGTAAACGC
HTR1D	GGGAGATCTTGAGCGCCTGG
HTR1D	AGGATGATGAGCAACACCGA
HTR1D	TTACCAAGATGGAAACCAAG
HTR1F	TCGCTGCAATTATTGTGACC

HTR1F	TGGTGCCTCCAGAATAGAGG
HTR1F	TCCAAATGTTGAGTAAATGG
HTR1F	TCTCAGCTATAGCTTTGGAT
HTR1F	AATAATTGCAGCGATCACAA
HTR2A	TCTGCCTCATAGGGTACCGG
HTR2A	AGGGAGCCAGGGTCCTACAC
HTR2A	GATTCTGGATGGCGACGTAG
HTR2A	TGGGCTACAGGACGATTCGA
HTR2A	GCACAAAGCTTGCTCGGCAG
HTR2B	AGCATTCATCAAGATTACAG
HTR2B	TTGCTCGTCACCGGAAAAGG
HTR2B	AGGCAGGACATAGAACAAGT
HTR2B	AAACAGATTGTTGAGGAACA
HTR2B	TGAAACAGCCAGAATAACAA
HTR2C	TCTGCGCTATATCGCTGGAT
HTR2C	CAAGCTTTGATGTTACTGCA
HTR2C	CGTCCCTCAGTCCAATCACA
HTR2C	GGGCACAAATATCTAGGTAG
HTR2C	GGACGCTTCAAATTCCCAGA
HTR3A	AATGACGTCAATGGATACGG
HTR3A	GATGCCGAATATACACGTAC
HTR3A	CCCAGGTGGTCATCCGCCGG
HTR3A	AGGAGTGTCTTCATGAACCA
HTR3A	GTTCATGAAGACACTCCTGT
HTR3B	CAGACTCACGACATAGACCA
HTR3B	ACCTGTTTACAACTGGACCA
HTR3B	AGTTCACATAAACATAGGGA
HTR3B	TCTTGAACACAATCCTGGCT
HTR3B	GATGAGTTCACATAAACATA
HTR4	CATTAATGCGATGCGCAGAG
HTR4	TCTGGACGTCCTGCTCACAA
HTR4	AGCATAGCACTCATCGCATG
HTR4	CCTTAGCTGTGACATAGATG
HTR4	CCAACTCTCAGATAGAAAAG
HTR5A	AAGGCTGCCAAGTTCCGCGT
HTR5A	ACACGGCGTAGGAAGGCTCG
HTR5A	GGTGAGCGCGATCATGACGT
HTR5A	GGTGGAAGGTGCGTACACGG
HTR5A	GGCTGTGGTTGGTCTCCAAA
HTR6	AGCTAGCAGGATCCTGCAGT
HTR6	GCAGCACATCACGTCGAAGG
HTR6	CAGACGTGAAGAGCGACACC
HTR6	GCCCAGGACCCCACGCCCAG

HTR6	GTGAAGCATATGGCACCCGA
HTR7	GGGGATATAAAATGCCACTG
HTR7	GTCAATGCTGATCACGCACA
HTR7	TGAGCCCATCCAAAGAGTGG
HTR7	CAGCCGGAGGCATTGTCCGG
HTR7	GGTGCCCGTAGAGGTCCGGG
HTRA2	CGTTCGAGATAGGGACCTCG
HTRA2	ATGAAGTTGTACTGACTCCG
HTRA2	GTCCGTGTGAGACTGCTAAG
HTRA2	GGGACTCCCCCAAACCAATG
HTRA2	CAGGATCTCGATATAGACCA
HTRA3	ATGAAGTTGAACTTGTAGCG
HTRA3	GATCATCACCAATGCCCACG
HTRA3	CAAAGACATCGACAAGAAGT
HTRA3	GGCACGTTGCGGCCAAACAG
HTRA3	GTTGGCATAGGTGTGCCCGT
HUNK	TGAACGGATCCGAGTAACCC
HUNK	ACTGGAGCGCTATTTGTCAG
HUNK	AAGTCGCGGAGCCGCTCGCG
HUNK	AGGCAGCGTCCCGGTCAACA
HUNK	CCTATGTCACCAAAAACCTG
HVCN1	TTAAGGCACTTCACGGTCGT
HVCN1	TTCCTCGCCTGAGACTGGTG
HVCN1	CAAGAATAACTATGCTGCCA
HVCN1	GGAAACTGTTCAGCTCCCAC
HVCN1	CATGCCCCTGAAGTCAAGGG
HYAL1	GGTGTTTGCATTCCAGACGG
HYAL1	GGATGTCAGTGTCTTCGATG
HYAL1	TGTGGCCGAGGCATTCCGTG
HYAL1	TGGGGCAGACCACCAAACAC
HYAL1	GATGTATGTGCAACACCGTG
HYAL2	CTTCCGGCAGAATCGAAGCG
HYAL2	GTCGTGTGAAGACGTAGACT
HYAL2	GGAGCACTACATTCGGACAC
HYAL2	GACGTGCCCACACAGGACTG
HYAL2	GCATCTTCCGGTGTGCCCAA
IAPP	TGAAAGCTACACCCATTGAA
IAPP	CCAGTCATCAGGTGGAAAAG
IAPP	CAGGCGCTGCGTTGCACATG
IAPP	CAATGGGTGTAGCTTTCAGA
IAPP	CCATGTTACCAGTCATCAGG
IARS	GCTTGTTGACAACTATGTGA
IARS	GAGTTGTTTGAAGACCCACC

IARS	GTTCTGGATTAAGAAGCGAT
IARS	AGTCATCGCTGACCCACAGT
IARS	TTTCTCGTAGATGTTGCCAG
IARS2	GATGACGGAGACCAAAACAC
IARS2	TAGTGGCAGATACCGGGACA
IARS2	ATAGCTATCCGTATGACTGG
IARS2	GCAAACGGTGACCCTCATGT
IARS2	GATGGACCTCCTTATGCAAA
ICAM1	TGACGTGTGCAGTAATACTG
ICAM1	GCCCGCTGAGGTCACGACCA
ICAM1	CGGGCTGTTCCCAGTCTCGG
ICAM1	TGCAGGGACTCCAGAACGGG
ICAM1	TCAAAAGTCATCCTGCCCCG
ICK	GGAATGCATGAACCTTCGGG
ICK	CTGGCTTAATATAAGGAGGT
ICK	CACCGCCCAGACGTCAATGG
ICK	CACGTACCCCTACAAAGCAG
ICK	AATGCTTCTTCCCAGCAGGA
IDE	TGCTGCTGATGACTTATCCG
IDE	TTGCAACTTTCATCGAACAA
IDE	ATCTTAGATGTATAGCCCCG
IDE	CATTAATGTGGACTTGACCG
IDE	CTTCAGTCATCAGCAAGCGG
IDH1	ATGTAGATCCAATTCCACGT
IDH1	CCCATCCACTCACAAGCCGG
IDH1	CAAGCTATGAAATCAGAGGG
IDH1	TACCTTCAAAGTTATGTACC
IDH1	TTATCTGCAAAAATATCCCC
IDO1	ATCCCAGAACTAGACGTGCA
IDO1	ACCAGACCGTCTGATAGCTG
IDO1	GATACTTACTCATAAGTCAG
IDO1	AGAACGGGACACTTTGCTAA
IDO1	TTTGCCCCACACATATGCCA
IDS	GTGTGGCTTATGATACCCAA
IDS	CCAGCTATACGGAGAATCAT
IDS	CGCCCTCGGATCCGAAACGC
IDS	GATGTGCTGGATGTTCCCGA
IDS	TGCCAGTGAGGAAAGAAACG
IFNAR1	GTACATTGTATAAAGACCAC
IFNAR1	ATAATTGGATAAAATTGTCT
IFNAR1	CTCCGCGTACAAGCATCTGA
IFNAR1	TAGATGACAACTTTATCCTG
IFNAR1	CGCCACGGCGACGAGCACTA

IFNAR2	TGAAAGTGATAGCGATACTG
IFNAR2	TGAGTGGAGAAGCACACACG
IFNAR2	CGTCATTGAAGAACAGTCAG
IFNAR2	ATATCCATGGCTTCCAACGG
IFNAR2	TTTGTGACCTCACAGATGAG
IFNG	CCAGAGCATCCAAAAGAGTG
IFNG	TGCAGGTCATTCAGATGTAG
IFNG	TTCTCTTGGCTGTTACTGCC
IFNG	TGAAGTAAAAGGAGACAATT
IFNG	CATTCAGATGTAGCGGATAA
IFNGR1	GCCGCGAACGACGGTACCTG
IFNGR1	TGTGAGAATGAACGGAAGTG
IFNGR1	TTACCTCTACGGTAAAAACA
IFNGR1	TTTCTGATATCCAGTTTAGG
IFNGR1	GCTCACACCCTGCATGACAA
IFNGR2	GGCCGACATCATGTCCATAG
IFNGR2	TGGCCCTGAGCAATAGCACG
IFNGR2	TTCCGATAGTGTTGAAACCA
IFNGR2	TTGTAAACAGTACACTCTGG
IFNGR2	TCCCTCAGCAGGCTTCCCAA
IGF1	CAGGTAGAAGAGATGCGAGG
IGF1	GATGCTCTTCAGTTCGTGTG
IGF1	GGCGCCTCAGACAGGCATCG
IGF1	CAGCACTTAAATAATTGGGT
IGF1	GTATGGCTCCAGCAGTCGGA
IGF1R	GGAGAACGACCATATCCGTG
IGF1R	TTCCGAAATTTACCGCATGG
IGF1R	GGTACAATGTGAAAGGCCGA
IGF1R	TGTGGGGAATAAGCCCCCAA
IGF1R	GGTCATCTCGAAGATGACCA
IGFBP3	GGCATCTACACCGAGCGCTG
IGFBP3	AGGAAATGCTAGTGAGTCGG
IGFBP3	GGAACTTGGGATCAGACACC
IGFBP3	GCGCACCAGCTCCGCGCACA
IGFBP3	CGCTCGGTGTAGATGCCGCA
IKBKB	GCTGGTTCATATCTTGAACA
IKBKB	GCCATGGAGTACTGCCAAGG
IKBKB	TTTGCAGGCATTCAAAAGTG
IKBKB	TGAGGGCCACACATTGGACA
IKBKB	ATGAGACTCAGATCTCCCCA
IKBKE	AGCATCCCGACATGTATGAG
IKBKE	TCAACACTACCAGCTACCTG
IKBKE	CGTGCACAAGCAGACCAGTG

IKBKE	TGCATCGCGACATCAAGCCG
IKBKE	CCGCAGGTACCGGATCACCA
IL11RA	CTGGTACTGACTCTACCCGC
IL11RA	GTACCGGATTAATGTGACTG
IL11RA	TCCTAGGAGCTGATAGCCAG
IL11RA	GCAAGAGAAGTTCTCATAGT
IL11RA	CTAGGAGGAGTCCATCCACA
IL12B	GGCCAGTACACCTGTCACAA
IL12B	TCAGTTCCCATATGGCCACG
IL12B	TGAGGACCACCATTTCTCCA
IL12B	CTCCGCACGTCACCCCTTGG
IL12B	GGGAGATGCCAGAAAAACCA
IL1B	CTTCGACACATGGGATAACG
IL1B	GGTGGTCGGAGATTCGTAGC
IL1B	CATGGCCACAACAACTGACG
IL1B	CTGAAAGCTCTCCACCTCCA
IL1B	GCTGGATGCCGCCATCCAGA
IL1R1	CAAGCAATATCCTATTACCC
IL1R1	TTTGTGTTGATGAATCCTGG
IL1R1	AATAGTCTTCCCCTAGCACT
IL1R1	ATTACAGATCAATTGTATCT
IL1R1	CTTAACCCAAATGAACACAA
IL1R2	TTACCTAGTAGTGCAGACGT
IL1R2	AGGGCATACTAATACCCCAG
IL1R2	GCCATGTCAGGTTGATGCGG
IL1R2	AGCTGATATGGTCTTGAGGG
IL1R2	ACAACCAGTAGGGCACCTGG
IL2	AAACTTAAATGTGAGCATCC
IL2	AGAAGAAGAACTCAAACCTC
IL2	ACAACTGGAGCATTTACTGC
IL2	TTCTTTGTAGAACTTGAAGT
IL2	ACAAGAATCCCAAACTCACC
IL2RA	GGATACAGGGCTCTACACAG
IL2RA	TGGCTTTGAATGTGGCGTGT
IL2RA	TTGTTTCGTTGTGTTCCGAG
IL2RA	CTGCAGGGAACCTCCACCAT
IL2RA	GGTTTCCGCAGAATAAAAAG
IL2RB	GCTGGGAAAAGAACTTCGAG
IL2RB	CCACAGATGCAACATAAGCT
IL2RB	TTGGGAAGGACACCATTCCG
IL2RB	CAGGGTGACGATGTCAACTG
IL2RB	CGGAATGGTGTCCTTCCCAA
IL2RG	CATACCAATAATGCAGAGTG

IL2RG	CTGCCCATCCACACTAGGCA
IL2RG	GGTGCAGTACCGGACTGACT
IL2RG	CATATCTCCAGTGATCCCCT
IL2RG	GGGCAGCTGCAGGAATAAGA
IL5	TGTGGGGATGGCATACACGT
IL5	ATGAGTAGAAAGCAGTGCCA
IL5	TGGAGAGTCAAACTGTGCAA
IL5	AGAAATTCCCACAAGTGCAT
IL5	CCTTTCCTCTCCAGACTCTG
IL6	CAAATTCGGTACATCCTCGA
IL6	TGCCTGGTGAAAATCATCAC
IL6	TTTGTCAATTCGTTCTGAAG
IL6	TACTCTCAAATCTGTTCTGG
IL6	ATTCGTTCTGAAGAGGTGAG
ILK	ACATTGTAGAGGGATCCATA
ILK	GATCAATGTAATGAACCGTG
ILK	CGGAGAACGACCTCAACCAG
ILK	TCAATGCAGTGAATGAACAC
ILK	CCCCTGAGAGAGCTTCTCCG
IMPA1	TCTTAACCGACAACCCCACA
IMPA1	GTTGTGTACAGTTGTGTGGA
IMPA1	CTACAAGTTTCACAACAAGA
IMPA1	CCAATTGAAACAGCTACAAA
IMPA1	GTAACTCTAGCAAGACAAGC
IMPDH1	GACGCCAAGGATTGAACTGG
IMPDH1	TCATCGCAATCATTGACGAT
IMPDH1	TCTCACAGGACTCGTCCCAA
IMPDH1	CTTCTACATCAGCTGATGGG
IMPDH1	GCTGGCGAAGAGCTGCTGCG
IMPDH2	GAGAAAATCAATGTCCCTGG
IMPDH2	ATTCAGGTGTACAGTTGTGG
IMPDH2	GGCAGCCATTGGCACTCATG
IMPDH2	GACGGCCTCACCTACAAGTG
IMPDH2	AGGAGATGATGCCCACCAAG
INHA	TGCCCCGAAGACATGCCCTG
INHA	GAGGACAAGTCAGCTGCCAG
INHA	CGGACCAGACCACCCAGTGG
INHA	CAGCAGCACCAGGACGGGGT
INHA	GCTGGGCTGAAGTCACCTGG
INSL3	GGCCGACAGTAATCTCACGC
INSL3	CTAGCGCGCGTACGAAGTGG
INSL3	CAACTTCTCACGCATCTCTG
INSL3	GCCAGGAGGCCTGCGACCGG

INSL3	GCTGGTCCACCGAAGCCAGG
INSR	TGTTGTGAATGACGTACTGG
INSR	TTATCGGCGATATGGTGATG
INSR	GGATGAACGCCGGACCTATG
INSR	AGTGAGTATGAGGATTCGGC
INSR	ACAAATGCAAGAACTCGCGG
INSRR	GGCCATCACGCTAACCACTG
INSRR	GATGCCACATCTGCGTGACG
INSRR	CCATCAACAAGAGCCCCCAA
INSRR	GGTGCGCGCAAAGACGAAGG
INSRR	ATATTCTGCCACAAGTGCGA
IRAK1	ATTTATCCCACAGAAAGACC
IRAK1	GATCAACCGCAACGCCCGTG
IRAK1	AGGAGTACATCAAGACGGGA
IRAK1	ACACGGTGTATGCTGTGAAG
IRAK1	CTTTGGGTGCGTGTACCGGG
ITGA10	CTAGCCGACCATCCACACTT
ITGA10	GCTCCTCGATTTAGACATCG
ITGA10	AGGTCTGAACTTCAGACCAG
ITGA10	CCAGCGACCAGGAGTACGGG
ITGA10	TTTCTCTTCTCAGCACAGAA
ITGA11	ATGAGAGGCGGTATACACCG
ITGA11	TGGGCTACTACAACCGCAGG
ITGA11	GGAGCTCCTACTACACCACA
ITGA11	CTTTGGGAGTGAAATCACCT
ITGA11	GCACCCATGTACTTCAACGA
ITGA2	GCAACCATGACAATATACTG
ITGA2	TGGGAATCAGTATTACACAA
ITGA2	AAAGGCACCAATAGACACAT
ITGA2	GACATCAGTCTGGAAAACCC
ITGA2	GTAATGGTAGTTGTAACTGA
ITGA2B	GGCTGTCACTGACGTCAACG
ITGA2B	GGCCGTGGGCGAGTTCGACG
ITGA2B	ATGTCTATAGACCTGATCGT
ITGA2B	TCCACAAGGACAGCCATGGG
ITGA2B	GCTGGGGCCCAGGGTCCGCG
ITGA4	AGTTCCAATACCTACCACGA
ITGA4	CATATTTGTCACTTCCAACG
ITGA4	CTCACCATCGGTTCGCCCCG
ITGA4	ATCATCTCCAGCATTAAACA
ITGA4	GGTCGTGCTGCACAGCCACG
ITGA5	TGGATCGGACCCCTGACGGG
ITGA5	AATTCGGGTGAAGTTATCTG

ITGA5	CACTAGCGGACACGATGGGG
ITGA5	CCCCGAGTACCTGATCAACC
ITGA5	ATCCCTCTACAACTTCTCAG
ITGA8	ATAGACAAGAATGATTACCC
ITGA8	GTTAACATCTGATACGACAA
ITGA8	CGGGTCGGGCGACTTACGTG
ITGA8	TCGAGTTCAAATCCAATCAG
ITGA8	GCAAGGAGAGAACTCGGCAT
ITGA9	GTATAAGAAGAAGTACGGAG
ITGA9	CCTGAGAAGAACCACGCTGT
ITGA9	GTCCTGCGGAAAGACCTGCC
ITGA9	GGAAACATTGAAGGACACGT
ITGA9	TCTATCATGGTGATGCCGGT
ITGAL	GGAGCTGTGTGGCGTCGACG
ITGAL	CCAGGTCACGGTGTAACCTG
ITGAL	TCTGGCGGAAGAGGTAACAC
ITGAL	GCCACCGGACCAGAAGACGG
ITGAL	CATCAGTGCTGACCTCAGCA
ITGAM	TGGAACACGTGATCACGAGG
ITGAM	AGTTCAGGCGCAGCACAATG
ITGAM	TCATCCGCCGAAAGTCATGT
ITGAM	ATTTGAGTGTAATGATCAGG
ITGAM	GCAGCCCCAGAAGTTCCCAG
ITGAV	AGAACATGACTATTTCAAGG
ITGAV	AGGCAATAGAGATTATGCCA
ITGAV	GCACCTCTCTTCATGGATCG
ITGAV	AACGATGAGCTCAGCTTCGT
ITGAV	GGTTTACATAGATTTCCAGG
ITGAX	GCTGACAGACGTGGTCATCG
ITGAX	AGGTAGGGCTCATATTTGGG
ITGAX	GCTGCAAGGGTTTACATACA
ITGAX	ACAGCTGCCAACCAAACGGG
ITGAX	TCTAGTCATGACCAGGACCA
ITGB1	TTGGCTGGAGGAATGTTACA
ITGB1	TAGGCCTCTGGGCTTTACGG
ITGB1	GAACGGGGTGAATGGAACAG
ITGB1	AATGTAACCAACCGTAGCAA
ITGB1	GATGACATAGAAAATCCCAG
ITGB2	TCAGATAGTACAGGTCGATG
ITGB2	CTCCAACCAGTTTCAGACCG
ITGB2	TCAGGGTGCGTGTTCACGAA
ITGB2	TCATCCCCAAGTCAGCCGTG
ITGB2	GGTCTTCCTGGGTTTCAGCG

ITGB3	GGTGAGCTTTCGCATCTGGG
ITGB3	TCACTCAAGTCAGTCCCCAG
ITGB3	GGGACTCAAGATTGGAGACA
ITGB3	CATGGACAGAACCCCAACTG
ITGB3	ATAGAGACAACTCTTCAGGG
ITGB4	CTGCGAGATCAACTACTCGG
ITGB4	CTACTCCTATAGCTACTACG
ITGB4	TTCAACGGAGACTTCGTGTG
ITGB4	CCCAGATTGACACCACCCTG
ITGB4	TGAGGATGTACAGGTCCACG
ITGB6	TGAGCACACCAGGCACACTG
ITGB6	GCTAATATTGACACACCCGA
ITGB6	ACACACCAAGACAGTTGACA
ITGB6	CCAGACTGAGGACTACCCGG
ITGB6	TGAGGTAATACAAATCCACC
ITGB7	CCACGTCCGAATCAACCAGA
ITGB7	CCGGGTATCCCTCAGCACGA
ITGB7	GGGACGCACAAGCCTTCGAG
ITGB7	AGCACTCACAGAGCCGACCT
ITGB7	GAGGAGCTGGAGGAGCCCCG
ITGB8	AGCACATGGATGTATCCATG
ITGB8	CTATGTCAAATCGACAACCA
ITGB8	TTACCGCCATCTGTCCAGAT
ITGB8	GTATTATAACATGCACAGAT
ITGB8	TTGCAGGCAGACAAATGCAG
ITK	ATACTTTGAAGATCGTCATG
ITK	AACTATCACCAACATAATGG
ITK	ATCCTCAGGAACTCGCACTG
ITK	GGAAGGGGCTATGTCAGAAG
ITK	TGATGAGCTGTTCTTCCAGG
ITLN1	AGATAACACCATTCTCAGTG
ITLN1	GGAGGAAGCCAGTGTCCGTG
ITLN1	GGAAAGTGTTGGACTGACAA
ITLN1	CAGCAAAGCAGTCTACCCAG
ITLN1	TGGTCGCTATGAGAAACAGC
ITPR1	GAGGCGGGCATATTTCACGG
ITPR1	CAAGCCCCATTCACAGACTG
ITPR1	ACTGCATAGAGACTCACGGT
ITPR1	CAAAGACGACATATTAAAGG
ITPR1	CCTGGAAAAACACATTACCG
IVD	GTCCACGGGCAAAAGCGAGT
IVD	CTGTGATGCCCAATACGCCC
IVD	CTCCTGAAGGAACTTAGCCA

IVD	GAAAGAGAAGTATCTCCCGA
IVD	TGTCTCTATGAAGCTCAAAG
JAG1	ATGGGCCCCGAATGTAACAG
JAG1	AAGTGCAAGAGTCAGTCGGG
JAG1	TCATCAGCCGTGTCTCAACG
JAG1	GATGAGGCCACGTGCAACAA
JAG1	CTATTTCTGCAAGTGCCCCG
JAK1	CCGGAAGTAGCCATCTACCA
JAK1	GCCTAGACAGCACCGTAATG
JAK1	TGGTTTCATTCGAATGACGG
JAK1	CACACTTACTCTCCACGTCG
JAK1	CATCCGGTAGTGGAGCCGGA
JAK2	CTGCCACTGCAATACCAACG
JAK2	AATGAAGAGTACAACCTCAG
JAK2	AGAAAACGATCAAACCCCAC
JAK2	TCTTCAGGAGAGAATACCAT
JAK2	ATCTGCCTCAGATTTCCCAA
JAK3	TGACGCGGAGGCGTATTCGG
JAK3	ACTCTCCAGGCTTAACACAG
JAK3	GTGTACAAATTCCTGCACCA
JAK3	AGCTCTCGAAGACTGCTGTG
JAK3	TGCCGCCGTCACCAGCCACG
JUN	GGCGGCGCAGCCGGTCAACG
JUN	GCTCTCGGACGGGAGGAACG
JUN	TGAACCTGGCCGACCCAGTG
JUN	GCCCCACGTCGGGCGAGGTG
JUN	TGAGCAGGAGGGCTTCGCCG
JUNB	GGGTAAAAGTACTGTCCCGG
JUNB	CCGGAGTCTCAAAGCGCCTG
JUNB	CTGAGGTTGGTGTAAACGGG
JUNB	CACAGCTACGGGATACGGCC
JUNB	CTGATTGTCCCCAACAGCAA
JUND	TTACACAAGCAGAACCAGCT
JUND	TAGAGGAACTGTGAGCTCGT
JUND	GCGAACCTGAGCAGCTACGC
JUND	CGAGGAGCAGGAGTTCGCCG
JUND	GTTCCTCTACCCCAAGGTGG
KALRN	GCAGTTCCAACTGGCCATCG
KALRN	AAGCAGAAGAAAGTTCGCGA
KALRN	CTTTGAGCAGTACACCATCG
KALRN	GCAGAATACGTACACCAATG
KALRN	CTAGAAGGAAGCTCATACCG
KARS	GGCCATGTAGAACTCACAGG

KARS	AGGACCCACACCATTATCAG
KARS	ATTAATAACAAACTGCGTCG
KARS	TGTCTACATGGAACTTGTGT
KARS	GCTGCCATCCACTTTCACCT
KCNA1	TCCTCGGAGAACATGTCCAG
KCNA1	TCTGGAGGCCCTTAGAGTGG
KCNA1	GTTGGAATTGTAGATGACCG
KCNA1	TCGAGTACCCCGAGAGCTCG
KCNA1	GGCCATGGAGAAGTTCCGGG
KCNA10	TATAGGATTCCATCAAAACT
KCNA10	GCTGGTTGAGTCGAAGTCTG
KCNA10	TCTCATCACAGAGCTAGTCC
KCNA10	TGGAGTCTACCTGCATCGTG
KCNA10	GGCCATGGACCAGTTCCGGG
KCNA2	GATGAGAATGAAGACATGCA
KCNA2	AGATGAAGGCTACATCAAGG
KCNA2	AAATGGCATCAAAGCTAGGG
KCNA2	TATAGCTATTGTGTCTGTCA
KCNA2	GTTTCCAGAGACCCTCTTAG
KCNA3	TGGGCACGTTGACCGGCCGG
KCNA3	GCTGGTGAACCACGGCTACG
KCNA3	GAGAAGTTCCGCGAGGACGA
KCNA3	CATGAACCTGATCGACATTG
KCNA3	GGCCATGGAGAAGTTCCGCG
KCNA4	AGCTCTCTGGATATTCAAAG
KCNA4	CCACCACCACCAGTCACGCG
KCNA4	GGACGACAGGGATCTCGTCA
KCNA4	GGCATCAGGTCAGAGCAATG
KCNA4	GCTGCTGTCGAAGGTAGCGG
KCNA5	GGTGAACCAGATGACGCACG
KCNA5	AGATACGCTTCTACCAGCTG
KCNA5	CGGAGAGCTCTGGGTCCGCG
KCNA5	GCAGCCCGGAGATGTTGATG
KCNA5	GGCCATGGAGCGCTTCCGCG
KCNA6	GCCCCCAAGAGTACTGAGTG
KCNA6	CGACCATCTACACGGAACTG
KCNA6	TCCTCCAGGAAAATGTCCAG
KCNA6	GGGGCTGCTGTAGTAGCGAG
KCNA6	TTCCACCTCGACCATCTACA
KCNA7	TCTACTACTACCAGTCCGGT
KCNA7	GGATAGCCACAAAATCGATG
KCNA7	CATCCTGAGAGTCATCCGAT
KCNA7	TGGAGCCATTCAGCGGAGCG

KCNA7	GGACTGGTAGTAGTAGAGCA
KCNC1	GAGATCGAGAACGTTCGCAA
KCNC1	GGATGTGCGCGAAGACGCCG
KCNC1	CGTACTCGTCCCGCTACGCG
KCNC1	GGCAGAAGATGACACGCATG
KCNC1	GCAAGTGCGCTACTACCGGG
KCNC3	CACGCACACCCCCTCCACGT
KCNC3	TCGAGGACCCCTACTCGTCG
KCNC3	CCTGCCCTTCTATCTCGAGG
KCNC3	CCGGTCAAAGAAGAACTCGT
KCNC3	TGTTCTCCGGAGGTGCCCCG
KCNC4	TGGTGATGTTCCCTACGCGG
KCNC4	GTGTGAGCTTGAAGATACGC
KCNC4	ATCTTCGAGAGCCCGGACGG
KCNC4	GCCAGGCCCTCCGACCCTCG
KCNC4	GCGGCCCGCACACGTCCGCG
KCND2	TGGTGTTCTACTATGTCACG
KCND2	CGCGGATACCGACACCGCTG
KCND2	TCGTCACCATGACAACACTA
KCND2	CGTTACCCAGACACTCTACT
KCND2	CGTGACATAGTAGAACACCA
KCNE1	AGCTTGCCGTCACTGCTGCG
KCNE1	CAGTACCATGAGGACGTAGA
KCNE1	ACTCGATGTAGACGTTGAAT
KCNE1	CAGAAAGGGCGTCACCGCTG
KCNE1	GATCCTGTCTAACACCACAG
KCNG1	CGTCGGATCATCATCAACGT
KCNG1	GAAGGTCAGGATAGTGCCGA
KCNG1	CAGCGAGGGCCGCGACAGCG
KCNG1	ATCGTGGAGTCGGTGTGCGT
KCNG1	GCTGACGGAGAGGTTGACGG
KCNH1	GCTGGTTGTTGATAGCATCG
KCNH1	CTGGCTGTACCAACTAGCGA
KCNH1	CAAGCTGTCTGGCTATCACA
KCNH1	ATCATCTTGGATCACCTCCA
KCNH1	GGTCATTGTGAAATACAACG
KCNH2	GCATCGACATGAACGCGGTG
KCNH2	CCGATGCGTGAGTCCATGTG
KCNH2	CTCGTCGGCCGACGACATCG
KCNH2	GTTCATCCTCAATTTCGAGG
KCNH2	TCGCTACTCAGAGTACGGCG
KCNH3	ATCAGCGAAACCAAGAACCG
KCNH3	GTACGTCAGCCCCTTCACGT

KCNH3	CCCAGCCCTGATGCACGCGG
KCNH3	CCCCGCATCTCAGTACTTCG
KCNH3	GTACTCGCAGTACAGCGCCG
KCNH4	CACAGGCTACGGTCGCACCG
KCNH4	TACCGTGATTACTGTCCCCG
KCNH4	TAGTGGAGGCCAATGGAACG
KCNH4	GGTGCCCTATGTCAATGGCT
KCNH4	TTACATCTTCAACATCACCG
KCNH5	GTATAGCATCGGAGACTACG
KCNH5	CACGCCAATGAATAAAACAG
KCNH5	GTGACCTACACATCATCAAG
KCNH5	TGGATTATATTGTCTCAACA
KCNH5	TTGCATATAAAACCAAACAG
KCNH6	ACTCTTCGGCTACTCCCGAG
KCNH6	GATCAGGACGAATCACGGCG
KCNH6	GGGGACACGCTGGTGCACCT
KCNH6	CGCGATGATCTCAATCTCCG
KCNH6	GCACCACATCTACCAGGCAG
KCNH7	GACATCCCCACAGTGAACGA
KCNH7	ATTATCCAAAGACTATACTC
KCNH7	TCCATGGACCCGAGACCAAG
KCNH7	ATCGTAAACTTGTTGATGCG
KCNH7	AATTCCAGTGAAAAACCAAG
KCNH8	TCAAATAGAAAAGTCACTGG
KCNH8	CAAGCGACTCCCATCCATTG
KCNH8	GTATGCTTTCAACGTCACAG
KCNH8	ACTATGGCAACAATACCTTG
KCNH8	GCTCAGCGTGAAGTACAGAG
KCNJ1	TGTACGCTACTGCATACCAC
KCNJ1	GCTTTGCCTCCTAATCCGAG
KCNJ1	TGCCAATCACACTCCCTGTG
KCNJ1	ACACCTGAATCCATATCCAA
KCNJ1	CAGAAGGATGGAATTCCGGG
KCNJ10	TGGGCCCAGGGATACGACGG
KCNJ10	GCTTCAGACCCACCAAACCA
KCNJ10	AGGTTGTCCACAGGTCCTTG
KCNJ10	GCAACTCGGATCATGAGGCA
KCNJ10	GGTCTGACTGTAATACACCT
KCNJ11	CCTCATCTTCAGCAAGCATG
KCNJ11	TCTTGCGTACCACCTGCATG
KCNJ11	CAAGAAAGGCAACTGCAACG
KCNJ11	CCACGATGTTCTGCACGATG
KCNJ11	TCATCGTGCAGAACATCGTG

KCNJ12	CATCTTGCATGAGATTGACG
KCNJ12	TGTGACGGAGGAGTGCCCGG
KCNJ12	GGTTACCCACACGCCACATG
KCNJ12	CCTGTGTGGACATCCGCTGG
KCNJ12	GCTGTTCAGCCACAACGCCG
KCNJ13	CAAATGGATGGCGCTCAAAG
KCNJ13	TATGGTACCATGTTCCCCAG
KCNJ13	TGATAAAAGCCTCTAGCATG
KCNJ13	GCGCTGGCGTTGGATGATGT
KCNJ13	TCCTAAGTCAAAGATACCGG
KCNJ14	CGGCGTGCGCAGCGTCACCG
KCNJ14	AACGTGCGTTTCGTAAACCT
KCNJ14	CTTCAGCGAGAACGCCGTCG
KCNJ14	CATGACAGCACCCACGACGA
KCNJ14	GCACCGGTGCAGTCACCCGT
KCNJ15	CCACTTCATGTCGATAACTG
KCNJ15	CCACTCTTGGACATGACGCG
KCNJ15	GTCCCCATGAATAAACGCGA
KCNJ15	AGCCACTGTCAAATTCCACG
KCNJ15	GCTTGGTGATGACTGCACAG
KCNJ16	AATGCGGACGCAAAATACCC
KCNJ16	GAATGGACGTTGTCAACACA
KCNJ16	TGCAACTTAAGATGGACTGG
KCNJ16	AATCACCAATGCGCCACATG
KCNJ16	CCACTCTTGTGGACACCAAG
KCNJ2	CCAGCGAATGTCCACACACG
KCNJ2	GAACATGAGCTTCCACCAAG
KCNJ2	GTGGTGTTCCAGTCAATCGT
KCNJ2	GGGCAAAGCTTGTGTGTCCG
KCNJ2	TGATGAACTGAACATTACAG
KCNJ3	AGGCGTGTAGTTACCGACGT
KCNJ3	ATCACGTGGCAAATTGTGAG
KCNJ3	ACTCACGCTTATGTTCCGGG
KCNJ3	CATCACAGACAAGTGCCCCG
KCNJ3	GTAGTGACCACATCGTCCAG
KCNJ4	CATTGTCCACGAGATCGACG
KCNJ4	CGCGCACCGAAATGACCGCG
KCNJ4	CAAGTCGCAGCGCTACATGG
KCNJ4	GACCAGCGGGACCTCAACGT
KCNJ4	CATGCTCATGATCTTCTCCG
KCNJ5	AACCAGACAGACATCAACGT
KCNJ5	ACAACGCAGTCATCTCCATG
KCNJ5	CCAGGTGACAGTGTAAACCA

KCNJ5	GGCAAGTGCAACGTGCACCA
KCNJ5	AATGAAGCCGAAGAACAGCC
KCNJ6	TGGATACAGACCAACTCAG
KCNJ6	AACGTAGGGTATTACACGG
KCNJ6	AAGTTGCCTAAGCAGGCCA
KCNJ6	AGGAATTCCCACATTGTGG
KCNJ6	TGTTCATCACGGCAACGTGA
KCNJ8	GGCTGCTCTTCGCTATCATG
KCNJ8	CTGTGCTTCATGTTCCGAGT
KCNJ8	CTGTGTGTGTGACTAATGTC
KCNJ8	CTGGAGAATCAAAACCGTGA
KCNJ8	GTAAGCATAGATGTCCCCAT
KCNJ9	CGAGACATACCGCTACCTGA
KCNJ9	AGGAGCGCAAGTCGCCCACG
KCNJ9	GTTGACGCACGGCGTCCACG
KCNJ9	GTGCGAGGAGAAGACGAGCG
KCNJ9	CCAGGTGCTCCAGGTCGCCG
KCNK1	ACCGTGCCCTTGTCAGATGG
KCNK1	CAAACCCAAGGAGCACGGCA
KCNK1	GGCCAGCAACTACGGCGTGT
KCNK1	GGAAGTAGAGGACCGGCCTG
KCNK1	GGTGCTGGCGAAGAAGAGCG
KCNK10	AGTGGTATAAGCCCCTAGTG
KCNK10	GTCATCTTTAAGTACATCGA
KCNK10	ATTGCTCCGAGCACTGAAGG
KCNK10	TCACACAGACATGATCCCGC
KCNK10	GGAGAAGGCGGAATTCCTGC
KCNK13	CAGCATGACGTAGTACACGG
KCNK13	AGTAGCTCCAGCCTTCAATG
KCNK13	ATGACAACTCCGGCGACAGT
KCNK13	GTGGCACGACTTCATGATGT
KCNK13	CTGAGCCGCGACGAGCTGCG
KCNK2	CAGCTCCGTCGAATTGACAC
KCNK2	ATCTCACCACGCACAGAAGG
KCNK2	GACAACCACCAGGAATATCG
KCNK2	ATGTGTTTGAATATGATCGC
KCNK2	GGTCTCCACGATATTCCTGG
KCNK3	AGAAGGTCCAGTGCTCGTAG
KCNK3	GTGCAGCAGGTACCTCACCA
KCNK3	CGCTACAACCTCAGCCAGGG
KCNK3	CGGCGAGCCGTTACCGATGG
KCNK3	CTACCTGCTGGTGGGCGCCG
KCNK4	ATATAGCAGAACACGAACGT

KCNK4	ATAGTGACGCTTACCACCGT
KCNK4	GCCAGTAGGATCCCAAACAG
KCNK4	GACAGCACTCTTACTAGCTC
KCNK4	GGTCCGAGAGAAGTTCCTGA
KCNK6	GCCGGCACCAGAAAGCAGA
KCNK6	CCAGCAGGCCGCCCGCCGG
KCNK6	GCATGCTCAGCCAAGACAG
KCNK6	AGCACGCTGATCACCACCG
KCNK6	CGCATGCTCAGCCAAGACAG
KCNK9	CACGGCGTAGAACATGCAGA
KCNK9	CACGTAGTCCCCGAACCCAA
KCNK9	GCAGAAGAAGCCGCTCTACG
KCNK9	AAGCGCATTAAGAAGTGCTG
KCNK9	GGAGGAGAAACTCAAAGCCG
KCNMA1	ATACGGGGGCTCCTATAGTG
KCNMA1	TTTCACATTACAGATCGACA
KCNMA1	GAGGCCCAGAAGATTAACAA
KCNMA1	TCTCCATATTTATCAGCACG
KCNMA1	CTAGGCTGAGATGGTTCGCG
KCNN4	GAGAGCGCCGATGCTGCGGT
KCNN4	CTTTACATGAACACGCACCC
KCNN4	CCTAAATCCTGCACGCACGG
KCNN4	CGATGGTCAGGAATGTGATG
KCNN4	GCTTGGCCACGAACCAGTGG
KCNQ1	GGTGAACGAGTCAGGCCGCG
KCNQ1	ATGCTACACGTCGACCGCCA
KCNQ1	GCGATACTCACACGGCGAAG
KCNQ1	GCACTCCACAGACCTCATCG
KCNQ1	GTACCTGGCTGAGAAGGACG
KCNQ2	GTGGCAGTACTACGAGCGAA
KCNQ2	ATGATCCGCATGGACCGGCG
KCNQ2	GCTGACCACCATTGGCTACG
KCNQ2	TGGAAAACACAGACAGCACG
KCNQ2	AACGTGCTGGAGCGGCCGCG
KCNQ3	TATGGCATCTACATTCAGAG
KCNQ3	CGGGTACTCACACCAACGCG
KCNQ3	GACTTACCCAACATGCACAG
KCNQ3	GGTTGAGAAAGACGTCCCAG
KCNQ3	CCTTTAGTATTGCTACCACG
KCNQ4	CAAGATGAGGAGACACTCGT
KCNQ4	GATACTGTCATAGTAGTACC
KCNQ4	GTGACAAGACACCGCACACA
KCNQ4	GATGTTGCCCTGGGTACCCG

KCNQ4	TGAGCGCCACTAGCTCCGCG
KCNQ5	ACAGGTAGTTCTGCACCCGC
KCNQ5	ACAGACGATCTCAGTATGCT
KCNQ5	ATGCCAGTGTGATGTATCAG
KCNQ5	GGAGGCACTTGGAAATTACT
KCNQ5	GCTACTGCTGGGCACCCGCG
KCNT2	AATGGATGTACAGGTTCGAA
KCNT2	ATAACCTGTAAGCCCCACAA
KCNT2	CATAGCATAATTGCCAGCAT
KCNT2	TATACAGTTTAAAGCTAACA
KCNT2	GGACTTACTGAGATAATGAA
KCNV1	GCTTAACTCAGCTGACATCA
KCNV1	CTGCAGGAGATCCAGTACTG
KCNV1	CCAGAGCTTCTGGCGAACAG
KCNV1	TTCTGGTAGAGAGCCTAAGT
KCNV1	CATCGTCGCAAAGCTCCAGA
KDM1A	TGGAATAGCAGAGACTCCGG
KDM1A	CTAAATAACTGTGAACTCGG
KDM1A	TTTCTGAAACAGGATCGTGT
KDM1A	TGAGAAGTCATCCGGTCATG
KDM1A	GGAATAGCAGAGACTCCGGA
KDM3A	TGCATTTGAAACATCCGATG
KDM3A	CATTCTGTAAGAGCGAAATG
KDM3A	TGGCTGGCCGACCTAACCAG
KDM3A	TTTCTGAACGAATTGTACAG
KDM3A	AGAACATGCACCTTCCCCAT
KDM4A	TGTGCACAGTTATGCCAAAG
KDM4A	ATCATACAGACAGCGCAGTG
KDM4A	GTGGTATTTCAAGACAAACT
KDM4A	ATATTTCTTCAGCATTAACG
KDM4A	GGACATGGTGAAGATCTCCA
KDM4B	ATGTCATCATACGTCTGCCG
KDM4B	ACCCGTGATTGAAGCCGGCG
KDM4B	TGAGTGACCCGGACGCCTTG
KDM4B	GGCTGGGGCAGCGCTCCTAG
KDM4B	TCACCAGGTACTGTACCCCG
KDM4C	GGTCATCTGTGACTGAGTCG
KDM4C	GCAAGAGTATAATGCAACAG
KDM4C	TTCTCATAGCCACTAGACAA
KDM4C	AGCTATTTCTCTCTCCACTG
KDM4C	TACCTTGCAGATAACCCAGG
KDM4D	AAATCGGTGAATTATAGATG
KDM4D	AACCTGAACGCTATGACCTG

KDM4D	ATAATCAATCCATCGCGGAG
KDM4D	CTTCAATCGCATAACTCAGG
KDM4D	GCAGGTCCTGAATTGTTCCC
KDM6A	CCAACTATCTAACTCCACTC
KDM6A	CTGGTAAGTCTCACCTTCCG
KDM6A	TCTTTGTATGAACAGCTGGG
KDM6A	CAATTGTCAGAAGTATTCTG
KDM6A	AATTCGTGCTGCAAGTGCAG
KDM6B	GACAAAAGTACTGTTATCGG
KDM6B	GCAGTCGGAAACCGTTCTTG
KDM6B	GCTGGACGAATCCATTCGCA
KDM6B	GGTGCTAGAAGAGATCAGCC
KDM6B	CATTTCAGCTAACCAAGCCA
KDR	TAATGTACACGACTCCATGT
KDR	CCAATCACACAATTAAAGCG
KDR	CAGCCTCTGCCAATCCATGT
KDR	CAAGAACTGAACTAAATGTG
KDR	ATACCAGTGGATGTGATGCG
KEAP1	CAGCACCGTTCATGACGTGG
KEAP1	AGTACGACTGCGAACAGCGA
KEAP1	ACAACCCCATGACCAATCAG
KEAP1	CCTGGAGGATCATACCAAGC
KEAP1	CTTGTGGGCCATGAACTGGG
KEL	GCAGGACAACCAGTCGATGG
KEL	TCCACGCACTTCATCCATCG
KEL	AACACACAGATGTCTCACAG
KEL	TGAAGTGATGGAGATTGACA
KEL	AGGAGATGCTGCTAAAGCAG
KIF11	TCTTGTGTAGGAGTATACGG
KIF11	GACTGAATTACCTTGTTACG
KIF11	GAAGTTAGTGTACGAACTGG
KIF11	ACCTAATGAAGAGTATACCT
KIF11	GAAGGGGAAGAACATCCAGG
KIT	TCAGACTTAATAGTCCGCGT
KIT	GAAAGAAGACAACGACACGC
KIT	GAATGGCATGCTCCAATGTG
KIT	TCTAGTGCATTCAAGCACAA
KIT	TTTGTCCAGGAACTGAGCAG
KLK10	GTGCTGACGGCCGCGCACTG
KLK10	GTCCATCCCAAGTACCACCA
KLK10	CCGGGCTGAGCACAGCGGTA
KLK10	TATGGCTCCCCGTGCGCGCG
KLK10	TTGAAGAGCGAGACCTGCCA

KLK11	TAGCCGCGTCTTCTCGAACA
KLK11	GCCGCTACATAGTTCACCTG
KLK11	GCCAGCAGTGACACAGCGTG
KLK11	GATTAACTGCAGAATCCTCA
KLK11	GCCCCAGGTGAACTATGTAG
KLK14	GCAGCCCGCACGGATCGGGA
KLK14	TATATCCAGCCCCATCGGTG
KLK14	GTCATCACTGCTGCTCACTG
KLK14	AGCCATGACACAGAGCCAAG
KLK14	GCTGTGTCTTCATGTCCCTG
KLK15	CAAGTGGCTCTCTACGAGCG
KLK15	TTTCACGCACCTTGTGACCG
KLK15	GCAGCAACATGATGTCGTTG
KLK15	CCGCAGACAGCACCCAGTGT
KLK15	CCTCACCTGTGGATGCCAGC
KLK5	TGAGCATGAGGTCGTTAGAG
KLK5	CTGGGTGTGCATATCGCAGT
KLK5	AGGGCACGGTGTTAGAGGGG
KLK5	GCCCAACCAGCTCTACTGCG
KLK5	GTTGGGCCTTAGCAACAGCG
KLK6	TGGCGGCATCATAGTCAGGG
KLK6	CCAGGTGGATGTATGCACAC
KLK6	GGAAGCATAACCTTCGGCAA
KLK6	GCTGAGCAGTCCCTCTCCAG
KLK6	TGTTCTGGGTGATCTGGCCA
KLK7	GCTTGACATCCACGCACATG
KLK7	GAGCATGAGGTCATTAACAT
KLK7	CACCTGGGCAGTGATACGCT
KLK7	TGGTTCCAGGGGGTTCGCAG
KLK7	GGGTCTGTGTGGAGTAGCCG
KLK8	TGGACAAGCCCACTACCTCG
KLK8	CAGGGCCAGCAACTACTCTG
KLK8	AGGAAATACACAGTACGCCT
KLK8	GAAGCATCAGATCATGGTTG
KLK8	GACCTCGTGCGGCCAAGACG
KLKB1	CTCAGCACCTTTATAGCGGT
KLKB1	TTTGAGATTGTGTAACACTG
KLKB1	TATGGAAAATCGAGTCACAA
KLKB1	TGCCATTCTAAAATTTACCC
KLKB1	CTATTAAAGTACAGTCCCGG
KPNB1	CACACAGTGTCCAGATACGA
KPNB1	TATCTCGAACAACTACACTG
KPNB1	AGCTCCTAGAGACTACAGAC

KPNB1	ATCTTGGCAAATATAACCGA
KPNB1	GGAACCCCTGCAAAGCAGCA
KRAS	AAGAGGAGTACAGTGCAATG
KRAS	AGATATTCACCATTATAGGT
KRAS	CTGAATTAGCTGTATCGTCA
KRAS	GATGTACCTATGGTCCTAGT
KRAS	TCCCTTCTCAGGATTCCTAC
KRT8	GAAGACGGCTCGAAGCAACA
KRT8	GCTCTGGTTGACCGTAACTG
KRT8	CCTCACCTTGTCTATGAAGG
KRT8	ACTCCCTCACCTTCTTGATG
KRT8	GCTACATCAACAACCTTAGG
KSR1	CTGACACGGAGATGGAGCGT
KSR1	GGATGCCTACCGGGTACCGT
KSR1	GTTGGAGTTCATTGGATGCG
KSR1	TGGGTTGGATGATGTCGGGA
KSR1	GCAGCAGCTGGAAGACCTTG
KSR2	CCACGTGGACAGGCTTACCG
KSR2	AATTGAAGTGGAGCCAACGT
KSR2	TTAAATAGTTACCTCCTCGG
KSR2	GAGTTGCCGAGATCTCTGCG
KSR2	GTTCATCCGTCATCTCCAAG
KYNU	GGTGGCTCTCCACCTAGATG
KYNU	CTTCCCCACTTCATGACCAT
KYNU	AAGCCAACATAACAACCCTA
KYNU	GCGGATGATAAAGCCAAGAG
KYNU	TGAACTCAAATGCCACCCAA
LAMA1	TCTCGACATTCGTTGCACTG
LAMA1	GGATGTACTCAATATCGCTG
LAMA1	CCTCTCTGGCTATTACCGCG
LAMA1	ATGTGAGCATAATACTTGCG
LAMA1	GGTCAGCATCAACAACACCG
LAMA5	CTTCCGATACGTCAACCGGG
LAMA5	GCGCATCACACGGGACGACG
LAMA5	GCCATTCACGAGCAACTGCG
LAMA5	CTGCACTCAGAGACCCAGCG
LAMA5	TCCAGATTGTGACTGCAGCG
LAMB1	ACGGAACTTAACGAACCCTG
LAMB1	GAAGTATCTATACACACCCC
LAMB1	ATGTGCAGGCATAACACCAA
LAMB1	TGACTGCGACCCAGTGACTG
LAMB1	GTTCCCTTCTCAAAGCACAC
LAMC1	TGACCTCTATCAAGATACGT

LAMC1	CTGGAATCATCTAATCCTCG
LAMC1	TGCCATTTACAAGCGCACAC
LAMC1	GCAGCTGTAACCCCGTGACG
LAMC1	GGAATACTGTGTGCAGACCG
LAP3	CTCCACCGCAGACATGACGA
LAP3	AAGTGCTAGTAGTAAAACCG
LAP3	TGTCGGCAAAGCTCTATGGA
LAP3	GACCTCATGAGGGCTGACAT
LAP3	GGCCAACTAGCACCACGCTG
LARS	TGTCTATCATGATCCATGCA
LARS	TGTTGTTAAGGAATTAATGG
LARS	TGGCTATGTGGCCGGAACAA
LARS	TGCTATAAGAGACTAACCAC
LARS	CTTCATCTGGAAAATCAGGG
LARS2	TGGGCTGGCCTATCAAAAGG
LARS2	CCATCAGCGACACCATAGCA
LARS2	CACACAGTCCCCAATCCAGT
LARS2	CCAGCCACAGACTCCTACAT
LARS2	CCTTTAATGTGAAGTCCAGG
LATS1	GTAACACTCCTTACTTGAGG
LATS1	CTTGATTAGGAGGATTCATG
LATS1	CCTTCTGCTTTACAAACAGG
LATS1	GCAGCCATCTGCTCTCGTCG
LATS1	AAGAGGAGTGAAAAGCCAGA
LATS2	ACCAGCAGAAGGTTAACCGG
LATS2	AGAGCCGCAAAAGCGCCAAG
LATS2	GTAGGACGCAAACGAATCGC
LATS2	AAGACGCCGCCGGAGACCGG
LATS2	GCTATTTCCAGAATAAGTCG
LBP	GTCGCCAGGATCACCGACAA
LBP	AGTCGGATATTAGAGTCAGG
LBP	GACTTGAGGATCCCCCACGT
LBP	TGGACATGTCGGGAGACTTG
LBP	TGATCCTGGCGACCAAGCCG
LCK	GACCCACTGGTTACCTACGA
LCK	GCCGGGAAAAGTGATTCGAG
LCK	CTACAACGGGCACACGAAGG
LCK	GCTGGTTCGGCTCTACGCTG
LCK	ATAGTCCCACTGGATGGCAA
LCMT2	GGCGAAGCAGGATTTACGCG
LCMT2	TAGTAGCCTCGGTGAATGAG
LCMT2	AAGGGCATTAGGAAAACGCT
LCMT2	TTAACCGGGCCTTTCGAGAG

LCMT2	TGAGGCTGCGTCGAGCCCCG
LDHA	CCGATTCCGTTACCTAATGG
LDHA	GGGGAACATGGAGATTCCAG
LDHA	ACAACTGTAATCTTATTCTG
LDHA	AGCCGTGATAATGACCAGCT
LDHA	GCTGGGGCACGTCAGCAAGA
LDHB	TGCCAATTCTAAGATTGTAG
LDHB	TACATCCACTTCCAATCACG
LDHB	GGGGAACATGGCGACTCAAG
LDHB	GGACTGTACTTGACGATCTG
LDHB	GTAGTGGGTGTTGGACAAGT
LDLR	CTTAAGGTCATTGCAGACGT
LDLR	CAGAGCACTGGAATTCGTCA
LDLR	GACAACGGCTCAGACGAGCA
LDLR	ATGAACAGGATCCACCACGA
LDLR	TCCAGAACTGAGGAATGCAG
LHCGR	GTGGCTGGGGTAAGTCAACG
LHCGR	GCACAATGGAGCCTTCCGTG
LHCGR	CTTGGTTTGGGAATCAACTG
LHCGR	TAGCCCATAATATCTTCACA
LHCGR	GAGCCTTCCGTGGGGCCACA
LIAS	GGCTGGCCCTACCATGATCG
LIAS	CAGGAGTAAGACACTCCACA
LIAS	AGGAGCTTAACGGTCTGACT
LIAS	ATGGAGGTAGTCTTAACCTA
LIAS	GGAAATGTCTCTACGCTGCG
LIG1	TTGTAACCAGATGGATCCAG
LIG1	CTTCACCGGAGAGTCACTCT
LIG1	GGTCCTGAAACGCTTTGAGG
LIG1	AGAATTCCCACCAGCCATGG
LIG1	ACGAGGACAGAGAAGCCAAG
LIG3	CTGTTAGGTACACATCACCG
LIG3	TGTGTGGACTATGCCAAGCG
LIG3	CTTGGCTGACATGATAACCC
LIG3	TGAAGACAGACTTCTCTTAG
LIG3	GAAAAGCCAGAAAATTTCCG
LIMK1	GTGAAGAATTCCATCCACGT
LIMK1	TCCGGCTTATACTCCCAGCG
LIMK1	CGATAAAGGTCCCACACGTG
LIMK1	GGTGTGGCCGGCAGACTACG
LIMK1	GCACTGCTACTACCAGACTG
LIMK2	ACTGTCAACGAAACCTGGCA
LIMK2	GCCCTGTGACCTAATCCATG

LIMK2	CCTCCTCCACTCGAAGTGTG
LIMK2	CTGACAGAGTACATTGAGGG
LIMK2	ATAGCTGCTGGAACAACGAA
LIPG	TGCGGTCAATAATACCAGGG
LIPG	AATACCAATGCTCAAGCCGA
LIPG	GAAGGATGCTACCTCTCCGT
LIPG	GTGCACTGGAAGGCAAAACT
LIPG	GGATGCGGTCAATAATACCA
LIPH	TTTGTTGGAGAGATGTACGA
LIPH	TTGGTACGGGACTAAATGTG
LIPH	TGCCCCAAAACAATATTGGG
LIPH	AGGAGCGTTACCATCAGTGT
LIPH	AGTCATCCATCCAAACAGGA
LMAN1	GATGTGGCAACGCGACCGCG
LMAN1	CCCCTTACACTATAGTGACG
LMAN1	AAAGCCAAAGAGGCTCAGTG
LMAN1	GATCAGCTGATCCAAACACA
LMAN1	ATATGATTATCCCTGCACAA
LMTK2	TCTGGAGCAGTCCATCGCAG
LMTK2	ACAATAACATCTAAACACGT
LMTK2	CCGACATGGATAATCCAGAA
LMTK2	AAAAGCAAGTGCCAACCCAA
LMTK2	GGTGAGAAAACAATTCCGCA
LMTK3	CGTGCTCACTTGAACCACGA
LMTK3	CGGGGAGTACACTCCCCCTG
LMTK3	CCCGTAGTCTCCGATGCGCA
LMTK3	TGACCGTGAGCACATCGTCG
LMTK3	GTAATGTCTGCGTAACCGCA
LNPEP	GGGTTTGCTCACCTTCCGAG
LNPEP	TTCATAGCGTAGTGGCACAA
LNPEP	AGGATAGTGGAATATGCCAC
LNPEP	GCCTGAGCTTCTATTCATGA
LNPEP	ACCAGCAGTCGGGAACCCCG
LPL	ATCAGGAGAAAGACGACTCG
LPL	GAGATGAATGGAGCGCTCGT
LPL	AAGAGATGGACATTGTCCAG
LPL	TCCCGGAGTAGCAGAGTCCG
LPL	TCTTACACACATTCACCAGA
LPO	GGTCACCTAGCAAAATGGGT
LPO	GCACGGGTCGCATCTCACCA
LPO	GTGGTGTTGATGAACTCACA
LPO	GAATACCTCAAGCATGCCAA
LPO	GTTATGCTCGCGGAGAAAGA

LRP1	CGATGCGCCGGATTTCATGG
LRP1	CCTGGGAGATCACCACGTAG
LRP1	GCGCTCGGGGACGCACACGT
LRP1	GCCGGCCCTTGCCATACACG
LRP1	GGGCAAAATGACACACCCCA
LRP2	GGTGACTATAGCGACGAGAG
LRP2	GATGAACTACCACCGACCGT
LRP2	TGACAAACGCAACGACTGTG
LRP2	GCATAACCCGACGAGTAGCA
LRP2	TGACTTTAAGAAGAACACGG
LRP5	TGAGGCGCGTCACCTCGATG
LRP5	GGAGGGACTTGGACAACCCG
LRP5	CGAGACCAATAACAACGACG
LRP5	CCCGGCGGACGGACCTACGG
LRP5	CCACCAGCCGTACGTCCCGG
LRRC4B	CCAGCCGGGTGATCTGCACA
LRRC4B	GCGGCTGGAATACATCTCGG
LRRC4B	GGGCGTCAGCCAGTTGACGG
LRRC4B	AGGTACTCGAAGGCCTGCGT
LRRC4B	CCAGCATCCCGGTCAACACG
LRRK1	GATCAATCAAGGAATTGACG
LRRK1	GGCCCCAGGGGATAACCGAG
LRRK1	CTGATGAAGATGATCATCGT
LRRK1	CGACGACGTGCAGTACCTGA
LRRK1	GCACTCAAAGAAGTTCCCCT
LRRK2	CCTGTTACAAAGCATTAACG
LRRK2	AGTGGTAATCTCGTATGGCA
LRRK2	CATGCCAGAAGAATCCAGGG
LRRK2	AAATCTAATTCAATTAGTGT
LRRK2	CTACTTACAAGGAATCGCTA
LSS	GGCGCTCACCATATACCACG
LSS	GCAGAGGGATGCGTGCCACG
LSS	AGTACTTCTGGTAGTCGGGA
LSS	TTCCGCGGCACTCAGCCGAA
LSS	GCTGGCGCAGAGGAACAACG
LTA	CAGGTGGATGTTTACCAATG
LTA	ACGAAGTAGATGCCACTGGT
LTA	ACTGCTCTGGAGAGCAAACA
LTA	CTCATGGGCCAGGTAGAGTG
LTA	TCAAGGAGAAACCATCCTGG
LTB	GAACAGGCGTTTCTGACGAG
LTB	AGGTAATAGAGGCCGTCCTG
LTB	CAGAAACAGATCTCAGCCCC

LTB	ACCCAACCAGGTAACGGAGA
LTB	TCTGGTGACCTTGTTGCTGG
LTF	CAGCCCAGAATCTATCCTCG
LTF	ATTCACACTTCGTGCCACAA
LTF	AGCTGCGCAAGTGTAACCAG
LTF	ACGAACTCACTATTATGCCG
LTF	GTGAAGATACCTGTGCCTGG
LTK	AGCACGTACCCGGAAAACGT
LTK	CTCACCCGGAGAATTCAGCG
LTK	CTCACAGTCACCGAGAACCA
LTK	CTGGCAATAATAGGGATTGG
LTK	CAACGGCTCGAGACTCCCCG
LYN	TGAAAGACAAGTCGTCCGGG
LYN	GCTCGTGAGGCTCTACGCTG
LYN	TTACTATAACAACAGTACCA
LYN	TAATAACATCACCATGCACA
LYN	CCAACTTGATGGACTCCCGG
MAF	GATCACGGCGGACACCACGG
MAF	TGAAGTGAAAAAGGAACCGG
MAF	GCTGCACGGCGTGCTCATGG
MAF	GAAGACTACTACTGGATGAC
MAF	GGTGTCCGCCGTGATCGCCG
MAK	TGATCGATTATATCCGGCAG
MAK	CACTGAGTCATAAAAGTGGT
MAK	CTGGCCAGAAGGATACCAGC
MAK	ATTCCAGATGATTTGACGAA
MAK	TTATGTATCAAATATTGCAA
MAOA	GAAGTGAATCTTGGCAGTCA
MAOA	GTGCATGATGTATTACAAGG
MAOA	TGGTATGTGAAGCAGTGCGG
MAOA	AGATTCCAACTGATGCACCC
MAOA	GGTGACAGAGAATATCCGAG
MAOB	GTCCAACATAGGATCCTCCA
MAOB	GCAAATCATACCCCTTCAGG
MAOB	TTTGGTATCATCCAACGTGT
MAOB	ATCATCTCGACAACAAATGG
MAOB	CATGAGCAACAAATGCGACG
MAP2K1	CATCCTAGTCAACTCCCGTG
MAP2K1	GCAGCAGCGAAAGCGCCTTG
MAP2K1	GGGCACAAGGTCCTACATGT
MAP2K1	TATGGTGCGTTCTACAGCGA
MAP2K1	GAGTTGACTAGGATGTTGGA
MAP2K2	AAGCACCAGATCATGCACCG

MAP2K2	ACGGCGAGTTGCATTCGTGC
MAP2K2	GGCCCATCCCCTACCAGCGA
MAP2K2	GGATTCCCGAGGAGATCCTG
MAP2K2	GTACATCGTGGGCTTCTACG
MAP2K3	CTACGGGGCACTATTCAGAG
MAP2K3	TTGGTGACCATCTCAGAACT
MAP2K3	CTTGGACAAGTTCTACCGGA
MAP2K3	AAGCTGTCGGTGATCCACAG
MAP2K3	CTACACTGTCACCTTCTACG
MAP2K4	TTTGTAAAACTTATCAAACG
MAP2K4	CCAAGAATACTCACATGTGT
MAP2K4	GACCTTGGAGAAATTGGACG
MAP2K4	CCAGAGAATTCGGTCAACAG
MAP2K4	TTATGGTTCTGTCAACAAAA
MAP2K6	TTGGAGTCTAAATCTCGAGG
MAP2K6	TTATGGCGCACTGTTTCGGG
MAP2K6	ACTGGGACGAGGTGCGTACG
MAP2K6	TAAAGGCCAGACAATTCCAG
MAP2K6	ACTTACGTGGAACTGGTCTG
MAP2K7	ACGGGCTACCTGACCATCGG
MAP2K7	CTTAACGGCAATGACGTGGC
MAP2K7	CATTCTGGGCAAGATGACAG
MAP2K7	CAAAGCACTGCACGATGTAG
MAP2K7	GCTCACCTCTCCATGCTGCG
MAP3K1	CAAGATGGATGATCGTCCAG
MAP3K1	CTTCTCACCATATAGCCCTG
MAP3K1	ATCTGCACATTTGACTAGGA
MAP3K1	GGAGAGCACTGGAAATTCTG
MAP3K1	GCATCACTTTGTTAACACGG
MAP3K12	TGTGGAGAGTACATCAGCTG
MAP3K12	TCTCGAAGTACACACTGGGT
MAP3K12	AACATCATCACTTTCAAGTG
MAP3K12	TTCATTGCGGATCACCTCAG
MAP3K12	CCTGCACAAGATTATCCACA
MAP3K13	GATGGATGATAGGACGAACA
MAP3K13	TGCCCATGGACAACTCTACG
MAP3K13	AGTGTTGACGAGCGTAAGTG
MAP3K13	AAATCCTTATGAAACAGACG
MAP3K13	GTGTCTGCCGAAAAGAAGGT
MAP3K5	GGGCAGCCGACGGACCACGG
MAP3K5	ACAGTCAGGAATTAATTATG
MAP3K5	ACTTATGGGATAGTCTACGC
MAP3K5	CTGACTTCGGAACATCAAAG

MAP3K5	GTAAAAGCGGTCCAGCACGG
MAP3K6	CAGTGGCCGTCACCACATAG
MAP3K6	GGGCCGCGATCGCCACACGA
MAP3K6	TCTGGATGCCTTCTACAACG
MAP3K6	GCTGCTTTCCCATACCCGCG
MAP3K6	GTAGAAGGCATCCAGAGCCG
MAP3K8	ATCAGTCAGATATGGAACTG
MAP3K8	CTTCGGTCATTTGAACACTT
MAP3K8	CCAGGGGATCAGGAGAACAT
MAP3K8	TGACACATGGTCATTAGACT
MAP3K8	TTAGTGGCCAAGAGGTACCA
MAP3K9	CTTAGCAGTCGCTTATGGAG
MAP3K9	CTGCTTATATCCATCTACCA
MAP3K9	TGCTGGACTTAAGGTCGCGG
MAP3K9	GCAGGAACTTCGCACCTGGG
MAP3K9	ACTGCAGCAGAAGAACCAGG
MAP4K5	GTATGACGAATGATTGCATG
MAP4K5	AATGAGAACACCTCATTGGG
MAP4K5	GAATCTCAATGAGCTACATG
MAP4K5	ATTTGTATGGAATACTGTGG
MAP4K5	AGACTGTAAAGCTCCACAG
MAPK1	ATCCAGACCATGATCACACA
MAPK1	CAACCTCTCGTACATCGGCG
MAPK1	GCTGACCTTGAGATCACAGG
MAPK1	CCTACTGCCAGAGAACCCTG
MAPK1	CTACTGCCAGAGAACCCTGA
MAPK10	CACACCCCAGAAAACGCTGG
MAPK10	CTGCTGTACCAAATGTTGTG
MAPK10	ACGTTATTACAGAGCCCCTG
MAPK10	CACATGCCAAGAGAGCGTAC
MAPK10	GGGATGGGCTACAAGGAGAA
MAPK11	GCTTCTGGACGTCTTCACGC
MAPK11	CTGCGGTCGCACCTACCCGG
MAPK11	TGCGCGCGTGGATCAGCGAC
MAPK11	CCAGACGGAGCCGTAGGCGC
MAPK11	TCTGCCCCGCCCCTACCAGA
MAPK12	CAGTGAGATGACTGGGTACG
MAPK12	TGGCCACCTTAGCGCCGGTG
MAPK12	CTCATGAAACATGAGAAGCT
MAPK12	TTGGATGCGCTACACGCAGA
MAPK12	CAAGTCCGTGAAGTCATCCA
MAPK13	TCCAGGAGCCCAATGACCTG
MAPK13	GACGTGCGTCGGGGACACGT

MAPK13	CGGATCTGCAGAAGATCATG
MAPK13	ACAGCTCGGCCATCGACAAG
MAPK13	ATGAGCCTCATCCGGAAAAA
MAPK14	TGATGAAATGACAGGCTACG
MAPK14	CACAAAAACGGGGTTACGTG
MAPK14	AAGTAACCGCAGTTCTCTGT
MAPK14	CAAGGCGAGTAATACCTGTC
MAPK14	GCTGAACAAGACAATCTGGG
MAPK15	CTGAACGCAGTCATCCGGAA
MAPK15	CCCTGGGCGACCTCCCCGAG
MAPK15	CTCGTGCCCACTCGTCGCTG
MAPK15	CTGCCCCAGATACACCCTTG
MAPK15	GGTATCTCCGGACAATGCGA
MAPK3	GCAGTTGCAGTACATCGGCG
MAPK3	AGTAGGTCTGATGTTCGAAG
MAPK3	TTCCGCCATGAGAATGTCAT
MAPK3	TGGAGGGCTTTAGATCTCGG
MAPK3	GGGAGCCCCGTAGAACCGAG
MAPK4	TGATCAGCATTACTCCCACA
MAPK4	CAGCTGTTAGGCGATCCATG
MAPK4	CAACATCGTCAAAGTGTACG
MAPK4	TCCTGGCTGAGATGCTTACG
MAPK4	CCATTGACACCGAAGCCCAG
MAPK6	CTGCTGTTAACCGATCCATG
MAPK6	AGCCAATTAACAGACGATGT
MAPK6	CATACCTTATGGGAATAATG
MAPK6	ATACTTGTAACTACAAAACG
MAPK6	CCATTGCCTCCACAACCCAA
MAPK7	GGGCCTGAAGTACATGCACT
MAPK7	TGACCGCGAAGCCCTCACTC
MAPK7	TGCCATCTCAGACAATACTA
MAPK7	GGCGGAGGACACCACTCCAT
MAPK7	CATGAAGTACTGATGTTCAG
MAPK8	TAGTGGATTTATGGTCTGTG
MAPK8	AGAATCAGACTCATGCCAAG
MAPK8	TGATATTAGATATTGATCAG
MAPK8	AGAAACTGCAACCAACAGTA
MAPK8	ATTCTGCTGGAATTATTCAT
MAPK9	AGTACCGTGTCACCACGTAA
MAPK9	CCGGGAACAGGACTTTATGG
MAPK9	AGAAACTTCAGCCAACTGTG
MAPK9	CTTATGTCAGGTTATTCACA
MAPK9	CCTTGGGCCCCAGAGCCAAT

MAPKAPK2	GTGTACGAGAATCTGTACGC
MAPKAPK2	TGTTATACACCGTACTATGT
MAPKAPK2	CCGGACTTGACGTGGAACTG
MAPKAPK2	GATCTTCAACAAGAGGACCC
MAPKAPK2	TCCGAAATCATGAAGAGCAT
MAPKAPK3	GTGTATGAGAACATGCACCA
MAPKAPK3	ACATAATAGGGAGTATAGCA
MAPKAPK3	CTTGGACAACTGGTAGTCGT
MAPKAPK3	CAGAAGCTGCAGAGATAATG
MAPKAPK3	GCTCCTGTATGACAGCCCCA
MAPKAPK5	GACGCCCTACACTTACAACA
MAPKAPK5	GGAGGAGAGACTCACCATCG
MAPKAPK5	GGGGTGTCATCAAGTCACCT
MAPKAPK5	CCACAGCCGGACTATCCCAA
MAPKAPK5	GCTGTGACTTGTGGTCCCTA
MARK1	GTAATGGAGTTTCTACACCG
MARK1	AGTCATGGAATACGCGAGTG
MARK1	AAGACCTCAGGCTAACAGTG
MARK1	CAATGCTACGTATCGATCTG
MARK1	CAGTCTGTGGACATATAGAA
MARK2	CAAAAAATATGATGGACCCG
MARK2	TGATGAACTAAAGCCTTACG
MARK2	AGAGGTATTTGATTACCTAG
MARK2	GTAACAACGCAGAAAATAAG
MARK2	GGTGGGCCAGAGATACAACG
MARK3	AGGACAGGTGGATCAATGCA
MARK3	TTTGACTATTTGGTTGCACA
MARK3	ACTGTTGAAAACAATCGGCA
MARK3	AGTGATCTCAACAACAGTAC
MARK3	GCAGATGAACAACCTCACAT
MARK4	CCCTCGACTCCCACTCCCCG
MARK4	AAAGACAAATGGATCAACAT
MARK4	TACTCACCTCCCGACCAGTG
MARK4	TCATGCGGCCATGCGACACG
MARK4	CTTGCCCTGAAACAGCTCCG
MARS	ATAACCCGAGACCTCAAATG
MARS	CAAACGAAATGTTAAACCAG
MARS	GATCGGCAGACTTTACACTG
MARS	AAGATGACCTCACTAACCAG
MARS	TGACAGCCCTTCCCTCAGCG
MARS2	GGTACCAGTGGAGAATCGCG
MARS2	TTGCCTTAACCGCTATACCG
MARS2	CGCCCTTGTAGAGCAGACCG

MARS2	TTTCCTGTATCTCTCGAGAG
MARS2	GCACGGGCTGAAGATTCAGC
MASP2	CCAACGAGAAGCCGTTCACG
MASP2	ACGACCGTACTGCTGCACGC
MASP2	CAGGAGGGCGTATATATGGA
MASP2	TGACGAAGTCGTACTCGCAG
MASP2	ATACTCCCCTGGAAAGCCGG
MAST1	GAGCTTGATGTGACCCATGG
MAST1	CTTCACCAGCTGAGTAACGA
MAST1	TGACGAGGATGACACGACGG
MAST1	TGGTGCCATAGCCAGATGAA
MAST1	CTACAAGGAGAGGTTCCCGA
MAST2	CAGTATGTCACGCTCCACGA
MAST2	AGCTGGCTAACGATGTAGCG
MAST2	GCACATCACCTACACTACCA
MAST2	CTCAGAGCGATACCACCACA
MAST2	GCTGAACTCCATCCTCCCGG
MAST3	CCCTGGTCGGCCAGTCACGG
MAST3	CTGACCGAAGAGTTCCTCGG
MAST3	TCAGCCCGGGCCGTGCAACG
MAST3	GGACGAAGATGACTGACAGG
MAST3	AATTCCGAGGACTATCCAAG
MAST4	AAACAGCTATAAGAGCCGGA
MAST4	CCATCGTCAGACACATCGTG
MAST4	AGTTGGCTAATGATGTACCT
MAST4	ACCCAGTCCGACCCACGGGT
MAST4	GGACGCGGCGACCAGCGCAG
MASTL	AAGCGATAACACTTGTCCTG
MASTL	TTTCCATCAGTCAAATCAGT
MASTL	GAAGGTGTGGGATTGACTAC
MASTL	ACACAAGAGCGTCCAGTGGT
MASTL	ATAGTGAAGCCCATTAGCCG
MAT1A	CAGAGACAAGAATGCACCTG
MAT1A	AGGAGTCTTCATGTTCACAT
MAT1A	GCACAACGAAGACATCACGC
MAT1A	TCACAAGCTCAACGCCCGGA
MAT1A	GGCTACGATGACTCAGCCAA
MAT2A	GTGCAGTATATGCAGGATCG
MAT2A	CCTGATGCCAAAGTAGCTTG
MAT2A	TCCTCATCAAGGTATTTCGC
MAT2A	CAGTCACCAGATATTGCTCA
MAT2A	ACAGCTCAACGGCTTCCACG
MATK	ATTGGGAGCACAGATCGGAG

MATK	ACACGGCCTCATCGATTGTG
MATK	CGCGTCAAGCACCACACCAG
MATK	TGAGACCAAAGCGGAAACAC
MATK	CCTGGCCCGAGATCTTCCCG
MATN3	CATTGGGCCAGCCGACACGC
MATN3	TAGAAAACATGCTCCTCTAG
MATN3	TTGATAGTTCTCGTAGCGTA
MATN3	CTCTTCTAACATCCCTAAGG
MATN3	ACATTGGGCCAGCCGACACG
MBD2	AGCCGGTCCCTTTCCCGTCG
MBD2	CCTCAGTTGGCAAGGTACCT
MBD2	CCTCTCAATCAAAATAAGGT
MBD2	CGAAAATCTGGGCTAAGTGC
MBD2	GCGACTCCGCCATAGAGCAG
MC2R	GTGGTTTCAAAACTGCCACG
MC2R	TCCTCCGGCAAAACCACACG
MC2R	TCACCATCTTCCACGCACTG
MC2R	TTGGCCATATCTGATATGCT
MC2R	AAGCAGGGAGAGGACAAACA
MC3R	GATGGTGACGTACCTGTCGA
MC3R	CTGGATAAACTGGTCCTCGA
MC3R	ACCACAGCATCATGACCGTG
MC3R	GGACACACTTACCAGCATGT
MC3R	GCAGGTCTTCATCAAGCCCG
MC4R	GTTTAATAGGGTGATGACAA
MC4R	TGTGCAGTCTGTAACTGCTG
MC4R	TAACATTATGACAGTTAAGC
MC4R	GAAAAGGCTACTCTGATGGA
MC4R	TGTCCACTGCAATTGAAAGC
MCCC1	AGAAGTTTGTAGACACACCG
MCCC1	GTCATGATTAAAGCCGTCCG
MCCC1	CGACCAGCTTCGCAATCATG
MCCC1	AAGGTCCTCATTGCAAACAG
MCCC1	GCTGGAGTACCTGTTGTGGA
MCCC2	GTAGGCACCTCCTTTGACGG
MCCC2	TTAGGTCATTGCTAGAATCG
MCCC2	TTGACAATCTCATAGACCCA
MCCC2	ACTTAACTAGGAAGGTTGTG
MCCC2	GTATCTGCTGAGGATCTTGG
MCL1	AGGCGCTGGAGACCTTACGA
MCL1	GTAATAACACCAGTACGGAC
MCL1	AGTCGCTGGAGATTATCTCT
MCL1	CCAAAAGTCGCCCTCCCGGG

MCL1	GTTTGGCCTCAAAAGAAACG
MDM2	GAGAACATTACCGGATTCGA
MDM2	TACCATGATCTACAGGAACT
MDM2	AGACACTTATACTATGAAAG
MDM2	CAACATCTGTTGCAATGTGA
MDM2	AGTTACTGTGTATCAGGCAG
MEFV	GATGCGACCTAGAAGCCTTG
MEFV	AGACTCCAGACCACCCCGAG
MEFV	GCTGCAGGAATCACGCACAC
MEFV	AGATGCCCCTCCATCCGGAG
MEFV	GCTGGCCGAGGAGCTCCACA
MELK	CTGTTACGCAATCATCATCG
MELK	TATCTGACGGAAGACAACCC
MELK	GCAGCACCTGAGTTAATACA
MELK	CTTCTAGCCAAGAAGGCTCG
MELK	GTGGCAGTATGATCACCTCA
MERTK	GCTACCGGATATCCCACGTG
MERTK	TAGTGGACAACTCTTCACAG
MERTK	ATGACTGAGCCATTACTCAG
MERTK	TCAGGCTGCTTAGTAAAGTG
MERTK	GTACCCACTGGCGTGAGGAA
MET	CCGATCGCACACATTTGTCG
MET	AGCTGTGGCAGCGTCAACAG
MET	CTCACTGATATCGAATGCAA
MET	TACTGTATTGTGTTGTCCCG
MET	TCAGCTTCCCAACTTCACCG
METAP2	TCTTGCTACTTCATCCACTG
METAP2	AATACCCACCCACACAAGAT
METAP2	TTATTGAGAGAACATCCAGT
METAP2	GTAAAAGATGCTACTAACAC
METAP2	ATATGTGACCTGTATCCTAA
MFAP4	TAGATGTCGTCACAGTCCAG
MFAP4	CTTCTGTGACATGACCACCG
MFAP4	CTTCTCCATCTCCCCGAACG
MFAP4	GCAGAAGTATGAGCTGCGAG
MFAP4	GCTTGTAGTCATTCCAGCCG
MGAM	TTCCATATTCGTAACGACTG
MGAM	CTACGAGGACAACAGCACTT
MGAM	GTTAGTGCTAGGCAGTCGAG
MGAM	TGGAGTGACTCCACTCATTG
MGAM	GGCTGTTGCTGGAATCCCCA
MGMT	CGCAAACGGTGCGCACCGCG
MGMT	GGTACTTGGAAAAATGGACA

MGMT	CTGCACGAAATAAAGCTCCT
MGMT	ACTCTTCGATAGCCTCGGGC
MGMT	GACCTCCGAGAACCGCAGCG
MID1	GAATTGGCTCAATCAGACGA
MID1	GAGATACTCACTAACGACGT
MID1	CCTTGTGTAAACTGGTTGGG
MID1	GTGTGATACTAGGATGCGGT
MID1	TCAAGAAGCCAAATTGACAG
MINK1	GGTGTTTGATGTGTGCCTCG
MINK1	CCCCGACCATAATGGTACAG
MINK1	GGGGAGCAGAGATACCCCTG
MINK1	CACTGCCCTTAACACCAGTG
MINK1	GACCGATCCCGGAAGAAGCG
MIP	TCTTTGCCACATACGACGAG
MIP	AGGAAACCTAGCACTCAACA
MIP	AGTGACTGCAGGATTGACGT
MIP	GGTAACGCTATACAGCACAG
MIP	CCTGGCCCACGCTCACCGCA
MKNK1	GTCTTTGAGAAATTGCAAGG
MKNK1	CCATCGCAGATGGTGACAGG
MKNK1	TCGGAGTAGGGTGTTTCGAG
MKNK1	GGCACCTTGAACTTTGGCAT
MKNK1	GTACACCCAATCTTACCTAT
MKNK2	ATCTTGTATATCCTACTCAG
MKNK2	CAGCGGCATCAAACTCAACG
MKNK2	TTGGACTTTCTGCATAACAA
MKNK2	GCTGATTGAGTTCTTCGAGG
MKNK2	AGGGGCAAGAAGAAGAAGCG
MLH1	TAATAGTAACATGAGCCACA
MLH1	ACTACCCAATGCCTCAACCG
MLH1	AGAAATCAGTCCCCAGAATG
MLH1	AATCTGTACGAACCATCTGG
MLH1	GTTAATGATCCTTCTCCGGG
MMAA	AAACCACTAGCATTTCGAGT
MMAA	GCAGCATCCACTTTGTACAA
MMAA	GAAAGATGAAGTGGTAACAT
MMAA	GAAGCTATTCTGTTGTGTGA
MMAA	GCAAAGGGCCTGTTTAGCAG
MME	GCACTCTATGCAACCTACGA
MME	GTTGTACCATAAAGGGCCTA
MME	TCTCGGCATCCATCCAAGTG
MME	AAGGGAGGCCAAGTCGAGGT
MME	GGAGCAGGACAAGGACCGAG

MMEL1	CACACGAGTGAACTACCGCA
MMEL1	CGAGAGTACTACTTCAACGG
MMEL1	GACAGGTGGAACGAGACCGT
MMEL1	CCAGAACATGGACCCGACCA
MMEL1	TTTGTAAAACGAAAACCCCG
MMP1	GATGCTATAACTACGATTCG
MMP1	CACACCTCTGACATTCACCA
MMP1	TACCCTAGCTACACCTTCAG
MMP1	AGGTGGACCAACAATTTCAG
MMP1	ACAACCTGAAGAATGATGGG
MMP10	CTGTGAATGAGTTGTAGAGT
MMP10	GGACAGAAGATGCATCAGGT
MMP10	GGACAAAGCAGGATCACACT
MMP10	GAAGCTAGACACTGACACTC
MMP10	CAAGTTCATGAGCAGCAACG
MMP11	TGATGAGACCTGGACTATCG
MMP11	ACCTTTACTGAGGTGCACGA
MMP11	GAGACTCAGTGGGTAGCGAA
MMP11	TCGGGCACGCCACAGCGGGG
MMP11	TGGACTATCGGGGATGACCA
MMP12	CACACCTGACATGAACCGTG
MMP12	TGGCCATTCTAGTGATCCAA
MMP12	AAATTCAGCAAGATTAACAC
MMP12	AGGGGATGCACATTTCGATG
MMP12	AGTAGGTCCTATAAAAACG
MMP13	AAAATTCAGAGGAGTTACAT
MMP13	CTACCATCCTACAAATCTCG
MMP13	AGTGGTCAAGACCTAAGGAG
MMP13	GATGCCATTACCAGTCTCCG
MMP13	GGAAGACCTCCAGTTTGCAG
MMP14	CTGCCCGATGATGACCGCCG
MMP14	GGCACCTCGCGGAAGCGCAG
MMP14	CCATCAACACTGCCTACGAG
MMP14	TGACGGGAACTTTGACACCG
MMP14	ATATGGCTACCTGCCTCCCG
MMP15	TCCCCACTGTGACGCCACGG
MMP15	CCTACCTAAAGGTCAGATGG
MMP15	GATCTGGGCGGAACGCATGG
MMP15	CGACGGGGACTTTGACACAG
MMP15	GGCCGAGGCCAAGATCTGGG
MMP16	TAAACTGCATCGATACTAGG
MMP16	GAATAGCTTTACGAGTCTCA
MMP16	GGTGTACCTGACCAGACAAG

MMP16	ATGACAGGCCAAAACCTCCT
MMP16	AATGCCATAGAACTGCTGCA
MMP17	TCCAAGGCCGACCATAACGA
MMP17	CATGGCTTAACCCAATGGCG
MMP17	TGAAGAAGAAAGCTTCACCC
MMP17	CGCACCGTGTCGTGCCCCAG
MMP17	TGCGCCCCTGAACTTCCACG
MMP2	GTCCGTCCTTACCGTCAAAG
MMP2	AGAATACCATCGAGACCATG
MMP2	GAAGTATGGGAACGCCGATG
MMP2	CTACGATGATGACCGCAAGT
MMP2	TGTCCCACTTGGGCTTGCGA
MMP20	AAAATTCACAAACCTGTGCG
MMP20	TTGGGTACATCAGTGCTGAT
MMP20	ATCCATTCGATGGGCCTCGG
MMP20	TTCCATGAGTTCTGTCGAGG
MMP20	GCATGGGCTAGAGTCCCCCG
MMP21	GCCCAGCTTGATGTCGACCG
MMP21	GGTTCCTGTCCAGATACGGC
MMP21	AGAGAGAAACCAATATGGAG
MMP21	CAGGACCACTGACCTTGAGA
MMP21	GACGCCGCTGGACTTCCGCG
MMP24	ATCACCCCCACTTAAGCCGT
MMP24	TCCCCCTTCTCCATCAAATG
MMP24	CCCGTAAAACTGCTGCATAG
MMP24	AAGTGGGTGAGCTAGACACG
MMP24	GCTGCCTCCCGGGCGCCGCG
MMP25	CGTCGCCGGTACGCTCTGAG
MMP25	AAGTCATGCAGAGGTTCGCG
MMP25	AATCCACCTCATGAAATGTG
MMP25	TGGGGAGCACCCCATCTCCG
MMP25	ATAGCTCATGAGGACCCGCA
MMP27	AAGGGACCATCAAAATAGCG
MMP27	TATCATAATAGATCCTCCAT
MMP27	TGAGGATGAAAACTGGACCA
MMP27	TAAGGAACCTGCTAAGCCAA
MMP27	GCATGGCCAAGCACTCCCAA
MMP3	TGATGATGAACAATGGACAA
MMP3	CTGTGAGTGAGTGATAGAGT
MMP3	TACCTGAACAAGGTTCATGC
MMP3	TCTGGAGGGACAGGTTCCGT
MMP3	AATCCTACTGTTGCTGTGCG
MMP7	ATGATTGGCTTTGCGCGAGG

MMP7	AGACTTACCGCATATTACAG
MMP7	AGGCATGAGTGAGCTACAGT
MMP7	TCTGCATTATTTCTATGACG
MMP7	TTAACATTCCAGTTATAGGT
MMP8	TGGGATACATCAAGGCACCA
MMP8	ACCCTAGAGATATCACCTGT
MMP8	GATGCTATCACCACACTCCG
MMP8	CATTCAGGCCATCTATGGTA
MMP8	GCATGAGCAAGGATTCCATT
MMP9	CCGCTATGGTTACACTCGGG
MMP9	CGAACTCATGCGCCGCCACG
MMP9	ACTACTCGGAAGACTTGCCG
MMP9	AGGTCGAGTACTCCTTACCC
MMP9	GATTGGTTCTCAGGTCTCCA
MOS	GTGTACAAGGCGACTTACCG
MOS	AGAACCGACTAGCATCTCGG
MOS	CCGCAGGGTCCAATAGCCTA
MOS	AGTGCACAATGCTTTGCGAG
MOS	TTATGGCCACAGGAACACCG
MPO	TCATTGTAGGAACGGTACGT
MPO	AGTAGGATAGGAGTTCCATG
MPO	AAGTAAGAGGGTGTGCATGG
MPO	GTTGACGCCAGTGACGAAGG
MPO	GCAGGTCTAGAGCCACGTGC
MRAS	ATCATCGGGAATGACTCCCT
MRAS	GGAGCAATACATGCGCACGG
MRAS	CAACCTCCCCACATACAAGC
MRAS	AATCTCCGTATGTTTCAGGT
MRAS	GCACTTGAGGAAGATCACCA
MS4A1	TGGGTGCATAGATCCCTGCT
MS4A1	TCATGAAGAAGCTTTGCGTG
MS4A1	GTAACAGTATTGGGTAGATG
MS4A1	TATTATTTCCGGATCACTCC
MS4A1	GATCATCAGAAGACCCCCCA
MS4A2	ACTGTCAGCCATGTATGCAG
MS4A2	AGTCTTGGAAATATCTCCCC
MS4A2	AGACAACTGTTCCAAAACAA
MS4A2	ACTTGATATTTCACACATTG
MS4A2	AAAAGAGCAGGAGTTCCTGG
MSR1	GAATACCTTCAAACAACAAG
MSR1	GATATAACTCAAAGTCTCAC
MSR1	ATCCCCCTCTACTTACTCGG
MSR1	GGAAATGCTCTGTGTCCATG

MSR1	GCAGTTCTCATCCCTCTCAT
MST1	ACTCGCGCCCTGACTCCGTG
MST1	AATGACTTCCAAGTGCTCCG
MST1	CACAGCCAATACCACCACTG
MST1	TGTAGCACCAAGGACCTCCG
MST1	TGCAATGGCGAGGAATACCG
MST1R	CTGCCCACCTAAGCTTACTG
MST1R	GTGGCATGTTAGTCACGGTG
MST1R	GTGGGTATCAACGTGACCGT
MST1R	CTCGGACCACATATTCAGGA
MST1R	ATACGTGCACAGCTTCCACA
MTAP	TCTGCCCGGGAGCTAAAACG
MTAP	AAATACCATACCTTGCAAGG
MTAP	GAAGGACTGAGGTCTCATAG
MTAP	GTCATAGTGACCACAGCTTG
MTAP	GGCTCATCTCACCTTCACGG
MTHFD1	CAGAAGCAAGTCATGCATCG
MTHFD1	CCGGCCTGATGGGAAATACG
MTHFD1	AGCTAATAACCTCGTTGCTG
MTHFD1	AGGGGAGTGGATCAAACCTG
MTHFD1	GCGCCAGCAGAAATCCTGAA
MTHFD2	GGGGCGCATGAACGTCCCGG
MTHFD2	GCTGGAAGGTCAAAAAACGT
MTHFD2	ATGTTACCGGCTACTCCATG
MTHFD2	CCTCTTACCGAACTGCCGCG
MTHFD2	CTATGTCCTCAACAAAACCA
MTHFR	GAGTTACATCTACCGTACCC
MTHFR	GGGAGGCTTCAACTACGCAG
MTHFR	CGGTGCATGCCTTCACAAAG
MTHFR	AGCACCGCCGTGAACTACTG
MTHFR	CAGGCCACAGTAGTTCACGG
MTNR1A	ACACCGACAGGATGACCAGG
MTNR1A	CTGATGTCGATATTTAACAA
MTNR1A	GTTTGTCGTACTTGAGACTG
MTNR1A	CTTTGTGGTGAGCTTAGCGG
MTNR1A	TCTTGTTCCGATACACCGAC
MTNR1B	AAGGTGCAGGAATAGATGCG
MTNR1B	AGGACACGACAGCGATAGGG
MTNR1B	CCCCTACCCGCTAATCCTCG
MTNR1B	ACCACCGAATCTACCGGCGC
MTNR1B	GCACGGAGAGGATCACCAGG
MTOR	TCAGGAAATGATCCGCACAG
MTOR	GGTGATGGCCTGGACAACCA

MTOR	CAGCATCGGATGCTTAGGAG
MTOR	GTGAAGGGGGTAATGTGACG
MTOR	CTGAAGACTGAGCAGAACCA
MTR	TTGGAGGAGTCGATGCACAA
MTR	TATGGATATCATCATACACA
MTR	GTGTACGGTCCAATCCTGAA
MTR	CACCTGCATTGGGATAACAG
MTR	CTAAACGAAGAACACTTCCG
MTRR	TAAACCAGGAATATTCAGAG
MTRR	GTCACTGGTATAGTCCACAA
MTRR	CTTGCACACGAATATGGTCT
MTRR	GTTAGGGCAGATCACGCTGA
MTRR	TGAGTCCTCACTTACCCGTT
MTTP	CCAGTTGATCCAAATAACGG
MTTP	AAATTGTAAAGTGACCTACC
MTTP	GAACATCCTGCTGTCTATTG
MTTP	CAGAAGGACATCCTTTACAG
MTTP	GCAGCATTATCCTCCAGGAG
MUT	GGAGTGAAGCCATTCACACG
MUT	TTTGATCTGGCGACACATCG
MUT	ATCATGTAAGAACCTCCCCA
MUT	CATATTTGAATATACAGCAA
MUT	GTATCTCTCTTGGAATACAA
MYC	CTTCGGGGAGACAACGACGG
MYC	AGAGTGCATCGACCCCTCGG
MYC	CTGCGGGGAGGACTCCGTCG
MYC	GCTGCACCGAGTCGTAGTCG
MYC	GGTAGGGGAAGACCACCGAG
MYH1	GTCCATCTATAAGCTCACAG
MYH1	GTTGCCAGTGTATAATGCAG
MYH1	GGATCCACTTCGGTACCACA
MYH1	TCAGTTGATCTTCTAGAGCG
MYH1	TTATGCCTTCGTCAGTCAAG
NAALADL1	GAAATCCGGAGACATTGGCA
NAALADL1	TACACCCCCATATCGAGTCA
NAALADL1	ACGCTACCCTTAGGGTGCAG
NAALADL1	TCACGTTCTCCTCAGTCCGG
NAALADL1	GCACCAGGTCCTCATCCCGA
NAGA	TGTCATCATAGTTACGCCAG
NAGA	CCTGAAGTTGGGTATCTACG
NAGA	CTGCAACATTAACTGTGATG
NAGA	ATTGATGACTGCTGGATCGG
NAGA	GAGCATGTCTACCTTCCACT

NAGK	TTGACGTAGCCGATATCATG
NAGK	CTCTACACCCCCATAGATCG
NAGK	AAGCTTCGCAGCGGTACCAG
NAGK	TGCTTGGTGTGCGATCCAGT
NAGK	AGACTCACCAAGCTTCGCAG
NAGLU	GGAGACCCCATACCGCCGGG
NAGLU	TGCACACATTCTGGTAATAG
NAGLU	TGCATGCTGCACAACTTTGG
NAGLU	CAGAGCGCGCTCCACCGACA
NAGLU	ACACGGAGAAGTCGGCCGCG
NAGPA	GTCCCTGAAGTGCGACACGA
NAGPA	GTGCTTACCAGTGATCTGAC
NAGPA	GGAACGTGGTGAGCGACGAG
NAGPA	GTCGTGTGGCTGATTCGTAA
NAGPA	TCAGGTGGCCGGCCACCGCG
NAPSA	AGGGACCGTGACTGGCACGA
NAPSA	ACATCCCTGTAGTTCGAGAG
NAPSA	CTCCACCAGTACATCCATCG
NAPSA	AGGCTTTGGGATCAAATCGG
NAPSA	CGTACCTCTCTCGAACTACA
NARS	AGCTGACAACCTGATCAATG
NARS	GGTCTCCAAGTACAACTGAG
NARS	GGTGCGTTAGAAGGATATAG
NARS	AGAGATCACTTCTTTGATAG
NARS	TTATCTTCAGTGTGTCTTGG
NDOR1	AGTTCGAAGAAGGAGCGGCG
NDOR1	CATCCCCGTTATCCGGCCGA
NDOR1	GCTGATTCAGCCCTCCAACT
NDOR1	CGGCAAAGTCCATCTGACAG
NDOR1	ACTCGGCTGCCCATGTCCAG
NEK1	ACAGTAGTTTAACTGATACC
NEK1	TTCAGGTGACAAGTAGTATG
NEK1	ATAACTGAGACACCAAACTG
NEK1	GCATTTGGTCAAAAATGGCA
NEK1	ACTAGAACGAAAGAGAAAGG
NEK11	CATTATCACGGAGTACTGTG
NEK11	CCAAGGCTATGACACAAAGT
NEK11	GAGTTGACTACATGCATGAG
NEK11	TCAGACAAGAAAGCCAAACG
NEK11	CCTGAGGCTCTGAAACACCA
NEK8	CAGGCATACACTGACGACAG
NEK8	CTGGTGCTGAAGATCATGAG
NEK8	AGGAAACGCGAGATGAACTG

NEK8	CACCGATCTTGACGACCATG
NEK8	GTACCTGCAGGTATCCCCCG
NEU1	CTGCGGAGGTCCATGGACCA
NEU1	AAGGCGAGAAGAGTGCCCCG
NEU1	CCCACTTCCGTAGCGCCAGG
NEU1	ATCCTTGCTCCATACCAACA
NEU1	ATATCCAGGGAGAGATTCCG
NFAT5	CAGGCCTGATAAAATCCATG
NFAT5	CAAGTCAGTCAAATTCAGAG
NFAT5	ATATGTTGGTCATGATAGGG
NFAT5	GAGTGCATTGTCTACCAATG
NFAT5	TGTGCTTTCTCAGCTTACCA
NFATC1	CACGAGGTTATCTCGATGCG
NFATC1	TCACCTCATGTAGGACGTAG
NFATC1	GTACGAGTAGTTGGACTCGT
NFATC1	GTTGCCCAACCACGAGTCGT
NFATC1	GTTCACGTCCCGCAACCCAG
NFATC3	GAAGTGTGACAGAAGATACG
NFATC3	ACTGCTGGGTTATGATATGG
NFATC3	CTCACCGAAATATAGGGGTG
NFATC3	ATGTGGTAAGCAAAGTGGTG
NFATC3	TGAAACTGAAGGTAGCCGAG
NFATC4	GGAGGTCGGGGAATACCGAT
NFATC4	CTGCAGATGAAAGGAACCTG
NFATC4	CCACATAGTCAAAGGGACCA
NFATC4	CTGTATGGTCCAAGCCCCGG
NFATC4	GCATCCCTCAGAAGACACGG
NFE2L2	CTTCCACTTCAGAATCACTG
NFE2L2	CACATCCAGTCAGAAACCAG
NFE2L2	GTAGCCCCTGTTGATTTAGA
NFE2L2	GAATTCAATGATTCTGACTC
NFE2L2	CATACCGTCTAAATCAACAG
NFKB1	TTGTCTATGAACATCTGTGG
NFKB1	TAGATGGCGTCTGATACCAC
NFKB1	AAGTAGGAAATCCATAGTGT
NFKB1	GGCACCAGGTAGTCCACCAT
NFKB1	AGTGACCTCACCATTCCCAA
NID1	TCATTACTACGTAAGAGTGG
NID1	ACAATCTGCAATAATCACCC
NID1	TGGACACGACCGATGGCCTG
NID1	GCAATCTGGTCATTAAGCAG
NID1	GTAGGGCTCAATGTGCACGG
NIM1K	CTTCGGAAAAATTAGCACTG

NIM1K	CTAAAAAAGAGCATCCTCGA
NIM1K	TTCCGGGACGAGCACTACAT
NIM1K	TAGGCTTCTACCGAATTCGA
NIM1K	AGTTAGACCAGAAAACCCAG
NLK	TGAATCCCGTCATATGACTC
NLK	CTGCTGTACAGGGTGTACAG
NLK	TTGACATGATCTGAGCTGAG
NLK	TCTTCAAAATAGTCAATGTG
NLK	GAATCTGGTCTCTTGCAAAA
NLN	AACCTAGAAAGGCTGTTACG
NLN	ATGCTGGGGAAAGTCTAGCA
NLN	AAGTCCCAACAACTCCTGGT
NLN	TCTATTTGGACCTCTATCCA
NLN	TCTCGTTCTGCTTCACCCAA
NMT1	GGGTTCGAGTGGTCTCAAGT
NMT1	GGGCTTTGGTAGTACCACCC
NMT1	AGGACAACAGCTACAACCGG
NMT1	GCATGTACATACCCAGCTTG
NMT1	TGTCCTTGTCAGGCTCCACG
NNMT	ACTAATCCAGACGGTGTGAA
NNMT	TAAAGGATTCACAAGCAGAG
NNMT	CTGAGCACGCAGTCAGCCGG
NNMT	CACACACATAGGTCACCACT
NNMT	GGAGACCTGCTGATTGACAT
NOS1	ACATCGAAGCGGCCTCTAGG
NOS1	GCTGGTGGAGATCAATATCG
NOS1	GATGTAGTTGAACATCCCGT
NOS1	CGGTCGTTCTCCACGCCGAG
NOS1	CATTGCCTCTGAGACCCACG
NOS3	TGAGCACTGAGATCGGCACG
NOS3	CCTCCCAGTTCTTCACACGA
NOS3	GTGTATGGATGAGTATGACG
NOS3	TGGATCCGGCCCACGCAGCG
NOS3	GTATTTCCACGGAAACTACA
NOTCH1	TGCAGGTCAGTACTGTACCG
NOTCH1	TCCTGCCAGAACACCCACGG
NOTCH1	TCGCACGCCTCCTCGATCAG
NOTCH1	TTGACGTCGATCTCGCATCG
NOTCH1	GTTCCAGTGCGAGTGCCCCA
NPBWR1	GGCTGTACCAGTTGTCTACG
NPBWR1	TTGGTGACGGTCTTCATGCG
NPBWR1	CGGCGCCAGAGTCGACGCGT
NPBWR1	GGTCGGCGCTCATGACGGTG

NPBWR1	CTACGCGGTGATCTGCGCCG
NPC1	AAAGAGTTACAATACTACGT
NPC1	CAAACTTGTATCATTCAGAG
NPC1	ACGCCATGTATGTCATCATG
NPC1	GAAGGTCTCACTAGGCATCG
NPC1	TGATACAGAGAAGCTCCAGA
NPC1L1	CTTCGGGCGACCATACCTGG
NPC1L1	AGAACTCACTACGGACCCCG
NPC1L1	CCTCAACAATTACCCTGCCG
NPC1L1	AGAGCCATACACGCCACACA
NPC1L1	GGTGCTATCTGTCATCCCGG
NPFFR1	AATGCCACATGCAAGATGAG
NPFFR1	TGAGCACGATGAAACAGACC
NPFFR1	CACGCTGACCGTCACCCGTG
NPFFR1	ATGACCACGATGAGCGCCAG
NPFFR1	GAGCACGATGAAACAGACCA
NPR1	CGACCGCCTCAATATTACGG
NPR1	ACGGAGACTCTGGCACATGG
NPR1	CAGCGTTCTTACCCCGCTCA
NPR1	CCTCAAGTCATCCAACTGCG
NPR1	GGTCCACCGTAATATTGAGG
NPR2	ATTCTTAGCCGAAAAACCAG
NPR2	CTCACCTGTAAACTCCCACG
NPR2	GCTCGCTCATGACAGCCCAT
NPR2	AATGAGACAATACAGGAAGG
NPR2	CGAATTGTGGAAAAGATGCA
NPR3	GCTGCGCAGAGCATACTCGA
NPR3	GTACTCGCACCTCACGCGCG
NPR3	CTTACGAGGATTCAGACTGT
NPR3	TCCAGACAGTCACTCTACTG
NPR3	TGTTCCCTGACCTTGCGCCG
NPY	TGCTAGGTAACAAGCGACTG
NPY	GGACATGGCCAGATACTACT
NPY	CGGCTTGGAGGGGTACGCCT
NPY	CGCACCCAGGCACACGAGCA
NPY	CTTCTGTGCCTGCAGATGCT
NQO1	GACAAAGGACCCTTCCGGGT
NQO1	ATTTCCAGAAAGGACATCAC
NQO1	GAATGACATTCATGTCCCCG
NQO1	GGACTCCAAACCACTGCAGG
NQO1	GCAGCGGCTTTGAAGAAGAA
NQO2	GGCACGCTGAACCAGTACAG
NQO2	TGAGCAGAAAAAGGTTCGGG

NQO2	GCGCTCCTTTCCGTAACCAC
NQO2	ATGCCATGAACCTTGAGCCG
NQO2	ATCCATCCAGCCCTTCAGGA
NR0B1	TAGCTCAAAGCAAACGCACG
NR0B1	GAGCACAAATCAAGCGCAGG
NR0B1	GAGCGCAAAGCAAACGTACG
NR0B1	GGTAAAGAGGCGCTACCAGG
NR0B1	GCAGCGGTACAGAAGCGCCG
NR0B2	GAAGTGCGTAGAGAATGGCG
NR0B2	ACCTCATCGCACCTGCCGGG
NR0B2	GGTGCTGCCTACATAGGCAG
NR0B2	CATACTCAAGAAGATTCTGC
NR0B2	AGATGCTGTGACCTTTGAGG
NR1D1	TTCTGGGGCTGCATACACGT
NR1D1	GCTGGGTGGAATGCTCCCAA
NR1D1	GTAGGTGAAGATCTCTCGAT
NR1D1	GAGTCTACAAGTGGCCATGG
NR1D1	TCCCCAAACGAGAGAAGCAG
NR1D2	TTCCTAAGCGTGAAAAACAG
NR1D2	CGCAAGCATGAACTCCATAG
NR1D2	AGTTACCTGTGCAACACTGG
NR1D2	TACTCTTTGAGTATAAGCAT
NR1D2	AGAACCCTCACTGTGACAAG
NR1H2	TTCCGGCGCAGTGTGGTCCG
NR1H2	CACAGACACGGCAAAGCTCG
NR1H2	TCACCCACTGTAAAGGAGGA
NR1H2	CATCTCAGTCCAGGAGATCG
NR1H2	CGAGGGTGTCCAGCTAACAG
NR1H3	CTACAATGTTCTGAGCTGCG
NR1H3	ACCAGATCCCCATAGCCGGG
NR1H3	GACTTTGCTAAACAGCTACC
NR1H3	CAGAGATCCGTCCACAAAAG
NR1H3	GCCCACAGCCCTGCTCACCA
NR1H4	TGTGTACAAGTGTAAAAACG
NR1H4	TAGGATGACGAGGAAATCTG
NR1H4	TGCATTATAGTGGTATCCAG
NR1H4	AATGGCAACCAATCATGTAC
NR1H4	GTTGGAATAATAGGATGACG
NR1I2	GGAAGAAAAGTGAACGGACA
NR1I2	CCACATACACGGCAGATTTG
NR1I2	AGGTTGACATGTCAGCCATG
NR1I2	CGAGGGAAGAAGCTGCCAAG
NR1I2	GATCATGTCCGACGAGGCCG

NR1I3	TCTGCGAAGTGTGTGACCAG
NR1I3	TCTTCAATTGTGTAGCGAAG
NR1I3	GTTCAAACATGGTGCCCATG
NR1I3	GTGAAGTCAGCAAGACTCAG
NR1I3	GCTGATCCGGACACTCCTGG
NR2C2	CCCCAGTAAACGCTCCACAG
NR2C2	CCAGTCGACACCCATCATTG
NR2C2	TCTTTGTCTGCCACAAACGT
NR2C2	GAACGTCACCTTAGAATCCG
NR2C2	TCAGCCGGCAAAACTGACAG
NR2E1	TGATTCACACACCGACTCCG
NR2E1	CGGTGTCCACCACTCCAGAG
NR2E1	CTACTTCCGTGGACACAAGG
NR2E1	GAAGTCAACATGAACAAAGA
NR2E1	GTATCTCTATGAAGTGGCCA
NR2F1	CCAGTACGCACTCACCAACG
NR2F1	ATGTGTAAGTTAAGTTCCTG
NR2F1	GGCTGCCGTAGCGCGACGTG
NR2F1	GGTCGGTGATCTGCAGATCC
NR2F1	GCGAGATCCGCAGGACGACG
NR2F6	GCGGCGGAGACCTCTTCCCG
NR2F6	GCTGGTCGATCTGGCAGTCA
NR2F6	CGAGCTCTTCGTGCTGAACG
NR2F6	AAGTCGAGCGGCAAGCATTA
NR2F6	GCTATGGCCATGGTGACCGG
NR3C1	TAGAAAAAACTGTTCGACCA
NR3C1	CATCGAACTCTGCACCCCTG
NR3C1	ATCAACAGGTCTGATCTCCA
NR3C1	CTTTAAGTCTGTTTCCCCCG
NR3C1	TCTCTTGCTTAATTACCCCA
NR3C2	TCCACTAAAGTATTGACAGG
NR3C2	AGAATCCATATATAAACCCA
NR3C2	TCCCCTAATGTTGAAAATCG
NR3C2	TCTGGGAGCTCCGTGAATGG
NR3C2	ACACAGAGTTGATTCCAGCA
NR4A1	TACACCCGTGACCTCAACCA
NR4A1	GGCTAACAAGGACTGCCCTG
NR4A1	TCGCCCAGCCAGACTTACGA
NR4A1	GTCCAGGTGTGCACGGACCA
NR4A1	GAAGTCCTCGAACTTGAAGG
NR4A3	GAAATCGACAGTACTGACAT
NR4A3	TGGTCAGCTTGGTGTAGTCG
NR4A3	CGGGTGGCTCTCAAGCGCGG

NR4A3	CCTGCGTGTACCAAATGCAG
NR4A3	ATACAGCTCGGAATACACCA
NR5A1	CACACGTGAGCAGTCCGTAG
NR5A1	GTGCGCGCTGACCGTATGAG
NR5A1	GGGGCCCCAAAGTCGCCCAG
NR5A1	ACGTTGGGCCCTCCAGAGAA
NR5A1	CCGCTTCCAGAAATGCCTGA
NR5A2	AGGGCCGACCGAATGCGTGG
NR5A2	GGGCATTGTCATGCTAATGG
NR5A2	GGGACTGGCTCGATCGCATG
NR5A2	GAATAGCCCATTATGGACTC
NR5A2	GTAAGGGCCGACCGAATGCG
NR6A1	CCTCACCCACCGTTGCGCGG
NR6A1	CACCCATCTTCAATCAACAT
NR6A1	ACGAACCTGTCTCATTTGTG
NR6A1	CCAGAATAGCTAAAAAGGTG
NR6A1	CATAGTGCAAGCCTGTAGCG
NRAS	CCATGAGAGACCAATACATG
NRAS	TGAATATGATCCCACCATAG
NRAS	TGATGTACCTATGGTGCTAG
NRAS	TTGCGGATATTAACCTCTAC
NRAS	GGATTCTTACAGAAAACAAG
NRBP1	TCTGTGATAAAAATGACCTG
NRBP1	GCACTTCAAACAATGCTGGG
NRBP1	AGGTATGCACTGTCAATACC
NRBP1	CACTAATGTGACAACAGCAG
NRBP1	ACCATCTTCATCCAGCACAA
NRBP2	ACTGGAAATCCAGACCAATG
NRBP2	GGTTCCCGTGGATGATTGGG
NRBP2	TAAACCAAGGGAACATGCCA
NRBP2	AGTGCACCTCGAAGAGCACG
NRBP2	GAAGTTCCTCTCGCTCAGCG
NRCAM	CAGCGGGACCTACACGTGTG
NRCAM	GCAGGTGTGAGGATTCGTGG
NRCAM	GAGTCTATCAGTGTACAGCA
NRCAM	CTGATAATTTGGTGATTACG
NRCAM	GGATTACAATATTCTCCCGA
NRK	TGTTCCAAAAGAGACCGCTG
NRK	TTGGACACTAGAACCCCCAC
NRK	AGAACCACAAGATTTGGACC
NRK	AGAAGCTCGTGAGTGCAAGA
NRK	GCTCCCACAGTCAAATCCAG
NRP1	GATCGACGTTAGCTCCAACG

NRP1	GCTGTCGGTGTAAAAAACCA
NRP1	AAGATCGGGTACAGCAACAA
NRP1	CAGATCACATCATCCAACCA
NRP1	TGTCCTCCAAATCGAAGTGA
NT5C2	GAGTCACATACGGTACCTTG
NT5C2	ATCGTCGAGAAGCCTATCAT
NT5C2	GCAAAGCTGAGCAACTCCTG
NT5C2	CAGCATGGTATCGTCTACTC
NT5C2	CTGTAACCGATCACTCCAGG
NT5E	GCAGCACGTTGGGTTCGGCG
NT5E	CCGCTTTAGAGAATGCAACA
NT5E	AGTGAGGGGTGTGGACGTCG
NT5E	CCTGATATTTGAGATGCTAG
NT5E	CTATGTGTCCCCGAGCCGCG
NTRK1	CCCTTTCGAGTTCAACCCCG
NTRK1	GCGCAGACACCCGTGCCGCA
NTRK1	AGGGCACAAGAACAGTGCAG
NTRK1	CTGGAGCTCCGTGATCTGAG
NTRK1	AGGTCTTCTCACCATCACCG
NTRK2	TGAATGGAATGCACCAGTGG
NTRK2	ACGTCACTGATAAAACCGGT
NTRK2	AACCTGCAGATACCCAATTG
NTRK2	TTGGTGATGCCAAAGTACTG
NTRK2	GCTGGTTGTGGGCTTCTGGA
NTRK3	TCTTCACACGCTCAACGCCG
NTRK3	CGTCAACCTGACCGTACGAG
NTRK3	CATGTGGAATACTACCAAGA
NTRK3	TCATGCCATCAACTTGACGC
NTRK3	AGACTGAGATCAATTGCCGG
NTSR1	GACCGTCATGGTACGCCAGG
NTSR1	CTGTATGACGACCTTGACGG
NTSR1	CTACGCCACGGCCCTCAACG
NTSR1	GAAGAGCGCCAGGTACACGG
NTSR1	GCTCGCTGCTGGGTGCCGCC
NTSR2	GGAAAGCAGTTAGTGCCAAG
NTSR2	CAGCACCGTGCACACTCGCG
NTSR2	CGTGCACGAAGTAGTAGCCG
NTSR2	GGTAGAAGTGGACGGCACTT
NTSR2	CCAGGTCGCCGAAGACCCAG
NUAK1	AAGTCAAGCGGGCCACCGAG
NUAK1	AGTGTAAAGCAACACACCCA
NUAK1	TAGATGCTCGAGGACTCATA
NUAK1	GGCCAAACCCACGACCTCTG

NUAK1	TCAATGGGAGACCTTACCGA
OAT	ACAGACCCAACCAGTTACGA
OAT	TAAGTGGGGCTATACCGTGA
OAT	TGCTCTTCAGGATCCAAATG
OAT	TGACAGCACTGTAAGAACTC
OAT	GATTGGCCAGAACTGGTAGA
OAZ1	TGATCGGCTGAATGTAACAG
OAZ1	GTGGGCGAGGGAATAGTCAG
OAZ1	CTCTACATCGAGATCCCGGG
OAZ1	CTCCACTGCTGTAGTAACCC
OAZ1	GCTTCGCCAGAGAGAAGGAA
OBSCN	ACACACACACGAGTACTCCG
OBSCN	CAGCTCGAAAGTGCGCATGG
OBSCN	TGCCGGGGAGTATAGCTGCG
OBSCN	AGAAGATGTGAGAAATCACG
OBSCN	ACTGACCTGAAACATCCAGG
ODC1	CAACGCTGGGTTGATTACGC
ODC1	CTGCACGAAGGTCTCAGGAT
ODC1	GAAGGGGCTTTACATGTGCG
ODC1	ATGTATCTGCTTGATATTGG
ODC1	GCATAAAAGGGGGTGACACG
OMG	AAATACACTAAGAAGTCTCG
OMG	GCCCTCCAAACTACATATCG
OMG	TAACCCAATATACCAATCTG
OMG	ACTTACAGTGAATAAGCTTG
OMG	GACATGTTCCACAGAGACCG
OPN1SW	ACCACGTATAGGACTCGCTG
OPN1SW	CTGAAGCGGAAGTTGCCGAA
OPN1SW	GACGAAGTATCCGTTACAGC
OPN1SW	CCACAGGTCTGGTTACAGGA
OPN1SW	TCAAAAATATCTCTTCAGTG
OPN4	ATGGTCTGGAACATCAACCG
OPN4	CATGTTCATTATCAACCTCG
OPN4	TGTGGCGTCCAAGAGGCGTG
OPN4	GAGCTGTCCCACCAGCTGGG
OPN4	TGAGGAAGTCGCTGACCGCG
OPRD1	ATGGCTGTGACCCGTCCCCG
OPRD1	GCGCTGGCGCCAATGCGTCG
OPRD1	AAGGTACACTAAGATGAAGA
OPRD1	GTAGCGGTCAACACTCATCA
OPRD1	GCACACGGCCGAGTAGAGCG
OPRK1	CTACTCCGTAGTGTTCGTCG
OPRK1	AGATGATGACTACTCCTGGT

OPRK1	AGTCCAAAGCCTTCACGGGG
OPRK1	AAGTAGACCGTACTCTGAAA
OPRK1	GGAAGTCCAAAGCCTTCACG
OPRL1	TACATAGCGATCCACACTCA
OPRL1	AACGGGAACACCGACAACAG
OPRL1	CGGGCCCCAGTAATCCTGAG
OPRL1	ATGGTGACCTTGAGCCCGAG
OPRL1	GATAACCTCCCAGAACGGCG
OPRM1	GGCAACCTGTCCGACCCATG
OPRM1	GGAAATCTAAGGCCTTGACA
OPRM1	GATCATCAGTCCATAGCACA
OPRM1	TGGAGATCACTATCTTGCAA
OPRM1	CCAGAGTGTGAATTACCTAA
OTC	GAATGAAAGTCTCACGGACA
OTC	GAACACTATAGCTCTCTGAA
OTC	CAGCCCATTGATAATTGGGA
OTC	AACTTGGTTACACTAGCATC
OTC	GATGGATGCTTCTTTAGCCA
OVCH2	CATGTGCCGGAATAAGAAAG
OVCH2	AATGTCTGTGAAGATCCCAG
OVCH2	CTTCCTGCAAGACTTGTGAG
OVCH2	ACCACAGTGGGTGATCACGG
OVCH2	ATGTCTGTGAAGATCCCAG
OXGR1	CATTGCCTGGAAATCCCACG
OXGR1	GGTGAATGATCACACAGTAG
OXGR1	CAAAGTGTCACTATCACCAA
OXGR1	AATGATCCACACCACAGCAC
OXGR1	TAATGCCATAAATAACAGGG
OXSM	GTTTGGGATCGTCTTATCGG
OXSM	AATGGTCGACATGCCAACTT
OXSM	CAGCATTCGATATAAACTCA
OXSM	TGCCAATATCCAGATTGCAT
OXSM	GTTGCTGCTTATGTGCCAAG
OXT	GACTCACCTTGCGCACGTCG
OXT	AGATATTGGGCCCGAAGCAG
OXT	GCAGTTCTGGATGTAGCAGG
OXT	CGGGCCCAATATCTGCTGCG
OXT	GCGCACGTCGAGGTCCGGCG
OXTR	CGGACCCCCGCGGCGCAACG
OXTR	CCTGCAAGTACTTGACCAGG
OXTR	GCTAGCTGTCTACATCGTGC
OXTR	GCGCGAGTGCTTCTGGCGTG
OXTR	ACGTGGCGAGCACTGCCAGG

P2RX2	GAAGCGCTGCACGTTCCACG
P2RX2	GCTGTGTGCCCTATTACCAG
P2RX2	CACGTCCGAGCACAAAGTGT
P2RX2	CAGCCAATTTCTGGGTACGA
P2RX2	GGAGTACGTGAAGCCCCCCG
P2RX3	GGGACGTGGTCAAGTTTGCG
P2RX3	TACTCGGTTGATGATCCCGA
P2RX3	CTTCACCTTGGTTACCACCG
P2RX3	CTCACTTTAGGGATCCTCAC
P2RX3	GGTGATGATGACAAAGACCG
P2RX4	CGGGTCTGTCAAGACGTGTG
P2RX4	TCACGTTGGTCATGACGAAG
P2RX4	GGTCAGCTCCGTTACGACCA
P2RX4	GGCATCTGATTTACACACAG
P2RX4	GGATGAGCAGTTGCACGGCG
P2RX7	TGAGTGACAAGCTGTACCAG
P2RX7	CAAAGGGAAGGTGTAGTCTG
P2RX7	CAGAAGGTACCTTTGCTCTG
P2RX7	GTAGTATTCGTTGACCACAC
P2RX7	ACGCTCTGTTCCTCTGACCG
P2RY1	CAAGGCGCATTTGAACGACG
P2RY1	TAAAACAGACTGGATCTTCG
P2RY1	AGCCCAGAATCAGCACCAAG
P2RY1	ACTTCGCAGGTACTCGTCTG
P2RY1	CGAGGAGGAGAGAATGACCG
P2RY12	CCGGTCATACGTAAGAACGA
P2RY12	CCAACCCCAAAAATCTCTTG
P2RY12	GATCGATAGTTATCAGTCCC
P2RY12	CAGAGACTACAAAATCACCC
P2RY12	TGATAAGTCCAACAAAAAAC
P2RY13	CATGAGTGTCATTATCAAGT
P2RY13	ATTCCCAGCCCTCTACACAG
P2RY13	AAATACGATCTTGAGCAACA
P2RY13	ATGAACACCACAGTGATGCA
P2RY13	GTTGAAGCCTTGCATCACTG
P2RY14	ATCCTGACACTCCATTGAGT
P2RY14	TAATATTTGGAACAGCAAGG
P2RY14	AAAGTGAACTGGGACGGAAG
P2RY14	ATTCCGACTTGACTTAAGGT
P2RY14	TGAGAGCAGGATTCATCTGG
P2RY2	CTGGTCTATTACTACGCCCG
P2RY2	TGGGCTGTGTCTGAACGCCG
P2RY2	CGTAACCTGCCACGACACCT

P2RY2	CACCACATATATGTTCCACC
P2RY2	CTACAGGTGCCGCTTCAACG
P2RY4	CCACTTCGGGCACTACGCTG
P2RY4	GGGCTGCATAATAGTAGATG
P2RY4	AAGACAACTGCATAGCTCAC
P2RY4	GTCAAACTCTTCAGGCCGAG
P2RY4	TTGCAGATCTCAGTGCCAAA
P4HA1	ATTATTACCATACGGAACTG
P4HA1	AGCTATGCGGTATATCAGCA
P4HA1	TCATGGAAGCGAATAATACG
P4HA1	GTACGAAATGCTGTGCCGTG
P4HA1	GTTCCATCCACAGTTCCGTA
P4HA2	CCCAGGCACAATTTCCAGAG
P4HA2	GCTCATTGCCCCCTTCAAAG
P4HA2	TTACGAGAGCCTCTGTCGTG
P4HA2	AGCAGCTGACTTGCTAGTCA
P4HA2	CCAGTTTGTAGGCATTCACA
P4HB	TCGGTGAACTCGATGACAAG
P4HB	CCACACCTGTATATTCCTTG
P4HB	AGAAGGTTCCGAGATCAGGT
P4HB	ATACCAGCTCGACAAAGATG
P4HB	GCTGCCACCACCCTGCCTGA
PADI4	CCATACACTGGTGCTCCACG
PADI4	AGCTCTACTCTACCTCACCG
PADI4	CCCACACAAAACGCTGCCCG
PADI4	CCATGTTGTGCTTTCCACCG
PADI4	TCATGATCCAGGGCGCCACG
PAH	AGAAGGTCTAGATTCAATGT
PAH	CGGGCCATGGACTCACAGGG
PAH	GCCACCCAAGAAATCCCGAG
PAH	CACGGTTCGGGGGTATACAT
PAH	CCTCCATGTATTCCACTCGA
PAICS	ATTGGAATCATTTCACACTG
PAICS	TGCAGCTAGAAAAAACCACC
PAICS	TACGAATTGTTAGACAGTCC
PAICS	GTGGGTTGCAGAGAGAGTAG
PAICS	GTTGCCCCAGAATTGTACAC
PAK4	GGACGAGTTTGAGAACATGT
PAK4	CCAGTGGCAGAGCCTGATCG
PAK4	CCGGTTCGCCGGTCACAGCG
PAK4	TGCTCATGGGATACTCGCTG
PAK4	CGTGGGGTCCCAGCACTCCA
PAM	GTTCAGAACCATACCACCAG

PAM	CATGTCTATGCGAATACCAG
PAM	GTACTTACGGCAGACGTGTG
PAM	ACAGACTAGTATCTACCTTG
PAM	ATACACATCTTGCAGCCAGT
PAOX	CTGGAACGGGTCCTTCCAGG
PAOX	GAGATGGCGACTCTGTTCTA
PAOX	GTGGAGACCGGGGGTCACGT
PAOX	AAGACGGGGTTACCCCGGGA
PAOX	TACCGAAGCAGCGCTCCGAG
PAPPA	TCTTATATCTCACGTGACCG
PAPPA	CACAATGTCTGTCCGCACAG
PAPPA	TAAGCCCCTGAAGTATAAGG
PAPPA	ACATGGACTGCAACTATGAA
PAPPA	AGATGATCATAAGAACCCGA
PARK7	GTTACAGGGACCATATGATG
PARK7	GGGCGCACAGAATTTATCTG
PARK7	TCATCCCTGTAGATGTCATG
PARK7	AGTACAGTGTAGCCGTGATG
PARK7	CTCGCCTCATGACATCTACA
PARP1	CGATGCCTATTACTGCACTG
PARP1	TACCGATCACCGTACCCACA
PARP1	AGCTAGGCATGATTGACCGC
PARP1	GGCCATGATTGAGAAACTCG
PARP1	GCAGAAAGTCAAGAAGACAG
PASK	ACGGACCCGTCCGAACCGCG
PASK	CTCAGGGCCAGCATCCAACG
PASK	AGCCACGGCTCATCTAGAGT
PASK	AAGGCCCAGCTAGAGCGGAT
PASK	GAGGTCTGTTGGAAGAGCCA
PBK	AAGCTTCTGCATAAACGGAG
PBK	CCACTGGATGAAAATATGAC
PBK	TGTCTTGCTATGGAATATGG
PBK	TAAATGTGTACCTAATGAAA
PBK	GAGTGGCTTTCACAATGGAA
PBRM1	ATGTGCGATGTAAGCCTGAG
PBRM1	GGCACTACCAGTATCAGAGG
PBRM1	AGGAGTTGTCGGAATAACCA
PBRM1	CAAATCCCAGAGTTTGCAAG
PBRM1	GGAGTTGTCGGAATAACCAA
PC	GGTCCATGCTCAGATCCACG
PC	CATCTCATACACGGGCGACG
PC	CAGAGTAGATGGCTACGGTG
PC	TCGGACGCGGAACTCCGCAA

PC	GCTGGAGGAGAATTACACCC
PCBD1	CACTCTTGTCATGAACCCAA
PCBD1	CGTTAAACCATTCAGGATGG
PCBD1	AGCACACAGGCTGAGCGCTG
PCBD1	CACCCCACAGCCCTCAGGTT
PCBD1	CCAGCTGCTGCCAAACCTGA
PCCA	ACCTGGATTTCTATATGACG
PCCA	GTAAACCAAAAGACTTGTAG
PCCA	GGAAGCCATTAAGAAAACCA
PCCA	ATATGGCTCTGATAGAACTG
PCCA	GCAGCAGCTGATGCTGAGCG
PCCB	GAAATCCCGGAGATTACACA
PCCB	CATGGACCAGGCCATAACGG
PCCB	TTCGATGCGTTCGTTAACAG
PCCB	ATCAACCAAAGCCTACAACA
PCCB	GTAGTTTCCTGGAGACAGCG
PCMT1	ATTGTGGAGAATCCATGTAT
PCMT1	AACCACCTTCAGCGCGACGA
PCMT1	GATTGTGGATTAGCTCCGAG
PCMT1	GGCGGCGACGGCAGTAACAG
PCMT1	GCGATGGCCTGGAAATCCGG
PCNA	TGCTTCAAATACTAGCGCCA
PCNA	ATACGTGCAAATTCACCAGA
PCNA	TGGCCAGGTTGCGGTCGCAG
PCNA	GCTGGAGCTAATATCCCAGC
PCNA	ACTAAGGGCCGAAGATAACG
PCSK1	GGCCAGCGGGTCATACTCAG
PCSK1	GAGCACTTCTCAGCGTACCA
PCSK1	TCGTTTGTGGGATCATATCG
PCSK1	TACCATTGCTGATTCCACAT
PCSK1	GCACTTCTCAGCGTACCAGG
PCSK2	GGATTTGACCGAAAAAAGCG
PCSK2	TGCTCACCACACCGGCCTCG
PCSK2	AACCAGTCATCTGTGTACCG
PCSK2	CTTGTGGAGTTGCATAAAGG
PCSK2	GACACAGAAGAGGAACCCGG
PCSK5	GCAGTGCGAATTGTTTGCAG
PCSK5	GGAAGAGTGTTCATCCACGC
PCSK5	TATTGTCAGGACTTCCCGTG
PCSK5	ATATTCATGTCAGACTGGCA
PCSK5	CTGGGCAGTCAAAATCGCCG
PCSK7	TCAGGCTGCCTTACAACATG
PCSK7	GCGATGTGCAGGAGAGATCG

PCSK7	TGACGTAGGATGCTAAGTAA
PCSK7	TGGGTGACTACCTATGGTGA
PCSK7	GCCCGGCTAATTCCAGCCAG
PCYT1B	GCCCGACGTAACCTCCAGAG
PCYT1B	GCCTCCCTCAGAAACCATGG
PCYT1B	CCTCACCTGGTGTTCCTAAG
PCYT1B	AACAGCTACTTGTTGGTAGG
PCYT1B	ACTGATGCTGAGTCAGAAAC
PDCL	GAAGGCATCTCAGTTAACAC
PDCL	GACCACGAGGACAAGGACCG
PDCL	GCAGTACCGGAAGCAGCGAA
PDCL	GGAGTTTGCCATAATGAATG
PDCL	GTACTACTATAGCAGCAGTG
PDE10A	TGTGTATATTCACGCCACCT
PDE10A	CTTACTTTGATACGTCGAGT
PDE10A	TGGCCAAGTAGCAAGAACAG
PDE10A	GAACAACGGTTGGACACAGG
PDE10A	CATGCACAGGATGTTCCGCG
PDE11A	CCCTGAGTAGTGTACGACGG
PDE11A	ATGGCAAGTTCTGACACCAG
PDE11A	AGAAATCATCATACTCCGAC
PDE11A	AATGCAAAGTCCTACCTGGT
PDE11A	GCAGTGCTGATGCTGAGAAC
PDE1A	CAGTTTATATCGATGAAACA
PDE1A	ATTGTGCATGCTGTTCAAGC
PDE1A	GTCGATAAGCTGCACTCACG
PDE1A	TTCATGATTATGAGCATACA
PDE1A	CTACCTTTACACGGAAAATG
PDE2A	ATGGTTCAGCAAGTTCGACG
PDE2A	GCCGCGGTAGAAAAGCGGAT
PDE2A	CATGCCGCTAGCGGACAAGG
PDE2A	TGCAGGGGAACTCTACGACC
PDE2A	GCTCCAGGAGATCATCACGG
PDE3A	ACAAAGCCTAGGTTCTCACA
PDE3A	TCAGAATGGGACCACAAACG
PDE3A	CAGCACCAGCCATGTCGCGG
PDE3A	GTTAGCTGCAGAAATAGCAT
PDE3A	GGTCACAGCCGACCTCCCCG
PDE3B	CTGTTGAACAGTCTTCAAGG
PDE3B	GCTACTAATACTCCGTAGAG
PDE3B	AAGTGCCTGTGATCCGACCC
PDE3B	CCTGCGTCGCCATCCCCCCA
PDE3B	GGTCCTCTATTTCAGAACAG

PDE4A	GACATACATGCTGACGCTGG
PDE4A	TCGGTCTTCACCCCAAATCG
PDE4A	CACAGTGCACCATGTTCCGG
PDE4A	GCTCATGTACAACGATGAGT
PDE4A	GGAACTGGTTGGAGACCCCA
PDE4B	GCAGCGTCGTCGCTTCACTG
PDE4B	TGTGATATGCCACGTCAGAA
PDE4B	AAACGCTGGAGGAATTAGAC
PDE4B	GCAGTTTGAAACCCACAGCA
PDE4B	CATCTCACTGACAGACCGGT
PDE4C	GCGGACCGTTCGGAGCAACG
PDE4C	TGCAAGCGCCATCCACGACG
PDE4C	CTAGAAGACACCAACAAGTG
PDE4C	CGGACACCTGGTTCCCGGAG
PDE4C	ATCCGCAGACCAGCAGACCG
PDE5A	TGTTGCTGAAGGTTCAACAC
PDE5A	GGGGCACTGTTATCTGCACG
PDE5A	AAGAGAGCTACAGTCGTTAG
PDE5A	AATTAAGAATCATAGGGAAG
PDE5A	TCAACTTCTGCATTGAACCG
PDE7A	CCATAACGCAGTCCACGCTG
PDE7A	GGATTTGAATCAGAAAGAAG
PDE7A	GTGAAGATCTAAGATATCGC
PDE7A	TGATTATAATGGACAAGCCA
PDE7A	GATCCACTTGATAAGAACCA
PDE7B	AATCTTCTTGAACCATGACT
PDE7B	CACAGGTACAACATACTCAG
PDE7B	GCTCTCCAAAGTGGGAATGT
PDE7B	ATGGCTGGTTCACCCCTGGG
PDE7B	AGACTACCTTGGACAAGCAA
PDE8A	TTCATGCATCAGGATAGGCA
PDE8A	CTGTGGTGAATATAACTCAG
PDE8A	GTTTAAGTGTACAGTTACCA
PDE8A	ATGAACTCACCTATTGTGGG
PDE8A	GCAGGGCATGTGAAAAAGCA
PDE8B	CCAACAAGATCACTGGTGTG
PDE8B	AGTAATTGAGAGATACAAGG
PDE8B	CGGGGCATTTACCTTCACGC
PDE8B	ACACTCGCGAAACCACTGCG
PDE8B	GCTGGGCAGCGGTAGCAGCG
PDGFRA	TAAGTCAGGGGAAACGATTG
PDGFRA	CCTGCGTTCTGAACTCACGG
PDGFRA	GACTTGGTCGATGATCACCA

PDGFRA	AAATAATCCGTCATTCCTAG
PDGFRA	GTACACTTTGACGGTCCCCG
PDGFRB	TGTGGTAAGGCATATCCAAG
PDGFRB	GACTAACGTGACGTACTGGG
PDGFRB	GTCCCCTATGATCACCAACG
PDGFRB	CTCCCGTGTCTAGCCCAGTG
PDGFRB	AAAGGCCATCAACATCACCG
PDIA2	GCTGCAGTACTTTGGACTCA
PDIA2	ACGCTGAGGGCATTGCCGAG
PDIA2	GGCCAAGCTCCTCGTCCACG
PDIA2	AGCTGCCTTGCTGTACTCGG
PDIA2	CCAAACTGCTGAAAGAGCCG
PDIA3	ACAGAGCAAAAAATGACCAG
PDIA3	CTCCGACGTGCTAGAACTCA
PDIA3	ACCCTGAAGATATTTAGAGA
PDIA3	TTTCGATGATTCATTCAGTG
PDIA3	GGCAGAATGGACTCACCAGG
PDK2	CAGGAGCTTAGCCATGTCGT
PDK2	CTCCAAACAGCCGATTCACA
PDK2	CTTGAGTACAAGGACACCTA
PDK2	GGTGCTGCCATCAAAGATGA
PDK2	GGAACTCCATGATGTCCAGG
PDK3	ACATCCGCCACGTTACAGGT
PDK3	TAAGCATGCGGAAAGAGATG
PDK3	TTACTTAACCGCCCTTCAGT
PDK3	TAGCATATGAAACAGATGTG
PDK3	GAACCAATCCCACTGAAGGG
PDK4	CATACACGATGTGAATTGGT
PDK4	ACATGAACCGTATTTCTACT
PDK4	TATTTACTAATTGGGTCGGG
PDK4	GTAGTCCCTACAATGGCACA
PDK4	GGTGGGCGTCAAGATGAAGG
PDPK1	CAAGTTTGGGAAAATCCTTG
PDPK1	CCCGCTCTCTGGTTACATAG
PDPK1	TCTGCTTTAGAGTACTTGCA
PDPK1	GTTCTTCGAGTCCGTCACGT
PDPK1	TTATGCATCCAAGAGCCCAA
PDXK	TCCCCCTAAAGGTTATACGA
PDXK	GAGCTCCAGGAGTTGTACGA
PDXK	CTGTCTTCCAGGTTACTGAG
PDXK	TTTCTTTGTAGACGGGAAGG
PDXK	ATACAGAGCCACGTCATCCG
PF4	CTGGCGAAGGCGACCACAAG

PF4	GGGGCGTGAGGCGCAGAACC
PF4	ACCCCAGGAACAGCAGCCCG
PF4	CTGGCTTCTGCTCTCACCGC
PF4	TGAGGCGCAGAACCCGGCTG
PGF	CGTGTCCGAGTACCCCAGCG
PGF	CCAGCGCCCGGCAGTAGCTG
PGF	TGGGAACGGCTCGTCAGAGG
PGF	GCTCCTAAAGATCCGTTCTG
PGF	ACATGTGCTCCACCTCGCTG
PGGT1B	ATGGATTGAACGGAATACCC
PGGT1B	TATGTTGGATTCCTTAGATG
PGGT1B	TAAGCATGAGAGGCCAGTGT
PGGT1B	AAATAAAGAAGCTTGCTTAG
PGGT1B	CCTCCCATCTTCCAGCTGAA
PGK1	TTCTTCATACCCGCCCAGGA
PGK1	AAAAACCCACCAGCCTTCTG
PGK1	TAAGGTGCTCAACAACATGG
PGK1	GCTCATAAGGACTACCGACT
PGK1	GTAGAACTCAAATCTCTGCT
PGR	AGACGAAAGTTACGACGGCG
PGR	CCCCTCCGACGAAAAGACGC
PGR	GGAGGACGCAGACGAGACTG
PGR	GAAGATTTGTTTAATCTGTG
PGR	GTTGCTCTCCCACAGCCAGT
PHEX	ACATGAAAACCGAACCAGCG
PHEX	GAAGCCAGAATGCATCGAAG
PHEX	TAGATTCAAGCACGGGCCAG
PHEX	CTTTCCAACAACATAAGGGA
PHEX	GTAGTCTTCCCTCACGGCCA
PHF12	GAATCACATCGAACATGTGG
PHF12	GGCCGCGGGTGCAATGAGAG
PHF12	ACGAGGCAGAAAAGCGCAGT
PHF12	GTCGACATCATTCTGCTCAG
PHF12	TGACACATCCACTCTCCAGG
PHKG1	TCCATGAATGAGGACCACCC
PHKG1	TCCGAGTAATCATCCCACTC
PHKG1	TCAAGGTGACCTTCTCAGTG
PHKG1	AAGGTCATCGACGTCACCGG
PHKG1	GAGCCGGGAGAGAGGCTGCG
PHYH	CAGATACTGCACTCTCCCCG
PHYH	GGATAATAACGTTCTAACCC
PHYH	TTACAATTACCAGAATCTGG
PHYH	TCTCATCGTTTGCGCCTGGA

PHYH	GAGCACATCAGCCGGAACAA
PIK3C2A	AGAAGATGATGAAACACCCG
PIK3C2A	ACTAAACTGTACATCATGCA
PIK3C2A	TTTAGACCTACTATTCAGAG
PIK3C2A	TGTGGAGGTATTAGACCATG
PIK3C2A	GCTCAGATATCTAGCAACAG
PIK3C2B	GAAGCGATATTACTGCCACT
PIK3C2B	TTCCCTTAACCAGCAGACTG
PIK3C2B	GTATCCCAGATAGAAGCTCG
PIK3C2B	TTGGCTGTGAGGGAAAACCG
PIK3C2B	TCAGGTTGACCTCATCCCCG
PIK3C2G	GAAATACCTATTGAAAACCC
PIK3C2G	ACCTAGGTCACTTACAGTAG
PIK3C2G	TACATGGCAGAAGAATATAG
PIK3C2G	AGCCCCATAGGAAAACATCA
PIK3C2G	ACTTACCTGCAGGGTCACCG
PIK3CA	GTTCGAACAGGTATCTACCA
PIK3CA	AACCTCGAACCATAGGATCT
PIK3CA	GGATTTAGCTATTCCCACGC
PIK3CA	GAAGCTGTATAATGCTTGGG
PIK3CA	TTATTAATGTAGCCTCACGG
PIK3CB	AAAAATGCGCAAATTCAGCG
PIK3CB	TGTAGCGTGGGTAAATACGA
PIK3CB	AAAGAGCACTTGGTAATCGG
PIK3CB	TAAAACCATCGTAAGCTCAG
PIK3CB	AGACATCCTTGACATCTGGG
PIK3CD	CAAGATGTGCCAATTCTGCG
PIK3CD	TGTGCGCAGTAACCCCAACA
PIK3CD	CAGCGGCTGCCGGAACACTG
PIK3CD	TGATGGCGAAGGAGCCTACG
PIK3CD	CCTTGGTCCAGAATTCCATG
PIK3CG	ACTTAACCCTCTCACAGCAG
PIK3CG	TGGCGGCGGACTTCTACCAC
PIK3CG	GAGAATACGTCCTCCACATG
PIK3CG	TTGCCTCTACAAAAACTGTG
PIK3CG	GGAGAACTATAAACAGCCCG
PIK3R1	TTTCCTAGATACACCCTCCG
PIK3R1	AGCGTAAGCCAATACTGATG
PIK3R1	CCTACTACTGTAGCCAACAA
PIK3R1	GTGATTATACTCTTACACTA
PIK3R1	ACTGAGCTAGAGATTCATTC
PIK3R4	ACTGGCAAATTCGTAAACAT
PIK3R4	CAAGAACCAGATGACAAACG

PIK3R4	TAAATATTGATATCATTACG
PIK3R4	GCGTATTCTGGTTATACGGA
PIK3R4	TTATCTTCCAGAAGACAACC
PIM1	GGCGAGTCGGAGGACAACTG
PIM1	GTCCAGGAGCCTAATGACGC
PIM1	AAGGACCGGATTTCCGACTG
PIM1	TCTTCGACTTCATCACGGAA
PIM1	AGAAGGACCGGATTTCCGAC
PIM2	ATCCTGATAGACCTACGCCG
PIM2	ATAGCAGTGCGACTTCGAGT
PIM2	CAAGCAGGCGGATCACGCCA
PIM2	TGTCACGATGGACAACTCCA
PIM2	CTACTTGGCCAAAGAAGCAG
PIM3	GCTCCGTGATAAAGTCGAAG
PIM3	GGCTTCGGCACGGTCTACGC
PIM3	GACTGGTTCGAGCGGCCCGA
PIM3	ACGGTCTACACCGACTTCGA
PIM3	GCTGCAGGATCTTCACCGGG
PINK1	CCTCATCGAGGAAAAACAGG
PINK1	GCTGGTCCCAGCGAGCCGAG
PINK1	AGCACTGCAGCCCTTACCAA
PINK1	ACATCATCTTGATGGCCAAG
PINK1	TTACCCAGAAAAGCAAGCCG
PKD2	CCGGGTGTAGTAGTACACAT
PKD2	AAGTTGCAATGATTCCCCAG
PKD2	TCTGGATGTTGTGATCGTTG
PKD2	ATGCCAACTGAGCATACGCT
PKD2	GCATCCGGCAGGCGGCCGCG
PKD2L1	CCACATATTCCGAACCCTCG
PKD2L1	GAAGTCTTCATGCACCACAC
PKD2L1	GAAGAGCTCAGACATCACTT
PKD2L1	CAGGTGGACATACCACTCGC
PKD2L1	TTATATCAAGACCACCCTGA
PKLR	GCACGACCCGGACAATATTG
PKLR	AGCAAAATTGAGAACCACGA
PKLR	GTGACCCAAGTGGAGAACGG
PKLR	GAGTCGCGCAATGTTCATCC
PKLR	ATTGGGGTAGTCCACCCACA
PKM	CAAAATCGAGAATCATGAGG
PKM	CTTCTCTCATGGAACTCATG
PKM	GTTTGCGTCATTCATCCGCA
PKM	GTGGTGAATCAATGTCCAGG
PKM	GCAGAGGTGGAGCTGAAGAA

PKN1	TGAACATCGATGTCGCCACG
PKN1	CCACTTCCGAGTGGAGCACG
PKN1	ACCTCCCAGAGACCATCCCG
PKN1	CGTGGTGCTTCCCGACCCGG
PKN1	GCTTCTCCAGGCCCGCCACG
PKN2	GTAGATATCATACTTTGACG
PKN2	TTGTTGCTAGTAGAACAACG
PKN2	TTTGAAGCTCGATAATACTG
PKN2	GGGATGCGTACATCAACCAC
PKN2	GCAGTTGCTGAGCTGTACCA
PKN3	ATGGAGCCTAGGACTCGACG
PKN3	ACAGCATCGACTGCACGTTG
PKN3	ACCAAGGCCAAGCACCAGCG
PKN3	AGGGCTTCGCAGATGAACCT
PKN3	GCACATGGCCCAAGTGGCGG
PLA1A	GTGGACTACTTCGTCAACGG
PLA1A	GCCCACAGCTAGGATTCGAA
PLA1A	TGGTGTACTCAGGTCCAGCG
PLA1A	ATCTGATCTGTGATCACATG
PLA1A	TCTGATCTGTGATCACATGA
PLA2G1B	GCACGAGTATGAATAGGTGT
PLA2G1B	GGAACTGCCACACGGCCCGA
PLA2G1B	CTTGTCCAGTTCATCCACGG
PLA2G1B	TGATCAAGTGCGTGATCCCG
PLA2G1B	ACTTGTCCAGTTCATCCACG
PLA2G2D	AGAAAGGAGTTACCAGTCCG
PLA2G2D	GGTGTGATTCCAATCCAGGG
PLA2G2D	AGTCCGCAGTGACAGCCGTA
PLA2G2D	ACAGCAGTGGATGTTCCCCT
PLA2G2D	GCAGGTGTGATTCCAATCCA
PLA2G4D	GGTCTCACCTGCAACCACAG
PLA2G4D	CTCAGGGTCCCCGTACAGGT
PLA2G4D	GGAGCTTAGCATCTATGATG
PLA2G4D	TAGCGCCCACAGGTCCACAA
PLA2G4D	ACAGCTGTAGGATCACGTAA
PLAT	CAAACATAATTACTGCCGGT
PLAT	GAGCCAAGGTGTTTCAACGG
PLAT	GCCTTAAAGACGTAGCACCA
PLAT	CCTCCTTTGATGCGAAACTG
PLAT	GGTGCTACGTCTTTAAGGCG
PLAU	GCTTAACTCCAACACGCAAG
PLAU	CCCCCAATAATCTTAAAGCG
PLAU	CCCACCTGCACATAGCACCA

PLAU	TGGCGCTGATCACCCAGCAA
PLAU	ACTGCCCAAAGAAATTCGGA
PLAUR	GGGTTAGACTTGTGCAACCA
PLAUR	GACCAACGGGGATTGCCGTG
PLAUR	GTGACCCACTGGATCCAGGA
PLAUR	CAACACCACCAAATGCAACG
PLAUR	GAAGATCACCAGCCTTACCG
PLCB1	ATATCCAAAAGGAACACGTG
PLCB1	CTTGCACTGGCAAGATACGG
PLCB1	TAAGAAAGTTGGGACTTACG
PLCB1	TTGGCGATACATCTCAACAG
PLCB1	GCTGGCCCAAAACATGTCCA
PLCB2	CCCACCAGTATCCTTACTGG
PLCB2	CCAGTGGCATCAATGCACAG
PLCB2	TCAAGCATGAGTTCATGCGC
PLCB2	GGATTGATGGAGTTAGTACT
PLCB2	AGATGCAGCTCAACTCTGAA
PLCB3	TGCGCCGCGTATCAACAGGG
PLCB3	AGTGAGATAGGTGTTATGCG
PLCB3	TTCGATGTAGTTGACAAGCG
PLCB3	GGGGTGCTCACTTCTTGACG
PLCB3	GCTGGAGGAGAAGCTGATGA
PLCB4	AGTCGCATTTACCCCAAGGG
PLCB4	GAAAATTGGCACCTACGTAG
PLCB4	TAAACGATCTAGGAAGACTG
PLCB4	AGATCTTCTATATCTGTCCG
PLCB4	GCTCCTTCTTGCAAAAAGGA
PLCD1	CCCTCTCGCAGTCTCCATCG
PLCD1	TGGGCTCGTAGCGCTCAATG
PLCD1	TGGAAGTGAAAGTATAGCCG
PLCD1	TGTGGTGTCAGACGAAGACG
PLCD1	ACTTCTCCAGACCCTCCGTG
PLCD3	CCAACAACGACCGTCTAGAG
PLCD3	GCACATCGAGGCGGTCCGCG
PLCD3	ACAGTACCTAACATAGGCCT
PLCD3	GCTGGACTCCCCAAATCCCG
PLCD3	CAGGTCGCAGCCCAAGTCG
PLCD4	CTGAGCGATTGGTTTCAACG
PLCD4	CACTGTCTGAAGGTTCATAG
PLCD4	GGCATTGACTAAACGTGCTG
PLCD4	TGGTGAAGCCCTGCTCCAGG
PLCD4	GCACTCACGGTCTTGCAGCA
PLCE1	CTGGCTGTAGAGTTCTACCG

PLCE1	AATGCTTACAGGATACCACT
PLCE1	TCTTCTTGGGAATTGTACCG
PLCE1	AGCAGCTATCGGACAACCAG
PLCE1	AGAGCAGACTATTTACCGCA
PLCG1	ATAGCGATCAAAGTCCCGTG
PLCG1	TCAAACTCATTACAGCGCAG
PLCG1	AGACCCCTTACGAGAGATCG
PLCG1	CTCAGTGGATCGGAATCGTG
PLCG1	GTTCCTTCTTGACTACCAGG
PLCG2	CCACGCCCACCATTATCGAG
PLCG2	CGTACGGCTTGGACTCGTGG
PLCG2	ACCACGCCTTTGTTACCTCG
PLCG2	AAATAAAAGAAATCCGCCCA
PLCG2	GCTCTTCTCATATTCCGCAA
PLCH2	TTGAAGCGTAGGTACTGCGC
PLCH2	GCTGAAGCAGACGTTTGACG
PLCH2	TGGAGAAGCTTCCCACGACG
PLCH2	CAAGGGCAAGATCCTCGTGA
PLCH2	GCTCCCAGCCAACATCAGCG
PLCL1	AGGATGTCGGTAGATTACAA
PLCL1	TATTAGATAGGTGTTATGAG
PLCL1	AGGAGTCACCCATATCACCG
PLCL1	AGGGCAACCAGAACACACCA
PLCL1	GATGAGGGACCGTCGCAGCG
PLCZ1	TTAGACATCTGACTACCCAG
PLCZ1	TTTAGGAGACAATCAAGACA
PLCZ1	AGCAGTCAATCTCCAAACAA
PLCZ1	TGATACTCTACCATCACCAG
PLCZ1	ACAACGGCATCCTTTCACAA
PLD1	ACAGCTATAGACATGCTCGG
PLD1	GTGAGCCCACAAATAGACGG
PLD1	TAGGAGGCAAAACGTCAGAG
PLD1	CAGTGGTTGAGGGAAATCGT
PLD1	AATCTTCCTGAAACGCCCAG
PLD2	GTAGTTGCGATAGAAAGACA
PLD2	TAAACCTCAGGACTCAACCT
PLD2	GGCACCGAAAGATATACCAG
PLD2	ATGAGATGGCACCTGTCTCG
PLD2	CCACTTGCACCTCAAAGCCA
PLG	TGTGGGAGCCAATTGTTCCG
PLG	AAAGTTCCCAGCGCTTGTTG
PLG	CCAGAGACAAATCCACGGGC
PLG	TGTCCTGTTATGTGTGTGAG

PLG	TGACTATGTGAATACCCAGG
PLK1	AACCAAAGTCGAATATGACG
PLK1	CCTGCCTGACCATTCCACCA
PLK1	AGCCAAGCACAATTTGCCGT
PLK1	CGTTGTCCTCGAAAAAGCCG
PLK1	GTTGGAGCTCTGCCGCCGGA
PLK2	ATCACCACCATTCGCACTCG
PLK2	AGCCATGGAACTAAAAGTTG
PLK2	TATGTTGTCCAAAAACCCAG
PLK2	GTCCTCAACAAACAAGGACA
PLK2	CTTGGAATACTGCAGTAGAA
PLK4	ACTGTGTCAGTGTCGAAGGG
PLK4	CGATTCTGATAACCCCATGG
PLK4	CAGAGGAATAAGCTCTACGA
PLK4	TTCTCATGGTATACTACACC
PLK4	TCATGCACCAGATCATCACA
PLOD1	GGAGCAGATCAATATCACCC
PLOD1	ATGACGTGCTGTTTGCATCG
PLOD1	TGGCTACTATGCCCGTTCCG
PLOD1	CAGCTGCAGTTGAACTACCT
PLOD1	GGTTCTGGAAGATACGGCAG
PLTP	ATCACCATTCCGGACCTGCG
PLTP	CTCTGTCTCCAGAATGCACG
PLTP	AAATCACCAATGCCTCCTTG
PLTP	AAGCCACAGGATCCTTCATG
PLTP	GCTGCTTCAGTGAAGCAGGA
PLXNB2	TTCCACGGCGATATCCAGTG
PLXNB2	CACCTGGCAAAGCTTCGTAG
PLXNB2	GGCAATGGGCCACACGACAA
PLXNB2	AGATGTTGCCTCGTCTAAGG
PLXNB2	GCTGCAGCTTCGCATCCAGC
PMPCB	ATCTTAACTAGATTCGTGTG
PMPCB	ATATAACCACACATTATAAG
PMPCB	TCTTCTAATCCGAGGCGCTG
PMPCB	TCCAGATACAATCTGTCTCA
PMPCB	CCAGAGCCGCCGCCGCGCCG
PMS2	TCACACACGGAGTCACTAGG
PMS2	TCACTGCAGCAGCGAGTATG
PMS2	ACTGGTGTCGATTATACATG
PMS2	CGACTGATGTTTGATCACAA
PMS2	TTACCTTCAACATCCAGCAG
PNLIP	TTCCGTAATTCCTGACCATG
PNLIP	TTTGGAATGAGCCAAGTCGT

PNLIP	GTGGCCAATGACATGCACAT
PNLIP	AAGACGTTGTAAGAGGCACA
PNLIP	GATGCGTCCAATGGTCCCAT
PNLIPRP2	TCCACAAACACGGCGTCAGA
PNLIPRP2	AACTCATCGTAGGAGGCACA
PNLIPRP2	CTGGCCATCGGACATGTGCA
PNLIPRP2	CACGGAACCAGACACCATTG
PNLIPRP2	TGAGGAACTCACCTAGGGA
PNMT	GGCCTGAACCAATGTCGATG
PNMT	GGGGCGCGTAGTTGTTGCGG
PNMT	CACCATGACAGATTTCCTGG
PNMT	CTTACCCCTTGCCCTCAATG
PNMT	GCCAGCGCCCCAGCTCCTGG
PNP	AGTGGTCAGAACCCTCTCAG
PNP	CTGCCTCATAGTCCGGTCGT
PNP	TTGCCAGTACCTGTACTTCG
PNP	TGCTGCATTGGTGACTACCA
PNP	TCAGGAACCCAAACACCAGT
POLA1	CATGACACAACAGCTCACAT
POLA1	CAACAAGAACTCGTTACGCT
POLA1	AAGCACGCAATAAAGACAAG
POLA1	CTCTACACACTTTACCGTGG
POLA1	AGAGGAAGTGAAACAAGAGG
POLB	ATTTGTAACATCTCTTCACG
POLB	TTCTGAAGTGAAGCTGGGAT
POLB	ACAAGTACAATGCTTACAGG
POLB	TGACTCGAGTTAGTGGCATT
POLB	GCCGCAGGAGACTCTCAACG
POLD1	TGTAACCGGTGATCACGTCG
POLD1	CCGGATCTCAAAGTCGACGT
POLD1	GCTCACGGCATTGAGCGTGT
POLD1	GGTTTCGGGCCCGAGCACAT
POLD1	GCAGACAGAGAACCCCTCAT
POMC	GGCGGCCGAATCGGTCCCAG
POMC	GGTGTACCCTAACGGCGCCG
POMC	ACATGGGAGTCTCGGCCGAG
POMC	GGCTTGGCACCATCGCTGCG
POMC	CCAGCAACAGGGCCCCCGAG
PON1	GTGAATGTGCTAATCCCATG
PON1	TCTCCCAGGATTGTAAGTAG
PON1	TCCAAGTGAAGTTCGAGTGG
PON1	GGAGATACTGCCTAATGGAC
PON1	GAGCGCAATCAGCTTCGCCA

PON2	CGGGAATTAAGAATCAGTCG
PON2	AACATACTTGAACTTACACT
PON2	ATCAGCACTTTCATAGACAA
PON2	TACCAATTCCTTTAATCAGG
PON2	TTTGCACCAGATAAGCCTGG
POR	TCGTACAGCACGTTGGTACG
POR	ACATGCCTCGCATCCCGTAG
POR	GTGGTCCCCAGATTCATACC
POR	ACGGATTCTTGGCATCAAAG
POR	GTATGAATCTGGGGACCACG
PPARA	GATTTCGCAATCCATCGGCG
PPARA	TCTGTCGGGATGTCACACAA
PPARA	GACTCCGTAATGATAGCCTG
PPARA	GTGTATGGCTGAGAAGACGC
PPARA	TCTGGCCAAGAGAATCTACG
PPARD	GCTTCATGCGGATCGTACGA
PPARD	GTGCACGCCATACTTGAGAA
PPARD	GCACCACAGTGGAGACCGTG
PPARD	AGGAGCCCCAGAGCTCAATG
PPARD	GAACTCAGTGAGCTCCCGCA
PPARG	CACGACATTCAATTGCCATG
PPARG	AGTGAAGGGCTTGATATCAA
PPARG	TGGCATCTCTGTGTCAACCA
PPARG	ACAGATGTGATCTTAACTGT
PPARG	CTATAGCCATCAGGTTTGGG
PPAT	ACTTCATGGGAAGATAGCTG
PPAT	AGACCAGACAGTATGTTCGA
PPAT	CTGGTATTGTGACTAGTGAT
PPAT	GTGTCTGATATAAATGACAA
PPAT	TGAAGTGTTTCAACAACGAA
PPBP	AGGACAAACTAAGAGAAACT
PPBP	TTGTCTTTATACACATGCAG
PPBP	GCTCTGGCTTCCTCCACCAA
PPBP	ATCCAAAGTTTGGAAGTGAT
PPBP	CTTAGTTTGTCCTTTGGTGG
PPIA	CGTACCTGACACATAAACCC
PPIA	GACAAGGTCCCAAAGACAGC
PPIA	GAACTTCATCCTAAAGCATA
PPIA	CGCCGCCCGCCCGACCTCAA
PPIA	GGCAATGTCGAAGAACACGG
PPIB	TGAAGTCCTTGATTACACGA
PPIB	AAAGACTGTTCCAAAAACAG
PPIB	CAGGGCGGAGACTTCACCAG

PPIB	GGGGCCCAAAGTCACCGTCA
PPIB	TTGCCGCCGCCCTCATCGCG
PPIF	ACTTTACACTGAAGCACGTG
PPIF	TGAAGGAAGGGATCACCCTG
PPIF	GTACACGAGCGGGTTCCCGG
PPIF	TCCAGCACCACGCGGCCGAG
PPIF	CAAAGGCTCCACCTTCCACA
PPP1CC	GAAGCACCACTCAAAATATG
PPP1CC	AGCCTAAGACATCTTTATCG
PPP1CC	AAGCAACTACCTGTTTCTTG
PPP1CC	TTTACCGATAGCAGCCATCG
PPP1CC	ACATCGACAGCATTATCCAA
PPP2CA	AACGCATCACCATTCTTCGA
PPP2CA	AAAAGAATCCAACGTGCAAG
PPP2CA	AATAAAAGTCATACCTCATG
PPP2CA	CTCGCCATCTATAGATACAC
PPP2CA	CTGTCTGTGGAGATGTGCAT
PPP2CB	AGCTGAACGAGAACCAAGTG
PPP2CB	AACTTCCTGTAAACGATCCA
PPP2CB	GAACGCATTACAATATTGAG
PPP2CB	ATGAATGTCTGCGAAAGTAT
PPP2CB	CATCTCCACAGACAGTAACA
PPP3CA	GCCTTTAAGATATCCACACG
PPP3CA	GACCTATGTGTGATATCCTG
PPP3CA	CAGGAACTGTTGGTTCATCA
PPP3CA	TTAGGGGACTATGTTGACAG
PPP3CA	GCATTGAGAATAATAACAGA
PPP3CB	GATGTCAAGCGATGTGTTGG
PPP3CB	TGCGCTTAGAATTATCAATG
PPP3CB	GACCATAACAAGTCACACAT
PPP3CB	AAAAGGTATCGTGTATTAGC
PPP3CB	TGATTTGGATGGGATACCCA
PPP3R1	AGATGGCTATATTTCCAATG
PPP3R1	CAGTGTGAGCACATTTCCAA
PPP3R1	AAAGGATTCTGTTGTAACTC
PPP3R1	TGCTTTCCGTATCTATGACA
PPP3R1	TCTTATTCAGAATTCATTGA
PPP4C	TGAGAGTCGCCAGATCACGC
PPP4C	GATTGTCCGAATCTGATCCA
PPP4C	CGATCAGGATAGCGAACCTG
PPP4C	TGGATGTCGCCGCACACCTG
PPP4C	GATCGCATCACACTGATCCG
PPT1	AAGGATCCTAAATTGCAGCA

PPT1	GAGATCAGATTGATCATGGG
PPT1	GCCAGTATTCGGCTTGCACG
PPT1	TGTTGCAATCCCTTAAGCAT
PPT1	TGCCAGATCACCAACGGCAG
PRDX2	GTGAAGCTGTCGGACTACAA
PRDX2	TGAACGCGATGATCTCGGTG
PRDX2	TGGTCACGTCAGCAAGCAGG
PRDX2	GGCGCCATCAACCACCGCTG
PRDX2	ACACAAAAGTGAAGTCCAGA
PRDX5	GGGCTATATACTCGTCGGTG
PRDX5	GCAGCAAGACGGTACAGTGA
PRDX5	GCCCCACTCGCCAGTCACAA
PRDX5	CCTCACCTTGGAACATCCAG
PRDX5	TCAGCGGGCTATATACTCGT
PREP	CATCGTCAGACAGTATGTTG
PREP	GAATGTTCTTGACGTCATGG
PREP	TCTTTGTATAAACCTCTGAT
PREP	GCAGGAATCCAGTGGCATCG
PREP	GTACACGTCGGGGTACTGAA
PRKAA1	TCTTTACAACAGAAATCACC
PRKAA1	GAAGATTCGGAGCCTTGATG
PRKAA1	ATCACCATGAAAATATCAGA
PRKAA1	CACCAGAAGTAATTTCAGGA
PRKAA1	TGAGGTTCTGAATTTCTCTG
PRKAA2	CGGATCTTCTTAAATAACGT
PRKAA2	CAGGCCTGGGGGAATATGCA
PRKAA2	ATGACGTTAGCATCATAGGA
PRKAA2	GATGATAAGCCACTGCAAGC
PRKAA2	TTGAAGAGATGGAAGCCAGG
PRKAB1	GTGGACGCACGACCCTTCCG
PRKAB1	GTGGCCATAAGACGCCCCGG
PRKAB1	AACGGTGTTTCGATGGACGG
PRKAB1	GGATGGCTACAAAGTTATTG
PRKAB1	GTAAACTTCCCCTCACCAGA
PRKAB2	TTCAGACCAGCGGATAACAG
PRKAB2	GTGGGTTCATGATCCATCAG
PRKAB2	GGACGCTTACCTTGGAGTCA
PRKAB2	CAAAATCAGATTTCTTGACA
PRKAB2	GATCTTGTGCTCCTTCCCCG
PRKACA	TTCATAGATAAGAACCCCCA
PRKACA	TTTGAACGAATCAAGACCCT
PRKACA	GAAGATCCTCGACAAACAGA
PRKACA	AGGAGAACTCGAGTTTGACG

PRKACA	GCCCCATGCCCGTTTCTACG
PRKACB	AGATGAGGTCTAGTGAATGG
PRKACB	GAAGATCTTAGATAAGCAGA
PRKACB	AAGCATACTCCAGTCGAACA
PRKACB	TGTGAAAACATTTCACCCCC
PRKACB	CATGGCATAATACTGTTCAG
PRKCA	GCTCCACACTAAATCCGCAG
PRKCA	CCTTGACCGAGTGAAACTCA
PRKCA	AGGAAGGAAACATGGAACTC
PRKCA	AGGTGGGGCTTCCGTAAGTG
PRKCA	AGATTTCTACAGACAGTCGT
PRKCD	TTCCCAACGATGAACCGCCG
PRKCD	TGCAGAGCGTGGGAAAACAC
PRKCD	ATCTCTCGGGCAGACAACAG
PRKCD	CAGCACCCGCTTCTCAACCA
PRKCD	TTGCACACAGAACAGAAGGT
PRKCDBP	TCCGTGCGCCGCATCCAGAG
PRKCDBP	AGTTGGAGAGAGCTCGGACG
PRKCDBP	TCCCGTGCACGCCGTGACGG
PRKCDBP	GTCCGTCACATGCAGGAGGA
PRKCDBP	AGAGCGGTCAGGGATCATGA
PRKCE	GCACCGCTTGTGGACCACGC
PRKCE	GCCTTGTCATTTGACAACCG
PRKCE	CCACGTTGGTCTCACATCGA
PRKCE	ATGTGATCATCGATCTCTCA
PRKCE	TCTTAAGATCAAAATCTGCG
PRKCH	CAACAAACCCACGTACAACG
PRKCH	AGATCGACCCTAAGACGACA
PRKCH	TATTCGATGTCAAGCGAACG
PRKCH	GCTACTCACCCAACCCTCGA
PRKCH	GCTGCTGGACCCCTATCTGA
PRKCI	TCAGAATCCATCTACCGTAG
PRKCI	TGTCTCGAACCTCATTGCAA
PRKCI	ATCTGCACAGACCGAATATG
PRKCI	AGCAAGAATGCAGCCCAACA
PRKCI	TCAACAGGCAATGAACACCA
PRKCQ	TACTGTACCAGACAAACTCG
PRKCQ	CGATGATGTTGAGTGCACGA
PRKCQ	GCTCCATCAAAAATGAAGCA
PRKCQ	CAGCGAGTAGAGCTCCACGG
PRKCQ	TTTGCTTTGCATCAGCGCCG
PRKCZ	CAGCATTAAAGACGACTCGG
PRKCZ	CCTGCTTCCAGACGACAAGT

PRKCZ	CCAGAATCTATCTACCGCCG
PRKCZ	CCCCACCCCCAGGTTCTACG
PRKCZ	TGTGCCCGTCCGCATCCAGG
PRKD1	AGATCCAGACCCAGACCACG
PRKD1	CATTAATGGTCACTTCGCCA
PRKD1	CAATGGAGCAAGCCATCTCG
PRKD1	TGGTAATTCAGACCACACCC
PRKD1	GCACAGTCATGAAAGAAGGA
PRKD2	TTTCAAACATGACCCCACGT
PRKD2	GGGGCGGCCCGTATACGATG
PRKD2	GCAGTCATTAGGGACGCGGG
PRKD2	AATCGGTGCGACACACGACG
PRKD2	ATACGATGAGGCAGAAGAGG
PRKD3	GAAGTGCAAACACTTGCTTG
PRKD3	TTGCAGTACTGACATATCGT
PRKD3	TGGTAACACTCTCCCGTGTG
PRKD3	ATAAATAGTGATAGTAGTCG
PRKD3	GTAGGGGTCTTGGAACTGAG
PRKG1	TGGAAGGACCTGTACGTCTG
PRKG1	GGGTCCAGGAAAAGTGTTTG
PRKG1	TGTGGATTGTATGTACCCGG
PRKG1	CTTACTCTCTGTCAATCACA
PRKG1	GGAAGTTCACCAAGTCCGAA
PRKG2	GATGTGGTGCATATGCAGGG
PRKG2	TGTCTGCTGAGCCAACAACC
PRKG2	AGAACATTCAACCAAACTGT
PRKG2	CCAGTGTTGCAATAATCTCA
PRKG2	GGCCATTGCTGAACTCACAG
PRLR	CCATGAATGATACAACCGTG
PRLR	TGTCCAGACTACATAACCGG
PRLR	AAGACAGAAAACCCTACCTG
PRLR	AATGGACTGACATTAGATGC
PRLR	TTATTCACTGACTTACCACA
PRNP	AACCGCTACCCACCTCAGGG
PRNP	TCACTGCCGAAATGTATGAT
PRNP	CGGCTTGTTCCACTGACTGT
PRNP	GCCTGTAGTACACTTGGTTG
PRNP	AAGAAGCGCCCGAAGCCTGG
PROC	CCCACAAGGGAACTTCACTG
PROC	AATTGCTCGCTGGACAACGG
PROC	AAGAAGACCAAGTAGATCCG
PROC	GGAGATCTGTGACTTCGAGG
PROC	ATCTTCCCATCAATGAGCCG

PROCR	TCCGCGACCCCTATCACGTG
PROCR	GGGCTGCAGCTGAATGATCG
PROCR	AGCCACTTCGAAGAAGACAT
PROCR	GCGGATGGTCAGAGGAACTG
PROCR	GCTCACAGCCCAGGAAGCAG
PRODH	AGATGACCAGGATGCTACAG
PRODH	CACCTACTTCTACGCCAATG
PRODH	CTAGCACTTACCAGAAACTG
PRODH	GTTGGGGACTACCAAGTGCT
PRODH	ATTGGCGTAGAAGTAGGTGC
PROKR1	GGACAATAGCCAGATACCTA
PROKR1	CATAGAATTCGTGGGCCCCG
PROKR1	GACAATGACGAGGACCGTCT
PROKR1	AGTGCGCAGGTAGTTGACAG
PROKR1	GGCCATCTCTGACTTCCTGG
PROS1	AATCTATCGATCACTCAGCG
PROS1	TTTGAATGTGAATGCCCCGA
PROS1	GGAGGTCTTTGAAAATGACC
PROS1	TTTACTTGCACTTGTAAACC
PROS1	TTACTTGAAGAAACCAAACA
PROSC	TTCAGAGACCATAGCCATCG
PROSC	TGATCGAGGCCTATGGACAT
PROSC	ATGGTCCAGATTAACACCAG
PROSC	AGTCGGGTGCGCATTGCGGG
PROSC	GCGTGCAGCAGGCTGTGGCG
PROZ	AAAATGAATGTCACCCAGAG
PROZ	TGTGCACATGCGGTATGACG
PROZ	TGTCCTGGCAAGAGCCGTTG
PROZ	TGCAGACCAGTGTGCCTGCG
PROZ	AGTTGCTGCCCTCATAGCCG
PRSS12	GACTGAGCTGAATACATACG
PRSS12	TGGATGAAGTACGCTGCACT
PRSS12	AAGGCCCAGTCCAGAAAACG
PRSS12	GTGGATAATGTGAAGTGCAC
PRSS12	GGATCAGTACGACTTCGTGG
PRSS22	GTACATACTCCTTGAAACAG
PRSS22	GAAGGTTCCTATCATCGACT
PRSS22	CAGGTATCCTGGCCGCATTG
PRSS22	TGAGCAGAGAACCTGCGCAG
PRSS22	CCTTCCTTCCAGGAATACAC
PRSS27	ACCCGGGCATACATAGCGTG
PRSS27	GAGAGCAACCCCCTGTACCA
PRSS27	ATGCTGAACCGAATGGTGGG

PRSS27	CAACGGAAGCCACTTCTGCG
PRSS27	CAAGTCAGCATCCAGCGCAA
PRSS3	CCGCCACCCTAAATACAACA
PRSS3	GGACACGCGGGCATTGATGA
PRSS3	ACAACATCAAAGTCCTGGAG
PRSS3	GATGATGACAAGATTGTTGG
PRSS3	GCACAACATCAAAGTCCTGG
PRSS33	CATGTCCAGTCGGATCGTTG
PRSS33	CCGTCCTCGGAGTAGTCCGG
PRSS33	GCGACCGCTACAAGGAGTAA
PRSS33	GGTTGGACGCGAGCGCTCAG
PRSS33	GCTGCCCCCGGACTACTCCG
PRSS36	TGGCAAGTGAGCCTGCACCA
PRSS36	GCCGGCCAACTACAGCCAAG
PRSS36	TGCTGCAGAATGACTCGCGT
PRSS36	GCAGGAAGTAGTGTTCCGGG
PRSS36	CGTCATGAAACAGTGAGCAG
PRSS8	CCAGCTAGACTCCTACTCCG
PRSS8	GGAGCCGCACTTTGTCCAAG
PRSS8	TATGAAGGCGTCCATGTGTG
PRSS8	CTTTCACCATTTGGTACCTG
PRSS8	GCCAACGCCTCCTTCCCCAA
PRTN3	GTTTCTGAACAACTACGACG
PRTN3	CGAGCTGCGGAGATCGTGGG
PRTN3	TGCTCGGAGCCCACAACGTG
PRTN3	CGGCCGCTCACATGTCCCGC
PRTN3	CATGTCCCGCAGGCAGTGCG
PRX	GGAGTTGGTGGAAATTATCG
PRX	GAAGTTCTCGAAGAACACTC
PRX	GGTCAGCGGCATCAACGTAG
PRX	GAAGGAGACTTTGTAAGGCT
PRX	CTGCAGGCTGAGGCTCCTGG
PSAP	ACGTCTTTGCATATGTCGCA
PSAP	GGACACAAGGCTCACTATGT
PSAP	GATTGACAACAACAAGACTG
PSAP	GCTTCTGGAGAGACTCGCAG
PSAP	TGGACTGAAAGAATGCACCA
PSEN1	GCCACGCAGTCCATTCAGGG
PSEN1	TAAAACCTATAACGTTGCTG
PSEN1	ACCTGCCGGGAGTTACCCTG
PSEN1	TGTATTTATACAGAACCACC
PSEN1	TTATCTAATGGACGACCCCA
PSENEN	CTGTGCCGGAAGTACTACCT

PSENEN	CCTGGAGCGAGTGTCCAATG
PSENEN	ACAGAACAGAGCCAAATCAA
PSENEN	TGAGCACTATCACCCAGAAG
PSENEN	CATCTTCTGGTTCTTCCGAG
PSKH1	CATACTTAGCTGTAACACGT
PSKH1	CACCGAGCGTGACGCCACGC
PSKH1	CTGAGTTGGTGTATGGCTTG
PSKH1	AGGTGTTCGAGACACAGGAG
PSKH1	GGTGCTCTACACGTACCACT
PSMB1	ATAATAAGGCCATGACTACG
PSMB1	GTATACAGCTTTGATCCAGT
PSMB1	TCTGATACTCGATTGAGTGA
PSMB1	TTACCCTCCGTTGAAAACGT
PSMB1	GATGGAACCGCACAGAGCCG
PSMB2	GAGGAGGTTCACATGATATG
PSMB2	TTTATAAGATGCGAAATGGT
PSMB2	AAGATATTACTCCTGTGTGT
PSMB2	AGGGCCAGCGCTGTATTACA
PSMB2	AATATTGTCCAGATGAAGGA
PSMB5	TTTGTACTGATACACCATGT
PSMB5	GCTTCATGGAACAACCACCC
PSMB5	CCGCTACCGGTGAACCAGCG
PSMB5	ATCTGTGGCTGGGATAAGAG
PSMB5	TCTACTACGTGGACAGTGAA
PSMD13	GAACCGATGTCACACCAGGA
PSMD13	AGCTCTAAAATTAAACATCG
PSMD13	CATCTTTGTAGTAGGACGCG
PSMD13	ATGAAGAATGATTTCCACGA
PSMD13	GGAGCTCTAAAATTAAACAT
PSMD2	AGCCCACTAGCCGATACTTG
PSMD2	GATGCTCGTGGAACGACTAG
PSMD2	GGAAATCGTCCCCTATAACA
PSMD2	CCTGTGGAATGATAGCAGTA
PSMD2	AAACTTTCGGAACACACCCA
PTDSS1	AAATCTTCGATACGCCACAA
PTDSS1	ACCGTAACTACGGATCAGCA
PTDSS1	AGCACTCGGCAAAATTGGGG
PTDSS1	GGGGTCAAACCATCGAACAT
PTDSS1	CGGCCGGTAGAAGAAGTCAA
PTEN	AGAGCGTGCAGATAATGACA
PTEN	CCAATTCAGGACCCACACGA
PTEN	AGCTGGCAGACCACAAACTG
PTEN	ATTCTTCATACCAGGACCAG

PTEN	AGAGGCCCTAGATTTCTATG
PTGDR	GCACAACGAGTTGTCCAATG
PTGDR	TGTGGAAAAAGGCAACTCGG
PTGDR	GGCGCAGGGTGATGTGCCGT
PTGDR	AGGTAGCGCGCAGAAAGCCA
PTGDR	GTAGAAGAAAGGGTGCCCTA
PTGDR2	GCGATGCCACGTGCAACTCG
PTGDR2	GATCATCGCCTCGAGCCACG
PTGDR2	CAGGCAGACTTTGTGCGCCG
PTGDR2	TTTCTCAACATGTTCGCCAG
PTGDR2	CATCCGCTACATCGACCACG
PTGER1	GGTGCTGCGTCTGTACACTG
PTGER1	CTGGTGTGCAACACGCTCAG
PTGER1	GCCGACGTGTTGGGGACCCA
PTGER1	CAGCGCCAGCAGGTTGGACA
PTGER1	GGGCGAGGCGACCACATGCG
PTGER2	CGTACGAAGCCAGTACCACT
PTGER2	CCAGCACGTGGAACAAGGAG
PTGER2	GCTGGGGAACCTCATAGCAC
PTGER2	GGCGAAGAGCATGAGCATCG
PTGER2	TACTGCCCATAGTCCAGCAG
PTGER3	GTACACGACGATGACGACCG
PTGER3	GGATTGCGGATCGGTGTCCG
PTGER3	ACTAGCTCTTCGCATAACTG
PTGER3	GGCGCTGGCGATGAACAACG
PTGER3	AAGTCCTTCCTGCTGTGCAT
PTGER4	GCCCGCGTACATGTAGGAGT
PTGER4	CAGCGCGCAAAAGAGCACGT
PTGER4	GGCGGCGCCGAAAGTCGCTG
PTGER4	GTTCACAGAAGCAATTCGGA
PTGER4	CAGCCCAGTGACCATCCCGG
PTGFR	GGACATTTGATTGGTCAAAG
PTGFR	ACTATAAAATTCAGGCGTCG
PTGFR	GACTCCAATACACCGCTCAA
PTGFR	GAGATTTAGACAGAAGTCCA
PTGFR	GCAGTGTGATGGCCATTGAG
PTGIR	GCGCGCATAGGCCACGAACA
PTGIR	GAAGGCGAAGGCATCGCACA
PTGIR	CACACCGGCCACGAACATCA
PTGIR	CGCCAGCAGAAGCGCCACCA
PTGIR	CCAGCACCGCGAAGGCCGAG
PTGIS	GGACCCACACTCCTACGACG
PTGIS	CGCCCAACAGCACTGCATGG

PTGIS	TCCAAGGCATACCCCAACCA
PTGIS	CGAGAGTATCCTTTGGCAAG
PTGIS	GATGCTACAGAAGCAGGCAG
PTGS1	ACGAGTGTAATAGCTCACGT
PTGS1	GTGTTGATGCACTACCCCCG
PTGS1	TGGAGCGTCAGTATCAACTG
PTGS1	TGGGGCCGGAATAGCCCGTG
PTGS1	CATCCCGCCCCAGAGCCAGA
PTGS2	GGGCTCTAGTATAATAGGAG
PTGS2	GTGGCATACATCATCAGACC
PTGS2	TCAAGACAGATCATAAGCGA
PTGS2	AGTATAAGTGCGATTGTACC
PTGS2	TCCCACCCATGTCAAAACCG
PTH	ATACAGCTTATGCATAACCT
PTH	GGGAAAACATCTGAACTCGA
PTH	TAAAGTTATGATTGTCATGT
PTH	CCAGCATCTCTGGGAGCTAG
PTH	TCACTCACAGATCTCTTCCT
PTK2	TCTGATGATAAATGACTGCG
PTK2	ATGTGGGAGATACTGATGCA
PTK2	ACTTAAAGCTCAGCTCAGGT
PTK2	AGAGCAAAAGATTTGTACAC
PTK2	GCGAGGTTCCATTCACCAGC
PTK2B	ATGAGGGTATAAAGGACCGG
PTK2B	GCAGTACGCCTCGCTCAGGG
PTK2B	GGTCCTGAATCGTATTCTTG
PTK2B	TTGGTAAAAATAGAGCAGCG
PTK2B	CCATTACAATGAGTTCACAG
PTK6	CGCACCCGACAGGACGTAGT
PTK6	CGTGGAAGACGTCCCCCGCG
PTK6	CTCTCCCAGTCATCCCAATG
PTK6	GCCGACGCACAGCTTCCGAG
PTK6	CGAGGAGCTGAGCTTCCGCG
PTK7	GAGCGTACGACTGTGTACCA
PTK7	GGTAGTAGCGAGGTATGAGG
PTK7	GCTCTGACCATCAGAAAGGG
PTK7	GCAGCCAGTACACATGTACC
PTK7	TCTGCCCCTGGCCAATGCAG
PTOV1	CCATAGGGAGACCGACCAG
PTOV1	GTCCGCTTCAGCTTTGCAG
PTOV1	CCTGGCCTGCCAGCCCCCG
PTOV1	CTGGTTGGCAATGACACGC
PTOV1	GCTCACGGCCAGACCCCCGA

PTP4A1	TGAACAGCAATACAACAACC
PTP4A1	ATTGCGTTGCAGGCCTTGGG
PTP4A1	TTAACCAGTCATCAACAATC
PTP4A1	GTTAAATACTTACAAGAACA
PTP4A1	TGACTTCCACAGGAGCTGGG
PTPN1	GAAGCTTGGCCACTCTACAT
PTPN1	GGCCCTTTGCCTAACACATG
PTPN1	AGGGCCTCCTTACCAGCAAG
PTPN1	AAGGTGCCAAATTCATCATG
PTPN1	GTGTGGGAGCAGAAAAGCAG
PTPN11	CTGACAGCGAATCATAACAT
PTPN11	GATTACTATGACCTGTATGG
PTPN11	TTATAAGAAGAATCCTATGG
PTPN11	AAATGTTACTGACCTTTCAG
PTPN11	GGAGGAACATGACATCGCGG
PTPN12	ATATAGTCTGAATCTTGTGA
PTPN12	GGCCTGCCGAGAATTTGAGA
PTPN12	TTTGTGCCATAGATTATACG
PTPN12	AAGAAGGTCCCTCTCCAAGA
PTPN12	GCCTGCCGAGAATTTGAGAT
PTPN13	TTACTTACAAGAATAGACCG
PTPN13	TCACAATGGAGTGCGCACAT
PTPN13	CAAACCGTTGCAGAGTTGGT
PTPN13	AAGGATCACCACTGGTCACG
PTPN13	GTAAATGACACACTACCAGA
PTPN14	ATTACGATGTACATTGGACC
PTPN14	CATGACTGTCTCATAATCGG
PTPN14	TGTGCTTACCGTGTGAAAGA
PTPN14	CAAGCCAGAGTTACCTTGCG
PTPN14	GGATCTGGTGTACAGCCAAC
PTPN18	TGTGCACCGTGGATTATGTG
PTPN18	GCCACTCACGCTGAACTCGC
PTPN18	TGACCACATGCTCGCCATGG
PTPN18	GCAGTCGGCCAGAGAACGTG
PTPN18	GCCGCAGCCTGGACTCGGCG
PTPN20	GCCCCTTCAGAAGAGACAGG
PTPN20	AGCAGCGTATGATATCATGC
PTPN20	ATAACCAGAGAGATAGAAGG
PTPN20	CGTTCCTCTTGGAAAAAGCA
PTPN20	TGTTCAGTAAACGATTATGA
PTPN21	CATTACGGATCCGACCGTTG
PTPN21	GCACAAACGGAACAGCATCG
PTPN21	TCGCTGCGAAACCTCAACAT

PTPN21	ACCACTCCAAAATAGACGGT
PTPN21	GTACGCCTACAGCAGGCCCG
PTPN22	GGGTTGTAGATAAAGGACCC
PTPN22	ATTCAAAGGTGCCAATAACA
PTPN22	GGATGTACGTTGTTACCAAG
PTPN22	TCCGGGAAATGCGGACACAG
PTPN22	GCATGCATGGAGTATGAAAT
PTPN3	CCTGCTATATCAACATAGCG
PTPN3	GATATGGTGCACAACCACCT
PTPN3	GAACGTATGGTGCTCAACAC
PTPN3	AGAACACACGCATGACCAAG
PTPN3	TATGGAGTAGAACTGCACAG
PTPN4	TACTGATTAGGAGTTCCCGG
PTPN4	TAGTTGTGAGAGACATTCTG
PTPN4	ATGATTGGAGTGATGTCAGG
PTPN4	ATTAGGACAAGATTATCATG
PTPN4	GCAGCAGTATTAGAAGGACA
PTPN5	GCGGGCGGACTCCTCACGTG
PTPN5	AGGAACTCAGAGGGTCGTCA
PTPN5	GCCTTACCAGGGTGGTAACG
PTPN5	TCGGATCCACAAAGTTCATG
PTPN5	GCTCAGAAGCCACCACCTCG
PTPN6	CCAGCCGTACTATGCCACGA
PTPN6	TCACGCACAAGAAACGTCCA
PTPN6	CGGCCCAGTCGCAAGAACCA
PTPN6	CCAGGGTGGACGCTACACAG
PTPN6	GCTCCGATCCCACTAGTGAG
PTPN7	GGTGTGTTCACAGAGCAGAT
PTPN7	CATGCTCACTCAGCTCCGAG
PTPN7	CAGCATGGTCCAAGCCCATG
PTPN7	GTGCCCGGCCTAGACAGACA
PTPN7	GGCAGGCCAGAGAACAGCAG
PTPN9	TCTCATCGAAGGGATCTGGG
PTPN9	ACATGTTGGACTGACTTGTG
PTPN9	CTGAGGATCTCAGAACGAAG
PTPN9	CATCAAACTTCCTTGCCATG
PTPN9	ACAGCCACATTCCAAGACAG
PTPRA	AGAGTCTGAAGAATTGACAG
PTPRA	GAACTTGAGCATGCCGATCG
PTPRA	AAACACGATCACTAGCAGAG
PTPRA	CAGGTTGGTAACCATGACGA
PTPRA	AATTCTTTCCGCTTATCCAA
PTPRC	AGCATTATCCAAAGAGTCCG

PTPRC	GGAAACTTGCTGAACACCCG
PTPRC	TCCAAATGGTAACGTTCATG
PTPRC	TGTGGATTACTTATATAACA
PTPRC	AATAGGCCATCTGCAAGCTG
PTPRF	AGTCCGGAGTATAGTAGACG
PTPRF	GGGGACAAGAAGAACTACCG
PTPRF	TCGCACATACAGGTTCGCAG
PTPRF	CAACACCATAGATTTCGGCA
PTPRF	TGGAGAAACGAGGAGCCACG
PTPRH	TCCAGTGTACTCAACCCCGT
PTPRH	AGCACACACTAACATCACCG
PTPRH	AGACGGAGTAAATAGCTCTG
PTPRH	CCAGGACAGATCTTCATGTG
PTPRH	CCGTGGATAGACTTGAACCC
PTPRJ	TTACTGTTGTGCATCAACCA
PTPRJ	ACTGACAGATGTAATATTAG
PTPRJ	CTATACCTACAAGATACATG
PTPRJ	ATGGGTCCACAGGTCCCACG
PTPRJ	AAGCCTGTGATGTTACACCA
PTPRK	CCTCTACATCCCCTAGACGG
PTPRK	TGTGTAACTCAGTCAGAACG
PTPRK	GATGATCCTAACCAATCCAG
PTPRK	CGTACCTCGCTACCTCTGTG
PTPRK	ATGATCCTAACCAATCCAGA
PTPRM	AGGTCAATAACGGGCCACTG
PTPRM	GCAGGGCACAAACTGCGACG
PTPRM	AGCTGCTAGTAGAGCCAATG
PTPRM	TTCTAAATAGATCCCATGCG
PTPRM	GCAAGAGTAATTCTCCTCCG
PTPRN	TTGTAAGCGTTGGAGAACTG
PTPRN	CAGGTTCGTAACTCAGTGAA
PTPRN	CTCTTCTGAACAGACAATGG
PTPRN	ACTCAAAGGTAGTGTCACCA
PTPRN	GCAGCCCTACCTGTTCCACC
PTPRN2	TGCTTCAGGACGCCTCAGGT
PTPRN2	CAGGTAAGAGTCGAGACCCG
PTPRN2	TGTCCAAAACGTGACCACTG
PTPRN2	CTGATGCAAGGCGTGGACCA
PTPRN2	GGGAGAGTCTGGAGAACAGG
PTPRQ	GTGGCAGCCTCAACCCACGT
PTPRQ	ATGGAGGTGTAACTCTACCA
PTPRQ	CGCAGATACACGGATAGAGT
PTPRQ	GAATTCAAACAGTAACTACA

PTPRQ	CCCCGGCTAGGAAGACTGG
PTPRR	CATTGGACATGAGTAGCTTG
PTPRR	ATGCTGAATTGACAATCTGG
PTPRR	AGCAGCAAATGTAATTGTGG
PTPRR	TTCTCACAAGGTCTCAGCTG
PTPRR	TTGTGTCTATACCAACACCA
PTPRU	AGGGCTCCCCATACCACACG
PTPRU	CCTATCGGAACGTTCACGTG
PTPRU	CCCACCTGATGGGCGCACCG
PTPRU	TGGAATATGACTGGATCCCA
PTPRU	TGGAGAGTAGACTTACCGTG
PTPRZ1	ACAACCCAACCGGTATACAA
PTPRZ1	AATGACTACCGTGTCAGCGG
PTPRZ1	GACATGCCTACTGATAATCC
PTPRZ1	ATGGTATCATAAACGACTCG
PTPRZ1	TCTCTGAGAACATATCCCAA
PTS	TGTGGTGACAGTACATGGAG
PTS	TCCAGATTCTTATGATCAAG
PTS	GCAACAATCCAAATGGCCAT
PTS	GCGCGAGCCACCGATTGTAC
PTS	GGGAAATGCAACAATCCAAA
PXDN	CATACAGAACGTCGTACAGG
PXDN	AGGTTACCACTACAACGACC
PXDN	GTTTGTAGCTACCTCCATCG
PXDN	TGAAAACCTACGCGGAGTCG
PXDN	GGATTTGCTGAAAACCTACG
PYCR1	CGTGCCTGTGGCATACACGG
PYCR1	GAAGTTGACACCCCACAACA
PYCR1	CCGCAGTCCTTACGTAGGCG
PYCR1	TGAAATAGGCGCCGACATTG
PYCR1	ACACATTGTGGTGTCCTGCG
PYGB	CAACGTGGGAGACTACATCG
PYGB	ACTGTTTCACGATCTCCGAG
PYGB	GAAAGATAATAAATGCGCTG
PYGB	TCTCGAAACAGGTTCTCACA
PYGB	TAGATTATCTCCAGGTGCCG
PYGL	CGCGAAGTAGTAGTCGCGGG
PYGL	GTCATTGGGATAGAGGACCC
PYGL	CAAACACAGTTCCTGCACCA
PYGL	AAATGATTTCCAAATGTCGA
PYGL	GCTCAGATATGGAAACCCTT
PYGM	ACTCGTAAAGGACCGCAATG
PYGM	GGTGACGCACATTATCATTG

PYGM	TTTAACCAGAAGATCTCCGG
PYGM	CAGAACCAGCCAGCGCCGAG
PYGM	TAGATGATCTGGAGGTGCCG
RAB11A	CATTTCGAGTAAATCGAGAC
RAB11A	GAGTGATCTACGTCATCTCA
RAB11A	TGTTGCAAACTCTACTCCAA
RAB11A	CATATGAAAATGTAGAGCGA
RAB11A	CCATGGCCTCACCTTTAAAG
RAC1	TCACATCTAGTGGTATCCTG
RAC1	CTGTTTGCGGATAGGATAGG
RAC1	ATTTAAGATACTTACACAGT
RAC1	CTGGGCTTATGGGATACAGC
RAC1	AAATGATGCAGGACTCACAA
RAC2	ACAGCAAGCCAGTGAACCTG
RAC2	ATCCCCAGGAACTCACACGG
RAC2	TTATGAGAACGTCCGCGCCA
RAC2	TCTGTGGATAGGAGAGCGGC
RAC2	ATAAGAGGCTGGGCTGACGA
RAD50	CTAGGAACGTGAGTTAAGCA
RAD50	AAGCGGCGTGATGAAATGCT
RAD50	AAACAGCACAAGTTAGACAC
RAD50	AAAAACTGCCAACCAACTGA
RAD50	TTAAAGCCTTAGAAACACTT
RAD51	CTATAGCTTCCCATTGACCG
RAD51	TGTTTGGAGAATTCCGAACT
RAD51	ATACCTAGATTCTACCATCA
RAD51	TTGGTGGAATTCAGTTGCAG
RAD51	GTTGCAGTGGTGAAACCCAT
RAF1	GACCATGTGGACATTAGGTG
RAF1	AGACTTCTCCACGAACACAA
RAF1	GCCGAACAAGCAAAGAACAG
RAF1	TGTTGCAGTAAAGATCCTAA
RAF1	GCATCAATGGAGCACATACA
RALA	TCTACAGTTCATGTACGATG
RALA	GAAGAAGGTAGTGCTAGATG
RALA	GAGACAACTACTTCCGAAGT
RALA	TCGATATCTTAGATACAGCT
RALA	ATGGCTGCAAATAAGCCCAA
RALB	CGAGATAACTACTTTCGGAG
RALB	ATACATGAACTGAAGCGTCA
RALB	GGGAAACAAGTCTGACCTAG
RALB	TAGATATTCTGGACACCGCT
RALB	TGCGTAGTCCTCTTGCCCAG

RANBP2	CGAAACGAAACAATTTCGCG
RANBP2	ACGGGAATTCTATCGCCCAG
RANBP2	TGGTACTTCAGAGACAAGCA
RANBP2	TGTGAACATCCGGCTAGTGG
RANBP2	GGTGATAGTGAATATCTGGT
RAP1A	TGAACAAACTGAACTGTCTG
RAP1A	TCGAAATCCTGGATACTGCA
RAP1A	GTCCTGTAAGTCGTTAAACG
RAP1A	TTGTATATGAAGAACGGCCA
RAP1A	CTAGTGGTCCTTGGTTCAGG
RAP1B	CTAGTCGTTCTTGGCTCAGG
RAP1B	TTATACATGAAAAATGGACA
RAP1B	GTCTTGTAAATCGTTAAATG
RAP1B	GATAGAAGATTCTTATAGAA
RAP1B	AATCTTGGATACTGCAGGAA
RARA	GTGTAGCTCTCAGAGCACTC
RARA	CTTCAAAGCACTTCTGCAGT
RARA	AGAGTCCACCCAGCATAGGG
RARA	AAGCAAGGCTTGTAGATGCG
RARA	AGATCCTGCGGATCTGCACG
RARB	AAGCAGGGTTTGTACACTCG
RARB	GTGGATTGACCCAAACCGAA
RARB	AAGGCCGTCTGAGAAAGTCA
RARB	GTGTTATTAATAAAGTCACC
RARB	CCAGCTGGGTAAATACACCA
RARG	TGGGCAAGTATACCACGGTG
RARG	GGGCTCAGCATCTCGAAAGG
RARG	AAGCATGGCTTGTAGACCCG
RARG	GCTACAGAAGTGCTTCGAAG
RARG	AGATCGTGGAGTTTGCCAAG
RARRES1	ATCCCAGATGAGTCTCAGAG
RARRES1	CACAGAGCGCTACAACCCAG
RARRES1	GTATGCTGACTATTTCCAAG
RARRES1	GCTCCGCACTCACCCACGCG
RARRES1	GTGCTGGCCGAGGTGCAGGA
RARS	GAAAAGTCAACTATAACCTG
RARS	CATTATTAGCCGCCTACAAG
RARS	GAAAGATTGTATTTGTCCCA
RARS	TAAGAAGAGGTTTGATACTG
RARS	AACCTGCTGCAGCAGCCGCG
RB1	GGTTCTTTGAGCAACATGGG
RB1	TGAACTACTTACGAACTGCT
RB1	AAACAATCAAAGGACCGAGA

RB1	AACATCTAATGGACTTCCAG
RB1	GTTCGAGGTGAACCATTAAT
RBCK1	GGAGACCCTGCACTCCCATG
RBCK1	AGTGCGCCCTGATATGACAG
RBCK1	CCAGCACCGAGTAGCACACG
RBCK1	CGCCTCATACCAGCCCGACG
RBCK1	CCTTCATCAACAAGCCCACG
RBL2	GTTTCTCAATGATACAAGGG
RBL2	TAGAAACTGGAGTCACACAA
RBL2	CAGGCGTCCCTCCATCAGAG
RBL2	CATGAGCGAAAGCTACACGC
RBL2	GTACGTTCTCTGAAATGTGG
RBP1	TGAAGTCGACTGGCATTTCG
RBP1	CCGGAAATGAGCGCCCTCCG
RBP1	GTTGGTCAACGAGAATTTCG
RBP1	CCCCACCGCAGACGTCAATG
RBP1	GCTACAATGGATCCTCCCGC
RBP4	CTGACTTCAAAAGACGGACT
RBP4	AGTCTGTGTCGACGATCCAG
RBP4	AACTTCGACAAGGCTCGCGT
RBP4	GACGTGTGCGCAGACATGGT
RBP4	CTCGGCAGTCGCGCTCCGCG
RDH12	GGTGGTTAATGTGTCCTCGG
RDH12	CCTGCAGAGATGTACTGAAG
RDH12	GATGGCTTTGAAACCCACCT
RDH12	CCATATTCTGATCAACAATG
RDH12	GCCTGCAGAGATGTACTGAA
RDH13	GGCAGCAAAGGACATCCGCG
RDH13	CAGGGACTATGTCACCGGTG
RDH13	GAGGTTGATGATCCGCGAAG
RDH13	TCAACAACGCGGGTGTGATG
RDH13	GGTGATTGAGGGTCTCCCCG
RDH5	TGGGATACGAGTCTCCATCG
RDH5	GTATCATCGGACCCACACCA
RDH5	GGGATCAGTGATATCCAACA
RDH5	GAACACAATGGGTCCCATCG
RDH5	CCTCTGCTGCAGCAAGCCCG
RDH8	GCTTTCGAAGAATCCCTCCA
RDH8	CCGGAGATCAACACAGTCCG
RDH8	CCTCAAACTCGGTGACCACG
RDH8	CAGTGTCTCAGCTGTATCCA
RDH8	TCTGGGGCAGACCCTCACCG
RDX	CTCGTCTGAGAATCAATAAG

RDX	ATGATAGACTCCTACCCCAG
RDX	ATAAAAAAGGAACTGAATTG
RDX	AGTACAACAGATGAAGGCTC
RDX	ATACTTGGCTTGGACAGCAT
REL	ATTGGGTTCGAGACAACAGG
REL	TTCCTTCTCCAATTGAACCG
REL	TAACTGTGTACTTACAACAA
REL	ATGTGACAATCCACTTGAGA
REL	GGTCTATTACCTGGATAGAA
RELA	TCAATGGCTACACAGGACCA
RELA	GCTTCCGCTACAAGTGCGAG
RELA	GGAAGATCTCATCCCCACCG
RELA	ACTACGACCTGAATGCTGTG
RELA	GTTCCTATAGAAGAGCAGCG
RELB	ATTGAGCGGAAGATTCAACT
RELB	GCCTCATATCGGGACCAGCA
RELB	CGGTGCAGTCTTTCCCCACG
RELB	GTACTCGTCGATGATCTCTG
RELB	CCTTGGGGAGAGCAGCACCG
REN	TCACCCTCCGCTATTCAACA
REN	TACCAATTACCGTTTAAAGG
REN	GGTGACACAGATGTTTGGAG
REN	AATTCCCTTCGTAATGCTGG
REN	GTAGTTGGTGAGGATCACGG
RENBP	CATCATACACCCGCCCCTCG
RENBP	TGGACGCAGCAAAAGCAGGT
RENBP	CAGTGAGTGTTTCTACACCA
RENBP	GATGGATCAGATCGTCCACT
RENBP	CCATCCAGAAAGCCACCACG
RET	CGGCACAGCTCGTCGCACAG
RET	CTAGATCGGGAAAGTCTGTG
RET	TGCCGAACTTCACTACATGG
RET	CCCGGTGACCGTGTACGACG
RET	TGACTTCTCTCTGCAGACCG
RFK	CCAACCATAGTAAATACCAG
RFK	AGCCCCGCACCACTTGACCC
RFK	TATCATGCATACCTTCAAAG
RFK	TGGTCTCAGGTAGCCAACAA
RFK	TTACTTCTGCCGGGGTCAAG
RHO	GATCAGCAGAAACATGTAGG
RHO	CCGGCTCATACCGCCCAGGG
RHO	AGTACTGTGGGTACTCGAAG
RHO	CATCCTGCTCAACCTAGCCG

RHO	TGGTGGTCCTGGCCATCGAG
RHOA	CTATGTGGCAGATATCGAGG
RHOA	AAAACACATCAGTATAACAT
RHOA	ACAGAAATGCTTGACTTCTG
RHOA	GCCACTCACCTAAACTATCA
RHOA	CAGCAAGGACCAGTTCCCAG
RHOB	CGGTGGGCACGTACACCTCG
RHOB	GAAGCACATGAGAATGACGT
RHOB	CTTGCCGTCCACCTCAATGT
RHOB	GGGACAGAAGTGCTTCACCT
RHOB	CAGTAAGGACGAGTTCCCCG
RHOC	AGGAAGACTATGATCGACTG
RHOC	TGGGGAATAAGAAGGACCTG
RHOC	CAGCAAGGATCAGTTTCCGG
RHOC	AAAGAAGCTGGTGATCGTTG
RHOC	TGGCTCTGTGGGACACAGCA
RHOJ	GCTCGGACTGTATGACACCG
RHOJ	AAACACATCCGTGTTGGGGT
RHOJ	GCATGCAGTCCTTGAGCTCG
RHOJ	ACGTGCCTTATGTCCTCATA
RHOJ	ATAAAAATGTACCTGTCCCG
RIPK1	TGGAAAAGGCGTGATACACA
RIPK1	GGCACCGCTAAGAAGAATGG
RIPK1	CCATGCGGCTGCCATAAAGA
RIPK1	GGGAAGCGAATCCGGAAGCT
RIPK1	GATGCACGTGCTGAAAGCCG
RIPK2	GAGATCATACGTGCTCGGTG
RIPK2	ACTGCCTACCTGTCGAGCAG
RIPK2	TGTAAATCATGGTCCACAAG
RIPK2	ACAGCTATGCAGTTATCACA
RIPK2	ACACAGTGCCAGAGGCGCCG
RIPK3	CGGGCGCAACATAGGAAGTG
RIPK3	GTTTGTTAACGTAAACCGGA
RIPK3	ACTGCTTCGTACACGAGTGA
RIPK3	TCCCGGCTTAGAAGGACTGA
RIPK3	GTTCCTCGATGGACACCAAG
RLBP1	CGCGCACGGAAGTTCAACGT
RLBP1	CATACTTGTCCCGACTAGAG
RLBP1	CAGCTCACAACCAAGGACCA
RLBP1	GGATGAGCTGAACGAGAGAG
RLBP1	GTTCCTCTTCAGGTACCATG
RND3	AAATGCAAGATAGTTGTGGT
RND3	TCTTACCCGAAGTGTCCCAC

RND3	GACGCCAGTGTCCTATGACC
RND3	TCAGCACAGCATCCGAATCA
RND3	CGCCAAGGACTGCTTCCCCG
RNF111	TAACAGTAGAAATCCTACTG
RNF111	AGATGGCTATGGATCAAGCA
RNF111	TATGAGGATGTCCTAATGCA
RNF111	AACGAACTGCCATGTAAACA
RNF111	ATGAGATCTGGAGTGTCCAA
ROCK1	GCAAAGTCTGTGGCAATGTG
ROCK1	AGTCATACCTGAACAACCCA
ROCK1	TTACATATTATAGCAATCGT
ROCK1	CATGGTACGATGTGATACAG
ROCK1	GTACGATGTGATACAGCGGT
ROCK2	TGTTTAGGGAGGTACGACTT
ROCK2	ACCGGATTATATATCACCTG
ROCK2	AGCTGAACATAAGGCCACAA
ROCK2	TAGTAGGTAAATCCGATGAA
ROCK2	CTGAGGTTCTGAAATCACAA
ROR2	TTGTGGCACAGATCGCGGCG
ROR2	AGAGAATACATACTACACGA
ROR2	GCTGGCAGAACCCATCCTCG
ROR2	GAAACCCACCCCCTAACGTG
ROR2	GGACACTGAGAGCAGAAGCG
RORA	ATAGCTCTGTCTGCGCACCG
RORA	ACCATCTCGAGACATCCCTA
RORA	AGTTGGGGAAGTCTCGCCGT
RORA	GTCTGCCTTACTCCCCTCAG
RORA	GTAATCGACAGTGTTGGCAG
RORC	CAGCTGACCCCTGACCGATG
RORC	GTCGTCTGGGATCCACTACG
RORC	AGGAAGTGACTGGCTACCAG
RORC	TTCGGCTGGTGCGGTCGATG
RORC	AGGTCAGGCGAGGAGCCCAG
ROS1	TACACCCCAGTCTACCGCAG
ROS1	CTGGGCTGGAAAGACATATG
ROS1	TGGTGATGCCATACCATGTG
ROS1	GTGCACACCATACCTCCATG
ROS1	TTAGGGCCTTTACATCTAAG
RPA1	ACATCCGTCCCATTACTACG
RPA1	ACAACAGAGAAGTTGCCAAG
RPA1	GGACGACCATCATTTACCTA
RPA1	GCTCCTTTGATAGCCAACAC
RPA1	ATGAGCAGTCGATAACGCGG

RPA3	GGTTGGAAGAGTAACCGCCA
RPA3	GATGAATTGAGCTAGCATGC
RPA3	TACGGGTTCCATCAACTCGA
RPA3	TGGACATGATGGACTTGCCC
RPA3	TCAGATGGAGAAGGAAAAAA
RPS6KA1	TGATGTAAATCACCCATTCG
RPS6KA1	ACTCACCATCAACACCCCAT
RPS6KA1	AGGGTGGCTTGATCTCACGA
RPS6KA1	CTTGACAGCATACTCCATGT
RPS6KA1	TTTGCAGGTGATGTTCACGG
RPS6KA2	GGGGACCACTCACATCCTTG
RPS6KA2	AGGAGTACGCTCTCTTGTCG
RPS6KA2	GCCTCCCCACTGAGGAACTG
RPS6KA2	AGACATCAGCCATCATGTGA
RPS6KA2	TTACTAGGTCATGTTCACGG
RPS6KA3	CCAGAAGTAGTTAATCGTCG
RPS6KA3	AGCTGATGTGCATTAGCACT
RPS6KA3	GGAACGTGATATCTTGGTAG
RPS6KA3	ATCACACATCATGTAAAGGA
RPS6KA3	TTAATCTCCTCCTCTCCCAT
RPS6KA4	TCTCTTTGACCACAACAACG
RPS6KA4	CGCCACGGGCCCGATCCGAG
RPS6KA4	CCAGCGCCAGTACTTCAAGG
RPS6KA4	CTTGCTACGGATGATTTCGG
RPS6KA4	GCTGCACTACGCTTTCCAGA
RPS6KA5	GGCACCAGATATTGTCAGAG
RPS6KA5	CATGCAACTTCACAATATTG
RPS6KA5	GATTTGAAGGACAAACCCCT
RPS6KA5	TGCCCTCGAACATCTCCACA
RPS6KA5	TACCTTGTGGAGATGTTCGA
RPS6KA6	GTTGATGAGCCAATGGAAGA
RPS6KA6	GCATCGCTTGCAAACAGAGT
RPS6KA6	CCTGAAGTAGTAAATAGGAG
RPS6KA6	GTGCAGATGATCCAAAGCAA
RPS6KA6	GTTCTAGGTTCTGTTTACAG
RPS6KB1	CTCTTAGCCCCCATTCACTG
RPS6KB1	AATGAAAGCATGGACCATGG
RPS6KB1	CTTCGGGTACTTGGTAAAGG
RPS6KB1	AGCAGAACGGAATATTCTGG
RPS6KB1	TTCCCTGTCTCGGAAGTCCG
RPS6KB2	GGCCCGCACTCATACCACTG
RPS6KB2	ACTGCGCACCAGAATCTCAG
RPS6KB2	CAAAGTCGGTCAGTTTGATG

RPS6KB2	CGGTTCTCTGCGGTGAAGGG
RPS6KB2	CGAGCCAGAGCTCAGCCCCG
RRAS	TGTTCCCGACCAACACAACG
RRAS	CACACTGCAGATCTTCGTGT
RRAS	GTCGTAGTCAGACACGAAGT
RRAS	CTTCACTCGCAGTTTCAACG
RRAS	AGCGGTGGCGACATGAGCAG
RRM1	CTTGTACCCCAATTCCAATG
RRM1	CCTACCTAGAAAGTTGTGGG
RRM1	TGGCAAACACTCTCCCATGG
RRM1	GGATCTCTTCATGAAACGAG
RRM1	GCGATGCATGTGATCAAGCG
RXRA	CCTACGTGGAGGCAAACATG
RXRA	AGGACTGCCTGATTGACAAG
RXRA	AGGAAGCCATGTTTCCTGAG
RXRA	CAAGGACCGGAACGAGAATG
RXRA	GCACATCTGCGCCATCTGCG
RXRB	GGACAACAAAGACTGCACAG
RXRB	ACGGCTATGTGCAATCTGCG
RXRB	GCCCTGGCTGGATCCCGCAG
RXRB	GTGGCTTCACATCTTCAGGG
RXRB	TCCCCAAATCCCCTTCCCCA
RXRG	TGTGTTTAACCAGAGATCCG
RXRG	GAGGCAGAATGTGCTACCAG
RXRG	TACGCTTGGCCCATTCAACG
RXRG	CTTCAAGAGGACGATAAGGA
RXRG	ATACCCCAGTGAGTGCCCCA
RYR1	CTTCAAACTCGAAGTACCAG
RYR1	GCGTTGTACTCGTTCAGCTG
RYR1	CCCCGTACTTGATCTCAGGG
RYR1	CATCAAGGAGTATCGACGGG
RYR1	GTACCTGGACAGTGTCCACA
RYR2	GGTCTATGCACTCAAGCACG
RYR2	GATTATCAATTACACCACAG
RYR2	GATGGTCCCTCACCAAATAG
RYR2	ATTTGCCGTTAATACAAACA
RYR2	GTACCTGGCTGGTATCCACA
RYR3	TGTTTGGGGAGCATAGTGCG
RYR3	GAGATCAAATCGGAGCAACG
RYR3	AATTCTGTGTCTAACGAGAG
RYR3	CCGCCCGTGCTTATCCAACA
RYR3	CCACCATTCATAAGGAGCAG
S100A1	ACGCCCACTCGGGCAAAGAG

S100A1	GGCGTGGAACACGTTGATGA
S100A1	GGATGTGGATGCTGTGGACA
S100A1	CAAGTACAAGCTGAGCAAGA
S100A1	AATGGGCTCTGAGCTGGAGA
S100A13	TTGCCCCATCTGCTCAAGGT
S100A13	AGAGGAGTCCATTGAGACCG
S100A13	CTTTGAACTCGTTGACGCTG
S100A13	AGAACCACTGACAGAGCTAG
S100A13	CCACCTTCTTCACCTTTGCA
SAA1	GCTGCTGACACCCAGGACCA
SAA1	GGAACTATGATGCTGCCAAA
SAA1	AGACAAATACTTCCATGCTC
SAA1	CGTGATCACTTCTGCAGCCC
SAA1	ACAAATACTTCCATGCTCGG
SAT1	GCTGGCTAAATATGAATACA
SAT1	CCGACTGCAGTGACATACTG
SAT1	ACAATAACTTGCCAATCCAC
SAT1	AAAGAGCACTGGACTCCGGA
SAT1	GCACTTCTGCAACCAGGCAG
SCARB1	TTGTTGATGAGATTCACAAG
SCARB1	TGTTGAAGGACAGGCTACTG
SCARB1	GAGCCACGAAGCGATAGGTG
SCARB1	GAAGGTGCGGTACTCGAGGA
SCARB1	GCGGTACTCGAGGAAGGACA
SCN10A	TGGTTGTAAGGATCAGAGCG
SCN10A	GGATACCACCAAGAGTCCAT
SCN10A	GTGGCAATGGATCTGACTCA
SCN10A	AATCGTGCAAAGGATATGAG
SCN10A	ATAGAGATATACTCACGCCA
SCN11A	AACACACGGAAGGTACGCAG
SCN11A	GGGGGCAACAGTTCCACACG
SCN11A	GATGCGGAATACCACTAGGA
SCN11A	TCCGGACTCTACGAGCACTG
SCN11A	GTAGAGCCCAGTAGTACGCA
SCN1A	AGAACTTGGACTCGCCAATG
SCN1A	GCTGTGGATAGGATGCACAA
SCN1A	ACAGCTTAAAAAGCAACAGG
SCN1A	TGAGGAATAAATGTATACAA
SCN1A	ACTTGCAGCCAATGTCCAGA
SCN2A	TGATATTGGAGCTCCCGCCG
SCN2A	TTTGGGACTGTTGTAAACCA
SCN2A	AGTTCTCCGATCATTCCGGC
SCN2A	GACAGTGAGCATATTTAACT

SCN2A	GCACGAACAGAGAGTCTCTT
SCN3A	TGTACTACATACATACCACA
SCN3A	TCAGCTTGTTCACCTTCTCG
SCN3A	TCGGTGCCATCAAATCATTA
SCN3A	GTCCCAGATCAAGAACACAT
SCN3A	GAGATTCTCTAGTAAAAAGG
SCN4A	CGTGTCATTGCTGTACCACG
SCN4A	CCTGCTGGAAATACTCGTAG
SCN4A	ACCTTCATCGTACTCAACAA
SCN4A	AATCACATCCTGAACCACAT
SCN4A	TCAACGACACCAACACCACG
SCN5A	AATGCTCAAGAAAGAACACG
SCN5A	ATGATGAAAACAGCACAGCG
SCN5A	TGCTTGATGGACATCCACAG
SCN5A	CAAGACCTGCTACCACATCG
SCN5A	GCAAACTTCCTATTACCTCG
SCN7A	GCCAGTTCCATGGATCACCG
SCN7A	GACAAAGTTCTATTAAACCA
SCN7A	TAGCTTAATAGGCAAAACTC
SCN7A	TCTTCACTTGGTCAAATCAG
SCN7A	GTTTCCAGTTCTGTTGTGCA
SCN8A	TGCCAGACAACAGAATAGGG
SCN8A	TGGAAACTAAAAGAGATCCA
SCN8A	GTACCCGTACAGTCAAGTTG
SCN8A	CGATCAAGGCAAAAACACTC
SCN8A	GATTTCAGAAGAGCTCCGAG
SCN9A	TATGACCATGAATAACCCAC
SCN9A	GGAACACCACCCAATGACTG
SCN9A	AGGTAACCTCACCTTAGTGT
SCN9A	GTGTCCGAAGGGATTTAATG
SCN9A	GGAATGTCCCCATAGATGAA
SCNN1A	GGGTGCAGATGGTCACTGCG
SCNN1A	GGGCCGCGGATAGAAGATGT
SCNN1A	GGCCCGTCGAGCCCGTAGCG
SCNN1A	GGAGCGGTGGAACTCGATCA
SCNN1A	TCCATGCCTGGAATCAACAA
SCNN1B	GTCCTGTTGAGGCTACACTG
SCNN1B	CCAGGATCATCTGCTCGCCG
SCNN1B	GGCCCAAGAAGAAAGCCATG
SCNN1B	TTGCAGTATTTCTCCCCACG
SCNN1B	AGCACTGGTGAAGTTCCGGA
SCNN1G	GCACATCGAGTCCAAGCAAG
SCNN1G	ATCTGTTACCTGGAGCGAGT

SCNN1G	GTTCCAAGTCAGCTAGAAGG
SCNN1G	GGCCGCGGGACACCACGATG
SCNN1G	ATAGAAGGAGAAGACGAGGA
SCTR	TGTAGCACGTCACATAGTCG
SCTR	GCTGAAAGTCATGTACACCG
SCTR	CTTGTTCCGAAACTGCACAC
SCTR	CGAATGCCACAAATCCCTGG
SCTR	GTTCTCACCGGAAAGCACAG
SCYL1	CTTGAGGTATATTCCCAACG
SCYL1	AGAACATCTTGACCACCACA
SCYL1	CCCCATCGTAGACCAGACAC
SCYL1	CCCACCAGCTCACAGTAATG
SCYL1	CTATGATGTGAAGCCTGGCG
SDC2	GTTCTGTATATTCAGCGTCG
SDC2	GAAAATGGACCCAGCCGAAG
SDC2	CACCGACTCCGCCGACACGC
SDC2	CAAGATACCTGCTCAGACAA
SDC2	GGGCTTGGTGGCCTGCGTGT
SDHA	ATAAGGTGTGCAATAGCGAG
SDHA	ACCGTGCATTATAACATGGG
SDHA	TGTCATCGCACTGTGCATAG
SDHA	GCCCATCACCTCGACCACGG
SDHA	CGGCACGGCCATGATCACCA
SDHB	TTAAAGCATCCAATACCATG
SDHB	ATGGCAAATTTCTTGATACG
SDHB	TCCTTTATCACATACATGTG
SDHB	TGTGCAATGAACATCAATGG
SDHB	TCACATACATGTGTGGAAGA
SDPR	CTCACAGGTGAACGCAGTCA
SDPR	GTGGGGCAAATCATCATCTG
SDPR	CAAAGATCGTATCTGTAGAG
SDPR	CAGCGACAGATCAGTTTGGA
SDPR	GGTCAGCGCCCACACGCGCG
SDS	GGAAGCAGATCACTTACCCG
SDS	GCCCTAGCGAAGAACAACCC
SDS	GTCCATCTTGAGGTAGACGC
SDS	GCTCTTTCACGATGGAAGCG
SDS	AGACACTGTGGGAAAAGCCG
SEC14L2	TGCTTCTAGATCAACTACGG
SEC14L2	CACCATAATTTATGACTGCG
SEC14L2	GATCCAACAGTATCTGTCAG
SEC14L2	TCTTACGAGTGTCCTCACTC
SEC14L2	ATAGTCATCTGGATTCGGCA

SELE	TGGTCTCTACACATTCACCG
SELE	AATTCATGTAGCCTCGCTCG
SELE	GGAAGCTATGACTTATGATG
SELE	TCCCAGATGAGGTACACTGA
SELE	GCAAAAAGATGAGGACTGCG
SELL	GACTTACCAAACTGACACTG
SELL	AGTTGCCATACAAAACAAGG
SELL	AGATAGGAGGAATATGGACG
SELL	TGACGCCTGCCACAAACTAA
SELL	CCATGGCCACTGCATGACCA
SELP	AAGCACGCATTGTGTTACAC
SELP	AGTAGGGTAGGACCTTATTG
SELP	GTCACAGATGAATTGACATG
SELP	ATAGTTCGGTGTGATAACTT
SELP	GGAGCAGGTGTAGTTCCCGA
SERPINA1	GTGCTGCTGATGAAATACCT
SERPINA1	GAAACAGATCAACGATTACG
SERPINA1	GGCTGTAGCGATGCTCACTG
SERPINA1	CGAGGAAGAGGACTTCCACG
SERPINA1	GAGCCTCCGGAATCTCCGTG
SERPINA10	AAGATCTCCATGAGGCACGA
SERPINA10	TACATTAACAAAGAGACTCG
SERPINA10	GGTGAAGACAGGGTCAAATG
SERPINA10	CCCTCAGAACCAGACCAGCA
SERPINA10	GCTTGATGCTGGGGGCCACA
SERPINA11	AAGCCAAATCCTGGTCATAG
SERPINA11	CAGATTCTGTTACAACTGGG
SERPINA11	CCCAAACTCGAACTAAAAGT
SERPINA11	GGGTCTGGTAGCGACTGAAA
SERPINA11	TGTCCTGGCTGAACTCCGGG
SERPINA12	CTGATCGAGAATATAGACCC
SERPINA12	GTGCCCATGATGTTCCGTAG
SERPINA12	TCTTCCAAAAACTTACGCTG
SERPINA12	GAATTATAAAGCTTTGAGCG
SERPINA12	TTATCAGTCAAAAAACCCAT
SERPINA3	CGTTGGCGGAGGCTAATCCG
SERPINA3	GAGTTGCTCTTTGACAAACA
SERPINA3	GTGGTGGAGCTGAAGTACAC
SERPINA3	CCCAAGATACTCATCAGTCA
SERPINA3	TCTGCTGGACAGGTTCACGG
SERPINA5	TAAGAACCTCGATAGCAATG
SERPINA5	GGTAGTGATACTGATCCTCG
SERPINA5	CAACTTTAGGGACTCTGCAG

SERPINA5	CCTCCATGTAGGTGCCACGG
SERPINA5	ACTCTCTTCTTCATCTCCCG
SERPINA6	AACATGAGTAACCATCACCG
SERPINA6	TCTGAGACTGAGATCCACCA
SERPINA6	ATGTCAAGAATAAGACACAG
SERPINA6	CTGGTGCAGATGAACTACGT
SERPINA6	AGATATAGTTGACCAGGACG
SERPINA7	GCAGGAGATTAACAGTCATG
SERPINA7	GGAACAATACTATCACCTAG
SERPINA7	TCTGTACCGGAGGTTCACTG
SERPINA7	ATAGTTCACTAAGACCATGA
SERPINA7	CCAATGGTAGAGATCCAGCA
SERPINA9	AAGGTTGTAGACATAATCCA
SERPINA9	GAAGAAGATGTTCTGACTCG
SERPINA9	AAGTGCCCTCTTCGTCAAGA
SERPINA9	GGATTACAAGGGAGATGCCG
SERPINA9	GTGCTGCAGATGGATTACAA
SERPINB1	AATGCTGATATCAACAAACG
SERPINB1	TCTGAATGGTGCATTCGTCG
SERPINB1	CAAGGAAGACCATAAACCAG
SERPINB1	GAGATGAAGATGTTTCCAGC
SERPINB1	ATAGCAGATGAAATGCTGAA
SERPINB12	ACCCAAGCGTACCATACCAA
SERPINB12	CTTTGACCATGAAAACACGG
SERPINB12	CAACACTTTCAATCGTCGTG
SERPINB12	CAGAATTGGCTTCATAGAGG
SERPINB12	GGGTCCTTAAACAATGAGAG
SERPINB13	CTTCTGCCCAACGACATCGA
SERPINB13	TGCAGTCAAGATGCCCACAG
SERPINB13	ACTTGCAGGCCAAAATTCTA
SERPINB13	TATTAGTAGCTCTACCAAGC
SERPINB13	ATTGCAGTCAAGATGCCCAC
SERPINB2	TGGGTCAAGACTCAAACCAA
SERPINB2	AAAGTTCTCTGGAGTCATGG
SERPINB2	CATACCGAGTTTACACGGAA
SERPINB2	ATTCTAGGAAGTCTACTGCC
SERPINB2	CTGCAATCAATGCATCCACA
SERPINB4	TATTGGCAATGATACGACAC
SERPINB4	CACTGCACAACAAATTAGCA
SERPINB4	AAAATCAGTAGATTCCACAC
SERPINB4	ACAAATCTGTACAGATGATG
SERPINB4	GAACAGATCGAACATGAACT
SERPINB5	AATGTTTCCCATACAGAACG

SERPINB5	TACGAAGAGACCGTATGCAA
SERPINB5	TCAATTAAGGATCTCACAGA
SERPINB5	CTATGTGAAAAGGAGCCACT
SERPINB5	GTTACTGTTTGAAATCCAAA
SERPINB6	CTTCCGCTCAGGTAAAATTG
SERPINB6	TGTTACCGTTCTCAAGTCAG
SERPINB6	CTGGGATGAACAGTTTGACA
SERPINB6	CTTTCTTTCAATAAAAGTGG
SERPINB6	GTTTGACAAGGAGAACACCG
SERPINB8	ATTTGACAGAAAGTACACAA
SERPINB8	AAAGACGGAGATATTCACCG
SERPINB8	GCCTGGAGCTTACCACGGCG
SERPINB8	GAAGCATATAAATGACTGGG
SERPINB8	TGACAACACGGACCTCGCCG
SERPINB9	CACGTGGGCGAGCTTAAACG
SERPINB9	CGTTTGACGAAACATACACA
SERPINB9	GGCACTGTCTTTAAACACAG
SERPINB9	TGGGTCTCAAAAAAGACCGA
SERPINB9	GTTTGACGAAACATACACAA
SERPINC1	GGGGAGCGGTAAATGCACAT
SERPINC1	CAGTTCCCAGACACGCCGGT
SERPINC1	GGCTTCCGAGGGAATGACAT
SERPINC1	CAAGTTCCGTTATCGGCGCG
SERPINC1	GGATTCATGGGAATGTCCCG
SERPIND1	TGAAAGCGAACTTGGCGTTG
SERPIND1	CACCAACGACTGGATTCCAG
SERPIND1	ACTCACATCAGAGTCTGTCG
SERPIND1	CACATCATGAAGCTCACCAA
SERPIND1	ATCGCAGTAGAAATGCCAAC
SERPINE1	AGGGTGAGAAAACCACGTTG
SERPINE1	CAGACGCGATCTTCGTCCAG
SERPINE1	CCGGAGCACGGTCAAGCAAG
SERPINE1	GCTGAGTTCACCACGCCCGA
SERPINE1	AGACCCTTCACCAAAGACAA
SERPINE2	AGGACCGACGCAATCCCATG
SERPINE2	GTTGTACCATAAATCATTGG
SERPINE2	TGAAATACACTGCGTTGACG
SERPINE2	AGGAACTAGGCTCCAACACG
SERPINE2	GCAGCGTCACAGAGGCCAAG
SERPINF1	CCTTAGGGTCCGACATCATG
SERPINF1	CTTCGGCTATGACCTGTACC
SERPINF1	CCCAGACCCCGACAGCACAG
SERPINF1	GGAGCTCCTTATAGGTACCA

SERPINF1	AATGCAGAGGAGTAGCACCA
SERPINF2	ACAATCCGAACAGCTATTTG
SERPINF2	ACGTTCCATTCAAAGTGGGT
SERPINF2	CTCAGAACCACACGTTGCAG
SERPINF2	TTCGGCCCGCTAGTTAGCTG
SERPINF2	GGTACATCCTGGCAGCCAGT
SERPING1	CTTGGAGAGTCATTCAACAG
SERPING1	GAGCATCCTCTCTTACCCCA
SERPING1	TAAGGGCACCCTTACCACTC
SERPING1	GTTTGCAAGACAGAGGCGAA
SERPING1	GCTGCTGTACAGGGTCCGAG
SERPINH1	CACAAGATGGTGGACAACCG
SERPINH1	CGACACGAGCCCTAGCGACG
SERPINH1	GCTGCAGTCCATCAACGAGT
SERPINH1	CTGAGCTGGGTCCGTACAGT
SERPINH1	CTAGGGCTCGTGTCGCTGGG
SERPINI1	TGTCATATCCCATTGAGTGG
SERPINI1	ATTCCAATGATGTATCAGCA
SERPINI1	CAACTACATCAATAAGTGGG
SERPINI1	ATTCACTGACAAGTCAGCAA
SERPINI1	AGTCAGCAATGGCTTCCTCA
SGPL1	ACAGGATCTATTACACCATG
SGPL1	AGATAGAGGCAGAAATCGTG
SGPL1	CTTTCTCCATAAAGACGATG
SGPL1	GATGCCCATTATTGGTCGTA
SGPL1	ATATCCCCAGACTATCAGCA
SHBG	GCAAACGCCATCCCATCATG
SHBG	CTACTGCGTCACACCCGCCA
SHBG	CCAGGTTCGAACCTCAAAGG
SHBG	GTGATGTAGAATCAAATCCC
SHBG	TGCTGAGGTGGACAGCCGGA
SHMT1	GAACGGGGCGTATCTCATGG
SHMT1	AGGCCCATGATGCGCCCATG
SHMT1	ACCACTCACAAGACCCTGCG
SHMT1	TCTCCCACAGATACTATGGC
SHMT1	CTTGTCTGTCATGAACCCAT
SHMT2	TCATGCGGGCGTAGTCAATG
SHMT2	CTCCCACGGCAGATACTATG
SHMT2	AACCTCACGACCGGATCATG
SHMT2	CTACTCACAAGACTCTTCGA
SHMT2	GCTACATGTCTGACGTCAAG
SI	AATGCGACTATAAGTCCTAG
SI	GTAACATATAGAGTTACCGG

SI	GAACAAAAGTGCACACAACG
SI	ACATATGCAACCTATGAGAG
SI	GGAATAAGAGAGTCATTCCA
SIGMAR1	TGGGTGTTCGTGAATGCGGG
SIGMAR1	CAGCACATACTCGGACAGCG
SIGMAR1	CAGCCAGAGCCAGACGACCT
SIGMAR1	CTCCACGATCAGACGAGAGA
SIGMAR1	GCTCCTCGTCGGGCAGCACG
SIK2	AAAGTTTGATTATGTGAGGG
SIK2	AGAAAATGAGCCATCCATCG
SIK2	TGGAGCGCCTGAAATCACAT
SIK2	ATGAAGGGAGATGTCATGCG
SIK2	GGAAGAGTCGGTCCATCAAA
SIK3	GCACCAGTCAATATCCAGGT
SIK3	TGTGCTTCCATCAAATGGCA
SIK3	GCAGATTGATGAAAGAACCC
SIK3	GCTGCTGAAAGATTGCACTG
SIK3	GTTCAGGTGCAGCATAGGGA
SIRT1	CTCTGAGCCATACCTATCCG
SIRT1	GCGGCGGCGATTGGGTACCG
SIRT1	GTTGACTGTGAAGCTGTACG
SIRT1	ATAGCCTTGTCAGATAAGGA
SIRT1	TCTGGTTTCATGATAGCAAG
SIRT2	TGGATGGAGAGCGAAAGTCG
SIRT2	AGGAGAAGAAACGCGCTGGG
SIRT2	CTGCGCTGCTACACGCAGGT
SIRT2	TCTGGGAGAATAAGTTCCGC
SIRT2	GTACCATCTTCCCTACCCAG
SKP1	ACTATTAAGACCATGTTGGA
SKP1	GAATTCCTGAAAGTTGACCA
SKP1	TGTTGTTGTAGGTCATTCAG
SKP1	CATCATCTTCAGGAGGAGGA
SKP1	GCTGCATTCACATTTGGTAG
SLC10A1	GGGGGACATGAACCTCAGGT
SLC10A1	GTATGGCATCATGCCCCTCA
SLC10A1	AGGACGATCCCTATGGTGCA
SLC10A1	TGAAGAACAACATGAACACC
SLC10A1	TCCTGTACATCTACTCCAGG
SLC12A1	ACTAACGGGTTTGTTCGTGG
SLC12A1	CCTGTTAAGACAATGCACTG
SLC12A1	ATAGAGTACTATCGTAACAC
SLC12A1	ATGTTGTAAATTCCATACGC
SLC12A1	GTTGCTCGTGAATCTCAAGC

SLC12A2	ATTACTACATTGGTTTACGT
SLC12A2	GTTAAGATGTAGCCACGAAG
SLC12A2	TATCATAATAGTAGTGCTGG
SLC12A2	TATGTTACCTACAAAAAACC
SLC12A2	GGTCCGCGATGAGGGCCCCG
SLC12A3	TGGCTACAACACGATCGATG
SLC12A3	ATTCCAATACTACAACAAGT
SLC12A3	TGATGCGGATGTCGTTAATG
SLC12A3	GACCAGCTGTACCCACTGAT
SLC12A3	CAGCAGCGAGAAGAACCCCG
SLC12A4	TCATGATCTCTCGTTCACTG
SLC12A4	GTTCGTGAACCTCGCCTGTG
SLC12A4	GAAGAAACTCCATAGCTGGG
SLC12A4	AGAGCTGGACATCCGCCCAA
SLC12A4	TTACTTACGGAAACACGGGA
SLC12A5	TGGAGAGGATGACACAACCC
SLC12A5	ACGGTGACCACACGGCTATG
SLC12A5	GCAGGCCCCAAACAGAACAA
SLC12A5	CAGTGGTAATATCGAAAGCG
SLC12A5	TCATGCTCCCTACTTCCCTG
SLC12A6	AATGGGGTGATGGTATCCGT
SLC12A6	AATTACACTAATCTGACTCA
SLC12A6	ACAGTCCCATCAAAGTTATG
SLC12A6	AGAGCCTCACAGGTGCACCG
SLC12A6	GTAATACTATACCTAACAGT
SLC12A7	GAGTACGTACGCGCACGCGG
SLC12A7	GGGCTGACAGCGCTTACCCG
SLC12A7	CACAGAAGTCCATCCCCACG
SLC12A7	CCTGGGCACGACGTTTGCAG
SLC12A7	GCGGGGACGAGACTGCCGAG
SLC12A9	ACGGCGACGATCGTGCCCAG
SLC12A9	GTGAAGTGGCCAAACCGGGG
SLC12A9	TCATGATCAGCCGCACACTG
SLC12A9	GGCCAAGGTTGTGTCCCGAG
SLC12A9	CTATGCTGAGGACTACACCA
SLC13A2	GGAGTCCTTAAGATACTCGA
SLC13A2	TGAAGCCCAGGATTAGCCTA
SLC13A2	CTCGGTCTGGATGACGCAGT
SLC13A2	TGGCGGAGTAGCACACGCAC
SLC13A2	GCAGCCCCCCGAAGAACAGG
SLC13A3	TTAAAGCAAAGACCACCCTG
SLC13A3	GATGGTGACAATAGCCACGC
SLC13A3	AGGGCAAGATGCCCATGAAG

SLC13A3	AAATTCACCACGTCACACTG
SLC13A3	ACAGGAACAACAGCATAAGA
SLC15A1	TTGTCCAATTGTGTAGACAA
SLC15A1	GGGCCCACACTAGAAGCGTG
SLC15A1	CCACCAAACGCAGACACACA
SLC15A1	TCTTGTACATCCCACTGCCA
SLC15A1	TCAAAAATAGATTTAGGCAT
SLC15A2	GAAGGCATGGTATATAGATG
SLC15A2	GTTTGCCCTAGGGTTCACGA
SLC15A2	TCCCCAAAGCTATTAGACTC
SLC15A2	AGGTGCAGTTTGGAATAGTG
SLC15A2	GGATGGGAGTAAAATAACAG
SLC16A11	CCACCGCCAAGACTCGACGG
SLC16A11	AGGCCCCGGTCTAAAGCGTG
SLC16A11	GGTGGAGGGTGATCGCGCCG
SLC16A11	GGACAGCCCGTTTATCGCGA
SLC16A11	GCACTTTGACCGAAGCGCCC
SLC16A2	GAAGATCATACCCATCGCGA
SLC16A2	AGCTGGAGTTCGAGTCCGAG
SLC16A2	CCAGGCGGCGTTGAAAGTAG
SLC16A2	TATCCCCCAGCATTCTGATG
SLC16A2	ACGCCTACGGTAGAGACCCG
SLC16A4	TCACAAATAATAGCAACCAG
SLC16A4	TGAAGTATGCTAACTGACTG
SLC16A4	CAAGGACAGTACTACGCAGA
SLC16A4	GTGACTATGGGACTTCTACC
SLC16A4	CCAACCTTACACTAAAACCC
SLC16A5	AAGAAGATGCCGATACACGT
SLC16A5	TCCTGGTGCCATATGCCATG
SLC16A5	GACATCCTGCGGCACAACAC
SLC16A5	GTACTTGCGGTGGCTAGCAA
SLC16A5	GCTGCCATCTGCACGCTCCA
SLC16A6	AGTTCCAGGTTAGTGTGAGT
SLC16A6	TAGATAATAAAAAAGCAGCG
SLC16A6	AAATGTCTTGATGATGCCGT
SLC16A6	CTTACCAGAGATGATGCCGA
SLC16A6	CCCCCAACATCACTACCAGA
SLC16A7	CTGGCTGTTATGTACGCAGG
SLC16A7	TACCACGCTGCTACTAAAGG
SLC16A7	GTGGTTTGATTGGGTCCAAG
SLC16A7	ATGGAGCCAAGAATATAATG
SLC16A7	CTCCTGCTATCACCACCGGC
SLC16A8	CTGAACAGATACGGGACGTG

SLC16A8	TGCTGGGGCTGTACTTCGAG
SLC16A8	GTGAACTACGCCAAGGACGC
SLC16A8	GTACACGGCGAAGGCGCGGT
SLC16A8	GGAAGCTAGGATCATGCCCG
SLC18A1	TTCATCCATGCTATTCCACT
SLC18A1	GAGGAAGAGATTACCCGGGT
SLC18A1	TCTTTGAGAAGCATAAAGAG
SLC18A1	GGCCACAAAGAGTAGAGTAT
SLC18A1	CTTCTTCAACAACAACACCG
SLC18A3	GATCGCCGATAAGTACCCGG
SLC18A3	TACATCGCCCACATGCGCGG
SLC18A3	GCCCAGCACATGAGGCACGA
SLC18A3	CAGCGCGTCAAAGAGCGACA
SLC18A3	CTACATCGCCCACATGCGCG
SLC19A2	GAACAACAGGTTTATAACGG
SLC19A2	GCAAGAACCAGCATTCGCGA
SLC19A2	AGGGCAAATCCTTGTCTCAG
SLC19A2	TCAAGTTGTGAACTACACAC
SLC19A2	GCATTCGCGACGGACCCGAG
SLC1A1	AAATCGTTCAGATCATCATG
SLC1A1	CACCACCAGCACAATACCTG
SLC1A1	TATCAGCGGGAGAATTACAA
SLC1A1	CAATGACAAGTCCAAAGACA
SLC1A1	AGAATTTCTCTAGAGTTGAG
SLC1A4	TGCGGAGCAGCATCTCGCCG
SLC1A4	TGCCCTAGGTACGTACCTGT
SLC1A4	GAGCGCCGAGGCACTCAGTG
SLC1A4	AGCAAATGCTGTCGCAAATG
SLC1A4	GAGTTGCGCAATGAACACCG
SLC1A5	CAGCGCCACACCAAAGACGA
SLC1A5	GTGGTGTGCAGCTTGATCGG
SLC1A5	CGAAAATGCCCCCAGCAAGG
SLC1A5	AACCCCTACCGCTTCCTGTG
SLC1A5	GTACCTGGAAGAGGTCCCAA
SLC22A12	TGAAGCCAAAGGCGAACCTG
SLC22A12	GGCAGGCGTCATGATGAACA
SLC22A12	GAGCATGAGAGTCACACACG
SLC22A12	TGTCCAGTCCCGCACACCGT
SLC22A12	GTACTCACCTGTCTGAGGCA
SLC22A18	TCACCCGTACGCGGAGACCA
SLC22A18	CCCAGGTTCGCAGACCAGCG
SLC22A18	GGCCTTCAGGTCGAACACAC
SLC22A18	GATGGTCATCACGGACCTGT

SLC22A18	GTAGGTAAGCAAGATGACCG
SLC22A25	AGATGAATGAGCTTCCACTG
SLC22A25	TGAAGACATCCTAACCATGG
SLC22A25	AGCGCAGGGAGCAGTATACG
SLC22A25	GCATGAAACTTACCTCACAA
SLC22A25	ATTCGCATACCAGATCCCAC
SLC22A4	GCAACAACAGTGTCCCGCTG
SLC22A4	GGCACCCACCTCGGTCACGA
SLC22A4	ACATACCATTGAAGCCATTG
SLC22A4	CAGAGCAAAGTAACCCACTG
SLC22A4	TATGTCAGTCGTGTTCCTGG
SLC22A5	TCGAACCTGGAATATCCGGA
SLC22A5	GGTCGCTATCAGGAACACGG
SLC22A5	CTGAGAGATGAGCCATCGGG
SLC22A5	AATGTGCTGTTCGTGACCAT
SLC22A5	ACATTCTTCCGGCCAAACCT
SLC22A6	TCAAGATGAGTACCTTCCGG
SLC22A6	GTGGATGCCCATTCACACAC
SLC22A6	CCCATCTACCATCGTGACTG
SLC22A6	AAAGGGCAGTCCCCACTGCG
SLC22A6	GTTCTTGCTGAGGTTGGCAT
SLC22A7	TGGTAGAGGAGAATTCTGAG
SLC22A7	CCTGTCGGACAGATATCCAA
SLC22A7	AAACATTACATAGCTGACGG
SLC22A7	TCTCCCCAACACCACGTTGG
SLC22A7	GTTCATCCTCCAGCTCCCCA
SLC22A8	GGACACCAGAGTCCATACGC
SLC22A8	ACCTGCCATGAAGATAGACT
SLC22A8	ATGATGGCCCGCATCCGGGT
SLC22A8	TATCCGGAACAGGTCACTTG
SLC22A8	CTGCAGCTCACTCAAGATGA
SLC23A1	ACAGGCGTAGTAATCTCCGA
SLC23A1	TTGGCACCCACGGATACGGG
SLC23A1	TGACATGTTGTACAAGATCG
SLC23A1	CAGGCACGAACCGATGCCCG
SLC23A1	GGAGAGATGGAAATGCCCCC
SLC25A1	GCACAATCTCCCTAACCCCG
SLC25A1	GACGGCTGGACAGCACGCGT
SLC25A1	GAACTCGAACATTCCAAACC
SLC25A1	CTGCGTCTTCACGTACTCGG
SLC25A1	CTACGGTTCCATCCCCAAGG
SLC25A12	TCTGATACCACAACTTATAG
SLC25A12	ACCCGAATGCAAAACCAGCG

SLC25A12	TGTGACTTGTCCATAGCGTA
SLC25A12	GGTTATGCCCAAAATGCAGT
SLC25A12	CCTGTCTCTGAAGTTCTGCC
SLC25A13	GGGGCAACTCCCAATAACTG
SLC25A13	AAAAAGACACCTGACATATG
SLC25A13	AATCCGTTCAATGTCTGCTA
SLC25A13	CCAGCCTAATCCAAAGACTG
SLC25A13	GCAGATTTATATGAGCCAAG
SLC25A15	GTCCATGGTAGAACCCCAAG
SLC25A15	ACGTTCCCTGACCTGTACCG
SLC25A15	GTTGGCGATTAGTGCTGGAC
SLC25A15	GTACAGCATGTGTACTGACC
SLC25A15	GGCAGAGGCGAAGGAACCGG
SLC25A16	CGGACCCTAACCATGTCAAG
SLC25A16	ATAAACTGGATTGCACCATA
SLC25A16	ACTCACCTAAATGCTTGTAA
SLC25A16	TAGTTAATTACTACGAAGCT
SLC25A16	CTTTCACCTGGAATGCTAGG
SLC25A20	AGTCTTCCGGAAACAGTCAA
SLC25A20	GGCATCACGGGGCTATATCG
SLC25A20	TCCAGGAGTCATGATTCCTG
SLC25A20	ACTGTGCTTACCCTTATGCG
SLC25A20	GCACCTTGACCGTGTCCAGA
SLC25A22	CGGTCTCTACAAGGGACTCG
SLC25A22	CTGATGTCGGAAGAAGTCGT
SLC25A22	GCAGGTGACACCGATCAGCC
SLC25A22	GTACATGCCGAAGTAGCCCT
SLC25A22	GGCTACTTCGGCATGTACCG
SLC25A23	GCAACTCGTGCACGTCCACG
SLC25A23	GAACTTGATAGCTGACTCGG
SLC25A23	GATAGCGGGAAAATTCCTCC
SLC25A23	CAACCGGCTGAACATCCTTG
SLC25A23	GTATTTCTGGAAGCATTCCA
SLC25A29	ACACCCGTTTGACACGGTCA
SLC25A29	TCACCTATGACGCTCTCACG
SLC25A29	GCGCAGATCTACGGGCACGA
SLC25A29	TGAAGCAGTGCAACGTCCCG
SLC25A29	CGTGCCCGTAGATCTGCGCG
SLC25A31	CGAGGCGCGGTACAAAGGCA
SLC25A31	TGACGCCTCATCCTTCGGGA
SLC25A31	AGCTGTGTCCAAGACAGCGG
SLC25A31	GCAAAGTTTAGAGCTTGTGT
SLC25A31	CGAAAAAGAAGGCAGAAAAG

SLC25A32	GGAGTAACCCCAAATATATG
SLC25A32	TGCGATGTTCCAAACAGCCC
SLC25A32	TAATGGGTTTGTAATGCAGA
SLC25A32	TCCTTTATATTGTCGGTGTG
SLC25A32	TAGTCCATCAAGTTTCCAAA
SLC25A4	GGGGAAGTAACGGATCACGT
SLC25A4	TGGTAACCTGGCGTCCGGTG
SLC25A4	CAAAGGGATCATTGATTGTG
SLC25A4	CCAGACCATGGAACTCACGC
SLC25A4	TCACGCTTGGAGCTTCCTAA
SLC25A5	GGAGTTCTGTCCTTCTGGCG
SLC25A5	AGATACCGAAGTAGGCGGCT
SLC25A5	ACCCGTCTAGCAGCTGATGT
SLC25A5	ACACAGGGATGTGGCCCCTG
SLC25A5	AAGAGGGTACACAAAACACA
SLC29A1	GGCTGTGATAAAGTAGCCGA
SLC29A1	CCAGGATCCGTACGGACTGG
SLC29A1	AAGTTGGACCTCATTAGCAA
SLC29A1	TACACGGCCCCCATCATGAG
SLC29A1	AGAGAGTTCCGCTCAGGCAA
SLC2A1	GACATGGGTCCACCGCTATG
SLC2A1	CCTGCTCATCAACCGCAACG
SLC2A1	TGGCTCCGGTATCGTCAACA
SLC2A1	TTCATCATCGGTGTGTACTG
SLC2A1	CCAGTGCTAAAGAAGCTGCG
SLC2A2	GTGCCACTAGAATAGGCTGT
SLC2A2	CCTTTACATCAAGTTAGATG
SLC2A2	CACCGATATACATAGGAACC
SLC2A2	TAGTTGGAGCTCTCTTGATG
SLC2A2	GCAATTTCACCGATATACAT
SLC2A4	TACCTGAGTAGGCGCCAATG
SLC2A4	GATCAGAATGCCGATAACAA
SLC2A4	AGCACGACCGCAATGATCAG
SLC2A4	GCAGTTTGGGTACAACATTG
SLC2A4	GGATGATGTAGAGGTAGCGG
SLC36A1	AGTATCACCATAATCCACAA
SLC36A1	GCTTTGACCAAAGCGCTGGT
SLC36A1	CCTGCTGATCATAGGCATCG
SLC36A1	AGGATGAGTGGGAACTTCCG
SLC36A1	CTACCACGACTACAGCTCCA
SLC38A2	GTCACGTTATGTGACAAAGC
SLC38A2	GGATATTTGGGATATACCAG
SLC38A2	CCATGGCATAAGAAAGCCCA

SLC38A2	AGTGAAATATGAGTTGCCTT
SLC38A2	GCTGCTGCTGTCTTCATCCG
SLC38A3	ATCACGCTCCAGAACATCGG
SLC38A3	GAGTTGAGCGTGAAGTAGCT
SLC38A3	CCTGCTACTCAAGTCCTCAG
SLC38A3	CCCCCATGGCAGGCAACCAG
SLC38A3	GGAAGGTCTGTATGACAAG
SLC3A1	AGGGCGTCCAGCCCTATGCG
SLC3A1	CCATGTACCAGATCTACCCA
SLC3A1	GGTTTCAATTGAGTCGGACA
SLC3A1	AAACAGTGTCTAGCATGCTG
SLC3A1	TTACGGTTCTGGCTCACAAA
SLC44A1	GTACATGTGGTGGTACCATG
SLC44A1	GTGACAATAGTGTCTTACAC
SLC44A1	AAGGGTTGGAAGACTAGCAG
SLC44A1	ATATATCAAGAGTACTTGTG
SLC44A1	GTAGCTGCACAGACATACCA
SLC46A1	AGAGCTGGACAATGGATCGG
SLC46A1	GAAGGCCACCGAAGTCGCCG
SLC46A1	CACCACAAAAACGGACACTA
SLC46A1	GCTGGAAGCCAGCATCGGGG
SLC46A1	CACTCTGAACTTTATGAAG
SLC4A4	AATTACAGTTGTTCCCGACG
SLC4A4	AGCCTGCTGTAGCCTAACAA
SLC4A4	TCTTCAAACTTGATCCACCT
SLC4A4	ATTAGTCCACCACAGAACCT
SLC4A4	GTGCTGCTCAGAATAGTGAG
SLC5A1	TTGGTGGAAAACATAGCCTG
SLC5A1	GGAAGCGGTTTGGAGGCCAG
SLC5A1	TTGGAAGTGGCCACTTTGTG
SLC5A1	AAGAGCTCATGATTGCCGGA
SLC5A1	GGAAAAATGCTACACTCCAA
SLC5A5	CGGATCGGCCTGTCTCACCG
SLC5A5	TGTCTACAGATGCTGTACAC
SLC5A5	CCATAGCGATAGGCCTCCGA
SLC5A5	CCAGCTTTAACCCTGACCCG
SLC5A5	GCAGACAGCGACAGGCCCAC
SLC5A6	CATGATGCTCTCCTTATACG
SLC5A6	CCTGCATTGAGAGCCAATGA
SLC5A6	AGATCTCTGACGGCACACCC
SLC5A6	CGAACATGACCAGGCCAATG
SLC5A6	GATGCAGGCGGTAGAAAACG
SLC5A7	CAGGATGTGACAATGCAAAG

SLC5A7	CACATCGATGATCACGCTGA
SLC5A7	ACATACCCCTTTGAACGCAT
SLC5A7	GTAGATGTTCCGTGCAAACA
SLC5A7	GCAAATCTATGGAAAACGCA
SLC6A1	GGCGTTCCTGGCATACCCAG
SLC6A1	GACGAATCCTGCGAACATGC
SLC6A1	GGCTGAAAAGTAGACCACCT
SLC6A1	CCTCGGAGTCAGACAGCTTG
SLC6A1	GCAGACTTACCCTTGAACAT
SLC6A11	GCTGATCCTCCTGATACGAG
SLC6A11	TCTGACGGGATCGAGCACAT
SLC6A11	GCGCGTCAAGCGCGACAAGG
SLC6A11	GGCATTCCTGATTCCCTACG
SLC6A11	GCAGCCTTCCCATTGCCCAG
SLC6A12	AAGCATCAGGTACGGAAACG
SLC6A12	TGAGGTAAAATTCTCAAATG
SLC6A12	AAAGTGGCACCTACCTGACT
SLC6A12	CCCAGCACGGTACATACCTG
SLC6A12	TATCTCTGCTACAAAAACGG
SLC6A13	GGTGGTCCTGTTAATTCGAG
SLC6A13	GGCCAGTACACTAGCCAGGG
SLC6A13	CGTCTACTACATCATTGTGT
SLC6A13	CTATCTCTGCTACAAAAATG
SLC6A13	GGGGCACTGGAACAACAAGA
SLC6A14	CTACTCATCCTGTTAGTACG
SLC6A14	AAGGGAGTAAAATATCTGAG
SLC6A14	ATCTATGATTGGATACGCAG
SLC6A14	TCAGTAAAGTGGCGCTCCAA
SLC6A14	GTACGAGGTGCAACTCTGGA
SLC6A15	ATACCTTAGGGGTAAACATG
SLC6A15	AATTACATAAGCCCTAAACT
SLC6A15	AGCCAAGCAAACCATGACCC
SLC6A15	TGGTTCCAAACATACTGCCA
SLC6A15	AGAGAGTTCCAAGAAAAAAA
SLC6A17	GTGGGTCAGAGGATCCGCCG
SLC6A17	CATGACGGACCAGAACGGGG
SLC6A17	CAGCACAGCAAACACCACGA
SLC6A17	TACCTACTTCTGGTACCGAG
SLC6A17	TTAAGGGCATCCAGTCCTCG
SLC6A18	GTGGCCTACCTACTCCACTG
SLC6A18	GTGTACATGTGTGTCATCAG
SLC6A18	AGCGATGGACGCGTACAGGG
SLC6A18	CCGGTACCAGAAGTAGCTCA

SLC6A18	ACTTGGGCCTCTCATCCCCG
SLC6A2	GTAGGGGAAGCGCCACACGT
SLC6A2	GTTCATGGGCCATGTAACCA
SLC6A2	AGCAGCGGGATTCATGACAT
SLC6A2	CAGCACGAAGTAAGGCAGCG
SLC6A2	CTACCGCTTGAAAGAGGCCA
SLC6A20	CCCACGTACCGACACCACTG
SLC6A20	TACCTGTGCCAGATGTACGG
SLC6A20	GGCATTCATCGTCTACACAG
SLC6A20	TAACCACACGGGCTACGATG
SLC6A20	CCGGGGCCATGGAGAAAGCG
SLC6A3	TGGTCCCAAAAGTGTCGTTG
SLC6A3	CACAGCCACCATGCCATACG
SLC6A3	GTAGGGGAACCGCCAGACGT
SLC6A3	TCTGCAGGTTTGGATTGACG
SLC6A3	GCTCCACCCTCACCAACCCG
SLC6A4	AGGCGTCAAGACCTCTGGCA
SLC6A4	TCGGTTACATGGCTGAGATG
SLC6A4	CATTCTGGTAACATATGTAG
SLC6A4	TGCAGATCCACCGGTCTAAG
SLC6A4	AGTCCGGGCAAATATCCAAT
SLC6A5	CACGGACGTGATTCCATCCG
SLC6A5	CTTGCAAACTCAGTAGCCCG
SLC6A5	GAGTACGACATACGGGAACG
SLC6A5	GCCAATAAGACATTTGTCAG
SLC6A5	GGGTTGTTGCAGGAGCCCCA
SLC6A6	AGTGCGGAGCGTTCACCCAG
SLC6A6	CTGCTGGGATGCCTGAACAG
SLC6A6	GAGGGACACAATTACAACGG
SLC6A6	GAACACACCTCACTGCATGG
SLC6A6	ATGATCTCCAAGAAAAACAC
SLC6A7	GGCCTGTGGGTAGACGACAA
SLC6A7	TATCGAGCGTACACCAATGG
SLC6A7	AGTCTCCAAGGACGGCAACG
SLC6A7	CCTTGGAAGACAACAAGTGG
SLC6A7	GGTTCCAGCGGATCTCCCCA
SLC6A8	TGGCGCGTCCAGGTCTCGCG
SLC6A8	GGGCGTGCACATCTCCAAGG
SLC6A8	TCTAGGTGTGGATAGATGCG
SLC6A8	GGCTGCTCACCTTTGAACAG
SLC6A8	CCAGCAGGCCAGCAGACAAA
SLC6A9	CTCCCCCGTTGCGATAGCAG
SLC6A9	TGTACTACCTAACCCCGCAG

SLC6A9	GGCCTCGGGGTAAGCCACGA
SLC6A9	ATGGTGGTGTCCACCTACAT
SLC6A9	CAGTGAGTAGAAGATCTGGG
SLC7A1	GCTGATAGGCAGACCCATCG
SLC7A1	GTGGCTGGCGGACATACCTG
SLC7A1	TAAAAACTGGCAGCTCACGG
SLC7A1	AGTAGGGCATCATGAGCGTG
SLC7A1	GGGACGCCACGATCCCCACG
SLC7A10	TGTACACGAAGGTCACCAGT
SLC7A10	GTAACCATTGATCCCTCCGA
SLC7A10	GCATCTTCATCTCGCCCAAG
SLC7A10	CTACGTCACAGAGATCTTCG
SLC7A10	GGAGCCCTGGAGGAAGGCCA
SLC7A11	AAGGGCGTGCTCCAGAACAC
SLC7A11	ATGGATATACATATTGCAAG
SLC7A11	GAAGAGATTCAAGTATTACG
SLC7A11	TGAGCTTGATCGCAAGTTCA
SLC7A11	CATGTCTCTGACCATCTGGA
SLC7A2	GCCATAGCATAGATTACACG
SLC7A2	AAACGGAACAAGTATCTATG
SLC7A2	GGTCCATGGTGGATAAGCAG
SLC7A2	ATTCAAACGCTACAGGAAGG
SLC7A2	ACATCGGGCAAAGGTCAGCG
SLC7A3	CCCATCGGGATGGAACGCTG
SLC7A3	GTACTTGCTCGGATCCACAC
SLC7A3	ACCTGGTAGAAAGAGCACAG
SLC7A3	AGATATGCCGAACCAGAACG
SLC7A3	AGAATTCCCTCGAAGCCGAA
SLC7A4	CTCCTCGAATACATCATCGG
SLC7A4	CAGGTGGGCTGGCTTCATCG
SLC7A4	TGTGCTCTTGTCCTTCGGTG
SLC7A4	CCAGAGGCACAGACCGCCGT
SLC7A4	ATGACGCCGGAGAAGCCGAA
SLC7A5	ACGTACACCAGCGTCACGAT
SLC7A5	CGACTACGCCTACATGCTGG
SLC7A5	CATCACGCTGCTCAACGGCG
SLC7A5	AGGACAGGCCCACGAAGACG
SLC7A5	GATGCTGGCCGCCAAGAGCG
SLC7A6	TGGGCAACATGATCGGCTCA
SLC7A6	TACCCTGGCACAGTTTAACA
SLC7A6	CAACTAGCAGTGAGACCCAC
SLC7A6	GTCTGGTCAGCAAATGTCTG
SLC7A6	GGGATCTTTGTCTCACCCAA

SLC7A7	TGTATATGAGCACACCCTTG
SLC7A7	GGCATTGTTAGACTTGGCCA
SLC7A7	GAAAGCAAGGAATCCTCCAA
SLC7A7	CAGGATAATGCAACTGACAG
SLC7A7	GCCCAGTTCCGCATAACAAA
SLC7A8	TGGAACAGTTGACCCATGTG
SLC7A8	AATCCCCATGATGATAATCA
SLC7A8	CATACACAAATGTGACCAGT
SLC7A8	CTCACCTACGATGATACCAC
SLC7A8	GGGATTGTACAGATATGCAA
SLC7A9	TGAAGTAGGACACGTTCATG
SLC7A9	TCTCTTTACCCAGCGTCGCG
SLC7A9	GAAGGGCGCACACACATACT
SLC7A9	TTCACTGTCGAGATGAACAC
SLC7A9	CGTCCAGAACATCTTCACCG
SLC9A1	TGACGGAATGATTAACAGGG
SLC9A1	TTTGCCAACTACGAACACGT
SLC9A1	CGGGCTACTTCCTGCCACTG
SLC9A1	TGAGGAACAGGTCACACATG
SLC9A1	AGACAGCCAGAACCGCCACG
SLC9A2	GGGTAATCCAGCGTAAACAC
SLC9A2	TACCCAGGGCTCGCCACATG
SLC9A2	ACATCAGTCTTCATTGCAGG
SLC9A2	AATAACTATTCGACCACTGG
SLC9A2	TGTTACTGCATCATTCAGCA
SLC9A3	GAAGAACACTACGATGATGG
SLC9A3	ACTCACCCATGAGCCCACTG
SLC9A3	CTGTCCGGATATGTCCTCGA
SLC9A3	GCTGAACGACGCAGTCACCG
SLC9A3	GGTGGTGGCCGCGTTCCACA
SLC9A4	GTGCCCGATTCATCGTTGTG
SLC9A4	CTGTGGGCAAGAATCACGAG
SLC9A4	ACATCCAGGTACCTGACCAG
SLC9A4	GACCGGAGGCGATTTGTGGT
SLC9A4	AGTAACAATGAAAAAGTACG
SLC9A5	ACTTGGTCAATCTTGTCCAG
SLC9A5	ACCATGGCTACCACTACTGG
SLC9A5	GAAGGCAAAGACTAAGCCCA
SLC9A5	ATTCCAGAGTGTGCCTACCA
SLC9A5	AGACAGCTAGCACGGCCACG
SLC9A7	TTACACCTACAACAATCTGT
SLC9A7	TTTGGTAAACTTAGTCACGT
SLC9A7	AGCAGTCCCCAAGAAGGCAT

SLC9A7	TGCAGCTGAGTGATTTGTCA
SLC9A7	GCGGTGCTTGAAGAGCCAGA
SLCO1A2	CTAGTAGGCAATATTGTACG
SLCO1A2	GTCAAACAAGCTGCCCACAT
SLCO1A2	CTCGTTGGGTCGGTGCATGG
SLCO1A2	GCCAACAAAAGTCCAATCAA
SLCO1A2	TCTATATAGGAAATACCCAA
SLCO1B3	AACATTCCAGTTGCAACCGT
SLCO1B3	TCTGTTTGCTAAAATGTACG
SLCO1B3	TCAATGTATGAAATCCCCAA
SLCO1B3	ACAGCTAATTTGACCAACCA
SLCO1B3	TATATTGCTAAAGCACTAGG
SLCO2A1	CGATGCCAATCAGACGTGGA
SLCO2A1	AGAAAACAAAGCGCTTCATG
SLCO2A1	ACGTTTAATGAAATCCACCA
SLCO2A1	CTCTGAGAAGTCATCCACAT
SLCO2A1	GGTGACTCACAGATGTACAG
SLCO2B1	GCCCCATAGCCAATCATTCG
SLCO2B1	CATGGATGACAAGCATACCT
SLCO2B1	CGATGCCCACGATGACCGAA
SLCO2B1	AGAAACCCAGCATCTGAGTG
SLCO2B1	TCGATGTAGGAGATGCCAAA
SLIT2	TGAGTGTACAGACCAACCCG
SLIT2	CCCAAGGAAAGCTTTCCGTG
SLIT2	ATAGTTTGAATGGCCCGAAG
SLIT2	TTTAATCCATCAAAGGTGCG
SLIT2	GAGAGGCCATAATGTAGCCG
SMARCA2	ACACCTAGGCTATTCAAATG
SMARCA2	CTCCCAGTCCTACTACACCG
SMARCA2	CCGTGGAACTAAAAGCACTT
SMARCA2	GTCTCCAGCCCTATGTCTGG
SMARCA2	GCTCTTGCAGAATTTCCACG
RPS6	AGTGGTGGGAACGACAAACA
RPS6	TACTTTCTATGAGAAGCGTA
RPS6	ACTGTAGTATCAGTCAGTCC
RPS6	GTCCGCCTGCTACTGAGTAA
RPS6	CAAGGTTTCCCCATGAAGCA
SMO	CAAGAACTACCGATACCGTG
SMO	GATTCTTGATCTCACAGTCA
SMO	CCACATTCGTGGCTGACTGG
SMO	CAAGTGTGAGAATGACCGGG
SMO	CGGAGACTCGGACTCCCAGG
SMOX	CGACCACAATCACGACACTG

SMOX	GCAGGTCTATAACTTGACCC
SMOX	CTTTAGCACACCTAGCGACA
SMOX	CCTCTATTCCAAGAATGGCG
SMOX	GCGCCGATCACCACCACACG
SMPD1	GAACCCAATGTGGCTCGCGT
SMPD1	CTGGTGCCAGACATCATGTG
SMPD1	AATTCATATTGAGAGAGATG
SMPD1	GGGGGAGGGAAGCTATTGAC
SMPD1	GGTGCCAGACATCATGTGCG
SMS	AAATATTCTCATCCTTAGTG
SMS	TTGACCAAATGGTGATTGAT
SMS	TTACCACCCATAGTGCGAGG
SMS	AGTGGCAAAGAAGATTACAC
SMS	GCCTCACTATGGCAGCAGCA
SNAP25	GGGCAATAATCAGGACGGAG
SNAP25	CAACCAGTTGCAGCATACGA
SNAP25	CTGCTCGTGTAGTGGACGAA
SNAP25	GTTATGTTGGATGAACAAGG
SNAP25	GCTGGAGGAGATGCAGCGAA
SNCA	GTAGCCCAGAAGACAGTGGA
SNCA	GCTGCTGAGAAAACCAAACA
SNCA	GAGCAAGTGACAAATGTTGG
SNCA	AGGGTGTTCTCTATGTAGGT
SNCA	GTAGGCTCCAAAACCAAGGA
SNRK	TTATTGCCATAAACTCCATG
SNRK	CGAGGTTACCTACCTACTGC
SNRK	TGTCTTTACGGGTGAAAAGG
SNRK	GGCATTGACGTACTCTACAA
SNRK	GCATGGCAGGATTTAAGCGA
SOAT1	TTTGATATTCCGAAACAAGG
SOAT1	CACCAGGTCCAAACAACGGT
SOAT1	TTAGCTGAATTTAGTACCCG
SOAT1	TCCCTAGAGACACCTAGTAA
SOAT1	GATCCACCAGGTCCAAACAA
SOAT2	CACCCCGACTTACTCAAGCA
SOAT2	GGTCCATTGTACCAAGTCCG
SOAT2	TTCATCAGGAACCTAACCTG
SOAT2	GGATTGTATAGCCTCCCGCA
SOAT2	GCTCGAAGACCAGGACACAA
SOD1	CCTCTATCCAGAAAACACGG
SOD1	AATGTGACTGCTGACAAAGA
SOD1	TATCTCCAAACTCATGAACA
SOD1	GGAAAGTAATGGACCAGTGA

SOD1	AATCCTCTATCCAGAAAACA
SOD2	ACAAACCTCAGCCCTAACGG
SOD2	ATGATCTGCGCGTTGATGTG
SOD2	GACGTTCAGGTTGTTCACGT
SOD2	CCACCATTGAACTTCAGTGC
SOD2	GCACCACAGCAAGCACCACG
SORD	TGATCGAGCCACTTTCTGTG
SORD	CAGAGGTTCCCGTCATCGGG
SORD	TTGTTTAGGGCCAATCGGGA
SORD	TCGGGGAGCACCAGGCTCGA
SORD	GCATGGATCCCCACAGAAAG
SORL1	AGGATGTCTTGTGACAAACT
SORL1	GGACCTCACTACTACACATG
SORL1	TCCTGTAAGTAGAACCCACA
SORL1	GGCTCCGATGAACAGCACTG
SORL1	AAGACAGTGGAGTAGCCAGA
SPAM1	ACAGGAGTAACTGTGAATGG
SPAM1	AAGCAACTTCCATTGTAACC
SPAM1	CTTGATCAGTAAAAACTATG
SPAM1	GAGAAGAGGCTCATATCTAG
SPAM1	TGTACAACTTAGTCTCACAG
SPARC	CCTTCTCAAACTCGCCAATG
SPARC	CAAGGACACTCACATTTGCA
SPARC	AGCCCTGCCTGATGAGACAG
SPARC	GGGAGCTAATCCTGTCCAGG
SPARC	GCCTGATGAGACAGAGGTGG
SPR	GAGGGACGAGATGTTAACCA
SPR	ACTGCTGCTTATCAACAACG
SPR	GCGGGCGCTAAGGACAAGCA
SPR	CACTCAAGTGAACAACTACT
SPR	CCTTAGCGCCCGCAACGACG
SPTLC1	CGAGTTACACGAGCCTTGCG
SPTLC1	AATGTGCCATAAAATCCTCT
SPTLC1	TTCCTGCTTACTCTAAAAGA
SPTLC1	TGATCTTATCAGTGCCAACA
SPTLC1	GATGGTACAGGCGCTTTACG
SQLE	GAAAACAATCAAGTGCAGAG
SQLE	GACCCAGAAGTTATCATCGT
SQLE	CGCTGTCGCCACCGAAACGG
SQLE	TGTGATGGGAGTTCAGTACA
SQLE	TTGAGAACATGATAACCACC
SRC	GACCTGGAACGGTACCACCA
SRC	TCAATGCAGAGAACCCGAGA

SRC	TGTCCTTCAAGAAAGGCGAG
SRC	GTCACGGAGTACATGAGCAA
SRC	CTATGACTATGAGTCTAGGA
SRD5A1	GCCATTGTACACGCCAACAG
SRD5A1	CGTCAGACGAACTCAGTGTA
SRD5A1	GACGTTACCGATGCCCGTAG
SRD5A1	CACTCACCTATTAGAAAACG
SRD5A1	GCAGTGCGCCGTGGGCTGCG
SRD5A2	GTGCTGAATACCCTGATGGG
SRD5A2	CCAGCTATACTCATTCTCAG
SRD5A2	GGAGGGCTTCGCGACGTACA
SRD5A2	CCCAGAAGTACCGTCCCAGG
SRD5A2	ATCCCCGCGGGCACCGCGA
SRMS	CGCTCTATGACTTCACGGCG
SRMS	TTGCAGCTGGTACTTTAGCG
SRMS	ACTCCGAATTCGCCCTTGGG
SRMS	TCACGGAACTCATGCGCAAG
SRMS	GTTGGGGTCCAGGGACCCGG
SRPK1	TGAATGAGCAGTACATTCGG
SRPK1	CTTATGGAACGTGATACAGA
SRPK1	TCATCAAATCCAATTATCAG
SRPK1	AGCCTAACTTTCGGATCACA
SRPK1	TTACCGGTCTCACCATGGAG
SST	AGTCCCTGGCTGCTGCCGCG
SST	GGCTGCGCTGTCCATCGTCC
SST	GCTGTCTGAACCCAACCAGA
SST	GGGACTTCTGCAGAAACTGA
SST	TCTCCTTACCTGCTTCCCCG
SSTR1	CATTGCTAAGATGCGCATGG
SSTR1	CGTGGTCTTCTCTCGCACCG
SSTR1	CAGAACGGGACCTTGAGCGA
SSTR1	GTGGCCAAGGTAGTAAACCT
SSTR1	GATCTCTTTCATCTACTCCG
SSTR2	GGAGCCCACTCGGATTCCAG
SSTR2	CATCGACCGATACCTGGCTG
SSTR2	TGGTCTTCATCTTGGCATAG
SSTR2	AGCCCAGCATATATCATGAT
SSTR2	TCTTCATCTTGGCATAGCGG
SSTR3	TGTAGACGTTGGTGACTGAA
SSTR3	GAGCACGAAGAGCGCCACCA
SSTR3	CGGCACATGAGGGAGCCGAA
SSTR3	TGGCAGGTGCTCATGCCGCG
SSTR3	GGTGGCCCGCACGGTCAGCG

SSTR4	GACACCAGACCGGCTCGCGG
SSTR4	CGCGCGCAGAGGGTGCACCA
SSTR4	GAGCAGGTAGCACAGGCCAA
SSTR4	GCGGCCGACGAGGCCACGAA
SSTR4	CGTCGACGCTGAGCACCGCG
SSTR5	AGGCGGTGACAACAGGACGC
SSTR5	ACGTCCGCGAACACCAGGAG
SSTR5	GAAGGACGCGGCGTTCTGCG
SSTR5	GAGAATGTAGATGTTGGTGA
SSTR5	GCTGGAACGCCTCCTCCCCG
ST14	CACTGATAACCAACACTGAG
ST14	TGACTGCACATGGAACATTG
ST14	TCCGGATACACCGCCCCGTG
ST14	AGCGCATCAGCTCCACACCG
ST14	TGATAACCAACACTGAGCGG
STAT1	TCCCATTACAGGCTCAGTCG
STAT1	AGAACACGAGACCAATGGTG
STAT1	GACGTTGGAGATCACCACAA
STAT1	CCTGATTAATGATGAACTAG
STAT1	ACCCCTGTCTTCAAGACCAG
STAT3	ACGCCGGTCTTGATGACGAG
STAT3	GAGACCGAGGTGTATCACCA
STAT3	AACATGGAAGAATCCAACAA
STAT3	TCGGCCGGTGCTGTACAATG
STAT3	CTACAGTGACAGCTTCCCAA
STAT4	AAATCCAATGCATGTAGCTG
STAT4	AACCCACCCTCAGAGGCCGT
STAT4	TGCAAGACGAATTTGACTAC
STAT4	GCAGCAAATCGCCTGCATCG
STAT4	GGTTATTTCAAACTGTTTAA
STAT5B	TAAGAGGTCAGACCGTCGTG
STAT5B	GTTCATTGTACAATATATGG
STAT5B	CAGCCAGGACAACAATGCGA
STAT5B	GTGGCCTTAATGTTCTCCTG
STAT5B	ATACAAGCTCAGCAGCTCCA
STC1	TTTCCAAAGGATGATTGCTG
STC1	CTTAAAATGCATCGCCAACG
STC1	CGAGTGGCGGCTCAAAACTC
STC1	ACTCCACCTGTGACACAGAT
STC1	GAGCCCCAGGAAATCCCGAG
STK24	AATACTTACTAGATCTAGTG
STK24	ACAGCTATTGAACTTGCAAG
STK24	CTGTAGTTTCCTTCCAACGT

STK24	TGACAATCGGACTCAGAAAG
STK24	GGGCAGGCCCGACTGCACCG
STK3	GGATGTATATCAGCATAAGG
STK3	TGGATTGTTATGGAGTACTG
STK3	ACAGCAACGAGAATTGGAAG
STK3	CATTCAGAAAGCCAGAACTT
STK3	AGTACTCCATAACAATCCAG
STK38	TCCATGAGTAACCACACAA
STK38	AAGAAGTTAGAAAAGGTGA
STK38	GAGCGTGACATTCTAGTGG
STK38	CAGAGATGGGAACTTCTGG
STK38	GCTTGTTCAGAAGAAAGATA
STK38L	GTAAAACATCTTCACCACCC
STK38L	GACTGTAGCCAAGCTCACAT
STK38L	ACTTACAGAAGTCACTTGGT
STK38L	ATAGATGCGATCCACCAGTT
STK38L	TGTCTGGCTTAATATCCCGA
STK39	ATAAAATACATTGTCAACCG
STK39	GGTGTAATAGGTCACTACGT
STK39	AGTGCGTTCCTAGCAACAGG
STK39	AAGAACGTGTAGCAATAAAA
STK39	TCTCCTCCGCAGCATCATGG
STK4	AGCTTTGTATACGCTGCCAT
STK4	CATTCCGAAAACCAGAGCTA
STK4	TGGATCGTTATGGAGTACTG
STK4	TTTAGGATACCATGGCCAAG
STK4	GGATGGATATCAGCATAAGG
STKLD1	TTATCGTTATTCGATCCGAG
STKLD1	CATGCTGTTAGAAGGCAACG
STKLD1	CAAATGGAATATTCGTGCGG
STKLD1	GACCATGGAGCTACATGACA
STKLD1	GATGCTCCAGATCGACCCCT
STYK1	GCCACTTAAGGAGACATCCG
STYK1	GAAAAACAAGTATATCACAT
STYK1	AAAGTTTGAGCTTTACCTTG
STYK1	TCGAGCCAATATGAACACTG
STYK1	CGGCACCTGCAGCTTAGCCA
SV2A	TGAATTGATTGAGATCCAGT
SV2A	GAAGCCATCCTACGGGAGTG
SV2A	GCATCCAGTGATGCTACTGA
SV2A	TGCCTGTCCGACTCCAACAA
SV2A	TCACCTCTAGGAAGAAACGG
SYK	GGTGTACGAGAGCCCCTACG

SYK	CACACCACTACACCATCGAG
SYK	ATCCGAGCCAGAGACAACAA
SYK	TAATAACTCATCTTTAAGAG
SYK	GTGATGTTGCCGAAAAAGAA
TAAR1	GATCACACACAGCATAGTAG
TAAR1	ATGAATGAGCCAATTTGTTG
TAAR1	ATAATTCTGACCACACTCGT
TAAR1	TTTCTGGAGCTAAACTTCAA
TAAR1	TTAAACTGTACAGGGAAGCA
TAAR2	ATATAGTATGATCAGATCGG
TAAR2	GAAGAAACCTGCCATAAACA
TAAR2	CTCTGAGAAGACCACCCCGA
TAAR2	TGTGTGAAGCTGCTTGAAGT
TAAR2	GCATGATGGTGAATCCCAGG
TAAR5	GAATAACGAAGTGTATGCTG
TAAR5	TGGGGCAAGACCCATTCACC
TAAR5	AGCTGGCAACTGCCCACACA
TAAR5	GGCATGCTGATTATCGTGCT
TAAR5	CCTGGTAGCAGAATGCCGCA
TAAR6	CACTATGTACAGAATCACCC
TAAR6	GACCGTTGTAAATCAAAACT
TAAR6	ACAGCACCGCTGTACATGAG
TAAR6	AGTGCAGCTGCTTGAAATGG
TAAR6	GGATTGAAATCATCACCAGG
TAAR8	ATTCTGCCTCTCACGTACAG
TAAR8	CGCCGTGTACAGAATTACCC
TAAR8	GAACTTGGTAGCATAGACCA
TAAR8	AATTATTGTAAGTCAAGGCT
TAAR8	GCAAATTCCCGACACAGACA
TAAR9	ACGGCGTAGAGGATAGATCG
TAAR9	CTTACTGTATATAAACACCA
TAAR9	GGAGAGCTGTTGGTACTTTG
TAAR9	CACAGTAAACTTGGTTGGAT
TAAR9	GATGCCCTTCAGCACAGTG
TAC1	CTGGTCGCTGTCGTACCAGT
TAC1	ACCATAAAGAGCCTTTAACA
TAC1	TTCTTCTGCAAACAGCTGAG
TAC1	CCAGTAATTCAGATCATCAT
TAC1	AAAAGACTGCCAAGGCCACG
TACR1	GGTCAAAATGATGATTGTCG
TACR1	ATGGGCCAGTGAGATCCCCG
TACR1	GATGACACAGATGACCACTT
TACR1	GAGTCGTGTGCATGATCGAA

TACR1	GTAGATACTGGCGAAGACAG
TACR2	TCCACCGTCACCATGGACCA
TACR2	CAGGATGATCCAGATGACGA
TACR2	GGCATAGACAAAGTTGAAGG
TACR2	TCCCCCAGAGGCCTTACAGG
TACR2	GGTGGAGTAGAAGCACTGAG
TACR3	TCTACGCGCTTCATAGCGAG
TACR3	ACAAAGCAGAGAGTACGGCC
TACR3	ACTTGTCACAGGTATCTCCT
TACR3	CACCACCACACCATACGCCA
TACR3	GCAGAAACCTGGATAGACGG
TAF1	TATTATTATCCCAAGCAACA
TAF1	TCTGGTATATGGACGCTGGG
TAF1	CATACGGACTACAAAAGATG
TAF1	GACCAGGATTCTATTACTGG
TAF1	GGAAAAGTAAAGATCGGCCA
TAOK2	TCAAGACAGACCAACCTCAG
TAOK2	CCCAACACCATTCAGTACCG
TAOK2	GCCAGGGTTAGTGAAGCTAG
TAOK2	GCTCTGGGCCCACCCAACAC
TAOK2	GCATTCATGTTAAAGAGCGG
TAOK3	CCTAATACTATTGAGTACAA
TAOK3	GTATTCATAAGGGATGAGGC
TAOK3	TTCATTAGACTGTAACGTTG
TAOK3	AACAGATGTCAGGTTATAAG
TAOK3	GCATTCATGTTGAAAAGGGG
TARS	CCTTTGATAGATCTCTGCCG
TARS	TTATGTTTGATCATCGGCCA
TARS	TAAAGTAAATAATGTTGTGT
TARS	GGCCCGAGGTCATCCAGAGT
TARS	AGAGTGGGAGAAGTTCCAAG
TAS1R2	AGGCCTGCCCGAATAACGAG
TAS1R2	CGTCTACAGCGTGTACTCTG
TAS1R2	CCTGTACCACTTCTTCAATG
TAS1R2	CTCCGAGTCTGTCATGACTG
TAS1R2	GCACAATGATCCAGTTCCAG
TAT	TGGCCAACACAGCTAAACAA
TAT	ACGTGCATGTCAACGTTGGT
TAT	AGGCTTCCTATCCAGTCGGG
TAT	CCCCAACTCACCAATGGACA
TAT	GCACGTCCAGAATTGAGGGG
TBC1D1	GTTACTTAATGTACTAGACA
TBC1D1	GAGGTTCCTGGCATTCGATG

TBC1D1	GCCAGGGAGAAGTCAACAGT
TBC1D1	TGTGCAGCCCACAGATATCG
TBC1D1	TCTTGTGCGCCACCGTCACG
TBK1	TCCACGTTATGATTTAGACG
TBK1	ACAGTGTATAAACTCCCACA
TBK1	AATCAAGAACTTATCTACGA
TBK1	AGTTGATCTTTGGAGCATTG
TBK1	GCAGTGATCCAGTAGCTGCA
TBXA2R	GCTACACCGTGCAATACCCG
TBXA2R	GATACCCAGGTAGCGCTCTG
TBXA2R	GCTGGTGACCGGTACCATCG
TBXA2R	GCTGACCGTGTTCAGCAGGA
TBXA2R	GCTCTTCGAGTGGCACGCCG
TBXAS1	GGGACGCATTTGACATCCAG
TBXAS1	CGACGACCAAGATAGTACCT
TBXAS1	GTAAAACCAGGATAGGTCTG
TBXAS1	AAAACTACGTACCATTCTGT
TBXAS1	CTGCACTTACCATTTCAGGA
TCN2	TCGGAGACAACGGATCACCA
TCN2	GAAGCGGGTCCATGACAGCG
TCN2	CTCTCAGGGCATGATCACAA
TCN2	GCCAACTGTGAGTTTGTCAG
TCN2	TCCTAGGTCTGCCTTCAGCG
TEC	AAACACTTACTTCACTGCGG
TEC	CATGATCTCAGATTAGAGAG
TEC	TCTTCCCTTTCACACTAACT
TEC	GGAAGGTGCAATGTGCGAGG
TEC	CTACTATGAGGGTCGAGCAG
TEK	TGGCACAGGAACACCCATAG
TEK	AGACCACTCTAAATTTGACC
TEK	GCCTGAAACAGCATACCAGG
TEK	TACTCGGCCAGGTATATAGG
TEK	GATCATATAGAAGTAAACAG
TERT	CACACGCTAGTGGACCCCGA
TERT	GTGACACCACACAGAAACCA
TERT	CTCACGCAGACGGTGCTCTG
TERT	GGCCCGCACACGCAGCACGA
TERT	GCTGCGCAGCCACTACCGCG
TF	GCCATCCGGAATCTACGGGA
TF	GTCGTGGCCCGAAGTATGGG
TF	CGGACCTGCCTAGACCCGTG
TF	TATAGTCGAGTGCTTGACAA
TF	TCTCTTTCAGGCAAACGAAG

TFPI	TGTGAACGTTTCAAGTATGG
TFPI	TATGTCGAGGTTATATTACC
TFPI	GAAGAACACACAATTATCAC
TFPI	CTTGGTTGATTGCGGAGTCA
TFPI	CTCAGAATCAGCATTAAGAG
TG	CAAGACGCAATATCACCTGG
TG	CTGCAGTATCCGGTACAGGG
TG	GGCCTGGTCACATTGCACTG
TG	GATTCTACCAAGAACAGGCA
TG	GCTGGTGTGTGGACGCCCAG
TGFB1	GGTTTCCACCATTAGCACGC
TGFB1	TTGATGTCACCGGAGTTGTG
TGFB1	GGTGAAGCGGAAGCGCATCG
TGFB1	GAATGGTGGCCAGGTCACCT
TGFB1	CTAATGGTGGAAACCCACAA
TGFB2	CGACGAAGAGTACTACGCCA
TGFB2	AGATGGAAATCACCTCCGGG
TGFB2	AGAAAACTATAAAGTCCACT
TGFB2	TGGACCAGTTCATGCGCAAG
TGFB2	GCTTGCTCAGGATCTGCCCG
TGFB3	ATAAATTCGACATGATCCAG
TGFB3	GGCAAGAATCTGCCCACACG
TGFB3	CAGGACCTGATAGGGGACGT
TGFB3	AAGAGGGTGGAAGCCATTAG
TGFB3	GGCCCTGCTGAACTTTGCCA
TGFBR1	AGAACGTTCGTGGTTCCGTG
TGFBR1	TAAAAGGGCGATCTAATGAA
TGFBR1	GTTGTGTATAACTTTGTCTG
TGFBR1	ATGGGCAAGACCGCTCGCCG
TGFBR1	ATTGTTCTCTGAACAAGCAA
TGFBR2	ACAGTGATCACACTCCATGT
TGFBR2	TATCATGTCGTTATTAACTG
TGFBR2	GCAGAAGCTGAGTTCAACCT
TGFBR2	ACCTACAGGAGTACCTGACG
TGFBR2	GCTTCTGCTGCCGGTTAACG
TGM1	TACTCTAGGAAACAACCCCG
TGM1	GGACGGAATATCCCGTGCGT
TGM1	CATGCTAGTAGTGAACGGTG
TGM1	CCGCACACAATCAGACGCTG
TGM1	GCATATGGAAAGGCTGCCCG
TGM2	CTGGATCCAGTCTACCACGT
TGM2	GAAGGTGAGACTGTCTACAC
TGM2	AAGAGCGAGATGATCTGGTG

TGM2	GGGTGACTGGACAGCCACCG
TGM2	ACGCTGGGACAACAACTACG
TGM3	ACCCATAGGACGGTACACAA
TGM3	GATGTGTACTACGACCCCAT
TGM3	GAACACCAATGACCGAAGCG
TGM3	TCGTCATGGCCGACTCTGAG
TGM3	GCCCTGGGAGAAGATCTGGA
TGM5	TGCCTGGCTGGAGACCAATG
TGM5	GATGAGTATTATGACAACAC
TGM5	TGGAGTGAGAATTACACAGA
TGM5	TGTGACGGCCTACCAGCTAG
TGM5	ACTTGGACAGTGAACCCCAG
TGM6	GAAGTTGGACACGACCCGTG
TGM6	GAGGTCAGTGATGTCCACGC
TGM6	GAGAAGCACATACGAGCCCA
TGM6	CAAAGCTGTGTTCCAGACAT
TGM6	GTATGTGCTTCTCCACGCCT
TGM7	CTCACCGGTGCACATAACAG
TGM7	CAACACCAGTTCCATCGGGA
TGM7	CCTGTATCACTTAAAGAACC
TGM7	CACAGTGTGACTTACCCGCT
TGM7	GTTCCTATCCACGTTGTGCG
TGS1	AAAGACAGACCACATGCCAG
TGS1	GCAGAATCACATGACAGCAA
TGS1	TTGGTTTATTAACCCATGTG
TGS1	ATCTGTATCACAGCTTTGCG
TGS1	AGAGAAACATTTCCGCCACG
TH	CCTGCGCCCAATGAACCGCG
TH	CGGCGACCCGATTCCCCGTG
TH	GCATGGGCGAGGACGCGTGG
TH	CTACGCCACGCACGCCTGCG
TH	ACAGGCCAAGGGCTTCCGCA
THBD	TTGCTACTGAACGGCGACGG
THBD	GGCACGGCTCGACCTCAATG
THBD	CTACCCTAACTACGACCTGG
THBD	GGTCTCGCACATGCACGAGT
THBD	TGTTGTCTCCCGTAACCCAC
THBS1	GTGGGTCCCATCCGTGCAG
THBS1	AGAGTTGGCCAATGAGCTG
THBS1	CTTGTCATCAGGCACAGGG
THBS1	AATGGCATCATCTGCGGGG
THBS1	CCTCCCCTATGCTATCACAA
THBS2	TGCGGAAAATGAAACGTGGG

THBS2	CATGTCACCACCGAGTACGT
THBS2	TGAGATCCAGCGTGTCCGCG
THBS2	GGAAAAGAGCCGGATGTACG
THBS2	CTACCCCTCACTCACCCACG
THRA	TGGTTCTAGATGACTCGAAG
THRA	GGTAACTAGGGATATACCCT
THRA	TACCGCTGTATCACTTGTGA
THRA	GATCTTGGTAAACTCGCTGA
THRA	CCCATTGTCTCCATGCCGGA
THRB	GAATATTGAGCTAGTCCAAG
THRB	ATCCTCACCTCACAAAACAT
THRB	AGAACCGGGAGAAAAGACGG
THRB	AGTCACGCGAAATCAGTGCC
THRB	AAGTGCCCAGACCTTCCAAA
TIE1	AGACCCACTGTGGATAGACG
TIE1	CTGTCCGCAAGAACCAAGCG
TIE1	ACGTGACGTTAATGAACCTG
TIE1	GCCTGTGGGACGGGACACGG
TIE1	ATAGAGCTACGCAAGCCAGA
TJP1	GTCGCATGTAGATCCAACAA
TJP1	GGAGTGGTGTGGTTAACAGA
TJP1	CGTGTAATGGCAGACTCCGG
TJP1	CCGAAGAGTCCTCAGAACGA
TJP1	ACATACAGTGACGCTTCACA
TK1	GCTGTCATAGGCATCGACGA
TK1	TCTTCCCAGGAACACCATGG
TK1	TTCACCACGCTCTCGGCCAG
TK1	CACTGGATGGGACCTTCCAG
TK1	TGACATCGTGGAGTTCTGCG
TKT	ACAGCCTCGTACATACCCGA
TKT	GAAACAAGCTTTCACCGACG
TKT	TTACCTTGGAGAGCACAAAG
TKT	CCTGCCCAGCTACAAAGTTG
TKT	GGATGGAGCTGATACGTAGG
TLR2	TGGAAACGTTAACAATCCGG
TLR2	AAATCCTTACAAAACCCTAG
TLR2	TACTAGTATTGAAAATCAGT
TLR2	CTTTAAACTCCATTCCCTCA
TLR2	TTAGCAACAGTGACCTACAG
TLR7	AATGGGGCATTATAACAACG
TLR7	CAGCTACTAGAGATACCGCA
TLR7	CAGTCTGTGAAAGGACGCTG
TLR7	GAAGATTATGTAATGGCGAG

TLR7	CATTTGACAGAAATTCCTGG
TLR8	CATCGTTAAAAATGCCCCAG
TLR8	ATTTAAGCGGGAACTGTCCG
TLR8	TCTTACTGAATTGTCCGACT
TLR8	AACTTATCGACTATCAACTT
TLR8	CCTCAACCTAAAAAACCTAA
TLR9	GCTCCGTGAATGAGTGCTCG
TLR9	CACTCGATGAGACCACGCTC
TLR9	GCCCACATCGAGCACACGCA
TLR9	GTTGTTCCGTGACAGATCCA
TLR9	AGGCTGGTGACATTGCCACG
TMPRSS11A	TGGAGTCATTGCACCCAAGG
TMPRSS11A	AGCAATGAGCTCATCAACAG
TMPRSS11A	AGTTCCCCTCTACTGAACAA
TMPRSS11A	CAAATACTAGGAAGTGAACC
TMPRSS11A	GTTCCCCTCTACTGAACAAA
TMPRSS11D	CTCAATAATGCCCACCACTG
TMPRSS11D	ATGTTTCATTGTCGTCGCAG
TMPRSS11D	CAATGTTATTAGGTCTGGAC
TMPRSS11D	GAGCTCATGTTGCCAAACTG
TMPRSS11D	GGAGGCACTGAGGCTGAGGA
TMPRSS11E	AGAGTCTCAGGATCGTTGGT
TMPRSS11E	AAACCTTCGAAAATGAAACG
TMPRSS11E	GAGTGAGGATCTACTTTAGG
TMPRSS11E	AGCAGCACTCACAAGCCATG
TMPRSS11E	GGTGGGACAGAAGTAGAAGA
TMPRSS11F	TTTCGACATTCTTCTGTAGG
TMPRSS11F	CCTCACATTTCGTTTCACTG
TMPRSS11F	CTATTGACAGCAAAAAGATG
TMPRSS11F	TTGTGACTTTAAAAGAGGCA
TMPRSS11F	GATATTTGTGACTTTAAAAG
TMPRSS12	CAAGGGTCTCGGATTATAGG
TMPRSS12	CCCACTGCACTAAAGACGCT
TMPRSS12	GCCTTCCAGAGGGCGAGTAG
TMPRSS12	ACCGGCGGCTAGTTCCCAGC
TMPRSS12	CCGAGCCTGTGCCTTCCAGA
TMPRSS13	GATGATGAGCGAAACCACCA
TMPRSS13	CTGGCAGGAGATGATCGGAT
TMPRSS13	TAGTGCTCACCGGACAACCG
TMPRSS13	GAGCGTGCCTCCACAGATGT
TMPRSS13	GATCAGGTACAAGGAGCAGA
TMPRSS2	ACTGGAACGAGAACTACGGG
TMPRSS2	GTCCAGAACGTCCACGTGTG

TMPRSS2	GGGGACGGGTAGTACTGAGC
TMPRSS2	GCAAATGCCGTCCAATGCCA
TMPRSS2	TGAAAACCATGGATACCAAC
TMPRSS3	AGTCTCGGATTGCAAAGACG
TMPRSS3	CCTACCTCACATATACTGAG
TMPRSS3	GCAGCAGTGATGATCCACAG
TMPRSS3	TGGGGCTGGATTGTCCAACA
TMPRSS3	CATCAAGGCCAAAAAGCGAT
TMPRSS4	CTCTCGCTGAGACAGCCTGT
TMPRSS4	ACAGCCAGGAGCTTCGCATG
TMPRSS4	CCTGGCGAGTATCATCATTG
TMPRSS4	CACCTTCCAGTTGAACACAT
TMPRSS4	GTACTCACCAAGACAGTGCA
TMPRSS6	GGAAGGCACTAGATTCCCGG
TMPRSS6	CTACGCCGAGAGGATCCCCG
TMPRSS6	AGAGTCCATTCACAGAACAG
TMPRSS6	CTACAGGGCCGAGTACGAAG
TMPRSS6	GTAGTAGCTGGGGAAGTACG
TMPRSS7	TCATAAACCGGACCTCTGTG
TMPRSS7	CTGAGGGACACATAAACCGG
TMPRSS7	AAGGGAGTCGTAAATGGTCA
TMPRSS7	AAGGCTGTGAGCATGGATGG
TMPRSS7	TTAAGTCTCCTCATATACGG
TMPRSS9	GAGGTAGGTCGCACCCACGT
TMPRSS9	GCCCTGTCTCCATAGCACGA
TMPRSS9	CAGCGTGCGGTGATAGTCCG
TMPRSS9	AATACCTTGTAGCTTAGGAA
TMPRSS9	GCTCGGCCGTGTGGTCCACG
TMX1	AAGACCTTGGATTGCCAGTG
TMX1	TATTTCTTAGTTATGCCCCG
TMX1	TCAGCTATCTATGTGGATCA
TMX1	GTGCTCCCTGGACGCACGGG
TMX1	GGAACTGCAAGACTCCCGGA
TNC	CTGTTTCGAAGGCTACGCCG
TNC	TTTGTGATGACGGCTACACA
TNC	CCGTCACTTCTGTCACAACG
TNC	ATAGTGAAAAACAATACCCG
TNC	TTGTGATGACGGCTACACAG
TNF	TTGGAGTGATCGGCCCCCAG
TNF	AGAGCTCTTACCTACAACAT
TNF	GGAGCTGAGAGATAACCAGC
TNF	GAGACACTTACTGACTGCCT
TNF	TTCTCCCCAACAGTTCCCCA

TNFRSF11B	TGAAGAATGCCTCCTCACAC
TNFRSF11B	GCAAACTGTATTTCGCTCTG
TNFRSF11B	GCAGTATAGACACTCGTCAC
TNFRSF11B	CAACCGCGTGTGCGAATGCA
TNFRSF11B	GCACCACTCCAAATCCAGGA
TNFRSF1A	GACCAGTCCAATAACCCCTG
TNFRSF1A	AAGACCAAAGAAAATGACCA
TNFRSF1A	GTGGACCGGGACACCGTGTG
TNFRSF1A	AGAGGTGCACGGTCCCATTG
TNFRSF1A	CAGCTGCTCCAAATGCCGAA
TNFRSF1B	AGGAACTGAAACATCAGACG
TNFRSF1B	CTGCGTGTGTTGGGATCGTG
TNFRSF1B	CACCGTGTGTGACTCCTGTG
TNFRSF1B	GGAAACTCAAGCCTGCACTC
TNFRSF1B	CGTGTTGGAGAACGTCCCCG
TNFSF13B	GCTGTCTTGCTGCCTCACGG
TNFSF13B	CAAACTCACTTTCAGTCCCG
TNFSF13B	TGTTTCCATCCTCCCACGGA
TNFSF13B	TGGCTTCTCAGCTTTAAAAG
TNFSF13B	GGTGGTGTCTTTCTACCAGG
TNIK	TGCTCAGCTTGATCGAACAG
TNIK	AGCAACGAGCAGTACAATGT
TNIK	GTTGGTCATCCATGCCTGGT
TNIK	CTCGGCTGAAGTCTAAGAAG
TNIK	GATAGAACAAAGAAGAAGCG
TNK2	GGGGCCCTTCCCTCGCAACG
TNK2	CGGTCCAACAACGATCCCAG
TNK2	GTTCAGTGGAAAGCGACTGG
TNK2	TCCCGCAGGGCCACAAACGT
TNK2	GTACTCACCATCTTCATGGG
TNN	GACTGAGTATAAGATCACGG
TNN	CCACGAAGACTTCATGTCGG
TNN	GGCCACCATTGACAAGTACG
TNN	GTGCACTACACGTCTGCCAA
TNN	GCAGCTGCTCAAGAACACGG
TNR	CAACGGTACCTGTTTATGCG
TNR	TAAAGGACGTAACATCGCTG
TNR	ACTAACAGATCTAGAGCCTG
TNR	GAGACTCTGGCAGAGTACAT
TNR	GCTGGGTTGCTCCAGCCGGG
TNXB	CTCACCAGTCACGCCCACGG
TNXB	GGCCGAGGAGAGTGTCACGA
TNXB	ATGAAGTGACAGCTCATACG

TNXB	GGACCATGAATGAGTCGAAG
TNXB	GGACCACGAAGGAATCAAAG
TOP1	CGACCATGAATATACTACCA
TOP1	TGGAAGAGGCTCATATGGTG
TOP1	ACTCACTCATCCTCATCTCG
TOP1	CAAACATAAAGACAGAGACA
TOP1	TGTCTTCCAGAAAATCAAAG
TOP1MT	GCTCCGATAACACCGTCACG
TOP1MT	TCATGAATACACAACAAAGG
TOP1MT	CCATACGAGCCCCTTCCCGA
TOP1MT	GTGGCGACCATCCCAAGATG
TOP1MT	GCAGCTGGACGTGCTCCACG
TOP2A	TCCCGTCAGAACATGGACCC
TOP2A	AGCATTGTAAAGATGTATCG
TOP2A	TGTACGCTTATCCTGACTGA
TOP2A	ATTCAGTACCAAATTTACTG
TOP2A	TGAACAAGTAAACCACAGGT
TOP2B	TAGGCTACATGGCTTACCAG
TOP2B	GTGTACACTGATATTAACAG
TOP2B	ATGATTATGACCGATCAGGT
TOP2B	ATCAACGTGTAGAGCCTGAG
TOP2B	TGCTGCTGACAATAAACAGA
TP73	GCTGGAAAGTGACCTCAAAG
TP73	CATGCCTGTTTACAAGAAAG
TP73	GGGCGGAACGGATTCCAGCA
TP73	AGAGATTATTGCCTTCCACG
TP73	GGACCAGATGAGCAGCCGCG
TPCN1	AGAAAGACGAGGTAACTCCA
TPCN1	CTGCCGTACCAACACCACGA
TPCN1	AGGACCACGATGAAATAGAA
TPCN1	TTCAAACACTACCACCATCA
TPCN1	GATTACTTACGGGGTCTGAA
TPH1	TAGAAGTACTTACGGCTCTG
TPH1	GGTGATAAGTAACCAGCCAC
TPH1	GAAGAGGAGATTAAGACCTG
TPH1	TCCATACATCAGAACTCTGT
TPH1	GAGATACTCTCTGCAAGCAT
TPH2	GTGGATTGGTAAAAGCACTG
TPH2	GTCCTGCCAGAAAGTCTCGT
TPH2	ATGGAGACATCTTCGAGTTG
TPH2	TTCTTCAGTATACTCCACCC
TPH2	GAGATCTTCCGAGGGAACCA
TPK1	GTTGATGTGATCGTGACACT

TPK1	CTTCGGTGATATCATATAAG
TPK1	GGAAAGAAATACAGTACCAG
TPK1	TGACGAAAATAGTTGTCCAA
TPK1	ACAGTACCAGTGGAAAGCAG
TPO	GGTTTGGACCCACTAATACG
TPO	AGAACACGTTGGACACAGTG
TPO	CGGGGAGCTGCCATACACGG
TPO	CTGCATCGTGGCGTACATGG
TPO	TCTTCCCCTTCATCTCGAGA
TPSAB1	GCAGCGAGTGGGCATCGTCG
TPSAB1	CAGGTGAGCCTGAGAGTCCA
TPSAB1	CTACACCGCCCAGATCGGAG
TPSAB1	CGGTCCCACGCAGTGCGCTG
TPSAB1	GCTGACCGCAGCGCACTGCG
TPSG1	GGAGGTGAAAGTCTCCGTGG
TPSG1	CCTTCGACTTAGGGTGTGGC
TPSG1	GGCTGGGGCTATACGCGGGA
TPSG1	CAGGATGATCTGCCTCACGG
TPSG1	AGTCATCTGAGGCCTCCGGG
TRHR	GATGCTATTGTGATATCCTG
TRHR	TCATGAGAACCAAGCACATG
TRHR	AAAGGCTGTTATTGAACAAG
TRHR	TCCAACATAGCCATAGACCC
TRHR	GGTGGCCTTAGAATACCAGG
TRIM17	TCTGGCGGCTCTCATACAGG
TRIM17	CGTGAGTGTGCAGTGCCCAG
TRIM17	GGGAGCAGATCACCAGGACA
TRIM17	GACCCTGTGATGACCACCTG
TRIM17	GCTGAGCTGGGAAAAGGCGA
TRIM21	GAAACACCGTGACCACGCCA
TRIM21	TCATCTCAGAGCTAGATCGA
TRIM21	ATCCAGACACAGCCAATCCG
TRIM21	GAGCCTGTGAGCATCGAGTG
TRIM21	GGAGCAGCTGAGAATCCTGG
TRIM25	TGTTCCGGGGCTCCAAACGT
TRIM25	GCAGCTACAACAAGAATACA
TRIM25	CAGAAAGCATCAAAACTGCG
TRIM25	TGGTAGACGGCGCGGCACTG
TRIM25	GAGCCGGTCACCACTCCGTG
TRPA1	TGCACAAATAGACCCAGTGG
TRPA1	TTCTACAGCACAATGCAGAG
TRPA1	ATTAATAAGATCGATTCTGA
TRPA1	CCATGTAAATCAAATAAATG

TRPA1	GTAGGTTATTTACAGAACCA
TRPC1	GGATGCATTCCATCCTACAC
TRPC1	GAGGCTCGTCACTAGACGTA
TRPC1	CATTCACAGCCAACTGCATG
TRPC1	TCTTACAGGTGGGCTTGCGT
TRPC1	GTACAGGGCCGCCATCATCG
TRPC3	GTGGGAGTGTCACTTCACTG
TRPC3	CTGGATCGCACCTTGCAGCA
TRPC3	CGACTTCTACGCTTACGACG
TRPC3	CGAGGATCAATGCCTACAAG
TRPC3	TTACCTCTCATTGTCCAGCG
TRPC4	CAGTGTCAAGAAATCCCTAG
TRPC4	TAATATCATCCACTCGACGA
TRPC4	CACATGTCCCATGATTCTCG
TRPC4	GCTATCAGAAAAGAAGTCGT
TRPC4	AGGAGAGCAGTTCTTCCGAG
TRPC5	AAAAACGGGTCACTATCCCA
TRPC5	CTGAACCACAGCGTGTATGT
TRPC5	GGACAGACCTTCATGTACAG
TRPC5	AGGGAAGCCATCATACCACA
TRPC5	GCAGTTCTCTGAATTCACAC
TRPC6	GGGAGAAGGTTAGCTAATCG
TRPC6	GATGTTGAAACGCTCCAGAG
TRPC6	AAGCATCCCCAACTCGAGAG
TRPC6	TTTCCTAGCCAGGATAAAGT
TRPC6	GCGCTGCCGGAGCCGCTGCG
TRPM1	ATCCACCAGATCACTCTCGG
TRPM1	AGGTGTTATCAGCCACGTAG
TRPM1	AGGCTGATGTGGTAATCAGG
TRPM1	GGCTTCGTGCTATGTCCACG
TRPM1	TGAAGAGGAAAGCAAACAGG
TRPM2	ATGAAGTTTGTGTCTCACGG
TRPM2	GATGGCACACTCGCACCAGG
TRPM2	CGCACGTTGAGCTTGACGTG
TRPM2	CCAGCACCACGCACACGATG
TRPM2	AGAACTTCAACATGAAGCCG
TRPM6	GGAGCGTGGATAATAACTGA
TRPM6	GGATGGTAAGAAAGCGATGG
TRPM6	TCTGGAATAGGCTCAAGACA
TRPM6	AGGTCATGATGTAGCGATAG
TRPM6	TTAGTAGTAGAATACCTCAT
TRPM7	ACAGGTGTGGCAAAACATGT
TRPM7	CTTCAGACAAGATGAAACCA

TRPM7	CAAATATAAGTGCCACCACA
TRPM7	GTTATTGAATATCTCATGGG
TRPM7	GCATACAGAACAGAGCCCAA
TRPM8	GGTGCTTGGATTCTCACGGG
TRPM8	CTGGTTGCGAACTTCCGAAG
TRPM8	GGCTAATGAGTACGAGACCC
TRPM8	TTTCACCAATGACCGCCGAT
TRPM8	GCTTCGTGCTTACCTCCCAT
TRPV1	GCTCCACCAAGAGCATGTCG
TRPV1	ATCAGCGCCAGGGACTCGGT
TRPV1	GCCATGCTCAACCTGCACGA
TRPV1	TGGAAGCCACATACTCCTTG
TRPV1	CCTGCGATCATAGAGCCTGA
TRPV2	ACATGTAGATCAGATTACAC
TRPV2	GTTGTCCGAGATCATCACTA
TRPV2	AGTCAACCTCAACTACCGAA
TRPV2	ACGAGATCCAGATGAACACG
TRPV2	GCTGTGCTGAACCTTAAGGA
TRPV3	TGTACGACATGATCCTACTG
TRPV3	AACCCCAAGTACCAACACGA
TRPV3	GATGTCGTTCTCTTCAGCAA
TRPV3	CACGGGTCCGTACGCCCAGT
TRPV3	TGAACATCGCCATCGAGCGG
TRPV4	CACCCTATATGAGTCCTCGG
TRPV4	GCGTCGCTGCAAACACTACG
TRPV4	GAGGCCGTGCTCAACAACGA
TRPV4	GTGGTGCGGTAAGGGTACGG
TRPV4	AATCTGCGCATGAAGTTCCA
TRPV5	TGCCACCACGAAACTTAAGG
TRPV5	CGTCTTTCCAAAATAGCGAG
TRPV5	GGTCTCCTCTGATAAACGAG
TRPV5	AGTGTCATGTATTTCACTCG
TRPV5	TGATGTCTCGAGAATGAGTG
TSHR	TTATGACTACACCATATGTG
TSHR	CCCCATGCAGAAATCCGCAA
TSHR	AATAAACTTTGGTCAGGTCA
TSHR	CATTACACATCAAGGACTCA
TSHR	CACAGAGTCTGCGTACTGGG
TSPO	GCACGCTCTACTCAGCCATG
TSPO	TCTGCAGGCCGGCGTACCAG
TSPO	TGGTGCCCGACAAATGGGCT
TSPO	GGGGGGCCATGCCCAGTTCA
TSPO	GTGCACGCCCACCTACCCCA

TST	CATCAGCAACCACACGCACG
TST	ACCGTATCAGTGCTCAATGG
TST	TGAATCTAAGAGGTTCCAGC
TST	AGGACGCGTCCAGCACCCGC
TST	GTACTCACCAGGCACCCGAG
TTK	GATGATGGCAAACAACCCAG
TTK	AAATGCTGGAAATTGCCCTG
TTK	GAGGACAGACTACTAAAGCC
TTK	AAAGACAGGTTGCTCAAAAG
TTK	TTGGAGGTTTAAATTCCGCA
TTN	AGAACCTGCAACAATCACCG
TTN	GTCCTTGTAGGATAGCAATG
TTN	AGAGGTTCAATAAAGTACGG
TTN	GTACCTAACGACGAAAGGTG
TTN	GTAGCCCTCTTGCTTCCAAG
TTPA	CATAAGCTGTAAAAACTTTG
TTPA	CAGGTCCAGATCGAAATCCC
TTPA	ATAAGAACTTTGCTGCCAGT
TTPA	TACCTCCTGTACAATAAGCT
TTPA	CCATGGAGTCCTGAGATCCA
TTR	GCTGCATGGGCTCACAACTG
TTR	ACACAAATACCAGTCCAGCA
TTR	AAAGGCTGCTGATGACACCT
TTR	TCTAGAACTTTGACCATCAG
TTR	ATACCAGTCCAGCAAGGCAG
TUBA1A	CTGTGATAAGTTGCTCAGGG
TUBA1A	AGGTTGGACGCTCAATATCG
TUBA1A	CTGGAGACCCGTGCACTGGT
TUBA1A	TACAGAAAGCTGTTCATGGT
TUBA1A	TTATAGGCCGACCAGTGCAC
TUBA1B	AAGTCTACAAACACAGCCCG
TUBA1B	CTGTGATGAGCTGCTCAGGG
TUBA1B	TGCGAATTCGGTCCAACACA
TUBA1B	CCAGAGGGAAGTGGATGCGG
TUBA1B	CCAAGTCTACAAACACAGCC
TUBA3E	TGCAGCCAGTAATTACGCCA
TUBA3E	GGATGCGGGGGTACGGCACG
TUBA3E	CGGCCACAGACAGCTGCTCG
TUBA3E	CCTAACCACCCACACGACCC
TUBA3E	GCAGCCAGTAATTACGCCAG
TUBA8	GGCAGAGATGATGGGCGCGT
TUBA8	AGATCTATCATGACGGCCCG
TUBA8	TTCAGATTGTGCTTTCATGG

TUBA8	CAGTGCCCCCACCAAAACTG
TUBA8	GCAGCTGATCACAGGAAAGG
TUBAL3	GCTGCTAACAATTACGCGCG
TUBAL3	TTTCTCCAGGCAGAACAGTG
TUBAL3	TCTCGGATAAGGTACCAGGT
TUBAL3	GTGGTGAGGACAGAGTTATA
TUBAL3	GCTCGGGGTGGAAGAGTGAA
TUBB	GCTGACCACACCAACCTACG
TUBB	CCCCACCGGCACCTACCACG
TUBB	AGATCCACCAGGATGGCACG
TUBB	CTGCATTCCAGGTCAGTCTG
TUBB	TGTTCCTCGTGCCATCCTGG
TUBB1	GCTGATCGAGAATGTCCTAG
TUBB1	GCTGACGACACCCACCTATG
TUBB1	TGTGGTGGAGCCCTACAACG
TUBB1	GCTCATGAACAAGATTAGAG
TUBB1	GCTGCAAGGCCGAGGCCCCG
TUBB2A	AACCTACTCCATTGATAACG
TUBB2A	AGATCCACCAGGATGGCCCG
TUBB2A	CTCCATGGTAACTGCCTGTG
TUBB2A	CAGCTGACCCACTCTCTGGG
TUBB2A	TGTACCTCGGGCCATCCTGG
TUBB2B	TGCCTGGCTCCAGATCCACG
TUBB2B	GAGCATGGGATTGACCCCAC
TUBB2B	AACCTACTGCATTGACAACG
TUBB2B	CGTGCCGCCCCCCAGAGAGT
TUBB2B	GATGTGCACGATCTCACGCA
TUBB3	CTGGCCCGGGAAGCGCAAGG
TUBB3	CATGGACAGTGTCCGCTCAG
TUBB3	CAGCTGGTGGATGGACAGCG
TUBB3	CTGGGCCAAGGGTCACTACA
TUBB3	TCATGGTGGCCGATACCAGG
TUBB4A	GCTCATCAGTAAGATCCGCG
TUBB4A	CAGCTGGTGCACAGACAGCG
TUBB4A	CGATGCCAAGAACATGATGG
TUBB4A	GGCAAAGGGGCACTACACGG
TUBB4A	CATCATGAACACCTTCAGCG
TUBB4B	GCTGGATGTTGTGAGAAAGG
TUBB4B	CAAAGTGTCAGACACAGTGG
TUBB4B	CCAGATCCACGAGCACGGCG
TUBB4B	CCCCGTGGTAGGTGCCCGTG
TUBB4B	CCTCATCAGCAAGATCCGGG
TUBB6	CAGCTGGTGCACCGACAGTG

TUBB6	CTGGGCGAAAGGGCACTACA
TUBB6	GCTGACAACGCCCACCTACG
TUBB6	GACCCGGCCGGAGGCTACGT
TUBB6	GCCTGGCTCTAAGTCCACCA
TXK	GGAAGGCAAGAGACCGTTTG
TXK	TGGTCCATTTAGGTGAATGG
TXK	AGAACATCTATTGAGACAAG
TXK	ACCGTAGCTAAACCCAGCTG
TXK	TCTTGATTGTAGAAGTACGG
TXNDC12	GTGGAATATAACCCCCGTCA
TXNDC12	TGGTGATTATTCATAAATCC
TXNDC12	TCATATTCATTGGAGGACAC
TXNDC12	TGGACATAATGGGCTTGGAA
TXNDC12	TTATGAATAATCACCATCAG
TXNL4B	TAGCAAAAAGGAAGTAGACC
TXNL4B	GATAAAAAGTACTGCTGAGA
TXNL4B	AGCATATGAAAGTGGATTAT
TXNL4B	CAGTTTGGTCCACATCTACC
TXNL4B	GCCCAAGCTGACTAGCAAAA
TXNRD1	ACTGAGCTACTACTCTGAGT
TXNRD1	ATAGGATGCTCCAACAACCA
TXNRD1	TCTTCTTAGAGGATTTGACC
TXNRD1	TCTCTGTTTCACAAACACAA
TXNRD1	TTACCCCATCTAGTTCCAAG
TXNRD2	CCAGATCCTTACCAGTCATG
TXNRD2	GAATCCCCTGGAAAAACGTA
TXNRD2	ATAGAGCACATGGCATCTCA
TXNRD2	TCATCATTGCTACTGGAGGG
TXNRD2	GCGGGACTATGATCTCCTGG
TXNRD3	GCGCCTACCCGAGTACTATG
TXNRD3	TCACAGGTATGAATTTCCGT
TXNRD3	CCTGGCAAAACATTAGTGGT
TXNRD3	CACATCAGCTCTCTAAACTG
TXNRD3	TGCTCCCACACCACTGCAG
TYK2	TGAATGACGTGGCATCACTG
TYK2	CAGGCGGCCCTCATACACGT
TYK2	AATACCTAGCCACACTCGAG
TYK2	TTGGGCCTGAGCATCGAAGA
TYK2	GCAAAGAGATTGAAGCAAGG
TYMS	ATGTGCGCTTGGAATCCAAG
TYMS	TCTACAGATTATTCAGGACA
TYMS	TTCCAAGGGAGTGAAAATCT
TYMS	ACCAAACGTGTGTTCTGGAA

TYMS	CTGCATGCCGAATACCGACA
TYRO3	GGAGTTTGACCATCCACACG
TYRO3	AGAAAGGCCCGAGCGCATCG
TYRO3	TGCGCTGTGCCAATGCCTTG
TYRO3	CCTACCTTGAAGGTGAACAG
TYRO3	AGACACTGTCAGCTTCACCG
TYRP1	ACTTCTCAAAGCCTCAACAG
TYRP1	AATAGGACATGTCACTGCAA
TYRP1	ATCTGCACGGATGACTTGAT
TYRP1	ACTGTTACAAAGTGTTCCCA
TYRP1	GTCCTATTGAAGAAGCGCAA
UCP1	GGGCGGATGAAACTCTACAG
UCP1	ATTCATTGGGCAACCCACAG
UCP1	CAGGATCGGCCTCTACGACA
UCP1	GAATCAAACCTCGCTACACG
UCP1	TGAGAAGAGCTGGACCCCCA
UCP2	CAGAATCATACAGGCCGATG
UCP2	CAGCTCAGCACAGTTGACAA
UCP2	CTACAAGACCATTGCCCGAG
UCP2	GCCCATTGTAGAGGCTTCGG
UCP2	GAATGGTGCCCATCACACCG
UCP3	TGTCCAGTGGAAAGGTAACG
UCP3	GGTGTACACCCCCAAAGGCG
UCP3	CGCCTACAGAACCATCGCCA
UCP3	TATCGTCAACTGTGCTGAGG
UCP3	TATTGTCCCTCAGATCCAGG
UGCG	TCCAGATACGCTTACTGACA
UGCG	CCGATTACACCTCAACAAGA
UGCG	TGGCCAAAGCGATAGCTGAC
UGCG	CATCATGATCTTGTACACAA
UGCG	CCTTACGTAGCAGACAGACA
ULK1	GGCAGCGTCCGGTTTCGAGG
ULK1	CCACCCAGTTCCAAACACCT
ULK1	GACCCCGAGCTTGGGTACGA
ULK1	CAGCTGACTTCGGCTTCGCG
ULK1	GGAGAACTCGAACTTGCCCA
ULK2	AGGCCCATGACGAGTAACCA
ULK2	TACCTTGCAAATAATCTGCG
ULK2	ACCCGATAAGAGAGACTGTG
ULK2	TGGTCTGACGAGATGTTGTG
ULK2	GTAAGGCCTAGAAGACCCAG
ULK3	CACGTGCTTCTCATCCCACG
ULK3	CATGGACCAGAGGTCCACGC

ULK3	GGATCTCAATCTCCGTGAGG
ULK3	CCGTAGCAACCGGGTCATCG
ULK3	CCATGGACCAGAGGTCCACG
UNG	TCCAACTGCTCGGCACTCAG
UNG	ATGGACCTAATCAAGCTCAC
UNG	GCGGCCCGCAACGTGCCCGT
UNG	GTCCAGGTGAAGACTTGGTG
UNG	GATCCAGAGGAACAAGGCCG
UQCRC1	ATGTCCATGGGATGCCACCG
UQCRC1	AAGGTAGAGCATCATCACGG
UQCRC1	GGGCCTTGTAATGTGTGCTG
UQCRC1	CACCGTGCAAGTGGGCTGAG
UQCRC1	GCTATTGCGCGCCCGCCGCT
UROD	GTGGACCCTGATGACATACA
UROD	ACTGCCTTACATCCGTGATG
UROD	CTGGGGTACAACAAGGATGT
UROD	ATATCTGGTAGGACAAGTGG
UROD	GCACCAGCAAAGCCAATCAG
VAMP2	GGACATCATGAGGGTGAACG
VAMP2	CCGTGCAGATGCACTCCAGG
VAMP2	CACACTCACCTCATCCACCT
VAMP2	GGAGCGAGACCAGAAGCTGT
VAMP2	TTACTGGTGAGGTTTGGAGG
VARS	CCGCAGCGAACGTACCACTG
VARS	TGTCACCCCAGATAGCGACG
VARS	GTGGGTCAGTTACGCCGACA
VARS	GGTGAGTGGACGCAATGAGG
VARS	CTTCCTGTAGAAAGGCCTCG
VAT1	TCAGTCGTGTGATAGTCGAT
VAT1	CCATGTGTACCAAGACGCTG
VAT1	TTCGCCGCCTCCGAAAACCG
VAT1	TGATGGTGTTGAACCGGTCA
VAT1	GTAGGTTGCCGAAGTCAAAG
VCAM1	CCAATCTGAGCAGCAATCCG
VCAM1	TAAGTAATTCAATCTCCAGC
VCAM1	ATGGGAAGGTGACGAATGAG
VCAM1	CTGATGTATACCCATTTGAC
VCAM1	CCAGAAATCGAGATGAGTGG
VDAC1	GCTCTGGTGCTAGGTTACGA
VDAC1	TGGAATACCGACAATACACT
VDAC1	AAGCGGGAGCACATTAACCT
VDAC1	GCAACACTCACCATAGCCCT
VDAC1	GATGTCTTCACCAAGGGCTA

VDAC2	AATCAAGTCTTCTTACAAGA
VDAC2	CAAAGTCAACATCACAACCA
VDAC2	AGAAATCGCAATTGAAGACC
VDAC2	GTGCCAAATCAAAGCTGACA
VDAC2	CTACCTTCTCACCAAACACA
VDR	ACAGCTCTAGGGTCACAGAA
VDR	CCACACACCCCACAGATCCG
VDR	CTGCCGGCTCAAACGCTGTG
VDR	CCATCATTCACACGAACTGG
VDR	ATTCACCTGCCCCTTCAACG
VEGFA	TGGTTTCGGAGGCCCGACCG
VEGFA	GGAGGGCAGAATCATCACGA
VEGFA	GGAGGAAGAGTAGCTCGCCG
VEGFA	AGATGTACTCGATCTCATCA
VEGFA	GCTCTACCTCCACCATGCCA
VIPR1	AGGGGTCTTACCTTGAATGG
VIPR1	CTTCACAGAACCGTAGAACA
VIPR1	GGAGTCGGACCAGTGCTCCG
VIPR1	ACAGGAAGCTCCACTGCACG
VIPR1	GGTACCCAGCACATTCACCA
VIPR2	ACTTACCCCATCCGATCAGG
VIPR2	CAATGTGGGAGAGACCGTCA
VIPR2	ATTTCATCTGGAAATACAGG
VIPR2	CCAGATTTCGTCGATGCCTG
VIPR2	TCTCAAACAGAAAAACACAA
VKORC1	GATGCAACCGAATATGCTGT
VKORC1	GACGCGCGAACAGCTGATGG
VKORC1	GCTCTACGCGCTGCACGTGA
VKORC1	AGGTTGCCTGCGGACACGCT
VKORC1	TGCCCACGTCGCAGAGCGCG
VLDLR	CCAGCCAGCGAAATCCAGTG
VLDLR	TCTGACTTCGTGTGCAACAA
VLDLR	GGTATCCGAGACTGTGTCGA
VLDLR	GGTGAAAATGATTGTGACAG
VLDLR	GGAGAAGATGAAGAAAACTG
VRK3	TGTGGGCAAAGCTTCAAGTG
VRK3	TCTGAGAAGAGGGATAATCG
VRK3	TTCCAGACCAGGGACAACCA
VRK3	TGACACATGTGGATTGACAA
VRK3	CTGTGGCAAAAGTATCCAAG
VTN	AGCCGTCAGAGATATTTCGG
VTN	TGGGCGCCTCTAAGCCTGAG
VTN	GAGTACACGGTCTATGACGA

VTN	AAGCTCATCCGAGATGTCTG
VTN	TTCCTCATAGAGTCATGCAA
VWF	ATTCCCCACTAGGATCCGAA
VWF	TTCATGCACTGTACCATGAG
VWF	TCTTTCCTGAGGCAAAACGC
VWF	GGCCTGTGTGGGAATTACAA
VWF	GCTCAAATACCTGTTCCCCG
WARS	GCTCGTAAGGTCCCTCAAAG
WARS	AGCCTTGTAATCCTCCCCCG
WARS	TCTCTGACCTGGACTACATG
WARS	CCAGGCCTATAGCTATGCTG
WARS	TGAGCTACAAAGCTGCCGCG
WARS2	CTCCCCAACAGGAACGTGTG
WARS2	CTAGTTCAGGATCTAGCACA
WARS2	ACTACCAAGCAGAAGCACGA
WARS2	CTCCACCTGGGCAATTACCT
WARS2	AGATGGCGCTGCACTCAATG
WAS	TCACGAGTTCACGATACCGT
WAS	ATGTGCAGGACTGCCAAGCG
WAS	ACAACCTCGACCCAGATCTG
WAS	CGGATGAAGTAGGACTTCTG
WAS	GTATGTGCAGGACTGCCAAG
WEE1	TCATCAACAGAGCCCGCCAA
WEE1	CCATGAAGAGAGAACTACCC
WEE1	CCAGGAGATGCGTCGCCGCG
WEE1	ATAGGATGCCTTTTAAACG
WEE1	TCTACGACGACACTGTCCTG
WNK1	GATCCCGGGGGTATCAACTG
WNK1	GCCGTGGGAATGTCTAACGA
WNK1	TTTCTCCTCATACATCTCAG
WNK1	GCTGATGGGACGGTTGACAG
WNK1	TTAAGAGGAAACGAGAGCAG
WNK4	GTACGAGGAAAAGTACGATG
WNK4	CTTGAGGTATCGGCCATCGG
WNK4	AGCTCCAAAGAACCCCCCGA
WNK4	TGAAGCCGATTACCAGCCAG
WNK4	CCACAAGGTGAAGATACCCG
WRN	GTAAATTGGAAAACCCACGG
WRN	ATCCTGTGGAACATACCATG
WRN	GAATACTGTTCAGGTAACAT
WRN	TAGCATGAGTCTATCAGATG
WRN	TCTTCCATCAGAGAAATAAG
WWOX	GCCGTCGTATCTTTGCCGGG

WWOX	ACACCGAGGAGAAGACTCAG
WWOX	CAAGGTAGAAGCAATGACCC
WWOX	CCAAGATCACATGTGCACCA
WWOX	TGGCAGCGCTGCGCTACGCG
XDH	CATACTCATGACGATGCCAG
XDH	TGCTTGCCCCCTGAGCATTG
XDH	TGGACCACTTCAGCAATGTG
XDH	AAGTTGCACTGGCGAAAGTG
XDH	GCTTCCTGCCCAAAAGACAG
XIAP	ATGACAACTAAAGCACCGCA
XIAP	ATGGATATACTCAGTTAACA
XIAP	TCTGACCAGGCACGATCACA
XIAP	TATCAGACACCATATACCCG
XIAP	CTGGTGAAGGAGATACCGTG
XPO1	AGTGAGCTCTCAAAAAACGT
XPO1	TAGTCGAATGGCTAAACCAG
XPO1	TCTCAGGGAAACTCTTATGG
XPO1	TCACACCAGCAATCTCAGTG
XPO1	TTTCTGAACTGGATTCCCCT
YARS	AGAGTGTTACCGGACTTAAG
YARS	GAGTTCTAGAAGTTCCCATG
YARS	TGATCTCCTTGATCGGAAGG
YARS	CGGGAACTTAAAATTTACTG
YARS	TGTTATCCAGGTATGCGTGG
YARS2	CATGAAGCGAGTCTGCCGTG
YARS2	GGGCCACAACGTGATCGCGC
YARS2	GGACCCTGCATCCATAACGC
YARS2	TAACCCCTGAGCGCCCGAGT
YARS2	GCACCTGGTGGACTTCCTGG
YES1	CTAGTCGCAAAGATTCTCGA
YES1	TCCAAAAGGCGTTACCCCTG
YES1	AAATTGGTGAAACACTACAC
YES1	AGAGAGAGTGAAACAACTAA
YES1	TTGAATCCTGGAAATCAACG
ZAP70	CCAACTCACATGAGTCAACG
ZAP70	GCCGGCGGCAAAGCGCACTG
ZAP70	CGAGCGCAAACTTTACTCTG
ZAP70	GGCAAGTACTGCATTCCCGA
ZAP70	GCTGACATTGAACTTGGCTG
BRDT	CAACTCCAGTTCACAAACTG
BRDT	TGACGTTGTCAAAAATCCGA
BRDT	AACTCCCTGGAGATAAACTT
BRDT	ATGGCCCTTTCAACGTCCTG

BRDT	GCTCCCTGTACCACGTTCAA
Non-Targeting Control	AAAACAGGACGATGTGCGGC
Non-Targeting Control	AAAACATCGACCGAAAGCGT
Non-Targeting Control	AAAATAGCAGTAAACTCAAC
Non-Targeting Control	AAAATCGATGGGCTGAATCT
Non-Targeting Control	AAAATTATCGGAAACGGTAG
Non-Targeting Control	AAACCCTATGCCCAAATGAG
Non-Targeting Control	AACTACAAGTAAAAGTATCG
Non-Targeting Control	AACTAGAATAGGCGGGCTTG
Non-Targeting Control	AACTAGCCCGAGCAGCTTCG
Non-Targeting Control	AAGAAGAATTGGGGATGATG
Non-Targeting Control	AAGAAGGGCCGTACCCGAAA
Non-Targeting Control	AAGGGCGTGCCCTGCGTTGT
Non-Targeting Control	AAGTGACAGATGGGCAGGCG
Non-Targeting Control	AAGTGACGGTGTCATGCGGG
Non-Targeting Control	AAGTGTGTGCATAGCAGGGT
Non-Targeting Control	AATATTTGGCTCGGCTGCGC
Non-Targeting Control	ACACCCATTCTCATAACGGA
Non-Targeting Control	ACACCGAAGCACCTGTACGT
Non-Targeting Control	ACAGCCCTCACGAGCCCGAA
Non-Targeting Control	ACAGCGCTCTCGTGTACTAT
Non-Targeting Control	ACAGGTTCTTATTCATTGAC
Non-Targeting Control	ACCCGATAATAGCTACTGGT
Non-Targeting Control	ACCCTCCGAATCGTAACGGA
Non-Targeting Control	ACCGCTCATATAGGTAAAAA
Non-Targeting Control	ACCTATAATCGACCACATTT
Non-Targeting Control	ACCTATTGTCCCTTCAAGCT
Non-Targeting Control	ACGTCAACTGCTGGAGTGGG
Non-Targeting Control	ACGTCCATACTGTCGGCTAC
Non-Targeting Control	ACGTCGTTTAGCACCCGGCT
Non-Targeting Control	ACGTGGGGACATATACGTGT
Non-Targeting Control	ACGTTCGAGTACGACCAGCT
Non-Targeting Control	AGCCGGCTTGTGACAGTGAA
Non-Targeting Control	AGCGATCTGGACACTCTCCA
Non-Targeting Control	AGCGATTCACGTATTAGATG
Non-Targeting Control	AGCGCAGATAGCGCGTATCA
Non-Targeting Control	AGCGCTCTGGTTGCATCCCT
Non-Targeting Control	ATTAGGCCTTTTTCTTAACT
Non-Targeting Control	ATTCAGCGCGCTCGCCCTGG
Non-Targeting Control	ATTCATGCGCCGCCTCCTCT
Non-Targeting Control	ATTCCTTCGGCGCTCTGCGT
Non-Targeting Control	ATTGAGAATTCGTTTCAAGG

Non-Targeting Control	CAAATGCCATTTAGGTTATC
Non-Targeting Control	CAACACCCCGCGTTATGCTA
Non-Targeting Control	CAACCGGCGGGCCCCTACAA
Non-Targeting Control	CAACGACGGGCCTAGTCTCA
Non-Targeting Control	CAACGGGTTCTCCCGGCTAC
Non-Targeting Control	CCATCACCGATCGTGAGCCT
Non-Targeting Control	CCATTCACAATCCCACTACA
Non-Targeting Control	CCATTCCGTAAGGGCTTGGA
Non-Targeting Control	CCATTCTCAACCGGTCCAAT
Non-Targeting Control	CCCAATGGCTTCTGCGTGAC
Non-Targeting Control	CGAAACCCTCTTAAGTTAAC
Non-Targeting Control	CGAAACCTCCTAACTGAGAG
Non-Targeting Control	CGAACTTAATCCCGTGGCAA
Non-Targeting Control	CGAACTTCTGGCTGCAGTTT
Non-Targeting Control	CGAAGTCTTTCTTAGATGGT
Non-Targeting Control	CGCCGGGCTGACAATTAACG
Non-Targeting Control	CGCCGTTCCGAGATACTTGA
Non-Targeting Control	CGCCTAATTTCCGGATCAAT
Non-Targeting Control	CGCCTCTCACGTGTAGGCTT
Non-Targeting Control	CGCGACGACTCAACCTAGTC
Non-Targeting Control	CTAAAATTTTTGCGTGTTTG
Non-Targeting Control	CTAACATGAGTACATAGATA
Non-Targeting Control	CTAACGGACTGCAGAACGGA
Non-Targeting Control	CTAAGTTTGTTAATGGGCCA
Non-Targeting Control	CTAATCACGACCTCACCCTA
Non-Targeting Control	GAAAACACGATGACGTCTCT
Non-Targeting Control	GAAACGAGAAGTTTGTACTA
Non-Targeting Control	GAAAGGCATAGTGAGAATGG
Non-Targeting Control	GAAATGCTATGCTTCGGTTC
Non-Targeting Control	GAACCCAACCTTTTACCGCA
Non-Targeting Control	GAGGACCTTAAGGTGACATG
Non-Targeting Control	GAGGGGGCTTCAAACATGTG
Non-Targeting Control	GAGGTATGTCATCGCCATGA
Non-Targeting Control	GAGTAATTTCGAACGTATTG
Non-Targeting Control	GAGTACAGCGATTCCTCATG
Non-Targeting Control	GCCCCAAGCTAGAACTCAGC
Non-Targeting Control	GCCCCGCCGCCCTCCCCTCC
Non-Targeting Control	GCCCCGTAAATCTCATTACA
Non-Targeting Control	GCCCCTTATGATTGTTATAG
Non-Targeting Control	GCCCTTCAATGCGTTCCGTA
Non-Targeting Control	GGGCGTGTATGTTCGTATTG
Non-Targeting Control	GGGGAAACAAGTAGGCTTTG

Non-Targeting Control	GGGGCAGGGACCGAGTATCC
Non-Targeting Control	GGGGCTTACGTGAAGGGCGG
Non-Targeting Control	GGGTATAGACGCGATCCTCA
Non-Targeting Control	TAAAGCAGAAGAATATACAG
Non-Targeting Control	TAAATTCAGACCACAGCTAA
Non-Targeting Control	TAACCCAGAAGCCCATTCAG
Non-Targeting Control	TAACCGATACTCCCCACATT
Non-Targeting Control	TAACGCGCATATCTGAACAC
Non-Targeting Control	TCATCTTACATCTGGGAGAC
Non-Targeting Control	TCATGCTTGCTTGGGCAAAA
Non-Targeting Control	TCCAGCGCGAGCTTACTCGT
Non-Targeting Control	TCCCAAGGGTTTAAGTCGGG
Non-Targeting Control	TCCCCGAGACCATCTTAGGG
Non-Targeting Control	TTGCAATGCTGCTATAGAAG
Non-Targeting Control	TTGCAGCCACTCCTGCAATA
Non-Targeting Control	TTGCGTCAGCGCTGCACATC
Non-Targeting Control	TTGGATATTAATTAGACATG

Supplementary Table 3. The small-molecule compounds information of library.

Raddeanoside R8	HY-107242
Lucidenic acid D	HY-107260
Imperialine 3-β-D-glucoside	HY-107271
Officinalisinin I	HY-107284
Norswertianolin	HY-N6617
Sabinene	HY-108943
6-(γ,γ-Dimethylallylamino)purine	HY-112103
(E)-tri-Pcoumaroylspermidine	HY-114292A
Chrysanthemic acid	HY-114502
2-Hydroxymethyltetrahydropyran	HY-115051
Anisylacetone	HY-116047
13(E)-Docosenoic acid	HY-116739
5-Pentadecylresorcinol	HY-116934
trans-β-Ocimene	HY-117215A
(±)-Lavandulyl acetate	HY-117419A
Isogentisin	HY-118622
N-Feruloylserotonin	HY-118824A
Leucocyanidin	HY-119580
Rotenolone	HY-119694
Aerugidiol	HY-121083

(-)-Lupinine	HY-121219
Kadsurin	HY-121271
Gypenoside XIII	HY-N6881
Yuankanin	HY-121597
Yibeissine	HY-121631
Militarine	HY-122308
Ganoderic acid N	HY-123100
Linamarin	HY-123114
Lobetyolinin	HY-124031
Fenchone	HY-124215
Kansuiphorin C	HY-125120
Dactylorhin A	HY-125531
Salvianolic acid F	HY-125847
Cyanidin 3,5-diglucoside (chloride)	HY-129138
6-Hydroxyluteolin 7-glucoside	HY-129529
trans-2-Undecenoic acid	HY-133022
Chrysoobtusin	HY-133860
β-Bisabolene	HY-136552
β-Gentiobiose	HY-137940
Erlose	HY-139338
Oleyl alcohol	HY-141573
Rocagloic acid	HY-19355
5-Hydroxyflavone	HY-22024
N-Phenethylbenzamide	HY-32135
Azetidine-2-carboxylic acid	HY-75308
Ammonium glycyrrhizinate	HY-76225
Methyl N-methylanthranilate	HY-76705
Hydroquinidine	HY-B0997
Methyl nicotinate	HY-B1695
Casanthranol	HY-B2134
(E/Z)-Demethoxycurcumin	HY-N0006A
2'-Acetylacteoside	HY-N0026
Forsythiaside A	HY-N0028
Notoginsenoside Fe	HY-N0046
Allomatrine	HY-N0050
Angoroside C	HY-N0062
(Rac)-Byakangelicin	HY-N0075
Evodiamine	HY-N0114
(E/Z)-Polydatin	HY-N0120
(-)-Sesamin	HY-N0121A
Methyl-Hesperidin	HY-N0165
4-Methylumbelliferone	HY-N0187

Amygdalin	HY-N0190
Pulchinenoside C	HY-N0205
Scoparone	HY-N0228
Atractyloside A	HY-N0237
Atractylodin	HY-N0238
Hederacoside D	HY-N0254
Epimedin A1	HY-N0258
Epimedin B	HY-N0259
Epmedin C	HY-N0260
Toosendanin	HY-N0263
Dipsacoside B	HY-N0266
Caftaric acid	HY-N0321
Deltaline	HY-N0329
Yunaconitine	HY-N0333
(-)-Syringaresnol-4-O-β-D-apiofuranosyl-(1→2)-β-D-glucopyranoside	HY-N0338
Helicid	HY-N0343
Farrerol	HY-N0344
3,29-Dibenzoyl rarounitriol	HY-N0357
Nardosinone	HY-N0380
Griffonilide	HY-N0386
Gelsemine	HY-N0388
Fructose	HY-N0395
Picroside III	HY-N0409
Sesamoside	HY-N0412
Quercimeritrin	HY-N0419
Sophoricoside	HY-N0423
Cyclogalegenin	HY-N0424
Alismoxide	HY-N0426
5-O-Methylvisammioside	HY-N0442
Rubiadin	HY-N0444
3,5-O-Dicaffeoylquinic acid	HY-N0459
Blinin	HY-N0463
Rebaudioside D	HY-N0468
Wilforlide A	HY-N0476
Neoline	HY-N0478
Mogroside III	HY-N0500
Rosin	HY-N0508
Aristolochic acid B	HY-N0511
(+)-Gallocatechin	HY-N0521A
2"-O-Galloylhyperin	HY-N0526
Pseudoginsenoside RT5	HY-N0542

Nomilin	HY-N0547
Isoescin IB	HY-N0557
Alnustone	HY-N0558
Kirenol	HY-N0559
Lathyrol	HY-N0561
5,15-Diacetyl-3-benzoyllathyrol	HY-N0562
Panaxatriol	HY-N0597
alpha-Boswellic acid	HY-N0611
Siamenoside I	HY-N0612
L-Tryptophan	HY-N0623
Kaempferol-7-O-β-D-glucopyranoside	HY-N0627
α-L-Rhamnose monohydrate	HY-N0642
Pinoresinol Diglucoside	HY-N0657
Jujuboside B	HY-N0660
Alliin	HY-N0661
Tenacissoside H	HY-N0670
Asarinin	HY-N0701A
Neomangiferin	HY-N0723
Senkyunolide H	HY-N0744
Oxysophocarpine	HY-N0746
Oxypaeoniflorin	HY-N0748
Monocrotaline	HY-N0750
Acetylcorynoline	HY-N0759
Isobergapten	HY-N0764
Ginkgolide J	HY-N0786
Delsoline	HY-N0789
Tectorigenin	HY-N0792
20(R)-Protopanaxatriol	HY-N0798
Alisol B	HY-N0805A
Anemarsaponin E	HY-N0813
Lithospermoside	HY-N0821
Shionone	HY-N0829
Benzoylhypaconine	HY-N0850
Alisol A 24-acetate	HY-N0853A
Alisol C 23-acetate	HY-N0856
Macranthoidin B	HY-N0864
Sagittatoside A	HY-N0873
Cinobufotalin	HY-N0880
Desacetylcinobufagin	HY-N0881
Telocinobufagin	HY-N0885
Aloin B	HY-N0886
Isoastragaloside I	HY-N0887

Isoastragaloside II	HY-N0888
Tubeimoside II	HY-N0891
Britannilactone	HY-N0895
Wilforine	HY-N0899
Melittoside	HY-N0915
Ligustrazine (hydrochloride)	HY-N0935
Coixol	HY-N0936
(-)-Anomalin	HY-N0947
Triptonoterpene	HY-N10406
Sericoside	HY-N10496
3-p-Coumaroylquinic acid	HY-N10543A
Gigantol	HY-N10549
Herbacetin-3-sophoroside-8--glucoside	HY-N10592
Ethyl rosmarinate	HY-N10610
6-Methoxykaempferol	HY-N10699
Wighteone	HY-N1073
Visnagin	HY-N1082
Viscidulin III	HY-N1083
Barbatic acid	HY-N10981
Torachrysone-8-O-b-D-glucoside	HY-N1141
Tokinolide B	HY-N1145
4-(β-D-glucopyranosyloxy)benzoic acid	HY-N11891
Isolugrandoside	HY-N12084
Smyrindioloside	HY-N1234
α-Ergostenol	HY-N12501
Bartsioside	HY-N12525
Senkyunolide G	HY-N1286
Clinodiside A	HY-N1371
Qingyangshengenin	HY-N1375
Scutellarin methyl ester	HY-N6925
Tussilagone	HY-N1388
Acetagastrodin	HY-N1395
(20R)-Ginsenoside Rh1	HY-N1400
Tigogenin	HY-N1403
Noricaritin	HY-N1413
(E)-3',6-Disinapoylsucrose	HY-N1414
Tiliroside	HY-N1425
L-(+)-Abrine	HY-N1436
Hydroxygenkwanin	HY-N1438
Methyl gypsogenin-3-O-glucuronide	HY-N1439
Complanatuside	HY-N1444
Neonuezhenide	HY-N1449

(S)-Campesterol	HY-N1459A
Aristolochic acid D	HY-N1465
(-?)?-?α-?Terpineol	HY-N1467
Agarotetrol	HY-N1468
Plantagoside	HY-N1470
Liquiritin apioside	HY-N1471
Nicotiflorin	HY-N1475
Polygalic acid	HY-N1479
7beta-Hydroxylathyrol	HY-N1484
Plumbagin	HY-N1497
Methyl deacetylasperulosidate	HY-N1503
Ganoderenic acid C	HY-N1515
Ponasterone A	HY-N1534
Przewaquinone A	HY-N1551
16-Epivoacarpine	HY-N1599
1β-Hydroxybaccatin I	HY-N1628
4',?7-?Di-?O-?methylnaringenin	HY-N1884
Andropanolide	HY-N1912
Crassicauline A	HY-N1924
Protostemotinine	HY-N1955
Syringaresinol diglucoside	HY-N1958
Gibberellic acid	HY-N1964
Sennoside C	HY-N1972
Sennoside D	HY-N1973
Fuziline	HY-N1974
Podophyllotoxin glucoside	HY-N1977
3'-Methoxypuerarin	HY-N1978
4'-Methoxypuerarin	HY-N1979
Platicodigenin	HY-N1991
7-Hydroxyaristolochic acid A	HY-N2012
Hastatoside	HY-N2015
Maoecrystal A	HY-N2017
Parishin	HY-N2031
Paeonol	HY-N0159
Moracin M	HY-122942
Genistin	HY-N0595
Macamide B	HY-N2365
Chenodeoxycholic Acid	HY-76847
Niacin	HY-B0143
Taurine	HY-B0351
Ursolic acid	HY-N0140
Naringin	HY-N0153

(-)-Epigallocatechin	HY-N0225
Bergapten	HY-N0370
Galangin	HY-N0382
Homovanillic acid	HY-N0384
Tetrahydrocurcumin	HY-N0893
Apigenin	HY-N1201
Cephalin form bovine brain	HY-W250118
Piperine	HY-N0144
Daurisoline	HY-N0221
Reserpine	HY-N0480
Isobavachin	HY-N0762
Tomatidine	HY-N2149
Bakuchiol	HY-N0235
Rhoifolin	HY-N0755
Ferulic acid methyl ester	HY-W018643
Cryptotanshinone	HY-N0174
Glycodeoxycholic Acid	HY-125731
Raffinose	HY-N7088
Silybin A	HY-13748
Schisandrin B	HY-N0089
Astragaloside II	HY-N0433
Schisandrol B	HY-N0692
Glucosamine	HY-B1125
Leonurine (hydrochloride)	HY-N0741A
Yangonin	HY-N0919
Norepinephrine (hydrochloride)	HY-13715A
Pregnenolone monosulfate (sodium)	HY-110189
Pregnenolone	HY-B0151
Pregnenolone monosulfate	HY-B1739
Androsin	HY-N1399
Atractyloside (potassium salt)	HY-N1462
Forskolin	HY-15371
Schaftoside	HY-N0703
Icariin	HY-N0014
Eupatilin	HY-N0783
Retinoic acid	HY-14649
Tubeimoside I	HY-N0890
Vinblastine (sulfate)	HY-13780
Vinorelbine (ditartrate)	HY-12053A
Cabazitaxel	HY-15459
Cucurbitacin E	HY-N0417
Guaiol	HY-N3980

4,4'-Dimethoxychalcone	HY-136064
Shogaol	HY-14616
Genipin	HY-17389
Vitexin	HY-N0013
Wogonoside	HY-N0399
Liensinine (Diperchlorate)	HY-N0485
Schisandrin	HY-N0691
Typhaneoside	HY-N0712
Phellodendrine (chloride)	HY-N0735
Syringin	HY-N0824
Pterostilbene	HY-N0828
20-Deoxyingenol	HY-N0866
Corynoxine	HY-N0901
Glaucocalyxin B	HY-N2113
Sedanolide	HY-N2114
Notoginsenoside Fc	HY-N2531
Isorhapontigenin	HY-N2593
Dehydropachymic acid	HY-N2991
Ginkgolide K	HY-N4176
Pennogenin 3-O-beta-chacotrioside	HY-N4180
Palovarotene	HY-14799
SF1670	HY-15842
GW406108X	HY-115570
Resveratrol analog 1	HY-136203
Pentoxifylline	HY-B0715
Obeticholic acid	HY-12222
GW 4064	HY-50108
Cytarabine (hydrochloride)	HY-13605A
Zebularine	HY-13420
6-CEPN	HY-114569
Flavopiridol (Hydrochloride)	HY-10006
Sofalcone	HY-B2184
AS1842856	HY-100596
Autophagy inducer 4	HY-146087
Tangeretin	HY-N0133
Withanolide A	HY-N7028
Osthenol	HY-N2554
Ginsenoside Rg1	HY-N0045
Galanthamine	HY-76299
Scopoletin	HY-N0342
Notoginsenoside R1	HY-N0615
Isatin	HY-Y0265

(E)-Ferulic acid	HY-N0060B
2,6-Dihydroxyacetophenone	HY-Y0106
L-Proline	HY-Y0252
Gingerenone A	HY-120912
(Rac)-Salvianic acid A (sodium)	HY-N0106
Dauricine	HY-N0220
L-Methionine	HY-N0326
18α-Glycyrrhetinic acid	HY-N0375
Ursonic acid	HY-N1486
Cynaropicrin	HY-N2350
Kamebakaurin	HY-N6046
Se-Methylselenocysteine	HY-114245
Rosmarinic acid	HY-N0529
4-Hydroxyderricin	HY-N7204
Coenzyme Q9	HY-101415
9-Methoxycanthin-6-one	HY-112642
Delta-Tocopherol	HY-113026
Myristoleic acid	HY-113332
trans-Vaccenic acid	HY-113427
Stachyose (tetrahydrate)	HY-113529
(-)-Epigallocatechin Gallate	HY-13653
D-Pantothenic acid (sodium)	HY-B0430A
Deoxyarbutin	HY-B1461
Stearic acid	HY-B2219
2'-Deoxyuridine	HY-D0186
Geniposidic acid	HY-N0010
Costunolide	HY-N0036
Betulin	HY-N0083
Beta-Sitosterol (purity>98%)	HY-N0171A
Ecdysone	HY-N0179
Lobetyolin	HY-N0327
1,4-Dicaffeoylquinic acid	HY-N0358
Wedelolactone	HY-N0551
Ginsenoside Rh2	HY-N0605
Trifolirhizin	HY-N0616
Notoginsenoside Ft1	HY-N0910
Sorbifolin	HY-N11552
Tomatine	HY-N2166
3-Dehydrotrametenolic acid	HY-N2177
Aloesin	HY-N2460
Desoxyrhaponticin	HY-N2486
Ginsenoside F4	HY-N2503

4-Methylesculetin	HY-N4288
Homovanillyl alcohol	HY-N7513
Malabaricone B	HY-N8517
Maltol	HY-W012788
Rutin (trihydrate)	HY-W013075
Butylated hydroxytoluene	HY-Y0172
Benzophenone	HY-Y0546
4-Hydroxybenzyl alcohol	HY-Y0892
1-Monopalmitin	HY-W009141
(-)-Huperzine A	HY-17387
(E)-Cardamonin	HY-N1378
Cycleanine	HY-N2005
14-Deoxyandrographolide	HY-N4323
Uridine 5'-monophosphate	HY-101981
L-Glutamic acid	HY-14608
L-Glutamic acid (monosodium salt)	HY-14608A
Oleic acid	HY-N1446
Chrysosplenetin	HY-N1457
Solamargine	HY-N0069
Sodium oleate	HY-N1446B
Rotundic acid	HY-N2217
Higenamine (hydrochloride)	HY-N2037A
Ononin	HY-N0270
Saponins	HY-100597
Arcyriaflavin A	HY-103382
Pyrethrin II	HY-108170
Polygodial	HY-108450
Petroselinic acid	HY-113362
Ascaridole	HY-118494
Chalcone	HY-121054
Ledol	HY-121388
Piperlonguminine	HY-126562
Linustatin	HY-129385
Colchicoside	HY-131300
(+)-Neomenthol	HY-135286A
Dieckol	HY-147059
Helioxanthin	HY-16678
2-Phenylethyl isothiocyanate	HY-23155
2'-Hydroxy-4'-methylacetophenone	HY-34204
Isoegomaketone	HY-46866
trans-4-Hydroxycyclohexanecarboxylic acid	HY-76199
Methyl indole-3-carboxylate	HY-79635

Azulene	HY-B0055
1-Docosanol	HY-B0222
1R-cis-Permethrin	HY-B0887C
Ethylparaben	HY-B0934
(+)-Camphor	HY-B1173
Urethane	HY-B1207
2-Phenylethanol	HY-B1290
Ethyl Vanillate	HY-B1643
Santonin	HY-B1761
Methyl stearate	HY-B1934
Lactate (potassium),60% in water	HY-B2227C
Glycitin	HY-N0012
Punicalagin	HY-N0063
Betulinaldehyde	HY-N0084
Hordenine	HY-N0113
Sclareolide	HY-N0129
Glycyrrhizic acid	HY-N0184
Dipotassium glycyrrhizinate	HY-N0184A
Ardisiacrispin A	HY-N0206
Patchouli alcohol	HY-N0207
Epigoitrin	HY-N0224
Saikosaponin B2	HY-N0248
Hederasaponin B	HY-N0306
Allicin	HY-N0315
DL-Methionine	HY-N0325
(+)-Magnoflorine (iodide)	HY-N0334A
3-Butylidenephthalide	HY-N0336
Tuberostemonine	HY-N0352
Dihydrotanshinone I	HY-N0360
(+)-Columbianetin	HY-N0363
(+)-Columbianetin acetate	HY-N0363A
Sennoside A	HY-N0365
Harpagide	HY-N0397
Artemether	HY-N0402
Deoxycorticosterone	HY-113414
DHEA	HY-14650
Ethinylestradiol	HY-B0216
Estrone	HY-B0234
Estriol	HY-B0412
Progesterone	HY-N0437
Gypenoside XVII	HY-N0553
Pinoresinol 4-O-β-D-glucopyranoside	HY-N2168

Neoruscogenin	HY-N2253
Iristectorigenin B	HY-N2509
4-Ethylresorcinol	HY-W015782
Cyclopamine	HY-17024
Ferulic acid	HY-N0060
Taurocholic acid (sodium)	HY-N0545
2,5-Dihydroxybenzoic acid	HY-W001179
1-Deoxynojirimycin	HY-14860
L-Leucine	HY-N0486
Creatine (monohydrate)	HY-W017462
Stearamide	HY-W130610
Brassinin	HY-111334
Uracil	HY-I0960
Phenylglyoxylic acid	HY-W010255
Cryptochlorogenic acid	HY-N0787
γ-Tocotrienol	HY-108694
Pyridoxine	HY-B1328
Sarsasapogenin	HY-N0073
Stachydrine	HY-N0298
Nordihydrocapsaicin	HY-N0449
Tyrosol	HY-N0474
Ginsenoside Rh3	HY-N0606
Pyridoxine (hydrochloride)	HY-N0682
Stachydrine hydrochloride	HY-N0738
4-Hydroxyphenylacetic acid	HY-N1902
5-Methyl-7-methoxyisoflavone	HY-N1993
Benzoyloxypaeoniflorin	HY-N2101
Nervonic acid	HY-N2526
Damascenone	HY-N2543
Quercetagitrin	HY-N4150
4-Phenyl-7,8-dihydroxycoumarin	HY-128410
5-Hydroxyferulic acid	HY-133068
D-(-)-Quinic acid	HY-N0464
(-)-Gallocatechin gallate	HY-N0522
Allantoin	HY-N0543
Polygalacic acid	HY-N0801
Norharmane	HY-W008566
Melatonin	HY-B0075
Chelerythrine (chloride)	HY-12048
Icaritin	HY-N0678
6-Demethoxytangeretin	HY-N4126
Corosolic acid	HY-N0280

Anthraquinone-2-carboxylic acid	HY-W031757
Gypsogenin	HY-121382
Sakuranetin	HY-N3006
Mollugin	HY-N0316
Ellagic acid	HY-B0183
Scutellarein tetramethyl ether	HY-N4314
Atractylenolide II	HY-N0202
Taxifolin	HY-N0136
Piceatannol	HY-13518
Formononetin	HY-N0183
Brevilin A	HY-N2959
Ponicidin	HY-N1535
Curculigoside B	HY-N7646
N-(p-Coumaroyl) Serotonin	HY-129440
Guanfu base A	HY-N1483
Sophocarpine	HY-N0103
(20R)-Ginsenoside Rg3	HY-N1376
Curcumol	HY-N0104
Biochanin A	HY-14595
Tannic acid	HY-B2136
Daphnetin	HY-N0281
20(R)-Ginsenoside Rh2	HY-N1401
Protosappanin A	HY-113573
Butein	HY-16558
Genistein	HY-14596
Ganoderic acid B	HY-N2006
Cyasterone	HY-N0211
Furanodienone	HY-N2184
(Rac)-Norcantharidin	HY-N0585
Methyl 2,5-dihydroxycinnamate	HY-101006
Chrysophanol	HY-13595
Khellin	HY-B1394
Palmatine (hydroxide)	HY-N0110B
Anthraquinone	HY-N0354
Cynaroside	HY-N0540
Febrifugine	HY-N2384
2'-O,4'-C-Methyleneadenosine	HY-110407
5'-O-TBDMS-dG	HY-138598
5'-O-TBDMS-dA	HY-138599
5'-O-DMT-N2-DMF-dG	HY-138607
5'-O-DMT-rI	HY-138608
5-BrdU	HY-15910

Ethynylcytidine	HY-16200
2'-O,4'-C-Methyleneguanosine	HY-W406070
5-Methoxyflavone	HY-107790
Sorivudine	HY-123032
N-Dodecyl-β-D-maltoside	HY-128974
And1-IN-1	HY-150184
Thermospermine	HY-151224
Ac-rG	HY-164248
2-Fluoroadenine	HY-W008469
5'-O-DMT-N2-ibu-dG	HY-W010702
5-Fluorocytidine	HY-W039722
2'-O-MOE-5-Me-rU	HY-W048488
2′-O-(2-Methoxyethyl)adenosine	HY-W048491
2'-O-MOE-5-Me-rC	HY-W048497
1,4-Anthraquinone	HY-W077242
N4-Acetyl-2'-O-methylcytidine	HY-W114327
Homoharringtonine	HY-14944
Picroside I	HY-N0407
Casticin	HY-N0516
3'-Demethylnobiletin	HY-N4127
SU5204	HY-126319
SU-4313	HY-21291
β-Hydroxyisovalerylshikonin	HY-N4201
Tectochrysin	HY-14592
Triacetylresveratrol	HY-N1410
2,4,5-Trimethoxybenzoic acid	HY-Y0586
Corylifol A	HY-N0897
Mogrol	HY-N2312
Hispidulin	HY-N1950
Quercetagetin	HY-N4149
Isoquercitrin	HY-N1445
Echinacoside	HY-N0020
Methyl vanillate	HY-75342
DMU-212	HY-137977
Astragaloside IV	HY-N0431
7β-Hydroxycholesterol	HY-113341
Oridonin	HY-N0004
Bicyclol	HY-B0766
Ligustroflavone	HY-N0546
Pterosin B	HY-N1570
D-Pantothenic acid (hemicalcium salt)	HY-N0681
3,4-Dimethoxyphenol	HY-N1780

Aloe-emodin-8-O-β-D-glucopyranoside	HY-N2451
Monobutyl phthalate	HY-N7143
Trithiozine	HY-108287
L-Fucitol	HY-N4112
N-Benzyloctadecanamide	HY-N4188
Karacoline	HY-N6812
Isorhamnetin-3-O-neohespeidoside	HY-N0778
Mirificin	HY-N2134
Melilotic acid	HY-W017158
cis-Verbenol	HY-W674037
Silychristin	HY-N0647
9,10-Dihydroxystearic acid	HY-N8522
Syringetin	HY-N8920
Cimicifugoside H-1	HY-N9331
Linoleyl alcohol	HY-W005627
Harmine	HY-N0737A
(-)-Isocorypalmine	HY-N0927
Atropine (sulfate monohydrate)	HY-B0394
Meranzin	HY-N3298
Anisodamine	HY-N0584
Spiramide	HY-100971
Gentisein	HY-118166
Rotundine	HY-N0096
Tetrahydropalmatine (hydrochloride)	HY-N0300A
Sec-O-Glucosylhamaudol	HY-N0398
L-Hyoscyamine	HY-N0471
L-Hyoscyamine (sulfate)	HY-N0471A
Isocorynoxeine	HY-N0775
Tetrahydroberberine	HY-N0925
Racanisodamine	HY-N2064
Kainic acid (hydrate)	HY-N2309A
Pyocyanin	HY-111278
Cynarin	HY-N0359
Squalene	HY-N1214
Gibberellin A7	HY-125572
4-Aminobenzoic acid	HY-B1008
Octyl gallate	HY-N2011
3,5-Di-tert-butylphenol	HY-W041080
Chlorogenic acid	HY-N0055
Methyl gallate	HY-N2010
3,4-Dimethoxycinnamic acid	HY-N1778
(E)-3,4-Dimethoxycinnamic acid	HY-N1778A

Psoralidin	HY-N0232
10-Undecenoic acid,98% (stabilized with TBC)	HY-B0914
D(+)-Raffinose (pentahydrate)	HY-N1938
(E)-Osmundacetone	HY-N1966
Tenuifoliside A	HY-N6076
L-Quebrachitol	HY-N2375
Magnolin	HY-N1374
Corynoxeine	HY-N0590
Citropten	HY-N7085
Methylnissolin	HY-N2484
α-Amyrin	HY-N8423
Pachymic acid	HY-N0371
2,3,5,4'-Tetrahydroxystilbene 2-O-β-D-glucoside	HY-N0652
2,5-Dihydroxyacetophenone	HY-W001174
Ricinine	HY-121944
Raspberry ketone	HY-N1426
trans-Cinnamyl alcohol	HY-N1867
Oroxin A	HY-N2025
Angeloylgomisin H	HY-N2209
Gypenoside XLIX	HY-N1990
Daidzein	HY-N0019
Cephradine	HY-B1156
4-O-Methyl honokiol	HY-U00450
Eleutheroside E	HY-N0272
Astilbin	HY-N0509
Boldine	HY-N6973
Chelidonic acid	HY-W041489
Forsythoside B	HY-N0029
Barlerin	HY-N0758
Homoplantaginin	HY-N1949
DL-alpha-Tocopherol	HY-W020044
Methyl 3,4-dihydroxybenzoate	HY-Z0548
Isovitexin	HY-N0773
Ethyl pyruvate	HY-Y1362
Geraniin	HY-N0472
Oxysophoridine	HY-N1402
Loureirin A	HY-N1505
Esculetin	HY-N0284
Heterophyllin B	HY-N1476
Dihydroevocarpine	HY-N2517
24-Methylenecycloartanyl ferulate	HY-N8122
p-Hydroxycinnamic acid	HY-N2391

Mequinol	HY-30270
Diethyl phthalate	HY-Y0284
Terbutaline	HY-B0802A
3',4'-Dimethoxyflavone	HY-N8572
Anacardic Acid	HY-N2020
Deoxyshikonin	HY-N2187

Supplementary Table 4. The small molecules inhibitor information of library.

MNS	HY-78263
2-D08	HY-114166
Tomatine	HY-N2166
GS143	HY-110261
Xenin 8 acetate	HY-P1257
Vimseltinib	HY-136256
UNC569	HY-117596
NBI-31772	HY-110135
TAM-IN-2	HY-126216
PKI 14-22 amide, myristoylated Acetate	HY-P1291
PKG Substrate acetate	HY-P1561
NVP-BHG712 isomer	HY-13258
Linsitinib	HY-10191
MK-8033	HY-13299
LOXO-195	HY-101977
JNJ-38877618	HY-111050
HNMPA	HY-124097
Cyclotraxin B acetate	HY-P1178
VEGFR-IN-1	HY-101219
CE-245677	HY-112423
Bozitinib	HY-125017
BMS-777607	HY-12076
AWL-II-38.3	HY-18832
Zotarolimus	HY-12424
YKL-06-061	HY-120056
YKL-05-099	HY-101147
VS-5584	HY-16585
VPS34 inhibitor 1	HY-12794
Umbralisib	HY-12279
UCB9608	HY-112613
TMBIM6 antagonist-1	HY-137175
TGX-221	HY-10114

TG100-115	HY-10111
TG 100713	HY-13514
Tenalisib	HY-17645
Serabelisib	HY-12285
Seletalisib	HY-16754
Selective PI3Kδ Inhibitor 1	HY-15288
PS210	HY-121629
PQR620	HY-100026
PQR530	HY-107365
Pilaralisib	HY-16526
PIK-294	HY-10303
PIK-293	HY-13504
PI4KIIIbeta-IN-9	HY-19798
PI4KIIIbeta-IN-10	HY-100198
PF-04979064	HY-100398
OTSSP167 hydrochloride	HY-15512A
Osu03012	HY-10547
NSC781406	HY-100470
NIH-12848	HY-101423
MP7	HY-14440
LY-294002 hydrochloride	HY-10108A
Linperlisib	HY-102031
KU-0063794	HY-50710
JR-AB2-011	HY-122022
IPI-3063	HY-111510
IC-87114	HY-10110
HG-9-91-01	HY-15776
Heterophyllin B	HY-N1476
GSK2636771	HY-15245
GSK2334470	HY-14981
GNE-493	HY-10811
GNE-477	HY-11042
GNE-317	HY-12763
Gedatolisib	HY-10681
GDC-0326	HY-101272
GDC0084	HY-19962
ETP-46321	HY-12340
Eganelisib	HY-100716
Duvelisib	HY-17044
AKT Kinase Inhibitor	HY-10249A
Desmethyl-VS-5584	HY-101776A
CZC24832	HY-15294
CZ415	HY-100222

CNX-1351	HY-16596
CH5132799	HY-15466
CAL-101	HY-13026
AZD-8835	HY-12869
AZD8186	HY-12330
AS-604850	HY-13531
AS-041164	HY-118521
ARN-3236	HY-120856
AMG319	HY-12948
Alpelisib	HY-15244
Acalisib	HY-12644
A66	HY-13261
(2S,3R,4S)-4-Hydroxyisoleucine	HY-W010271
N-(4-fluorophenyl)benzo[d]thiazol-2-amine	HY-108896
LP-935509	HY-117626
GSK-1520489A	HY-147312
GSK-114	HY-117658
GCN2-IN-1	HY-100877
BT173	HY-128439
BMS-986176	HY-134829
TBK1/IKKε-IN-5	HY-128679
TBK1/IKKε-IN-2	HY-12453
NIK SMI1	HY-112433
NF-κB-IN-1	HY-138537
MLN120B	HY-15473
JTP 0819958 - HOIPIN-1	HY-122881
INH14	HY-114454
IMD-0560	HY-105661
GSK8612	HY-111941
BOT-64	HY-136741
BMS-345541 hydrochloride	HY-10518
BMS-345541	HY-10519
BAY-985	HY-133117
AZD3264	HY-19362
DCP-LA	HY-108599
Syntide 2 acetate(108334-68-5 free base)	HY-P0271A
CALP1	HY-P1077
Autocamtide 2	HY-P0225
YZ9	HY-110156
Tryptophan	HY-N0623R
Terphenyllin	HY-119821
SR3335	HY-14413
SR0987	HY-101454

phosphate dibasic	HY-D0885B
SI-113	HY-117357
SGK1-IN-4	HY-142687
S18-000003	HY-119366
Prudomestin	HY-N1547
Pinoresinol diglucoside	HY-N0657
N-Caffeoyl O-methyltyramine	HY-N7203
MK-0941 free base	HY-19843A
M2N12	HY-128769
Kaempferol 3-O-gentiobioside	HY-N1510
IACS-13909	HY-137092
GSK805	HY-12776
EMD638683	HY-15193
DL-Serine	HY-Y0507
Conduritol B epoxide	HY-100944
Cintirorgon	HY-104037
Castanospermine	HY-N2022
Butyl isobutyl phthalate	HY-N7377
Bevurogant	HY-132810
AZ PFKFB3 26	HY-101971
AP-III-a4	HY-15858
AMG-3969	HY-12411
4',5-Dihydroxyflavone	HY-N1881
3-Oxo-5β-cholanoic acid	HY-125801
β,β-Dimethylacrylshikonin	HY-N5112B
XMD17-109	HY-15665
Ulixertinib	HY-15816
Tenuifoliside A	HY-N6076
Temuterkib	HY-101494
TAK-733	HY-13449
TAK-580	HY-15246
SLV-2436	HY-112113
SCH772984	HY-50846
SB-590885	HY-10966
Ro 5126766	HY-18652
Ravoxertinib	HY-15947
RAF709	HY-100510
Raf inhibitor 2	HY-109574
Raf inhibitor 1	HY-14177
PLX8394	HY-18972
PLX7904	HY-18997
PLX-4720	HY-51424
Pimasertib	HY-12042

PF-06260933	HY-19562
PD318088	HY-12062
Nomilin	HY-N0547
Necrosulfonamide	HY-100573
MK2-IN-1 hydrochloride	HY-12834A
magnolin	HY-N1374
K-Ras(G12C) inhibitor 9	HY-12446
K-Ras(G12C) Inhibitor 6	HY-107841
JNK-IN-7	HY-15617
GDC-0879	HY-50864
ERK5-IN-1	HY-14403
Encorafenib	HY-15605
DB07268	HY-15737
Dabrafenib	HY-14660
Corynoxeine	HY-N0590
BMS582949	HY-14305A
BI-78D3	HY-10366
Bentamapimod	HY-14761
AZD8330	HY-12058
Avicularin	HY-N0222
AS601245	HY-11010
APS-2-79 hydrochloride	HY-100627
5,6,7-TRIMETHOXYFLAVONE	HY-110398
VAF347	HY-135750
SGA360	HY-122208
Pyrazoloadenine	HY-34595
Ophiopogonin-D	HY-N0515
ITE	HY-19317
GNF351	HY-102023
Carminic acid	HY-N8407
Blumeatin	HY-N2358
BAY-218	HY-111449
BAY 2416964	HY-135829
8-Epideoxyloganic acid	HY-N2772
CGS 15943	HY-100678
Norisoboldine	HY-N0586A
Meranzin hydrate	HY-N3297
Xanthotoxol	HY-30152
Cannabigerol	HY-137961
Phenoxybenzamine	HY-B0431A
ABT-702	HY-103161
5-Iodotubercidin	HY-15424
kuwanon G	HY-N4247

ZT-12-037-01	HY-122866
Ketoconazole	HY-B0105B
Atranorin	HY-N2907
XRP44X	HY-107753
Sotorasib racemate	HY-114277A
Sotorasib	HY-114277
SCH54292	HY-124161
RBC8	HY-12873
Rasarfin	HY-139950
Pan-RAS-IN-1	HY-101295
MRTX-1257	HY-114436
MRTX1133	HY-134813
K-Ras-IN-1	HY-18674
K-Ras G12C-IN-4	HY-128771
HJC0197	HY-117958
pan-KRAS-IN-16	HY-164645
CID-1067700	HY-13452
BQU57	HY-12875
BAY-293	HY-114398
ARS-1630	HY-U00417
ARS-1620	HY-U00418
APS6-45	HY-124944
Adagrasib	HY-130149
6H05 (TFA)	HY-12408A
BODIPY-X-Alkyne	HY-D1588
LS-BF1	HY-P3350
Raloxifene hydrochloride	HY-13738A
Zofenopril calcium	HY-B0655
Norgestrel	HY-N7137
KY19382	HY-131447
IWP-2	HY-13912
Gigantol	HY-N10549
Echinacoside	HY-N0020
TNIK-IN-2	HY-145292
KY-05009	HY-124745
GSK1324726A	HY-13960
CPI-203	HY-15846
GNF2133	HY-142295
CLK-IN-T3	HY-115470
HQ461	HY-144981
IPR-803	HY-111192
Olomoucine	HY-W011428
ROCK2-IN-2	HY-103620

AAPK-25	HY-126249
SP-96	HY-131339
GW843682X	HY-11003
Hydroxyfasudil	HY-13911
AT13148	HY-16071
CKI-7	HY-133028
GSK269962A	HY-15556
GSK-25	HY-14362
GSK180736A	HY-18990
CCG-222740	HY-121750
Afuresertib hydrochloride	HY-15727A
Afuresertib	HY-15727
Y-33075	HY-10069
CMPD101	HY-103045
ZINC00881524	HY-101244
Thiazovivin	HY-13257
SAR407899 hydrochloride	HY-15687
SAR407899	HY-15687A
ROCK-IN-2	HY-10319
ROCK inhibitor-2	HY-119937
RKI-1447	HY-15755
RKI1313	HY-107209
Ripasudil	HY-15685
Netarsudil hydrochloride	HY-12798B
Hydroxyfasudil	HY-13911A
GSK429286A	HY-11000
Chroman 1	HY-15392
Belumosudil	HY-15307
BDP5290	HY-12437
Mps1-IN-2	HY-13994
AMG 900	HY-13253
SC-514	HY-13802
XL413 hydrochloride	HY-15260
A-674563	HY-13254
Desmethylglycitein	HY-N5072
CD532	HY-112273
SNS-314	HY-12003
Phthalazinone pyrazole	HY-12564
NU6140	HY-107419
Centrinone	HY-18682
CCT129202	HY-12049
Bromosporine	HY-15815
Aurora kinase inhibitor-3	HY-112373

Aurora kinase inhibitor-2	HY-112355
AKI603	HY-123159
ZN-c3	HY-132295
YKL-5-124	HY-101257
WEE1-IN-3	HY-138239
CLK1/2-IN-1	HY-113825
Trilaciclib hydrochloride	HY-101467A
Tricin	HY-N1127
CDK7-IN-2	HY-143587
THZ531	HY-103618
THZ2	HY-12280
THZ1	HY-80013
THAL-SNS-032	HY-123937
TH-257	HY-122630
TAK-960	HY-15160
T56-LIMKi	HY-19352
SU-9516	HY-18629
STF-083010	HY-15845
SRI-29329	HY-123600
Simurosertib	HY-100888
Senexin B	HY-101800
Senexin A	HY-15681
Seliciclib	HY-30237
SCH900776 (S-isomer)	HY-15532B
SCH900776	HY-15532
SAR-020106	HY-100195
Ro3280	HY-15161
Ribociclib succinate	HY-15777B
Poloxin	HY-12134
PNU112455A hydrochloride	HY-112468
PF-06873600	HY-114177
Palbociclib monohydrochloride	HY-50767A
Palbociclib Isethionate	HY-A0065
Palbociclib	HY-50767
ON-01910	HY-12037A
ON-013100	HY-112822
P18IN003	HY-123535
NSC23005	HY-100791
NG 52	HY-15154
MSC2530818	HY-101611
MLN0905	HY-15155
MKC9989	HY-12399
MKC8866	HY-104040

MKC3946	HY-19710
MC180295	HY-119940
CDK7-IN-20	HY-151878
LY3177833	HY-100023
Cdc7-IN-1	HY-101523
LY2880070	HY-148962
LX7101	HY-12659
Lerociclib dihydrochloride	HY-112272A
LDC4297	HY-12653
Kira8	HY-114368
KIRA6	HY-19708
KB-0742	HY-137478A
JSH-150	HY-X0150
IRE1α kinase-IN-1	HY-136735
HMN-214	HY-12045
HMN-176	HY-13647
GSK461364	HY-50877
GSK2850163	HY-U00459
GDC0575 monohydrochloride	HY-112167A
GDC-0575	HY-112167B
Fadraciclib	HY-101212
Eciruciclib	HY-145563
Dalpiciclib	HY-114338
CVT-313	HY-15339
CK7	HY-103646
CFI-400945	HY-12300B
CDK9-IN-2	HY-16462
Cdk5 Substrate acetate	HY-P2668
CDK4/6-IN-15	HY-142076
CDK4/6-IN-2	HY-114339
CDK2-IN-4	HY-169910
CCT-251921	HY-19984
CCT245737	HY-18958
CC-671	HY-108709
CAN508	HY-100429
BUR1	HY-121025
BSJ-03-123	HY-111556
BS194	HY-14372
BS-181 hydrochloride	HY-13266A
BMS-5	HY-18305
BMS-3	HY-18304
BMS-265246	HY-15275
BML-259	HY-108348

BI-1347	HY-120350
AZD-7762	HY-10992
AZD-5597	HY-50914
AZD-5438	HY-10012
AUZ 454	HY-15004
Atuveciclib	HY-12871
APY29	HY-17537
Amsilarotene	HY-14653
Aminopurvalanol A	HY-104013
Adavosertib	HY-10993
Abemaciclib methanesulfonate	HY-16297
Abemaciclib metabolite M20	HY-129336
Abemaciclib	HY-16297A
6-Bromo-2-hydroxy-3-methoxybenzaldehyde	HY-107371
2-Amino-4-(4-pyridyl)-thiazole	HY-W044805
4μ8C	HY-19707
3MB-PP1	HY-102069
CDK2-IN-30	HY-164664
(1E)-CFI-400437	HY-132135
(±)-Enitociclib	HY-103019A
Dacinostat	HY-13606
Dorsomorphin	HY-13418A
Metformin hydrochloride	HY-17471A
BGT226	HY-13334A
Autogramin-1	HY-128339
STO-609	HY-19805
LX2343	HY-111383
CaMKII-IN-1	HY-18271
Calmidazolium chloride	HY-103319
YM-201636	HY-13228
A-484954	HY-110096
Emodin	HY-14393
Silmitasertib sodium salt	HY-50855B
Silmitasertib	HY-50855
3-Methyladenine	HY-19312
Tacrolimus	HY-13756
SJFα	HY-114404
Isobavachin	HY-N0762
p38α inhibitor 1	HY-114423
Bakuchiol	HY-N0235
MW-150	HY-120111
TA-02	HY-100115
Doramapimod	HY-10320

Neflamapimod	HY-10328
AZD7624	HY-103672
VX-702	HY-10401
Losmapimod	HY-10402
LY3009120	HY-12558
URMC-099	HY-12599
Pamapimod	HY-10405
L-779450	HY-12787
AL 8697	HY-108645
Rapamycin	HY-10219
PFKFB3-IN-2	HY-153077
PFK-015	HY-12204
KAN0438757	HY-112808
3PO	HY-19824
KN-62	HY-13290
Nifedipine	HY-B0284
Pexmetinib	HY-16782
AZ304	HY-117273
H-89 dihydrochloride	HY-15979A
PF-4708671	HY-15773
BI-D1870	HY-10510
TOMATIDINE HYDROCHLORIDE	HY-N2149A
Tomatidine	HY-N2149
U0126-EtOH	HY-12031
PD 169316	HY-10578
TA-01	HY-100114
Rhoifolin	HY-N0755
Galangin	HY-N0382
Glycycoumarin	HY-N4113
SD 0006	HY-11087
PH-797804	HY-10403
Dilmapimod	HY-10404
Adezmapimod	HY-10256
Acumapimod	HY-16715
RSVA405	HY-103238
Ginsenoside Rb1	HY-N0039
Rhein	HY-N0105
Pinocembrin	HY-N0575
Mefloquine hydrochloride	HY-17437A
Quercitrin	HY-N0418
Tempol	HY-100561
Silibinin	HY-N0779A
Schisandrol B	HY-N0692

PD98059	HY-12028
Trifluoperazine dihydrochloride	HY-B0532A
Trifluoperazine	HY-B0532
Paroxetine hydrochloride	HY-B0492
Peretinoin	HY-100008
Lonafarnib	HY-15136
Salirasib	HY-14754
Schisandrin B	HY-N0089
Torin 1	HY-13003
Samotolisib	HY-12513
OSI-027	HY-10423
KU-55933	HY-12016
Dactolisib	HY-50673
AZD 6482	HY-10344
Berberine chloride	HY-18258
Berberine	HY-N0716
CHIR-99021	HY-10182
Capivasertib	HY-15431
ULK-101	HY-114490
sbp-7455	HY-137742
PFE-360	HY-120085
PF-06454589	HY-112855
PF-06447475	HY-12477
MRT68921 HCl	HY-100006
MRT68921 dihydrochloride	HY-100006A
MLi-2	HY-100411
JH-II-127	HY-16936
Hydroxyprogesterone caproate	HY-B0742
HG-10-102-01	HY-13488
GSK2578215A	HY-13237
GNE-9605	HY-12282
GNE-7915	HY-18163
GNE0877	HY-15796
GCN2iB	HY-112654
EB-42486	HY-142647
CZC-54252 hydrochloride	HY-B0792A
CZC-54252	HY-B0792
CZC-25146 hydrochloride	HY-15800
CZC-25146	HY-15800A
Oroxin B	HY-N1435
BAY 11-7082	HY-13453
Bardoxolone Methyl	HY-13324
Cordycepin	HY-N0262

Valinomycin	HY-N6693
Myricetin	HY-15097
Salubrinal	HY-15486
PFK-158	HY-12203
3-Bromopyruvic acid	HY-19992
Pseudolaric Acid B	HY-N6939
Trametinib	HY-10999
SB 202190	HY-10295
Ralimetinib dimesylate	HY-13241
Mirdametinib	HY-10254
Sodium salicylate	HY-B0167A
Quercetin	HY-18085
(-)-Epigallocatechin Gallate	HY-13653
α-Thujone	HY-121618R
Piperlongumine	HY-N2329
TPEN	HY-100202
Mito-LND	HY-134832
Hesperidin	HY-15337
Cysteamine hydrochloride	HY-77591
Bigelovin	HY-116506
TBHQ	HY-100489
Hesperetin	HY-N0168
Decursin	HY-18981
PF-543 hydrochloride	HY-15425
Melatonin	HY-B0075
LY294002	HY-10108
Torin 2	HY-13002
Pictilisib dimethanesulfonate	HY-20180
PI-103	HY-10115
CCT128930 hydrochloride	HY-13260A
Vistusertib	HY-15247
Polyphyllin I	HY-N0047
Actein	HY-N6872
Isobavachalcone	HY-13065
Sophocarpine monohydrate	HY-N0103A
Perifosine	HY-50909
MK-2206 dihydrochloride	HY-10358
Deguelin	HY-13425
Tanespimycin	HY-10211
OSU-T315	HY-18676
Glucosamine	HY-B1125
Ethyl 3,4-dihydroxybenzoate	HY-W016409
AZD1208	HY-15604

Rottlerin	HY-18980
Enzastaurin	HY-10342
Chelerythrine chloride	HY-12048
Chelerythrine	HY-N2359
PFI-1	HY-16586
Urolithin A	HY-100599
Resveratrol	HY-16561
Wogonin	HY-N0400
SP600125	HY-12041

Supplementary Table 5. MAP of Phosphorylation Antibody Array.

	A	B	C	D	E	F	G	H	I	J	K	L
1	POS1	POS1	POS2	POS2	POS3	POS3	ABL1	ABL1	ACK1	ACK1	ALK	ALK
2	NEG	NEG	NEG	NEG	Axl	Axl	Blk	Blk	BMX	BMX	Btk	Btk
3	Csk	Csk	Dtk	Dtk	EGFR	EGFR	EphA1	EphA1	EphA2	EphA2	EphA3	EphA3
4	EphA4	EphA4	EphA5	EphA5	EphA6	EphA6	EphA7	EphA7	EphA8	EphA8	EphB1	EphB1
5	EphB2	EphB2	EphB3	EphB3	EphB4	EphB4	EphB6	EphB6	ErbB2	ErbB2	ErbB3	ErbB3
6	ErbB4	ErbB4	FAK	FAK	FER	FER	FGFR1	FGFR1	FGFR2	FGFR2	FGFR2 (α isoform)	FGFR2 (α isoform)
7	Fgr	Fgr	FRK	FRK	Fyn	Fyn	Hck	Hck	HGFR	HGFR	IGF-IR	IGF-IR
8	Insulin R	Insulin R	Itk	Itk	JAK1	JAK1	JAK2	JAK2	JAK3	JAK3	LCK	LCK
9	LTK	LTK	Lyn	Lyn	MATK	MATK	M-CSFR	M-CSFR	MUSK	MUSK	NGFR	NGFR
10	PDGFR-α	PDGFR-α	PDGFR-ß	PDGFR-ß	PYK2	PYK2	RET	RET	ROR1	ROR1	ROR2	ROR2
11	ROS	ROS	RYK	RYK	SCFR	SCFR	SRMS	SRMS	SYK	SYK	Tec	Tec
12	Tie-1	Tie-1	Tie-2	Tie-2	TNK1	TNK1	TRKB	TRKB	TXK	TXK	NEG	NEG
13	Tyk2	Tyk2	TYRO10	TYRO10	VEGFR2	VEGFR2	VEGFR3	VEGFR3	ZAP70	ZAP70	POS4	POS4

POS = Positive Control Spot
NEG = Negative Control Spot
BLANK = Blank Spot

Supplementary Table 6. Clinicopathological characteristics of ECCpatient used for PDX.

Gender	Age	FIGO 2023	TNM	Pathological grading	histological subtypes	Molecular Classification
Female	53	IIIC2m	T3cN1M0	G2	Serous	P53abn
